# Deoxygenative α-alkylation and α-arylation of 1,2-dicarbonyls†

**DOI:** 10.1039/d0sc03118f

**Published:** 2020-07-01

**Authors:** Shengfei Jin, Hang T. Dang, Graham C. Haug, Viet D. Nguyen, Hadi D. Arman, Oleg V. Larionov

**Affiliations:** Department of Chemistry, The University of Texas at San Antonio One UTSA Circle San Antonio TX 78249 USA oleg.larionov@utsa.edu

## Abstract

Construction of C–C bonds at the α-carbon is a challenging but synthetically indispensable approach to α-branched carbonyl motifs that are widely represented among drugs, natural products, and synthetic intermediates. Here, we describe a simple approach to generation of boron enolates in the absence of strong bases that allows for introduction of both α-alkyl and α-aryl groups in a reaction of readily accessible 1,2-dicarbonyls and organoboranes. Obviation of unselective, strongly basic and nucleophilic reagents permits carrying out the reaction in the presence of electrophiles that intercept the intermediate boron enolates, resulting in two new α-C–C bonds in a tricomponent process.

## Abstract 2

α-Branched carboxylic acids and other carbonyls are readily accessed by a metal- and base-free deoxygenative α-alkylation and α-arylation of 1,2-dicarbonyls *via* boron enolates, resulting in a tricomponent coupling with unconventional electrophiles.

## Graphical abstract

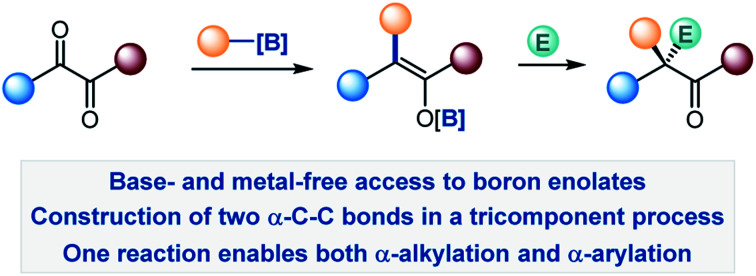

## Introduction

Chemists have long relied on reactions that allow for generation of unstable and reactive intermediates but forcing conditions, as well as strong and unselective reagents are often required to achieve the desired transformations. Much of the recent progress in synthetic methodology has been fueled by the necessity to access transient reactive intermediates in a rapid and modular manner and in the absence of unselective reagents.^1^

Enolates are among the most common and synthetically versatile intermediates used for α-functionalization of carbonyls. Despite their broad utility, the most common method of generation of enolates remains deprotonation with strong bases, *e.g.*, organolithiums, lithium amides and alkoxides that are incompatible with many base-sensitive functional groups.^2^ Moreover, the deprotonation approach is inherently synthetically inefficient, since it requires prior assembly of the entire carbon skeleton of the carbonyl precursor. Given the central role of enolates in organic synthesis, reactions that allow for modular generation of enolates, *e.g.*, by carbon–carbon bond-forming reactions under neutral conditions and in the absence of strong bases are highly sought after but have remained elusive.

**Scheme 1:**
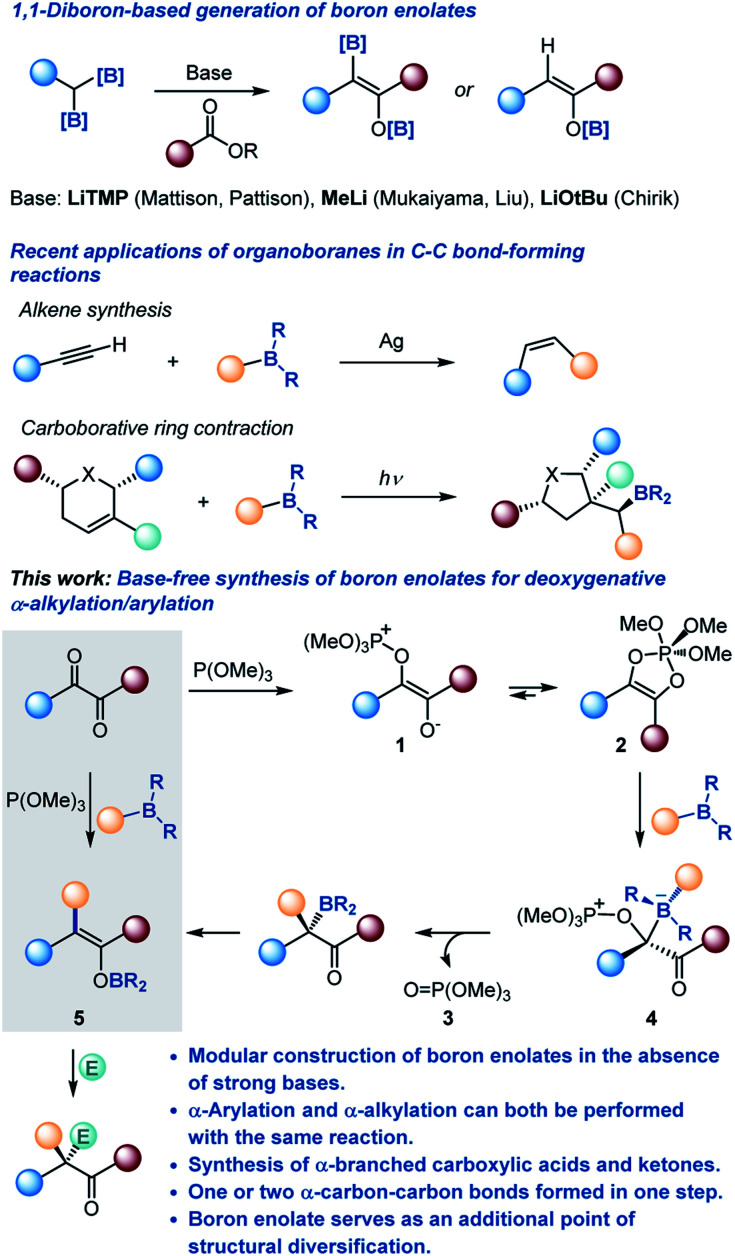
Boron enolates and the deoxygenative α-alkylation/arylation of 1,2-dicarbonyls.

Since their introduction by Mukaiyama in 1973,^3^ boron enolates have become the preferred class of carbon-centered nucleophiles for many chemo- and stereoselective carbon–carbon bond-forming reactions, due to the presence of the inherently Lewis acidic boryl group that enables more selective reactions with a broader range of electrophiles.^2,4^ Consequently, there has been an upsurge of interest in new approaches to their generation, exemplified by geminal diboronate-based methods recently developed by Pattison, Liu and Chirik rooted in the earlier studies by Mukaiyama and Matteson (Scheme 1).^5^

Organoboranes have long served as broadly useful synthetic linchpins,^6^ and the recent examples of carbon–carbon bond-forming reactions, for instance, Lalic's *Z*-alkene synthesis^7^ and the carboborative ring contraction,^8^ showcase the untapped reactivity of organoboranes. The versatility of organoboranes coupled with the ease of their preparation by well-established routes, *e.g.*, hydroboration, ensures their continued presence at the forefront of organic synthesis.^9^

In a parallel development, there has been an increased realization of the expanding synthetic potential of 1,2-dicarbonyl compounds, stemming from the proximity of the two reactive carbonyl groups and ready synthetic accessibility of structurally diverse 1,2-dicarbonyls.^10^

Merging these two classes of versatile synthetic intermediates, we hypothesized that an addition of an organoborane to 1,2-dicarbonyl-phosphite adducts **1** and **2** (ref. 11) would be followed by a 1,2-metallate shift that is accompanied by the departure of phosphate **3** from the zwitterionic intermediate **4** by analogy with the Matteson reaction that has recently been adopted to a variety of valuable synthetic transformations.^12^ Subsequent 1,3-borotropic shift would result in the formation of boron enolate **5**. Notably, construction of the boron enolate would be achieved in the absence of strong bases or strongly Lewis acidic boron reagents (*e.g.*, boryl triflates and halides) and in a modular manner, *i.e.*, with concomitant formation of a carbon–carbon bond in the α-position. Furthermore, the modularity of the reaction combined with the neutral reaction conditions and the weakly Lewis acidic character of the boryloxy group were expected to enable a streamlined synthesis of highly α-branched carboxylic acids and other carbonyls by engaging a range of electrophiles in a tricomponent cascade process.

α-Branched carbonyl compounds, in particular carboxylates, are among the most synthetically valuable and medicinally important structural motifs, whose applications range from non-steroidal anti-inflammatory drugs (NSAIDs)^13^ to versatile reagents for late stage functionalization enabled by resurgent decarboxylative methodologies.^14^ A number of synthetic approaches to α-branched carbonyls have recently emerged, including transition metal-catalyzed α-arylation of enolates,^15^ carbonylation^16^ and electrophilic α-alkylation.^17^ However, many challenges remain unsolved, including the lack a unified synthetic platform that can enable both α-arylation and α-alkylation under essentially neutral conditions and in the absence of non-chemoselective or expensive reagents and catalysts. Thus, further work is needed to develop such a synthetic platform for production of structurally diverse α-branched carbonyls by a conceptually distinct aryl–C_α_ and alkyl–C_α_ bond forming process that encompasses both α-arylation and α-alkylation.

## Results and discussion

**Scheme 2:**
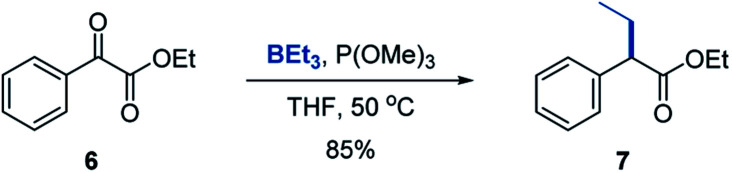
Deoxygenative α-alkylation/arylation of ester **6**. Reaction conditions: ester **6** (0.2 mmol), BEt_3_ (0.3 mmol), P(OMe)_3_ (0.24 mmol), THF (2 mL), 50 °C.

We chose triethylborane as a simple and readily available boron reagent for our initial synthetic scope studies, based on our prior experience^8^ and cognizant that successful development of the reaction with BEt_3_ would enable utilization of other organoboron sources, including the widely used organo-9-borabicyclo[3.3.1]nonanes (BBN). We found that the reaction of ester **6** with triethylborane and trimethyl phosphite proceeded cleanly at 50 °C in THF, affording α-branched carboxylate **7** in good yield (Scheme 2). Importantly, the more readily available and inexpensive P(OMe)_3_ can be used, although P(NMe_2_)_3_ is also a suitable P^III^ reagent.

**Scheme 3:**
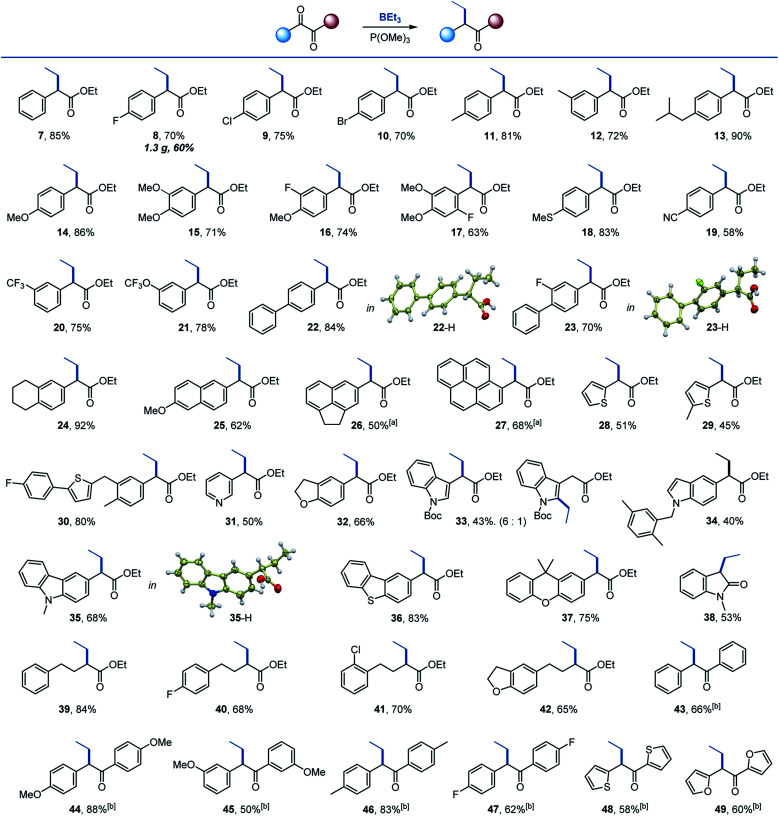
Scope of 1,2-dicarbonyl compounds. Reaction conditions: 1,2-dicarbonyl (0.1–0.2 mmol), BEt_3_ (1.5–2 equiv.), P(OMe)_3_ (1.2–2 equiv.) THF (1–4 mL), 50 °C. ^*a*^80 °C. ^*b*^P(NMe_2_)_3_ was used.

We then proceeded with the evaluation of the scope of the deoxygenative alkylation reaction (Scheme 3). Halo, alkyl, methoxy and thio-substituted aromatic keto esters were suitable substrates (**8–18**). The electron-withdrawing cyano, and the medicinally relevant trifluoromethyl and trifluoromethoxy groups were also tolerated (**19–21**). Polycyclic keto esters were readily converted to the corresponding α-alkyl-branched esters in good yields (**22–27**). Similarly, heterocyclic keto esters successfully participated in the reaction, including substrates containing pyridine, thiophene, dihydrobenzofuran, indole, carbazole, dibenzothiophene and xanthene cores (**28–37**). Interestingly, vinylogous substitution in the aromatic ring was also observed for the indole keto ester (**33**). A reaction with *N*-methylisatin afforded 3-alkylated indolone **38**. Aliphatic keto esters reacted equally well and produced the corresponding 2-alkyl branched esters **39–42** in good yields. 1,2-Diketones were evaluated next with P(NMe_2_)_3_ as the optimal P^III^ reagent, likely due to the increased nucleophilicity imparted by the more basic P^III^ reagent,^18^ and a range of α-branched ketones **43–49** were prepared, further highlighting the scope of the reaction. Esters **22**, **23**, and **35** were converted to corresponding acids, whose structures were confirmed by X-ray crystallographic analysis.

**Scheme 4:**
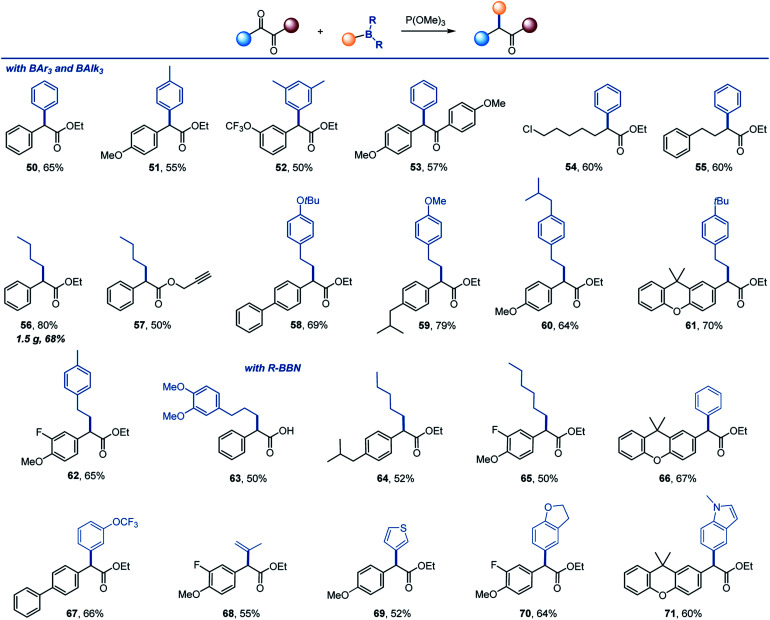
Scope of organoboron reagents. Reaction conditions: see footnote for Scheme 2.

The reaction can be further extended to other organoboranes (Scheme 4). α-Arylation of carboxylic acids and other carbonyl compounds remains a long-standing synthetic challenge.^15^ Significantly, facile arylation can be achieved, providing a metal-free access to α-mono- and diarylated esters and ketones (**50–55**). Other alkylboranes can be used as well (**56–62**), including those derived from alkenes by hydroboration (**58–62**). Importantly, 9-BBN-derived organoboranes that can be easily prepared by hydroboration of alkenes and other methods^19^ proved to be suitable reagents for the transfer of alkyl (**63–65**), aryl (**66**, **67**), vinyl (**68**) and heteroaryl groups (**69–71**).

**Scheme 5:**
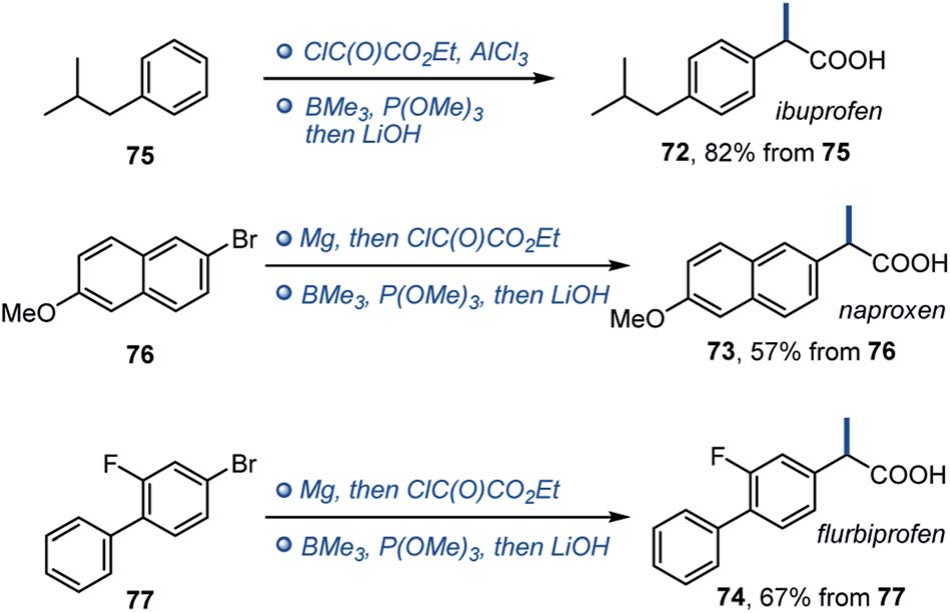
Synthesis of NSAIDs **72–74** with trimethylborane.

Given the importance of α-arylpropionic acids as NSAIDs, we evaluated trimethylborane in the synthesis of ibuprofen, naproxen and flurbiprofen (**72–74**, Scheme 5). In all three cases, NSAIDs **72–74** were readily accessed in two synthetic operations from the corresponding precursors **75–77**, pointing to the potential of the deoxygenative alkylation reaction in drug discovery applications.

**Scheme 6:**
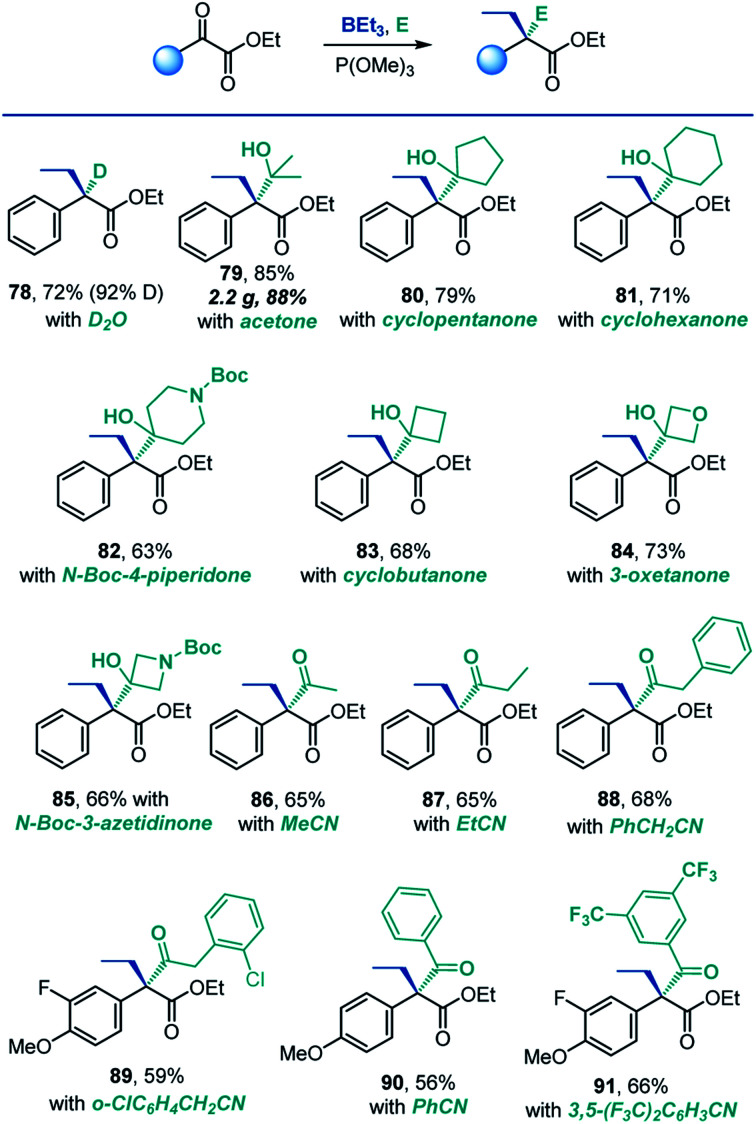
Scope of the tricomponent reaction. Reaction conditions: see footnote for Scheme 2, in the presence of electrophile.

A remarkable feature of the reaction is that it produces reactive boron enolates under neutral conditions in the absence of strong bases that are typically required for generation of enolates. We, therefore, next explored the feasibility of carrying out the reaction directly in the presence of electrophiles. This combination would enable a tricomponent coupling process that, due to the mildness of the reaction conditions and the enhanced reactivity of boron enolates, could engage a range of electrophiles, including both very reactive (*e.g.*, proton sources) and typically unreactive ones (*e.g.*, nitriles and ketones). Indeed, the reaction could be successfully carried out in the presence of deuterium oxide, and the corresponding α-deuterated ester **78** was isolated in 72% yield with 92% deuterium incorporation (Scheme 6). This result suggests that the method may be useful in the context of deuterated drug discovery as a means of improving metabolic stability.^20^

Ketones are typically challenging electrophiles for enolate trapping, because of their facile enolization, lower reactivity and reversibility of the addition reaction.^21^ Interestingly, a variety of ketones were compatible with the deoxygenative α-alkylation reaction, and the corresponding sterically congested addition products **79–85** featuring two adjacent quaternary carbons were readily formed in good yields.

Intermolecular C-acylation of enolates with nitriles is a potentially synthetically attractive approach to β-dicarbonyl compounds. However, examples of such reactions are rare.^22^ Significantly, both aliphatic and aromatic nitriles reacted smoothly in a tricomponent manner under the conditions of the deoxygenative α-alkylation (**86–91**, Scheme 6), presumably due to the Lewis acidic character of the boryl group of the intermediate boron enolate, leading to concomitant construction of two C–C bonds and an all-carbon quaternary α-position in one step.

**Scheme 7:**
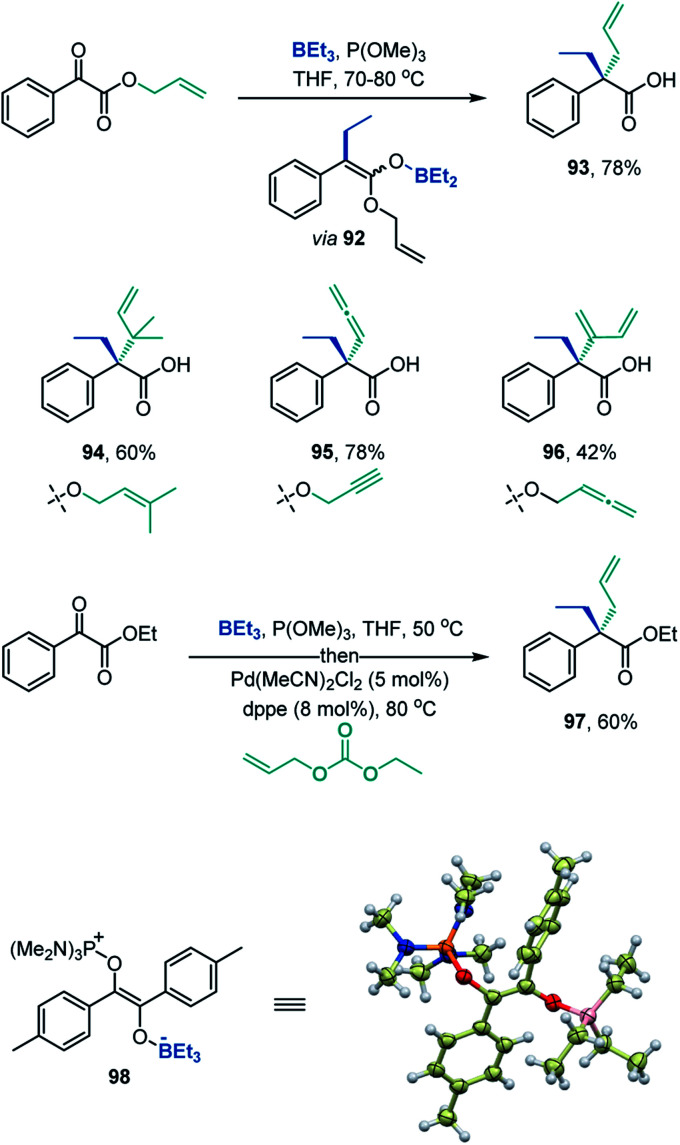
Construction of quaternary all-carbon α-positions in carboxylic acids and the structure of intermediate **98**.

The deoxygenative α-alkylation can be further leveraged for construction of all-carbon quaternary α-positions in carboxylic acids by the Ireland-Claisen rearrangement of the intermediate boron enolates,^23^*e.g.*, **92** (Scheme 7). The Ireland-Claisen-enhanced deoxygenative α-alkylation reaction enables installation of allylic α-side chains (**93**), including the sterically encumbered β,β-dimethylallyl group in the α-branched acid **94**. Expansion of the scope to propargylic and allenylic substrates provides straightforward access to carboxylic acids bearing α-allenyl and α-dienyl groups (**95** and **96**). The deoxygenative α-alkylation can also be combined with the Pd-catalyzed allylation (Scheme 7, **97**), indicating that the intermediate boron enolates can serve as a platform for C–C bond-forming functionalization by means of transition metal catalysis. Furthermore, the deoxygenative coupling reaction can be readily carried out on gram scale (**8**, **56**, **79**).

Kinetic and computational studies were further performed to clarify the reaction mechanism. We found that TEMPO had no detrimental effect on the reaction performance, indicating that the reaction does not proceed by a radical pathway.

Furthermore, organoboranes are known to form adducts with phosphites,^24^ and this process was expected to take place under the conditions of the deoxygenative α-alkylation/α-arylation. Indeed, formation of the 1 : 1 adduct was observed by NMR spectroscopy with *K*_assoc_ = 1.62 × 10^3^ M^−1^ for the association of triethylborane with trimethyl phosphite.

**Fig. 1:**
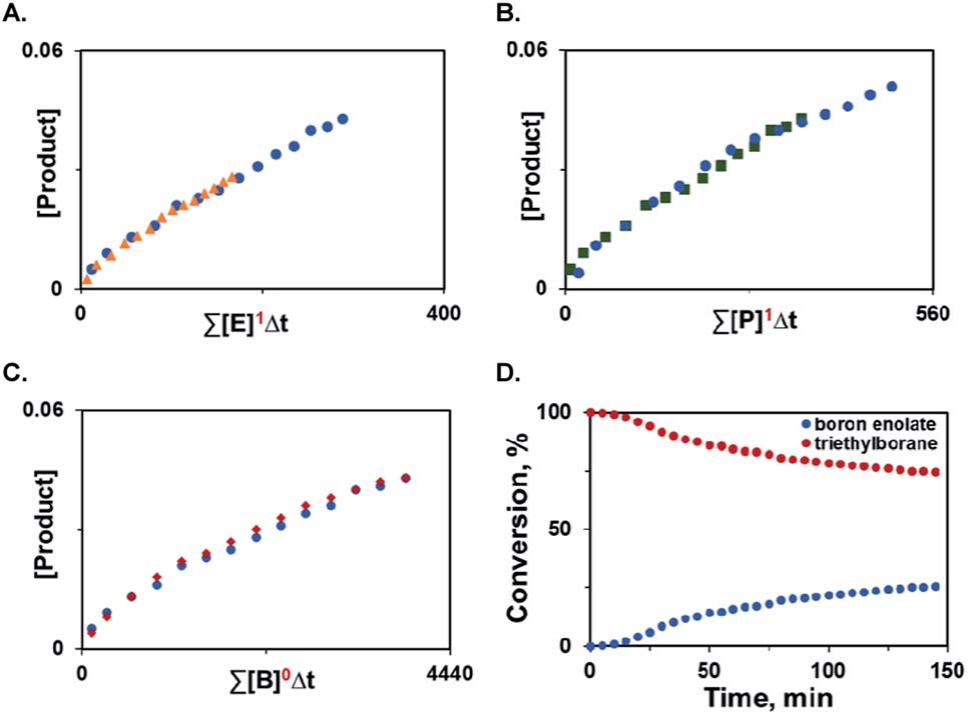
Kinetic studies of the deoxygenative α-alkylation and boron enolate formation. (A) Order in α-keto ester. (B) Order in trimethyl phosphite. (C) Order in triethylborane. (D) Time course of triethylborane consumption and boron enolate formation during the reaction of ester **6** with triethylborane and trimethyl phosphite.

Additionally, variable time normalization analysis^25^ revealed that the deoxygenative α-alkylation was zero order in triethylborane, and first order in α-keto ester and trimethyl phosphite (Fig. 1A–C). The intermediacy of boron enolates was supported by kinetic ^11^B NMR spectroscopy studies that confirmed formation of the dialkylboryloxy group (*δ* = 53.1 ppm)^2^ with concomitant consumption of triethylborane (Fig. 1D). Moreover, zwitterionic adduct **98** crystallized from a P(NMe_2_)_3_-mediated reaction of the corresponding 1,2-diketone with triethylborane and was characterized by X-ray crystallography (Scheme 7), confirming participation of adducts **2** in the process. However, an adduct analogous to **98** was not isolated or observed with ester **6** and triethyl phosphite, indicating that it is less stable for these reactants.

**Fig. 2:**
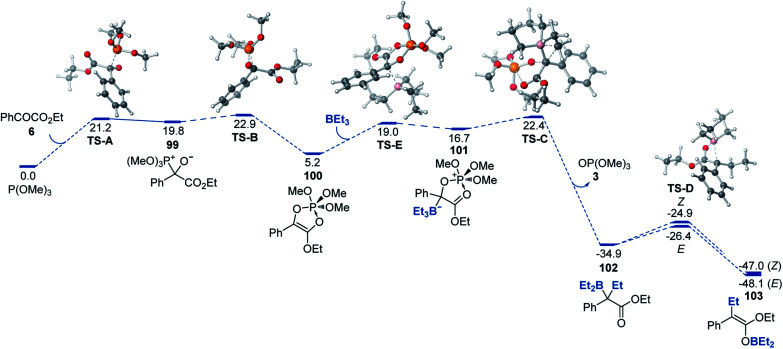
Computed Gibbs free energy profile of the deoxygenative α-alkylation reaction, Δ*G*, kcal mol^−1^.

A DFT computational study provided further insights into the mechanism of the reaction that were consistent with the experimental data (Fig. 2). We found that the addition of phosphite to the carbon of the keto group (**99**) that is followed by a 1,2-phospha-Brook migration of the phosphite group to the oxygen with subsequent ring closure en route to adduct **100** was kinetically accessible (**TS-A** and **TS-B**) with an overall barrier of 22.9 kcal mol^−1^, in excellent agreement with the experimental value (22.7 kcal mol^−1^). Alternative mechanisms of formation of adduct **100** (*e.g.*, a direct addition of phosphite to the O atom of the carbonyl or a [4 + 1] cheletropic cycloaddition of phosphite to ester **6**) were also considered, but were found to proceed over prohibitively high barriers (Δ*G*^≠^ > 40 kcal mol^−1^, see ESI†). The addition of the organoborane to cyclic ester–phosphite adduct **100** further produced zwitterionic intermediate **101** that underwent a Matteson-type displacement *via***TS-C** en route to α-boryl ester **102**. Subsequent low-barrier (**TS-D**) exergonic 1,3-borotropic shift produces *E*- and *Z*-isomers of boron enolate **103**, with the *E*-enolate being the more stable isomer.

Although the computed difference between **TS-B** and the second highest barrier (**TS-C**) is relatively small, we note that formation of adduct **101** as a rate-limiting step (*i.e.*, higher **TS-B**) is consistent with the experimentally observed zero order in organoborane and first order in both ester **6** and phosphite. The alternative pathway that proceeds *via* the acyclic adduct of type **1** was also investigated but was found to proceed over higher barriers and with the Matteson-type displacement as the rate-limiting step (see ESI†), thus being inconsistent with the experimental data. Taken together, our experimental and computational studies support the proposed mechanism of the modular boron enolate synthesis from organoboranes and 1,2-dicarbonyl-phosphite adducts.

## Conclusions

In conclusion, we have developed a modular construction of boron enolates in the absence of strong bases that enables installation of both α-alkyl and α-aryl groups in a reaction of readily accessible 1,2-dicarbonyls with organoboranes (easily derived by hydroboration from alkenes or from other precursors). The reaction produces α-branched carboxylates and ketones that are valuable synthetic intermediates and medicinally important motifs. The intermediacy of boron enolates enables a tricomponent coupling process with a range of electrophiles, including the typically unreactive nitriles and ketones, resulting in the construction of two α-C–C bonds. The utility and scope of the reaction are demonstrated with profen-type NSAIDs and a variety of substituted carbocyclic and heterocyclic carbonyls that can be further diversified by means of one-pot rearrangements and a palladium-catalyzed allylation of the intermediate boron enolates. The mechanism of the reaction was investigated experimentally and computationally, revealing the central role of 1,2-dicarbonyl-phosphite adducts and a 1,2-metallate shift in enabling the deoxygenative C–C bond-forming process.

## Conflicts of interest

There are no conflicts to declare.

## Supplementary Material

SC-011-D0SC03118F-s001

SC-011-D0SC03118F-s002
