# Supplementary material for: Deoxygenative α-alkylation and α-arylation of 1,2-dicarbonyls
Source: Chem Sci. 2020 Jul 1;11(34):9101–8. doi: 10.1039/d0sc03118f (PMC8161533; doi:10.1039/d0sc03118f)
Supplement: SC-011-D0SC03118F-s001 [file SC-011-D0SC03118F-s001.pdf]

Electronic Supplementary Material (ESI) for Chemical Science.
This journal is © The Royal Society of Chemistry 2020

Supporting Information

Deoxygenative α-Alkylation and α-Arylation of 1,2-Dicarbonyls

Shengfei Jin, Hang. T. Dang, Graham C. Haug, Viet D. Nguyen, Hadi D. Arman and Oleg V. Larionov*

Department of Chemistry, The University of Texas at San Antonio, San Antonio, Texas 78249, United States

oleg.larionov@utsa.edu

Contents

Materials and experimental details ………………………………………… S1
General procedures ………………………………………………………… S2
Mechanistic studies ………………………………………………………… S4
Synthesis of starting materials …………………………………………… S13
Deoxygenative α-alkylation/α-arylation products ………………… S18
X-ray crystallographic data ……………………………………………… S81
Computational data ………………………………………………………… S89
NMR Spectroscopic data ………………………………………………… S143
References ………………………………………………………………… S331

Materials and experimental details

Materials: Anhydrous tetrahydrofuran was freshly distilled from sodium and benzophenone under the atmosphere of nitrogen and collected fresh before use. Compounds **S7**[1], **S8**[2], **S9–S13**[1], **S14–S16**[2], **S17**[1], **S18**[2], **S19–S22**[1], **S23**[3], **S24**[1], **S25**[4], **S26–S28**[1], **S29–S32**[5] were prepared according to the previously reported procedures.

BMe_3,[6] trialkylboranes[7], triarylboranes[8], and 9-aryl-9-borabicyclo[3.3.1]nonanes (Ar-BBN)[9] were prepared according to the previously reported procedures and were used without further purification. All other chemicals were used as commercially available.

Experimental equipment: Glovebox work was carried out in a nitrogen-filled LC Technology Solutions LCPW-220 glovebox. For convenience, the reactions were routinely set up in the glovebox. However, the yields were comparable when reactions were set up outside of glovebox: **7**, 85% (84% outside of the glovebox), **13**, 90% (86% outside of the glovebox), **15**, 71% (70% outside of the glovebox), **22**, 84% (80% outside of the glovebox) and **44**, 88% (85% outside of the glovebox).

Purification: Column chromatography was performed using CombiFlash Rf-200 (Teledyne-Isco) automated flash chromatography system, as well as manually. Thin layer chromatography was carried out on silica gel-coated glass plates (Merck Kieselgel 60 F254). Plates were visualized under ultraviolet light (254 nm) and using a potassium permanganate stain.

Characterization: ^{1}H, ^{13}C, ^{11}B, and ^{19}F NMR spectra were recorded at 500 MHz (^{1}H), 125 MHz (^{13}C), 202 MHz (^{31}P), 470.5 MHz (^{19}F), and 160.4 MHz (^{11}B) on Bruker AVANCE III 500 instruments in $CDCl_3$ or other specified deuterated solvents with and without tetramethylsilane (TMS) as an internal standard at 25 °C, unless specified otherwise. Chemical shifts (δ) are reported in parts per million (ppm) from tetramethylsilane (^{1}H and ^{13}C), $BF_3{\cdot}OEt_2$ (^{11}B), and $CFCl_3$ (^{19}F). Coupling constants (*J*) are in Hz. Proton multiplicity is assigned using the following abbreviations: singlet (s), doublet (d), triplet (t), quartet (q), quintet (quint.), septet (sept.), multiplet (m), broad (br).

Infrared measurements were carried out neat on a Bruker Vector 22 FT-IR spectrometer fitted with a Specac diamond attenuated total reflectance (ATR) module.

General Procedures

General procedure for the deoxygenative α-alkylation/α-arylation of 1,2-dicarbonyls (GP1)

An oven-dried 8 mL reaction tube was charged with a magnetic stir bar, a 1,2-dicarbonyl (0.2 mmol), THF (0.6–3.7 mL), $P(OMe)_3$ or $P(NMe_2)_3$ (0.24–0.4 mmol). The reaction mixture was stirred for 1 min, and an organoborane (0.3–0.4 mmol) was added. The tube was sealed with a plastic cap and then stirred at the specified temperature (room temperature, 50 or 80 °C) for 16 h. The reaction was quenched with a 1M aqueous solution of potassium hydroxide (1 mL) at 0 °C, and the reaction mixture was stirred at room temperature for 10 min. The reaction mixture was extracted with ethyl acetate (3 × 10 mL). The combined organic layers were dried over sodium sulfate, filtered, and concentrated. The isolated material was purified by flash column chromatography on silica gel to give the corresponding product.

General procedure for the deoxygenative α-alkylation/α-arylation of 1,2-dicarbonyls (GP2)

Reactions were set up in a nitrogen filled glove box. An oven-dried 8 mL reaction tube was charged with a magnetic stir bar, THF (0.6–3.7 mL), $P(OMe)_3$ or $P(NMe_2)_3$ (0.24–0.4 mmol). An organoborane (0.3–0.4 mmol) was then added. The reaction mixture was stirred for 1 min, and a 1,2-dicarbonyl (0.2 mmol) was added. The tube was sealed with a plastic cap and then stirred at 50 °C for 16 h. The reaction was quenched with a 1M aqueous solution of potassium hydroxide (1 mL) at 0 °C, and the reaction mixture was stirred at room temperature for 10 min. The reaction mixture was extracted with ethyl acetate (3 × 10 mL). The combined organic layers were dried over sodium sulfate, filtered, and concentrated. The isolated material was purified by flash column chromatography on silica gel to give the corresponding product.

Go back to table of contents

General procedure for the tricomponent deoxygenative α-alkylation/α-arylation of 1,2-dicarbonyls (GP3)

An oven-dried 8 mL reaction tube was charged with a magnetic stir bar, a 1,2-dicarbonyl (0.2 mmol), THF (0.6–3.7 mL), $P(OMe)_3$ (0.24–0.4 mmol), and the corresponding electrophile (0.4–2 mmol). The reaction mixture was stirred for 1 min, and an organoborane (0.3–0.4 mmol) was added. The tube was sealed with a plastic cap and then stirred at 50 °C for 16 h. The reaction was quenched with a 1M aqueous HCl solution (1 mL) at 0 °C, and the mixture was stirred at room temperature for 10 min. The reaction mixture was extracted with ethyl acetate (3 × 10 mL). The combined organic layers were dried over sodium sulfate, filtered, and concentrated. The resulting crude mixture can be purified by flash column chromatography on silica gel to give the corresponding product.

Mechanistic studies

The association constant for trimethyl phosphite and triethylborane

The association constant for trimethyl phosphite and triethylborane was determined by ^{11}P NMR spectroscopy. The concentration of trimethyl phosphite in THF was 2 mM, and concentrations of triethylborane were 0 mM, 0.4 mM, 0.8 mM, 1.2 mM, 1.6 mM, 2.0 mM, 4.0 mM, 8.0 mM, 12 mM and 16 mM. Following a literature protocol,[10] the association constant was calculated at six different concentrations of triethylborane using the equation shown below, the obtained values (Table 1) were averaged to arrive at the average association constant of 1.62 x 10^3 M^{-1}. The Gibbs free energy was then calculated to give ΔG_{assoc} = −4.38 kcal/mol, in good agreement (< 1kcal/mol) with the DFT computed value.

$$\delta = \frac{[P,B] \times \delta_{final} + ([P]_0 - [P,B]) \times \delta_0}{[P]_0}$$

$$[P,B] = \frac{[P]_0 \times (\delta - \delta_0)}{(\delta_{final} - \delta_0)}$$

$$K_{assoc} = \frac{[P,B]}{([P]_0 - [P,B]) \times ([B]_0 - [P,B])}$$

$$\Delta G = -RT\ lnK_{assoc}$$

Table 1. Association constant values for trimethyl phosphite and triethylborane

[B] (mM)	δ, ppm	[P,B]	K_{assoc}
0	139.92	0.00	
0.4	134.38	0.30	1.68E+03
0.8	129.46	0.56	1.62E+03
1.2	125.31	0.78	1.54E+03
1.6	121.18	1.00	1.68E+03
2.0	18.60	1.14	1.55E+03
4.0	110.06	1.60	1.66E+03
8.0	104.61	1.89	
12	103.09	1.97	
16	102.55	2.00	

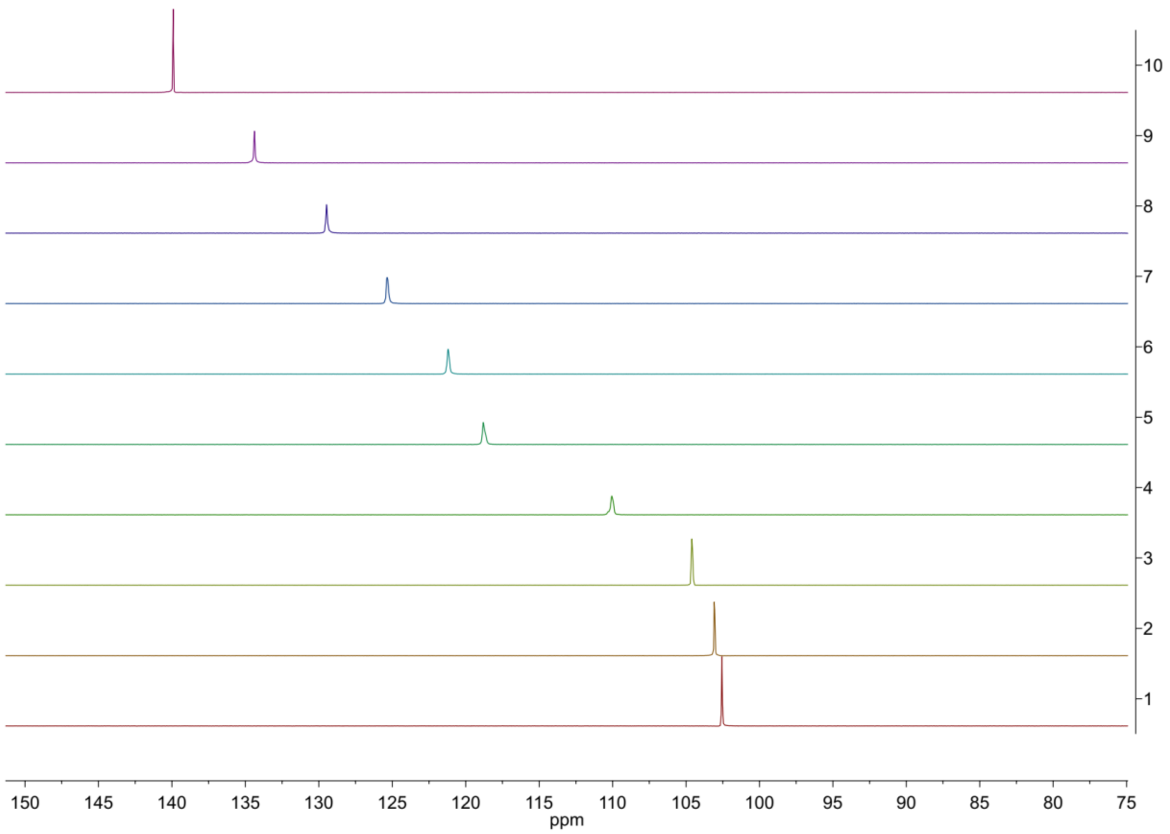

Figure S1. The ^{31}P NMR spectra for the titration of a solution of trimethyl phosphite with increasing amounts of triethylborane (top to bottom). The concentration of triethylborane varies from 0 mM (top spectrum) to 16 mM (bottom spectrum).

Job plot determination of the binding stoichiometry of triethylborane and trimethyl phosphite

The binding stoichiometry of triethylborane and trimethyl phosphite was determined by ^{31}P NMR spectroscopy by generating a Job plot using the chemical shift of trimethyl phosphite. For the plot, the total concentration of triethylborane and trimethyl phosphite in a THF solution was held constant at 2 mM. The concentration of the [P,B] adduct (Et_3B–$P(OMe)_3$) was calculated in the same manner as in the association constant measurements in the preceding section. The maximum of the plot occurring at a trimethyl phosphite

mole fraction of 0.5 confirms the 1 : 1 binding ratio of triethylborane and trimethyl phosphite.

Table 2. Job plot data for the trimethyl phosphite–triethylborane adduct formation.

[P]	δ, ppm	[P,B]
0	–	0
0.1	109.09	0.178105
0.2	110.78	0.336684
0.3	113.37	0.460139
0.4	115.96	0.553668
0.5	119.44	0.591566
0.6	123.5	0.569151
0.7	128.6	0.45777
0.8	132.66	0.335529
0.9	136.75	0.164818
1	139.92	0

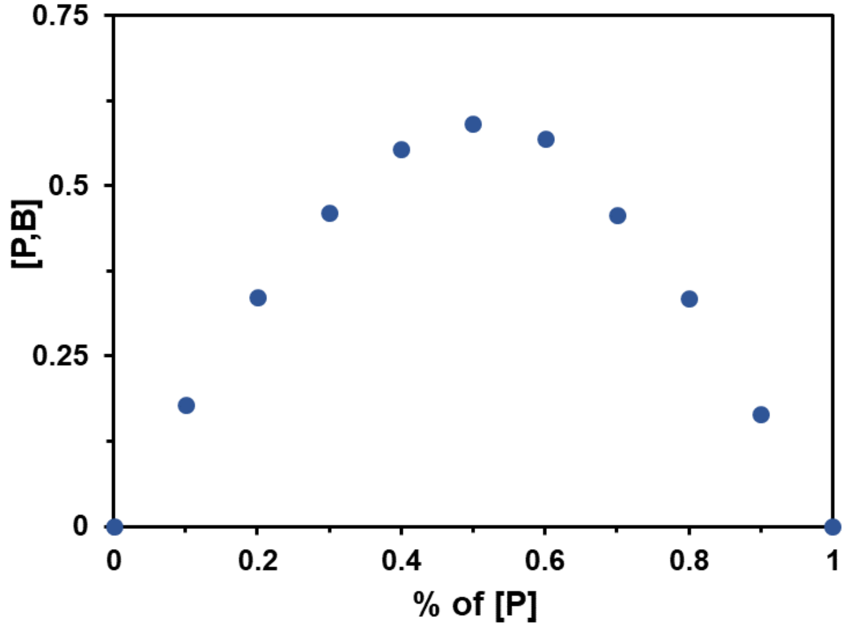

Figure S2. Job plot for the trimethyl phosphite–triethylborane adduct formation.

Variable time normalization analysis of the deoxygenative α-alkylation/ α-arylation of 1,2-dicarbonyls

General procedure for the variable time normalization analysis (VTNA) studies.

Ethyl 2-(4-fluorophenyl)-2-oxoacetate **6** (0.12–0.2 mmol) and fluorobenzene (internal standard) (0.05 mmol) were added to a two-dram screw cap vial and dissolved in freshly distilled THF (1.7 mL) in the glove box. Then $P(OMe)_3$ (0.24–0.32 mmol) was added, and the reaction was vigorously stirred at room temperature for 1 min. A dry NMR tube was then filled with 0.43 mL of the reaction mixture, capped with a rubber septum and sealed with parafilm. The NMR instrument was preheated to the desired temperature. Triethyl borane (0.075–0.1 mmol) was injected to the NMR tube via a microsyringe. ^{19}F and ^{31}P NMR spectra were recorded every 5 minutes. The variable time normalization analysis was conducted according to the literature procedure.[11]

The consumption of the α-ketoester and formation of the product were monitored by ^{19}F NMR spectroscopy with the peak of ethyl 2-(4-fluorophenyl)-2-oxoacetate at −104.2 (td, *J*

= 8.5, 4.3 Hz) ppm, product at −117.5 (tt, J = 8.9, 5.2 Hz) ppm, and fluorobenzene as an internal standard at −114.5 (tt, J = 9.4, 5.7 Hz) ppm.

The amount of trimethyl phosphite and triethylborane consumed were calculated using ^{31}P NMR based on the peaks of trimethyl phosphite at 138.0 (dt, J = 19.8, 9.7 Hz) ppm, and trimethyl phosphate at 2.40 (dhept, J = 21.8, 10.9 Hz) ppm.

Graphs for the determination of the reaction order in α-ketoester [E], trimethyl phosphite [P] and triethylborane [B]

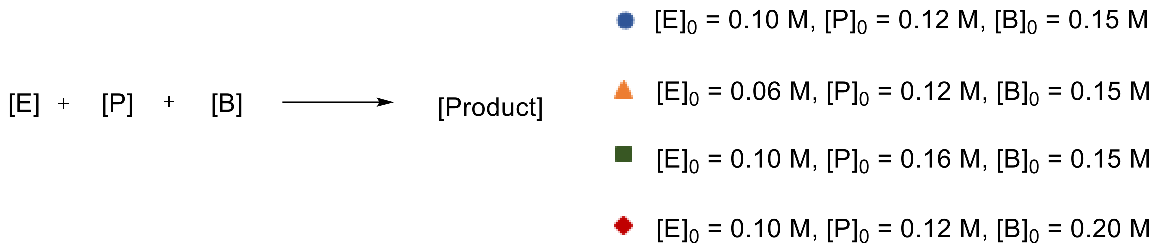

a. Order in α-ketoester [E]

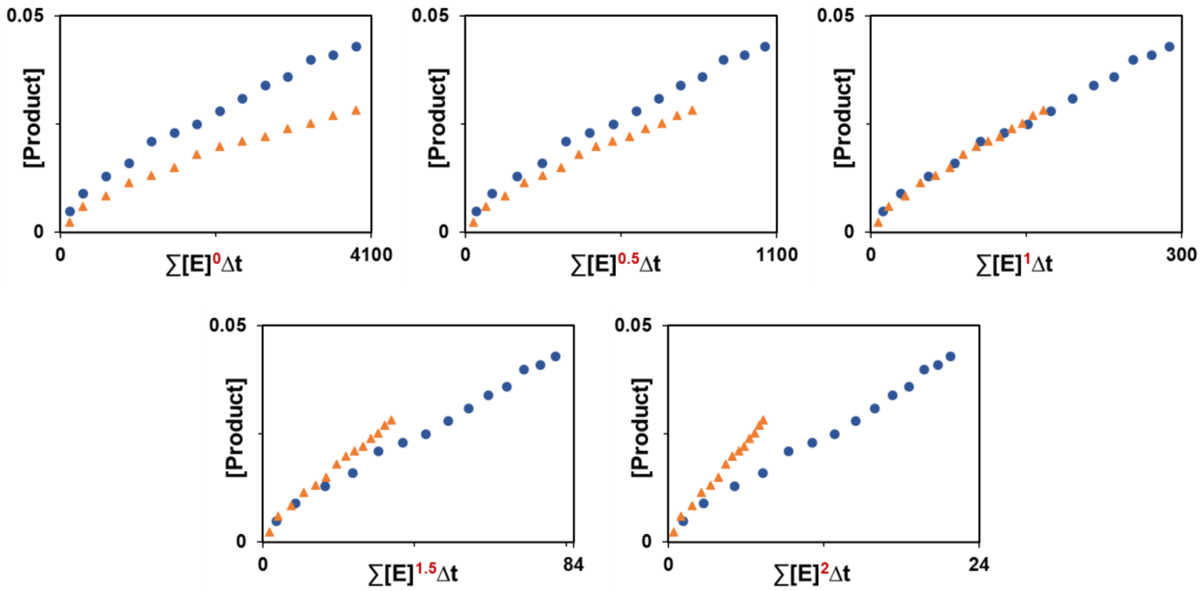

Figure S3. Rate order determination for the α-ketoester. The best fit was observed for the first order.

b. Order in trimethyl phosphite [P]

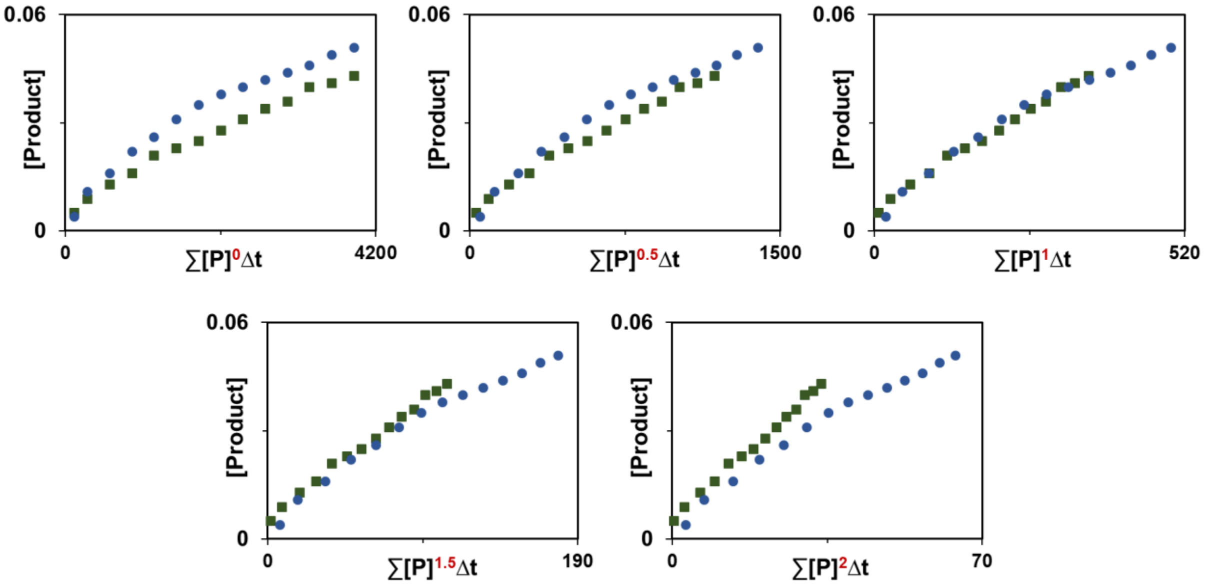

Figure S4. Rate order determination for trimethyl phosphite. The best fit was observed for the first order.

c. Order in triethylborane [B]

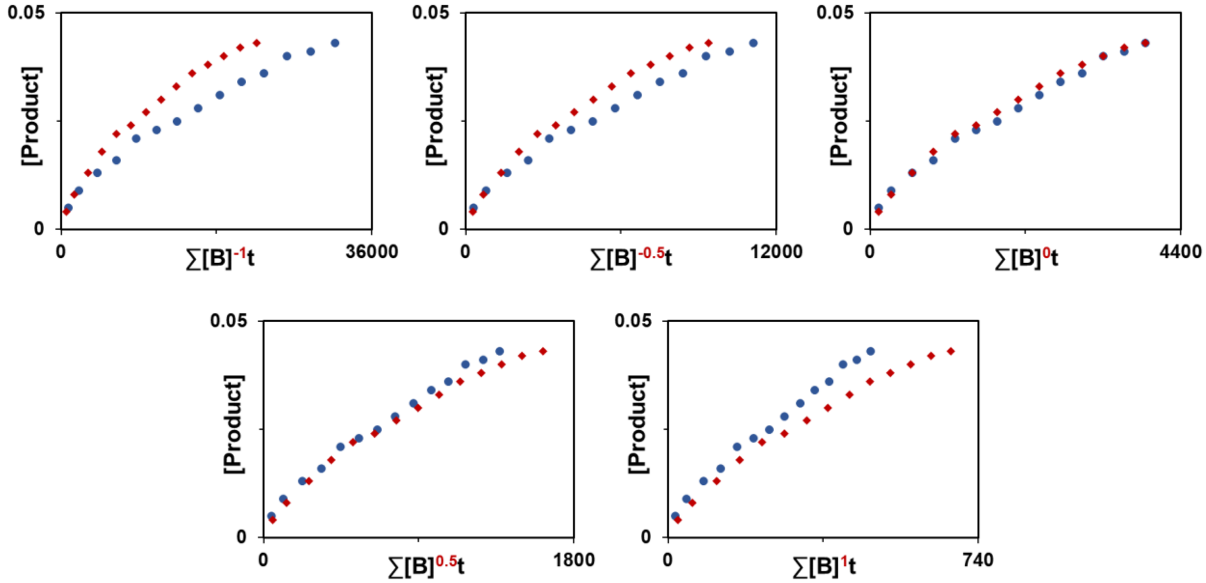

Figure S5. Rate order determination for triethylborane. The best fit was observed for the zero order.

Rate constant determination

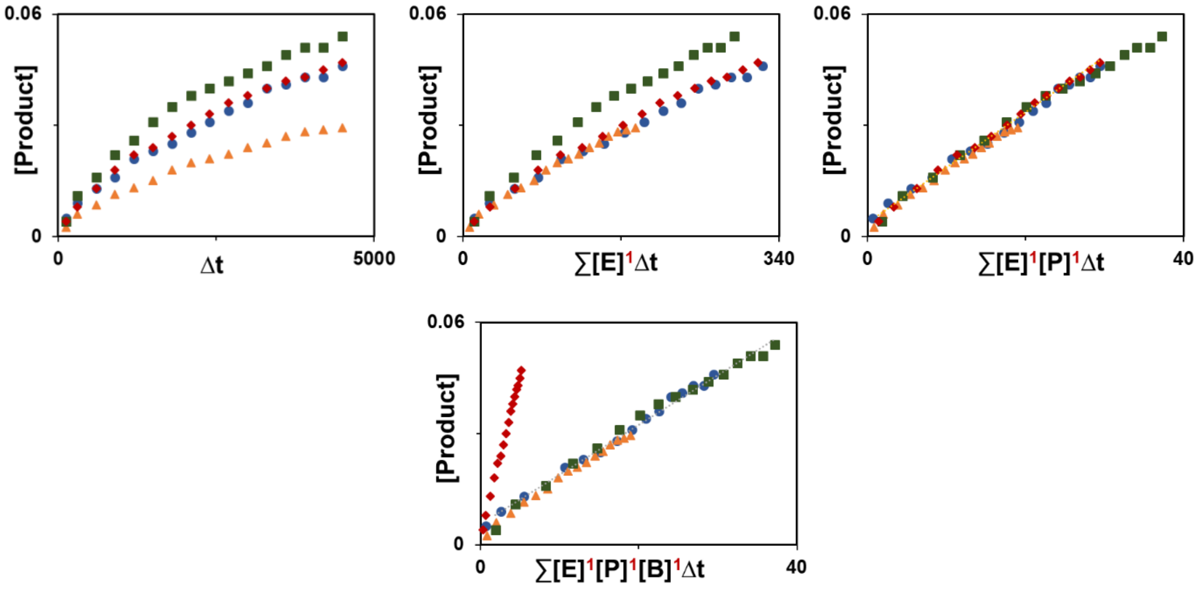

Figure S6. Rate constant determination.

Determination of Gibbs free energy, enthalpy and entropy of activation by the Eyring–Polanyi equation

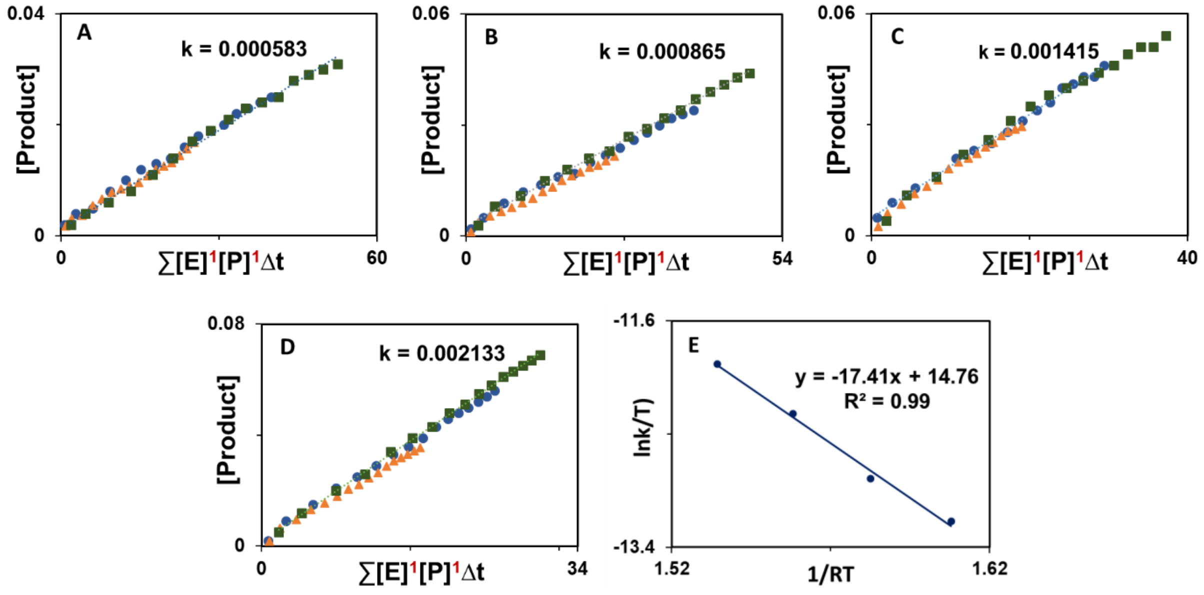

Figure S7. Determination of rate constants at different temperatures. **A**. 40 °C. **B**. 45 °C. **C**. 50 °C. **D**. 55 °C. **E**. The Eyring–Polanyi plot.

The general formula for the Eyring–Polanyi equation:

$$ln\frac{k}{T} = -\Delta H^{\neq}\frac{1}{RT} + ln\frac{\kappa k_B}{h} + \frac{\Delta S^{\neq}}{R}$$

From the Eyring–Polanyi plot: Δ*H*≠ = 17.4 kcal/mol, Δ*S*≠ = –17.9 e.u.

Gibbs free energy of activation at room temperature: Δ*G*≠ = 22.7 kcal/mol.

Eyring plot error was determined according to the previously described protocol.[12]

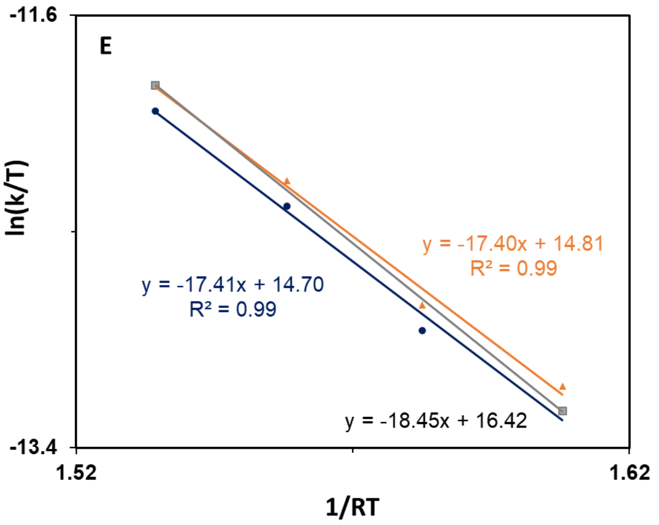

Figure S8. Eyring plot error analysis graph.

Error in T = ± 1 K ≈ 0.31%, Error in *k* ≈ 5% (combination of NMR integration and concentration)

Error (ln(*k*/T)) < ± 0.4%, Plot of upper to lower extreme gives y = –18.45x + 16.42

Error: Δ*S*‡ = ± 18%, Δ*H*‡ = ± 6%, Δ*G*‡ = ± 0.3%

ΔS‡ = −17.9 (3.2) eu, Δ*H*‡ = 17.4 (1) kcal/mol, Δ*G*‡ = 22.7 (0.1) kcal/mol.

Monitoring of boron enolate formation by ^{11}B NMR spectroscopy

The consumption of triethylborane and formation of the boron enolate were determined by integration of the signals of triethylborane at 61.1 ppm and of the corresponding boron enolate at 53.1 ppm in ^{11}B NMR spectra of the reaction mixture

of ester **6** with triethylborane and trimethyl phosphite. The boron enolate signal appeared in the range of the dialkylboryloxy (R_2B–O) group.[13]

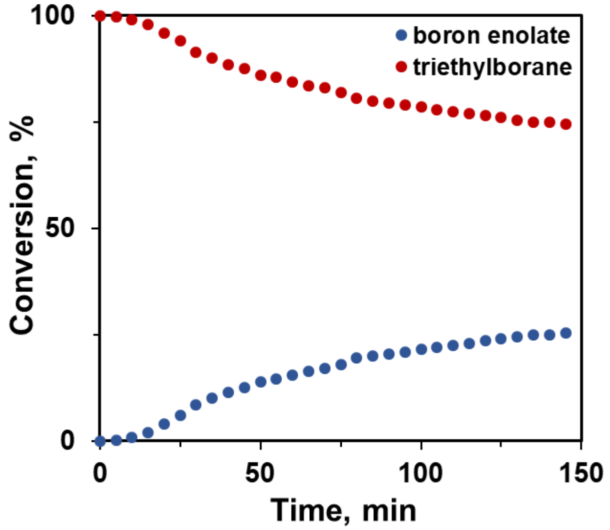

Figure S9. Time course of triethylborane consumption and boron enolate formation during the reaction of ester **6** with triethylborane and trimethyl phosphite at 50 °C.

Synthesis of starting materials

Ethyl 2-(2-fluoro-4,5-dimethoxyphenyl)-2-oxoacetate (S1)

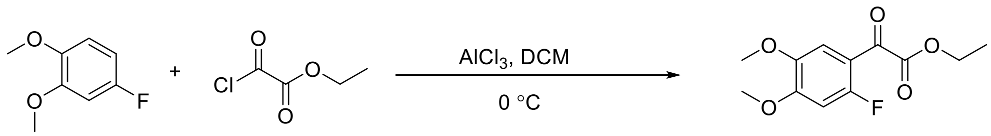

In a three-necked round-bottomed flask $AlCl_3$ (1.3 g, 10 mmol, 2.0 equiv.) was suspended in CH_2Cl_2 (25 mL) at 0 °C. To this mixture ethyl oxalate chloride (1.1 g, 8 mmol, 1.6 equiv.) was added dropwise. 4-Fluoro-1,2-dimethoxybenzene (780 mg, 5.0 mmol) was added dropwise in about 10 min. Then the solution was stirred at room temperature for 12 h. To this reaction mixture the threefold volume of H_2O with ice was carefully added. Extraction was performed with DCM (5 × 30 mL). The organic layers were collected and washed with saturated NaCl solution (2 × 100 mL), dried over Na_2SO_4 and concentrated.

Go back to table of contents

Purification by flash chromatography on silica gel (EtOAc/hexane, 20 : 80 v/v) afford product **S1** (1.25 g, 98%) as a white solid.

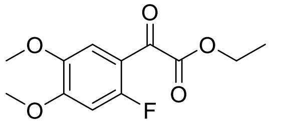

m.p.: 55–56 °C. – ^{1}H NMR (500 MHz, $CDCl_3$): 7.36 (1 H, d, *J* = 6.4 Hz), 6.63 (1 H, d, *J* = 11.9 Hz), 4.42 (2 H, q, *J* = 7.1 Hz), 3.95 (3 H, s), 3.91 (3 H, s), 1.39 (3 H, t, *J* = 7.1 Hz) ppm. – ^{13}C NMR (125 MHz, $CDCl_3$): 183.1, 165.2, 159.5 (d, *J* = 253.7 Hz), 156.5 (d, *J* = 10.7 Hz), 146.4, 112.9 (d, *J* = 11.3 Hz), 109.9 (d, *J* = 3.5 Hz), 99.8 (d, *J* = 28.1 Hz), 62.5, 56.8, 56.6, 14.1 ppm. – ^{19}F NMR (470.5 Hz, $CDCl_3$): −115.8 (dd, *J* = 11.9, 6.5 Hz) ppm. – IR: 2982, 2942, 1739, 1668, 1611, 1514, 1451, 1420, 1377, 1306, 1267, 1226, 1196, 1135, 1022 cm^{-1}. – HRMS calcd for $C_{12}H_{14}FO_5$: 257.0820, found 257.0822 [M+H$^+$].

Ethyl 2-(2-fluoro-[1,1'-biphenyl]-4-yl)-2-oxoacetate (S2)

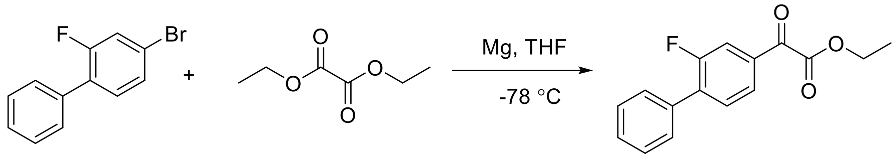

A solution of (2-fluoro-[1,1'-biphenyl]-4-yl)magnesium bromide was prepared by adding dropwise a solution of 4-bromo-2-fluoro-1,1'-biphenyl (1.25 g, 5 mmol) in tetrahydrofuran (1 mL) to magnesium (0.6 g, 25 mmol, 5 equiv.) and I_2 (5 mg) in tetrahydrofuran (6 mL) with magnetic stirring and heating to reflux for 2 h. The diethyl oxalate (730 mg, 5 mmol, 1 equiv.) was dissolved in THF (10 mL) under nitrogen and cooled to –78 °C in a dry ice/acetone bath. The Grignard solution prepared above was added dropwise over 10 min with stirring, and then the reaction mixture was stirred for about 1 hour. The reaction mixture was quenched with saturated NH_4Cl solution and extracted with diethyl ether. The combined organic layer was dried over $MgSO_4$, filtered and concentrated in vacuo. Purification by flash chromatography on silica gel (EtOAc/hexane, 20 : 80 v/v) afford product **S2** (1.2 g, 88%) as a yellow oil.

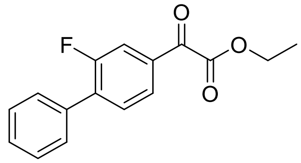

^{1}H NMR (500 MHz, $CDCl_3$): 7.90 (1 H, dd, *J* = 7.9, 1.7 Hz), 7.85 (1 H, dd, *J* = 10.8, 1.7 Hz), 7.65–7.56 (3 H, m), 7.51–7.38 (3 H, m), 4.48 (2 H, q, *J* = 7.1 Hz), 1.45 (3 H, t, *J* = 7.2 Hz) ppm. – ^{13}C NMR (125 MHz, $CDCl_3$): 184.7, 163.3, 159.8 (d, *J* = 250.6 Hz), 135.9 (d, *J* = 13.5 Hz), 134.4, 133.2 (d, *J* = 6.6 Hz), 131.4, 129.2, 129.0, 128.8, 126.4, 117.7 (d, *J* = 24.6 Hz), 62.8, 14.3 ppm. – ^{19}F NMR (470.5 Hz, $CDCl_3$): −115.7 ppm. – IR: 2998, 2943, 2292, 2252, 1738, 1692. 1443, 1374, 1217, 1157, 1037, 918 cm^{-1}. – HRMS calcd for $C_{16}H_{14}FO_3$: 273.0921, found 273.0930 [M+H$^+$].

Ethyl 4-(2,3-dihydrobenzofuran-5-yl)-2-oxobutanoate (S3)

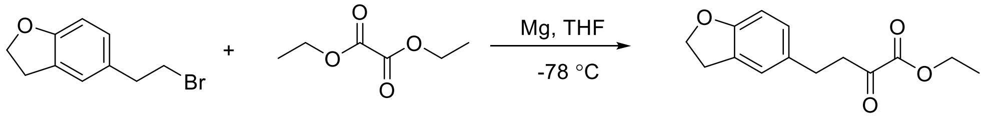

A solution of (2-(2,3-dihydrobenzofuran-5-yl)ethyl)magnesium bromide was prepared by adding dropwise a solution of 5-(2-bromoethyl)-2,3-dihydrobenzofuran (1.13 g, 5 mmol) in tetrahydrofuran (1 mL) to magnesium (0.6 g, 25 mmol, 5 equiv.) and I_2 (5 mg) in tetrahydrofuran (6 mL) with magnetic stirring and heating to reflux for 2 h. The diethyl oxalate (730 mg, 5 mmol, 1 equiv.) was dissolved in THF (10 mL) under nitrogen and cooled to –78 °C. The Grignard solution prepared above was added dropwise over 10 min with stirring, and then the reaction mixture was stirred for about 1 hour. The reaction mixture was quenched with saturated NH_4Cl solution and extracted with diethyl ether. The combined organic layer was dried over $MgSO_4$, filtered and concentrated in vacuo. Purification by flash chromatography on silica gel (EtOAc/hexane, 20 : 80 v/v) afford product **S3** (1.05 g, 85%) as a yellow oil.

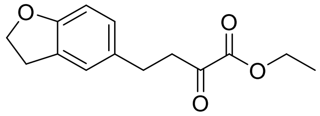

^{1}H NMR (500 MHz, $CDCl_3$): 7.04 (1 H, s), 6.92 (1 H, d, *J* = 8.1 Hz), 6.69 (1 H, d, *J* = 8.1 Hz), 4.54 (2 H, t, *J* = 8.6 Hz), 4.30 (2 H, q, *J* = 7.1 Hz), 3.17 (2 H, t, *J* = 8.7 Hz), 3.13 (2 H, t, *J* = 7.5 Hz), 2.88 (2 H, t, *J* = 7.5 Hz), 1.35 (3 H, t, *J* = 7.1 Hz) ppm. – ^{13}C NMR (125 MHz, $CDCl_3$): 193.9, 161.1, 158.8, 132.2, 127.9, 127.4,

125.1, 109.3, 71.3, 62.6, 41.6, 29.9, 28.6, 14.1 ppm. – IR: 3006, 2944, 2293, 2252, 1731, 1492, 1444, 1374, 1216, 1035, 889 cm^{-1}. – HRMS calcd for $C_{14}H_{17}O_4Na$: 271.0941, found 271.0941 [$M+Na^+$].

Ethyl 2-(1-(2,5-dimethylbenzyl)-1*H*-indol-5-yl)-2-oxoacetate (S4)

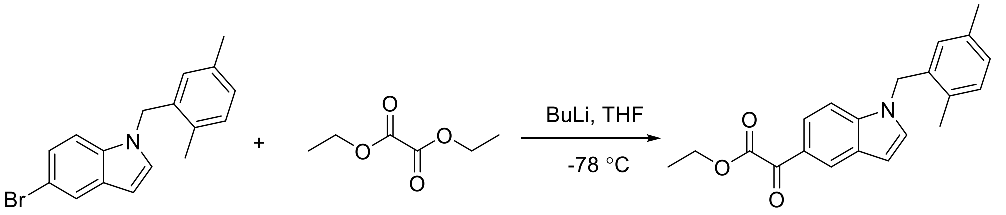

To a stirred suspension of 5-bromo-1-(2,5-dimethylbenzyl)-1*H*-indole (626 mg, 2 mmol) in anhydrous THF (10 mL) under argon, a solution of butyllithium (0.84 mL, 2.5M in hexane, 1.05 equiv.) was added dropwise at –78 °C, and the mixture was stirred for 1 h. Diethyl oxalate (876 mg, 6 mmol, 3 equiv.) was quickly added at –78 °C. The reaction mixture was stirred for 1 hour at –78 °C. The reaction mixture was quenched with saturated NH_4Cl solution and extracted with diethyl ether. The combined organic layer was dried over $MgSO_4$, filtered and concentrated in vacuo. Purification by flash chromatography on silica gel (EtOAc/hexane, 20 : 80 v/v) afford product **S4** (469 mg, 70%) as a white solid.

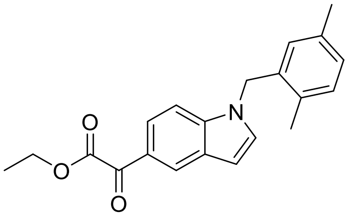

m.p.: 88–89 °C. – ^{1}H NMR (500 MHz, $CDCl_3$): 8.35 (1 H, s), 7.88 (1 H, d, *J* = 8.7 Hz), 7.37 (1 H, d, *J* = 8.7 Hz), 7.11 (1 H, d, *J* = 7.7 Hz), 7.08 (1 H, d, *J* = 3.2 Hz), 7.04 (1 H, d, *J* = 7.7 Hz), 6.68 (1 H, d, *J* = 3.2 Hz), 6.61 (1 H, s), 5.27 (2 H, s), 4.48 (2 H, q, *J* = 7.2 Hz), 2.22 (3 H, s), 2.22 (3 H, s), 1.44 (3 H, t , *J* = 7.2 Hz) ppm. – ^{13}C NMR (125 MHz, $CDCl_3$): 186.9, 165.1, 140.0, 136.3, 134.0, 132.9, 130.8, 130.2, 129.0, 128.4, 126.2, 124.8, 123.2, 110.2, 104.3, 62.2, 48.6, 21.2, 18.8, 14.3 ppm. – IR: 2981, 2915, 2867, 2254, 1731, 1667, 1602, 1453,

1348, 1218, 1130, 1018, 908 cm^{-1}. – HRMS calcd for $C_{21}H_{22}NO_3$: 336.1594, found 336.1603 [M+H$^+$].

Ethyl 2-(3-((5-(4-fluorophenyl)thiophen-2-yl)methyl)-4-methylphenyl)-2-oxoacetate (S5)

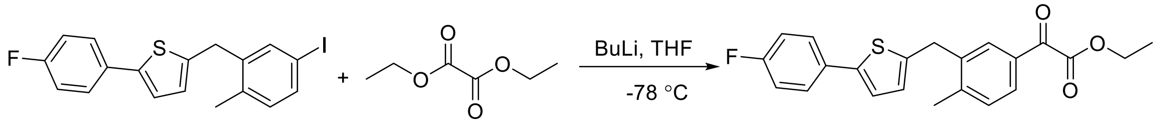

To a stirred suspension of 2-(4-fluorophenyl)-5-(5-iodo-2-methylbenzyl)thiophene (816 mg, 2 mmol) in anhydrous THF (10 mL) under argon, a solution of butyllithium (0.84 mL, 2.5M in hexane, 1.05 equiv.) was added dropwise at –78 °C, and the mixture was stirred for 1 h. Diethyl oxalate (876 mg, 6 mmol, 3 equiv.) was quickly added at –78 °C. The reaction mixture was stirred for 1 hour at –78 °C. The reaction mixture was quenched with saturated NH_4Cl solution and extracted with diethyl ether. The combined organic layer was dried over $MgSO_4$, filtered and concentrated in vacuo. Purification by flash chromatography on silica gel (EtOAc/hexane, 20 : 80 v/v) afford product **S5** (535 mg, 70%) as a yellow oil.

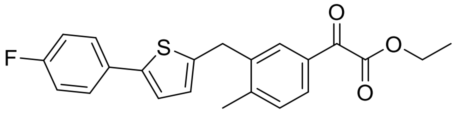

^{1}H NMR (500 MHz, $CDCl_3$): 7.88 (1 H, s), 7.83 (1 H, d, *J* = 7.9 Hz), 7.52–7.42 (2 H, m), 7.32 (1 H, d, *J* = 8.0 Hz), 7.07–6.96 (3 H, m), 6.67 (1 H, d, *J* = 3.5 Hz), 4.42 (2 H, q, *J* = 7.2 Hz), 4.19 (2 H, s), 2.41 (3 H, s), 1.39 (3 H, t, *J* = 7.2 Hz) ppm. – ^{13}C NMR (125 MHz, $CDCl_3$): 186.2, 164.1, 162.3 (d, *J* = 247.0 Hz), 145.0, 142.1 (d, *J* = 11.4 Hz), 139.3, 131.1 (d, *J* = 26.6 Hz), 130.9, 130.8 (d, *J* = 3.0 Hz), 129.1, 127.3 (d, *J* = 8.1 Hz), 126.5, 122.9, 115.9 (d, *J* = 21.8 Hz), 62.4, 34.2, 20.2, 14.3 ppm. – ^{19}F NMR (470.5 Hz, $CDCl_3$): −114.9 ppm. – IR: 2982, 2937, 2908, 1732, 1682, 1604, 1509, 1468, 1315, 1227, 1188, 1160, 1027, 835 cm^{-1}. – HRMS calcd for $C_{22}H_{20}FO_3S$: 383.1112, found 383.1113 [M+H$^+$].

Buta-2,3-dien-1-yl 2-oxo-2-phenylacetate (S6)

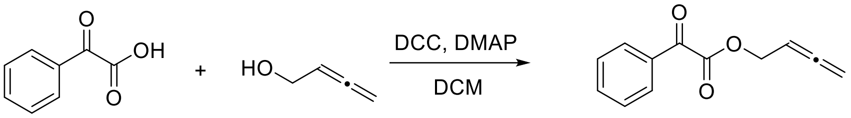

A 50 mL flask was charged with a solution of the 2-oxo-2-phenylacetic acid (750 mg, 5 mmol) in CH_2Cl_2 (10 mL). DMAP (61 mg, 0.5 mmol, 0.1 equiv.) and the allylic alcohol (1.17 g, 20 mmol) were added in to the solution. The reaction mixture was then cooled to 0 °C and stirred for 15 min before adding a solution of DCC (1.6 g, 7.5 mmol, 1.5 equiv.) in CH_2Cl_2 dropwise. After 6 h, the reaction mixture was filtered, diluted with 20 mL of saturated aqueous $NaHCO_3$ and extracted with Et2O (3x30 mL). The combined organic layer was dried over $MgSO_4$, filtered and concentrated in vacuo. Purification by flash chromatography on silica gel (EtOAc/hexane, 5 : 95 v/v) afford product **S6** (808 mg, 80%) as a yellow oil.

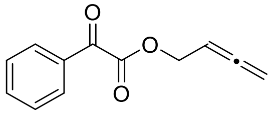

^{1}H NMR (500 MHz, $CDCl_3$): 8.02 (2 H, dd, *J* = 8.2, 1.5 Hz), 7.66 (1 H, t, *J* = 7.4 Hz), 7.52 (2 H, t, *J* = 7.8 Hz), 5.42 (1 H, p, *J* = 6.8 Hz), 4.92 (2 H, dt, *J* = 6.3, 2.3 Hz), 4.88 (2 H, dt, *J* = 7.1, 2.3 Hz) ppm. – ^{13}C NMR (125 MHz, $CDCl_3$): 210.5, 186.1, 163.6, 135.1, 132.6, 130.2, 129.0, 85.6, 77.3, 64.1 ppm. – IR: 3066, 2964, 2111, 1955, 1733, 1666. 1450, 1193, 1173, 964 cm^{-1}. – HRMS calcd for $C_{12}H_{10}O_3Na$: 225.0522, found 225.0518 [M+Na$^+$].

Deoxygenative α-alkylation/α-arylation products

Ethyl 2-phenylbutanoate (7)

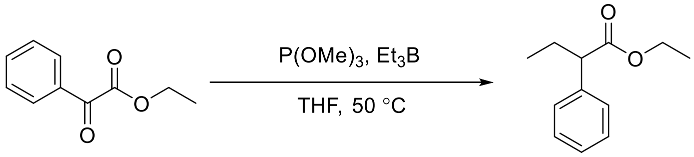

The general procedure GP1 was followed with ethyl 2-oxo-2-phenylacetate (**6**) (36 mg, 0.2 mmol), $P(OMe)_3$ (30 mg, 0.24 mmol, 1.2 equiv.), Et_3B (0.3 mL, 1M, 1.5 equiv.) and THF (1.7 mL). The mixture was stirred at 50 °C for 16 h. Purification by flash chromatography on silica gel (EtOAc/hexane, 3 : 97 v/v) afford product **7** (33 mg, 85%, or 33 mg, 84% outside glovebox) as a colorless oil.

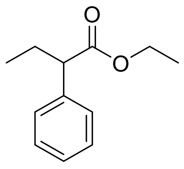

^{1}H NMR (500 MHz, $CDCl_3$): 7.33–7.18 (5 H, m), 4.23–3.93 (2 H, m), 3.44 (1 H, t, *J* = 7.7 Hz), 2.15–2.02 (1 H, m), 1.89–1.67 (1 H, m), 1.21 (3 H, t, *J* = 7.1 Hz), 0.90 (3 H, t, *J* = 7.4 Hz) ppm. – ^{13}C NMR (125 MHz, $CDCl_3$): 174.2, 139.4, 128.7, 128.1, 127.2, 60.8, 53.7, 27.0, 14.3, 12.3 ppm. – IR: 2966, 2934, 1732, 1454, 1369, 1265, 1200, 1172, 1024 cm^{-1}. – HRMS calcd for $C_{12}H_{17}O_2$: 193.1223, found 193.1222 [M+H$^+$].

Ethyl 2-(4-fluorophenyl)butanoate (8)

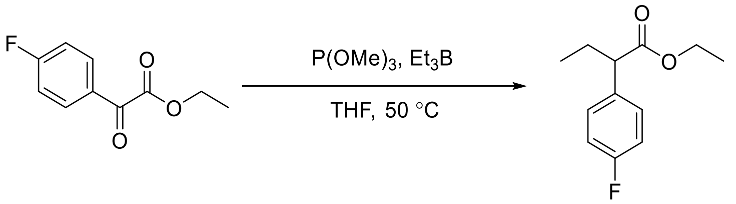

The general procedure GP2 was followed with ethyl 2-(4-fluorophenyl)-2-oxoacetate (39.2 mg, 0.2 mmol), $P(OMe)_3$ (30 mg, 0.24 mmol, 1.2 equiv.), Et_3B (0.3 mL, 1M, 1.5 equiv.) and THF (1.7 mL). The mixture was stirred at 50 °C for 16 h. Purification by flash chromatography on silica gel (EtOAc/hexane, 3 : 97 v/v) afford product **8** (29 mg, 70%) as a colorless oil.

Gram scale synthesis: The general procedure GP2 two identical reactions were run with ethyl 2-(4-fluorophenyl)-2-oxoacetate (980 mg, 5.0 mmol), $P(OMe)_3$ (744 mg, 6.0 mmol, 1.2 equiv.), Et_3B (7.5 mL, 1M, 2.0 equiv.), and THF (40 mL). The mixture was stirred at 50 °C for 16 h. Purification by flash chromatography on silica gel (EtOAc/hexane, 3 : 97 v/v) afford product **8** (1.3 g, 60%) as a colorless oil.

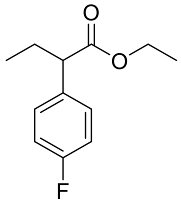

^{1}H NMR (500 MHz, $CDCl_3$): 7.33–7.21 (2 H, m), 7.01–6.96 (2 H, m), 4.23–3.99 (2 H, m), 3.41 (1 H, t, *J* = 7.7 Hz), 2.06 (1 H, dquint., *J* = 14.9, 7.4 Hz), 1.75 (1 H, dquint., *J* = 14.8, 7.4 Hz), 1.20 (3 H, t, *J* = 7.1 Hz), 0.87 (3 H, t, *J* = 7.4 Hz) ppm. – ^{13}C NMR (125 MHz, $CDCl_3$): 174.1, 162.1 (d, *J* = 245.3 Hz), 135.1, 129.6 (d, *J* = 7.8 Hz), 115.5 (d, *J* = 21.3 Hz), 60.8, 52.9, 27.0, 14.3, 12.2 ppm. – ^{19}F NMR (470.5 Hz, $CDCl_3$): −115.9 (tt, *J* = 9.4, 5.2 Hz) ppm. – IR: 2967, 2934, 2878, 1731, 1509, 1223, 1156, 1024, 837 cm^{-1}. – HRMS calcd for $C_{12}H_{16}FO_2$: 211.1129, found 211.1123 [M+H$^+$].

Ethyl 2-(4-chlorophenyl)butanoate (9)

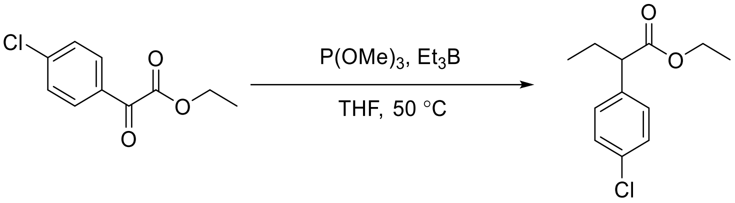

The general procedure GP1 was followed with ethyl 2-(4-chlorophenyl)-2-oxoacetate (42.4 mg, 0.2 mmol), $P(OMe)_3$ (30 mg, 0.24 mmol, 1.2 equiv.), Et_3B (0.3 mL, 1M, 1.5 equiv.) and THF (1.7 mL). The mixture was stirred at 50 °C for 16 h. Purification by flash chromatography on silica gel (EtOAc/hexane, 3 : 97 v/v) afford product **9** (34 mg, 75%) as a colorless oil.

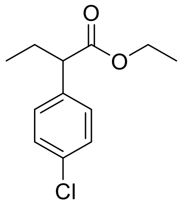

^{1}H NMR (500 MHz, $CDCl_3$): 7.29 (2 H, d, *J* = 8.6 Hz), 7.25 (2 H, d, *J* = 8.3 Hz), 4.28–3.86 (2 H, m), 3.42 (1 H, t, *J* = 7.7 Hz), 2.08 (1 H, dquint., *J* = 14.9, 7.7 Hz), 1.77 (1 H, dquint., *J* = 14.8, 7.6 Hz), 1.22 (3 H, t, *J* = 7.0 Hz), 0.89 (3 H, t, *J* = 7.2 Hz) ppm. – ^{13}C NMR (125 MHz, $CDCl_3$): 173.8, 137.8, 133.1, 129.5, 128.8, 60.9, 53.0, 26.9, 14.3, 12.2 ppm. – IR: 3003, 2965, 2921, 1711, 1420, 1361, 1221, 1091 cm^{-1}. – HRMS calcd for $C_{12}H_{16}ClO_2$: 227.0833, found 227.0829 [M+H$^+$].

Ethyl 2-(4-bromophenyl)butanoate (10)

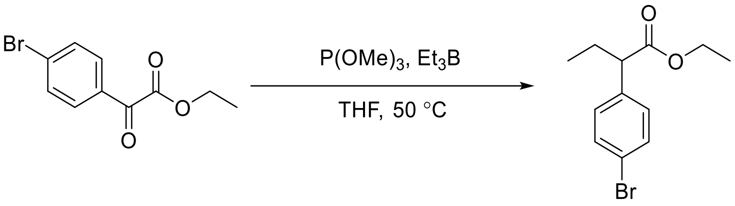

The general procedure GP1 was followed with ethyl 2-(4-bromophenyl)-2-oxoacetate (51.2 mg, 0.2 mmol), $P(OMe)_3$ (30 mg, 0.24 mmol, 1.2 equiv.), Et_3B (0.3 mL, 1M, 1.5 equiv.) and THF (1.7 mL). The mixture was stirred at 50 °C for 16 h. Purification by flash chromatography on silica gel (EtOAc/hexane, 3 : 97 v/v) afford product **10** (38 mg, 70%) as a colorless oil.

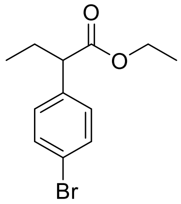

^{1}H NMR (500 MHz, $CDCl_3$): 7.51 (2 H, d, *J* = 8.5 Hz), 7.26 (2 H, d, *J* = 8.5 Hz), 4.56–3.97 (2 H, m), 3.46 (1 H, t, *J* = 7.7 Hz), 2.19–2.08 (1 H, m), 1.88–1.77 (1 H, m), 1.28 (3 H, t, *J* = 7.1 Hz), 0.95 (3 H, t, *J* = 7.4 Hz) ppm. – ^{13}C NMR (125 MHz, $CDCl_3$): 173.7, 138.3, 131.7, 129.8, 121.2, 60.9, 53.1, 26.8, 14.3, 12.2 ppm. – IR: 2968, 2941, 1731, 1488, 1367, 1199, 1159, 1073, 1010 cm^{-1}. – HRMS calcd for $C_{12}H_{16}BrO_2$: 271.0328, found 271.0319 [M+H$^+$].

Ethyl 2-(*p*-tolyl)butanoate (11)

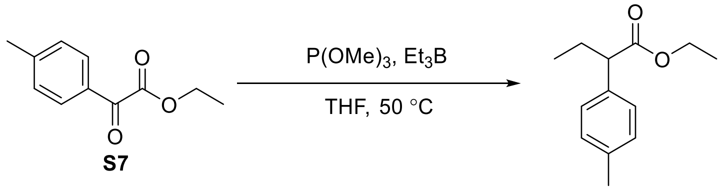

The general procedure GP1 was followed with ethyl 2-oxo-2-(*p*-tolyl)acetate (38.4 mg, 0.2 mmol), $P(OMe)_3$ (30 mg, 0.24 mmol, 1.2 equiv.), Et_3B (0.3 mL, 1M, 1.5 equiv.) and THF (1.7 mL). The mixture was stirred at 50 °C for 16 h. Purification by flash chromatography on silica gel (EtOAc/hexane, 3 : 97 v/v) afford product **11** (33 mg, 81%) as a colorless oil.

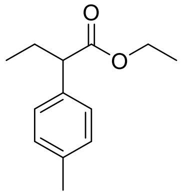

^{1}H NMR (500 MHz, $CDCl_3$): 7.20 (2 H, d, J = 8.2 Hz), 7.13 (2 H, d, J = 7.9 Hz), 4.21–4.04 (2 H, m), 3.41 (1 H, t, J = 7.7 Hz), 2.33 (3 H, s), 2.08 (1 H, dt, J = 13.5, 7.5 Hz), 1.77 (1 H, dt, J = 13.6, 7.3 Hz), 1.21 (3 H, t, J = 7.1 Hz), 0.90 (3 H, t, J = 7.4 Hz) ppm. – ^{13}C NMR (125 MHz, $CDCl_3$): 174.4, 136.8, 136.4, 129.3, 127.9, 60.7, 53.3, 26.9, 21.2, 14.3, 12.3 ppm. – IR: 2967, 2928, 2878, 1732, 1513, 1457, 1368, 1199, 1159, 1021 cm^{-1}. – HRMS calcd for $C_{13}H_{19}O_2$: 207.1380, found 237.1375 [M+H$^+$].

Ethyl 2-(*m*-tolyl)butanoate (12)

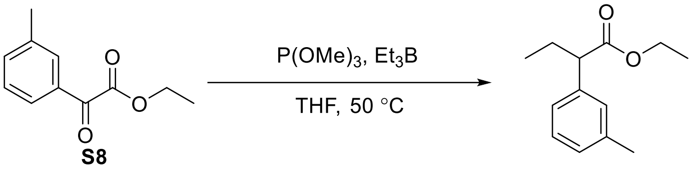

The general procedure GP1 was followed with ethyl 2-oxo-2-(*m*-tolyl)acetate (38.4 mg, 0.2 mmol), $P(OMe)_3$ (30 mg, 0.24 mmol, 1.2 equiv.), Et_3B (0.3 mL, 1M, 1.5 equiv.) and THF (1.7 mL). The mixture was stirred at 50 °C for 16 h. Purification by flash chromatography on silica gel (EtOAc/hexane, 3 : 97 v/v) afford product **12** (30 mg, 72%) as a colorless oil.

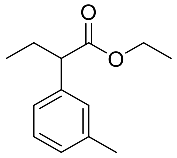

^{1}H NMR (500 MHz, $CDCl_3$): 7.24–7.03 (4 H, m), 4.30–3.98 (2 H, m), 3.40 (1 H, t, J = 7.7 Hz), 2.34 (3 H, s), 2.09 (1 H, dt, J = 14.5, 7.4 Hz), 1.78 (1 H, dt, J = 14.1, 7.2 Hz), 1.22 (3 H, t, J = 7.1 Hz), 0.90 (3 H, t, J = 7.4 Hz) ppm. – ^{13}C NMR (125 MHz, $CDCl_3$): 174.3, 139.3, 138.2, 128.8, 128.5, 128.0, 125.1, 60.7, 53.6, 27.0, 21.6, 14.3, 12.3 ppm. – IR: 2968, 2934, 2882, 1712, 1362, 1221, 1180, 1089, 910 cm^{-1}. – HRMS calcd for $C_{13}H_{19}O_2$: 207.1380, found 237.1376 [M+H$^+$].

Ethyl 2-(4-isobutylphenyl)butanoate (13)

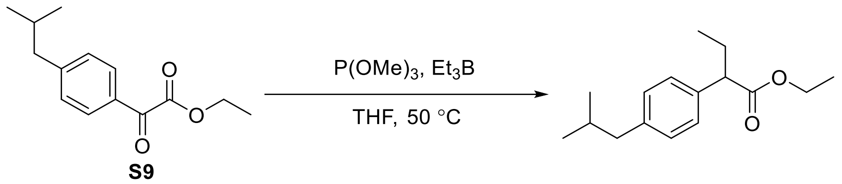

The general procedure GP1 was followed with ethyl 2-(4-isobutylphenyl)-2-oxoacetate (46.8 mg, 0.2 mmol), $P(OMe)_3$ (30 mg, 0.24 mmol, 1.2 equiv.), Et_3B (0.3 mL, 1M, 1.5 equiv.) and THF (1.7 mL). The mixture was stirred at 50 °C for 16 h. Purification by flash chromatography on silica gel (EtOAc/hexane, 3 : 97 v/v) afford product **13** (45 mg, 90%, or 43 mg, 86% outside glovebox) as a colorless oil.

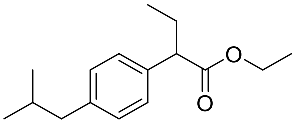

^{1}H NMR (500 MHz, $CDCl_3$): 7.21 (2 H, d, *J* = 8.2 Hz), 7.09 (2 H, d, *J* = 8.1 Hz), 4.49–3.93 (2 H, m), 3.41 (1 H, t, *J* = 7.7 Hz), 2.45 (2 H, d, *J* = 7.2 Hz), 2.08 (1 H, dq, *J* = 15.0, 7.5 Hz), 1.86 (1 H, dq, *J* = 13.6, 6.8 Hz), 1.83–1.73 (1 H, m), 1.21 (3 H, t, *J* = 7.1 Hz), 0.90 (6 H, d, *J* = 6.7 Hz), 0.90 (3 H, t, *J* = 7.4 Hz) ppm. – ^{13}C NMR (125 MHz, $CDCl_3$): 174.4, 140.6, 136.6, 129.3, 127.7, 60.6, 53.3, 45.2, 30.3, 27.0, 22.5, 14.3, 12.3 ppm. – IR: 2958, 2930, 2872, 1734, 1458, 1367, 1160 cm^{-1}. – HRMS calcd for $C_{16}H_{25}O_2$: 249.1849, found 249.1854 [M+H$^+$].

Ethyl 2-(4-methoxyphenyl)butanoate (14)

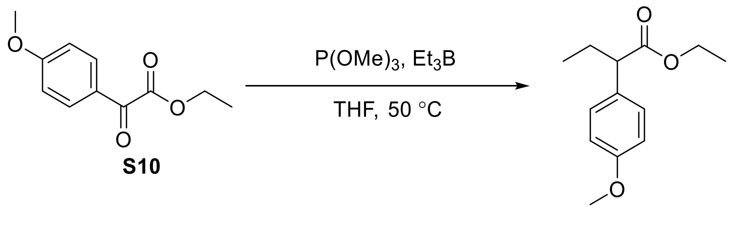

The general procedure GP1 was followed with ethyl 2-(4-methoxyphenyl)-2-oxoacetate (41.6 mg, 0.2 mmol), $P(OMe)_3$ (30 mg, 0.24 mmol, 1.2 equiv.), Et_3B (0.3 mL, 1M, 1.5 equiv.) and THF (1.7 mL). The mixture was stirred at 50 °C for 16 h. Purification by flash

chromatography on silica gel (EtOAc/hexane, 20 : 80 v/v) afford product **14** (38 mg, 86%) as a colorless oil.

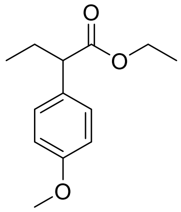

^{1}H NMR (500 MHz, $CDCl_3$): 7.23 (2 H, d, *J* = 8.7 Hz), 6.85 (2 H, d, *J* = 8.7 Hz), 4.30–4.01 (2 H, m), 3.79 (3 H, s), 3.38 (1 H, t, *J* = 7.7 Hz), 2.17–1.94 (1 H, m), 1.84–1.69 (1 H, m), 1.21 (3 H, t, *J* = 7.2 Hz), 0.88 (3 H, t, *J* = 7.4 Hz) ppm. – ^{13}C NMR (125 MHz, $CDCl_3$): 174.5, 158.8, 131.5, 129.0, 114.0, 114.0, 60.7, 55.3, 52.8, 27.0, 14.3, 12.3 ppm. – IR: 2967, 2939, 1731, 1512, 1457, 1369, 1250, 1159, 1036, 831 cm^{-1}. – HRMS calcd for $C_{13}H_{19}O_3$: 223.1329, found 223.1330 [M+H$^+$].

Ethyl 2-(3,4-dimethoxyphenyl)butanoate (15)

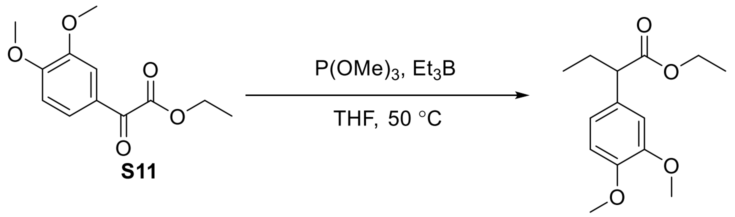

The general procedure GP1 was followed with ethyl 2-(3,4-dimethoxyphenyl)-2-oxoacetate (47.6 mg, 0.2 mmol), $P(OMe)_3$ (30 mg, 0.24 mmol, 1.2 equiv.), Et_3B (0.3 mL, 1M, 1.5 equiv.) and THF (1.7 mL). The mixture was stirred at 50 °C for 16 h. Purification by flash chromatography on silica gel (EtOAc/hexane, 20 : 80 v/v) afford product **15** (36 mg, 71%, or 33 mg, 70% outside glovebox) as a colorless oil.

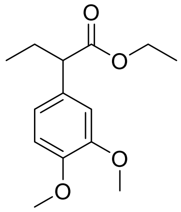

^{1}H NMR (500 MHz, $CDCl_3$): 6.87–6.79 (3 H, m), 4.23–4.01 (2 H, m), 3.87 (3 H, s), 3.85 (3 H, s), 3.36 (1 H, t, *J* = 7.7 Hz), 2.06 (1 H, dquint., *J* = 14.8, 7.3 Hz), 1.76 (1 H, dquint., *J* = 14.6, 7.2 Hz), 1.21 (3 H, t, *J* = 7.1 Hz), 0.89 (3 H, t, *J* = 7.4 Hz) ppm. – ^{13}C NMR (125 MHz, $CDCl_3$): 174.4, 149.0, 148.2, 131.9, 120.3, 111.2, 111.0, 60.7, 56.0, 53.2, 27.0, 14.3, 12.3 ppm. – IR: 2968, 2943, 1729, 1516, 1464, 1261, 1230, 1154, 1027 cm^{-1}. – HRMS calcd for $C_{14}H_{21}O_4$: 253.1434, found 253.1433 [M+H$^+$].

Ethyl 2-(3-fluoro-4-methoxyphenyl)butanoate (16)

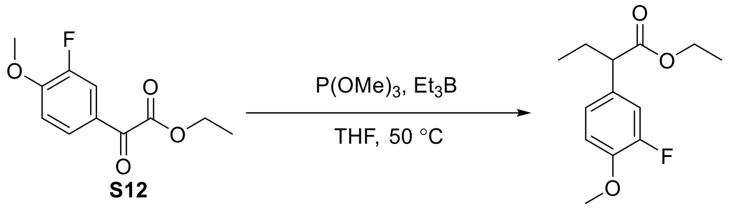

The general procedure GP1 was followed with ethyl 2-(3-fluoro-4-methoxyphenyl)-2-oxoacetate (45.2 mg, 0.2 mmol), $P(OMe)_3$ (30 mg, 0.24 mmol, 1.2 equiv.), Et_3B (0.3 mL, 1M, 1.5 equiv.) and THF (1.7 mL). The mixture was stirred at 50 °C for 16 h. Purification by flash chromatography on silica gel (EtOAc/hexane, 15 : 85 v/v) afford product **16** (35.5 mg, 74%) as a colorless oil.

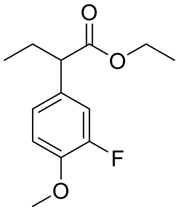

^{1}H NMR (500 MHz, $CDCl_3$): 7.07 (1 H, dd, J = 12.3, 2.2 Hz), 7.02–6.96 (1 H, m), 6.89 (1 H, t, J = 8.5 Hz), 4.28–4.02 (2 H, m), 3.87 (3 H, s), 3.35 (1 H, t, J = 7.7 Hz), 2.03 (1 H, dq, J = 14.9, 7.4 Hz), 1.75 (1 H, dq, J = 14.5, 7.4 Hz), 1.21 (3 H, t, J = 7.1 Hz), 0.88 (3 H, t, J = 7.3 Hz) ppm. – ^{13}C NMR (125 MHz, $CDCl_3$): 174.0, 152.4 (d, J = 245.7 Hz), 146.8 (d, J = 10.8 Hz), 132.3 (d, J = 6.0 Hz), 123.8 (d, J = 3.6 Hz), 115.7 (d, J = 18.7 Hz), 113.4 (d, J = 2.1 Hz), 60.8, 56.4, 52.7, 26.9, 14.3, 12.2 ppm. – ^{19}F NMR (470.5 Hz, $CDCl_3$): −135.0 (dd, J = 12.4, 8.7 Hz) ppm. – IR: 2969, 2937, 1731, 1586, 1518, 1273, 1219, 1176, 1130, 1024 cm^{-1}. – HRMS calcd for $C_{13}H_{18}FO_3$: 241.1234, found 241.1238 [$M+H^+$].

Ethyl 2-(2-fluoro-4,5-dimethoxyphenyl)butanoate (17)

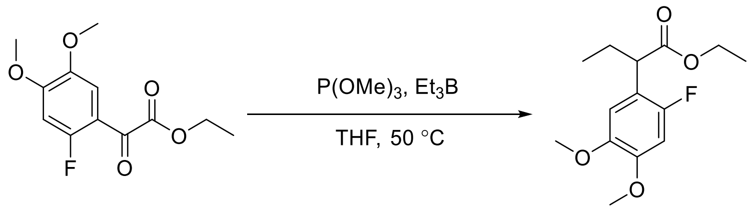

Go back to table of contents

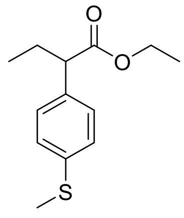

The general procedure GP1 was followed with ethyl 2-(2-fluoro-4,5-dimethoxyphenyl)-2-oxoacetate (**S1**) (51.2 mg, 0.2 mmol), $P(OMe)_3$ (30 mg, 0.24 mmol, 1.2 equiv.), Et_3B (0.3 mL, 1M, 1.5 equiv.) and THF (1.7 mL). The mixture was stirred at 50 °C for 16 h. Purification by flash chromatography on silica gel (EtOAc/hexane, 15 : 85 v/v) afford product **17** (34 mg, 63%) as a colorless oil.

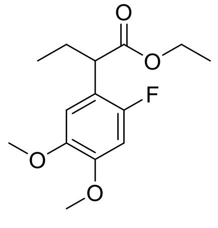

^{1}H NMR (500 MHz, $CDCl_3$): 6.82 (1 H, d, *J* = 6.9 Hz), 6.60 (1 H, d, *J* = 11.1 Hz), 4.34–4.00 (2 H, m), 3.83 (6 H, s), 3.76 (1 H, t, *J* = 7.7 Hz), 2.05 (1 H, dquint., *J* = 14.6, 7.3 Hz), 1.74 (1 H, dq, *J* = 14.1, 7.5 Hz), 1.21 (3 H, t, *J* = 7.1 Hz), 0.88 (3 H, t, *J* = 7.4 Hz) ppm. – ^{13}C NMR (125 MHz, $CDCl_3$): 173.8, 154.8 (d, *J* = 238.6 Hz), 148.8 (d, *J* = 9.9 Hz), 145.4 (d, *J* = 2.7 Hz), 116.7 (d, *J* = 16.3 Hz), 110.8 (d, *J* = 5.4 Hz), 99.9 (d, *J* = 29.0 Hz), 60.8, 56.5, 56.2, 44.8, 26.2, 14.3, 12.0 ppm. – ^{19}F NMR (470.5 Hz, $CDCl_3$): –125.9 (dd, *J* = 11.1, 6.9 Hz) ppm. – IR: 2969, 2934, 1731, 1517, 1448, 1213, 1192, 1109, 1024, 997 cm^{-1}. – HRMS calcd for $C_{14}H_{20}FO_4$: 271.1340, found 271.1344 [$M+H^+$].

Ethyl 2-(4-(methylthio)phenyl)butanoate (18)

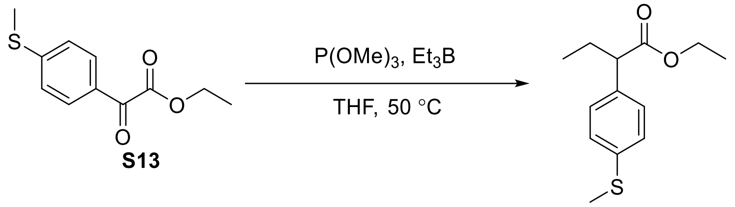

The general procedure GP1 was followed with ethyl 2-(4-(methylthio)phenyl)-2-oxoacetate (44.8 mg, 0.2 mmol), $P(OMe)_3$ (30 mg, 0.24 mmol, 1.2 equiv.), Et_3B (0.3 mL, 1M, 1.5 equiv.) and THF (1.7 mL). The mixture was stirred at 50 °C for 16 h. Purification by flash chromatography on silica gel (EtOAc/hexane, 3 : 97 v/v) afford product **18** (39.5 mg, 83%) as a colorless oil.

^{1}H NMR (500 MHz, $CDCl_3$): 7.29–7.14 (4 H, m), 4.28–3.93 (2 H, m), 3.39 (1 H, t, *J* = 7.7 Hz), 2.47 (3 H, s), 2.07 (1 H, dq, *J* = 15.0, 7.5 Hz), 1.77 (1 H, dq, *J* = 14.4, 7.3 Hz), 1.21 (3 H, t, *J* =

7.1 Hz), 0.89 (3 H, t, *J* = 7.3 Hz) ppm. – ^{13}C NMR (125 MHz, $CDCl_3$): 174.1, 137.2, 136.3, 128.6, 126.9, 60.8, 53.1, 26.9, 16.0, 14.3, 12.3 ppm. – IR: 2966, 2930, 1731, 1494, 1440, 1368, 1202, 1160, 1095, 1016 cm^{-1}. – HRMS calcd for $C_{13}H_{19}O_2S$: 239.1100, found 239.1100 [M+H$^+$].

Ethyl 2-(4-cyanophenyl)butanoate (19)

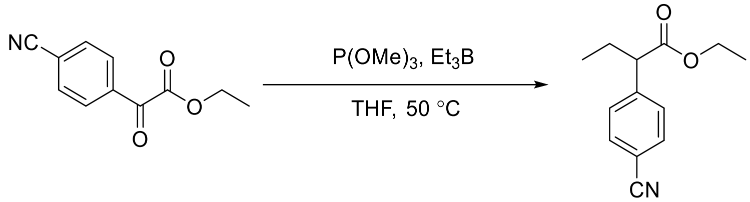

The general procedure GP2 was followed with ethyl 2-(4-cyanophenyl)-2-oxoacetate (40.6 mg, 0.2 mmol), $P(OMe)_3$ (30 mg, 0.24 mmol, 1.2 equiv.), Et_3B (0.3 mL, 1M, 1.5 equiv.) and THF (1.7 mL). The mixture was stirred at 50 °C for 16 h. Purification by flash chromatography on silica gel (EtOAc/hexane, 3 : 97 v/v) afford product **19** (25 mg, 58%) as a colorless oil.

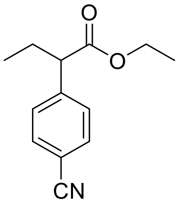

^{1}H NMR (500 MHz, $CDCl_3$): 7.61 (2 H, d, *J* = 8.4 Hz), 7.42 (2 H, d, *J* = 8.4 Hz), 4.39–3.87 (2 H, m), 3.49 (1 H, t, *J* = 7.8 Hz), 2.10 (1 H, dquint, *J* = 15.0, 7.4 Hz), 1.79 (1 H, dquint., *J* = 14.8, 7.5 Hz), 1.21 (3 H, t, *J* = 7.2 Hz), 0.89 (3 H, t, *J* = 7.4 Hz) ppm. – ^{13}C NMR (125 MHz, $CDCl_3$): 173.0, 144.7, 132.5, 129.0, 118.9, 111.3, 61.2, 53.7, 26.9, 14.2, 12.2 ppm. – IR: 2985, 1740, 1447, 1373, 1239, 1045, 917, 847, 734 cm^{-1}. – HRMS calcd for $C_{13}H_{16}NO_2$: 218.1176, found 218.1174 [M+H$^+$].

Ethyl 2-(3-(trifluoromethyl)phenyl)butanoate (20)

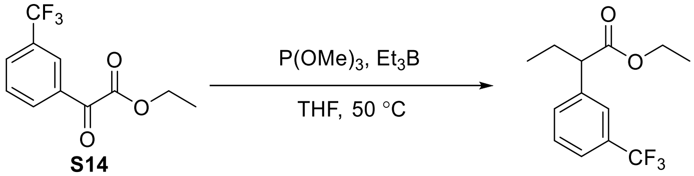

S14

Go back to table of contents

The general procedure GP2 was followed with ethyl 2-oxo-2-(3-(trifluoromethyl)phenyl)acetate (49.2 mg, 0.2 mmol), $P(OMe)_3$ (30 mg, 0.24 mmol, 1.2 equiv.), Et_3B (0.3 mL, 1M, 1.5 equiv.) and THF (1.7 mL). The mixture was stirred at 50 °C for 16 h. Purification by flash chromatography on silica gel (EtOAc/hexane, 3 : 97 v/v) afford product **20** (39 mg, 75%) as a colorless oil.

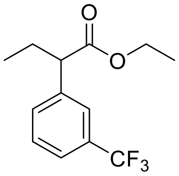

^{1}H NMR (500 MHz, $CDCl_3$): 7.57 (1 H, s), 7.52 (2 H, d, *J* = 8.1 Hz), 7.44 (1 H, t, *J* = 7.7 Hz), 4.29–3.98 (2 H, m), 3.50 (1 H, t, *J* = 7.7 Hz), 2.12 (1 H, dq, *J* = 15.0, 7.5 Hz), 1.81 (1 H, dq, *J* = 14.7, 7.3 Hz), 1.22 (3 H, t, *J* = 7.2 Hz), 0.90 (3 H, t, *J* = 7.4 Hz) ppm. – ^{13}C NMR (125 MHz, $CDCl_3$): 173.5, 140.3, 131.5, 131.0 (q, *J* = 32.1 Hz), 129.1, 125.0 (q, *J* = 3.8 Hz), 124.2 (q, *J* = 272.4 Hz), 124.2 (q, *J* = 3.7 Hz), 61.0, 53.5, 27.0, 14.2, 12.2 ppm. – ^{19}F NMR (470.5 Hz, $CDCl_3$): –62.6 ppm. – IR: 2984, 1740, 1373, 1240, 1045, 917, 846, 733 cm^{-1}. – HRMS calcd for $C_{13}H_{16}F_3O_2$: 261.1097, found 261.1095 [$M+H^+$].

Ethyl 2-(3-(trifluoromethoxy)phenyl)butanoate (21)

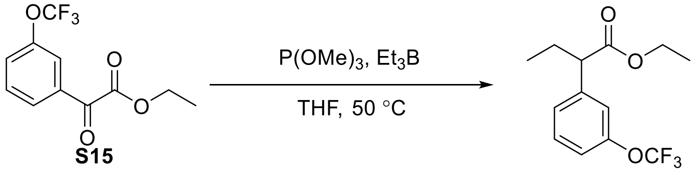

The general procedure GP1 was followed with ethyl 2-oxo-2-(3-(trifluoromethoxy)phenyl)acetate (52.4 mg, 0.2 mmol), $P(OMe)_3$ (30 mg, 0.24 mmol, 1.2 equiv.), Et_3B (0.3 mL, 1M, 1.5 equiv.) and THF (1.7 mL). The mixture was stirred at 50 °C for 16 h. Purification by flash chromatography on silica gel (EtOAc/hexane, 3 : 97 v/v) afford product **21** (43 mg, 78%) as a colorless oil.

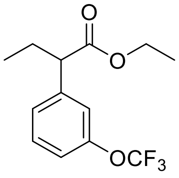

^{1}H NMR (500 MHz, $CDCl_3$): 7.41 (1 H, t, *J* = 7.9 Hz), 7.32 (1 H, d, *J* = 7.8 Hz), 7.26 (1 H, s), 7.19 (1 H, d, *J* = 8.1 Hz), 4.34–4.09 (2 H, m), 3.53 (1 H, t, *J* = 7.7 Hz), 2.16 (1 H, dq, *J* = 14.9, 7.4 Hz), 1.86 (1 H, dquint., *J* = 14.6, 7.4 Hz), 1.29 (3 H, t, *J* = 7.1 Hz), 0.97 (3 H, t, *J* = 7.4 Hz) ppm. – ^{13}C NMR (125 MHz, $CDCl_3$): 173.5, 149.5,

141.6, 129.9, 126.6, 120.8, 120.6 (q, *J* = 257.2 Hz), 119.7, 61.0, 53.4, 26.9, 14.2, 12.2 ppm. – ^{19}F NMR (470.5 Hz, $CDCl_3$): –57.8 ppm. – IR: 2969, 2934, 2889, 1735, 1448, 1370, 1258, 1216, 1160, 1024 cm^{-1}. – HRMS calcd for $C_{13}H_{16}F_3O_3$: 277.1046, found 277.1049 [M+H+].

Ethyl 2-([1,1'-biphenyl]-4-yl)butanoate (22)

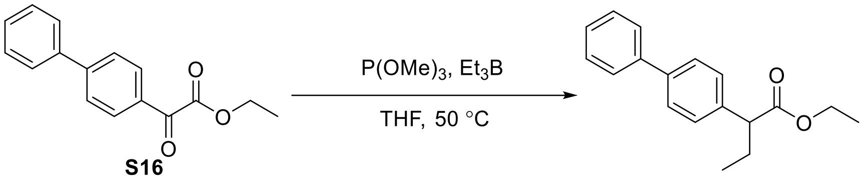

The general procedure GP1 was followed with ethyl 2-([1,1'-biphenyl]-4-yl)-2-oxoacetate (50.8 mg, 0.2 mmol), $P(OMe)_3$ (30 mg, 0.24 mmol, 1.2 equiv.), Et_3B (0.3 mL, 1M, 1.5 equiv.) and THF (1.7 mL). The mixture was stirred at 50 °C for 16 h. Purification by flash chromatography on silica gel (EtOAc/hexane, 3 : 97 v/v) afford product **22** (45 mg, 84%, or 43 mg, 80% outside glovebox) as a colorless oil.

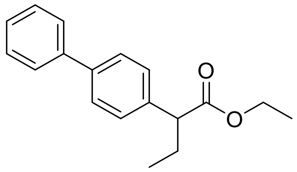

^{1}H NMR (500 MHz, $CDCl_3$): 8.41 (1 H, d, *J* = 9.3 Hz), 8.24–8.13 (4 H, m), 8.10–7.97 (4 H, m), 4.60 (1 H, t, *J* = 7.5 Hz), 4.28–4.03 (2 H, m), 2.59–2.29 (1 H, m), 2.20–1.98 (1 H, m), 1.16 (3 H, t, *J* = 7.1 Hz), 1.01 (3 H, t, *J* = 7.4 Hz) ppm. – ^{13}C NMR (125 MHz, $CDCl_3$): 174.2, 140.9, 140.1, 138.4, 128.9, 128.5, 127.4, 127.1, 60.8, 53.3, 27.0, 14.3, 12.3 ppm. – IR: 2965, 2936, 2878, 1730, 1485, 1368, 1263, 1159, 1021, 839 cm^{-1}. – HRMS calcd for $C_{18}H_{21}O_2$: 269.1536, found 269.1534 [M+H+].

Ethyl 2-(2-fluoro-[1,1'-biphenyl]-4-yl)butanoate (23)

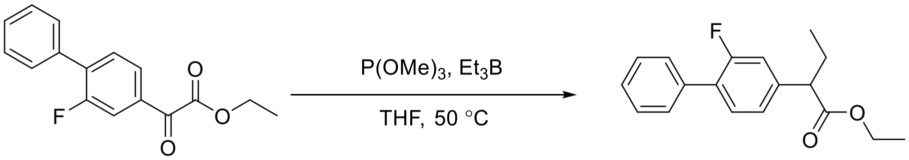

The general procedure GP1 was followed with ethyl 2-(2-fluoro-[1,1'-biphenyl]-4-yl)-2-oxoacetate (**S2**) (54.4 mg, 0.2 mmol), $P(OMe)_3$ (30 mg, 0.24 mmol, 1.2 equiv.), Et_3B (0.3 mL,

1M, 1.5 equiv.) and THF (1.7 mL). The mixture was stirred at 50 °C for 16 h. Purification by flash chromatography on silica gel (EtOAc/hexane, 3 : 97 v/v) afford product **23** (40 mg, 70%) as a colorless oil.

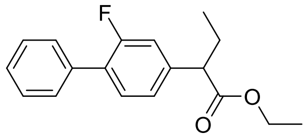

^{1}H NMR (500 MHz, $CDCl_3$): 7.55 (2 H, d, *J* = 7.8 Hz), 7.44 (2 H, t, *J* = 7.7 Hz), 7.41–7.34 (2 H, m), 7.19–7.13 (2 H, m), 4.30–4.05 (2 H, m), 3.48 (1 H, t, *J* = 7.7 Hz), 2.12 (1 H, dq, *J* = 14.9, 7.4 Hz), 1.85 (1 H, dq, *J* = 14.4, 7.3 Hz), 1.26 (3 H, t, *J* = 7.1 Hz), 0.95 (3 H, t, *J* = 7.4 Hz) ppm. – ^{13}C NMR (125 MHz, $CDCl_3$): 173.7, 159.8 (d, *J* = 248.2 Hz), 140.7 (d, *J* = 7.7 Hz), 135.7, 130.8 (d, *J* = 4.0 Hz), 129.0 (d, *J* = 3.1 Hz), 128.6, 127.9 (d, *J* = 13.6 Hz), 127.8, 124.2 (d, *J* = 3.5 Hz), 115.7 (d, *J* = 23.6 Hz), 61.0, 53.1, 26.9, 14.3, 12.3 ppm. – ^{19}F NMR (470.5 Hz, $CDCl_3$): –117.8 ppm. – IR: 2971, 2969, 2876, 1732, 1483, 1419, 1263, 1179, 1022 cm^{-1}. – HRMS calcd for $C_{18}H_{20}FO_2$: 287.1442, found 287.1444 [M+H$^+$].

Ethyl 2-(5,6,7,8-tetrahydronaphthalen-2-yl)butanoate (24)

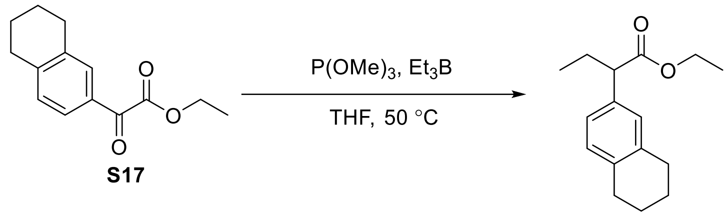

The general procedure GP1 was followed with ethyl 2-oxo-2-(5,6,7,8-tetrahydronaphthalen-2-yl)acetate (46.4 mg, 0.2 mmol), $P(OMe)_3$ (30 mg, 0.24 mmol, 1.2 equiv.), Et_3B (0.3 mL, 1M, 1.5 equiv.) and THF (1.7 mL). The mixture was stirred at 50 °C for 16 h. Purification by flash chromatography on silica gel (EtOAc/hexane, 3 : 97 v/v) afford product **24** (45 mg, 92%) as a colorless oil.

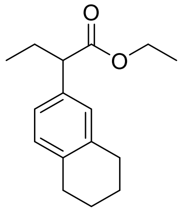

^{1}H NMR (500 MHz, $CDCl_3$): 7.10–6.96 (3 H, m), 4.17 (1 H, dq, *J* = 10.8, 7.1 Hz), 4.08 (1 H, dq, *J* = 10.8, 7.1 Hz), 3.37 (1 H, t, *J* = 7.7 Hz), 2.75 (4 H, t, *J* = 5.8 Hz), 2.15–2.01 (1 H, m), 1.87–1.70 (5 H, m), 1.23 (3 H, t, *J* = 7.1 Hz), 0.91 (3 H, t, *J* = 7.3 Hz) ppm. – ^{13}C NMR (125 MHz, $CDCl_3$): 174.4, 137.3, 136.4, 136.1, 129.4, 128.7, 125.1, 60.7, 53.3, 29.5, 29.2, 27.0, 23.3, 23.3, 14.3, 12.4 ppm. – IR: 2971, 2931, 1734, 1500, 1366, 1216 cm^{-1}. – HRMS calcd for $C_{16}H_{23}O_2$: 247.1693, found 247.1694 [M+H^+].

Ethyl 2-(6-methoxynaphthalen-2-yl)butanoate (25)

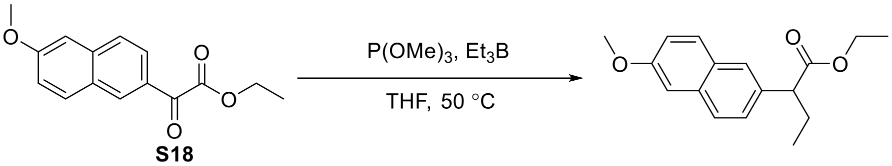

The general procedure GP1 was followed with ethyl 2-(6-methoxynaphthalen-2-yl)-2-oxoacetate (51.6 mg, 0.2 mmol), $P(OMe)_3$ (30 mg, 0.24 mmol, 1.2 equiv.), Et_3B (0.3 mL, 1M, 1.5 equiv.) and THF (1.7 mL). The mixture was stirred at 50 °C for 16 h. Purification by flash chromatography on silica gel (EtOAc/hexane, 15 : 85 v/v) afford product **25** (34 mg, 62%) as a colorless oil.

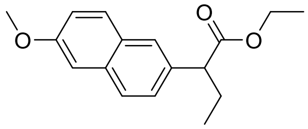

^{1}H NMR (500 MHz, $CDCl_3$): 7.80–7.66 (3 H, m), 7.44 (1 H, dd, *J* = 8.5, 1.8 Hz), 7.26–7.05 (2 H, m), 4.46–4.05 (2 H, m), 3.91 (3 H, s), 3.59 (1 H, t, *J* = 7.7 Hz), 2.19 (1 H, dquint., *J* = 14.8, 7.4 Hz), 1.90 (1 H, dquint., *J* = 14.6, 7.4 Hz), 1.22 (3 H, t, *J* = 7.1 Hz), 0.94 (3 H, t, *J* = 7.4 Hz) ppm. – ^{13}C NMR (125 MHz, $CDCl_3$): 174.3, 157.7, 134.5, 133.8, 129.4, 129.0, 127.1, 126.7, 126.6, 119.0, 105.7, 60.7, 55.4, 53.6, 26.8, 14.3, 12.3 ppm. – IR: 2964, 2937, 1729, 1605, 1482, 1265, 1176, 1029, 852 cm^{-1}. – HRMS calcd for $C_{17}H_{21}O_3$: 273.1485, found 273.1489 [M+H^+].

Ethyl 2-(1,2-dihydroacenaphthylen-5-yl)butanoate (26)

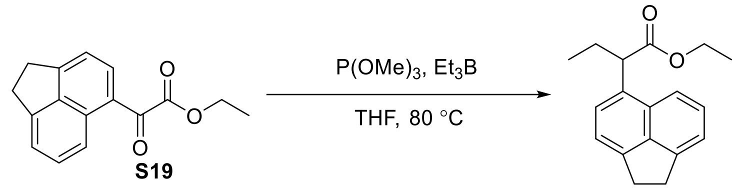

The general procedure GP1 was followed with ethyl 2-(1,2-dihydroacenaphthylen-5-yl)-2-oxoacetate (50.8 mg, 0.2 mmol), $P(OMe)_3$ (50 mg, 0.4 mmol, 2 equiv.), Et_3B (0.4 mL, 1M, 2 equiv.) and THF (3.6 mL). The mixture was stirred at 80 °C for 16 h. Purification by flash chromatography on silica gel (EtOAc/hexane, 3 : 97 v/v) afford product **26** (27 mg, 50%) as a colorless oil.

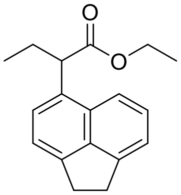

^{1}H NMR (500 MHz, $CDCl_3$): 7.82 (1 H, d, *J* = 8.4 Hz), 7.57–7.41 (2 H, m), 7.35–7.23 (2 H, m), 4.28–3.92 (3 H, m), 3.45–3.35 (4 H, m), 2.28 (1 H, dquint., *J* = 13.6, 6.6, 6.1 Hz), 1.96 (1 H, dquint., *J* = 14.3, 8.5, 7.8 Hz), 1.19 (3 H, t, *J* = 7.2 Hz), 0.99 (3 H, t, *J* = 7.4 Hz) ppm. – ^{13}C NMR (125 MHz, $CDCl_3$): 174.6, 146.7, 145.4, 139.7, 131.4, 130.5, 128.0, 126.4, 119.3, 119.1, 60.8, 48.4, 30.6, 30.0, 26.5, 14.3, 12.7 ppm. – IR: 2969, 2926, 1731, 1364, 1218, 1184, 1025 cm^{-1}. – HRMS calcd for $C_{18}H_{21}O_2$: 269.1536, found 269.1532 [M+H$^+$].

Ethyl 2-(pyren-1-yl)butanoate (27)

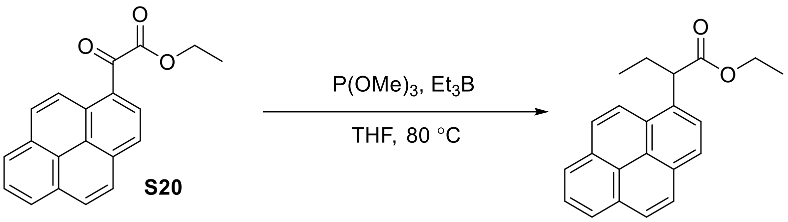

The general procedure GP1 was followed with ethyl 2-oxo-2-(pyren-1-yl)acetate (60.4 mg, 0.2 mmol), $P(OMe)_3$ (50 mg, 0.4 mmol, 2 equiv.), Et_3B (0.4 mL, 1M, 2 equiv.) and THF (3.6 mL). The mixture was stirred at 80 °C for 16 h. Purification by flash chromatography on silica gel (EtOAc/hexane, 3 : 97 v/v) afford product **27** (43 mg, 68%) as a colorless oil.

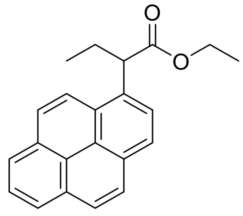

^{1}H NMR (500 MHz, $CDCl_3$): 8.41 (1 H, d, *J* = 9.3 Hz), 8.24–8.13 (4 H, m), 8.10–7.97 (4 H, m), 4.60 (1 H, t, *J* = 7.5 Hz), 4.28–4.03 (2 H, m), 2.59–2.29 (1 H, m), 2.20–1.98 (1 H, m), 1.16 (3 H, t, *J* = 7.1 Hz), 1.01 (3 H, t, *J* = 7.4 Hz) ppm. – ^{13}C NMR (125 MHz, $CDCl_3$): 174.5, 133.5, 131.5, 130.9, 130.5, 129.1, 127.9, 127.6, 127.4, 126.1, 125.4, 125.3, 125.2, 125.1, 125.0, 122.9, 61.0, 49.0, 27.1, 14.3, 12.7 ppm. – IR: 2967, 2926, 1728, 1459, 1366, 1180, 1113, 1022, 844 cm^{-1}. – HRMS calcd for $C_{22}H_{21}O_2$: 317.1536, found 317.1528 [M+H$^+$].

Ethyl 2-(thiophen-2-yl)butanoate (28)

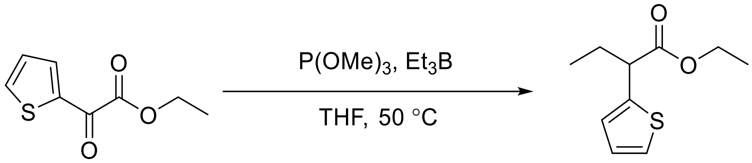

S21

The general procedure GP1 was followed with ethyl 2-oxo-2-(thiophen-2-yl)acetate (37 mg, 0.2 mmol), $P(OMe)_3$ (50 mg, 0.4 mmol, 2 equiv.), Et_3B (0.4 mL, 1M, 2 equiv.) and THF (1.7 mL). The mixture was stirred at 50 °C for 16 h. Purification by flash chromatography on silica gel (EtOAc/hexane, 3 : 97 v/v) afford product **28** (20 mg, 51%) as a colorless oil.

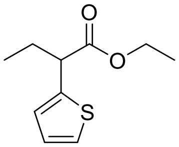

^{1}H NMR (500 MHz, $CDCl_3$): 7.19 (1 H, t, *J* = 3.4 Hz), 6.98–6.89 (2 H, m), 4.23–4.10 (2 H, m), 3.75 (1 H, t, *J* = 7.7 Hz), 2.09 (1 H, dquint., *J* = 15.0, 7.5 Hz), 1.86 (1 H, dquint., *J* = 14.8, 7.5 Hz), 1.25 (3 H, t, *J* = 7.2 Hz), 0.94 (3 H, t, *J* = 7.4 Hz) ppm. – ^{13}C NMR (125 MHz, $CDCl_3$): 173.3, 141.8, 126.6, 125.4, 124.5, 61.1, 48.9, 28.2, 14.3, 12.2 ppm. – IR: 3008, 1714, 1421, 1362, 1221, 1092, 898 cm^{-1}. – HRMS calcd for $C_{10}H_{15}O_2S$: 199.0787, found 199.0791 [M+H$^+$].

Ethyl 2-(5-methylthiophen-2-yl)butanoate (29)

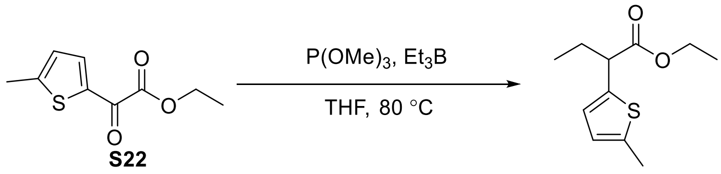

The general procedure GP1 was followed with ethyl 2-(5-methylthiophen-2-yl)-2-oxoacetate (40 mg, 0.2 mmol), $P(OMe)_3$ (50 mg, 0.4 mmol, 2 equiv.), Et_3B (0.4 mL, 1M, 2 equiv.) and THF (1.7 mL). The mixture was stirred at 80 °C for 16 h. Purification by flash chromatography on silica gel (EtOAc/hexane, 3 : 97 v/v) afford product **29** (19 mg, 45%) as a colorless oil.

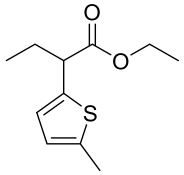

^{1}H NMR (500 MHz, $CDCl_3$): 6.70 (1 H, d, *J* = 3.4 Hz), 6.57 (1 H, dd, *J* = 3.5, 1.1 Hz), 4.30–3.93 (2 H, m), 3.65 (1 H, t, *J* = 7.6 Hz), 2.44 (3 H, d, *J* = 1.1 Hz), 2.05 (1 H, dq, *J* = 14.9, 7.5 Hz), 1.83 (1 H, dq, *J* = 14.5, 7.3 Hz), 1.25 (3 H, t, *J* = 7.1 Hz), 0.93 (3 H, t, *J* = 7.3 Hz) ppm. – ^{13}C NMR (125 MHz, $CDCl_3$): 173.4, 139.3, 139.0, 125.2, 124.7, 61.0, 49.1, 28.0, 15.4, 14.3, 12.2 ppm. – IR: 2973, 2910, 1712, 1364, 1223, 911, 730 cm^{-1}. – HRMS calcd for $C_{11}H_{17}O_2S$: 193.1223, found 193.1222 [M+H$^+$].

Ethyl 2-(3-((5-(4-fluorophenyl)thiophen-2-yl)methyl)-4-methylphenyl)butanoate (30)

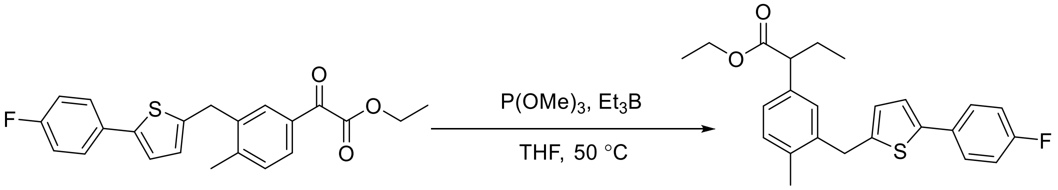

The general procedure GP1 was followed with ethyl 2-(3-((5-(4-fluorophenyl)thiophen-2-yl)methyl)-4-methylphenyl)-2-oxoacetate (**S5**) (76.4 mg, 0.2 mmol), $P(OMe)_3$ (30 mg, 0.24 mmol, 1.2 equiv.), Et_3B (0.3 mL, 1M, 1.5 equiv.) and THF (1.7 mL). The mixture was stirred at 50 °C for 16 h. Purification by flash chromatography on silica gel (EtOAc/hexane, 3 : 97 v/v) afford product **30** (63 mg, 80%) as a colorless oil.

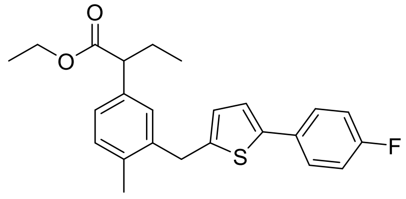

^1H NMR (500 MHz, $CDCl_3$): 7.53–7.41 (2 H, m), 7.22–7.10 (3 H, m), 7.03 (3 H, td, *J* = 8.6, 7.9, 2.9 Hz), 6.66 (1 H, d, *J* = 3.6 Hz), 4.41–3.91 (4 H, m), 3.43 (1 H, t, *J* = 7.8 Hz), 2.30 (3 H, s), 2.09 (1 H, dq, *J* = 15.0, 7.5 Hz), 1.80 (1 H, dquint., *J* = 14.7, 7.4 Hz), 1.22 (3 H, t, *J* = 7.2 Hz), 0.92 (3 H, t, *J* = 7.4 Hz) ppm. – ^{13}C NMR (125 MHz, $CDCl_3$): 174.2, 162.2 (d, *J* = 246.7 Hz), 143.6, 141.5, 138.3, 137.3, 135.3, 131.0 (d, *J* = 3.5 Hz), 130.8, 129.3, 127.2 (d, *J* = 8.0 Hz), 126.5, 126.0, 122.8, 115.8 (d, *J* = 21.7 Hz), 60.7, 53.2, 34.3, 26.9, 19.2, 14.3, 12.3 ppm. – ^{19}F NMR (470.5 Hz, $CDCl_3$): –115.2 ppm. – IR: 2966, 2933, 2875, 1730, 1508, 1463, 1230, 1177, 1158, 1096, 1023, 832, 802 cm^{-1}. – HRMS calcd for $C_{24}H_{26}FO_2S$: 397.1632, found 397.1636 [M+H$^+$].

Ethyl 2-(pyridin-3-yl)butanoate (31)

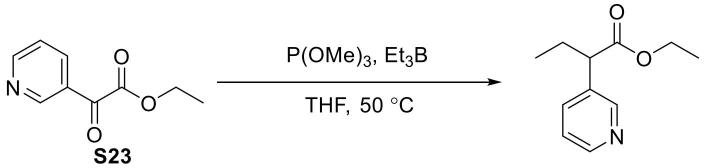

The general procedure GP2 was followed with ethyl 2-oxo-2-(pyridin-3-yl)acetate (36 mg, 0.2 mmol), $P(OMe)_3$ (50 mg, 0.4 mmol, 2.0 equiv.), Et_3B (0.4 mL, 1M, 2.0 equiv.) and THF (3.6 mL). The mixture was stirred at 50 °C for 16 h. Purification by flash chromatography on silica gel (EtOAc/hexane, 10 : 90 v/v) afford product **31** (19 mg, 50%) as a colorless oil.

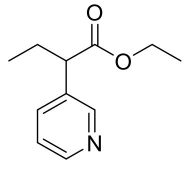

^1H NMR (500 MHz, $CDCl_3$): 8.53 (1 H, s), 8.51 (1 H, d, *J* = 3.8 Hz), 7.68 (1 H, dt, *J* = 7.9, 2.1 Hz), 7.27 (1 H, d, *J* = 6.7 Hz), 4.38–3.84 (2 H, m), 3.46 (1 H, t, *J* = 7.7 Hz), 2.12 (1 H, dquint., *J* = 15.0, 7.5 Hz), 1.80 (1 H, dquint., *J* = 14.8, 7.4 Hz), 1.22 (3 H, t, *J* = 7.1 Hz), 0.91 (3 H, t, *J* = 7.4 Hz) ppm. – ^{13}C NMR (125 MHz, $CDCl_3$): 173.5, 149.9, 148.8, 135.3, 134.9, 123.7, 61.1, 51.1, 26.9, 14.3, 12.2 ppm. – IR: 2967, 2937, 2879, 1732, 1426, 1369, 1191, 1163, 1023, 889 cm^{-1}. – HRMS calcd for $C_{11}H_{16}NO_2$: 194.1176, found 194.1180 [M+H$^+$].

Ethyl 2-(2,3-dihydrobenzofuran-5-yl)butanoate (32)

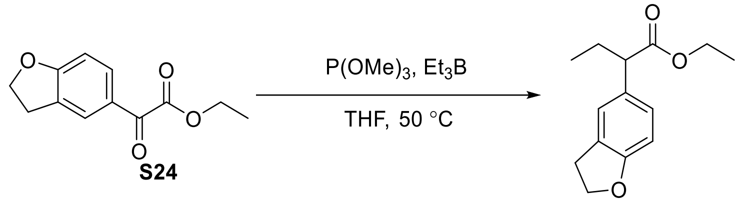

The general procedure GP1 was followed with ethyl 2-(2,3-dihydrobenzofuran-5-yl)-2-oxoacetate (44 mg, 0.2 mmol), $P(OMe)_3$ (30 mg, 0.24 mmol, 1.2 equiv.), Et_3B (0.3 mL, 1M, 1.5 equiv.) and THF (1.7 mL). The mixture was stirred at 50 °C for 16 h. Purification by flash chromatography on silica gel (EtOAc/hexane, 10 : 90 v/v) afford product 3**2** (31 mg, 66%) as a colorless oil.

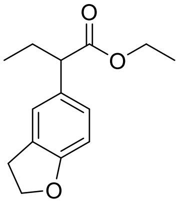

^{1}H NMR (500 MHz, $CDCl_3$): 7.16 (1 H, d, *J* = 1.9 Hz), 7.02 (1 H, dd, *J* = 8.2, 2.0 Hz), 6.71 (1 H, d, *J* = 8.2 Hz), 4.55 (2 H, t, *J* = 8.7 Hz), 4.15 (1 H, dq, *J* = 10.8, 7.1 Hz), 4.08 (1 H, dq, *J* = 10.8, 7.1 Hz), 3.36 (1 H, t, *J* = 7.7 Hz), 3.19 (2 H, t, *J* = 8.7 Hz), 2.04 (1 H, dq, *J* = 14.9, 7.4 Hz), 1.74 (1 H, dquint., *J* = 14.7, 7.4 Hz), 1.22 (3 H, t, *J* = 7.1 Hz), 0.88 (3 H, t, *J* = 7.3 Hz) ppm. – ^{13}C NMR (125 MHz, $CDCl_3$): 174.6, 159.4, 131.4, 127.8, 127.4, 124.4, 109.2, 76.9, 71.4, 60.6, 53.0, 29.9, 27.1, 14.3, 12.3 ppm. – IR: 3002, 2975, 2932, 1712, 1421, 1362, 1222, 1092 cm^{-1}. – HRMS calcd for $C_{14}H_{19}O_3$: 235.1329, found 235.1330 [M+H$^+$].

***tert*-Butyl 3-(1-ethoxy-1-oxobutan-2-yl)-1*H*-indole-1-carboxylate (33)**

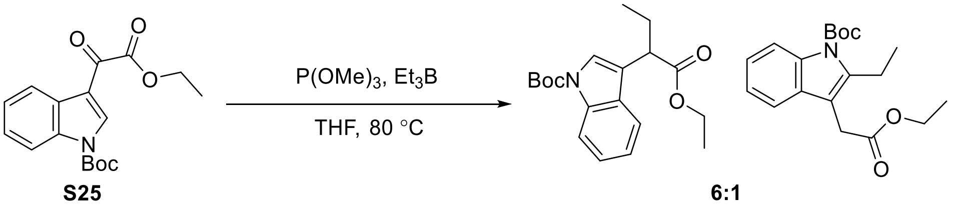

Go back to table of contents

The general procedure GP1 was followed with *tert*-butyl 3-(2-ethoxy-2-oxoacetyl)-1*H*-indole-1-carboxylate (63.4 mg, 0.2 mmol), $P(OMe)_3$ (50 mg, 0.4 mmol, 2 equiv.), Et_3B (0.4 mL, 1M, 2 equiv.) and THF (1.7 mL). The mixture was stirred at 80 °C for 16 h. Purification by flash chromatography on silica gel (EtOAc/hexane, 15 : 85 v/v) afford product 3**3** (28 mg, 43%) as a colorless oil.

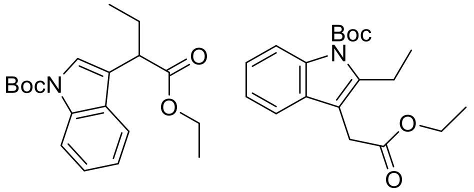

^1H NMR (500 MHz, $CDCl_3$): –Major compound: 8.13 (1 H, s), 7.63 (1 H, d, *J* = 7.8 Hz), 7.54 (1 H, s), 7.31 (1 H, t, *J* = 7.7 Hz), 7.27–7.21 (1 H, m), 4.22–4.05 (2 H, m), 3.71 (1 H, t, *J* = 7.6 Hz), 2.16 (1 H, dquint., *J* = 14.4, 7.3 Hz), 1.95 (1 H, dquint., *J* = 14.4, 7.3 Hz), 1.67 (9 H, s), 1.23 (3 H, t, *J* = 7.1 Hz), 0.99 (3 H, t, *J* = 7.3 Hz) ppm. –Minor compound: 7.70 (1 H, d, *J* = 8.0 Hz), 7.49 (1 H, d, *J* = 8.3 Hz), 7.36 (1 H, t, *J* = 7.8 Hz), 7.05 (1 H, t, *J* = 7.6 Hz), 4.94 (2 H, s), 4.28 (2 H, q, *J* = 7.1 Hz), 2.81 (2 H, q, *J* = 7.4 Hz), 1.53 (9 H, s), 1.37 (3 H, t, *J* = 7.1 Hz), 1.20 (3 H, t, *J* = 7.6 Hz) ppm. – ^{13}C NMR (125 MHz, $CDCl_3$): –Major compound: 173.9, 149.8, 129.8, 124.5, 123.5, 122.6, 119.5, 118.7, 115.4, 60.9, 44.7, 28.6, 28.3, 27.5, 25.6, 14.3, 12.4 ppm. –Minor compound: 168.0, 151.5, 146.9, 135.6, 131.5, 126.3, 122.4, 120.2, 85.3, 83.8, 60.6, 55.8, 29.8, 25.5, 22.0, 14.5, 12.6 ppm. – IR: 3008, 2971, 2253, 1712, 1362, 1221, 912, 730 cm^{-1}. – HRMS calcd for $C_{19}H_{25}NO_4Na$: 354.1676, found 354.1682 [M+Na^+].

Ethyl 2-(1-(2,5-dimethylbenzyl)-1*H*-indol-5-yl)butanoate (34)

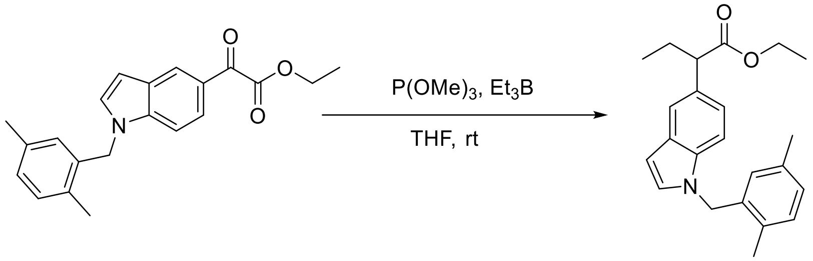

The general procedure GP1 was followed with ethyl 2-(1-(2,5-dimethylbenzyl)-1*H*-indol-5-yl)-2-oxoacetate (**S4**) (67 mg, 0.2 mmol), $P(OMe)_3$ (30 mg, 0.24 mmol, 1.2 equiv.), Et_3B

(0.3 mL, 1M, 1.5 equiv.) and THF (1.7 mL). The mixture was stirred at room temperature for 36 h. Purification by flash chromatography on silica gel (EtOAc/hexane, 3 : 97 v/v) afford product **34** (28 mg, 40%) as a colorless oil.

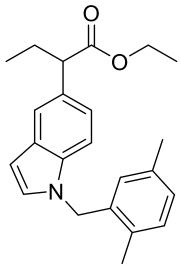

^{1}H NMR (500 MHz, $CDCl_3$): 7.59 (1 H, s), 7.25 (1 H, d, *J* = 10.6 Hz), 7.16 (1 H, dd, *J* = 8.6, 1.8 Hz), 7.09 (1 H, d, *J* = 7.6 Hz), 7.02 (1 H, d, *J* = 7.7 Hz), 6.97 (1 H, d, *J* = 3.2 Hz), 6.70 (1 H, s), 6.49 (1 H, d, *J* = 3.2 Hz), 5.20 (2 H, s), 4.17 (1 H, dq, *J* = 10.4, 7.2 Hz), 4.08 (1 H, dq, *J* = 10.5, 7.1 Hz), 3.53 (1 H, t, *J* = 7.7 Hz), 2.25 (3 H, s), 2.22 (3 H, s), 2.15 (1 H, dquint., *J* = 15.1, 7.4 Hz), 1.85 (1 H, dq, *J* = 15.2, 7.3 Hz), 1.22 (3 H, t, *J* = 7.2 Hz), 0.92 (3 H, t, *J* = 7.4 Hz, 3H) ppm. – ^{13}C NMR (125 MHz, $CDCl_3$): 175.0, 136.0, 135.9, 134.9, 132.9, 130.5, 128.9, 128.7, 128.6, 128.4, 121.9, 120.2, 109.6, 101.6, 60.6, 53.7, 48.3, 27.4, 21.2, 18.8, 14.4, 12.5 ppm. – IR: 2984, 2944, 2913, 1740, 1373, 1240, 1045, 915, 847, 732 cm^{-1}. – HRMS calcd for $C_{23}H_{27}NO_2$: 350.2115, found 350.2124 [$M+H^+$].

Ethyl 2-(9-methyl-9*H*-carbazol-3-yl)butanoate (35)

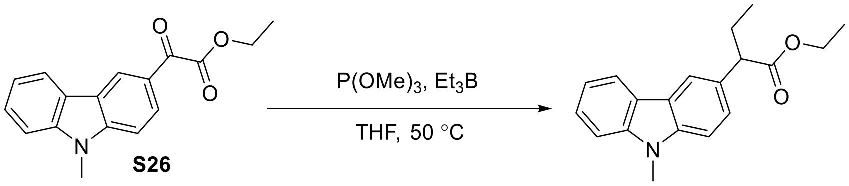

The general procedure GP1 was followed with ethyl 2-(9-methyl-9*H*-carbazol-3-yl)-2-oxoacetate (56.2 mg, 0.2 mmol), $P(OMe)_3$ (30 mg, 0.24 mmol, 1.2 equiv.), Et_3B (0.3 mL, 1M, 1.5 equiv.) and THF (1.7 mL). The mixture was stirred at 50 °C for 16 h. Purification by flash chromatography on silica gel (EtOAc/hexane, 5 : 95 v/v) afford product **35** (40 mg, 68%) as a colorless oil.

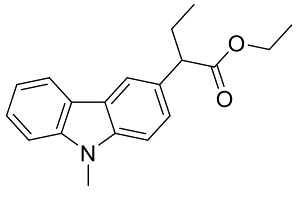

^{1}H NMR (500 MHz, $CDCl_3$ 8.13 (1 H, d, *J* = 7.7 Hz), 8.08 (1 H, d, *J* = 1.7 Hz), 7.53–7.44 (2 H, m), 7.39 (1 H, d, *J* = 8.2 Hz), 7.36 (1 H, d, *J* = 8.4 Hz), 7.26 (1 H, t, *J* = 7.4 Hz), 4.21 (1 H, dq, *J* = 10.8, 7.1 Hz), 4.13

(1 H, dq, *J* = 10.9, 7.1 Hz), 3.83 (3 H, s), 3.66 (1 H, t, *J* = 7.7 Hz), 2.25 (1 H, dquint., *J* = 14.9, 7.4 Hz), 1.95 (1 H, dquint., *J* = 13.8, 7.4 Hz), 1.25 (3 H, t, *J* = 7.1 Hz), 0.98 (3 H, t, *J* = 7.4 Hz) ppm. – ^{13}C NMR (125 MHz, $CDCl_3$): 174.8, 141.4, 140.4, 129.9, 125.8, 125.7, 123.0, 122.7, 120.4, 119.7, 118.9, 108.5, 108.5, 60.6, 53.6, 29.2, 27.3, 14.3, 12.4 ppm. – IR: 2964, 2932, 2874, 1727, 1485, 1471, 1329, 1248, 1183, 1151, 1022, 746 cm^{-1}. – HRMS calcd for $C_{19}H_{21}NO_2$: 296.1645, found 296.1652 [M+H$^+$].

Ethyl 2-(dibenzo[*b*,*d*]thiophen-2-yl)butanoate (36)

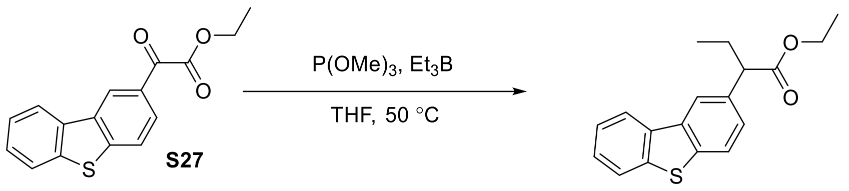

The general procedure GP1 was followed with ethyl 2-(dibenzo[*b*,*d*]thiophen-2-yl)-2-oxoacetate (56.8 mg, 0.2 mmol), $P(OMe)_3$ (30 mg, 0.24 mmol, 1.2 equiv.), Et_3B (0.3 mL, 1M, 1.5 equiv.) and THF (1.7 mL). The mixture was stirred at 50 °C for 16 h. Purification by flash chromatography on silica gel (EtOAc/hexane, 3 : 97 v/v) afford product **36** (49 mg, 83%) as a colorless oil.

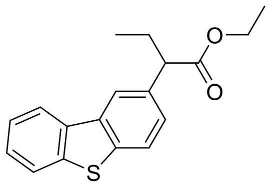

^{1}H NMR (500 MHz, $CDCl_3$): 8.21–8.15 (1 H, m), 8.12 (1 H, d, *J* = 1.9 Hz), 7.87–7.82 (1 H, m), 7.81 (1 H, d, *J* = 8.3 Hz), 7.49–7.39 (3 H, m), 4.27–3.99 (2 H, m), 3.64 (1 H, t, *J* = 7.8 Hz), 2.23 (1 H, dquint., *J* = 14.5, 7.2 Hz), 1.93 (1 H, dq, *J* = 14.9, 7.4 Hz), 1.24 (3 H, t, *J* = 7.1 Hz), 0.96 (3 H, t, *J* = 7.4 Hz) ppm. – ^{13}C NMR (125 MHz, $CDCl_3$): 174.2, 139.9, 138.4, 135.9, 135.8, 135.5, 126.9, 124.4, 122.9, 121.8, 121.1, 60.9, 53.6, 27.2, 14.3, 12.3 ppm. – IR: 2984, 1740, 1373, 1240, 1045, 916, 733 cm^{-1}. – HRMS calcd for $C_{18}H_{19}O_2S$: 299.1100, found 299.1100 [M+H$^+$].

Ethyl 2-(9,9-dimethyl-9*H*-xanthen-2-yl)butanoate (37)

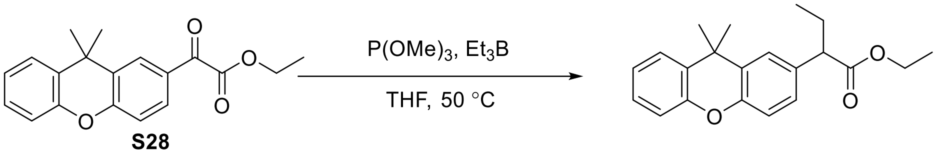

The general procedure GP1 was followed with ethyl 2-(9,9-dimethyl-9*H*-xanthen-2-yl)-2-oxoacetate (62 mg, 0.2 mmol), $P(OMe)_3$ (30 mg, 0.24 mmol, 1.2 equiv.), Et_3B (0.3 mL, 1M, 1.5 equiv.) and THF (1.7 mL). The mixture was stirred at 50 °C for 16 h. Purification by flash chromatography on silica gel (EtOAc/hexane, 3 : 97 v/v) afford product **37** (49 mg, 75%) as a colorless oil.

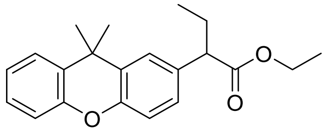

^{1}H NMR (500 MHz, $CDCl_3$): 7.41 (1 H, dd, *J* = 7.8, 1.8 Hz), 7.34 (1 H, d, *J* = 2.3 Hz), 7.20 (1 H, td, *J* = 7.7, 1.7 Hz), 7.16 (1 H, dd, *J* = 8.4, 2.2 Hz), 7.08 (1 H, td, *J* = 7.5, 1.4 Hz), 7.04 (1 H, dd, *J* = 8.1, 1.4 Hz), 7.00 (1 H, d, *J* = 8.4 Hz), 4.22–4.06 (2 H, m), 3.43 (1 H, t, *J* = 7.8 Hz), 2.10 (1 H, dq, *J* = 15.1, 7.5 Hz), 1.79 (1 H, dquint., *J* = 14.6, 7.4 Hz), 1.64 (3 H, s), 1.63 (3 H, s), 1.23 (3 H, t, *J* = 7.1 Hz), 0.92 (3 H, t, *J* = 7.4 Hz) ppm. – ^{13}C NMR (125 MHz, $CDCl_3$): 174.3, 150.5, 149.7, 133.9, 130.1, 130.1, 127.5, 126.9, 126.2, 125.7, 123.1, 116.5, 116.4, 60.7, 53.2, 34.2, 32.5, 32.4, 27.1, 14.3, 12.3 ppm. – IR: 2978, 1734, 1482, 1448, 1372, 1238, 1045, 917, 756, 733 cm^{-1}. – HRMS calcd for $C_{21}H_{25}O_3$: 325.1798, found 325.1795 [$M+H^+$].

3-Ethyl-1-methylindolin-2-one (38)

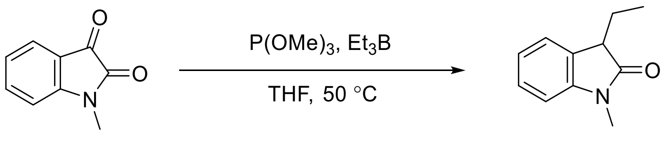

The general procedure GP1 was followed with 1-methylindoline-2,3-dione (16.1 mg, 0.1 mmol), $P(OMe)_3$ (15 mg, 0.12 mmol, 1.2 equiv.), Et_3B (0.2 mL, 1M, 2 equiv.) and THF (3.8

mL). The mixture was stirred at 50 °C for 16 h. Purification by flash chromatography on silica gel (EtOAc/hexane, 20 : 80 v/v) afford product **38** (19 mg, 53%) as a colorless oil.

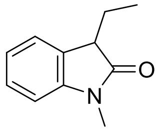

^{1}H NMR (500 MHz, $CDCl_3$): 7.40–7.19 (2 H, m), 7.05 (1 H, td, *J* = 7.5, 1.1 Hz), 6.82 (1 H, d, *J* = 7.7 Hz), 3.41 (1 H, t, *J* = 5.8 Hz), 3.20 (3 H, s), 2.11–1.86 (2 H, m), 0.89 (3 H, t, *J* = 7.5 Hz) ppm. – ^{13}C NMR (125 MHz, $CDCl_3$): 178.0, 144.7, 129.1, 127.9, 123.9, 122.4, 108.0, 46.8, 26.2, 23.9, 10.3 ppm. – IR: 2984, 1739, 1373, 1239, 1044, 917, 732 cm^{-1}. – HRMS calcd for $C_{11}H_{14}NO$: 176.1070, found 176.1069 [M+H$^+$].

Ethyl 2-ethyl-4-phenylbutanoate (39)

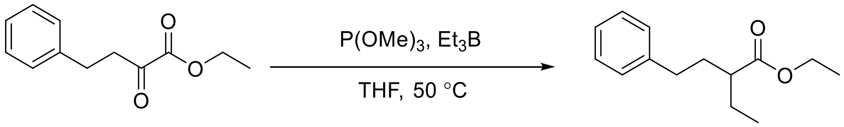

The general procedure GP1 was followed with ethyl 2-oxo-4-phenylbutanoate (41.2 mg, 0.2 mmol), $P(OMe)_3$ (30 mg, 0.24 mmol, 1.2 equiv.), Et_3B (0.3 mL, 1M, 1.5 equiv.) and THF (1.7 mL). The mixture was stirred at 50 °C for 16 h. Purification by flash chromatography on silica gel (EtOAc/hexane, 3 : 97 v/v) afford product **39** (37 mg, 84%) as a colorless oil.

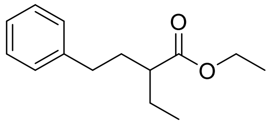

^{1}H NMR (500 MHz, $CDCl_3$): 7.32–7.25 (2 H, m), 7.18 (3 H, t, *J* = 7.4 Hz), 4.16 (2 H, q, *J* = 7.1 Hz), 2.70–2.52 (2 H, m), 2.31 (1 H, ddd, *J* = 14.2, 9.0, 5.3 Hz), 2.01–1.89 (1 H, m), 1.80–1.71 (1 H, m), 1.66 (1 H, dq, *J* = 15.5, 7.6 Hz), 1.59–1.47 (1 H, m), 1.28 (3 H, t, *J* = 7.1 Hz), 0.90 (3 H, t, *J* = 7.4 Hz) ppm. – ^{13}C NMR (125 MHz, $CDCl_3$): 176.2, 142.0, 128.6, 128.6, 128.5, 126.0, 60.2, 46.9, 33.9, 33.9, 25.7, 14.5, 11.9 ppm. – IR: 2965, 2932, 1731, 1454, 1374, 1201, 1158, 1032, 898 cm^{-1}. – HRMS calcd for $C_{14}H_{21}O_2$: 221.1536, found 221.1530 [M+H$^+$].

Ethyl 2-ethyl-4-(4-fluorophenyl)butanoate (40)

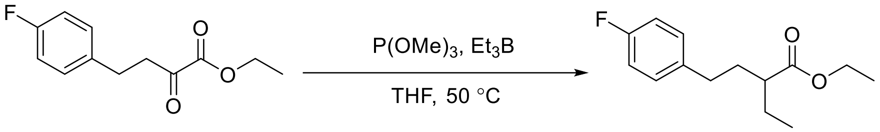

The general procedure GP1 was followed with ethyl 4-(4-fluorophenyl)-2-oxobutanoate (44.8 mg, 0.2 mmol), $P(OMe)_3$ (30 mg, 0.24 mmol, 1.2 equiv.), Et_3B (0.3 mL, 1M, 1.5 equiv.) and THF (1.7 mL). The mixture was stirred at 50 °C for 16 h. Purification by flash chromatography on silica gel (EtOAc/hexane, 3 : 97 v/v) afford product **40** (32 mg, 68%) as a colorless oil.

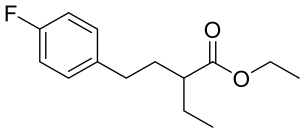

^{1}H NMR (500 MHz, $CDCl_3$): 7.12 (2 H, dd, *J* = 8.5, 5.6 Hz), 6.95 (2 H, t, *J* = 8.7 Hz), 4.16 (2 H, qd, *J* = 7.1, 0.9 Hz), 2.55 (2 H, tq, *J* = 20.5, 7.5, 6.6 Hz), 2.29 (1 H, tt, *J* = 9.2, 5.2 Hz), 1.99–1.88 (1 H, m), 1.77–1.60 (2 H, m), 1.54 (1 H, dq, *J* = 13.5, 6.7, 6.0 Hz), 1.28 (3 H, t, *J* = 7.1 Hz), 0.89 (3 H, t, *J* = 7.5 Hz) ppm. – ^{13}C NMR (125 MHz, $CDCl_3$): 176.1, 161.4 (d, *J* = 243.4 Hz), 137.5 (d, *J* = 3.3 Hz), 129.9 (d, *J* = 7.8 Hz), 115.2 (d, *J* = 21.2 Hz), 60.3, 46.8, 34.0, 33.0, 25.7, 14.5, 11.8 ppm. – ^{19}F NMR (470.5 Hz, $CDCl_3$): –117.7 ppm. – IR: 2985, 1739, 1373, 1238, 1044, 916, 846, 731 cm^{-1}. – HRMS calcd for $C_{14}H_{20}FO_2$: 239.1442, found 239.1445 [M+H$^+$].

Ethyl 4-(2-chlorophenyl)-2-ethylbutanoate (41)

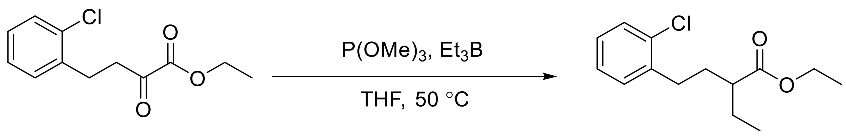

The general procedure GP1 was followed with ethyl 4-(2-chlorophenyl)-2-oxobutanoate (48 mg, 0.2 mmol), $P(OMe)_3$ (30 mg, 0.24 mmol, 1.2 equiv.), Et_3B (0.3 mL, 1M, 1.5 equiv.) and THF (1.7 mL). The mixture was stirred at 50 °C for 16 h. Purification by flash chromatography on silica gel (EtOAc/hexane, 3 : 97 v/v) afford product **41** (36 mg, 70%) as a colorless oil.

Go back to table of contents

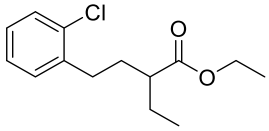

^{1}H NMR (500 MHz, $CDCl_3$): 7.33 (1 H, dd, *J* = 7.8, 1.5 Hz), 7.22–7.09 (3 H, m), 4.17 (2 H, q, *J* = 7.1 Hz), 2.72 (2 H, ddd, *J* = 9.4, 6.5, 3.0 Hz), 2.34 (1 H, tt, *J* = 8.7, 5.3 Hz), 1.98–1.88 (1 H, m), 1.82–1.74 (1 H, m), 1.73–1.63 (1 H, m), 1.62–1.55 (1 H, m), 1.29 (3 H, t, *J* = 7.1 Hz), 0.91 (3 H, t, *J* = 7.4 Hz) ppm. – ^{13}C NMR (125 MHz, $CDCl_3$): 176.0, 139.5, 134.0, 130.6, 129.6, 127.5, 126.9, 60.3, 47.1, 32.0, 31.6, 25.6, 14.5, 11.8 ppm. – IR: 2963, 2937, 2872, 1731, 1474, 1444, 1375, 1190, 1156, 1033, 751 cm^{-1}. – HRMS calcd for $C_{14}H_{20}ClO_2$: 255.1146, found 255.1150 [M+H$^+$].

Ethyl 4-(2,3-dihydrobenzofuran-5-yl)-2-ethylbutanoate (42)

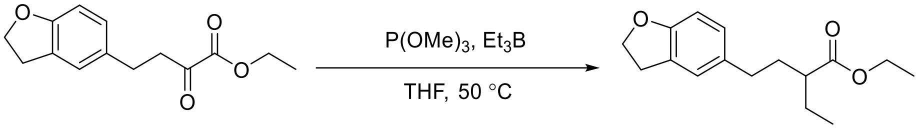

The general procedure GP1 was followed with ethyl 4-(2,3-dihydrobenzofuran-5-yl)-2-oxobutanoate (**S3**) (50 mg, 0.2 mmol), $P(OMe)_3$ (30 mg, 0.24 mmol, 1.2 equiv.), Et_3B (0.3 mL, 1M, 1.5 equiv.) and THF (1.7 mL). The mixture was stirred at 50 °C for 16 h. Purification by flash chromatography on silica gel (EtOAc/hexane, 5 : 95 v/v) afford product 4**2** (34 mg, 65%) as a colorless oil.

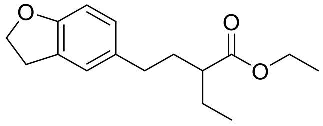

^{1}H NMR (500 MHz, $CDCl_3$): 7.01 (1 H, d, *J* = 1.9 Hz), 6.90 (1 H, dd, *J* = 8.1, 1.8 Hz), 6.69 (1 H, d, *J* = 8.1 Hz), 4.54 (2 H, t, *J* = 8.6 Hz), 4.16 (2 H, q, *J* = 7.1 Hz), 3.17 (2 H, t, *J* = 8.6 Hz), 2.51 (2 H, tq, *J* = 20.3, 7.4, 6.5 Hz), 2.30 (1 H, ddd, *J* = 14.1, 8.9, 5.3 Hz), 1.91 (1 H, ddt, *J* = 14.9, 9.3, 4.6 Hz), 1.77–1.59 (2 H, m), 1.54 (1 H, td, *J* = 13.3, 7.4 Hz), 1.28 (3 H, t, *J* = 7.1 Hz), 0.89 (3 H, t, *J* = 7.4 Hz) ppm. – ^{13}C NMR (125 MHz, $CDCl_3$): 176.2, 158.4, 134.0, 127.9, 127.1, 125.0, 109.1, 71.3, 60.2, 46.9, 34.5, 33.3, 29.9, 25.7, 14.5, 11.9 ppm. – IR: 2961, 1728, 1491, 1375, 1217, 1189, 1153, 1100, 1028, 983 cm^{-1}. – HRMS calcd for $C_{16}H_{23}O_3$: 263.1642, found 263.1640 [M+H$^+$].

1,2-Diphenylbutan-1-one (43)

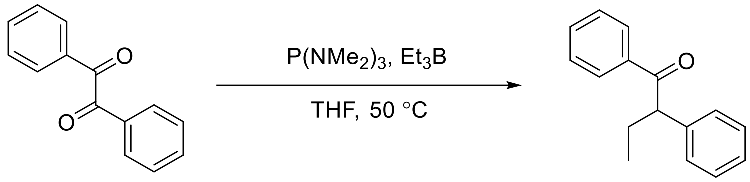

The general procedure GP2 was followed with benzil (42 mg, 0.2 mmol), $P(NMe_2)_3$ (40 mg, 0.24 mmol, 1.2 equiv.), Et_3B (0.3 mL, 1M, 1.5 equiv.) and THF (1.7 mL). The mixture was stirred at 50 °C for 16 h. Purification by flash chromatography on silica gel (EtOAc/hexane, 3 : 97 v/v) afford product **43** (30 mg, 66%) as a colorless oil.

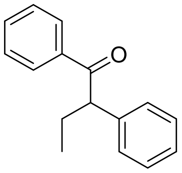

^{1}H NMR (500 MHz, $CDCl_3$): 7.97 (2 H, d, *J* = 7.5 Hz), 7.48 (1 H, t, *J* = 7.4 Hz), 7.39 (2 H, t, *J* = 7.6 Hz), 7.33–7.26 (4 H, m), 7.20 (1 H, t, *J* = 6.7 Hz), 4.45 (1 H, t, *J* = 7.3 Hz), 2.21 (1 H, dquint., *J* = 14.5, 7.3 Hz), 1.87 (1 H, dquint., *J* = 14.7, 7.4 Hz), 0.92 (3 H, t, *J* = 7.4 Hz) ppm. – ^{13}C NMR (125 MHz, $CDCl_3$): 200.2, 139.8, 137.2, 132.9, 129.0, 128.8, 128.6, 128.4, 127.1, 55.6, 27.3, 12.4 ppm. – IR: 2984, 1740, 1373, 1239, 1044, 916, 732 cm^{-1}. – HRMS calcd for $C_{16}H_{17}O$: 225.1274, found 225.1271 [$M+H^+$].

1,2-Bis(4-methoxyphenyl)butan-1-one (44)

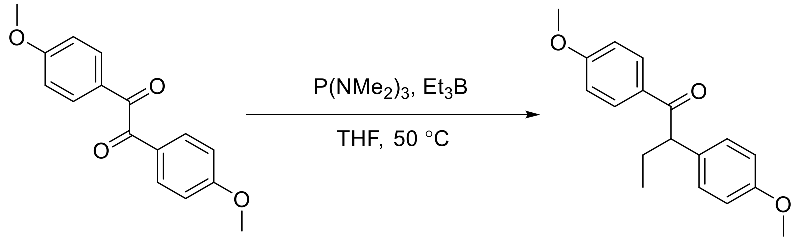

The general procedure GP1 was followed with 1,2-bis(4-methoxyphenyl)ethane-1,2-dione (54 mg, 0.2 mmol), $P(NMe_2)_3$ (40 mg, 0.24 mmol, 1.2 equiv.), Et_3B (0.4 mL, 1M, 2.0 equiv.) and THF (0.6 mL). The mixture was stirred at 50 °C for 16 h. Purification by flash chromatography on silica gel (EtOAc/hexane, 20 : 80 v/v) afford product **44** (50 mg, 88%, or 48 mg, 85% outside glovebox) as a colorless oil.

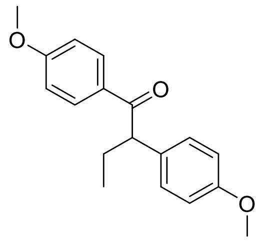

^{1}H NMR (500 MHz, $CDCl_3$): 7.95 (2 H, dd, *J* = 9.0, 2.1 Hz), 7.21 (2 H, dd, *J* = 8.6, 2.0 Hz), 6.87 (2 H, dd, *J* = 9.0, 2.1 Hz), 6.84–6.80 (2 H, m), 4.35 (1 H, td, *J* = 7.2, 1.9 Hz), 3.82 (3 H, s), 3.75 (3 H, s), 2.15 (1 H, dquint., *J* = 14.5, 7.3 Hz), 1.80 (1 H, dq, *J* = 14.7, 7.4 Hz), 0.89 (3 H, t, *J* = 7.3 Hz) ppm. – ^{13}C NMR (125 MHz, $CDCl_3$): 199.0, 163.3, 158.6, 132.3, 131.0, 130.2, 129.3, 114.3, 113.8, 55.6, 55.3, 54.3, 27.2, 12.4 ppm. – IR: 2961, 2937, 1737, 1670, 1599, 1510, 1461, 1253, 1168, 1031, 825 cm^{-1}. – HRMS calcd for $C_{18}H_{21}O_3$: 285.1485, found 285.1479 [M+H$^+$].

1,2-Bis(3-methoxyphenyl)butan-1-one (45)

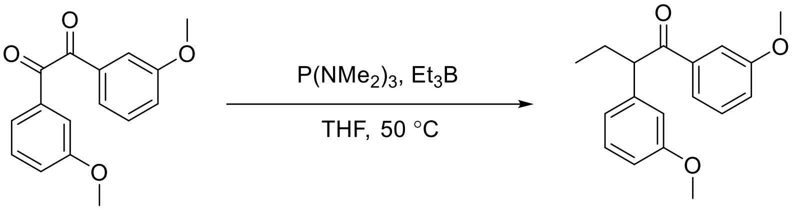

The general procedure GP2 was followed with 1,2-bis(3-methoxyphenyl)ethane-1,2-dione (54 mg, 0.2 mmol), $P(NMe_2)_3$ (65 mg, 0 4 mmol, 2.0 equiv.), Et_3B (0.4 mL, 1M, 2.0 equiv.) and THF (0.6 mL). The mixture was stirred at 50 °C for 16 h. Purification by flash chromatography on silica gel (EtOAc/hexane, 10 : 90 v/v) afford product **45** (28 mg, 50%) as a colorless oil.

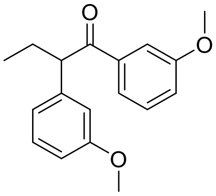

^{1}H NMR (500 MHz, $CDCl_3$): 7.56 (1 H, d, *J* = 7.7 Hz), 7.50 (1 H, s), 7.29 (1 H, t, *J* = 8.0 Hz), 7.21 (1 H, t, *J* = 7.9 Hz), 7.03 (1 H, dd, *J* = 8.2, 2.2 Hz), 6.90 (1 H, d, *J* = 7.6 Hz), 6.84 (1 H, t, *J* = 2.1 Hz), 6.74 (1 H, dd, *J* = 8.2, 2.1 Hz), 4.39 (1 H, t, *J* = 7.2 Hz), 3.80 (3 H, s), 3.76 (3 H, s), 2.19 (1 H, dquint., *J* = 14.6, 7.3 Hz), 1.86 (1 H, dquint., *J* = 14.7, 7.4 Hz), 0.91 (3 H, t, *J* = 7.4 Hz) ppm. – ^{13}C NMR (125 MHz, $CDCl_3$): 199.8, 160.0, 159.8, 141.3, 138.5, 129.9, 129.5, 121.4, 120.9, 119.4, 113.9, 113.1, 112.4, 55.7, 55.4, 55.3, 27.2, 12.4 ppm. – IR: 2982, 1738, 1683, 1597, 1373, 1241, 1045, 916, 732 cm^{-1}. – HRMS calcd for $C_{18}H_{21}O_3$: 285.1485, found 285.1483 [M+H$^+$].

1,2-Di-*p*-tolylbutan-1-one (46)

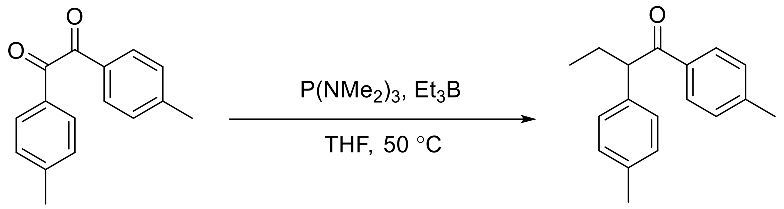

The general procedure GP1 was followed with 1,2-di-*p*-tolylethane-1,2-dione (48 mg, 0.2 mmol), $P(NMe_2)_3$ (40 mg, 0.24 mmol, 1.2 equiv.), Et_3B (0.3 mL, 1M, 1.5 equiv.) and THF (0.7 mL). The mixture was stirred at 50 °C for 16 h. Purification by flash chromatography on silica gel (EtOAc/hexane, 3 : 97 v/v) afford product **46** (42 mg, 83%) as a colorless oil.

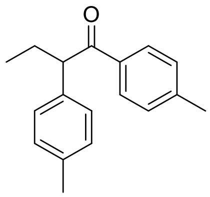

^{1}H NMR (500 MHz, $CDCl_3$): 7.90 (2 H, d, *J* = 8.3 Hz), 7.21 (2 H, d, *J* = 8.1 Hz), 7.19 (2 H, d, *J* = 7.9 Hz), 7.10 (2 H, d, *J* = 7.9 Hz), 4.41 (1 H, t, *J* = 7.3 Hz), 2.35 (3 H, s), 2.29 (3 H, s), 2.21 (1 H, tt, *J* = 14.6, 7.3 Hz), 1.91–1.79 (1 H, m), 0.92 (3 H, t, *J* = 7.4 Hz) ppm. – ^{13}C NMR (125 MHz, $CDCl_3$): 199.9, 143.5, 137.0, 136.5, 134.6, 129.6, 129.2, 128.9, 128.2, 55.0, 27.2, 21.6, 21.1, 12.4 ppm. – IR: 2963, 1736, 1676, 1606, 1512, 1455, 1372, 1239, 1044, 910 cm^{-1}. – HRMS calcd for $C_{18}H_{21}O$: 253.1587, found 253.1586 [$M+H^+$].

1,2-Bis(4-fluorophenyl)butan-1-one (47)

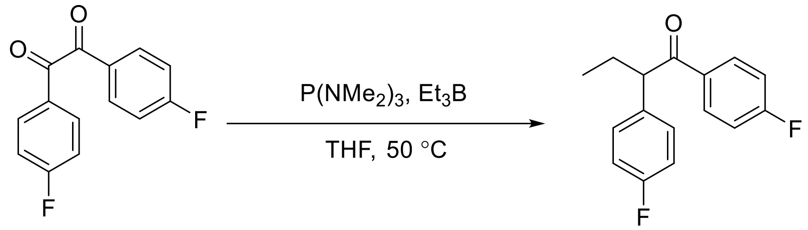

The general procedure GP2 was followed with 1,2-bis(4-fluorophenyl)ethane-1,2-dione (49.2 mg, 0.2 mmol), $P(NMe_2)_3$ (40 mg, 0.24 mmol, 1.2 equiv.), Et_3B (0.3 mL, 1M, 1.5 equiv.) and THF (1.7 mL). The mixture was stirred at 50 °C for 16 h. Purification by flash

chromatography on silica gel (EtOAc/hexane, 3 : 97 v/v) afford product **47** (32 mg, 62%) as a colorless oil.

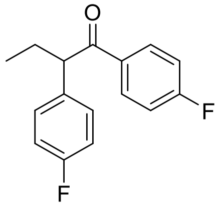

^{1}H NMR (500 MHz, $CDCl_3$): 8.03–7.94 (2 H, m), 7.30–7.20 (2 H, m), 7.06 (2 H, t, *J* = 8.6 Hz), 6.98 (2 H, t, *J* = 8.7 Hz), 4.38 (1 H, t, *J* = 7.3 Hz), 2.16 (1 H, dquint., *J* = 14.5, 7.3 Hz), 1.82 (1 H, dquint., *J* = 14.8, 7.4 Hz), 0.89 (3 H, t, *J* = 7.4 Hz) ppm. – ^{13}C NMR (125 MHz, $CDCl_3$): 198.5, 165.7 (d, *J* = 255.0 Hz), 162.0 (d, *J* = 245.9 Hz), 135.3 (d, *J* = 3.4 Hz), 133.3 (d, *J* = 3.3 Hz), 131.4 (d, *J* = 9.3 Hz), 129.8 (d, *J* = 7.9 Hz), 115.9 (d, *J* = 16.3 Hz), 115.8 (d, *J* = 16.8 Hz), 54.6, 27.3, 12.3 ppm. – ^{19}F NMR (470.5 Hz, $CDCl_3$): –105.3, –115.5 ppm. – IR: 2985, 1740, 1446, 1373, 1239, 1144, 916, 732 cm^{-1}. – HRMS calcd for $C_{16}H_{15}F_2O_2$: 261.1085, found 261.1083 [M+H$^+$].

1,2-Di(thiophen-2-yl)butan-1-one (48)

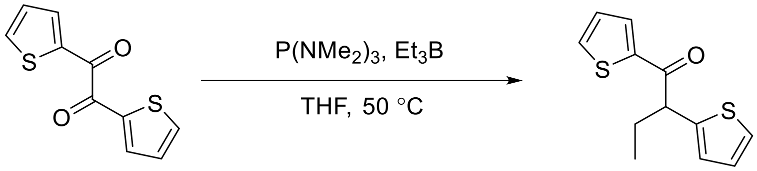

The general procedure GP2 was followed with 1,2-di(thiophen-2-yl)ethane-1,2-dione (22.1 mg, 0.1 mmol), $P(NMe_2)_3$ (20 mg, 0.24 mmol, 1.2 equiv.), Et_3B (0.2 mL, 1M, 2.0 equiv.) and THF (1.8 mL). The mixture was stirred at 50 °C for 16 h. Purification by flash chromatography on silica gel (EtOAc/hexane, 3 : 97 v/v) afford product **48** (27 mg, 58%) as a colorless oil.

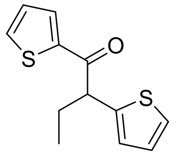

^{1}H NMR (500 MHz, $CDCl_3$): 7.80 (1 H, dd, *J* = 3.8, 1.2 Hz), 7.62 (1 H, dd, *J* = 5.0, 1.2 Hz), 7.18 (1 H, dd, *J* = 5.1, 1.3 Hz), 7.11 (1 H, dd, *J* = 4.9, 3.7 Hz), 6.96 (1 H, dd, *J* = 3.5, 1.3 Hz), 6.93 (1 H, dd, *J* = 5.1, 3.5 Hz), 4.59 (1 H, t, *J* = 7.4 Hz), 2.23 (1 H, dquint., *J* = 14.7, 7.4 Hz), 1.96 (1 H, dq, *J* = 13.7, 7.4 Hz), 0.96 (3 H, t, *J* = 7.4 Hz) ppm. – ^{13}C NMR (125 MHz, $CDCl_3$): 192.0, 143.8, 141.9, 134.3, 132.6, 128.3, 126.9, 125.6,

124.9, 51.5, 28.2, 12.3 ppm. – IR: 2984, 1739, 1446, 1373, 1239, 1045, 917, 846, 734 cm^{-1}. – HRMS calcd for $C_{12}H_{13}OS_2$: 237.0402, found 237.0403 [M+H+].

1,2-Di(furan-2-yl)butan-1-one (49)

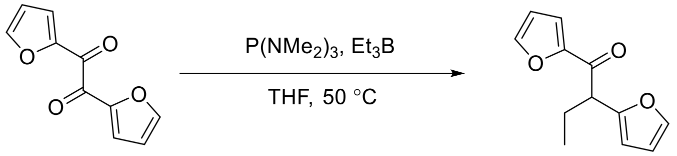

The general procedure GP2 was followed with 1,2-di(furan-2-yl)ethane-1,2-dione (19 mg, 0.1Mmol), $P(NMe_2)_3$ (20 mg, 0.12 mmol, 1.2 equiv.), Et_3B (0.2 mL, 1M, 2.0 equiv.) and THF (3.8 mL). The mixture was stirred at 50 °C for 16 h. Purification by flash chromatography on silica gel (EtOAc/hexane, 3 : 97 v/v) afford product **49** (25 mg, 60%) as a colorless oil.

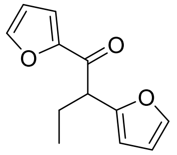

^{1}H NMR (500 MHz, $CDCl_3$): 7.59 (1 H, d, *J* = 1.7 Hz), 7.33 (1 H, d, *J* = 1.9 Hz), 7.21 (1 H, d, *J* = 3.6 Hz), 6.52 (1 H, d, *J* = 1.8 Hz), 6.31 (1 H, s), 6.20 (1 H, d, *J* = 3.2 Hz), 4.38 (1 H, t, *J* = 7.5 Hz), 2.13 (1 H, dquint., *J* = 14.7, 7.4 Hz), 1.98 (1 H, dquint., *J* = 14.7, 7.4 Hz), 0.95 (3 H, t, *J* = 7.4 Hz) ppm. – ^{13}C NMR (125 MHz, $CDCl_3$): 186.8, 152.6, 152.4, 146.8, 142.0, 118.3, 112.5, 110.7, 107.1, 49.1, 24.1, 12.2 ppm. – IR: 2971, 2932, 2884, 1738, 1678, 1566, 1465, 1374, 1217, 1008 cm^{-1}. – HRMS calcd for $C_{12}H_{13}O_3$: 205.0859, found 205.0855 [M+H+].

Ethyl 2,2-diphenylacetate (50)

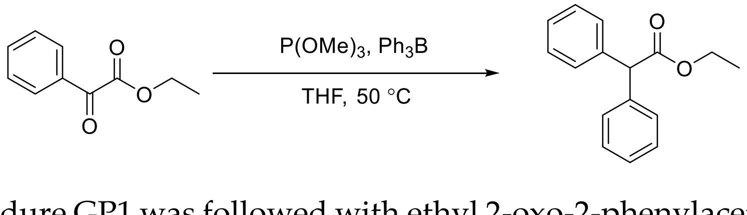

The general procedure GP1 was followed with ethyl 2-oxo-2-phenylacetate (**6**) (36 mg, 0.2 mmol), $P(OMe)_3$ (30 mg, 0.24 mmol, 1.2 equiv.), Ph_3B (73 mg, 0.3 mmol, 1.5 equiv.) and THF (2.0 mL). The mixture was stirred at 50 °C for 16 h. Purification by flash

chromatography on silica gel (EtOAc/hexane, 3 : 97 v/v) afford product **50** (31 mg, 65%) as a colorless oil.

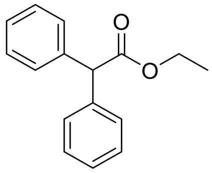

^{1}H NMR (500 MHz, $CDCl_3$): 7.38–7.13 (10 H, m), 4.96 (1 H, s), 4.16 (2 H, q, *J* = 7.1 Hz), 1.20 (3 H, t, *J* = 7.1 Hz) ppm. – ^{13}C NMR (125 MHz, $CDCl_3$): 172.6, 138.9, 128.7, 128.7, 127.4, 61.3, 57.3, 14.3 ppm. – IR: 2985, 1741, 1373, 1239, 1045, 917, 846, 733 cm^{-1}. – HRMS calcd for $C_{16}H_{17}O_2$: 241.1223, found 241.1225 [M+H$^+$].

Ethyl 2-(4-methoxyphenyl)-2-(*p*-tolyl)acetate (51)

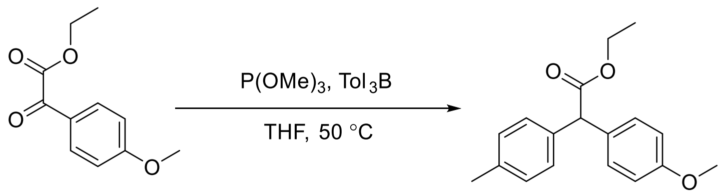

The general procedure GP2 was followed with ethyl 2-(4-methoxyphenyl)-2-oxoacetate (41.6 mg, 0.2 mmol), $P(OMe)_3$ (30 mg, 0.24 mmol, 1.2 equiv.), tol_3B (85 mg, 0.3 mmol, 1.5 equiv.) and THF (2.0 mL). The mixture was stirred at 50 °C for 16 h. Purification by flash chromatography on silica gel (EtOAc/hexane, 10 : 90 v/v) afford product **51** (31 mg, 55%) as a colorless oil.

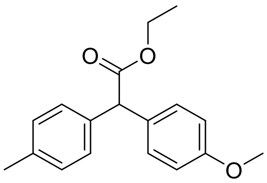

^{1}H NMR (500 MHz, $CDCl_3$): 7.26 (2 H, d, *J* = 8.6 Hz), 7.22 (2 H, d, *J* = 8.0 Hz), 7.15 (2 H, d, *J* = 8.0 Hz), 6.87 (2 H, d, *J* = 8.6 Hz), 4.95 (1 H, s), 4.22 (2 H, q, *J* = 7.1 Hz), 3.81 (3 H, s), 2.34 (3 H, s), 1.28 (3 H, t, *J* = 7.1 Hz) ppm. – ^{13}C NMR (125 MHz, $CDCl_3$): 173.0, 158.8, 136.9, 136.3, 131.3, 129.7, 129.4, 128.5, 114.1, 61.2, 56.1, 55.4, 21.2, 14.3 ppm. – IR: 2983, 2948, 2911, 1737, 1446, 1373, 1238, 1045, 917, 733 cm^{-1}. – HRMS calcd for $C_{18}H_{21}O_3$: 285.1485, found 285.1484 [M+H$^+$].

Ethyl 2-(3,5-dimethylphenyl)-2-(3-(trifluoromethoxy)phenyl)acetate (52)

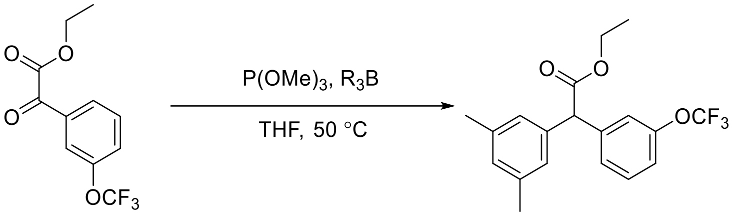

The general procedure GP2 was followed with ethyl 2-oxo-2-(3-(trifluoromethoxy)phenyl)acetate (52.4 mg, 0.2 mmol), $P(OMe)_3$ (30 mg, 0.24 mmol, 1.2 equiv.), tris(3,5-dimethylphenyl)borane (98 mg, 0.3 mmol, 1.5 equiv.) and THF (2.0 mL). The mixture was stirred at 50 °C for 16 h. Purification by flash chromatography on silica gel (EtOAc/hexane, 3 : 97 v/v) afford product 5**2** (35 mg, 50%) as a colorless oil.

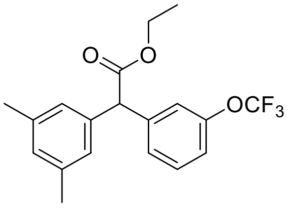

^1H NMR (500 MHz, $CDCl_3$): 7.34 (1 H, t, *J* = 8.0 Hz), 7.25 (1 H, d, *J* = 9.0 Hz), 7.21 (1 H, s), 7.12 (1 H, dd, *J* = 8.5, 2.5 Hz), 6.92 (3 H, s), 4.93 (1 H, s), 4.22 (2 H, qd, *J* = 7.1, 5.2 Hz), 2.30 (6 H, s), 1.26 (3 H, t, *J* = 7.2 Hz) ppm. – ^{13}C NMR (125 MHz, $CDCl_3$): 172.1, 149.4, 141.3, 138.5, 137.8, 129.8, 129.4, 127.2, 126.3, 121.5, 120.6 (q, *J* = 257.2 Hz), 119.6, 61.5, 56.8, 21.5, 14.2 ppm. – ^{19}F NMR (470.5 Hz, $CDCl_3$): –57.7 ppm. – IR: 2983, 2941, 2909, 1739, 1447, 1373, 1241, 1046, 916, 733 cm^{-1}. – HRMS calcd for $C_{19}H_{20}F_3O_3$: 353.1359, found 353.1360 [$M+H^+$].

1,2-Bis(4-methoxyphenyl)-2-phenylethan-1-one (53)

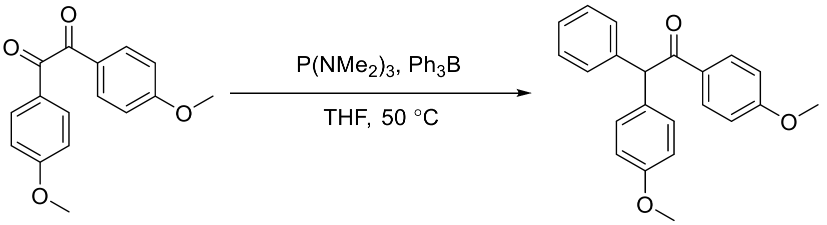

The general procedure GP2 was followed with 1,2-bis(4-methoxyphenyl)ethane-1,2-dione (54 mg, 0.2 mmol), $P(NMe_2)_3$ (40 mg, 0.24 mmol, 1.2 equiv.), Ph_3B (97 mg, 0.4 mmol, 2.0 equiv.) and THF (2.0 mL). The mixture was stirred at 50 °C for 16 h. Purification by

flash chromatography on silica gel (EtOAc/hexane, 15 : 85 v/v) afford product **53** (38 mg, 57%) as a colorless oil.

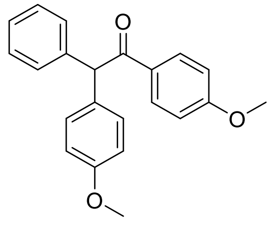

^{1}H NMR (500 MHz, $CDCl_3$): 8.07 (2 H, d, *J* = 8.9 Hz), 7.38 (2 H, t, *J* = 7.5 Hz), 7.34–7.28 (3 H, m), 7.26 (2 H, d, *J* = 8.7 Hz), 6.95 (2 H, d, *J* = 8.9 Hz), 6.93 (2 H, d, *J* = 8.6 Hz), 6.02 (1 H, s), 3.90 (3 H, s), 3.84 (3 H, s) ppm. – ^{13}C NMR (125 MHz, $CDCl_3$): 197.1, 163.5, 158.7, 139.9, 131.6, 131.4, 130.3, 129.9, 129.2, 128.8, 127.1, 114.2, 113.9, 58.4, 55.6, 55.4 ppm. – IR: 2985, 1739, 1446, 1373, 1240, 1045, 916, 733 cm^{-1}. – HRMS calcd for $C_{22}H_{21}O_3$: 333.1485, found 333.1487 [M+H$^+$].

Ethyl 7-chloro-2-phenylheptanoate (54)

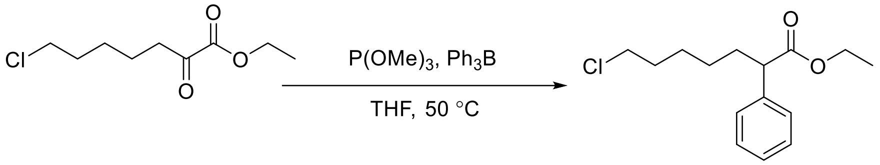

The general procedure GP1 was followed with ethyl 7-chloro-2-oxoheptanoate (41.2 mg, 0.2 mmol), $P(OMe)_3$ (30 mg, 0.24 mmol, 1.2 equiv.), Ph_3B (73 mg, 0.3 mmol, 1.5 equiv.) and THF (2.0 mL). The mixture was stirred at 50 °C for 16 h. Purification by flash chromatography on silica gel (EtOAc/hexane, 3 : 97 v/v) afford product **54** (32 mg, 60%) as a colorless oil.

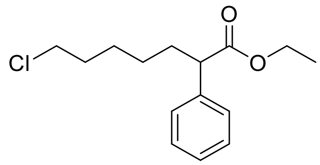

^{1}H NMR (500 MHz, $CDCl_3$): 7.38–7.23 (5 H, m), 4.32–3.99 (2 H, m), 3.53 (3 H, dt, *J* = 10.2, 7.2 Hz), 2.22–2.04 (1 H, m), 1.96–1.71 (3 H, m), 1.56–1.42 (2 H, m), 1.39–1.26 (2 H, m), 1.23 (3 H, t, *J* = 7.1 Hz) ppm. – ^{13}C NMR (125 MHz, $CDCl_3$): 174.1, 139.3, 128.7, 128.0, 127.3, 60.8, 51.8, 45.1, 33.5, 32.5, 26.9, 26.7, 14.3 ppm. – IR: 2984, 2941, 1740, 1373, 1239, 1044, 733 cm^{-1}. – HRMS calcd for $C_{15}H_{22}ClO_2$: 269.1303, found 269.1302 [M+H$^+$].

Ethyl 2,4-diphenylbutanoate (55)

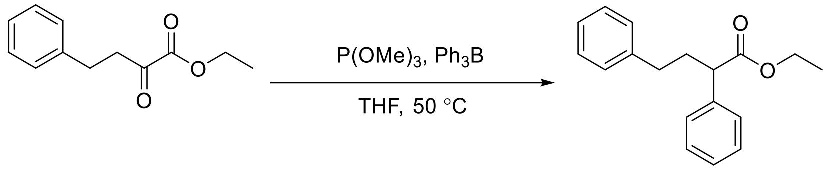

The general procedure GP1 was followed with ethyl 2-oxo-4-phenylbutanoate (41.2 mg, 0.2 mmol), $P(OMe)_3$ (30 mg, 0.24 mmol, 1.2 equiv.), Ph_3B (73 mg, 0.3 mmol, 1.5 equiv.) and THF (2.0 mL). The mixture was stirred at 50 °C for 16 h. Purification by flash chromatography on silica gel (EtOAc/hexane, 3 : 97 v/v) afford product **55** (32 mg, 60%) as a colorless oil.

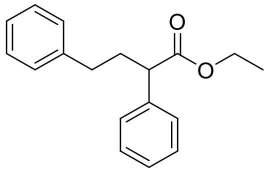

^{1}H NMR (500 MHz, $CDCl_3$): 7.45–7.08 (10 H, m), 4.25–4.00 (2 H, m), 3.56 (1 H, t, *J* = 7.7 Hz), 2.58 (2 H, t, *J* = 7.8 Hz), 2.49–2.36 (1 H, m), 2.11 (1 H, dq, *J* = 15.3, 7.4 Hz), 1.22 (3 H, t, *J* = 7.1 Hz) ppm. – ^{13}C NMR (125 MHz, $CDCl_3$): 173.9, 141.5, 139.1, 128.8, 128.6, 128.5, 128.1, 127.4, 126.1, 60.9, 51.2, 35.1, 33.7, 14.3 ppm. – IR: 2983, 1738, 1446, 1373, 1238, 1045, 917, 734 cm^{-1}. – HRMS calcd for $C_{18}H_{21}O_2$: 269.1536, found 269.1536 [$M+H^+$].

Ethyl 2-phenylhexanoate (56)

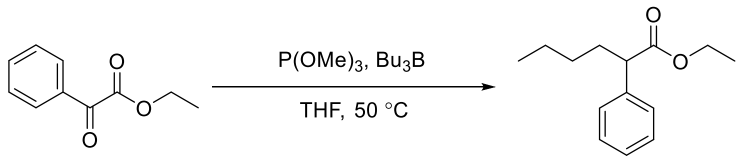

The general procedure GP1 was followed with ethyl 2-oxo-2-phenylacetate (**6**) (36 mg, 0.2 mmol), $P(OMe)_3$ (30 mg, 0.24 mmol, 1.2 equiv.), *n*Bu_3B (0.3 mL, 1M, 1.5 equiv.) and THF (1.7 mL). The mixture was stirred at 50 °C for 16 h. Purification by flash chromatography on silica gel (EtOAc/hexane, 3 : 97 v/v) afford product **56** (35 mg, 80%) as a colorless oil.

Gram scale synthesis: The general procedure GP1 two identical reactions were run with ethyl 2-oxo-2-phenylacetate (6) (890 mg, 5.0 mmol), $P(OMe)_3$ (744 mg, 6.0 mmol, 1.2

equiv.), nBu$_3$B (7.5 mL, 1M, 2.0 equiv.), and THF (40 mL). The mixture was stirred at 50 °C for 16 h. Purification by flash chromatography on silica gel (EtOAc/hexane, 3 : 97 v/v) afford product **56** (1.5 g, 68%) as a colorless oil.

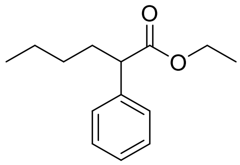

^{1}H NMR (500 MHz, $CDCl_3$): 7.39–7.13 (5 H, m), 4.40–3.88 (2 H, m), 3.52 (1 H, t, J = 7.7 Hz), 2.07 (1 H, dddd, J = 13.2, 9.8, 8.0, 5.3 Hz), 1.87–1.69 (1 H, m), 1.41–1.24 (4 H, m), 1.21 (3 H, t, J = 7.1 Hz), 0.87 (3 H, t, J = 7.2 Hz) ppm. – ^{13}C NMR (125 MHz, $CDCl_3$): 174.3, 139.6, 128.7, 128.1, 127.2, 60.7, 52.0, 33.5, 29.9, 22.6, 14.3, 14.0 ppm. – IR: 2956, 2930, 2860, 1732, 1495, 1454, 1369, 1252, 1161, 1031 cm^{-1}. – HRMS calcd for $C_{14}H_{21}O_2$: 221.1536, found 221.1532 [M+H$^+$].

Prop-2-yn-1-yl 2-phenylhexanoate (57)

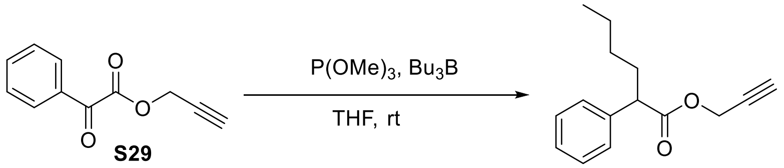

The general procedure GP1 was followed with prop-2-yn-1-yl 2-oxo-2-phenylacetate (38 mg, 0.2 mmol), P(OMe)$_3$ (50 mg, 0.4 mmol, 2 equiv.), nBu$_3$B (0.4 mL, 1M, 2 equiv.) and THF (1.7 mL). The mixture was stirred at room temperature for 16 h. Purification by flash chromatography on silica gel (EtOAc/hexane, 3 : 97 v/v) afford product **57** (23 mg, 50%) as a colorless oil.

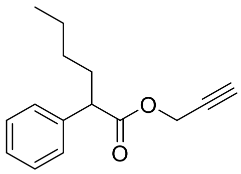

^{1}H NMR (500 MHz, $CDCl_3$): 7.35–7.22 (5 H, m), 4.71 (1 H, dd, J = 15.6, 2.5 Hz), 4.60 (1 H, dd, J = 15.6, 2.5 Hz), 3.58 (1 H, t, J = 7.8 Hz), 2.43 (1 H, t, J = 2.4 Hz), 2.18–2.03 (1 H, m), 1.83–1.76 (1 H, m), 1.42–1.15 (4 H, m), 0.87 (3 H, t, J = 7.2 Hz) ppm. – ^{13}C NMR (125 MHz, $CDCl_3$): 173.5, 138.9, 128.7, 128.1, 127.4, 77.7, 75.0, 52.3, 51.6, 33.5, 29.8, 22.6, 14.0 ppm. – IR: 2985, 1740, 1373, 1239, 1044, 916, 731 cm^{-1}. – HRMS calcd for $C_{15}H_{19}O_2$: 231.1380, found 231.1380 [M+H$^+$].

Ethyl 2-([1,1'-biphenyl]-4-yl)-4-(4-(*tert*-butoxy)phenyl)butanoate (58)

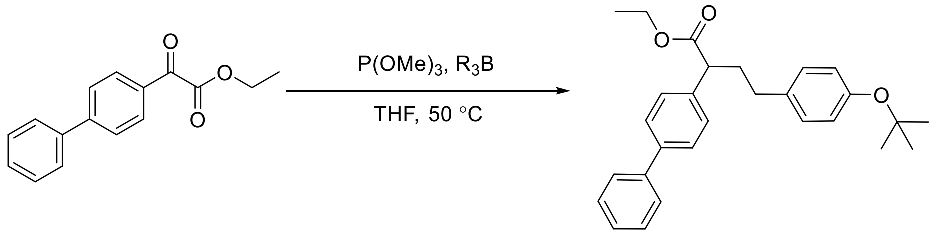

The general procedure GP2 was followed with ethyl 2-([1,1'-biphenyl]-4-yl)-2-oxoacetate (51 mg, 0.2 mmol), $P(OMe)_3$ (50 mg, 0.4 mmol, 2.0 equiv.), tris(4-(*tert*-butoxy)phenethyl)borane (0.4 mL, 1M, 2.0 equiv.) and THF (1.0 mL). The mixture was stirred at 50 °C for 16 h. Purification by flash chromatography on silica gel (EtOAc/hexane, 5 : 95 v/v) afford product **58** (57 mg, 69%) as a colorless oil.

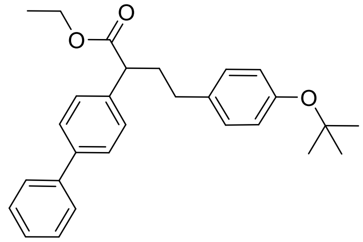

^{1}H NMR (500 MHz, $CDCl_3$): 7.61 (2 H, d, *J* = 7.4 Hz), 7.58 (2 H, d, *J* = 8.1 Hz), 7.45 (2 H, t, *J* = 7.6 Hz), 7.41 (2 H, d, *J* = 8.1 Hz), 7.35 (1 H, t, *J* = 7.3 Hz), 7.08 (2 H, d, *J* = 8.2 Hz), 6.93 (2 H, d, *J* = 8.3 Hz), 4.26–4.05 (2 H, m), 3.62 (1 H, t, *J* = 7.6 Hz), 2.59 (2 H, t, *J* = 7.5 Hz), 2.45 (1 H, dq, *J* = 15.5, 7.8 Hz), 2.14 (1 H, dq, *J* = 15.3, 7.4 Hz), 1.35 (9 H, s), 1.26 (3 H, t, *J* = 7.1 Hz) ppm. – ^{13}C NMR (125 MHz, $CDCl_3$): 173.9, 153.5, 140.8, 140.2, 138.2, 136.3, 128.89, 128.87, 128.5, 127.44, 127.37, 127.1, 124.3, 78.3, 60.9, 50.8, 35.3, 33.1, 29.0, 14.3 ppm. – IR: 2975, 2928, 2870, 1730, 1506, 1487, 1365, 1235, 1164, 896 cm^{-1}. – HRMS calcd for $C_{28}H_{33}O_3$: 434.2690, found 434.2693 [M+H$^+$].

Ethyl 2-(4-isobutylphenyl)-4-(4-methoxyphenyl)butanoate (59)

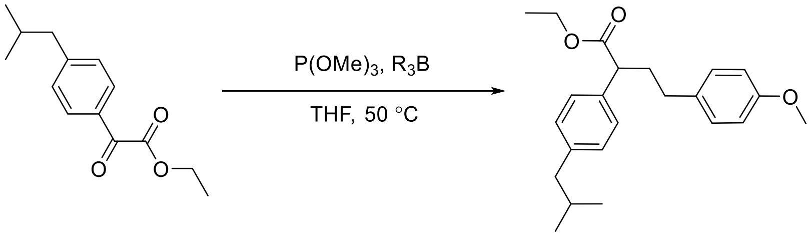

The general procedure GP2 was followed with ethyl 2-(4-isobutylphenyl)-2-oxoacetate (47 mg, 0.2 mmol), $P(OMe)_3$ (50 mg, 0.4 mmol, 2.0 equiv.), tris(4-methoxyphenethyl)borane (0.4 mL, 1M, 2.0 equiv.) and THF (1.0 mL). The mixture was stirred at 50 °C for 16 h. Purification by flash chromatography on silica gel (EtOAc/hexane, 5 : 95 v/v) afford product **59** (56 mg, 79%) as a colorless oil.

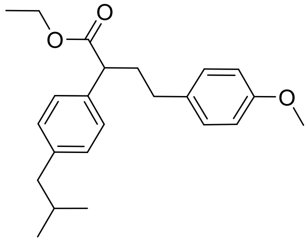

^{1}H NMR (500 MHz, $CDCl_3$): 7.21 (2 H, d, *J* = 7.9 Hz), 7.10 (2 H, d, *J* = 8.1 Hz), 7.08 (2 H, d, *J* = 8.6 Hz), 6.83 (2 H, d, *J* = 8.4 Hz), 4.36–3.96 (2 H, m), 3.79 (3 H, s), 3.52 (1 H, t, *J* = 7.6 Hz), 2.53 (2 H, t, *J* = 7.6 Hz), 2.46 (2 H, d, *J* = 7.1 Hz), 2.37 (1 H, q, *J* = 15.5, 7.8 Hz), 2.05 (1 H, dq, *J* = 15.0, 7.4 Hz), 1.86 (1 H, dquint., *J* = 13.4, 6.7 Hz), 1.22 (3 H, t, *J* = 7.1 Hz), 0.90 (6 H, d, *J* = 6.6 Hz) ppm. – ^{13}C NMR (125 MHz, $CDCl_3$): 174.2, 158.0, 140.7, 136.4, 133.6, 129.5, 129.4, 127.8, 113.9, 60.8, 55.4, 50.7, 45.2, 35.4, 32.8, 30.3, 22.5, 14.3 ppm. – IR: 2952, 2928, 2867, 1731, 1611, 1512, 1464, 1367, 1246, 1175, 1148, 1037, 828 cm^{-1}. – HRMS calcd for $C_{23}H_{31}O_3$: 355.2268, found 355.2270 [M+H$^+$].

Ethyl 4-(4-isobutylphenyl)-2-(4-methoxyphenyl)butanoate (60)

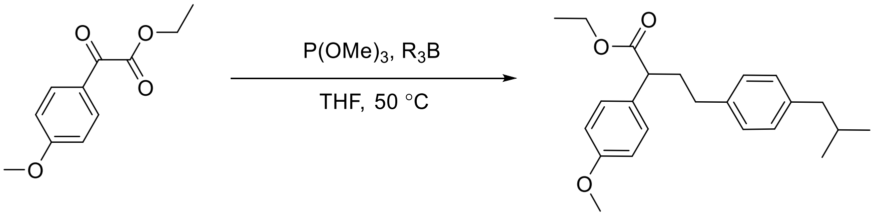

The general procedure GP2 was followed with ethyl 2-(4-methoxyphenyl)-2-oxoacetate (41.6 mg, 0.2 mmol), $P(OMe)_3$ (50 mg, 0.4 mmol, 2.0 equiv.), ethyl 2-(4-isobutylphenyl)-2-oxoacetate (0.4 mL, 1M, 2.0 equiv.) and THF (1.0 mL). The mixture was stirred at 50 °C for 16 h. Purification by flash chromatography on silica gel (EtOAc/hexane, 5 : 95 v/v) afford product **60** (45 mg, 64%) as a colorless oil.

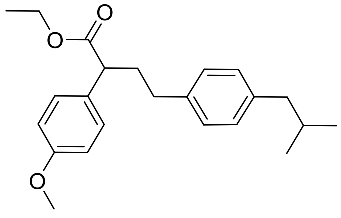

^{1}H NMR (500 MHz, $CDCl_3$): 7.24 (2 H, d, *J* = 8.6 Hz), 7.06 (4 H, s), 6.87 (2 H, d, *J* = 8.5 Hz), 4.20–4.04 (2 H, m), 3.80 (3 H, s), 3.51 (1 H, t, *J* = 7.6 Hz), 2.54 (2 H, t, *J* = 7.7 Hz), 2.45 (2 H, d, *J* = 7.1 Hz), 2.38 (1 H, dq, *J* = 15.6, 7.8 Hz), 2.06 (1 H, dq, *J* = 15.2, 7.6 Hz), 1.85 (1 H, tt, *J* = 13.4, 6.7 Hz), 1.22 (3 H, t, *J* = 7.1 Hz), 0.91 (6 H, d, *J* = 6.7 Hz) ppm. – ^{13}C NMR (125 MHz, $CDCl_3$): 174.3, 158.9, 139.4, 138.7, 131.2, 129.2, 129.1, 128.3, 114.1, 60.8, 55.4, 50.3, 45.2, 35.2, 33.3, 30.4, 22.5, 14.3 ppm. – IR: 2954, 2920, 2866, 1730, 1610, 1510, 1464, 1248, 1176, 1053, 833 cm^{-1}. – HRMS calcd for $C_{23}H_{31}O_3$: 355.2268, found 355.2274 [$M+H^+$].

Ethyl 4-(4-(*tert*-butyl)phenyl)-2-(9,9-dimethyl-9*H*-xanthen-2-yl)butanoate (61)

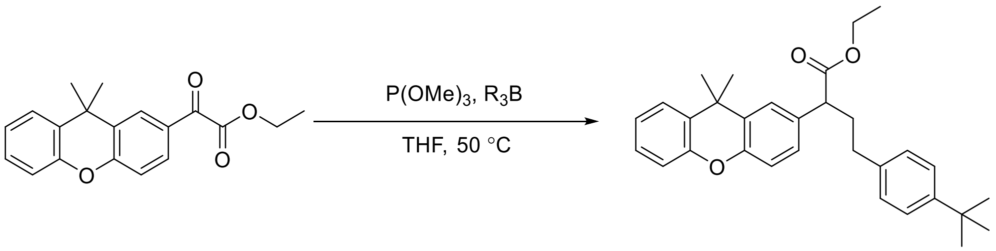

The general procedure GP2 was followed with ethyl 2-(9,9-dimethyl-9*H*-xanthen-2-yl)-2-oxoacetate (62 mg, 0.2 mmol), $P(OMe)_3$ (50 mg, 0.4 mmol, 2.0 equiv.), tris(4-(*tert*-butyl)phenethyl)borane (0.4 mL, 1M, 2.0 equiv.) and THF (1.0 mL). The mixture was stirred at 50 °C for 16 h. Purification by flash chromatography on silica gel (EtOAc/hexane, 5 : 95 v/v) afford product **61** (43 mg, 70%) as a colorless oil.

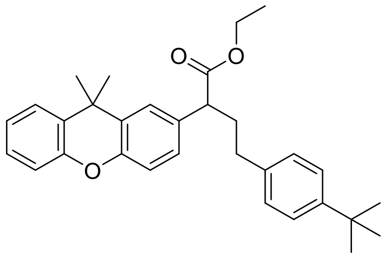

^{1}H NMR (500 MHz, $CDCl_3$): 7.43 (1 H, d, *J* = 7.7 Hz), 7.36–7.30 (3 H, m), 7.24–7.16 (2 H, m), 7.11 (2 H, d, *J* = 8.0 Hz), 7.10–6.96 (3 H, m), 4.39–4.05 (2 H, m), 3.57 (1 H, t, *J* = 7.7 Hz), 2.57 (2 H, t, *J* = 6.7 Hz), 2.43 (1 H, dq, *J* = 16.0, 8.1 Hz), 2.11 (1 H, dq, *J* = 14.9, 7.6 Hz), 1.65 (6 H, s), 1.33 (9 H, s), 1.23 (3 H, t, *J* = 7.1 Hz) ppm. – ^{13}C NMR (125 MHz, $CDCl_3$): 174.1, 150.5, 149.7, 148.9, 138.4, 133.6, 130.2, 130.1, 128.3, 127.5, 127.0, 126.2, 125.9, 125.4, 123.2, 116.6, 116.5, 60.8, 50.8, 35.2, 34.5, 34.2, 33.2, 32.54, 32.49, 31.6, 14.3

ppm. – IR: 2960, 2903, 2866, 1729, 1482, 1450, 1363, 1307, 1254, 1150, 1113, 1036, 909 cm^{-1}. – HRMS calcd for $C_{31}H_{37}O_3$: 457.2737, found 457.2745 [$M+H^+$].

Ethyl 2-(3-fluoro-4-methoxyphenyl)-4-(*p*-tolyl)butanoate (62)

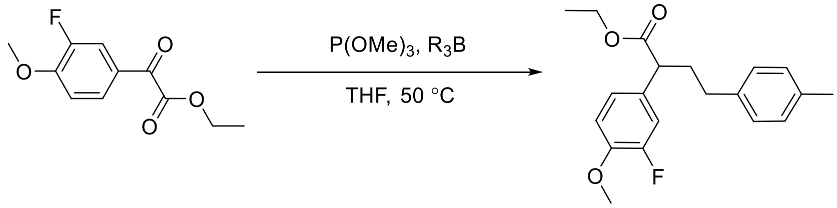

The general procedure GP2 was followed with ethyl ethyl 2-(3-fluoro-4-methoxyphenyl)-2-oxoacetate (46 mg, 0.2 mmol), $P(OMe)_3$ (50 mg, 0.4mmol, 2.0 equiv.), tris(4-methylphenethyl)borane (0.4 mL, 1M, 2.0 equiv.) and THF (1.0 mL). The mixture was stirred at 50 °C for 16 h. Purification by flash chromatography on silica gel (EtOAc/hexane, 5 : 95 v/v) afford product **62** (43 mg, 65%) as a colorless oil.

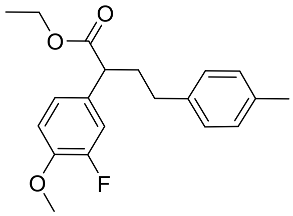

^{1}H NMR (500 MHz, $CDCl_3$): 7.13–6.98 (6 H, m), 6.91 (1 H, t, *J* = 8.6 Hz), 4.20–4.06 (2 H, m), 3.88 (3 H, s), 3.47 (1 H, t, *J* = 7.7 Hz), 2.53 (2 H, t, *J* = 7.8 Hz), 2.35 (1 H, d, *J* = 6.6 Hz), 2.33 (3 H, s), 2.04 (1 H, dq, *J* = 15.1, 7.6 Hz), 1.22 (3 H, t, *J* = 7.1 Hz) ppm. – ^{13}C NMR (125 MHz, $CDCl_3$): 173.8, 152.4 (d, *J* = 246.0 Hz), 146.9 (d, *J* = 10.7 Hz), 138.1, 135.6, 132.0 (d, *J* = 6.2 Hz), 129.2, 128.4, 123.9 (d, *J* = 3.5 Hz), 115.8 (d, *J* = 18.7 Hz), 113.4, 60.9, 56.4, 50.1, 35.1, 33.1, 21.1, 14.3 ppm. – IR: 2933, 2862, 1730, 1517, 1274, 1224, 1180, 1150, 1029, 813 cm^{-1}. – HRMS calcd for $C_{20}H_{24}O_3F$: 331.1704, found 331.1710 [$M+H^+$].

5-(3,4-Dimethoxyphenyl)-2-phenylpentanoic acid (63)

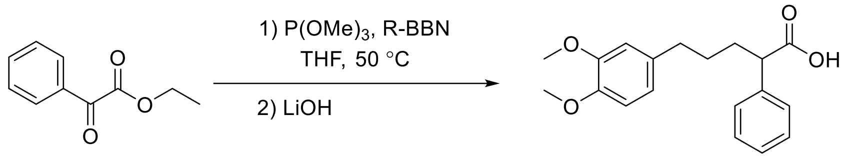

The general procedure GP2 was followed with ethyl 2-oxo-2-phenylacetate (**6**) (36 mg, 0.2 mmol), $P(OMe)_3$ (50 mg, 0.4 mmol, 2.0 equiv.), 9-(3-(3,4-dimethoxyphenyl)propyl)-9-borabicyclo[3.3.1]nonane (1.0 mL, 0.5 M, 2.5 equiv.) and THF (1.0 mL). The mixture was stirred at 50 °C for 48 h. Methanol (2.0 mL), $LiOH{\cdot}H_2O$ (82 mg, 2.0 mmol, 10.0 equiv.) and H_2O (1.0 mL) was added into the reaction mixture. The mixture was stirred at room temperature for 12 h. Purification by flash chromatography on silica gel (EtOAc/hexane, 40 : 60 v/v) afford product **63** (31 mg, 50%) as a colorless oil.

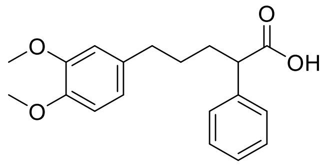

^{1}H NMR (500 MHz, $CDCl_3$): 7.41–7.17 (5 H, m), 6.76 (1 H, d, *J* = 8.1 Hz), 6.66 (1 H, d, *J* = 8.4 Hz), 6.64 (1 H, s), 3.84 (3 H, s), 3.83 (3 H, s), 3.56 (1 H, t, *J* = 7.6 Hz), 2.57 (2 H, hept, *J* = 7.0, 6.4 Hz), 2.18–2.00 (1 H, m), 1.94–1.75 (1 H, m), 1.69–1.45 (2 H, m) ppm. – ^{13}C NMR (125 MHz, $CDCl_3$): 179.9, 148.9, 147.3, 138.4, 134.6, 128.8, 128.2, 127.6, 120.3, 111.7, 111.3, 56.0, 55.9, 51.5, 35.2, 32.6, 29.4 ppm. – IR: 2984, 1740, 1447, 1373, 1240, 1045, 917, 846, 732 cm^{-1}. – HRMS calcd for $C_{19}H_{22}O_4Na$: 337.1410, found 337.1412 [$M+Na^+$].

Ethyl 2-(4-isobutylphenyl)heptanoate (64)

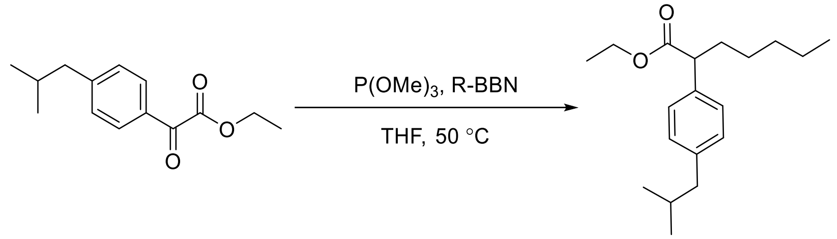

The general procedure GP2 was followed with ethyl 2-(4-isobutylphenyl)-2-oxoacetate (46.8 mg, 0.2 mmol), $P(OMe)_3$ (30 mg, 0.24 mmol, 1.2 equiv.), 9-pentyl-9-

Go back to table of contents

borabicyclo[3.3.1]nonane (1.0 mL, 0.5M, 2.5 equiv.) and THF (1.0 mL). The mixture was stirred at 50 °C for 48 h. Purification by flash chromatography on silica gel (EtOAc/hexane, 3 : 97 v/v) afford product **64** (30 mg, 52%) as a colorless oil.

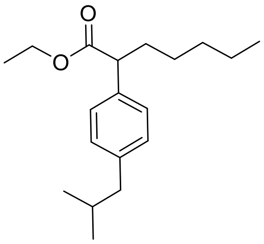

^{1}H NMR (500 MHz, $CDCl_3$): 7.21 (2 H, d, *J* = 8.0 Hz), 7.08 (2 H, d, *J* = 8.1 Hz), 4.35–3.92 (2 H, m), 3.49 (1 H, t, *J* = 7.7 Hz), 2.45 (2 H, d, *J* = 7.2 Hz), 2.06 (1 H, qd, *J* = 14.3, 12.6, 7.5 Hz), 1.85 (1 H, tt, *J* = 13.4, 6.7 Hz), 1.79–1.69 (1 H, m), 1.31–1.28 (6 H, m), 1.21 (3 H, t, *J* = 7.1 Hz), 0.95–0.80 (6 H, m), 0.87–0.82 (3 H, m) ppm. – ^{13}C NMR (125 MHz, $CDCl_3$): 174.5, 140.7, 136.8, 129.3, 127.7, 60.6, 51.6, 45.2, 33.8, 31.7, 30.3, 27.4, 22.6, 22.5, 14.3, 14.1 ppm. – IR: 2984, 1740, 1447, 1373, 1239, 1045, 917, 847, 733 cm^{-1}. – HRMS calcd for $C_{19}H_{31}O_2$: 291.2319, found 291.2316 [M+H$^+$].

Ethyl 2-(3-fluoro-4-methoxyphenyl)octanoate (65)

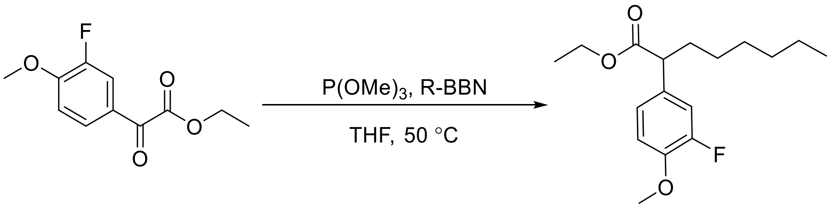

The general procedure GP2 was followed with ethyl 2-(3-fluoro-4-methoxyphenyl)-2-oxoacetate (45.2 mg, 0.2 mmol), $P(OMe)_3$ (30 mg, 0.24 mmol, 1.2 equiv.), 9-hexyl-9-borabicyclo[3.3.1]nonane (1.0 mL, 0.5M, 2.5 equiv.) and THF (1.0 mL). The mixture was stirred at 50 °C for 48 h. Purification by flash chromatography on silica gel (EtOAc/hexane, 10 : 90 v/v) afford product **65** (30 mg, 50%) as a colorless oil.

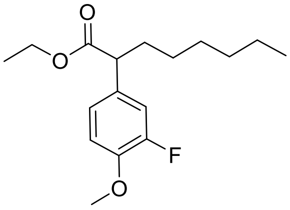

^{1}H NMR (500 MHz, $CDCl_3$): 7.07 (1 H, d, *J* = 14.1 Hz), 7.00 (1 H, d, *J* = 8.4 Hz), 6.89 (1 H, t, *J* = 8.5 Hz), 4.19–4.03 (2 H, m), 3.86 (3 H, s), 3.43 (1 H, t, *J* = 7.7 Hz), 2.00 (1 H, td, *J* = 13.0, 12.2, 8.2 Hz), 1.70 (1 H, dq, *J* = 14.4, 7.9 Hz), 1.34–1.16 (12 H, m), 0.86 (3 H, t, *J* = 6.8 Hz)

ppm. – ^{13}C NMR (125 MHz, $CDCl_3$): 174.1 (s), 152.4 (d, *J* = 245.6 Hz), 146.8 (d, *J* = 10.6 Hz), 132.5 (d, *J* = 6.1 Hz), 123.8 (d, *J* = 3.5 Hz), 115.7 (d, *J* = 18.8 Hz), 113.4, 60.8, 56.4, 51.0, 33.7, 31.7, 29.1, 27.6, 22.7, 14.3, 14.2 ppm. – ^{19}F NMR (470.5 Hz, $CDCl_3$): –134.9 ppm. – IR: 2983, 2944, 2909, 1736, 1446, 1373, 1235, 1097, 1044, 916, 732 cm^{-1}. – HRMS calcd for $C_{17}H_{26}FO_3$: 297.1860, found 297.1863 [M+H$^+$].

Ethyl 2-(9,9-dimethyl-9*H*-xanthen-2-yl)-2-phenylacetate (66)

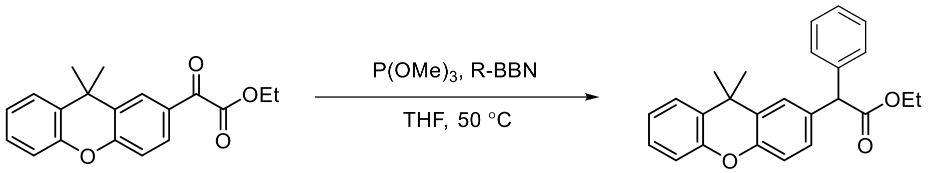

The general procedure GP2 was followed with ethyl 2-(9,9-dimethyl-9*H*-xanthen-2-yl)-2-oxoacetate (62.0 mg, 0.2 mmol), $P(OMe)_3$ (50 mg, 0.4 mmol, 2.0 equiv.), 9-phenyl-9-borabicyclo[3.3.1]nonane (1.0 mL, 0.4M, 2.0 equiv.) and THF (1.0 mL). The mixture was stirred at 50 °C for 16 h. Purification by flash chromatography on silica gel (EtOAc/hexane, 5 : 95 v/v) afford product **66** (50 mg, 67%) as a colorless oil.

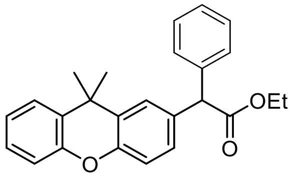

^{1}H NMR (500 MHz, $CDCl_3$): 7.43–7.37 (2 H, m), 7.37–7.30 (4 H, m), 7.31–7.24 (1 H, m), 7.23–7.14 (2 H, m), 7.11–6.96 (3 H, m), 5.01 (1 H, s), 4.23 (2 H, q, *J* = 7.1 Hz), 1.61 (6 H, d, *J* = 2.4 Hz), 1.28 (3 H, t, *J* = 7.1 Hz) ppm. – ^{13}C NMR (125 MHz, $CDCl_3$): 172.8, 150.5, 149.8, 139.1, 133.3, 130.2, 130.0, 128.7, 128.6, 127.9, 127.5, 127.3, 126.5, 126.2, 123.2, 116.6, 116.5, 61.3, 56.8, 34.2, 32.5, 32.5, 14.3 ppm. – IR: 2970, 2931, 2861, 1733, 1601, 1575, 1483, 1449, 1308, 1258, 1182, 1151, 1027, 878 cm^{-1}. – HRMS calcd for $C_{25}H_{25}O_3$: 373.1798, found 373.1802 [M+H$^+$].

Ethyl -2-([1,1'-biphenyl]-4-yl)-2-(3-(trifluoromethoxy)phenyl)acetate (67)

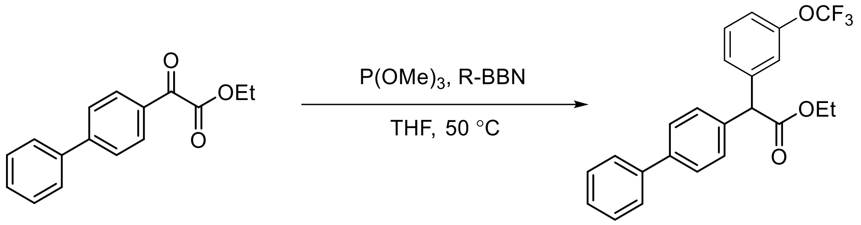

The general procedure GP2 was followed with ethyl 2-([1,1'-biphenyl]-4-yl)-2-oxoacetate (51.0 mg, 0.2 mmol), $P(OMe)_3$ (50 mg, 0.4 mmol, 2.0 equiv.), 9-(3-(trifluoromethoxy)phenyl)-9-borabicyclo[3.3.1]nonane (1.0 mL, 0.4M, 2.0 equiv.) and THF (1.0 mL). The mixture was stirred at 50 °C for 16 h. Purification by flash chromatography on silica gel (EtOAc/hexane, 3 : 97 v/v) afford product **67** (53 mg, 66%) as a colorless oil.

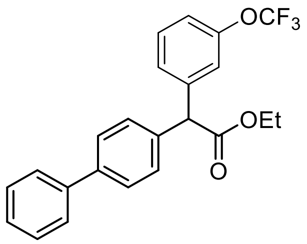

^{1}H NMR (500 MHz, $CDCl_3$): 7.57 (4 H, dd, *J* = 7.8, 2.2 Hz), 7.44 (2 H, t, *J* = 7.6 Hz), 7.40–7.33 (4 H, m), 7.29 (d1 H, , *J* = 7.8 Hz), 7.26 (1 H, d, *J* = 4.8 Hz), 7.15 (1 H, d, *J* = 8.5 Hz), 5.05 (1 H, s, 4.25 (2 H, q, *J* = 7.1 Hz), 1.28 (3 H, t, *J* = 7.1 Hz) ppm. – ^{13}C NMR (125 MHz, $CDCl_3$): 172.0, 149.5, 141.1, 140.7, 137.1, 130.0, 129.0, 128.9, 127.6, 127.6, 127.2, 121.5, 120.59 (q, *J* = 257.3 Hz), 119.8, 61.7, 56.6, 14.3 ppm. – ^{19}F NMR (470.5 Hz, $CDCl_3$): –57.7 (dd, *J* = 11.3, 8.5 Hz) ppm. – IR: 2983, 2944, 2907, 1737, 1713, 1446, 1372, 1242, 1222, 1046, 915, 849 cm^{-1}. – HRMS calcd for $C_{23}H_{20}F_3O_3$: 418.1625, found 418.1625 [M+H$^+$].

Ethyl 2-(2-fluoro-[1,1'-biphenyl]-4-yl)-3-methylbut-3-enoate (68)

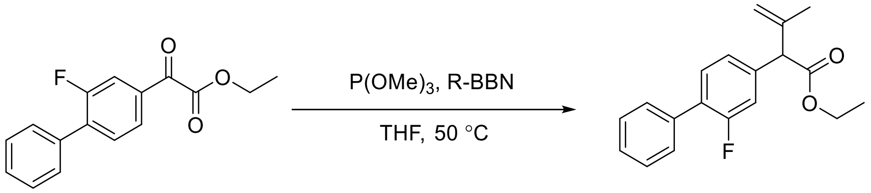

The general procedure GP2 was followed with ethyl 2-(2-fluoro-[1,1'-biphenyl]-4-yl)-2-oxoacetate (54.4 mg, 0.2 mmol), $P(OMe)_3$ (50 mg, 0.4 mmol, 2.0 equiv.), 9-(prop-1-en-2-

yl)-9-borabicyclo[3.3.1]nonane (1.0 mL, 0.4M, 2.0 equiv.) and THF (1.0 mL). The mixture was stirred at 50 °C for 16 h. Purification by flash chromatography on silica gel (EtOAc/hexane, 2 : 98 v/v) afford product **68** (33 mg, 55%) as a colorless oil.

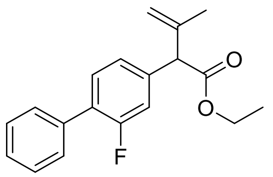

^{1}H NMR (500 MHz, $CDCl_3$): 7.55 (2 H, d, *J* = 7.8 Hz), 7.44 (2 H, t, *J* = 7.6 Hz), 7.40 (1 H, t, *J* = 8.0 Hz), 7.37 (1 H, t, *J* = 7.4 Hz), 7.18 (1 H, d, *J* = 2.3 Hz), 7.16 (1 H, s), 5.05 (1 H, s), 4.94 (1 H, s), 4.33 (1 H, s), 4.29–4.15 (2 H, m), 1.80 (3 H, s), 1.29 (3 H, t, *J* = 7.1 Hz) ppm. – ^{13}C NMR (125 MHz, $CDCl_3$): 171.7, 159.7 (d, *J* = 248.0 Hz), 142.4, 138.5 (d, *J* = 7.9 Hz), 135.7, 130.7 (d, *J* = 3.9 Hz), 129.1 (d, *J* = 3.1 Hz), 128.6, 128.1 (d, *J* = 13.4 Hz), 127.8, 124.9 (d, *J* = 3.5 Hz), 116.6 (d, *J* = 24.0 Hz), 114.5, 61.3, 58.4, 21.8, 14.3 ppm. – ^{19}F NMR (470.5 Hz, $CDCl_3$): –117.9 ppm. – IR: 2983, 2943, 2908, 2254, 1733, 1373, 1240, 1158, 1045, 915, 732 cm^{-1}. – HRMS calcd for $C_{19}H_{19}FO_2$: 299.1442, found 299.1431 [M+H$^+$].

Ethyl 2-(4-methoxyphenyl)-2-(thiophen-3-yl)acetate (69)

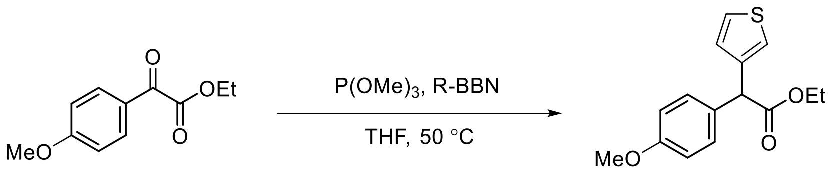

The general procedure GP2 was followed with ethyl 2-(4-methoxyphenyl)-2-oxoacetate (41.6 mg, 0.2 mmol), $P(OMe)_3$ (50 mg, 0.4 mmol, 2.0 equiv.), 3-(9-borabicyclo[3.3.1]nonan-9-yl)thiophene (1.0 mL, 0.4M, 2.0 equiv.) and THF (1.0 mL). The mixture was stirred at 50 °C for 16 h. Purification by flash chromatography on silica gel (EtOAc/hexane, 10 : 90 v/v) afford product **69** (29 mg, 52%) as a colorless oil.

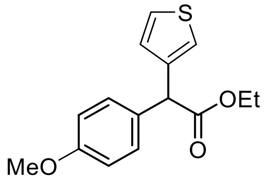

^{1}H NMR (500 MHz, $CDCl_3$): 7.30–7.23 (3 H, m), 7.15 (1 H, d, *J* = 2.6 Hz), 7.01 (1 H, d, *J* = 5.0 Hz), 6.86 (2 H, d, *J* = 8.6 Hz), 4.98 (1 H, s), 4.20 (2 H, qq, *J* = 6.4, 3.7 Hz), 3.79 (3 H, s), 1.26 (3 H, t, *J* = 7.1 Hz) ppm. – ^{13}C NMR (125 MHz, $CDCl_3$): 172.6, 159.0, 139.4, 130.8, 129.6, 128.1, 125.8, 122.6, 114.1, 61.3,

55.4, 52.1, 14.3 ppm. – IR: 3000, 2939, 2252, 1712, 1610, 1511, 1362, 1248, 1222, 1178, 1030, 911, 731 cm^{-1}. – HRMS calcd for $C_{15}H_{17}O_3S$: 277.0893, found 277.0897 [M+H$^+$].

Ethyl 2-(2,3-dihydrobenzofuran-5-yl)-2-(2-fluoro-[1,1'-biphenyl]-4-yl)acetate (70)

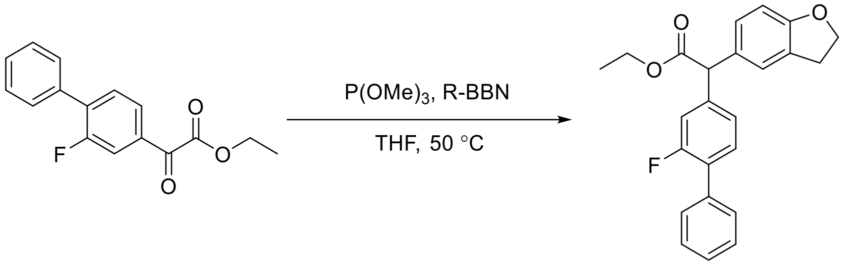

The general procedure GP2 was followed with ethyl 2-(2-fluoro-[1,1'-biphenyl]-4-yl)-2-oxoacetate (54.4 mg, 0.2 mmol), $P(OMe)_3$ (50 mg, 0.4 mmol, 2.0 equiv.), 5-(9-borabicyclo[3.3.1]nonan-9-yl)-2,3-dihydrobenzofuran (1.0 mL, 0.4M, 2.0 equiv.) and THF (1.0 mL). The mixture was stirred at 50 °C for 16 h. Purification by flash chromatography on silica gel (EtOAc/hexane, 10 : 90 v/v) afford product **70** (48 mg, 64%) as a colorless oil.

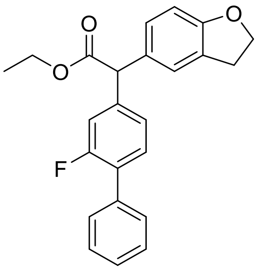

^{1}H NMR (500 MHz, $CDCl_3$): 7.53 (2 H, d, *J* = 7.9 Hz), 7.43 (2 H, t, *J* = 7.6 Hz), 7.42–7.34 (2 H, m), 7.21 (1 H, s), 7.15 (2 H, t, *J* = 9.5 Hz), 7.09 (1 H, d, *J* = 8.2 Hz), 6.77 (1 H, d, *J* = 8.2 Hz), 4.96 (1 H, s), 4.57 (2 H, t, *J* = 8.7 Hz), 4.24 (2 H, qq, *J* = 7.1, 3.7 Hz), 3.21 (2 H, t, *J* = 8.7 Hz), 1.29 (3 H, t, *J* = 7.1 Hz) ppm. – ^{13}C NMR (125 MHz, $CDCl_3$): 172.5, 159.8 (d, *J* = 248.3 Hz), 159.7, 140.8 (d, *J* = 7.7 Hz), 135.6, 130.8 (d, *J* = 3.9 Hz), 130.2, 129.1 (d, *J* = 3.0 Hz), 128.7, 128.4, 127.8, 127.7, 125.2, 124.5 (d, *J* = 3.3 Hz), 116.3 (d, *J* = 24.1 Hz), 109.4, 71.5, 61.5, 56.0, 29.9, 14.3 ppm. – ^{19}F NMR (470.5 Hz, $CDCl_3$): –117.5 ppm. – IR: 2980, 2895, 1731, 1622, 1581, 1490, 1418, 1362, 1241, 1158, 1103, 982, 765 cm^{-1}. – HRMS calcd for $C_{24}H_{22}FO_3$: 377.1547, found 377.1545 [M+H$^+$].

Ethyl 2-(9,9-dimethyl-9*H*-xanthen-2-yl)-2-(1-methyl-1*H*-indol-5-yl)acetate (71)

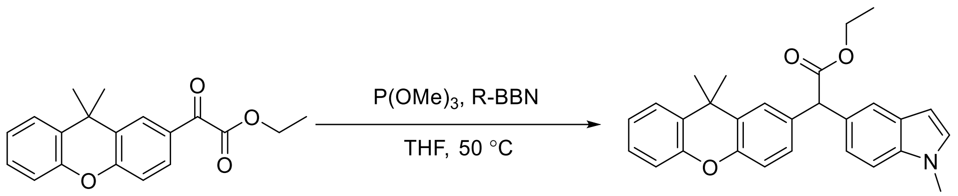

The general procedure GP2 was followed with ethyl 2-(9,9-dimethyl-9*H*-xanthen-2-yl)-2-oxoacetate (62 mg, 0.2 mmol), $P(OMe)_3$ (50 mg, 0.4 mmol, 2.0 equiv.), 5-(9-borabicyclo[3.3.1]nonan-9-yl)-1-methyl-1*H*-indole (1.0 mL, 0.4M, 2.0 equiv.) and THF (1.0 mL). The mixture was stirred at 50 °C for 16 h. Purification by flash chromatography on silica gel (EtOAc/hexane, 10 : 90 v/v) afford product **71** (54 mg, 60%) as a colorless oil.

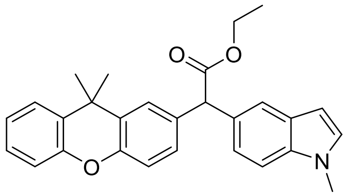

^1H NMR (500 MHz, $CDCl_3$): 7.58 (1 H, s), 7.44 (1 H, d, *J* = 1.7 Hz), 7.41 (1 H, d, *J* = 7.8 Hz), 7.32–7.24 (1 H, m), 7.24–7.16 (3 H, m), 7.08 (1 H, t, *J* = 7.5 Hz), 7.05 (1 H, s), 7.04 (1 H, d, *J* = 4.9 Hz), 7.00 (1 H, d, *J* = 8.4 Hz), 6.46 (1 H, d, *J* = 3.0 Hz), 5.13 (1 H, s), 4.24 (2 H, q, *J* = 7.1 Hz), 3.78 (3 H, s), 1.62 (3 H, s), 1.62 (3 H, s), 1.28 (3 H, t, *J* = 7.1 Hz) ppm. – ^{13}C NMR (125 MHz, $CDCl_3$): 173.5, 150.5, 149.5, 136.0, 134.3, 130.2, 130.1, 129.9, 129.4, 128.7, 127.9, 127.4, 126.5, 126.2, 123.1, 122.4, 120.8, 116.4, 109.5, 101.2, 61.1, 56.9, 34.2, 33.0, 32.5, 32.5, 14.4 ppm. – IR: 2971, 2917, 1730, 1438, 1448, 1421, 1308, 1247, 1154, 1028, 757 cm^{-1}. – HRMS calcd for $C_{28}H_{27}NO_3$: 426.2064, found 426.2057 [$M+H^+$].

Ibuprofen (72)[14]

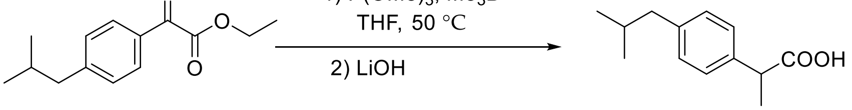

The general procedure GP1 was followed with ethyl 2-(4-isobutylphenyl)-2-oxoacetate (36 mg, 0.2 mmol), $P(OMe)_3$ (30 mg, 0.24 mmol, 1.2 equiv.), Me_3B (2.0 equiv.) and THF (2.0 mL). The mixture was stirred at 50 °C for 16 h. Methanol (2.0 mL), $LiOH{\cdot}H_2O$ (82 mg,

2.0 mmol, 10.0 equiv.) and H_2O (1.0 mL) was added into the reaction mixture. The mixture was stirred at room temperature for 12 h. The reaction mixture was concentrated and 5 mL H_2O was added. The mixture was extracted with with ethyl acetate (3 × 10 mL). The organic layers were discarded, 1M HCl was added into the mixture to adjust the pH to 1. The reaction mixture was extracted with ethyl acetate (3 × 10 mL). The combined organic layers were dried over sodium sulfate, filtered, and to afford product **72** (35 mg, 85%) as a colorless liquid.

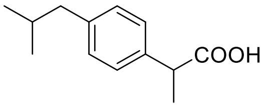

^{1}H NMR (500 MHz, $CDCl_3$): 7.24 (2 H, d, *J* = 8.0 Hz), 7.12 (2 H, d, *J* = 7.9 Hz), 3.72 (1 H, q, *J* = 7.2 Hz), 2.46 (2 H, d, *J* = 7.2 Hz), 1.86 (1 H, dquint., *J* = 13.5, 6.7 Hz), 1.51 (3 H, *J* = 7.2 Hz), 0.91 (6 H, d, *J* = 6.6 Hz) ppm. – ^{13}C NMR (125 MHz, $CDCl_3$): 181.3, 141.0, 137.1, 129.5, 127.4, 45.2, 45.1, 30.3, 22.5, 18.2 ppm. – IR: 2953, 2868, 1706, 1512, 1463, 1411, 1230, 1070, 938, 863 cm^{-1}.

Naproxen (73)[14]

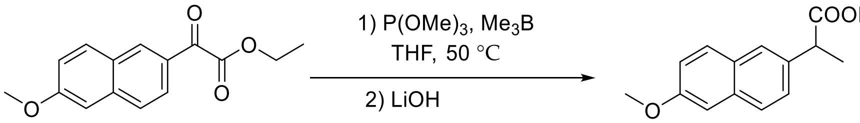

The general procedure GP1 was followed with ethyl 2-(6-methoxynaphthalen-2-yl)-2-oxoacetate (51.6 mg, 0.2 mmol), $P(OMe)_3$ (30 mg, 0.24 mmol, 1.2 equiv.), Et_3B (0.3 mL, 1M, 1.5 equiv.) and THF (1.7 mL). The mixture was stirred at 50 °C for 16 h. Methanol (2.0 mL), $LiOH{\cdot}H_2O$ (82 mg, 2.0 mmol, 10.0 equiv.) and H_2O (1.0 mL) was added into the reaction mixture. The mixture was stirred at room temperature for 12 h. 1M HCl was added into the mixture to adjust the pH to 1. The reaction mixture was extracted with ethyl acetate (3 × 10 mL). The combined organic layers were dried over sodium sulfate. Purification by flash chromatography on silica gel (EtOAc/hexane, 30 : 70 v/v) afford product **73** (28 mg, 60%) as a colorless solid.

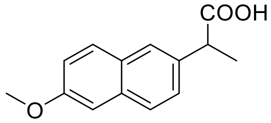

m.p.: 118–119 °C. – ^{1}H NMR (500 MHz, $CDCl_3$): 11.92 (1 H, brs), 7.76–7.61 (3 H, m), 7.42 (1 H, dd, *J* = 8.5, 1.8 Hz), 7.15 (1 H, dd, *J* = 8.9, 2.5 Hz), 7.11 (1 H, d, *J* = 2.4 Hz), 3.92 (3 H, s), 3.88 (1 H, q, *J* = 7.2 Hz), 1.60 (3 H, d, *J* = 7.2 Hz) ppm. – ^{13}C NMR (125 MHz, $CDCl_3$): 181.1, 157.8, 135.0, 133.9, 129.4, 129.0, 127.4, 126.3, 126.3, 119.2, 105.7, 55.4, 45.4, 18.2 ppm. – IR: 2983, 1738, 1446, 1373, 1239, 1045, 918, 849, 736 cm^{-1}.

Flurbiprofen (74)[14]

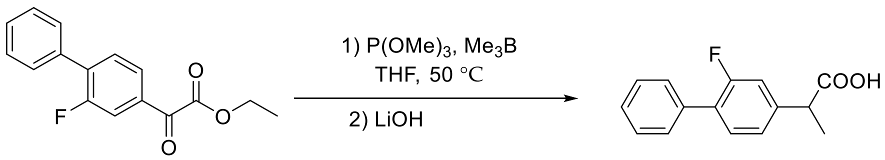

The general procedure GP1 was followed with ethyl 2-(2-fluoro-[1,1'-biphenyl]-4-yl)-2-oxoacetate (54.4 mg, 0.2 mmol), $P(OMe)_3$ (30 mg, 0.24 mmol, 1.2 equiv.), Et_3B (0.3 mL, 1M, 1.5 equiv.) and THF (1.7 mL). The mixture was stirred at 50 °C for 16 h. Methanol (2.0 mL), $LiOH{\cdot}H_2O$ (82 mg, 2.0 mmol, 10.0 equiv.) and H_2O (1.0 mL) was added into the reaction mixture. The mixture was stirred at room temperature for 12 h. 1M HCl was added into the mixture to adjust the pH to 1. The reaction mixture was extracted with ethyl acetate (3 × 10 mL). The combined organic layers were dried over sodium sulfate. Purification by flash chromatography on silica gel (EtOAc/hexane, 30 : 70 v/v) afford product **74** (37 mg, 76%) as a colorless oil.

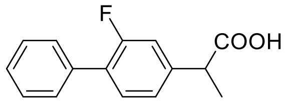

^{1}H NMR (500 MHz, $CDCl_3$): 7.53 (2 H, d, *J* = 7.8 Hz), 7.47–7.33 (4 H, m), 7.22–7.10 (2 H, m), 3.79 (1 H, q, *J* = 7.1 Hz), 1.57 (3 H, d, *J* = 7.2 Hz) ppm. – ^{13}C NMR (125 MHz, $CDCl_3$): 179.9, 159.8 (d, *J* = 248.5 Hz), 141.1 (d, *J* = 7.8 Hz), 135.5, 131.0 (d, *J* = 4.1 Hz), 129.1 (d, *J* = 2.9 Hz), 128.6, 128.3 (d, *J* = 13.6 Hz), 127.9, 123.8 (d, *J* = 3.4 Hz), 115.5 (d, *J* = 23.7 Hz), 44.9, 18.2 ppm. – ^{19}F NMR (470.5 Hz, $CDCl_3$): –117.4 (dd, *J* = 11.3, 8.5 Hz) ppm. – IR: 2984, 1740, 1447, 1373, 1240, 1045, 917, 846, 733 cm^{-1}.

Ethyl 2-phenylbutanoate-2-*d* (78)

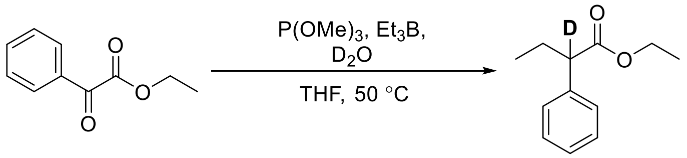

The general procedure GP3 was followed with ethyl 2-oxo-2-phenylacetate (**6**) (36 mg, 0.2 mmol), $P(OMe)_3$ (30 mg, 0.24 mmol, 1.2 equiv.), Et_3B (0.4 mL, 1M, 2.0 equiv.), D_2O (20 mg, 1.0 mmol, 5.0 equiv.) and THF (1.6 mL). The mixture was stirred at 50 °C for 36 h. Purification by flash chromatography on silica gel (EtOAc/hexane, 3 : 97 v/v) afford product **78** (28 mg, 72%, 92% D) as a colorless oil.

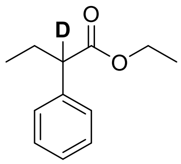

^{1}H NMR (500 MHz, $CDCl_3$): 7.38–7.16 (5 H, m), 4.23–3.94 (2 H, m), 2.09 (1 H, dq, *J* = 14.6, 7.4 Hz), 1.79 (1 H, dq, *J* = 14.5, 7.3 Hz), 1.21 (3 H, t, *J* = 7.1 Hz), 0.90 (3 H, t, *J* = 7.4 Hz) ppm. – ^{13}C NMR (125 MHz, $CDCl_3$): 174.2, 139.4, 128.6, 128.1, 127.2, 60.7, 53.4 (d, *J* = 19.9 Hz), 26.9, 14.3, 12.3 ppm. – ^{2}D NMR (77 MHz, $CDCl_3$): 3.41 (1 D, s) ppm. – IR: 2983, 1738, 1447, 1373, 1239, 1045, 917, 847, 733 cm^{-1}. – HRMS calcd for $C_{12}H_{16}DO_2$: 194.1286, found 194.1290 [$M+H^+$].

Ethyl 2-ethyl-3-hydroxy-3-methyl-2-phenylbutanoate (79)

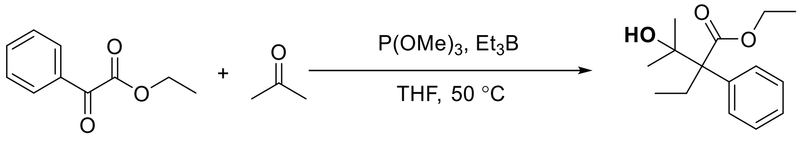

The general procedure GP3 was followed with ethyl 2-oxo-2-phenylacetate (**6**) (36 mg, 0.2 mmol), $P(OMe)_3$ (50 mg, 0.4mmol, 2.0 equiv.), Et_3B (0.4 mL, 1M, 2.0 equiv.), acetone (116 mg, 2.0 mmol, 10.0 equiv.) and THF (1.6 mL). The mixture was stirred at 50 °C for 16 h. Purification by flash chromatography on silica gel (EtOAc/hexane, 10 : 90 v/v) afford product **79** (43 mg, 85%) as a colorless oil.

Gram scale synthesis: The general procedure GP3 two identical reactions were run with ethyl 2-oxo-2-phenylacetate (**6**) (890 mg, 5.0 mmol), $P(OMe)_3$ (744 mg, 6.0 mmol, 1.2 equiv.), Et_3B (7.5 mL, 1M, 2.0 equiv.), acetone (2.0 g, 35.0 mmol, 7.0 equiv.) and THF (40 mL). The mixture was stirred at 50 °C for 16 h. Purification by flash chromatography on silica gel (EtOAc/hexane, 10 : 90 v/v) afford product **79** (2.2 g, 88%) as a colorless oil.

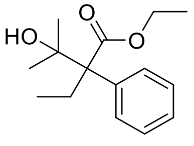

^{1}H NMR (500 MHz, $CDCl_3$): 7.33–7.14 (5 H, m), 4.31–4.18 (2 H, m, *J* = 7.1 Hz), 4.14 (1 H, s), 2.44 (1 H, dq, *J* = 14.8, 7.4 Hz), 2.29 (1 H, dq, *J* = 14.8, 7.5 Hz), 1.25 (3 H, t, *J* = 7.1 Hz), 1.19 (3 H, s), 1.04 (3 H, s), 0.94 (3 H, t, *J* = 7.4 Hz) ppm. – ^{13}C NMR (125 MHz, $CDCl_3$): 177.1, 139.1, 129.8, 127.3, 126.8, 75.2, 63.4, 61.0, 28.0, 25.50, 25.46, 14.2, 10.6 ppm. – IR: 2980, 1722, 1446, 1368, 1229, 1171, 1108, 1024, 940 cm^{-1}. – HRMS calcd for $C_{15}H_{22}O_3Na$: 273.1461, found 273.1463 [M+Na$^+$].

Ethyl 2-ethyl-3-hydroxy-3-(methyl-*d*3)-2-phenylbutanoate-4,4,4-*d*3 (79-D)

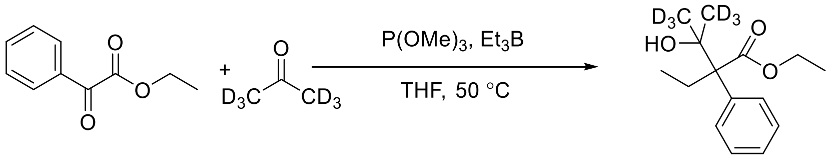

The general procedure GP3 was followed with ethyl 2-oxo-2-phenylacetate (**6**) (36 mg, 0.2 mmol), $P(OMe)_3$ (50 mg, 0.4 mmol, 2.0 equiv.), Et_3B (0.4 mL, 1M, 2.0 equiv.), propan-2-one-*d6* (64 mg, 1.0 mmol, 5.0 equiv.) and THF (1.6 mL). Purification by flash chromatography on silica gel (EtOAc/hexane, 5 : 95 v/v) afford product **79-D** (41 mg, 80%) as a colorless oil.

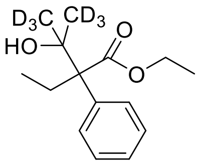

^{1}H NMR (500 MHz, $CDCl_3$): 7.35–7.14 (5 H, m), 4.37–4.16 (2 H, m), 4.10 (1 H, brs), 2.43 (1 H, dq, *J* = 14.8, 7.4 Hz), 2.27 (1 H, dq, *J* = 14.8, 7.5 Hz), 1.24 (3 H, t, *J* = 7.2 Hz), 0.93 (3 H, t, *J* = 7.5 Hz) ppm. – ^{13}C NMR (125 MHz, $CDCl_3$): 177.1, 139.1, 129.8, 127.3, 126.7, 74.9, 63.3, 60.9, 27.1 (pent, *J* = 20.8 Hz), 25.5, 24.5 (pent, *J* = 19.7 Hz), 14.2, 10.6 ppm. – ^{2}D NMR (77 MHz, $CDCl_3$): 1.21 (3 D, s), 1.06 (3D, s)

ppm. – IR: 2977, 2938, 1698, 1465, 1446, 1367, 1228, 1142, 1094, 1024 cm^{-1}. – HRMS calcd for $C_{15}H_{17}D_6O_3$: 257.2018, found 257.2016 [$M+H^+$].

Ethyl 2-(1-hydroxycyclopentyl)-2-phenylbutanoate (80)

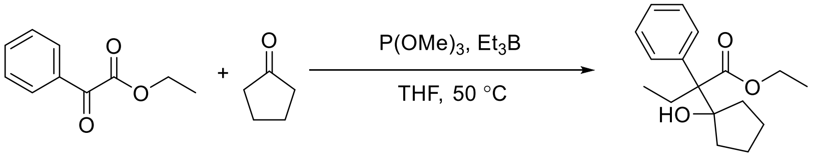

The general procedure GP3 was followed with ethyl 2-oxo-2-phenylacetate (6) (36 mg, 0.2 mmol), $P(OMe)_3$ (50 mg, 0.4mmol, 2.0 equiv.), Et_3B (0.4 mL, 1M, 2.0 equiv.), cyclopentanone (50.4 mg, 0.6 mmol, 3.0 equiv.) and THF (1.6 mL). Purification by flash chromatography on silica gel (EtOAc/hexane, 5 : 95 v/v) afford product **80** (44 mg, 79%) as a colorless oil.

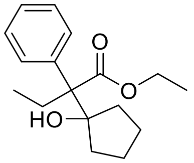

^{1}H NMR (500 MHz, $CDCl_3$): 7.33–7.18 (5 H, m), 4.23 (2 H, q, *J* = 7.1 Hz), 3.27 (1 H, s), 2.43 (1 H, dq, *J* = 14.7, 7.3 Hz), 2.24 (1 H, dq, *J* = 14.8, 7.5 Hz), 1.79–1.53 (6 H, m), 1.41–1.29 (2 H, m), 1.24 (3 H, t, *J* = 7.1 Hz), 0.99 (3 H, t, *J* = 7.4 Hz) ppm. – ^{13}C NMR (125 MHz, $CDCl_3$): 176.8, 139.8, 129.7, 127.4, 126.7, 86.6, 62.5, 60.9, 37.1, 35.4, 26.9, 23.5, 23.0, 14.2, 10.9 ppm. – IR: 2980, 1738, 1446, 1372, 1238, 1045, 917, 734 cm^{-1}. – HRMS calcd for $C_{17}H_{24}O_3Na$: 299.1618, found 299.1619 [$M+Na^+$].

Ethyl 2-(1-hydroxycyclohexyl)-2-phenylbutanoate (81)

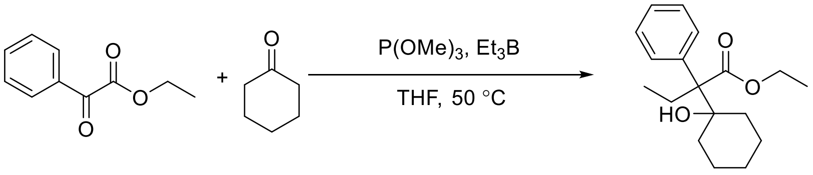

The general procedure GP3 was followed with ethyl 2-oxo-2-phenylacetate (**6**) (36 mg, 0.2 mmol), $P(OMe)_3$ (50 mg, 0.4mmol, 2.0 equiv.), Et_3B (0.4 mL, 1M, 2.0 equiv.), cyclohexanone (60 mg, 0.6 mmol, 3.0 equiv.) and THF (1.6 mL). Purification by flash

chromatography on silica gel (EtOAc/hexane, 5 : 95 v/v) afford product **81** (41 mg, 71%) as a colorless oil.

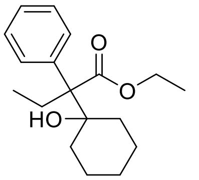

^{1}H NMR (500 MHz, $CDCl_3$): 7.33–7.14 (5 H, m), 4.47–4.02 (2 H, m), 3.78 (1 H, brs), 2.45 (1 H, dq, *J* = 14.8, 7.4 Hz), 2.30 (1 H, dq, *J* = 14.8, 7.4 Hz), 1.80–1.54 (4 H, m), 1.50 (1 H, d, *J* = 13.0 Hz), 1.41–1.28 (3 H, m), 1.24 (3 H, t, *J* = 7.1 Hz), 0.96–0.90 (1 H, m), 0.90 (3 H, t, *J* = 7.4 Hz), 0.81 (1 H, qt, *J* = 13.1, 3.8 Hz) ppm. – ^{13}C NMR (125 MHz, $CDCl_3$): 177.1, 138.6, 130.3, 127.1, 126.7, 75.9, 64.4, 60.9, 33.9, 31.9, 25.8, 24.7, 21.9, 21.7, 14.3, 10.6 ppm. – IR: 2982, 2936, 1739, 1446, 1373, 1240, 1045, 916, 847, 732 cm^{-1}. – HRMS calcd for $C_{18}H_{27}O_3$: 313.1774, found 313.1775 [M+H$^+$].

tert-Butyl 4-(1-ethoxy-1-oxo-2-phenylbutan-2-yl)-4-hydroxypiperidine-1-carboxylate (82)

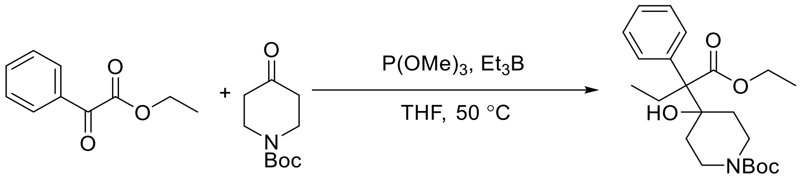

The general procedure GP3 was followed with ethyl 2-oxo-2-phenylacetate (**6**) (36 mg, 0.2 mmol), $P(OMe)_3$ (50 mg, 0.4mmol, 2.0 equiv.), Et_3B (0.4 mL, 1M, 2.0 equiv.), *tert*-butyl 4-oxopiperidine-1-carboxylate (80 mg, 0.6 mmol, 3.0 equiv.) and THF (1.6 mL). Purification by flash chromatography on silica gel (EtOAc/hexane, 5 : 95 v/v) afford product **82** (49 mg, 63%) as a colorless oil.

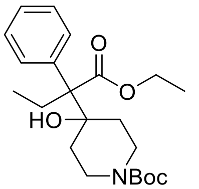

^{1}H NMR (500 MHz, $CDCl_3$): 7.29 (2 H, t, *J* = 7.3 Hz), 7.25 (1 H, d, *J* = 6.9 Hz), 7.19 (2 H, d, *J* = 7.3 Hz), 4.33–4.14 (2 H, m), 4.01 (1 H, s), 3.90–3.65 (2 H, m), 3.05 (2 H, brs), 2.43 (1 H, dq, *J* = 14.6, 7.3 Hz), 2.28 (1 H, dq, *J* = 14.7, 7.4 Hz), 1.79–1.42 (4 H, m), 1.36 (9 H, s), 1.25 (3 H, t, *J* = 7.1 Hz), 0.96 (3 H, t, *J* = 7.4 Hz) ppm. – ^{13}C NMR (125 MHz, $CDCl_3$): 177.0, 154.9, 129.9, 127.5, 127.0, 79.3, 74.4, 63.5, 61.2, 39.9, 39.2,

33.5, 31.6, 28.5, 24.9, 14.2, 10.6 ppm. – IR: 2984, 1739, 1446, 1373, 1240, 1045, 917, 846, 733 cm^{-1}. – HRMS calcd for $C_{22}H_{34}NO_5$: 392.2431, found 392.2430 [M+H$^+$].

Ethyl 2-(1-hydroxycyclobutyl)-2-phenylbutanoate (83)

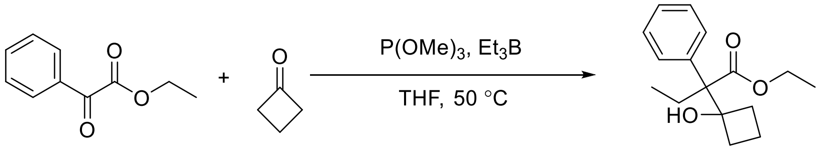

The general procedure GP3 was followed with ethyl 2-oxo-2-phenylacetate (**6**) (36 mg, 0.2 mmol), $P(OMe)_3$ (50 mg, 0.4mmol, 2.0 equiv.), Et_3B (0.4 mL, 1M, 2.0 equiv.), cyclobutanone (28 mg, 0.4 mmol, 2.0 equiv.) and THF (1.6 mL). Purification by flash chromatography on silica gel (EtOAc/hexane, 5 : 95 v/v) afford product **83** (36 mg, 68%) as a colorless oil.

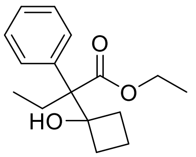

^{1}H NMR (500 MHz, $CDCl_3$): 7.34 (2 H, t, J = 7.4 Hz), 7.31–7.21 (3 H, m), 4.28 (2 H, q, J = 7.0 Hz), 3.28 (1 H, s), 2.43 (1 H, dd, J = 13.6, 6.3 Hz), 2.31 (2 H, dq, J = 14.6, 7.4 Hz), 2.11 (1 H, dq, J = 14.7, 7.4 Hz), 1.95–1.83 (3 H, m), 1.29 (3 H, t, J = 7.2 Hz), 1.21–1.09 (1 H, m), 1.00 (3 H, t, J = 7.5 Hz) ppm. – ^{13}C NMR (125 MHz, $CDCl_3$): 175.8, 139.2, 128.9, 127.7, 126.8, 80.8, 62.0, 60.9, 33.2, 31.9, 25.8, 14.2, 14.0, 10.5 ppm. – IR: 3522, 2983, 2941, 1705, 1604, 1446, 1228, 1106, 1026, 916 cm^{-1}. – HRMS calcd for $C_{16}H_{23}O_3$: 263.1642, found 263.1647 [M+H$^+$].

Ethyl 2-(3-hydroxyoxetan-3-yl)-2-phenylbutanoate (84)

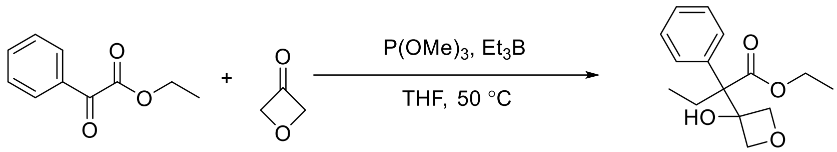

The general procedure GP3 was followed with ethyl 2-oxo-2-phenylacetate (**6**) (36 mg, 0.2 mmol), $P(OMe)_3$ (50 mg, 0.4mmol, 2.0 equiv.), Et_3B (0.4 mL, 1M, 2.0 equiv.), oxetan-3-one

(28.8 mg, 0.4 mmol, 2.0 equiv.) and THF (1.6 mL). Purification by flash chromatography on silica gel (EtOAc/hexane, 5 : 95 v/v) afford product **84** (39 mg, 73%) as a colorless oil.

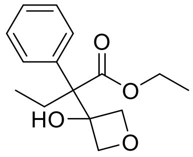

^{1}H NMR (500 MHz, $CDCl_3$): 7.35 (2 H, t, *J* = 7.5 Hz), 7.29 (1 H, t, *J* = 7.2 Hz), 7.22 (2 H, d, *J* = 7.6 Hz), 4.89 (1 H, d, *J* = 7.2 Hz), 4.82 (1 H, d, *J* = 7.3 Hz), 4.51 (1 H, d, *J* = 7.2 Hz), 4.37 (1 H, d, *J* = 7.3 Hz), 4.27 (2 H, q, *J* = 8.5, 7.1 Hz), 3.37 (1 H, s), 2.35 (1 H, dq, *J* = 14.7, 7.3 Hz), 2.25 (1 H, dq, *J* = 14.7, 7.4 Hz), 1.28 (3 H, t, *J* = 7.2 Hz), 1.01 (3 H, t, *J* = 7.4 Hz) ppm. – ^{13}C NMR (125 MHz, $CDCl_3$): 174.8, 137.6, 128.4, 128.2, 127.5, 80.8, 80.8, 79.0, 61.4, 60.5, 26.2, 14.2, 10.2 ppm. – IR: 3364, 2975, 2941, 2881, 1723, 1446, 1367 1309, 1232, 1174, 1108, 1023, 969 cm^{-1}. – HRMS calcd for $C_{15}H_{21}O_4$: 265.1434, found 265.1440 [M+H$^+$].

***tert*-Butyl 3-(1-ethoxy-1-oxo-2-phenylbutan-2-yl)-3-hydroxyazetidine-1-carboxylate (85)**

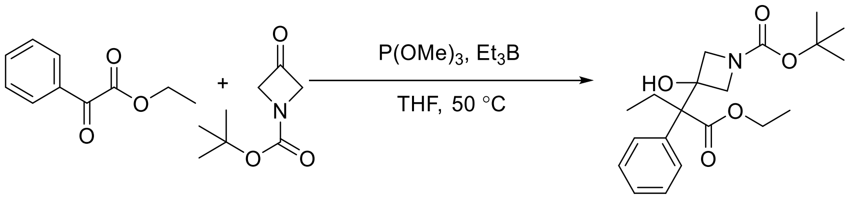

The general procedure GP3 was followed with ethyl 2-oxo-2-phenylacetate (**6**) (36 mg, 0.2 mmol), $P(OMe)_3$ (50 mg, 0.4mmol, 2.0 equiv.), Et_3B (0.4 mL, 1M, 2.0 equiv.), *tert*-butyl 3-oxoazetidine-1-carboxylate (68.4 mg, 0.4 mmol, 2.0 equiv.) and THF (1.6 mL). Purification by flash chromatography on silica gel (EtOAc/hexane, 5 : 95 v/v) afford product 8**5** (48 mg, 66%) as a colorless oil.

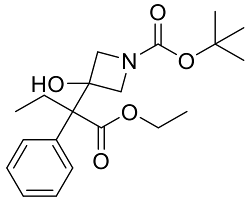

^{1}H NMR (500 MHz, $CDCl_3$): 7.35 (2 H, t, *J* = 7.5 Hz), 7.29 (1 H, t, *J* = 7.3 Hz), 7.21 (2 H, d, *J* = 7.5 Hz), 4.28 (2 H, qt, *J* = 7.1, 3.4 Hz), 4.16 (2 H, d, *J* = 9.0 Hz), 3.72 (1 H, d, *J* = 9.6 Hz), 3.65 (1 H, d, *J* = 9.7 Hz), 3.28 (1 H, s), 2.28 (1 H, dq, *J* = 14.5, 7.2 Hz), 2.15 (1 H, dq, *J* = 14.7, 7.4 Hz), 1.35 (9 H, s), 1.28 (3 H, t, *J* = 7.2 Hz), 1.01 (3 H, t, *J* = 7.4 Hz) ppm. – ^{13}C NMR (125 MHz, $CDCl_3$):

175.1, 156.2, 137.7, 128.5, 128.3, 127.6, 79.5, 75.1, 61.5, 60.9, 60.4, 59.1, 28.5, 26.1, 14.2, 10.4 ppm. – IR: 3391, 2976, 2944, 1727, 1702, 1678, 1420, 1366, 1231, 1164, 1027 cm^{-1}. – HRMS calcd for $C_{20}H_{30}NO_5$: 364.2118, found 364.2123 [M+H$^+$].

Ethyl 2-ethyl-3-oxo-2-phenylbutanoate (86)

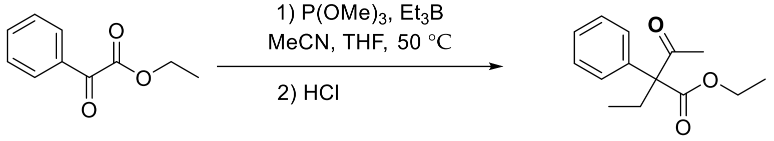

The general procedure GP3 was followed with ethyl 2-oxo-2-phenylacetate (**6**) (36 mg, 0.2 mmol), $P(OMe)_3$ (50 mg, 0.4mmol, 2.0 equiv.), Et_3B (0.4 mL, 1M, 2.0 equiv.), acetonitrile (82 mg, 2.0 mmol, 10.0 equiv.) and THF (1.6 mL). Purification by flash chromatography on silica gel (EtOAc/hexane, 3 : 97 v/v) afford product **86** (30 mg, 65%) as a colorless oil.

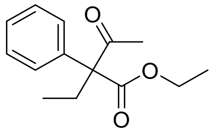

^{1}H NMR (500 MHz, $CDCl_3$): 7.40–7.27 (5 H, m), 4.29 (2 H, q, *J* = 7.1 Hz), 2.38 (1 H, dq, *J* = 14.6, 7.3 Hz), 2.17 (1 H, dq, *J* = 14.7, 7.5 Hz), 2.07 (3 H, s), 1.30 (3 H, t, *J* = 7.1 Hz), 0.85 (3 H, t, *J* = 7.4 Hz) ppm. – ^{13}C NMR (125 MHz, $CDCl_3$): 203.7, 171.1, 137.2, 128.7, 128.1, 127.7, 69.2, 61.4, 28.2, 27.8, 14.2, 9.5 ppm. – IR: 2981, 1712, 1446, 1355, 1300, 1225, 1125, 1027, 911, 746 cm^{-1}. – HRMS calcd for $C_{14}H_{19}O_3$: 235.1329, found 235.1332 [M+H$^+$].

Ethyl 2-ethyl-3-oxo-2-phenylpentanoate (87)

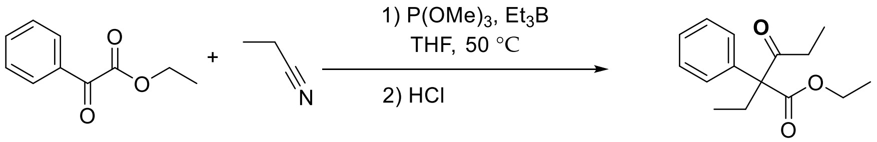

The general procedure GP3 was followed with ethyl 2-oxo-2-phenylacetate (**6**) (36 mg, 0.2 mmol), $P(OMe)_3$ (50 mg, 0.4mmol, 2.0 equiv.), Et_3B (0.4 mL, 1M, 2.0 equiv.), propiononitrile (110 mg, 2.0 mmol, 10.0 equiv.) and THF (1.6 mL). Purification by flash

chromatography on silica gel (EtOAc/hexane, 3 : 97 v/v) afford product **87** (31 mg, 63%) as a colorless oil.

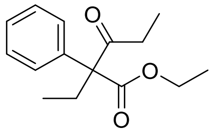

^{1}H NMR (500 MHz, $CDCl_3$): 7.40–7.25 (5 H, m), 4.28 (2 H, q, *J* = 7.2 Hz), 2.50–2.30 (2 H, m), 2.30–2.10 (2 H, m), 1.29 (3 H, t, *J* = 7.1 Hz), 0.96 (3 H, t, *J* = 7.3 Hz), 0.84 (3 H, t, *J* = 7.4 Hz) ppm. – ^{13}C NMR (125 MHz, $CDCl_3$): 206.9, 171.3, 137.3, 128.6, 128.2, 127.6, 69.0, 61.4, 33.4, 28.4, 14.2, 9.5, 9.0 ppm. – IR: 2978, 2936, 1715, 1496, 1446, 1365, 1300, 1225, 1122, 1022 cm^{-1}. – HRMS calcd for $C_{15}H_{21}O_3$: 249.1485, found 249.1486 [M+H^{+}].

Ethyl 2-ethyl-3-oxo-2,4-diphenylbutanoate (88)

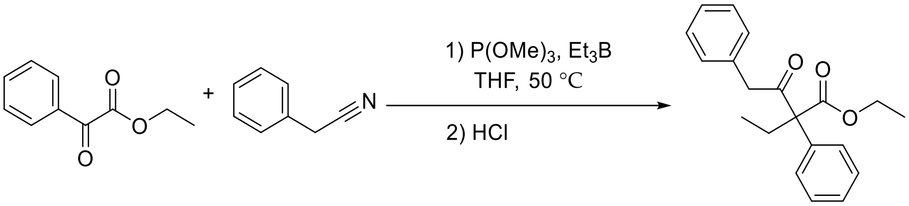

The general procedure GP3 was followed with ethyl 2-oxo-2-phenylacetate (**6**) (36 mg, 0.2 mmol), $P(OMe)_3$ (50 mg, 0.4mmol, 2.0 equiv.), Et_3B (0.4 mL, 1M, 2.0 equiv.), 2-phenylacetonitrile (234 mg, 2.0 mmol, 10.0 equiv.) and THF (1.6 mL). Purification by flash chromatography on silica gel (EtOAc/hexane, 3 : 97 v/v) afford product **88** (42 mg, 68%) as a colorless oil.

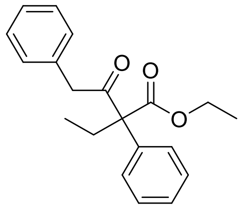

^{1}H NMR (500 MHz, $CDCl_3$): 7.44–7.30 (5 H, m), 7.20 (3 H, ddd, *J* = 11.0, 7.8, 5.9 Hz), 6.93 (2 H, d, *J* = 6.6 Hz), 4.30 (2 H, qq, *J* = 10.8, 7.1 Hz), 3.75 (1 H, d, *J* = 16.1 Hz), 3.57 (1 H, d, *J* = 16.1 Hz), 2.43 (1 H, dq, *J* = 14.6, 7.3 Hz), 2.18 (1 H, dq, *J* = 14.8, 7.5 Hz), 1.30 (3 H, t, *J* = 7.1 Hz), 0.86 (3 H, t, *J* = 7.4 Hz) ppm. – ^{13}C NMR (125 MHz, $CDCl_3$): 203.1, 171.1, 136.8, 134.9, 129.5, 128.8, 128.4, 128.3, 127.9, 126.7, 69.4, 61.6, 46.4, 28.6, 14.2, 9.6 ppm. – IR: 2970, 1717, 1609, 1511, 1496,

1454, 1367, 1229, 1124, 1022 cm^{-1}. – HRMS calcd for $C_{20}H_{23}O_3$: 311.1642, found 311.1645 [$M+H^+$].

Ethyl 4-(2-chlorophenyl)-2-ethyl-2-(3-fluoro-4-methoxyphenyl)-3-oxobutanoate (89)

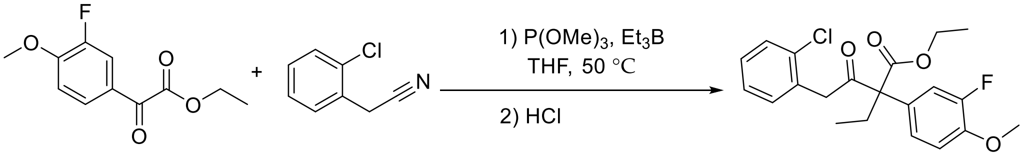

The general procedure GP3 was followed with ethyl 2-(3-fluoro-4-methoxyphenyl)-2-oxoacetate (45.2 mg, 0.2 mmol), $P(OMe)_3$ (50 mg, 0.4 mmol, 2.0 equiv.), Et_3B (0.4 mL, 1M, 2.0 equiv.), 2-(2-chlorophenyl)acetonitrile (303 mg, 2.0 mmol, 10.0 equiv.) and THF (1.6 mL). The mixture was stirred at 50 °C for 16 h. Purification by flash chromatography on silica gel (EtOAc/hexane, 3 : 97 v/v) afford product **89** (46 mg, 59%) as a colorless oil.

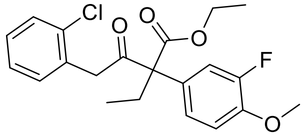

^{1}H NMR (500 MHz, $CDCl_3$): 7.37–7.25 (2 H, m), 7.21–7.11 (3 H, m), 7.05–7.00 (1 H, m), 6.96 (1 H, t, *J* = 8.7 Hz), 4.41–4.24 (2 H, m), 3.96 (1 H, d, *J* = 17.5 Hz), 3.91 (3 H, s), 3.78 (1 H, d, *J* = 17.5 Hz), 2.41 (1 H, dq, *J* = 14.6, 7.3 Hz), 2.21 (1 H, dq, *J* = 14.7, 7.4 Hz), 1.32 (3 H, t, *J* = 7.1 Hz), 0.86 (3 H, t, *J* = 7.4 Hz) ppm. – ^{13}C NMR (125 MHz, $CDCl_3$): 201.3, 170.8, 152.3 (d, *J* = 246.0 Hz), 147.2 (d, *J* = 10.6 Hz), 134.7, 132.7, 131.5, 129.5 (d, *J* = 6.4 Hz), 129.5, 128.6, 126.8, 124.3 (d, *J* = 3.6 Hz), 116.6 (d, *J* = 20.2 Hz), 113.4, 68.1, 61.8, 56.4, 43.6, 28.7, 14.2, 9.4 ppm. – ^{19}F NMR (470.5 Hz, $CDCl_3$): –134.0 (dd, *J* = 13.0, 8.7 Hz) ppm. – IR: 2970, 1718, 1518, 1444, 1365, 1303, 1276, 1230, 1217, 1138, 1023, 778 cm^{-1}. – HRMS calcd for $C_{21}H_{23}ClFO_4$: 393.1263, found 393.1268 [$M+H^+$].

Ethyl 2-benzoyl-2-(4-methoxyphenyl)butanoate (90)

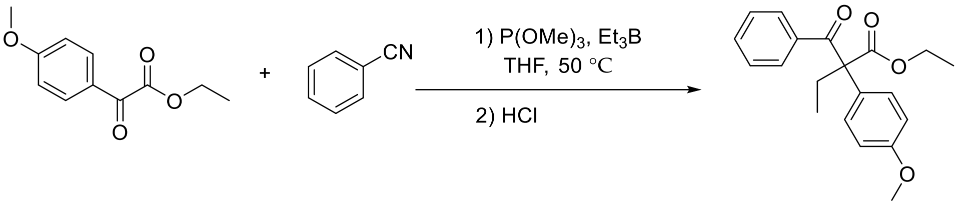

The general procedure GP3 was followed with ethyl 2-(4-methoxyphenyl)-2-oxoacetate (41.6 mg, 0.2 mmol), $P(OMe)_3$ (50 mg, 0.4 mmol, 2.0 equiv.), Et_3B (0.4 mL, 1M, 2.0 equiv.), benzonitrile (206 mg, 2.0 mmol, 10.0 equiv.) and THF (1.6 mL). The mixture was stirred at 50 °C for 16 h. Purification by flash chromatography on silica gel (EtOAc/hexane, 3 : 97 v/v) afford product **90** (37 mg, 56%) as a colorless oil.

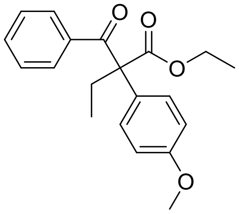

^{1}H NMR (500 MHz, $CDCl_3$): 7.71 (2 H, d, *J* = 7.8 Hz), 7.57 (2 H, d, *J* = 8.9 Hz), 7.39 (1 H, t, *J* = 7.3 Hz), 7.28–7.22 (2 H, m), 6.85 (2 H, d, *J* = 8.9 Hz), 4.13–4.00 (2 H, m), 3.78 (3 H, s), 2.38 (1 H, dq, *J* = 14.7, 7.4 Hz), 2.25 (1 H, dq, *J* = 14.7, 7.5 Hz), 0.95 (3 H, t, *J* = 7.1 Hz), 0.81 (3 H, t, *J* = 7.4 Hz) ppm. – ^{13}C NMR (125 MHz, $CDCl_3$): 195.8, 171.7, 158.6, 136.3, 132.4, 130.1, 129.5, 129.3, 128.2, 113.9, 65.1, 61.2, 55.3, 32.5, 13.8, 9.1 ppm. – IR: 2978, 1735, 1685, 1610, 1512, 1447, 1292, 1252, 1236, 1176, 1028 cm^{-1}. – HRMS calcd for $C_{20}H_{23}O_4$: 327.1591, found 327.1599 [$M+H^+$].

Ethyl 2-(3,5-bis(trifluoromethyl)benzoyl)-2-(3-fluoro-4-methoxyphenyl)butanoate (91)

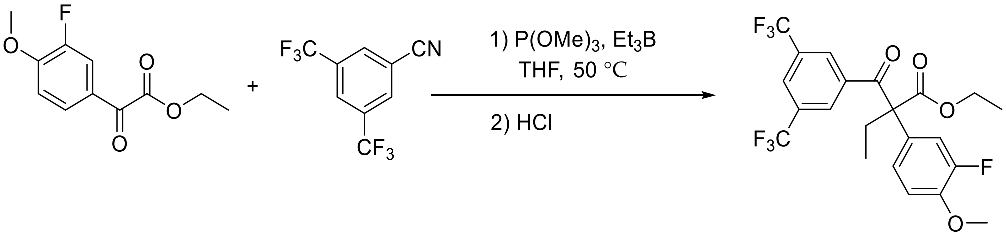

The general procedure GP3 was followed with ethyl 2-(3-fluoro-4-methoxyphenyl)-2-oxoacetate (45.2 mg, 0.2 mmol), $P(OMe)_3$ (550 mg, 0.4 mmol, 2.0 equiv.), Et_3B (0.4 mL, 1M,

2.0 equiv.), 3,5-bis(trifluoromethyl)benzonitrile (480mg, 2.0 mmol, 10.0 equiv.) and THF (1.6 mL). The mixture was stirred at 50 °C for 16 h. Purification by flash chromatography on silica gel (EtOAc/hexane, 3 : 97 v/v) afford product **91** (63.4 mg, 66%) as a colorless oil.

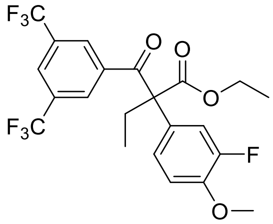

^{1}H NMR (500 MHz, $CDCl_3$): 8.12 (2 H, s), 7.90 (1 H, s), 7.46 (1 H, dd, *J* = 13.1, 2.4 Hz), 7.23 (1 H, ddd, *J* = 8.7, 2.4, 1.3 Hz), 6.91 (1 H, t, *J* = 8.8 Hz), 4.23–4.05 (2 H, m), 3.86 (3 H, s), 2.41 (1 H, dq, *J* = 14.7, 7.4 Hz), 2.24 (1 H, dq, *J* = 14.9, 7.5 Hz), 1.02 (3 H, t, *J* = 7.1 Hz), 0.84 (3 H, t, *J* = 7.5 Hz) ppm. – ^{13}C NMR (125 MHz, $CDCl_3$): 192.3, 170.3, 152.4 (d, *J* = 246.9 Hz), 147.3 (d, *J* = 10.7 Hz), 137.7, 131.9 (q, *J* = 34.0 Hz), 129.5, 129.4 (q, *J* = 3.6 Hz), 125.6 (q, *J* = 3.7 Hz), 123.8 (d, *J* = 3.6 Hz), 122.9 (q, *J* = 273.0 Hz), 115.9 (d, *J* = 20.8 Hz), 113.7, 65.0, 61.9, 56.3, 32.5, 13.8, 9.0 ppm. – ^{19}F NMR (470.5 Hz, $CDCl_3$): –63.2, –113.0 ppm. – IR: 2985, 1740, 1700, 1619, 1515, 1464, 1374, 1279, 1222, 1182, 1137, 1024, 909 cm^{-1}. – HRMS calcd for $C_{22}H_{20}F_7O_4$: 481.1244, found 481.1253 [M+H$^+$].

2-Ethyl-2-phenylpent-4-enoic acid (93)

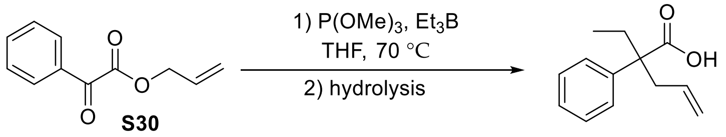

The general procedure GP1 was followed with allyl 2-oxo-2-phenylacetate (38 mg, 0.2 mmol), $P(OMe)_3$ (30 mg, 0.24 mmol, 1.2 equiv.), Et_3B (0.3 mL, 1M, 1.5 equiv.) and THF (1.7 mL). The mixture was stirred at 70 °C for 16 h. Purification by flash chromatography on silica gel (EtOAc/hexane, 20 : 80 v/v) afford product **93** (32 mg, 78%) as a colorless oil.

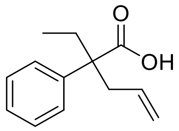

^{1}H NMR (500 MHz, $CDCl_3$): 7.37–7.19 (5 H, m), 5.52 (1 H, td, *J* = 17.1, 7.3 Hz), 5.07 (1 H, d, *J* = 17.0 Hz), 5.02 (1 H, d, *J* = 10.1 Hz), 2.83 (1 H, dd, *J* = 14.0, 7.7 Hz), 2.74 (1 H, dd, *J* = 14.0, 6.7 Hz), 2.14–1.97 (2 H, m), 0.79 (3 H, t, *J* = 7.4 Hz) ppm. – ^{13}C NMR (125 MHz, $CDCl_3$): 181.5, 141.5, 133.5, 128.5, 127.1, 126.8, 118.5,

54.1, 38.4, 26.9, 8.5 ppm. – IR: 2984, 1739, 1447, 1372, 1240, 1045, 916, 847, 732 cm^{-1}. – HRMS calcd for $C_{13}H_{17}O_2$: 205.1223, found 205.1223 [M+H+].

2-Ethyl-3,3-dimethyl-2-phenylpent-4-enoic acid (94)

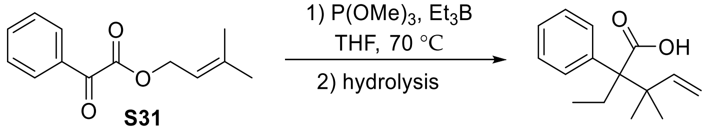

The general procedure GP1 was followed with 3-methylbut-2-en-1-yl 2-oxo-2-phenylacetate (38 mg, 0.2 mmol), $P(OMe)_3$ (43.6 mg, 0.24 mmol, 1.2 equiv.), Et_3B (0.3 mL, 1M, 1.5 equiv.) and THF (1.7 mL). The mixture was stirred at 70 °C for 16 h. Purification by flash chromatography on silica gel (EtOAc/hexane, 20 : 80 v/v) afford product **94** (28 mg, 60%) as a colorless oil.

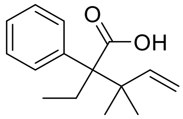

^{1}H NMR (500 MHz, $CDCl_3$): 7.45 (2 H, d, *J* = 7.5 Hz), 7.30 (2 H, t, *J* = 7.5 Hz), 7.25 (1 H, d, *J* = 7.8 Hz), 6.07 (1 H, dd, *J* = 17.4, 10.8 Hz), 5.01 (1 H, d, *J* = 10.8 Hz), 4.92 (1 H, d, *J* = 17.5 Hz), 2.37–2.13 (2 H, m), 1.07 (3 H, s), 1.05 (3 H, s), 0.89 (3 H, t, *J* = 7.2 Hz) ppm. – ^{13}C NMR (125 MHz, $CDCl_3$): 180.5, 145.6, 137.7, 130.5, 127.0, 126.6, 112.9, 62.0, 43.4, 25.7, 24.7, 24.5, 10.7 ppm. – IR: 2982, 2939, 2255, 1738, 1373, 1239, 1046, 917, 734 cm^{-1}. – HRMS calcd for $C_{15}H_{21}O_2$: 233.1536, found 233.1541 [M+H+].

2-Ethyl-2-phenylpenta-3,4-dienoic acid (95)

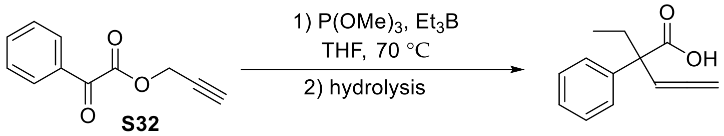

The general procedure GP1 was followed with prop-2-yn-1-yl 2-oxo-2-phenylacetate (38 mg, 0.2 mmol), $P(OMe)_3$ (30 mg, 0.24 mmol, 1.2 equiv.), Et_3B (0.3 mL, 1M, 1.5 equiv.) and THF (3.7 mL). The mixture was stirred at 70 °C for 16 h. Purification by flash

chromatography on silica gel (EtOAc/hexane, 20 : 80 v/v) afford product **95** (24 mg, 59%) as a colorless oil.

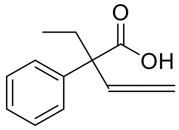

^{1}H NMR (500 MHz, $CDCl_3$): 7.40–7.19 (5 H, m), 5.77 (1 H, t, *J* = 6.8 Hz), 4.92–4.84 (2 H, m), 2.20–2.07 (2 H, m), 0.83 (3 H, t, *J* = 7.4 Hz) ppm. – ^{13}C NMR (125 MHz, $CDCl_3$): 208.1, 179.0, 141.5, 128.5, 127.3, 127.2, 93.5, 78.4, 55.3, 29.7, 9.5 ppm. – IR: 3006, 2970, 2922, 2252, 1712, 1421, 1362, 1222, 915 cm^{-1}. – HRMS calcd for $C_{13}H_{15}O_2$: 203.1067, found 203.1065 [M+H$^+$].

2-Ethyl-3-methylene-2-phenylpent-4-enoic acid (96)

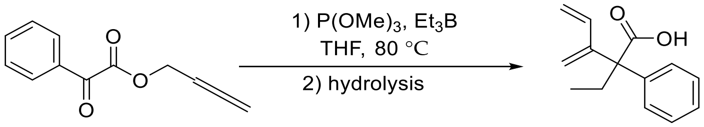

The general procedure GP1 was followed with buta-2,3-dien-1-yl 2-oxo-2-phenylacetate (40.4 mg, 0.2 mmol), $P(OMe)_3$ (30 mg, 0.24 mmol, 1.2 equiv.), Et_3B (0.24 mL, 1M, 1.2 equiv.) and THF (1.7 mL). The mixture was stirred at 80 °C for 3 h. Purification by flash chromatography on silica gel (EtOAc/hexane, 20 : 80 v/v) afford product **96** (18 mg, 42%) as a colorless oil.

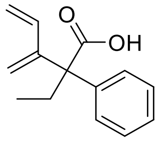

^{1}H NMR (500 MHz, $CDCl_3$): 7.42 (2 H, d, *J* = 7.5 Hz), 7.31 (2 H, t, *J* = 7.4 Hz), 7.25 (1 H, t, *J* = 6.7 Hz), 6.08 (1 H, dd, *J* = 17.3, 11.0 Hz), 5.63 (1 H, s), 5.34 (1 H, d, *J* = 17.3 Hz), 5.27 (1 H, s), 4.97 (1 H, d, *J* = 11.0 Hz), 2.34–2.08 (2 H, m), 0.86 (3 H, t, *J* = 7.2 Hz) ppm. – ^{13}C NMR (125 MHz, $CDCl_3$): 178.8, 146.8, 140.1, 136.8, 128.8, 128.1, 127.1, 115.8, 115.6, 60.0, 29.2, 9.8 ppm. – IR: 2983, 2942, 2911, 1739, 1373, 1241, 1045, 916, 732 cm^{-1}. – HRMS calcd for $C_{14}H_{17}O_2$: 217.1223, found 217.1224 [M+H$^+$].

Ethyl 2-ethyl-2-phenylpent-4-enoate (97)

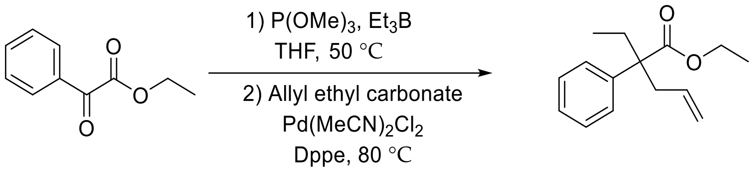

The general procedure GP1 was followed with ethyl 2-oxo-2-phenylacetate (**6**) (36 mg, 0.2 mmol), $P(OMe)_3$ (30 mg, 0.24 mmol, 1.2 equiv.), Et_3B (0.3 mL, 1M, 1.5 equiv.) and THF (1.7 mL). The mixture was stirred at 50 °C for 16 h. Another oven-dried 8 mL reaction tube was charged with a magnetic stir bar, $Pd(MeCN)_2Cl_2$ (2.6 mg, 0.01 mmol, 5 mol%), dppe (6.4 mg, 0.016 mmol, 8 mol%) and THF (1.0 mL). Allyl ethyl carbonate (52 mg, 0.4 mmol, 2.0 equiv.) was added into the tube followed with the afore mentioned reaction mixture. The mixture was stirred at 80 °C for 12 h. Purification by preparative TLC Plates (EtOAc/hexane, 3 : 97 v/v) afford product **97** (28 mg, 60%) as a colorless oil.

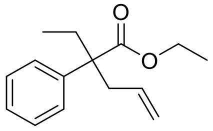

^{1}H NMR (500 MHz, $CDCl_3$): 7.43–7.13 (5 H, m), 5.53 (1 H, dq, *J* = 16.8, 7.6 Hz), 5.07 (1 H, d, *J* = 17.2 Hz), 5.03 (1 H, d, *J* = 10.1 Hz), 4.15 (2 H, q, *J* = 6.9 Hz), 2.84 (1 H, dd, *J* = 13.7, 7.9 Hz), 2.76 (1 H, dd, *J* = 13.8, 6.5 Hz), 2.07 (2 H, tq, *J* = 13.9, 7.3, 6.9 Hz), 1.19 (3 H, t, *J* = 7.2 Hz), 0.78 (3 H, t, *J* = 7.4 Hz) ppm. – ^{13}C NMR (125 MHz, $CDCl_3$): 175.6, 142.5, 133.9, 128.4, 126.7, 126.7, 118.2, 60.8, 54.1, 38.7, 27.1, 14.3, 8.5 ppm. – IR: 2980, 1740, 1446, 1373, 1240, 1046, 916 cm^{-1}. – HRMS calcd for $C_{15}H_{21}O_2$: 233.1536, found 255.1536 [M+H$^+$].

X-Ray Crystallographic Data

2-(2-Fluoro-[1,1'-biphenyl]-4-yl)butanoic acid (23-H)

CCDC 1988922

Bond precision:	C–C = 0.0031 Å		Wavelength = 1.54184
Cell:	a = 5.6436(1)	b = 36.3180(5)	c = 6.6768(1)
	α = 90	β = 107.690(2)	γ = 90
Temperature:	100 K		

	Calculated	Reported
Volume	1303.80(4)	1303.79(4)
Space group	P 21/n	P 1 21/n 1
Hall group	-P 2yn	-P 2yn
Moiety formula	$C_{16}H_{14}FO_2$	$C_{16}H_{14}FO_2$
Sum formula	$C_{16}H_{14}FO_2$	$C_{16}H_{14}FO_2$
Mr	257.27	257.27
D_x,g cm^{-3}	1.311	1.311
Z	4	4
Mu (mm^{-1})	0.783	0.783
F000	540.0	540.0
F000'	541.78	
h,k,l_{max}	7,45,8	7,45,8
N_{ref}	2733	2642
T_{min},T_{max}	0.948,0.963	0.671,1.000
T_{min}'	0.893	

Go back to table of contents

Correction method= # Reported T Limits: T_{min} = 0.671 T_{max} = 1.000

AbsCorr = GAUSSIAN

Data completeness = 0.967 Theta(max) = 76.520

R(reflections) = 0.0600(2515) wR2(reflections) = 0.1258(2642)

S = 1.046 N_{par} = 183

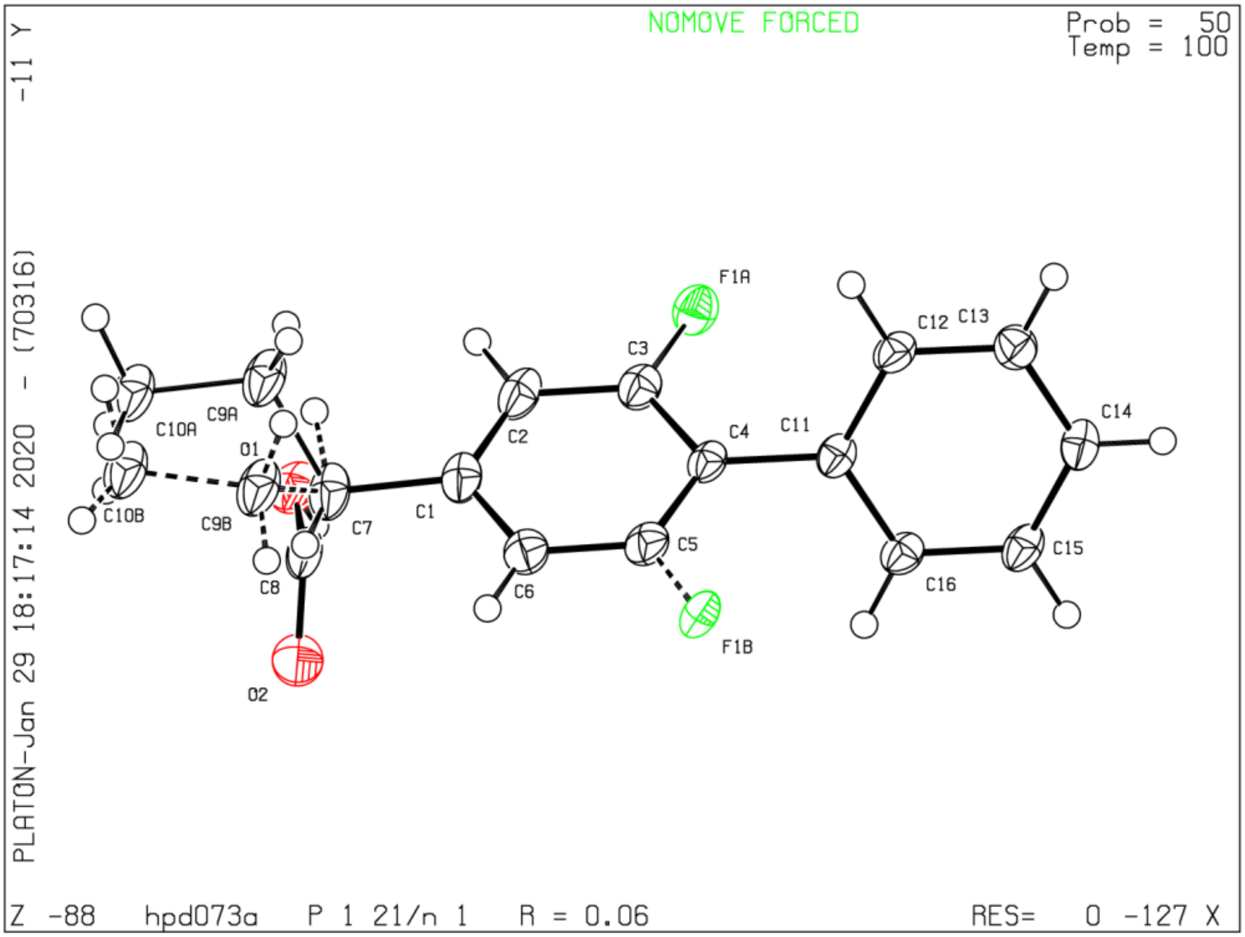

Go back to table of contents

2-([1,1'-Biphenyl]-4-yl)butanoic acid (22-H)

CCDC 1988923

Bond precision: C–C = 0.0031 Å Wavelength = 0.71073

Cell:	a = 7.1570(2)	b = 7.4823(2)	c = 11.6050(4)
	α = 90	β = 90.910(3)	γ = 90

Temperature: 98 K

	Calculated	Reported
Volume	621.38(3)	621.38(3)
Space group	P 21	P 1 21 1
Hall group	P 2yb	P 2yb
Moiety formula	$C_{16}H_{16}O_2$	$C_{16}H_{16}O_2$
Sum formula	$C_{16}H_{16}O_2$	$C_{16}H_{16}O_2$
Mr	240.29	240.29
D_x,g cm^{-3}	1.284	1.284
Z	2	2
Mu (mm^{-1})	0.083	0.083
F000	256.0	256.0
F000'	256.12	
h,k,l_{max}	8,9,14	8,9,14
N_{ref}	2429[1312]	2413
T_{min},T_{max}	0.983,0.994	0.988,1.000
T_{min}'	0.981	

Correction method = # Reported T Limits: Tmin = 0.988 Tmax = 1.000

AbsCorr = MULTI-SCAN

Data completeness = 1.84/0.99   Theta(max) = 25.986

R(reflections) = 0.0371(2315)   wR2(reflections) = 0.0918(2413)

S = 1.037 N_{par} = 165

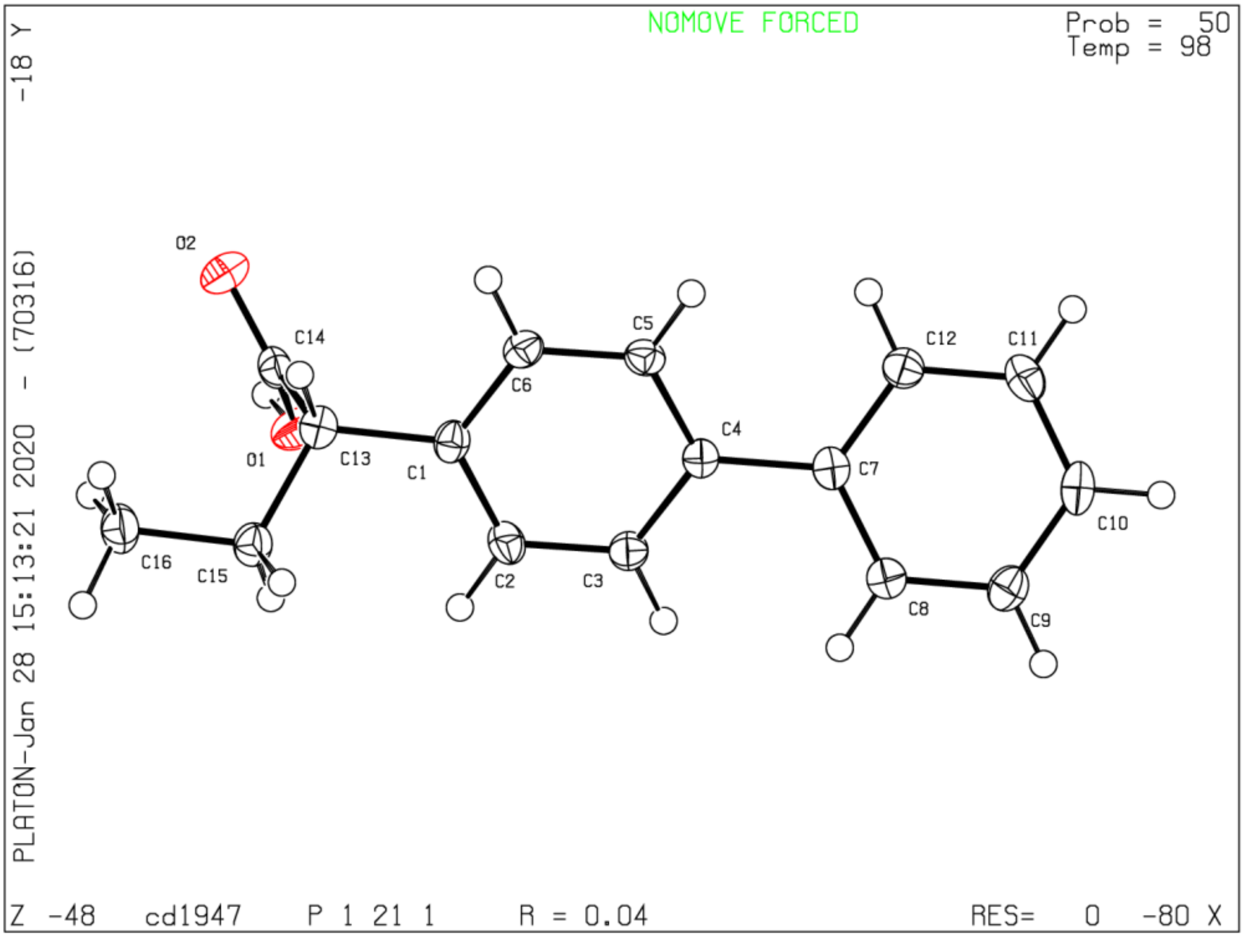

2-(9-methyl-9*H*-carbazol-3-yl)butanoic acid (35-H)

CCDC 1988924

Bond precision: C–C = 0.0063 Å Wavelength = 1.54184

Cell: a = 23.0271(3) b = 5.7175(1) c = 20.8790(4)

α = 90 β = 90 γ = 90

Temperature: 100 K

	Calculated	Reported
Volume	2748.88(8)	2748.88(8)
Space group	P n a 21	P n a 21
Hall group	P 2c -2n	P 2c -2n
Moiety formula	$C_{17}H_{17}NO_2$	$C_{17}H_{17}NO_2$
Sum formula	$C_{17}H_{17}NO_2$	$C_{17}H_{17}NO_2$
Mr	267.32	267.31
D_x,g cm^{-3}	1.292	1.292
Z	8	8
Mu (mm^{-1})	0.675	0.675
F000	1136.0	1136.0
F000'	1139.33	
h,k,l_{max}	29,7,26	28,7,26
N_{ref}	5781[2973]	5316
T_{min},T_{max}	0.917,0.968	0.787,1.000
T_{min}'	0.917	

Correction method = # Reported T Limits: T_{min} = 0.787 T_{max} = 1.000

AbsCorr = GAUSSIAN

Data completeness = 1.79/0.92 Theta(max) = 76.662

R(reflections) = 0.0565(5006) wR2(reflections) = 0.1298(5316)

S = 1.079 N_{par} = 368

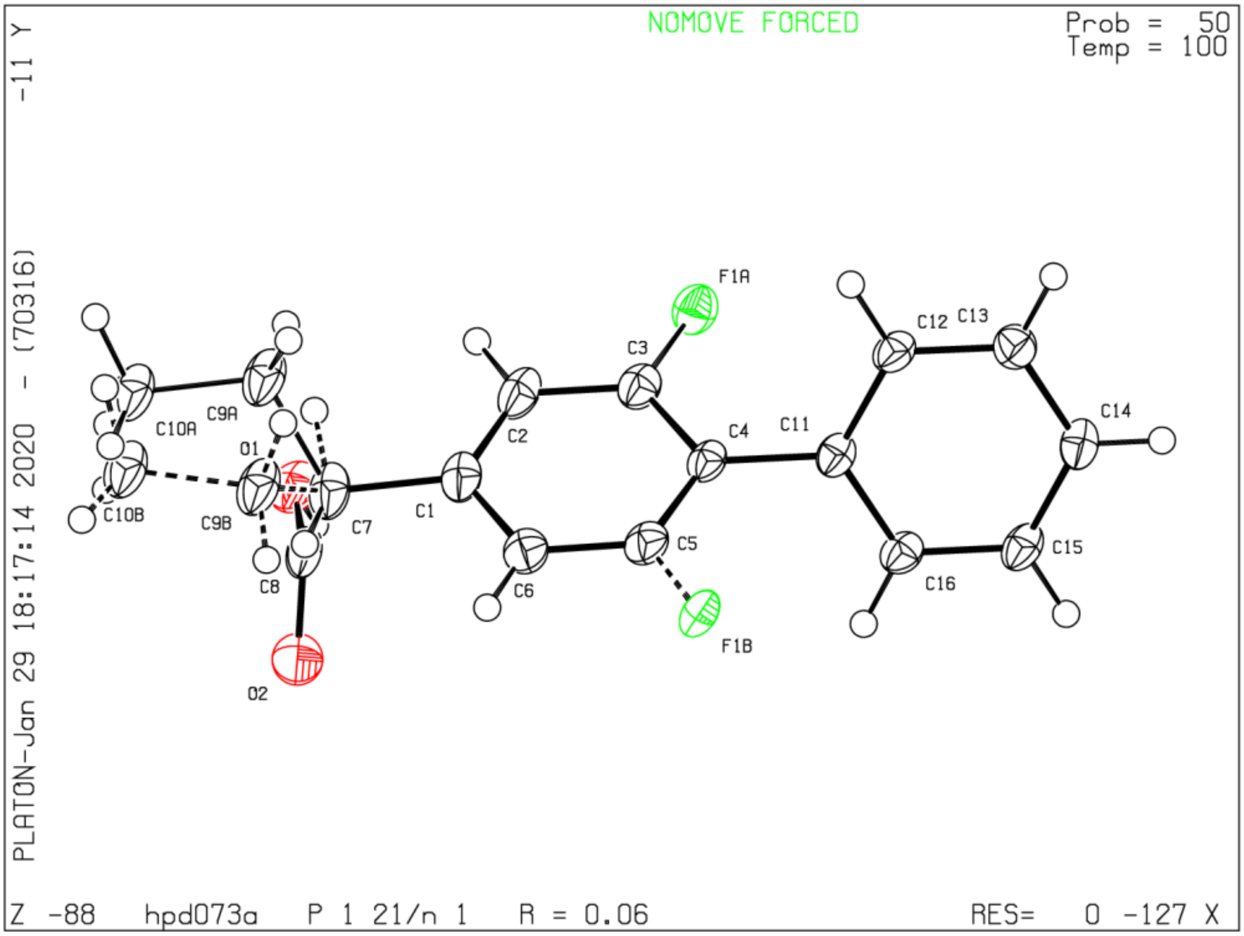

Go back to table of contents

Complex 98

CCDC 1993289

Bond precision: C–C = 0.0024 Å Wavelength = 1.54184

Cell: a = 11.3547(2) b = 17.4727(4) c = 14.7370(2)

α = 90 β = 99.889(2) γ = 90

Temperature: 100 K

	Calculated	Reported
Volume	2880.34(9)	2880.34(9)
Space group	P 21/c	P 1 21/c 1
Hall group	-P 2ybc	-P 2ybc
Moiety formula	$C_{28}H_{47}BN_3O_2P$	$C_{28}H_{47}BN_3O_2P$
Sum formula	$C_{28}H_{47}BN_3O_2P$	$C_{28}H_{47}BN_3O_2P$
Mr	499.47	499.46
D_x,g cm^{-3}	1.152	1.152
Z	4	4
Mu (mm^{-1})	1.055	1.055
F000	1088.0	1088.0
F000'	1091.84	
h,k,l_{max}	14,22,18	14,21,18
N_{ref}	6058	5795
T_{min},T_{max}	0.896,0.950	0.513,1.000
T_{min}'	0.763	

Correction method = # Reported T Limits: T_{min} = 0.513 T_{max} = 1.000

AbsCorr = GAUSSIAN

Data completeness = 0.957 Theta(max) = 76.534

R(reflections) = 0.0466(5058) wR2(reflections) = 0.1165(5795)

S = 1.012 N_{par} = 327

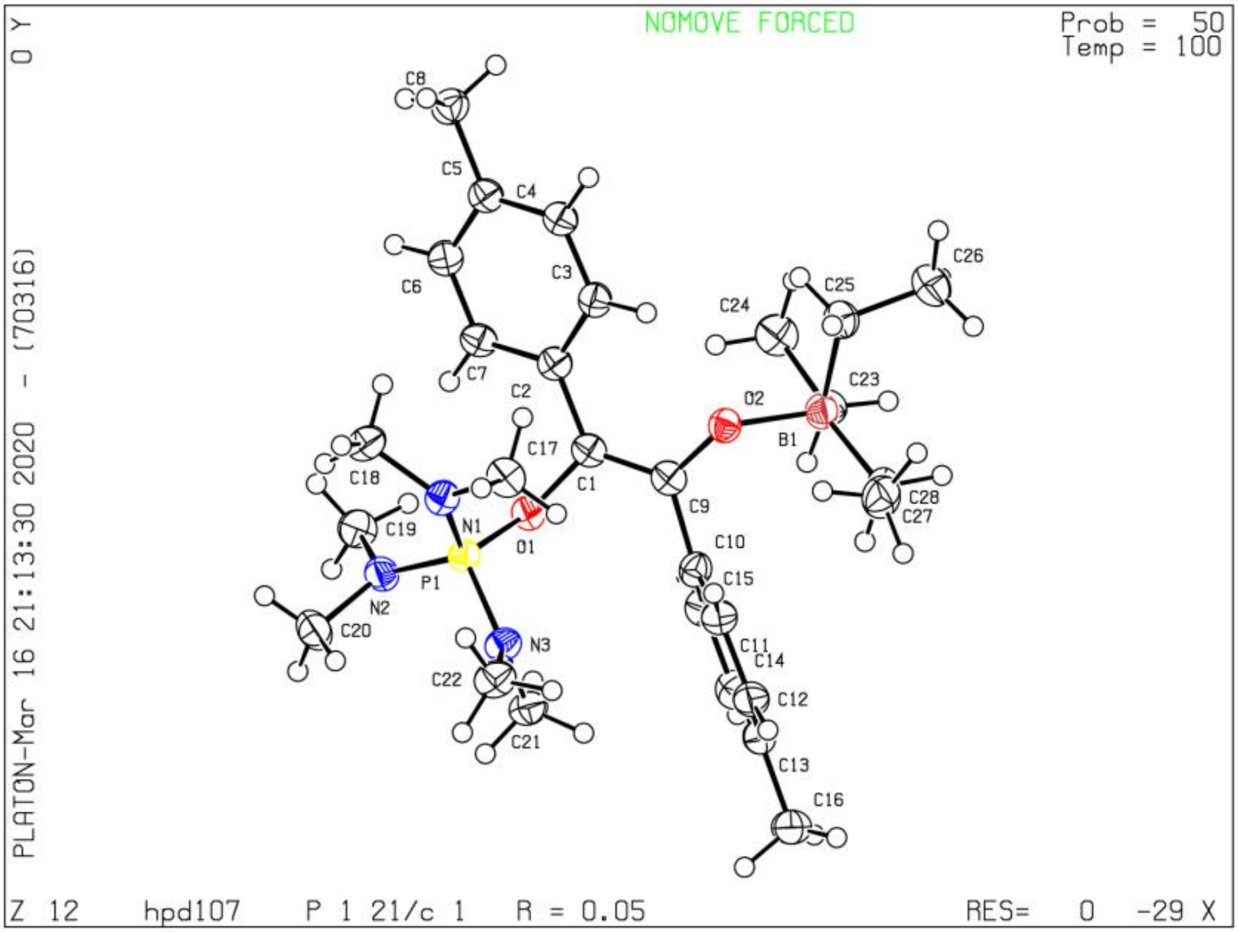

Computational Data

Software

All geometry optimizations, vibrational frequency calculations, and IRCs were conducted using the Gaussian 16 program.[15] DFT Calculations were performed using the Stampede2 supercomputer at the Texas Advanced Computing Center (TACC) hosted by the University of Texas in Austin, Texas.[16] Supplemental conformational searches were conducted using Gabedit 2.5.0.[17] Thermochemical corrections to low magnitude frequency vibrations were applied using GoodVibes.[18] General day-to-day visualization and monitoring of calculations was performed with Chemcraft.[19] Final images of minima and transition state geometries were rendered using CYLview.[20]

Computational Methods

All geometries were optimized first at the MN15[21]/Def2-SVP[22,23]/SMD[24] (THF) level of theory (see below for the details of the theoretical method selection). The convergence criterion was set to "tight" and an "ultrafine" grid was selected. Frequency calculations at the optimized geometries confirmed the nature of the stationary point isolated and provided the enthalpic and entropic correction terms for thermodynamic calculations. Geometries with zero imaginary frequencies were deemed stable minima whereas those with exactly one imaginary frequency were deemed transition states. Transition states were subjected to further IRC calculations to confirm that the transition state connected reactants to products along chemical paths of interest. The quasi-harmonic approximation from Grimme[25] was applied via GoodVibes to all structures to correct for potential errors associated with low magnitude vibrational frequencies with a 50 cm^{-1} cut-off. The electronic component of the Gibbs free energy obtained at the double-zeta def2-SVP level was then replaced with electronic energy obtained at the triple-zeta 6-311G** level of theory.

Conformational searches

An extensive sampling of possible conformations was conducted to attempt to locate an operative representation of the global minimum for each structure. An exhaustive manual conformational search was further augmented by an in-depth conformational investigation using Gabedit's AMBER-based conformational search tool.

Selection of the theoretical method

The MN15[21] functional was selected on the basis of its excellent performance with three-center two-electron bond systems that are commonly observed for organoboron intermediates and transition state structures including those in the present study,[26] as well as based on the excellent performance in kinetic modeling studies of relevant addition and 1,*n*-shift reactions[27] and its overall broad usefulness for noncovalent interactions, isomerization energies, thermochemistry, and barrier heights.[28] The def2-SVP[29,30] basis set was selected for initial optimization of geometries based on its parameterization for all atoms present in the structures as well as its relative speed. In order to assess the performance of the selected method, calculations of free energy of formation of complex **S33** ($(MeO)_3P–BEt_3$) from trimethyl phosphite and triethylborane were performed with the selected method as well as a range of combinations of other functionals (PW6B95D3[31] (with D3BJ[32]), M062X[33,34]-D3[35], M052X[36]-D3, B3LYP[37,38,39]-D3BJ, PBE0[40,41]-D3BJ, wB97X-D[42], and M11[43]) and several basis sets. The calculated values were compared with the experimentally determined value (see the Mechanistic Studies section), and the selected method was found to give the best fit of all the tested methods. The suitability of the selected method is further reflected in the excellent fit of the computed barrier (22.9 kcal/mol) with the experimentally determined value (22.7 kcal/mol, see the Mechanistic Studies section) and the consistency of the kinetic second

Go back to table of contents

order of the computed pathway with the experimentally observed overall second order for the reaction.

Discussion of the alternative pathways for the formation of adducts 100 and S34 and the deoxygenative α-alkylation proceeding via acyclic adduct S34.

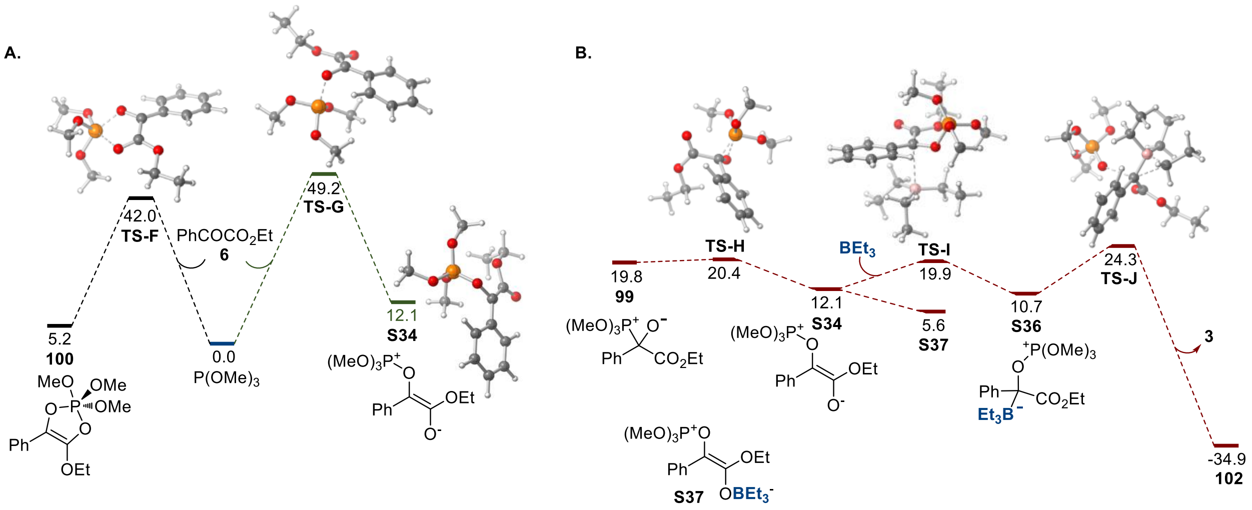

Figure S10. Computed alternative pathways. **A.** Formation of cyclic adduct **100** by a cheletropic [4+1] cycloaddition of ester **6** with trimethyl phosphite and direct *O*-addition of trimethyl phosphite to ester **6**. **B.** Deoxygenative α-alkylation via acyclic Kukhtin-Ramirez adduct **S34**.

Direct addition of phosphite to the keto oxygen of ester **6** to intermediate **S34** (Figure S10.A) is kinetically disfavored (**TS-G**, $\Delta G^{\neq}$ = 49.2 kcal/mol). Furthermore, the cheletropic [4+1] cycloaddition of trimethyl phosphite and ester **6** producing adduct **100** is also highly kinetically unfavorable (**TS-F**, $\Delta G^{\neq}$ = 42.0 kcal/mol).

The acyclic form of ester–phosphite adduct **S34** is also kinetically accessible from intermediate **99** (via **TS-H**, Figure S10.B) that can be formed by a sequence of the addition of phosphite to the carbon atom of the carbonyl group followed by the phospha-Brook

migration of the phosphorus to the oxygen atom as described for the cyclic adduct **100** (Figure 1). However, the subsequent sequence of organoborane addition (to **S37** via **TS-I**) and Matteson-type displacement steps proceeded over higher barriers (**TS-J**), making the pathway via acyclic zwitterion **100** kinetically less favorable. Consistent with the higher barrier for this pathway, the experimentally observed reaction is overall second order, indicating that the reaction proceeds via cyclic adduct **100** as shown in Figure 1. Also consistent with the experimental observations, formation of adduct **S37** was not thermodynamically favorable for ester **6** and trimethyl phosphite.

Optimized Geometries

Stable Minima

S33

E(RMN15) = -947.840348426

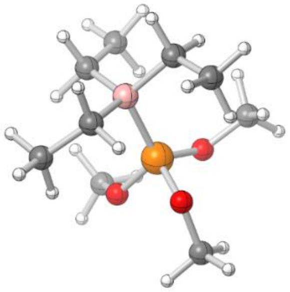

Zero-point correction= 0.332612 (Hartree/Particle)

Thermal correction to Energy= 0.353187

Thermal correction to Enthalpy= 0.354131

Thermal correction to Gibbs Free Energy= 0.284767

Sum of electronic and ZPE= -947.507736

Sum of electronic and thermal Energies= -947.487162

Sum of electronic and thermal Enthalpies= -947.486217

Sum of electronic and thermal Free Energies= -947.555581

	E, kcal/mol	CV, cal/(mol·K)	S, cal/(mol·K)
Total	221.628	73.085	145.989

Charge = 0 Multiplicity = 1

P	-1.8894669656	0.6128078889	1.1660775577
O	-0.658394473	0.3074564112	0.1954564428
O	-3.0792573337	-0.1646444519	0.4217647102
O	-2.3439702283	2.123273082	0.8322487092
C	-1.7141526408	3.2873590446	1.3560499417
H	-0.6301336861	3.1431033748	1.4821832097
H	-1.878490497	4.1026281622	0.6395641162
H	-2.1618880602	3.5650213709	2.3217275748
C	-0.7583856624	0.4833273121	-1.2169175517
H	0.2603932867	0.5789242413	-1.6116470486
H	-1.2481709252	-0.38676065	-1.6757815866
H	-1.3276324747	1.3928335464	-1.4614114883
C	-4.4594297152	0.1714011932	0.4982419504
H	-4.9799244053	-0.413662048	-0.270367889
H	-4.8788564242	-0.083172581	1.4829112492
H	-4.6116307719	1.2418265635	0.2999666292
B	-1.5059959781	0.0588830871	3.0077012173
C	-0.7756543758	-1.397129806	2.8434988671
H	0.1289616686	-1.3035472238	2.2161220502
H	-0.3985883619	-1.6444480757	3.8570458959
C	-0.5184458333	1.1476800857	3.7195611492
H	-0.4704682033	0.8346267537	4.7826269275
H	-0.9673955697	2.1558090991	3.7525550003
C	-2.9691303672	-0.0344285937	3.7290139186
H	-3.6271492053	-0.7513549433	3.2029200436
H	-2.7844173618	-0.5018818071	4.7171805978

C	-1.6208648277	-2.5597141602	2.3270509253
H	-2.0190386579	-2.3538026008	1.3194392047
H	-1.0536075133	-3.5033549637	2.2652749626
H	-2.4907498529	-2.7499780866	2.9766084929
C	-3.7139917116	1.2850249121	3.9301294729
H	-3.9057188241	1.7961192558	2.9689974
H	-4.6921031909	1.158923816	4.4235813164
H	-3.1309401074	1.9875149463	4.5475989618
C	0.9032768885	1.2421396546	3.1664840647
H	0.9060396613	1.4576980998	2.0835179212
H	1.5060831542	2.0231881379	3.6591448034
H	1.4461821864	0.2914659228	3.2896276695

S34

E(RMN15) = -1297.42917484

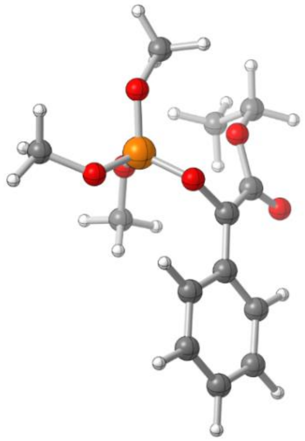

Zero-point correction= 0.315397 (Hartree/Particle)

Thermal correction to Energy= 0.337594

Thermal correction to Enthalpy= 0.338538

Thermal correction to Gibbs Free Energy= 0.263147

Sum of electronic and ZPE= -1297.113778

Sum of electronic and thermal Energies= -1297.091581

Sum of electronic and thermal Enthalpies= -1297.090637

Sum of electronic and thermal Free Energies= -1297.166027

	E, kcal/mol	CV, cal/(mol·K)	S, cal/(mol·K)
Total	211.843	79.641	158.672

Charge = 0 Multiplicity = 1

C	-0.8227064293	0.9642023505	-0.0514961415
O	-0.0043423398	2.1125631543	0.2528980719
C	-2.2112865292	1.1083758545	0.2822557287
C	-3.1518878614	0.0628283616	0.0756037266
C	-2.7128548462	2.3249510031	0.8153688548
C	-4.4970203529	0.235632322	0.3881431578
H	-2.7929606122	-0.8806992508	-0.3349673197
C	-4.0643813744	2.4801213004	1.1235329624
H	-2.0257497677	3.1555343723	0.9931565348
C	-4.9756503645	1.4413573255	0.9164250653
H	-5.1890209227	-0.5941280977	0.2164643254
H	-4.4076636951	3.4336216028	1.5353532566
H	-6.0329258806	1.5658972163	1.1607652055
C	-0.196757683	-0.0639579209	-0.7786235856
O	1.1196075135	0.2402242835	-1.0858359935
C	1.7590980679	-0.6366736895	-2.0024903216
H	1.5378631646	-1.6772129162	-1.7228800284
H	2.8391740825	-0.4672793159	-1.876379742
C	1.325824727	-0.3712752664	-3.4305516343
H	1.5219310979	0.6733594691	-3.7192010578
H	1.8672724339	-1.0296656377	-4.1266633047
H	0.24951238	-0.5711258709	-3.535930557
O	-0.6931537076	-1.1260994036	-1.1631849842
P	0.5548227286	3.0847219784	-0.8099301767
O	-0.0035754802	4.4862201981	-0.3947739707

O	2.0963841616	3.2534490209	-0.7776655572
O	0.1654715798	2.7364887441	-2.2790631255
C	-1.1765500471	2.5113418928	-2.7566301404
H	-1.9184995092	2.8939178076	-2.0400563037
H	-1.2734815251	3.0438132124	-3.7102224672
H	-1.3263647673	1.4339642364	-2.9022209003
C	0.4724695414	5.7062684187	-0.9853631842
H	-0.2384947487	6.4923699337	-0.7098749372
H	1.4678853972	5.9481573345	-0.5902492596
H	0.5133187455	5.6166595288	-2.0807902489
C	3.1191011391	2.4036472443	-1.3187262644
H	3.3025518574	1.5690198022	-0.6326852294
H	2.81561178	2.0291886317	-2.3050325329
H	4.0177370461	3.0230457694	-1.4132949211

100

E(RMN15) = -1297.44816044

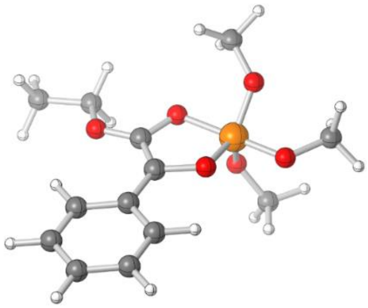

Zero-point correction= 0.317016 (Hartree/Particle)

Thermal correction to Energy= 0.338537

Thermal correction to Enthalpy= 0.339481

Thermal correction to Gibbs Free Energy= 0.263641

Sum of electronic and ZPE= -1297.131145

Sum of electronic and thermal Energies= -1297.109624

Sum of electronic and thermal Enthalpies= -1297.108680

Sum of electronic and thermal Free Energies= -1297.184519

	E, kcal/mol	CV, cal/(mol·K)	S, cal/(mol·K)
Total	212.435	78.070	159.618

Charge = 0 Multiplicity = 1

C	0.7384664427	1.5009860998	0.9105886716
C	-0.2767831213	1.2019951296	1.7571041085
O	-1.4268475413	0.9541971866	1.139199576
O	0.2551741013	1.4640233835	-0.4023943925
O	-0.2448287953	1.1699488405	3.0838487337
C	-1.353147858	0.5709720435	3.7635985889
H	-2.2688551368	1.1380187332	3.5338522209
H	-1.4955289837	-0.4553813312	3.3887752955
C	-1.0534405995	0.5844586707	5.2410790023
H	-0.9119858817	1.6137954202	5.6012345502
H	-1.8904385801	0.1362834919	5.7948439653
H	-0.1436246891	0.0078671271	5.462298368
C	2.1359848274	1.8329135371	1.1066078324
C	2.9587400274	2.0860157143	-0.0088750034
C	2.70983029	1.9140499119	2.3934568179
C	4.3059995393	2.4061250022	0.1579586471
H	2.5265586162	2.0270972179	-1.0093253437
C	4.0570252296	2.2323984349	2.5485671839
H	2.089402666	1.7261160544	3.2708672015
C	4.8666875936	2.4813479431	1.4351904087
H	4.9241992144	2.5987374891	-0.7226705496
H	4.4800866496	2.2887862111	3.5548186252

H	5.9221286869	2.7318505928	1.563443887
P	-1.2339490441	0.8444370105	-0.6059533422
O	-2.6202725151	1.6352018998	-0.7799240702
O	-0.9639464787	0.8843369718	-2.2202530076
O	-1.3524238873	-0.753133813	-0.4462424489
C	-3.5970801661	1.9532992249	0.1985576268
H	-4.4525240866	2.3778427675	-0.3433729863
H	-3.9244507683	1.0595900925	0.7472278519
H	-3.2188623942	2.6946777741	0.9147177562
C	-0.9423496794	-1.6911356184	-1.4313866384
H	-1.6639455257	-1.7574364161	-2.2585825512
H	0.0470594051	-1.4413935748	-1.8385549613
H	-0.8892254939	-2.6689610353	-0.9349244139
C	-1.9893226026	0.7803893253	-3.1792621231
H	-2.4281606855	1.76569426	-3.4013126298
H	-1.5507895272	0.3735126013	-4.1026337533
H	-2.8076875978	0.1145761253	-2.8577764737

S35

E(RMN15) = -536.351095439

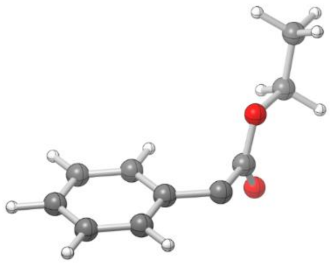

Zero-point correction= 0.176036 (Hartree/Particle)

Thermal correction to Energy= 0.187471

Thermal correction to Enthalpy= 0.188415

Thermal correction to Gibbs Free Energy= 0.136835

Sum of electronic and ZPE= -536.175060

Sum of electronic and thermal Energies= -536.163624

Sum of electronic and thermal Enthalpies= -536.162680

Sum of electronic and thermal Free Energies= -536.214260

	E, kcal/mol	CV, cal/(mol·K)	S, cal/(mol·K)
Total	117.640	41.237	108.559

Charge = 0 Multiplicity = 1

C	2.6431426248	0.4102073431	1.3199224822
O	2.270336313	-0.6110705095	0.5453076117
C	2.004364155	-1.8528679291	1.2091702803
H	1.1946077128	-1.7049486399	1.9407465099
C	1.6314321504	-2.8729861688	0.1631613631
H	1.4205353066	-3.8397067894	0.6421818668
H	0.7341143858	-2.5582409505	-0.3893321625
H	2.4512632481	-3.0148183716	-0.5558475564
O	2.7211279656	0.3545379393	2.5366447951
C	3.0717653296	1.5927780068	0.6032387066
C	2.1182416662	2.6218000526	0.3697075514
C	2.5579611627	3.8068625232	-0.2811712655
C	0.7446124423	2.5177976493	0.7373161093
C	1.6709838062	4.8380619129	-0.5592737471
H	3.6132437104	3.8751516474	-0.554912297
C	-0.1383460198	3.5498980496	0.4633496952
H	0.3935470007	1.6136318489	1.2428804607
C	0.3282211443	4.7047034273	-0.1844631116
H	2.0116276131	5.745098844	-1.0617639989

H	-1.1897974967	3.4706209967	0.7456551614
H	-0.3718597822	5.5158872778	-0.399781266
H	2.9000844309	-2.15503738	1.7742330313

99

E(RMN15) = -1297.41228062

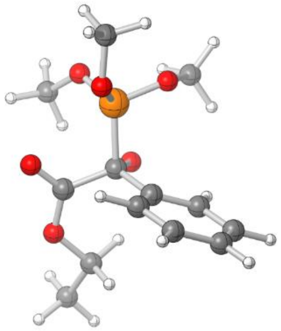

Zero-point correction= 0.314483 (Hartree/Particle)

Thermal correction to Energy= 0.337217

Thermal correction to Enthalpy= 0.338161

Thermal correction to Gibbs Free Energy= 0.261724

Sum of electronic and ZPE= -1297.097798

Sum of electronic and thermal Energies= -1297.075063

Sum of electronic and thermal Enthalpies= -1297.074119

Sum of electronic and thermal Free Energies= -1297.150556

	E, kcal/mol	CV, cal/(mol·K)	S, cal/(mol·K)
Total	211.607	80.413	160.875

Charge = 0 Multiplicity = 1

C	-1.5431583525	-0.8781665955	-2.223405905
O	-1.441757489	0.1281238048	-3.1099646185
C	-2.5523521376	-0.7114775137	-1.0831257437
C	-2.6776846346	-1.6656877779	-0.0656047485
C	-3.3772908535	0.4137705559	-1.0972689379
C	-3.6202040766	-1.4798898951	0.9464915943
H	-2.0407991032	-2.5561634521	-0.0724337354

C	-4.3211166601	0.5972396834	-0.0836462941
H	-3.2415164848	1.1310144163	-1.9112800037
C	-4.4424630967	-0.3479880094	0.9385378168
H	-3.7164365306	-2.2207518302	1.7437472452
H	-4.9648284097	1.4804108808	-0.0893343563
H	-5.1799295242	-0.2043297194	1.7318457273
C	-0.2127567437	-1.4223738766	-1.6710047095
O	0.5735053379	-0.6008191872	-0.9954844782
C	0.1736867375	0.7375646706	-0.6495925109
H	-0.4270948415	0.6882047735	0.272803071
H	-0.4497314139	1.1406148195	-1.4601234422
C	1.4237814327	1.5555255148	-0.4407287339
H	2.0122252758	1.6140578958	-1.3679882519
H	1.1524984851	2.5773460947	-0.1390028931
H	2.054822599	1.1191607023	0.3473023049
O	0.1287048391	-2.5700446733	-1.8835605211
P	-2.1808945579	-2.0779986938	-3.416375677
O	-2.6195719043	-3.4978244738	-2.8814574105
O	-3.5329850137	-1.5240762761	-4.0154864847
O	-1.223337625	-2.2966138394	-4.6474701463
C	0.1053114649	-1.8002684261	-4.8478107887
H	0.8323031853	-2.5108745084	-4.4354167804
H	0.250481915	-1.705305586	-5.9311650228
H	0.1997163701	-0.8171124281	-4.3671695364
C	-3.2923730781	-4.4528934723	-3.7065967774
H	-3.3440779868	-5.3913718094	-3.1428828423
H	-4.3082084203	-4.1057243453	-3.9411831708

H	-2.7324252585	-4.6187442869	-4.6386860081
C	-3.6354375823	-0.4230507229	-4.9174655263
H	-4.4961114856	-0.6189703216	-5.5702784238
H	-3.7878273396	0.5068770704	-4.3565979432
H	-2.7227940279	-0.3306625112	-5.5218775976

101

E(RMN15) = -1559.39953083

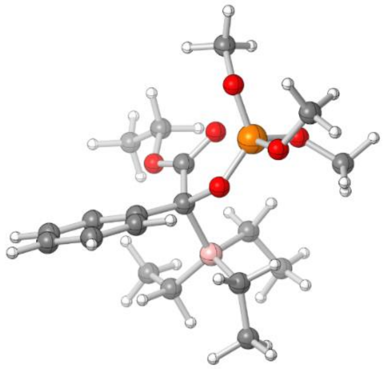

Zero-point correction= 0.519467 (Hartree/Particle)

Thermal correction to Energy= 0.551531

Thermal correction to Enthalpy= 0.552475

Thermal correction to Gibbs Free Energy= 0.457263

Sum of electronic and ZPE= -1558.880064

Sum of electronic and thermal Energies= -1558.848000

Sum of electronic and thermal Enthalpies= -1558.847056

Sum of electronic and thermal Free Energies= -1558.942268

	E, kcal/mol	CV, cal/(mol·K)	S, cal/(mol·K)
Total	346.091	119.409	200.390

Charge = 0 Multiplicity = 1

C	0.9833365907	0.9403451011	0.8917080843
C	-0.169450711	1.6730594311	1.4039447532
O	-1.2317816998	1.5832634838	0.7385071504
O	0.5326384872	0.502364769	-0.4017919012

O	-0.1242935703	2.3263239291	2.5189138142
C	-1.3552296384	2.8486661545	3.0696153017
H	-1.6860897246	3.6815514396	2.4310912801
H	-2.1134447028	2.0535076931	3.0186832085
C	-1.0801559706	3.2836170264	4.4844686249
H	-0.2818254594	4.0390071282	4.5168112993
H	-1.9910469391	3.7248239357	4.9127322139
H	-0.7849415932	2.4265285907	5.1065795154
C	2.2801557639	1.6841474248	0.7306023667
C	2.7931291355	2.5138721223	1.7417486976
C	3.0303170407	1.5268243694	-0.4456898714
C	4.0127962155	3.169861747	1.5750656196
H	2.2371247867	2.6455629473	2.6696185376
C	4.2549581967	2.1805406828	-0.6057530466
H	2.6448414843	0.8913316945	-1.2442279863
C	4.7533888397	3.0071573996	0.4012144682
H	4.3881720724	3.8122446248	2.3754079206
H	4.8196059475	2.0401693421	-1.5307960783
H	5.7101152127	3.5191660966	0.2751885607
P	-0.9245791481	0.6815169833	-0.9889040526
O	-2.1363645714	-0.2377832544	-0.5302856746
O	-1.4481752522	2.0200245871	-1.6584821597
O	-0.4951389244	-0.0381172712	-2.3668896024
C	-2.270049527	-1.6328448433	-0.8064644761
H	-3.2665976024	-1.8046020374	-1.2344297579
H	-1.5012712879	-1.9832760884	-1.5081594619
H	-2.1787337825	-2.1889554449	0.1374358243

C	-1.3661832167	-0.041812223	-3.4842158082
H	-2.4195928427	-0.1816620603	-3.187824927
H	-1.2838719377	0.9004785077	-4.045389303
H	-1.0726628993	-0.8755974966	-4.135728884
C	-2.4377129593	2.9237204994	-1.1753291087
H	-1.9932841266	3.6647728203	-0.4985791486
H	-2.8495717167	3.4346253172	-2.0545303501
H	-3.2421901141	2.3860374458	-0.6552696968
B	1.1202400258	-0.5311178958	1.9200034361
C	1.7307662228	-1.6233046909	0.8442172037
H	0.8977213064	-2.0235253673	0.2299608269
H	2.384761429	-1.1112342427	0.1128398077
C	-0.4007714713	-0.91186979	2.4011179052
H	-0.7917631376	-0.1569838957	3.1149697388
H	-1.0984673789	-0.8745766682	1.5461044713
C	2.126098576	-0.3167555886	3.1920668947
H	2.360499603	-1.3451150156	3.5287386767
H	3.1058081724	0.0807764336	2.8619113116
C	2.5343153408	-2.7896247025	1.4247907024
H	2.8196237576	-3.5278537512	0.6551119546
H	3.4710134553	-2.4389335987	1.8879768665
H	1.9850151875	-3.3381734534	2.2076741919
C	-0.5139262538	-2.2902789478	3.0506264885
H	-0.2618890042	-3.0888614295	2.3333555469
H	0.1807767575	-2.3984670063	3.9018532847
H	-1.5264842767	-2.5094114336	3.4312689534
C	1.6510070054	0.4424509121	4.4330151906

H	0.7085044695	0.0185387356	4.8217240132
H	2.3817869941	0.3944829148	5.2589094703
H	1.4549982437	1.5081825566	4.2426271393

102

E(RMN15) = -798.430235560

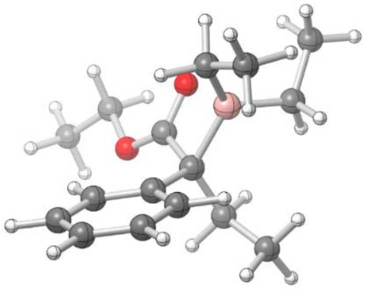

Zero-point correction= 0.382369 (Hartree/Particle)

Thermal correction to Energy= 0.403659

Thermal correction to Enthalpy= 0.404604

Thermal correction to Gibbs Free Energy= 0.332450

Sum of electronic and ZPE= -798.047866

Sum of electronic and thermal Energies= -798.026576

Sum of electronic and thermal Enthalpies= -798.025632

Sum of electronic and thermal Free Energies= -798.097786

	E, kcal/mol	CV, cal/(mol·K)	S, cal/(mol·K)
Total	253.300	80.228	151.861

Charge = 0 Multiplicity = 1

C	-0.8460015828	1.7023601934	0.5311545807
B	-0.5236492974	0.4834177249	-0.5901662239
C	0.9931414657	-0.016015021	-0.756588817
H	1.7032063621	0.7455224046	-0.3841064466
H	1.1841688598	-0.0828932355	-1.8450053885
C	1.3086114719	-1.3736969585	-0.1275369503

H	0.6611495423	-2.1654515941	-0.5392774926
H	2.350672837	-1.6871185134	-0.3027277498
H	1.1547079589	-1.3740973005	0.9630996643
C	-1.675358932	-0.562709941	-0.9577281912
H	-1.6493266013	-1.3758667573	-0.206084156
H	-2.6788913703	-0.1110868824	-0.863068714
C	-1.5142653871	-1.1583119892	-2.3569954971
H	-1.5110361391	-0.366247928	-3.1237609147
H	-0.5617317008	-1.7033281082	-2.4588354548
H	-2.3200583004	-1.8634470292	-2.6184023454
C	-0.8309723339	2.5454943992	-0.6933177743
O	-1.0561590997	3.8195653291	-0.7760787246
O	-0.6590360297	1.8106940567	-1.6919879173
C	-1.098308816	4.4206265272	-2.0953960356
H	-0.1404898924	4.2118915832	-2.5936210258
H	-1.8944495724	3.9207045745	-2.6662407504
C	-1.350226005	5.8951445845	-1.9304934144
H	-1.3946883404	6.3679830695	-2.9214462346
H	-2.3054845661	6.0769374481	-1.41809929
H	-0.5428911635	6.3723019252	-1.3570002246
C	-2.2756289184	1.7937664965	1.1216652003
H	-2.9969423975	1.7075705773	0.290804808
H	-2.4169730761	2.8044005661	1.5440566348
C	-2.6027007595	0.7459076448	2.1750154688
H	-3.6672493505	0.7968811702	2.4493700936
H	-2.3981478622	-0.271069121	1.8049392228
H	-2.0196860437	0.8948736833	3.0955489382

C	0.2210192517	2.0030826959	1.5582126149
C	0.6056859048	0.9856052822	2.4487771557
C	0.8452044979	3.2533867365	1.6793015379
C	1.5768287489	1.2075988917	3.4246752483
H	0.1337092736	0.0033570216	2.3632398915
C	1.8258462836	3.4769882096	2.6516362532
H	0.5623595121	4.0696060404	1.0113503878
C	2.1956876522	2.4572768563	3.5286343963
H	1.8576023599	0.3982438607	4.1031240117
H	2.3007375855	4.4587915466	2.7214402071
H	2.9615746806	2.6320722001	4.2878255378

103

E(RMN15) = -798.448846912

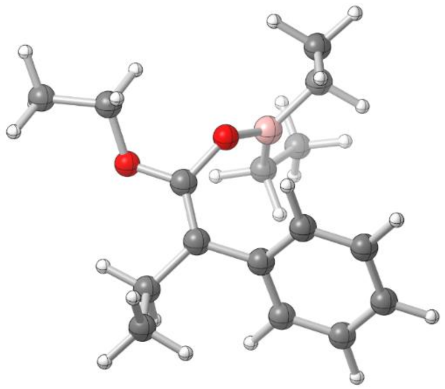

Zero-point correction= 0.381242 (Hartree/Particle)

Thermal correction to Energy= 0.403458

Thermal correction to Enthalpy= 0.404402

Thermal correction to Gibbs Free Energy= 0.327860

Sum of electronic and ZPE= -798.067605

Sum of electronic and thermal Energies= -798.045389

Sum of electronic and thermal Enthalpies= -798.044445

Sum of electronic and thermal Free Energies= -798.120987

	E, kcal/mol	CV, cal/(mol·K)	S, cal/(mol·K)
Total	253.174	80.868	161.098

Charge = 0 Multiplicity = 1

C	-3.2986520969	1.086742157	-0.4147295842
C	-2.4556708337	2.0743941025	-0.0361740163
C	-2.9995306813	3.4277802328	0.3592923541
H	-2.8329546689	3.5953563471	1.4386046423
H	-4.0890503656	3.420033488	0.2213411805
C	-2.3806239658	4.5800271607	-0.4282380178
H	-2.5553839793	4.4576625951	-1.5086029437
H	-2.8113604761	5.5456727132	-0.1227550821
H	-1.2919055803	4.6377203324	-0.2725520654
C	-0.9927822876	1.8343476418	0.0705158649
C	-0.2752075277	1.1003500901	-0.8937794225
C	-0.2736120076	2.3543319408	1.1632881478
C	1.0927978783	0.8638084249	-0.7502099795
H	-0.7982399763	0.7215558345	-1.7743078486
C	1.095738687	2.1218385176	1.3052329042
H	-0.7980406928	2.940315648	1.9225947961
C	1.7860416188	1.3677660569	0.353478919
H	1.6229474582	0.2907511055	-1.515210033
H	1.6254807785	2.5310660494	2.1690609622
H	2.8569680406	1.1832033622	0.4650701374
O	-4.634740858	1.2399182392	-0.4244250049
C	-5.3786712321	0.563932246	-1.4382907096
H	-5.3217086143	-0.5249321968	-1.2808595474
H	-4.9330968738	0.7810728063	-2.4246514486
C	-6.8071756732	1.0440816685	-1.367298143

H	-6.8678475808	2.1267603659	-1.5499319906
H	-7.415477633	0.5293091012	-2.1245502874
H	-7.2368426289	0.8333924597	-0.3770281294
O	-2.8831973255	-0.1512132485	-0.7917963593
B	-2.4195437442	-1.1085140964	0.0960418334
C	-2.5316977892	-0.8661676242	1.6486588523
H	-1.7363270629	-0.1356233261	1.8980814888
H	-3.474652484	-0.331522835	1.8668262319
C	-2.3878574071	-2.1104760815	2.5210572342
H	-3.1855106395	-2.8427126705	2.3175038966
H	-1.4282138984	-2.6197588447	2.3393621699
H	-2.4307839943	-1.8718653486	3.5949032551
C	-1.7852793042	-2.3955131222	-0.5410048437
H	-0.7634769221	-2.4678536543	-0.1169544628
H	-2.3050801086	-3.274982596	-0.1158181211
C	-1.7494291765	-2.4519463802	-2.0632176696
H	-1.2824229854	-3.3756020194	-2.438248515
H	-2.7629534975	-2.3956158209	-2.4903581272
H	-1.1827804984	-1.6032907321	-2.4785163382

S36

E(RMN15) = -1559.40547272

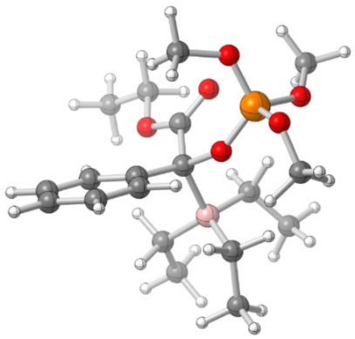

Zero-point correction= 0.519284 (Hartree/Particle)

Thermal correction to Energy= 0.551595

Thermal correction to Enthalpy= 0.552539

Thermal correction to Gibbs Free Energy= 0.457206

Sum of electronic and ZPE= -1558.886189

Sum of electronic and thermal Energies= -1558.853878

Sum of electronic and thermal Enthalpies= -1558.852933

Sum of electronic and thermal Free Energies= -1558.948266

	E, kcal/mol	CV, cal/(mol·K)	S, cal/(mol·K)
Total	346.131	119.822	200.645

Charge = 0 Multiplicity = 1

C	-1.0822218257	0.8006014226	0.2935763888
O	-0.7280339712	2.2038076218	0.1259608803
C	-2.4794154522	0.633631235	-0.2580112502
C	-3.017122135	-0.6253303498	-0.5834082223
C	-3.3068218514	1.758843559	-0.4310791369
C	-4.3248626681	-0.748118127	-1.0557205075
H	-2.4087632885	-1.5207886013	-0.4700334232
C	-4.6154398709	1.6322086648	-0.9029615912
H	-2.9216006588	2.7520701624	-0.1935067657
C	-5.1357221496	0.3769768077	-1.2180392179
H	-4.7106827624	-1.7409345532	-1.3001063055
H	-5.2293871843	2.5283163475	-1.022455975
H	-6.1589577825	0.2758619109	-1.5868178288
C	-0.002739165	0.0390296799	-0.3982104743
O	-0.1438270855	-1.2708698	-0.4291234523
C	0.9607396646	-2.0376823823	-0.9314812574
H	1.8565425163	-1.7754572894	-0.3470770176

H	1.1496339258	-1.7445500627	-1.9754033935
C	0.6094184467	-3.4976194916	-0.8044198868
H	-0.2887505466	-3.7397566679	-1.391129319
H	1.4404642293	-4.1125272283	-1.1777007951
H	0.4257271274	-3.7658538097	0.2462000421
O	0.9862274834	0.6007065717	-0.8541706813
P	0.3221938226	3.0796130082	-0.5913465615
O	1.7513088051	3.1549799526	0.0108951187
O	0.4382951304	2.9364953359	-2.1412749851
O	-0.2591908069	4.525311836	-0.3750048194
C	-0.7327810011	4.9925042142	0.8926065899
H	0.0414907338	4.8702447715	1.6642905059
H	-0.9629765232	6.0574348303	0.7753594655
H	-1.6397163366	4.4452290251	1.1863719445
C	2.9519871604	2.4537424721	-0.3386907539
H	3.7815803498	3.1406189533	-0.1348104059
H	3.0415501091	1.5516211463	0.2773644397
H	2.9357350932	2.1796087575	-1.4001943718
C	-0.6405922432	2.4511345172	-2.9522444004
H	-0.8192845462	1.3851308557	-2.7499230794
H	-1.5572860231	3.0294951727	-2.7673226544
H	-0.3343167944	2.5777123463	-3.9963133868
B	-0.9376921002	0.4855924509	2.0221264898
C	-1.6999944417	-0.9321434949	2.3374096787
H	-2.7978128142	-0.7739223718	2.3410952757
H	-1.5189340958	-1.6409399366	1.5104446051
C	-1.6611541527	1.7852730137	2.726261049

H	-0.9599879711	2.6436827945	2.7251411599
H	-2.5138167071	2.1170743314	2.0999082157
C	0.6846461143	0.4521841348	2.3100505471
H	1.1185424111	-0.5145328916	1.9732831408
H	1.1691471123	1.2177474461	1.6718842087
C	-1.2953310687	-1.6543573506	3.6253881757
H	-1.858507371	-2.5916550503	3.7796989593
H	-0.2267634997	-1.9282318646	3.6073629392
H	-1.4485874161	-1.03912377	4.5262436652
C	-2.1919574197	1.5838681647	4.146794787
H	-2.9452970656	0.7793850489	4.182772299
H	-1.3956261402	1.3029770595	4.8551404469
H	-2.67563277	2.4896011887	4.5533912107
C	1.1331260226	0.7279435955	3.74665427
H	0.7113201301	0.009810576	4.4670007582
H	2.2303793957	0.694502984	3.8657527473
H	0.8108998121	1.7304837072	4.0741795753

S37

E(RMN15) = -1559.39443512

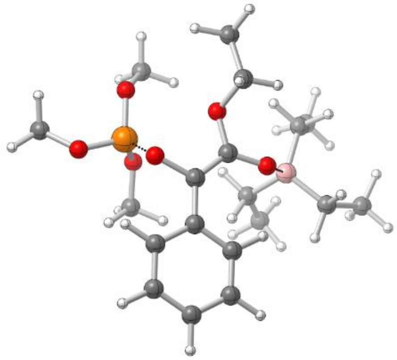

Zero-point correction= 0.518117 (Hartree/Particle)

Thermal correction to Energy= 0.550870

Thermal correction to Enthalpy= 0.551814

Thermal correction to Gibbs Free Energy= 0.454013

Sum of electronic and ZPE= -1558.876318

Sum of electronic and thermal Energies= -1558.843565

Sum of electronic and thermal Enthalpies= -1558.842621

Sum of electronic and thermal Free Energies= -1558.940422

	E, kcal/mol	CV, cal/(mol·K)	S, cal/(mol·K)
Total	345.676	120.277	205.840

Charge = 0 Multiplicity = 1

C	-0.8002433295	0.8550911033	-0.4481132418
O	-0.0126652281	2.0245579739	-0.2135170579
C	-2.2141184897	1.0228625413	-0.1246483245
C	-3.133210977	-0.0474337634	-0.1992107332
C	-2.7036339224	2.2844642156	0.2746298498
C	-4.4794064838	0.1504733905	0.1016328805
H	-2.7759307998	-1.0345342802	-0.4920860509
C	-4.0537576666	2.4709187883	0.5747605263
H	-2.017787486	3.130331715	0.3591144985
C	-4.9539768339	1.4077678841	0.488003823
H	-5.1675161711	-0.6960190766	0.0344849172
H	-4.4010148738	3.4606177991	0.8820871879
H	-6.0107462452	1.5544994686	0.7220184128
C	-0.1194089063	-0.2432984345	-0.901767012
O	1.2055579942	-0.0425625977	-1.1603416937
C	2.0861755346	-0.9091185169	-0.4357114153
H	1.8057312072	-1.9545366495	-0.6346048935
H	1.9482284224	-0.721029629	0.6437146442
C	3.5077350915	-0.6333389912	-0.8538098036

H	3.7875809408	0.4074645364	-0.6335664718
H	4.1909183335	-1.2957367608	-0.30302103
H	3.6443995156	-0.8192156242	-1.9302280695
O	-0.6183073992	-1.4154200244	-1.0790433664
P	0.6290475817	2.8837399806	-1.3370106113
O	0.42802235	4.330628229	-0.7877384091
O	2.1510098679	2.6931968128	-1.567779887
O	-0.0351190715	2.6551625023	-2.7268944884
C	-1.4349472856	2.8599387086	-3.0200167145
H	-1.8690924794	3.6018132329	-2.3343797524
H	-1.4921300127	3.2293360467	-4.0497660688
H	-1.9592849456	1.9011323519	-2.9305650948
C	1.1521875382	5.4631177441	-1.3036070906
H	0.6884066114	6.3553413653	-0.8705754204
H	2.2042633354	5.4024208096	-0.996886215
H	1.0767653308	5.5062425478	-2.3995452309
C	2.7644753204	2.1284443556	-2.7468832872
H	3.8317689558	2.0300696854	-2.5244513841
H	2.3283921605	1.1461091222	-2.9635521552
H	2.6213446004	2.8126357227	-3.5930633187
B	-0.847110618	-2.082208919	-2.5357592168
C	-1.4163242418	-0.8546953602	-3.4576209485
H	-2.090316005	-0.2642314405	-2.8044403279
H	-0.5910597923	-0.159939533	-3.7289313902
C	0.596425065	-2.6750517247	-3.029496021
H	0.8611807909	-3.5710219241	-2.4293194208
H	1.3709911988	-1.9221373726	-2.7915865751

C	-1.9653882812	-3.2255138277	-2.1965100916
H	-1.7192386963	-3.6225279665	-1.1916685225
H	-2.9610011948	-2.7484543002	-2.0801375726
C	-2.2096195231	-1.2380240525	-4.7069476933
H	-3.1028645414	-1.8261575242	-4.4371043136
H	-2.5688878658	-0.3622623027	-5.2757999356
H	-1.6261012658	-1.8563042342	-5.4076079541
C	-2.0856342088	-4.4058578155	-3.1602131046
H	-1.1365421277	-4.9641084879	-3.2270237127
H	-2.8593835665	-5.1322581036	-2.8559143379
H	-2.3360012465	-4.0858432338	-4.1855596024
C	0.7342298904	-3.001600526	-4.5184146821
H	0.6243798298	-2.0916597173	-5.1321562716
H	1.7172969652	-3.4356741776	-4.7725598192
H	-0.0286876499	-3.7136877411	-4.8717039343

Transition State Structures

TS-A

E(RMN15) = -1297.36988244

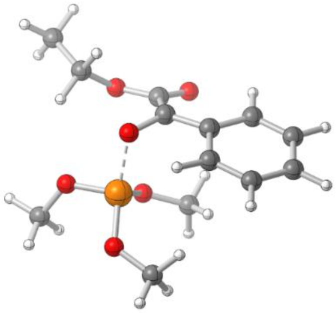

Zero-point correction= 0.313527 (Hartree/Particle)

Thermal correction to Energy= 0.335421

Thermal correction to Enthalpy= 0.336365

Thermal correction to Gibbs Free Energy= 0.262169

Sum of electronic and ZPE= -1297.056355

Sum of electronic and thermal Energies= -1297.034461

Sum of electronic and thermal Enthalpies= -1297.033517

Sum of electronic and thermal Free Energies= -1297.107714

	E, kcal/mol	CV, cal/(mol·K)	S, cal/(mol·K)
Total	210.480	78.819	156.159

Charge = 0 Multiplicity = 1

C	-0.6504442354	0.4625592642	-0.7200632602
O	-0.0051838245	1.2765494343	0.0978355051
C	-2.0842894843	0.618712402	-0.7409597168
C	-2.9651320837	-0.0489414581	-1.6363288433
C	-2.6347352437	1.6189507673	0.1031593698
C	-4.3036792353	0.3013848966	-1.6993326248
H	-2.5666103342	-0.8218637704	-2.2912001153
C	-3.9929610332	1.9648319414	0.0211697546
H	-2.0108888044	2.073111736	0.8737608344
C	-4.829429452	1.3201281588	-0.8810079775
H	-4.9604809641	-0.2206625119	-2.3999656683
H	-4.391477895	2.7337370058	0.6885845425
H	-5.8877535002	1.5835822276	-0.9430070468
C	0.1175360793	-0.1374393664	-1.8223936421
O	1.4591315006	-0.1640489634	-1.6921315753
C	2.1418299159	-0.3203287395	-0.4467830561
H	1.4746189375	-0.809301934	0.2810429316
H	2.4013003619	0.6682230957	-0.0431522135
C	3.3776774542	-1.1544884946	-0.6942213903
H	4.0332799829	-0.669821115	-1.4327524765

H	3.9441944004	-1.2749958003	0.2410291615
H	3.1115593815	-2.1531776431	-1.0704419085
O	-0.3734759111	-0.5904099198	-2.8435301737
P	0.1868842665	2.9942953115	-0.6304806561
O	1.7491669032	3.0470999915	-0.3788399392
O	0.0706972108	2.7146170856	-2.2000887804
O	-0.1482128682	4.567431652	-0.4228557296
C	-1.4407371384	5.1325504446	-0.600778397
H	-1.5942363995	5.4178395694	-1.6525671652
H	-2.2315556935	4.4250857663	-0.3001740864
H	-1.5045954193	6.0297964193	0.0275258894
C	2.5691212695	4.0948955062	-0.8992931933
H	2.4398558737	5.013545862	-0.3116848862
H	3.6088658983	3.7544421054	-0.8284993829
H	2.3231665277	4.2923303543	-1.953189239
C	-1.124635379	2.7219267151	-2.9861903606
H	-1.0235424424	3.4864016552	-3.7686723733
H	-1.2385989906	1.7295340942	-3.443278442
H	-2.0159096316	2.9347462554	-2.3750436687

TS-B

E(RMN15) = -1297.37931570

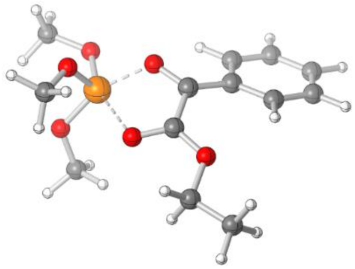

Zero-point correction= 0.312747 (Hartree/Particle)

Thermal correction to Energy= 0.335235

Thermal correction to Enthalpy= 0.336180

Thermal correction to Gibbs Free Energy= 0.258660

Sum of electronic and ZPE= -1297.066569

Sum of electronic and thermal Energies= -1297.044080

Sum of electronic and thermal Enthalpies= -1297.043136

Sum of electronic and thermal Free Energies= -1297.120656

	E, kcal/mol	CV, cal/(mol·K)	S, cal/(mol·K)
Total	210.363	79.034	163.154

Charge = 0 Multiplicity = 1

C	0.3118047239	2.2301522008	0.4831690695
C	-0.6519514572	1.7948008487	1.4784141048
O	-1.7026870649	1.2751627139	1.0779112166
O	-0.1553297591	2.145151853	-0.7122211139
O	-0.301074085	1.8052550236	2.761081873
C	-1.224697374	1.2314338917	3.6924136655
H	-2.2080827677	1.7090083067	3.5585723931
H	-1.3478127564	0.1618713006	3.4598278623
C	-0.6806770597	1.4417130803	5.0827656474
H	-0.5731136371	2.5135378375	5.3040706006
H	-1.3673605559	1.0043835588	5.8212161446
H	0.3012895907	0.9600291923	5.1966011339
C	1.7675669537	2.2498343637	0.6810567483
C	2.5850677672	1.9141958202	-0.4178200481
C	2.385551368	2.6077041034	1.8959165411
C	3.9726430189	1.9125739315	-0.2991708498
H	2.1060972494	1.6509202989	-1.3633886052

C	3.7759110109	2.6136565782	2.0031129508
H	1.7743471768	2.8909109691	2.7523578081
C	4.5765898155	2.2626083531	0.9125025653
H	4.5884367102	1.6390294665	-1.1594556363
H	4.2395356962	2.8991228375	2.950574036
H	5.6651156751	2.2675161	1.004629681
P	-1.3306376538	0.7456097506	-1.3256274207
O	-2.5652716722	1.7430463128	-1.5775962443
O	-0.7013201427	0.745907327	-2.8103028975
O	-2.1907613365	-0.6616204704	-1.3028716163
C	-3.7732746642	1.7487235305	-0.8434990909
H	-4.5948691463	1.9550490096	-1.5446122152
H	-3.954663807	0.7773736277	-0.3617220484
H	-3.7537370758	2.5320626463	-0.0728473365
C	-1.7544623249	-1.7341908265	-0.4885212934
H	-0.8697324384	-2.2348734026	-0.9145030308
H	-1.5103626548	-1.3867684236	0.5288778786
H	-2.5718649857	-2.4649454605	-0.423819807
C	-1.5194724636	0.5617651725	-3.9564244756
H	-2.0890626306	1.4756831824	-4.1814071259
H	-0.8610449533	0.3300184105	-4.8038664371
H	-2.2225822897	-0.2733130162	-3.8129346273

TS-C

E(RMN15) = -1297.40933907

Zero-point correction= 0.313065 (Hartree/Particle)

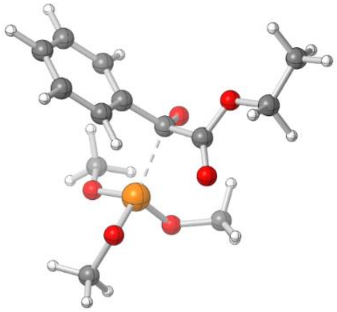

Thermal correction to Energy= 0.335751

Thermal correction to Enthalpy= 0.336695

Thermal correction to Gibbs Free Energy= 0.258483

Sum of electronic and ZPE= -1297.096274

Sum of electronic and thermal Energies= -1297.073588

Sum of electronic and thermal Enthalpies= -1297.072644

Sum of electronic and thermal Free Energies= -1297.150856

	E, kcal/mol	CV, cal/(mol·K)	S, cal/(mol·K)
Total	210.687	79.324	164.610

Charge = 0 Multiplicity = 1

C	-1.4502385275	-0.7888372156	-2.4006419863
O	-1.3263034687	0.0455596449	-3.3310185072
C	-2.1997078241	-0.4049721792	-1.1320319126
C	-2.4551464252	-1.335377818	-0.1159278834
C	-2.628344368	0.9163809558	-0.9953471484
C	-3.1321783033	-0.9382462308	1.0365174171
H	-2.136255905	-2.3758671794	-0.2412901546
C	-3.3097757094	1.3124168885	0.158297593
H	-2.4078933433	1.6152373523	-1.8059479317
C	-3.5605492087	0.3869785014	1.1742538375
H	-3.3318574965	-1.6636238272	1.8285841405
H	-3.6439778434	2.3470595363	0.2671733299
H	-4.0931518519	0.6964634264	2.0766401141
C	-0.2584976995	-1.7236580322	-2.0938972794

O	0.7954275518	-1.0138770964	-1.7336479969
C	1.9952745084	-1.7407493904	-1.4286135415
H	2.2769450636	-2.3364553842	-2.3107701863
H	1.7796258557	-2.4475386535	-0.6123790514
C	3.0644598111	-0.7484545735	-1.0500156668
H	2.7638276591	-0.1653238837	-0.1677211393
H	3.9964402499	-1.2802096541	-0.8115849465
H	3.2649746598	-0.0528911949	-1.8775904241
O	-0.267576218	-2.9351647854	-2.1747991664
P	-2.8072871391	-2.2738488186	-3.2310046431
O	-3.3559004666	-3.6648059782	-2.6719116969
O	-4.1963648968	-1.4969361804	-3.4549709611
O	-2.3755554192	-2.5282059662	-4.762024675
C	-1.0172627461	-2.5617388422	-5.1968574494
H	-0.4973130544	-3.4473980167	-4.8057932514
H	-1.0312261021	-2.6080054502	-6.293332091
H	-0.4933879853	-1.6465759767	-4.8828666792
C	-4.4459082778	-4.3463433375	-3.2947955966
H	-4.340886642	-4.3311138128	-4.390089325
H	-4.4272856359	-5.3859088903	-2.9451760464
H	-5.4009216157	-3.8811308555	-3.0137618178
C	-4.2960801698	-0.2752854701	-4.1772817693
H	-5.3116077908	-0.2150940866	-4.591592857
H	-4.1239995101	0.5821839241	-3.5112946653
H	-3.5654897372	-0.2416879389	-4.9983919366

TS-D

E(RMN15) = -1297.40504587

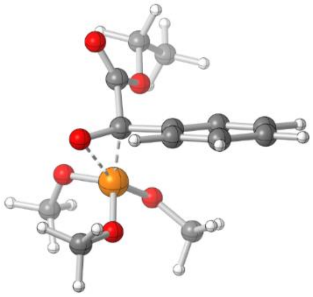

Zero-point correction= 0.313639 (Hartree/Particle)

Thermal correction to Energy= 0.336049

Thermal correction to Enthalpy= 0.336993

Thermal correction to Gibbs Free Energy= 0.259923

Sum of electronic and ZPE= -1297.091407

Sum of electronic and thermal Energies= -1297.068997

Sum of electronic and thermal Enthalpies= -1297.068053

Sum of electronic and thermal Free Energies= -1297.145122

	E, kcal/mol	CV, cal/(mol·K)	S, cal/(mol·K)
Total	210.874	78.666	162.207

Charge = 0 Multiplicity = 1

C	-1.2897024306	2.7384004866	0.1961487711
O	-0.8166046115	2.2932051322	-0.9684116266
C	-0.3744235276	2.5889948658	1.4238609381
C	-0.6571432266	3.1625247585	2.6717592075
C	0.8022667892	1.8610682869	1.2407177125
C	0.2417455319	3.0026998939	3.7281512745
H	-1.5763662591	3.739208905	2.8079179071
C	1.6957496667	1.6945171824	2.3019729821
H	0.9751342915	1.4399494364	0.2468452499
C	1.4178036638	2.2669259254	3.5457541028
H	0.0240545416	3.4532509493	4.6996691549

H	2.6137190076	1.1188222291	2.1587626418
H	2.118156051	2.1426407557	4.3752140209
C	-2.705190357	2.2537720439	0.5621031229
O	-3.4064928751	3.195451259	1.2054405632
C	-4.7393240715	2.8516393713	1.6025244569
H	-4.6963312455	1.9678109442	2.2579308534
H	-5.3096744051	2.5645328313	0.7055675305
C	-5.346007836	4.0427117402	2.2997638149
H	-5.366651189	4.9172179378	1.6332630838
H	-6.3777238903	3.813451757	2.6019048146
H	-4.7733231475	4.3047047684	3.2015738018
O	-3.1351960417	1.1616368967	0.2962376059
P	-1.2852060127	4.4385441445	-0.3612394091
O	-2.3584821677	4.5397360109	-1.5139934751
O	-1.6384601231	5.6803121318	0.5762398499
O	0.153578215	4.8638057763	-0.852231011
C	0.9842403281	4.2159333708	-1.8180506558
H	1.5583555723	3.4135295818	-1.3398968224
H	0.3768836758	3.7850383281	-2.6226108004
H	1.6612012769	4.9859110382	-2.2111536173
C	-2.6450441584	5.7963073677	-2.1318125789
H	-3.1752914653	5.5832914203	-3.0672300738
H	-3.2793954873	6.4102828491	-1.4777564168
H	-1.7166492594	6.3417070831	-2.3605272341
C	-0.6793436371	6.4426354497	1.3073104626
H	0.0958056421	5.8004155833	1.7501106033
H	-0.2019915802	7.1835532293	0.6504909618

H -1.2179197295 6.9611433509 2.1101248734

TS-E

E(RMN15) = -798.417996253

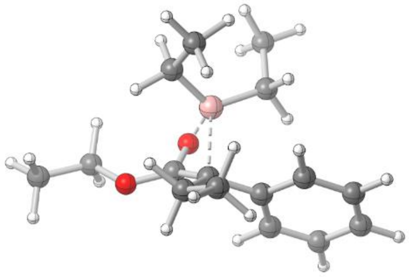

Zero-point correction= 0.381790 (Hartree/Particle)

Thermal correction to Energy= 0.402387

Thermal correction to Enthalpy= 0.403331

Thermal correction to Gibbs Free Energy= 0.333106

Sum of electronic and ZPE= -798.036206

Sum of electronic and thermal Energies= -798.015609

Sum of electronic and thermal Enthalpies= -798.014665

Sum of electronic and thermal Free Energies= -798.084890

	E, kcal/mol	CV, cal/(mol·K)	S, cal/(mol·K)
Total	252.502	78.426	147.801

Charge = 0 Multiplicity = 1

C 0.0903081389 0.5279520308 0.6724610131

C -1.3305629758 0.1027464406 0.8409529023

C -1.6980760605 -0.9593173784 1.6870106304

C -2.351977937 0.7674515678 0.1398056027

C -3.0315694524 -1.3463981454 1.8168519051

H -0.9236191644 -1.5176284931 2.2164264331

C -3.6851794489 0.3684073998 0.2531310181

H -2.0929334886 1.6097691441 -0.5076247766

C	-4.0327422978	-0.6893426161	1.0957690963
H	-3.2890812493	-2.1802272015	2.4745696153
H	-4.4572718132	0.8970855287	-0.3113300168
H	-5.0758537517	-0.9994536922	1.1917921371
C	0.9058567168	1.0339757937	1.8569097793
H	1.970278438	0.9720624376	1.5711040798
H	0.7024087515	2.1135050237	1.9777763963
C	0.7064598386	0.3503840415	3.2019457567
H	-0.3243196984	0.4522220978	3.5716633592
H	0.9469606121	-0.723531587	3.1628234501
H	1.3707009903	0.8090437895	3.9496415931
B	0.6013340091	-0.9150437419	-0.8297934837
C	-0.4069741894	-2.1344427598	-0.9331255314
H	-1.4407860972	-1.7724673061	-1.0590146988
H	-0.3974312894	-2.7335589167	-0.0061828718
C	-0.0127864755	-3.0221876205	-2.1199883668
H	0.9873381661	-3.4642483741	-1.9805022649
H	-0.7184740765	-3.8550330837	-2.2674439413
H	0.0153443606	-2.4497312882	-3.0618016813
C	2.1662953191	-1.1785283342	-0.6715792018
H	2.5438339364	-1.5751084764	-1.634836922
H	2.7205544305	-0.2358397512	-0.5075155827
C	2.4848311604	-2.1640084743	0.4513035797
H	2.094471507	-1.7988430985	1.4171107247
H	2.0168146534	-3.1450446383	0.2685624949
H	3.5653067914	-2.3367716818	0.576781538
C	0.4972419859	1.0273866334	-0.5691249941

O	1.3693325699	2.0080291354	-0.6688091743
O	0.2304133537	0.2992286796	-1.6336437104
C	2.0420260574	2.2260004641	-1.9227722953
H	2.447908558	1.2674027467	-2.2799617683
H	1.3035147704	2.5733551923	-2.6606008471
C	3.1265284738	3.2472210137	-1.6942827442
H	3.6486069162	3.448771669	-2.6402009841
H	2.705343655	4.192845724	-1.323914916
H	3.8623793051	2.8805611015	-0.9640083248

TS-F

E(RMN15) = -1297.41216262

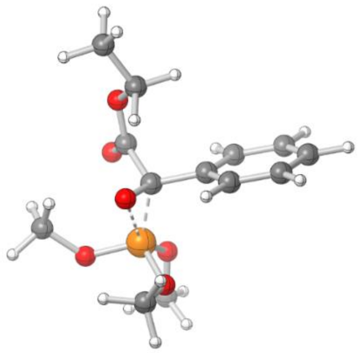

Zero-point correction= 0.314335 (Hartree/Particle)

Thermal correction to Energy= 0.336342

Thermal correction to Enthalpy= 0.337286

Thermal correction to Gibbs Free Energy= 0.262543

Sum of electronic and ZPE= -1297.097828

Sum of electronic and thermal Energies= -1297.075820

Sum of electronic and thermal Enthalpies= -1297.074876

Sum of electronic and thermal Free Energies= -1297.149620

	E, kcal/mol	CV, cal/(mol·K)	S, cal/(mol·K)
Total	211.058	78.401	157.311

Charge = 0 Multiplicity = 1

C	-1.5541480852	-0.952116576	-2.1701013549
O	-1.4675735582	0.0311758025	-3.114202903
C	-2.5527816444	-0.755187523	-1.0354942623
C	-2.657353423	-1.6803420798	0.0108485074
C	-3.3973786982	0.3549298841	-1.0799723145
C	-3.5972843098	-1.479293387	1.022142265
H	-2.0061548959	-2.5601292029	0.0269664718
C	-4.3376074833	0.553928928	-0.0658550187
H	-3.2830870347	1.0497696075	-1.9163098716
C	-4.4375312666	-0.3612260411	0.9854293044
H	-3.6774683398	-2.1976031292	1.841460336
H	-4.9959175131	1.4257393425	-0.0948995796
H	-5.1728897019	-0.2054775107	1.7783562339
C	-0.2074383773	-1.4643382787	-1.6374777414
O	0.5700401912	-0.6103849302	-0.9892780907
C	0.1468752662	0.7271125027	-0.6714262148
H	-0.4485424772	0.6892375827	0.2549145226
H	-0.4874071158	1.1022243395	-1.4873444754
C	1.3824539852	1.5729786161	-0.4881522684
H	1.9656747915	1.6204148823	-1.4193105544
H	1.0938222255	2.5963759068	-0.2087660956
H	2.0246190139	1.1663891584	0.3067465957
O	0.1590265135	-2.6043678412	-1.8387240209
P	-2.1831928781	-2.042409689	-3.4237684508
O	-2.6534704446	-3.468375357	-2.91785762
O	-3.5260967374	-1.4795321698	-4.0351663702
O	-1.1969265752	-2.3051341196	-4.622770249

C	0.1177354263	-1.7871245691	-4.8440077788
H	0.8599832336	-2.4872014063	-4.4399735447
H	0.2501710458	-1.6910349915	-5.929083632
H	0.2063074586	-0.8036255814	-4.363884021
C	-3.2842322133	-4.4204336407	-3.7731837393
H	-3.4015208329	-5.3489771888	-3.2022059807
H	-4.2722665619	-4.0568011166	-4.0896413099
H	-2.662045976	-4.6157719794	-4.6591455354
C	-3.6031974843	-0.3711501933	-4.9287846549
H	-4.4799093062	-0.5350266299	-5.5686964386
H	-3.7136532355	0.5621455191	-4.3637201354
H	-2.6977599864	-0.3086299406	-5.548404015

TS-G

E(RMN15) = -1559.38502859

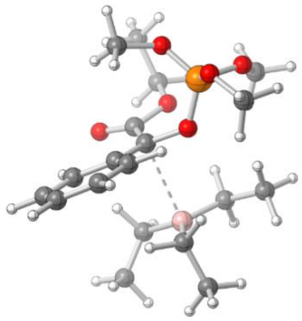

Zero-point correction= 0.516429 (Hartree/Particle)

Thermal correction to Energy= 0.549411

Thermal correction to Enthalpy= 0.550355

Thermal correction to Gibbs Free Energy= 0.452431

Sum of electronic and ZPE= -1558.868600

Sum of electronic and thermal Energies= -1558.835618

Sum of electronic and thermal Enthalpies= -1558.834674

Sum of electronic and thermal Free Energies= -1558.932597

E, kcal/mol	CV, cal/(mol·K)	S, cal/(mol·K)

Total	344.760	119.979	206.097

Charge = 0 Multiplicity = 1

C	-0.799340171	0.921638414	-0.259393925
O	-0.240980256	2.216833937	0.027628249
C	-2.233983714	0.891302409	-0.405174383
C	-2.936050104	-0.310800879	-0.681911013
C	-3.011992112	2.065544124	-0.247848962
C	-4.326700372	-0.325618983	-0.769732811
H	-2.364899168	-1.226207085	-0.832897901
C	-4.402898551	2.036089374	-0.343045754
H	-2.518420129	3.014704768	-0.029686435
C	-5.080072585	0.841123983	-0.599125271
H	-4.831868554	-1.272819111	-0.978996648
H	-4.964008778	2.965200404	-0.209853284
H	-6.169871715	0.819136459	-0.66806517
C	0.120681826	-0.030391879	-0.759988202
O	1.4162564	0.435550337	-0.737869494
C	2.402499521	-0.375998852	-1.362918752
H	2.178382867	-1.433203713	-1.161799795
H	3.354791757	-0.122765824	-0.873005113
C	2.460521182	-0.110897064	-2.852463138
H	2.683626522	0.947512055	-3.056825537
H	3.238852167	-0.72866148	-3.324911605
H	1.494568429	-0.361606731	-3.317092138
O	-0.133006167	-1.159752571	-1.182536059
P	0.371318431	3.169963005	-1.036512441

O	1.783361512	3.639000578	-0.603593911
O	0.42092276	2.588188847	-2.471849103
O	-0.483880792	4.477538475	-1.114523567
C	-0.748152206	5.286083085	0.04489377
H	0.158683635	5.839203804	0.32481506
H	-1.543970074	5.987300317	-0.227718193
H	-1.081455865	4.659100404	0.885995999
C	2.891967843	2.883354877	-0.087332588
H	2.541173367	2.150193863	0.648163463
H	3.404651633	2.375638895	-0.914591412
H	3.565954613	3.609342999	0.380208366
C	-0.699520956	2.110427932	-3.245488826
H	-0.729674342	1.01435319	-3.190033496
H	-1.642811205	2.529072901	-2.866935896
H	-0.53641076	2.43686189	-4.278904111
B	-0.644941684	0.174650458	2.4025066
C	-0.762268439	-1.387396848	2.123748517
H	-1.029803621	-1.488584992	1.055439264
H	0.226632642	-1.875087306	2.209606202
C	-1.925126542	1.026180059	2.790422727
H	-1.804151219	2.072327917	2.451260852
H	-2.846141311	0.644640212	2.316311022
C	0.806920008	0.769700305	2.664460508
H	1.206856509	0.147005779	3.492557935
H	1.440096464	0.488321809	1.800597817
C	-1.800119642	-2.149362952	2.950878126
H	-1.894667459	-3.199701281	2.630543215

H	-1.54947418	-2.163492287	4.024375087
H	-2.799642627	-1.69329116	2.854997452
C	-2.09905345	1.00229167	4.318451027
H	-2.323808351	-0.012205273	4.681925148
H	-1.187978993	1.332720634	4.843617869
H	-2.921795269	1.654704491	4.65323452
C	0.976581644	2.248144917	3.008585711
H	0.383593598	2.531145907	3.893096666
H	2.024553652	2.510576806	3.230683801
H	0.645966586	2.89929359	2.184604751

TS-H

E(RMN15) = -1559.37548501

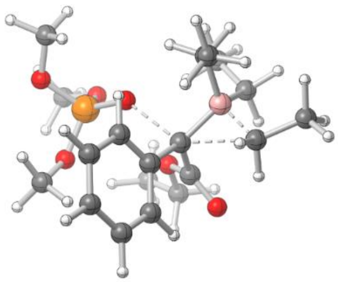

Zero-point correction= 0.516302 (Hartree/Particle)

Thermal correction to Energy= 0.549260

Thermal correction to Enthalpy= 0.550204

Thermal correction to Gibbs Free Energy= 0.451921

Sum of electronic and ZPE= -1558.859183

Sum of electronic and thermal Energies= -1558.826225

Sum of electronic and thermal Enthalpies= -1558.825281

Sum of electronic and thermal Free Energies= -1558.923564

	E, kcal/mol	CV, cal/(mol·K)	S, cal/(mol·K)
Total	344.666	119.828	206.854

Charge = 0 Multiplicity = 1

C	-0.4567750767	-0.9474983908	0.2939417673
O	0.0234259527	1.0814167997	-0.1955545947
C	-1.3296137973	-1.1386027306	-0.8887021904
C	-1.2257736139	-2.2974042	-1.6804654203
C	-2.2898215622	-0.1678315511	-1.2342011498
C	-2.0614800781	-2.4794617156	-2.7849519015
H	-0.4996981725	-3.0685074074	-1.4202410599
C	-3.0973323094	-0.3387620779	-2.3548259943
H	-2.3591649315	0.7375605073	-0.632086478
C	-2.9884960423	-1.4970326661	-3.1334560883
H	-1.9762173633	-3.3912708286	-3.3801459643
H	-3.8210669568	0.4343827449	-2.6227069686
H	-3.6290757297	-1.6315951324	-4.0080899242
C	0.9015391438	-1.5997392875	0.1776430472
O	1.9235900038	-0.7808260481	-0.012467622
C	3.2198964291	-1.3939206832	-0.0846657286
H	3.1850088513	-2.2020537995	-0.830824358
H	3.4405303759	-1.8574358236	0.8896793439
C	4.2252242637	-0.3332246443	-0.4522144488
H	5.2215261413	-0.788962405	-0.5435401233
H	3.9597763463	0.1266800241	-1.4154067503
H	4.2686294445	0.4536665427	0.3140683901
O	1.0307558842	-2.8029446167	0.2779713558
P	0.9435306489	1.7524608989	-1.153294867
O	1.4244223873	0.9609867918	-2.4342559157
O	0.2811177918	3.0546911092	-1.7913031604

O	2.2913953965	2.209098902	-0.4730519214
C	3.2713116587	2.9733810313	-1.1718422562
H	3.5175943079	2.5062469549	-2.136874225
H	2.907648191	3.9966913332	-1.3459245253
H	4.1700655938	3.0093530669	-0.5447247294
C	0.5231346025	0.5728029761	-3.4737737246
H	1.1006214426	0.5101606996	-4.4041201176
H	0.0838380409	-0.4102378314	-3.2509793717
H	-0.2766564669	1.3179397712	-3.5969572781
C	-0.6755261537	3.8134768087	-1.0505756912
H	-0.9314156076	4.6927669561	-1.6535116325
H	-1.5830698356	3.2200499893	-0.8654047163
H	-0.2578146856	4.1455957661	-0.0883172504
B	-0.9621602305	-0.7770893304	1.7964210203
C	-1.6110446739	-2.3505548969	1.8972009452
H	-2.5477625543	-2.3637794063	1.3134956921
H	-0.9681019593	-3.1296832337	1.4602527725
C	-2.1398020323	0.3413607981	2.0686838571
H	-2.0185806189	0.6578422055	3.1239508518
H	-1.8988703423	1.2481040203	1.4831038378
C	0.2850514968	-0.5994032085	2.8441706934
H	-0.1263898039	-0.7477359823	3.8608575677
H	1.0369839222	-1.4078341414	2.7285948401
C	-1.8880115079	-2.6959156958	3.3586874366
H	-2.4227804209	-1.8858806022	3.8828406981
H	-2.5007708459	-3.6094653982	3.4503797265
H	-0.955239934	-2.8764767046	3.9144777682

Go back to table of contents

C -3.6104654956 -0.0309820027 1.8703165244

H -3.9166276061 -0.8438095879 2.5488748914

H -4.2875627771 0.8177713598 2.0689899167

H -3.830749841 -0.3837285054 0.8502274597

C 0.9895205933 0.7579485928 2.8088598174

H 1.7421744385 0.8692933858 3.6092183641

H 1.4923827068 0.9292983521 1.8462548019

H 0.2652342906 1.581323188 2.9325733413

TS-I

E(RMN15) = -1559.39714389

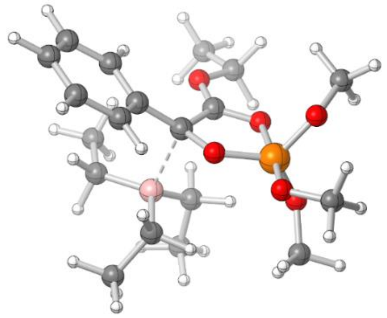

Zero-point correction= 0.518886 (Hartree/Particle)

Thermal correction to Energy= 0.550544

Thermal correction to Enthalpy= 0.551488

Thermal correction to Gibbs Free Energy= 0.457284

Sum of electronic and ZPE= -1558.878258

Sum of electronic and thermal Energies= -1558.846600

Sum of electronic and thermal Enthalpies= -1558.845656

Sum of electronic and thermal Free Energies= -1558.939860

	E, kcal/mol	CV, cal/(mol·K)	S, cal/(mol·K)
Total	345.471	118.031	198.268

Charge = 0 Multiplicity = 1

C 0.9890547642 1.2593807407 0.6945893405

C	-0.1622958539	1.826318082	1.2637837338
O	-1.2492818767	1.6188194094	0.6073438871
O	0.5904083167	0.7902696361	-0.5825849063
O	-0.194647071	2.4899321173	2.3853321979
C	-1.473075419	2.8141563083	2.9696838356
H	-1.9147355852	3.6434047121	2.3957454412
H	-2.1326638896	1.9399451723	2.8714256248
C	-1.2363263107	3.180853247	4.41139002
H	-0.5450958357	4.0320141528	4.4943600481
H	-2.1894557355	3.4625069036	4.8805787135
H	-0.8155059642	2.3281770142	4.9648677702
C	2.3455714402	1.8383930939	0.70251049
C	2.7774688244	2.7477032999	1.6864116169
C	3.2565078494	1.4584000184	-0.2988051114
C	4.0843447715	3.2332682297	1.6794127231
H	2.0863484329	3.0797212653	2.461025892
C	4.5639930326	1.9489051931	-0.2994257691
H	2.9278269886	0.7800780869	-1.0879607016
C	4.9902063117	2.8333119019	0.6924923206
H	4.3962788372	3.9371353968	2.4550471793
H	5.2528646904	1.6356760886	-1.0878888399
H	6.0140097304	3.2140435622	0.6935952048
P	-0.9509281589	0.7993618329	-1.0235950338
O	-1.9933760905	-0.2943473465	-0.5042569947
O	-1.6613245847	2.0415646846	-1.7311716669
O	-0.531619387	0.1040373079	-2.4306826283
C	-2.0726703447	-1.6625401547	-0.8845545346

H	-3.0509842517	-1.8500393573	-1.3492099312
H	-1.2741818689	-1.940581265	-1.5847951302
H	-1.9843791146	-2.2747852417	0.0249060941
C	-1.4474843247	0.0096879448	-3.5017889423
H	-2.4828744085	-0.1520031272	-3.1563160672
H	-1.4305322037	0.9242727627	-4.113357768
H	-1.1526463155	-0.8444361206	-4.1276506539
C	-2.5690351723	2.9846757521	-1.1780964829
H	-2.0456547032	3.7243292	-0.5573214866
H	-3.0410902419	3.4964606256	-2.0264252646
H	-3.3441855838	2.4889878329	-0.5775759435
B	1.0791167411	-0.5250441567	1.9093126417
C	1.5087967866	-1.5340220999	0.7168016972
H	0.6154893928	-1.8400459135	0.1384507498
H	2.1587281556	-1.0203058336	-0.0120364509
C	-0.4127996674	-0.717361278	2.4958611136
H	-0.6549621911	0.0723084085	3.232601281
H	-1.1774025681	-0.641342701	1.7052599905
C	2.2196897424	-0.2654500551	3.0154981044
H	2.4812682676	-1.2837663276	3.3678648727
H	3.1531906423	0.1009112315	2.5484883244
C	2.26250196	-2.7824513032	1.1921084856
H	2.4431567128	-3.4957643985	0.3703714762
H	3.2486388584	-2.5168067727	1.6052259947
H	1.7252353651	-3.3304082533	1.9832043528
C	-0.5585154713	-2.0812343125	3.1791045558
H	-0.4445006989	-2.9042464871	2.4544968216

Go back to table of contents

H	0.2081188523	-2.2337917049	3.958441244
H	-1.5415532441	-2.2122342964	3.6614172858
C	1.8660686235	0.5688380667	4.2459056111
H	1.047645165	0.0998092106	4.8188957829
H	2.7161759249	0.6804892475	4.9396241295
H	1.523116452	1.5803922323	3.9829042482

TS-J

E(RMN15) = -1559.37738513

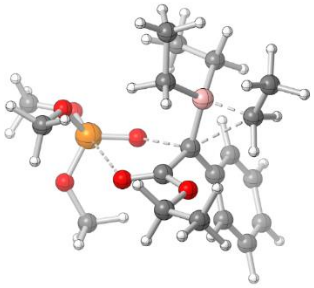

Zero-point correction= 0.516696 (Hartree/Particle)

Thermal correction to Energy= 0.549475

Thermal correction to Enthalpy= 0.550419

Thermal correction to Gibbs Free Energy= 0.452411

Sum of electronic and ZPE= -1558.860690

Sum of electronic and thermal Energies= -1558.827910

Sum of electronic and thermal Enthalpies= -1558.826966

Sum of electronic and thermal Free Energies= -1558.924975

	E, kcal/mol	CV, cal/(mol·K)	S, cal/(mol·K)
Total	344.801	119.690	206.277

Charge = 0 Multiplicity = 1

C	0.618483332	0.3966789448	0.3983251914
C	-0.5612867929	-0.0684800213	1.2316162447
O	-1.4552629571	-0.7952855957	0.8521559279

O	0.1865241522	-0.8513124397	-1.2741002934
O	-0.551701221	0.4650548161	2.4478550281
C	-1.7271124724	0.2738439862	3.2496549543
H	-2.6031070243	0.5783216754	2.6553070587
H	-1.8357427665	-0.7989964488	3.4715628525
C	-1.5750512797	1.1034018154	4.4988044131
H	-1.4650792035	2.1685910874	4.2470580452
H	-2.4657260208	0.9875084621	5.1323473422
H	-0.6954902693	0.7879767042	5.0784077156
C	1.8937264008	-0.2939495282	0.6517717003
C	2.0999318429	-1.0419468021	1.8261888941
C	2.9335791416	-0.2175319762	-0.2948496874
C	3.3254052497	-1.6653137462	2.0647943541
H	1.3070940422	-1.1197155832	2.5731486387
C	4.1481446596	-0.8549657849	-0.0631541242
H	2.7540193622	0.3129394958	-1.2291292651
C	4.3505085489	-1.5720528398	1.1218374438
H	3.4769225996	-2.2292565513	2.9876494575
H	4.9425218068	-0.798252611	-0.8105139018
H	5.3075894551	-2.0662053635	1.3047947176
P	-0.9999048914	-1.5266719063	-1.8699590163
O	-2.3698654692	-0.7540003307	-2.0214120325
O	-0.6628043831	-1.8730092195	-3.3821590939
O	-1.408201652	-2.8970634841	-1.1790981593
C	-3.5052317329	-0.7957912871	-1.1632047391
H	-4.3985182232	-0.7595475655	-1.8003911703
H	-3.5103149908	-1.7183886373	-0.568397518

H	-3.4956581848	0.0704827208	-0.4886794979
C	-0.6580022621	-3.5061047772	-0.1316534754
H	-0.2831076671	-4.4783990073	-0.4798705154
H	0.1896775416	-2.8694272894	0.1623044222
H	-1.3153320439	-3.6541272191	0.7347890237
C	-1.6284769997	-2.4855845822	-4.2283976591
H	-2.4438115649	-1.7846266346	-4.4594856531
H	-1.1204178075	-2.7699986898	-5.1577874013
H	-2.0462588815	-3.3891814904	-3.7578569684
B	0.4297166341	1.8830177487	-0.1487593783
C	1.2279649408	2.56265472	1.2129189173
H	1.1094132052	2.0178961167	2.1607407983
H	2.305189914	2.5148527901	0.9719272391
C	-1.1774119865	2.2229594813	-0.2442615693
H	-1.6473711431	1.3831401503	-0.7906660986
H	-1.6413544996	2.2014072149	0.7639529763
C	1.2465702723	2.389275462	-1.4682045494
H	1.0514945794	3.478028079	-1.5234349722
H	2.3417468187	2.3229924801	-1.3349731738
C	0.7896778909	4.0058281248	1.4366730096
H	1.3662657	4.4801625481	2.249683874
H	0.9249751532	4.6258776425	0.5362012083
H	-0.274041359	4.063690954	1.719706053
C	-1.5854034074	3.5212349615	-0.9422032025
H	-1.1512582338	4.4120581361	-0.462186038
H	-1.2509401576	3.5357293485	-1.9921899696
H	-2.6793966487	3.665276328	-0.9566107797

C	0.8542083461	1.7514264122	-2.8015560219
H	-0.2420602126	1.6715680744	-2.9055916412
H	1.2266278396	2.3189512095	-3.6721313646
H	1.2335550516	0.7219991236	-2.8838412666

6. Single Point Electronic Corrections (MN15 / 6-311G** / SMD (THF))

6.a Stable Minima

S33 E(RMN15) = -948.686315027

S34 E(RMN15) = -1298.63357882

100 E(RMN15) = -1298.64641325

S35 E(RMN15) = -536.936514656

99 E(RMN15) = -1298.61978276

101 E(RMN15) = -1560.90385060

102 E(RMN15) = -799.310616532

103 E(RMN15) = -799.328878147

S36 E(RMN15) = -1560.91316521

S37 E(RMN15) = -1560.89988195

6b. Transition State Structures

TS-A E(RMN15) = -1298.57294368

TS-B E(RMN15) = -1298.58219938

TS-C E(RMN15) = -1298.61710386

TS-D E(RMN15) = -1298.61403509

TS-E E(RMN15) = -799.297161779

TS-F E(RMN15) = -1298.61940369

TS-G E(RMN15) = -1560.89426286

TS-H E(RMN15) = -1560.89069868

TS-I E(RMN15) = -1560.89994332

TS-J E(RMN15) = -1560.89078248

GoodVibes Quasi-harmonic Correction Terms

Stable Minima

Structure	E	ZPE	H	T.S	T.qh-S	G(T)	qh-G(T)
**100**	-1297.45	0.32	-1297.11	0.08	0.07	-1297.18	-1297.18
**101**	-1559.40	0.52	-1558.85	0.10	0.09	-1558.94	-1558.94
**102**	-798.43	0.38	-798.03	0.07	0.07	-798.10	-798.10
**103**	-798.45	0.38	-798.04	0.08	0.07	-798.12	-798.12
**S33**	-947.84	0.33	-947.49	0.07	0.07	-947.56	-947.55
**S34**	-1297.43	0.32	-1297.09	0.08	0.07	-1297.17	-1297.16
**S35**	-536.35	0.18	-536.16	0.05	0.05	-536.21	-536.21
**S36**	-1297.41	0.31	-1297.07	0.08	0.07	-1297.15	-1297.15
**S36**	-1559.41	0.52	-1558.85	0.10	0.09	-1558.95	-1558.95
**S37**	-1559.39	0.52	-1558.84	0.10	0.09	-1558.94	-1558.94

7b. Transition State Structures

Structure	E	ZPE	H	T.S	T.qh-S	G(T)	qh-G(T)
**TS-A**	-1297.41	0.31	-1297.07	0.08	0.08	-1297.15	-1297.15
**TS-B**	-1297.41	0.31	-1297.07	0.08	0.07	-1297.15	-1297.14
**TS-C**	-1559.38	0.52	-1558.83	0.10	0.09	-1558.92	-1558.92
***E*-TS-D**	-798.42	0.38	-798.01	0.07	0.07	-798.08	-798.084
***Z*-TS-D**	-798.42	0.38	-798.01	0.07	0.07	-798.08	-798.081
**TS-E**	-1559.40	0.52	-1558.85	0.09	0.09	-1558.94	-1558.94
**TS-F**	-1297.37	0.31	-1297.03	0.07	0.07	-1297.11	-1297.11
**TS-G**	-1297.38	0.31	-1297.04	0.08	0.07	-1297.12	-1297.12
**TS-H**	-1297.41	0.31	-1297.07	0.07	0.07	-1297.15	-1297.15
**TS-I**	-1559.39	0.52	-1558.83	0.10	0.09	-1558.93	-1558.93
**TS-J**	-1559.38	0.52	-1558.83	0.10	0.09	-1558.92	-1558.92

Potential Energy Scan for Barrierless formation of 100 (Ring closure)

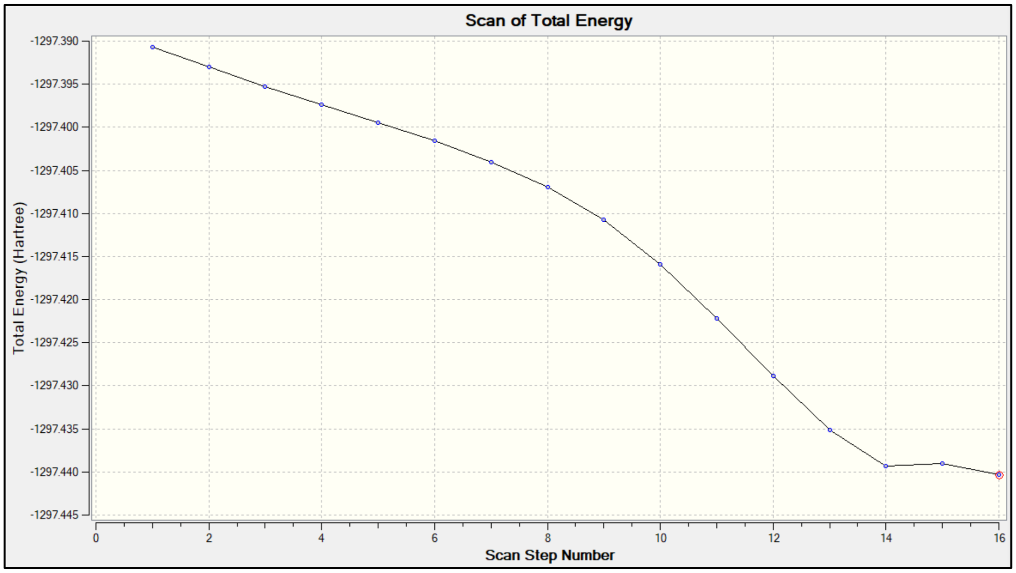

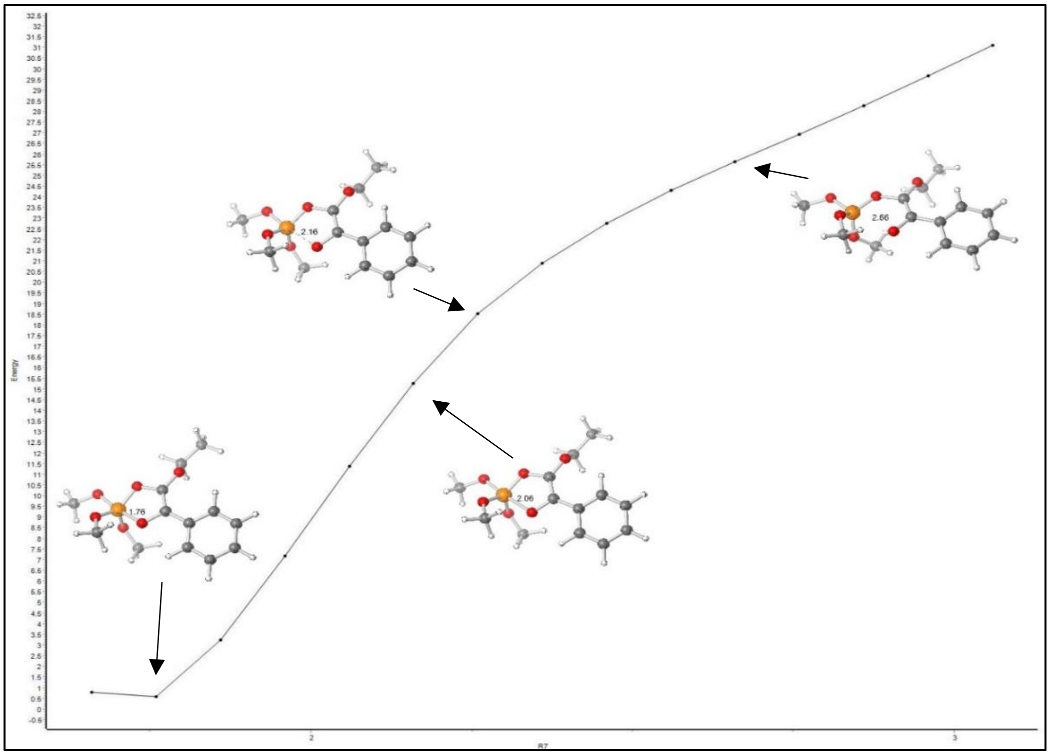

Go back to table of contents

NMR Spectroscopic data

Ethyl 2-(2-fluoro-4,5-dimethoxyphenyl)-2-oxoacetate (S1)

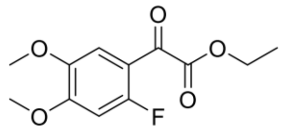

^1H NMR (500 MHz, $CDCl_3$)

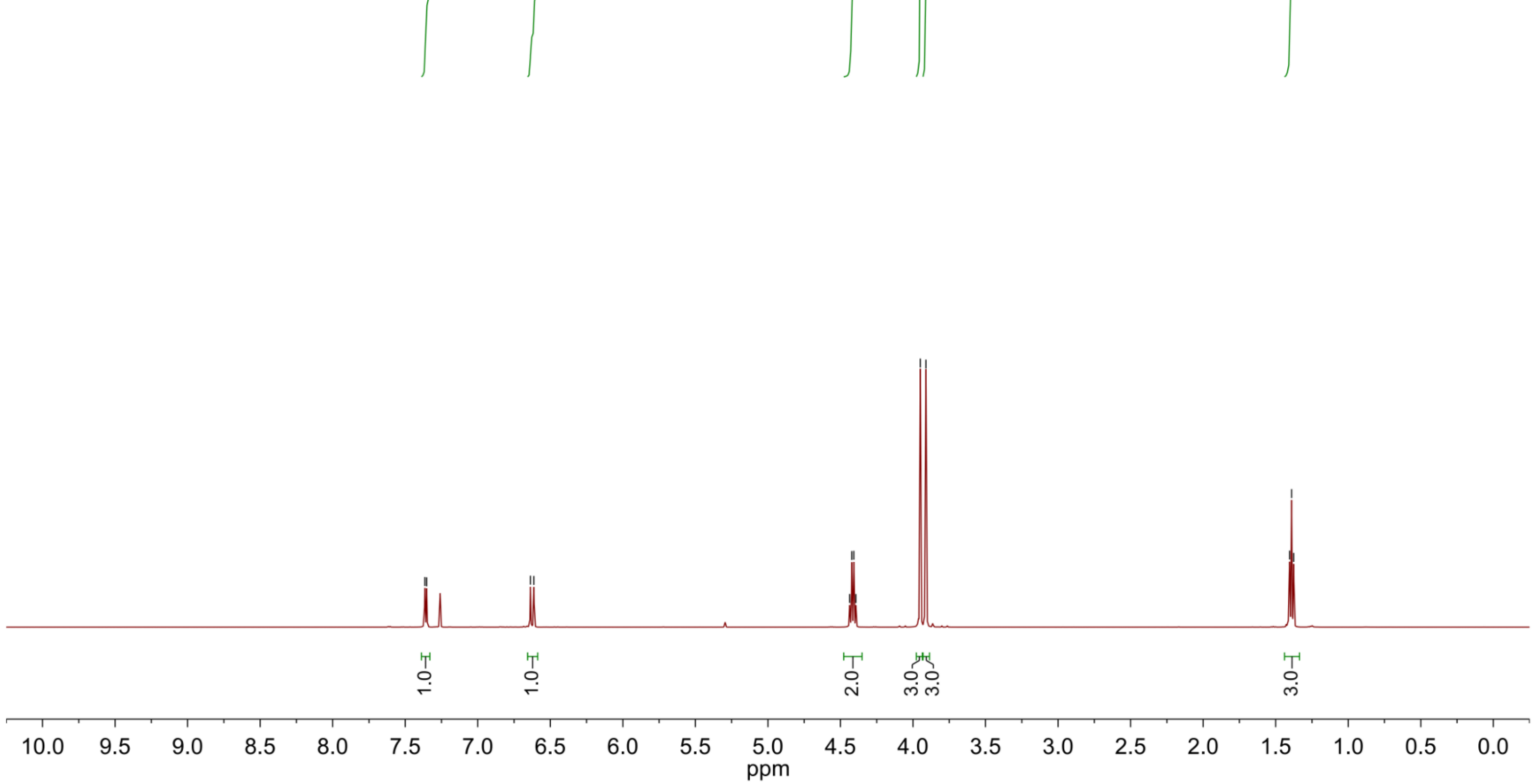

Go back to table of contents

Ethyl 2-(2-fluoro-4,5-dimethoxyphenyl)-2-oxoacetate (S1)

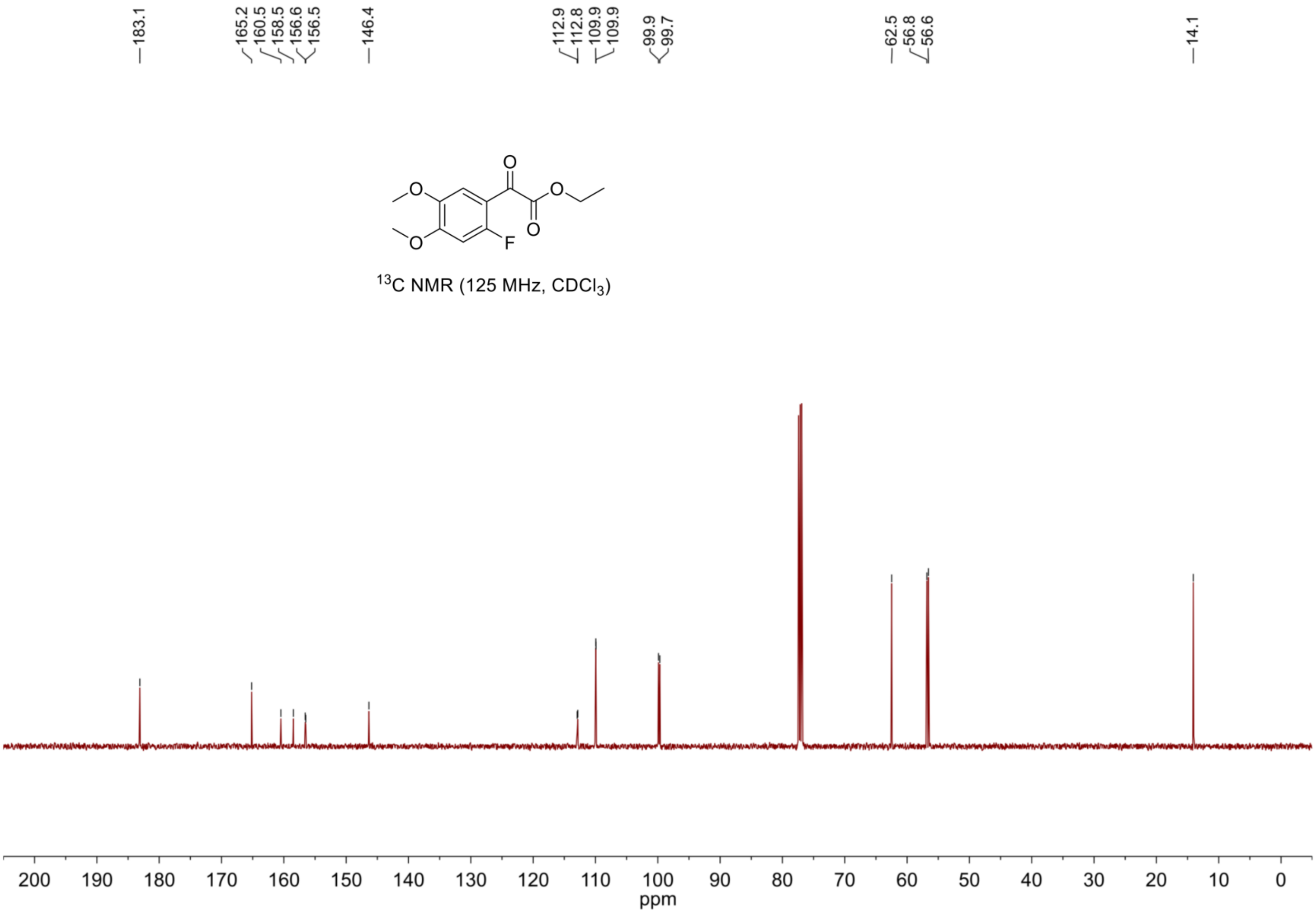

Go back to table of contents

Ethyl 2-(2-fluoro-[1,1'-biphenyl]-4-yl)-2-oxoacetate (S2)

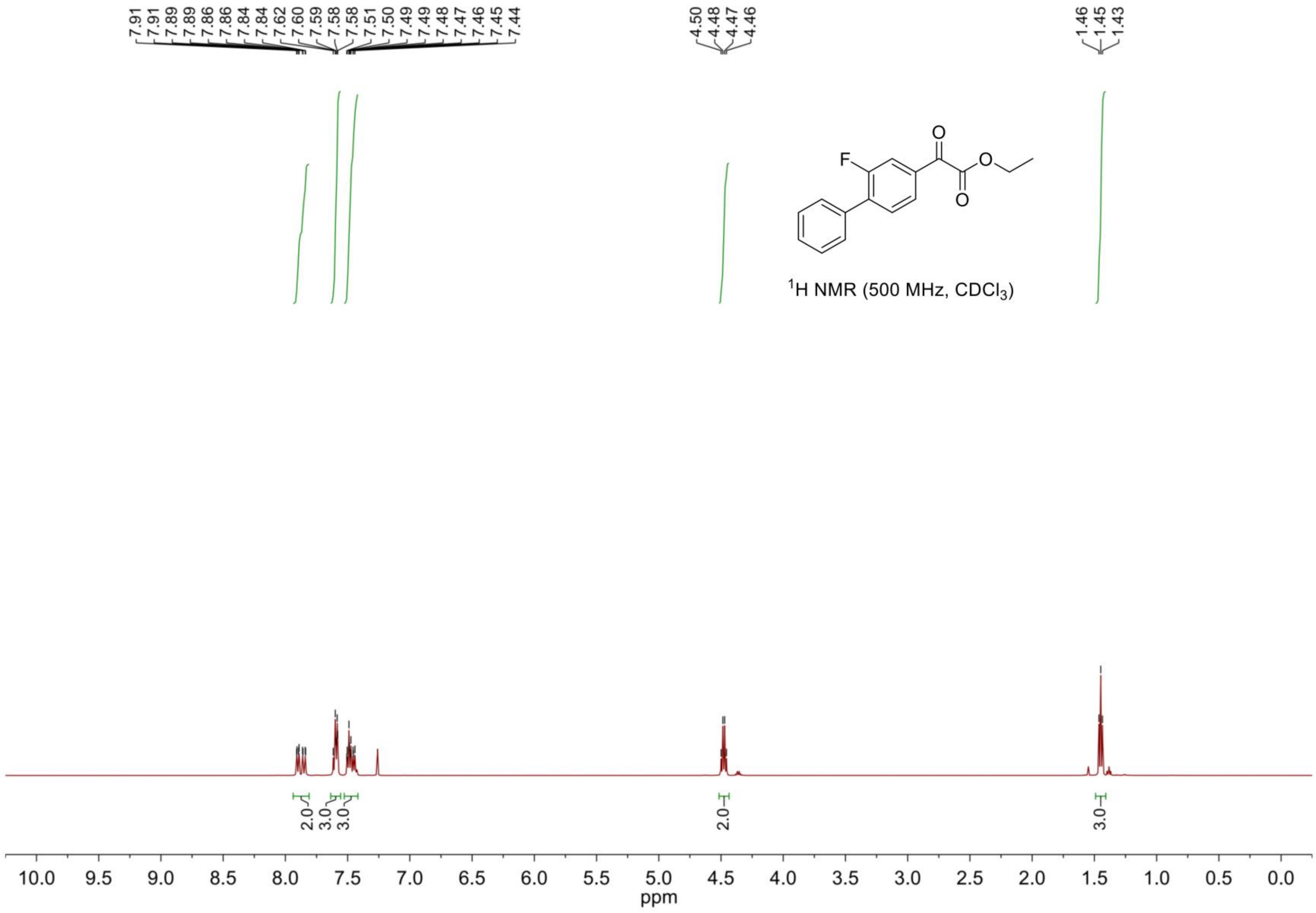

Go back to table of contents

Ethyl 2-(2-fluoro-[1,1'-biphenyl]-4-yl)-2-oxoacetate (S2)

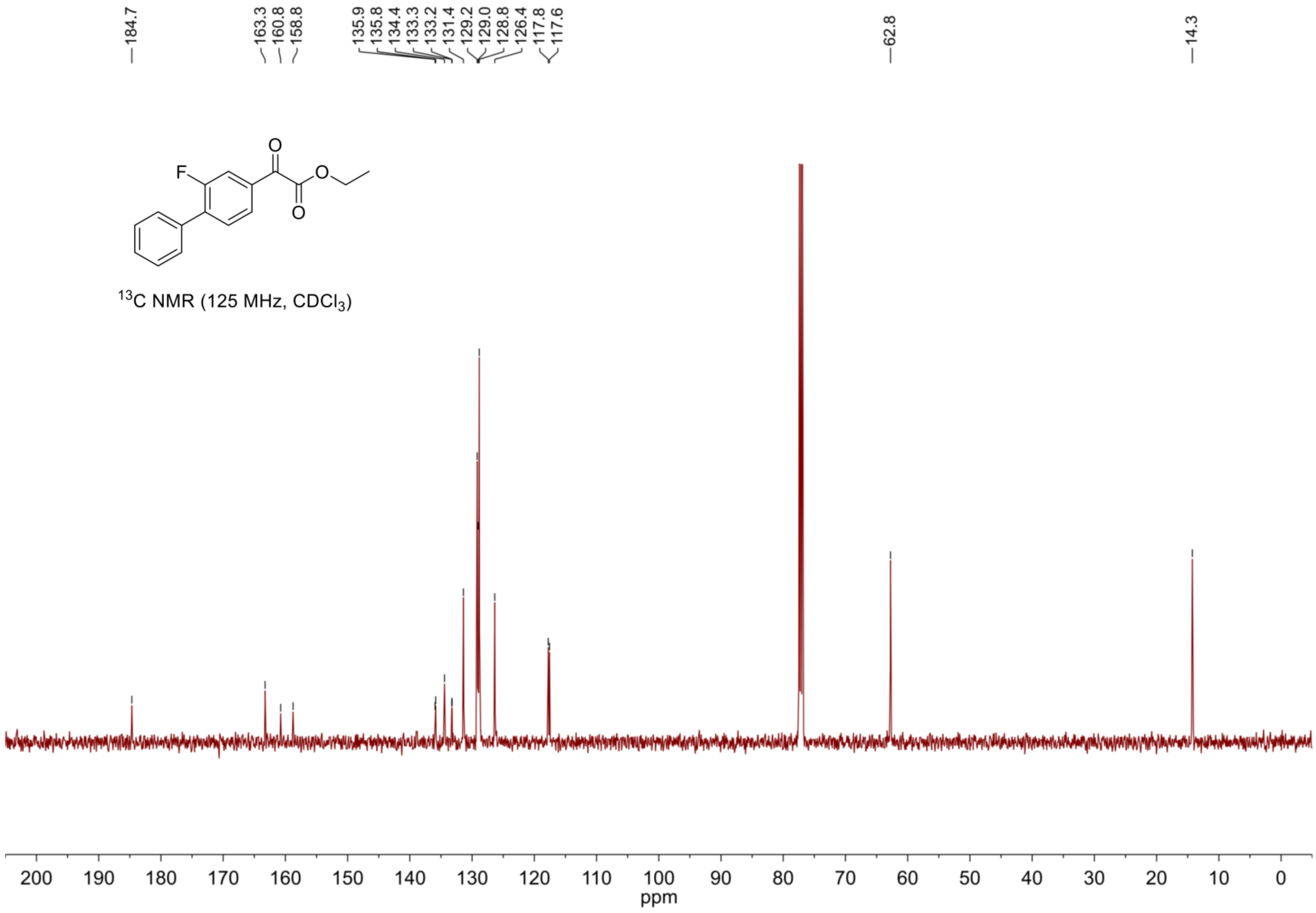

Ethyl 4-(2,3-dihydrobenzofuran-5-yl)-2-oxobutanoate (S3)

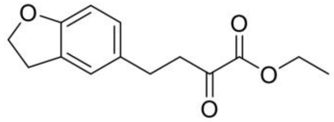

^{1}H NMR (500 MHz, $CDCl_3$)

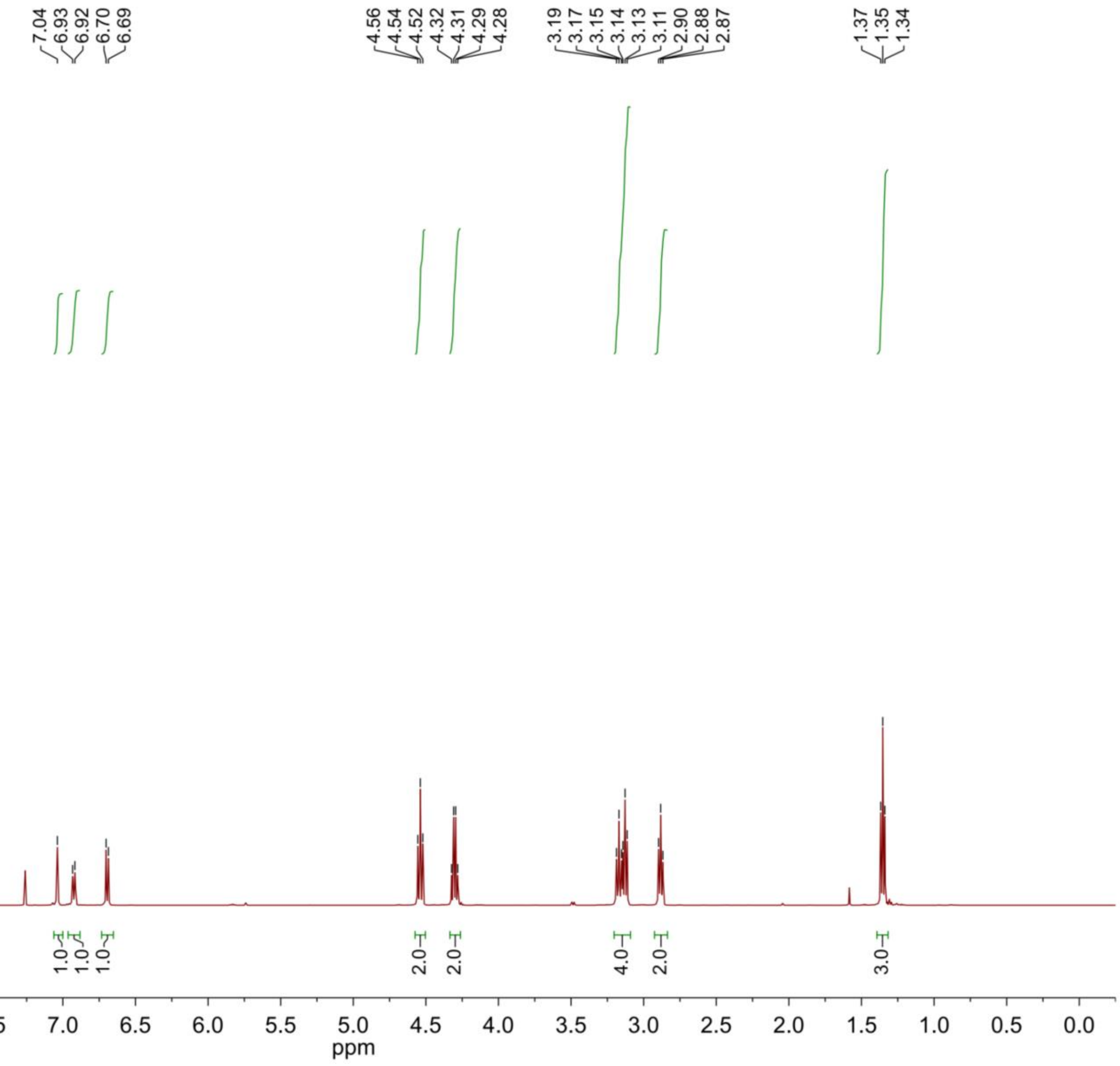

Go back to table of contents

Ethyl 4-(2,3-dihydrobenzofuran-5-yl)-2-oxobutanoate (S3)

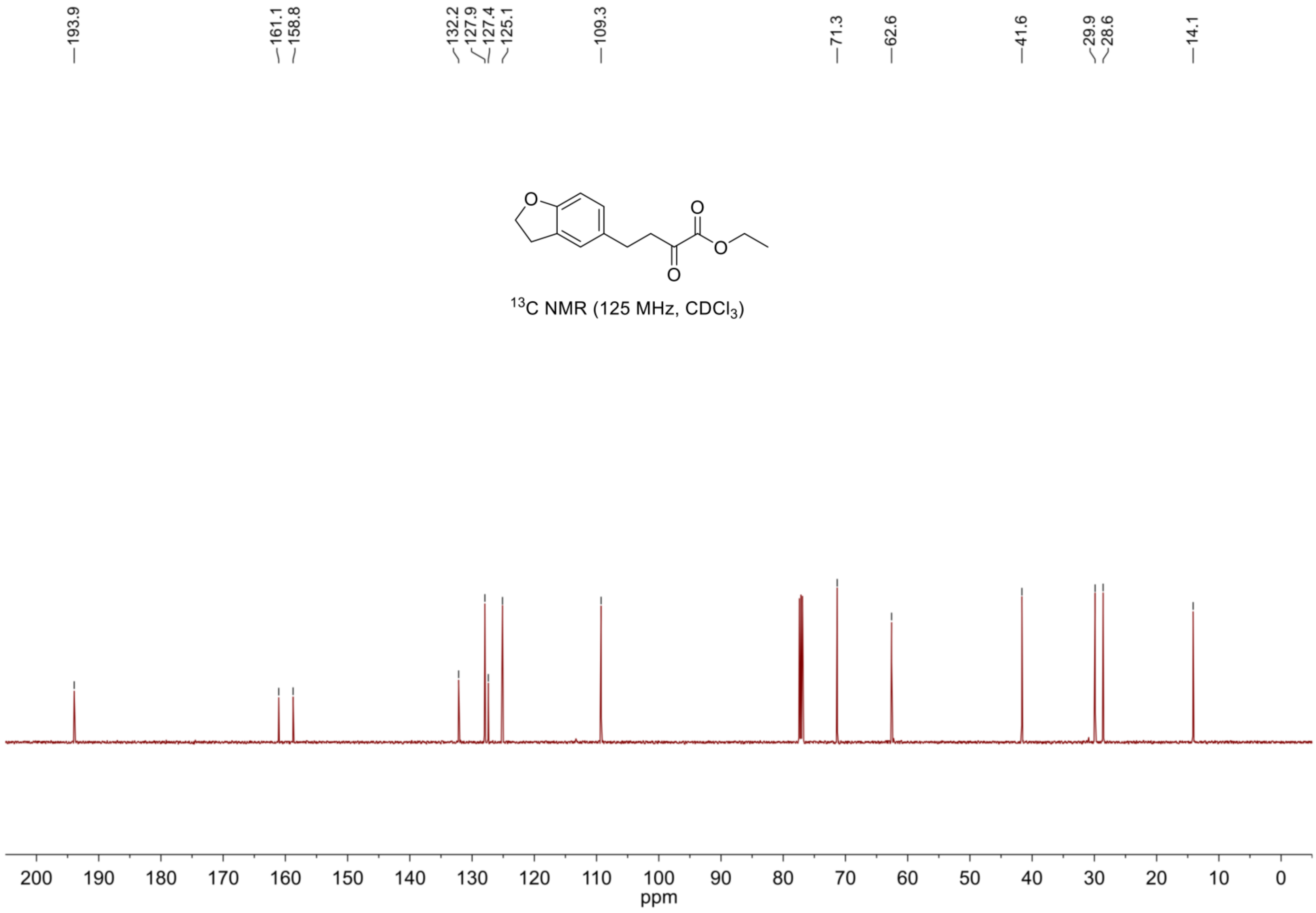

Go back to table of contents

Ethyl 2-(1-(2,5-dimethylbenzyl)-1*H*-indol-5-yl)-2-oxoacetate (S4)

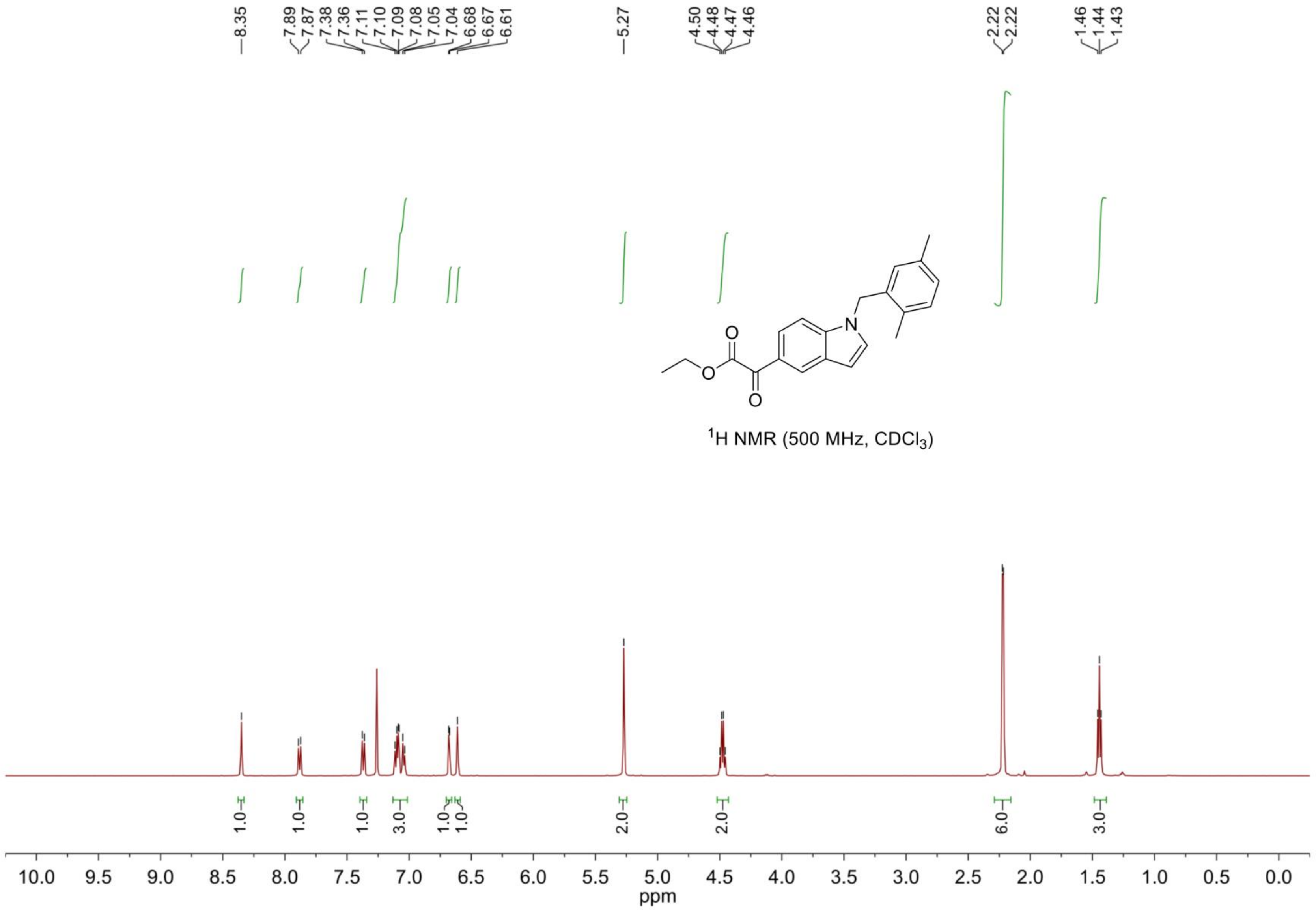

Go back to table of contents

Ethyl 2-(1-(2,5-dimethylbenzyl)-1*H*-indol-5-yl)-2-oxoacetate (S4)

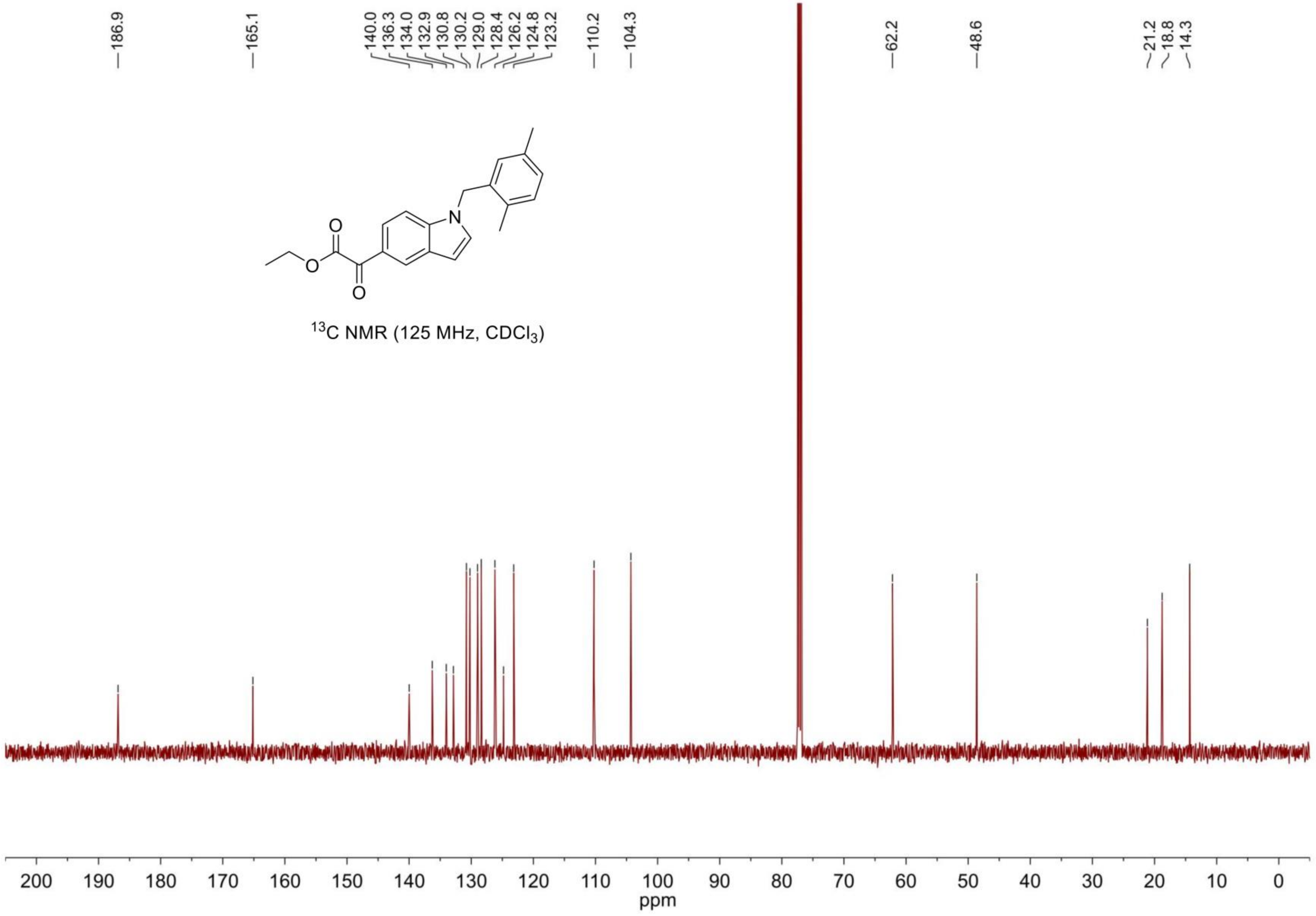

Go back to table of contents

Ethyl 2-(3-((5-(4-fluorophenyl)thiophen-2-yl)methyl)-4-methylphenyl)-2-oxoacetate (S5)

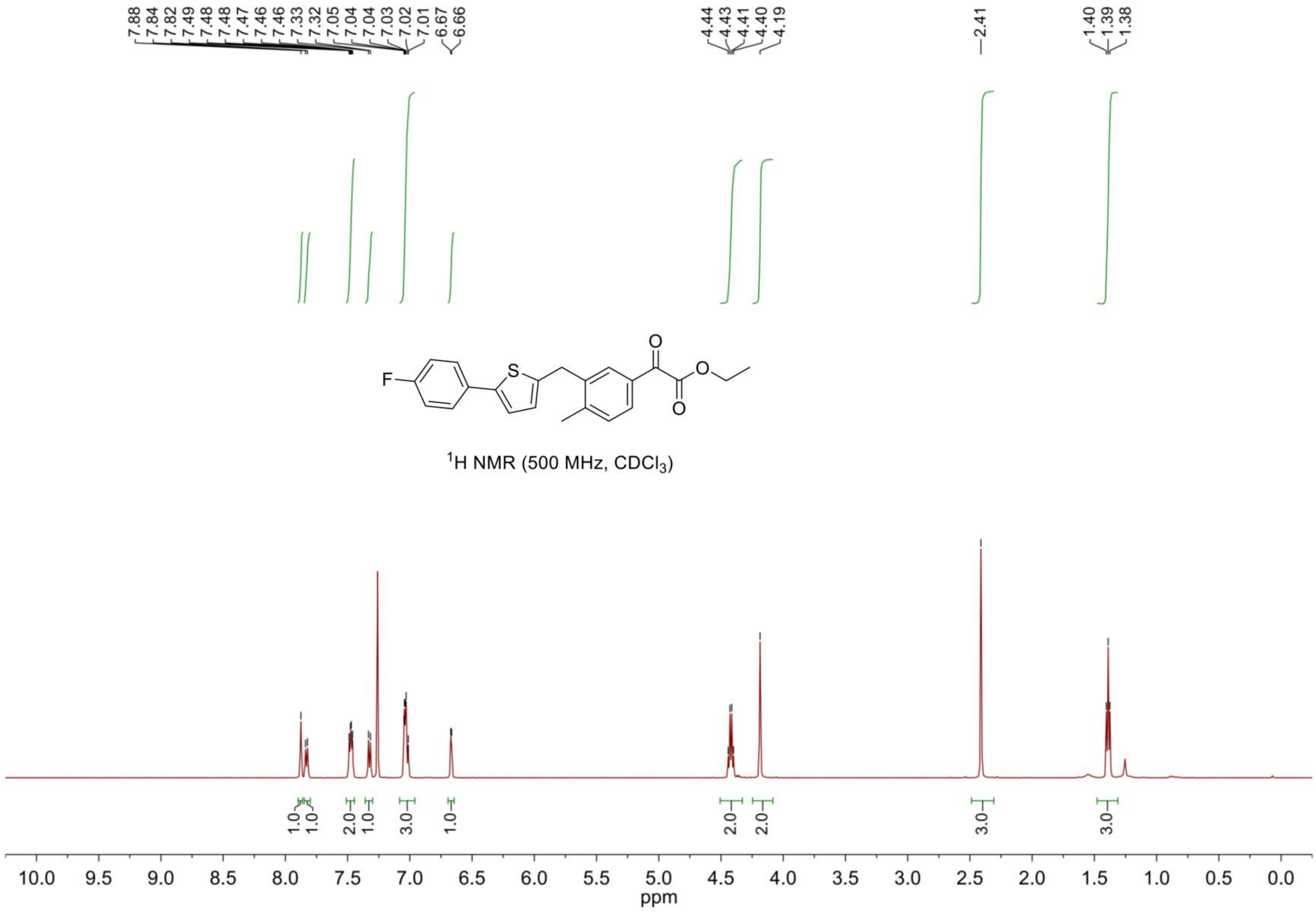

Go back to table of contents

Ethyl 2-(3-((5-(4-fluorophenyl)thiophen-2-yl)methyl)-4-methylphenyl)-2-oxoacetate (S5)

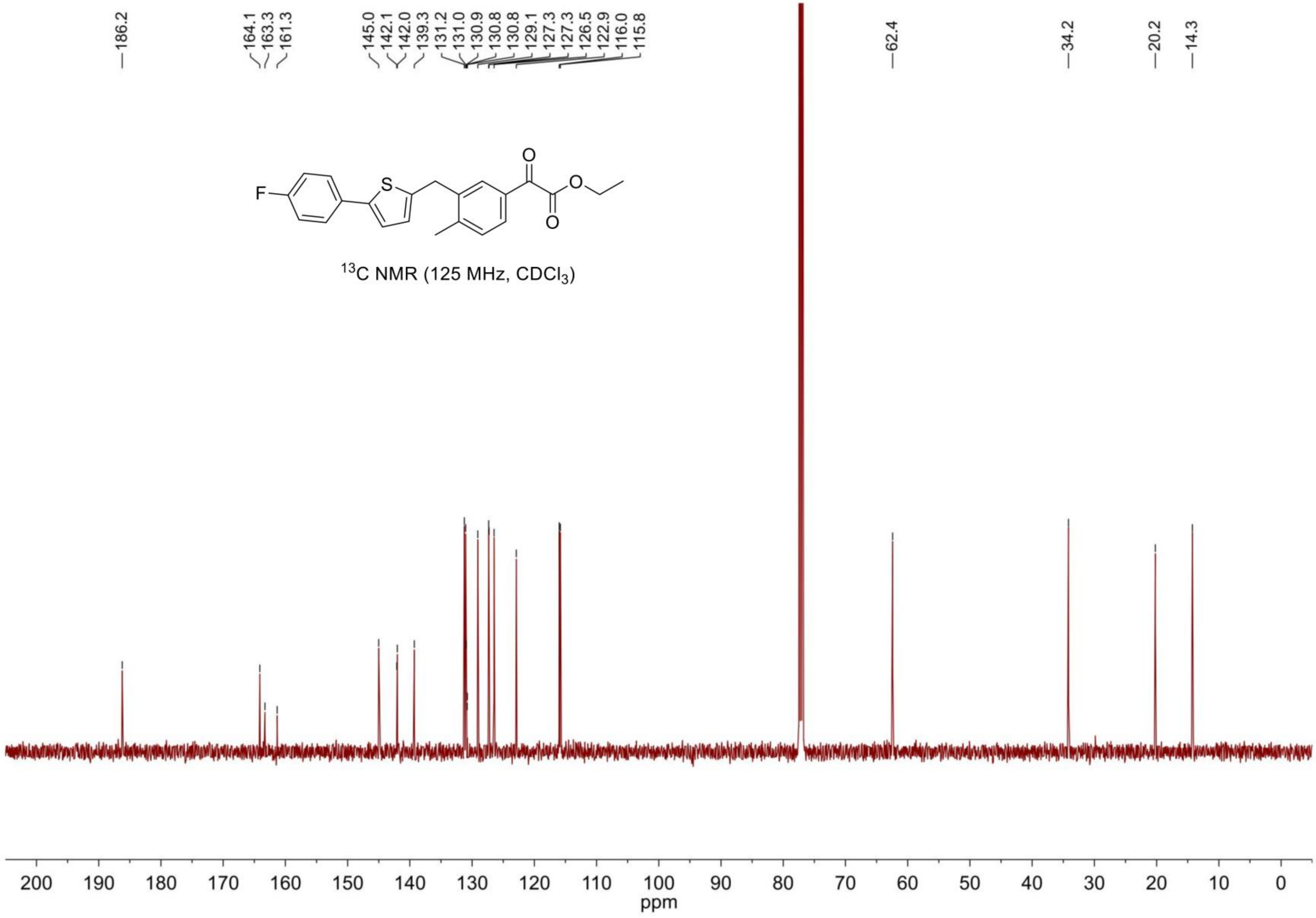

^{13}C NMR (125 MHz, $CDCl_3$)

Go back to table of contents

Buta-2,3-dien-1-yl 2-oxo-2-phenylacetate (S6)

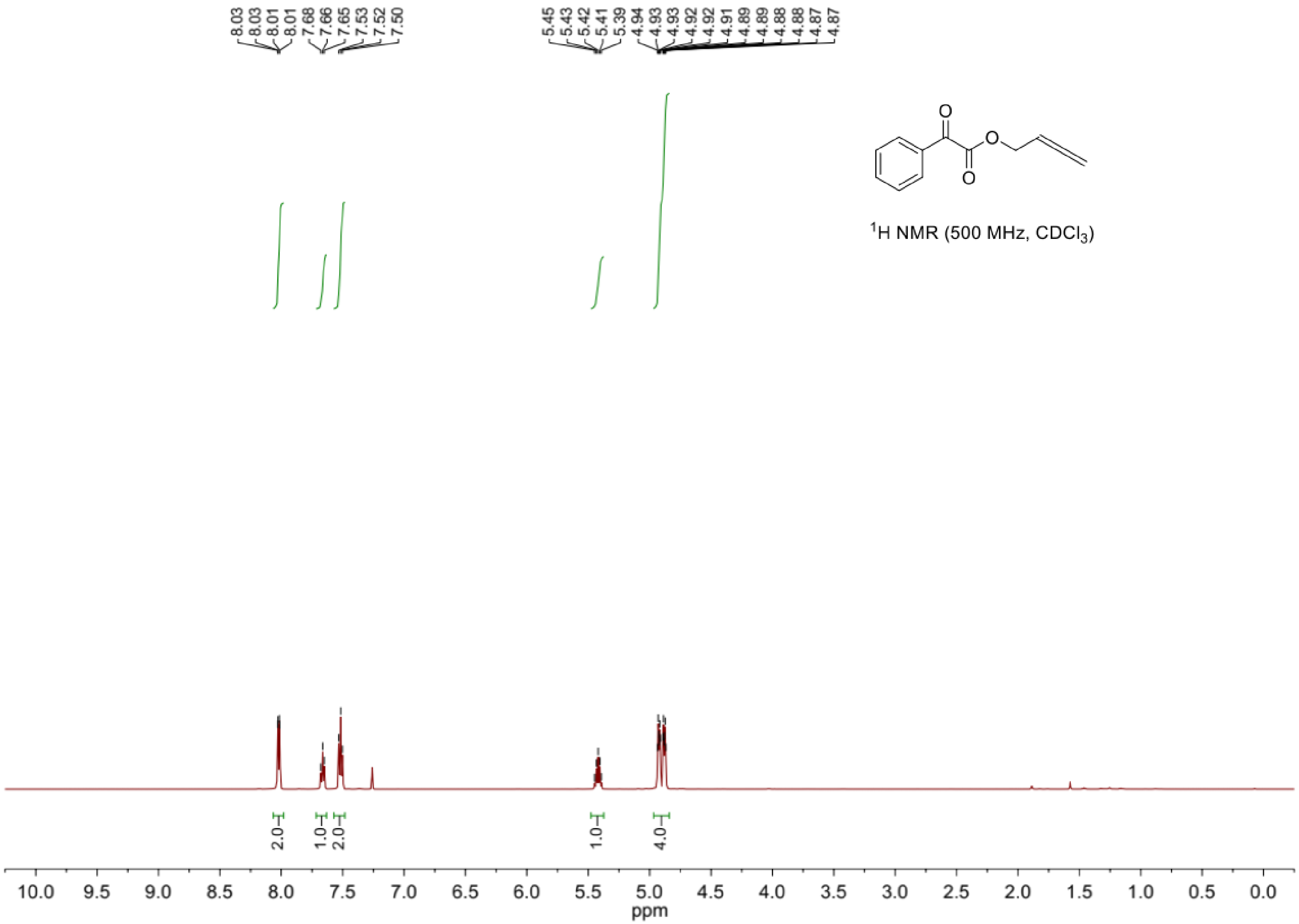

Buta-2,3-dien-1-yl 2-oxo-2-phenylacetate (S6)

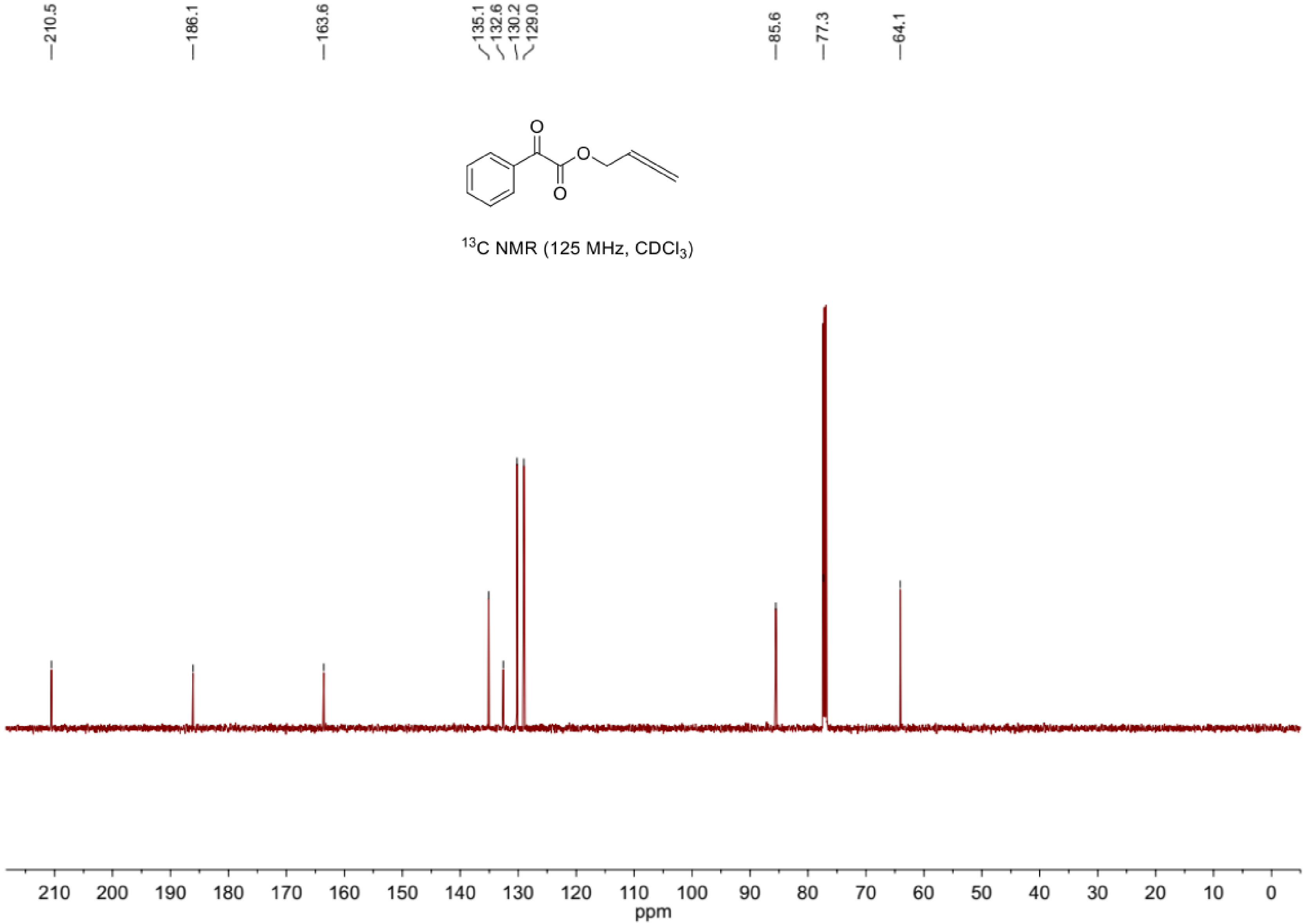

Go back to table of contents

Ethyl 2-phenylbutanoate (7)

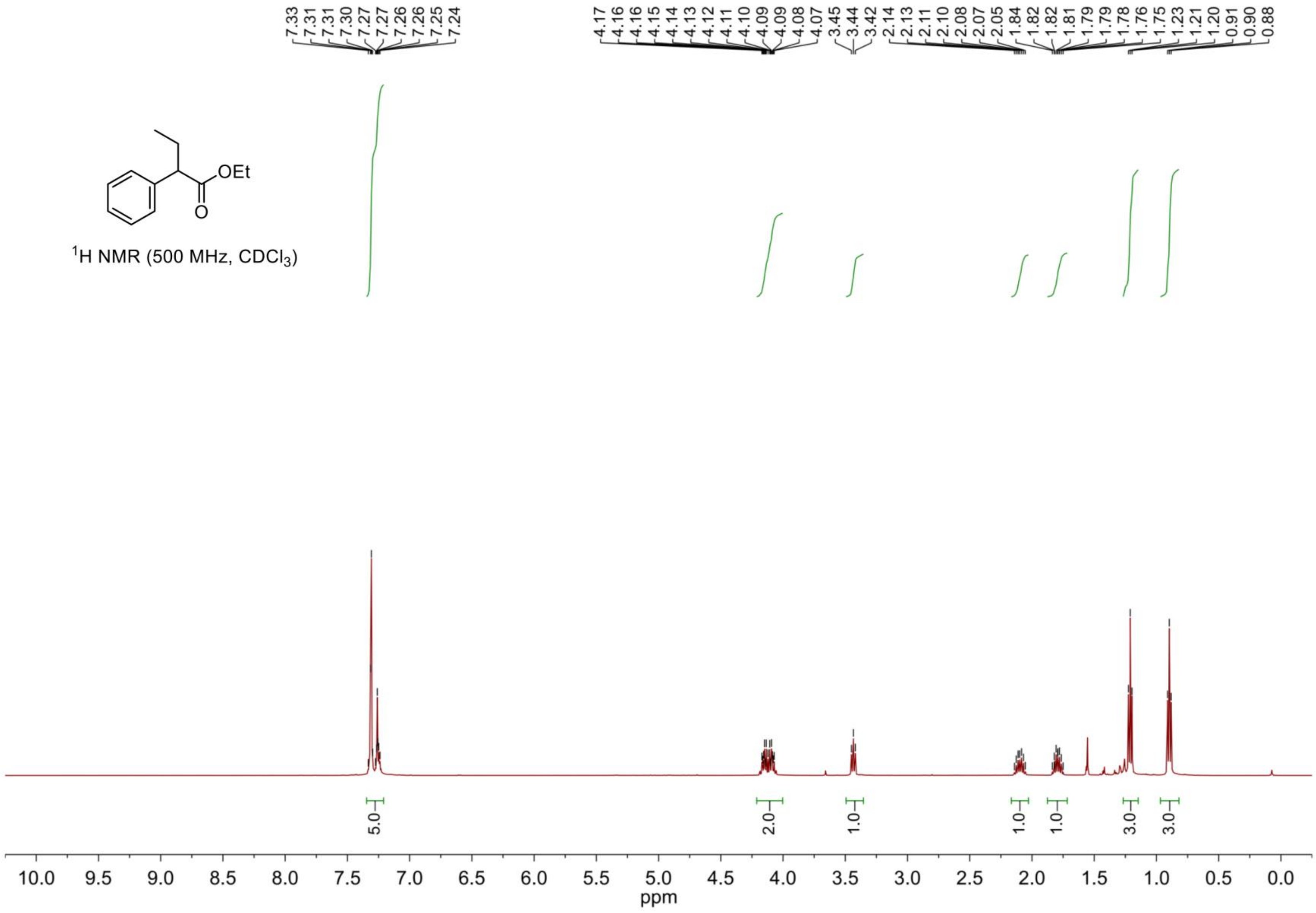

Go back to table of contents

Ethyl 2-phenylbutanoate (7)

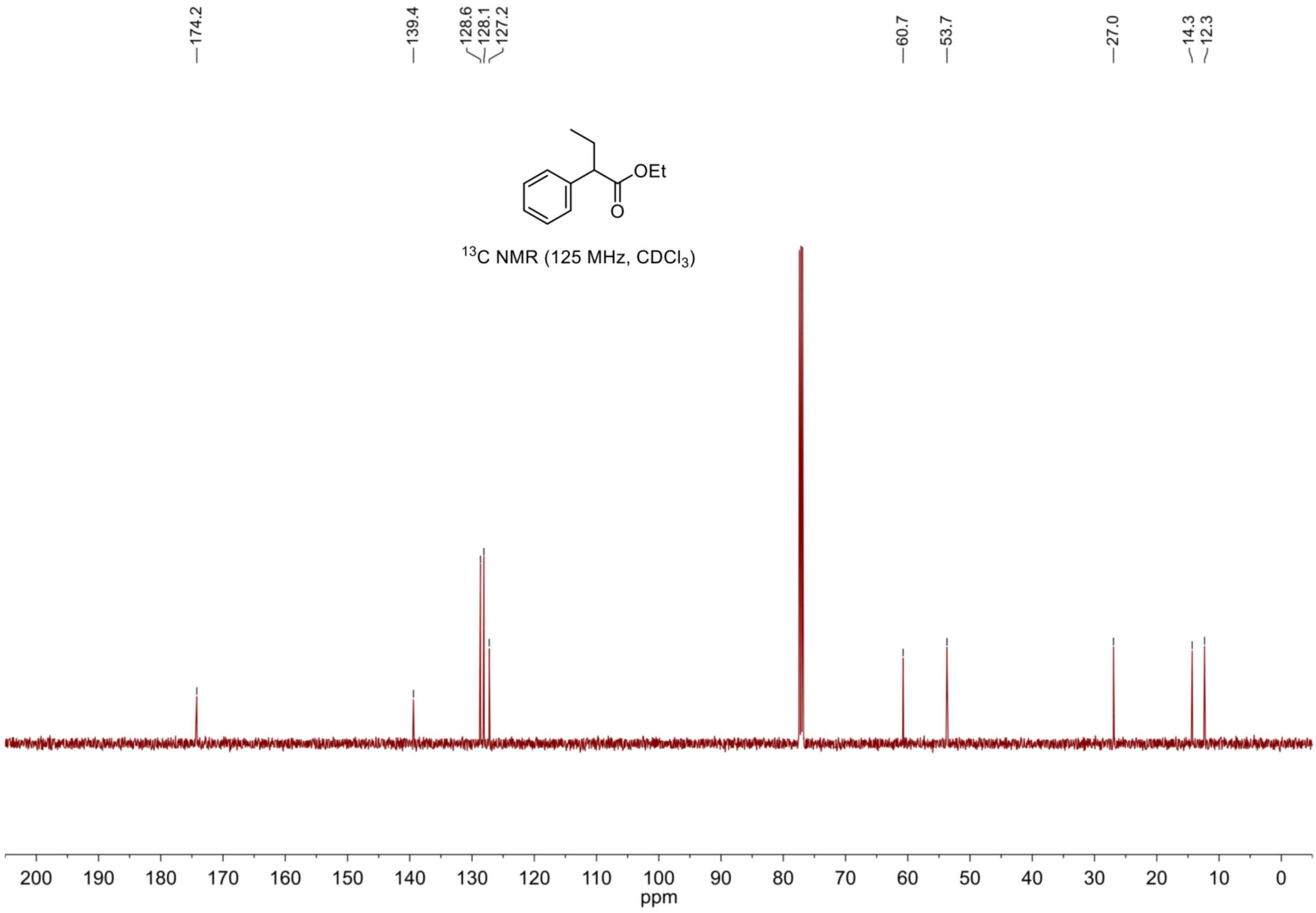

Go back to table of contents

Ethyl 2-phenylhexanoate (56)

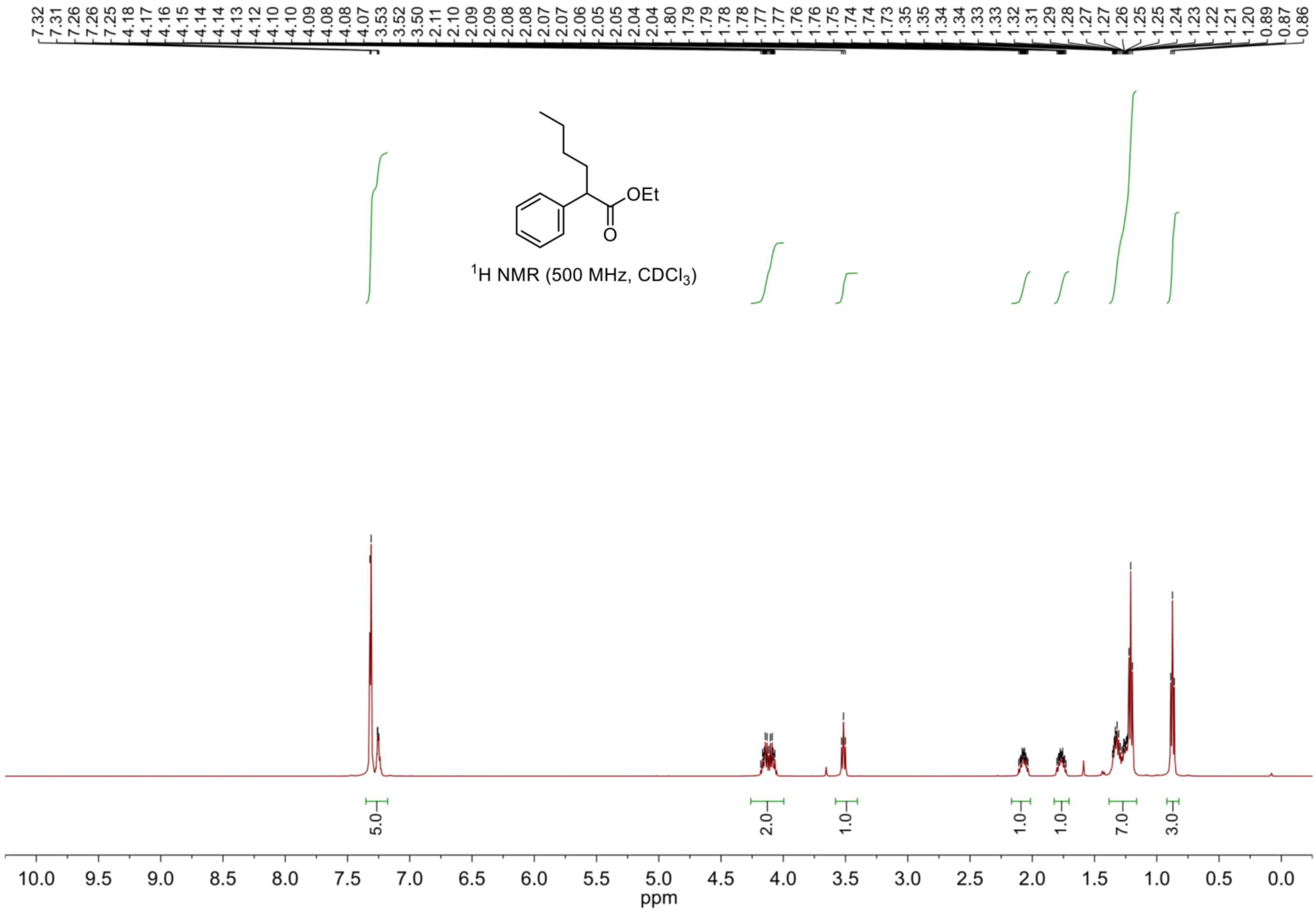

Go back to table of contents

Ethyl 2-phenylhexanoate (56)

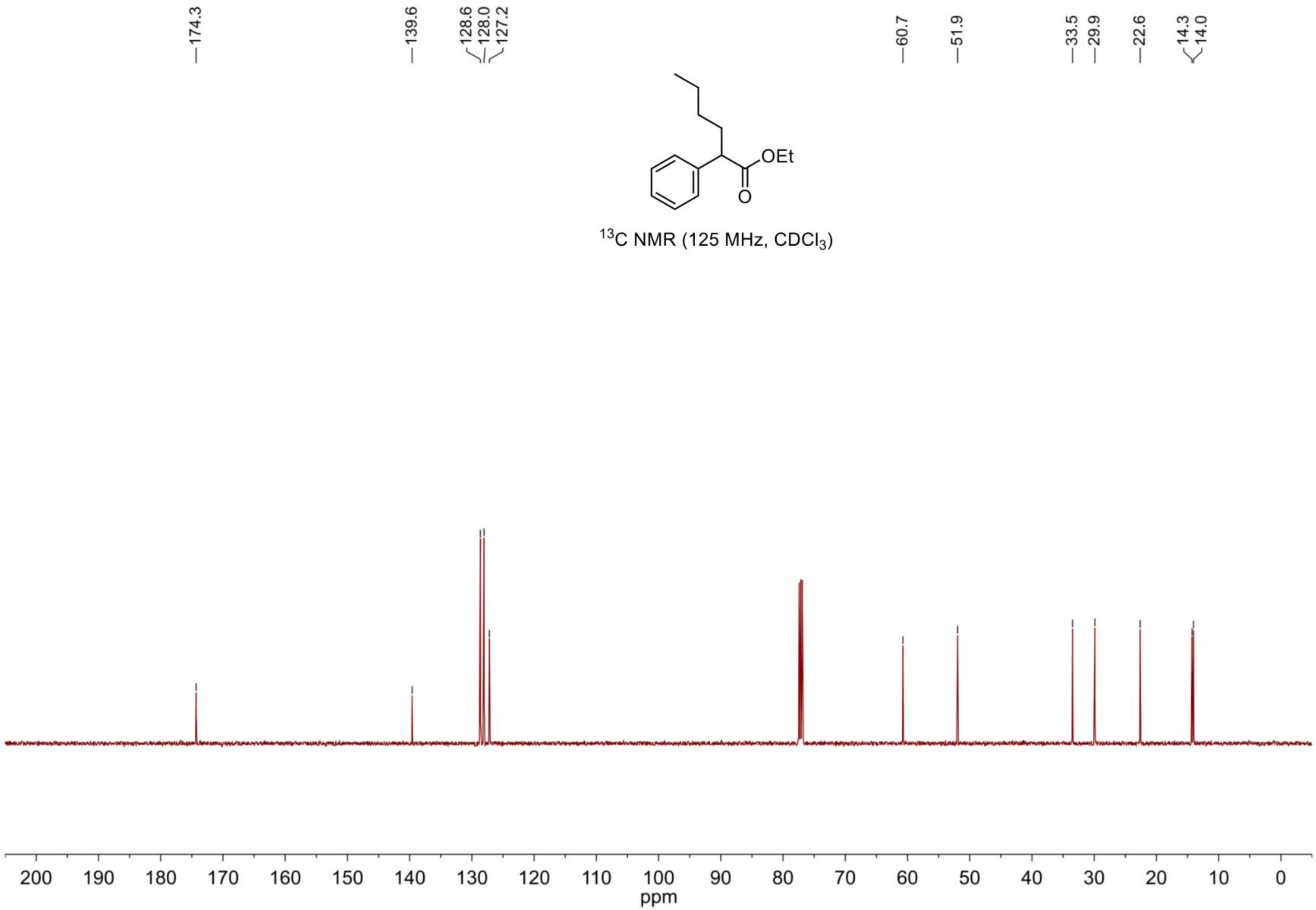

Go back to table of contents

Ethyl 2-(4-fluorophenyl)butanoate (8)

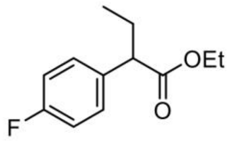

^{1}H NMR (500 MHz, $CDCl_3$)

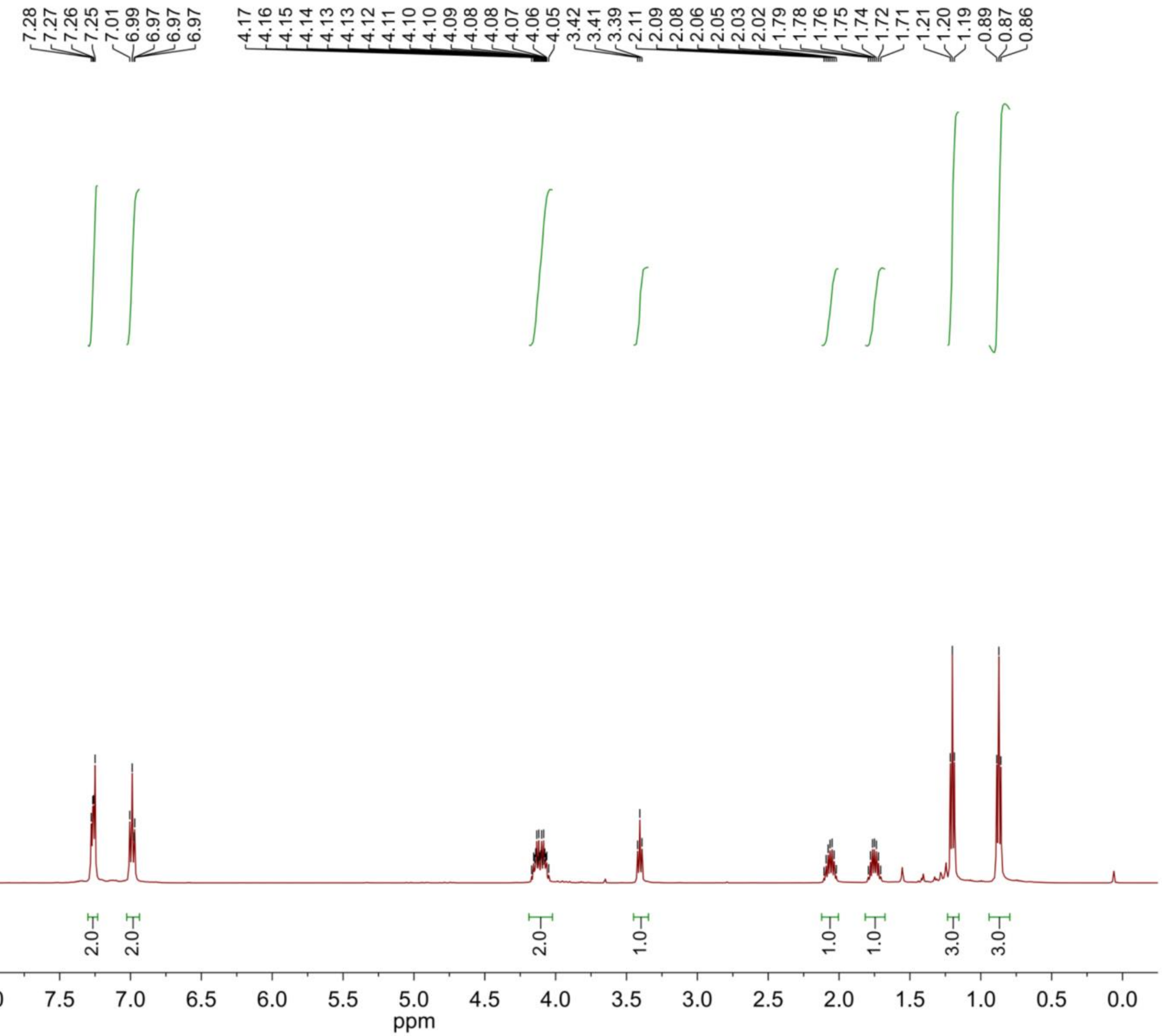

Go back to table of contents

Ethyl 2-(4-fluorophenyl)butanoate (8)

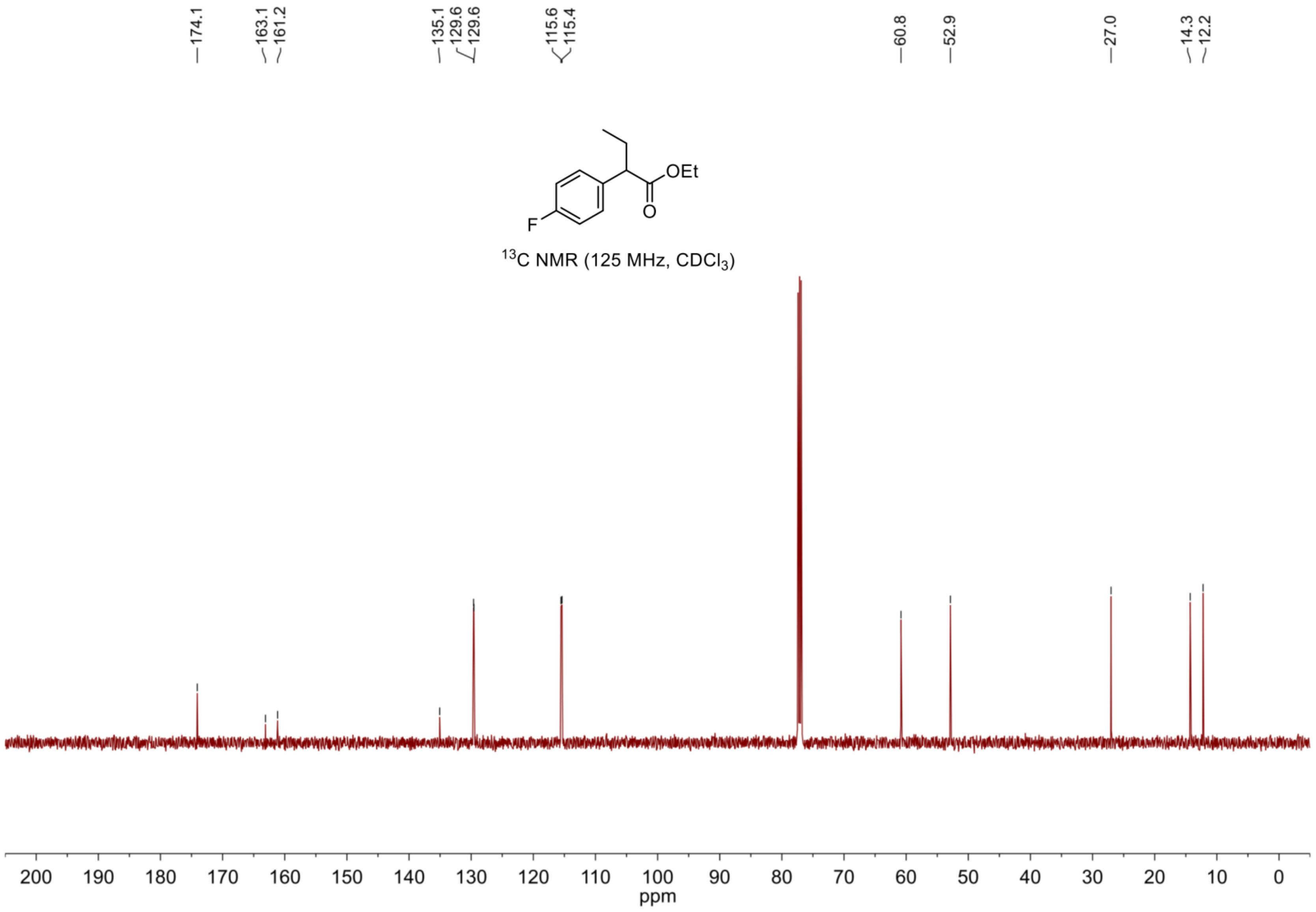

Go back to table of contents

Ethyl 2-(4-chlorophenyl)butanoate (9)

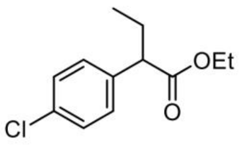

^{1}H NMR (500 MHz, $CDCl_3$)

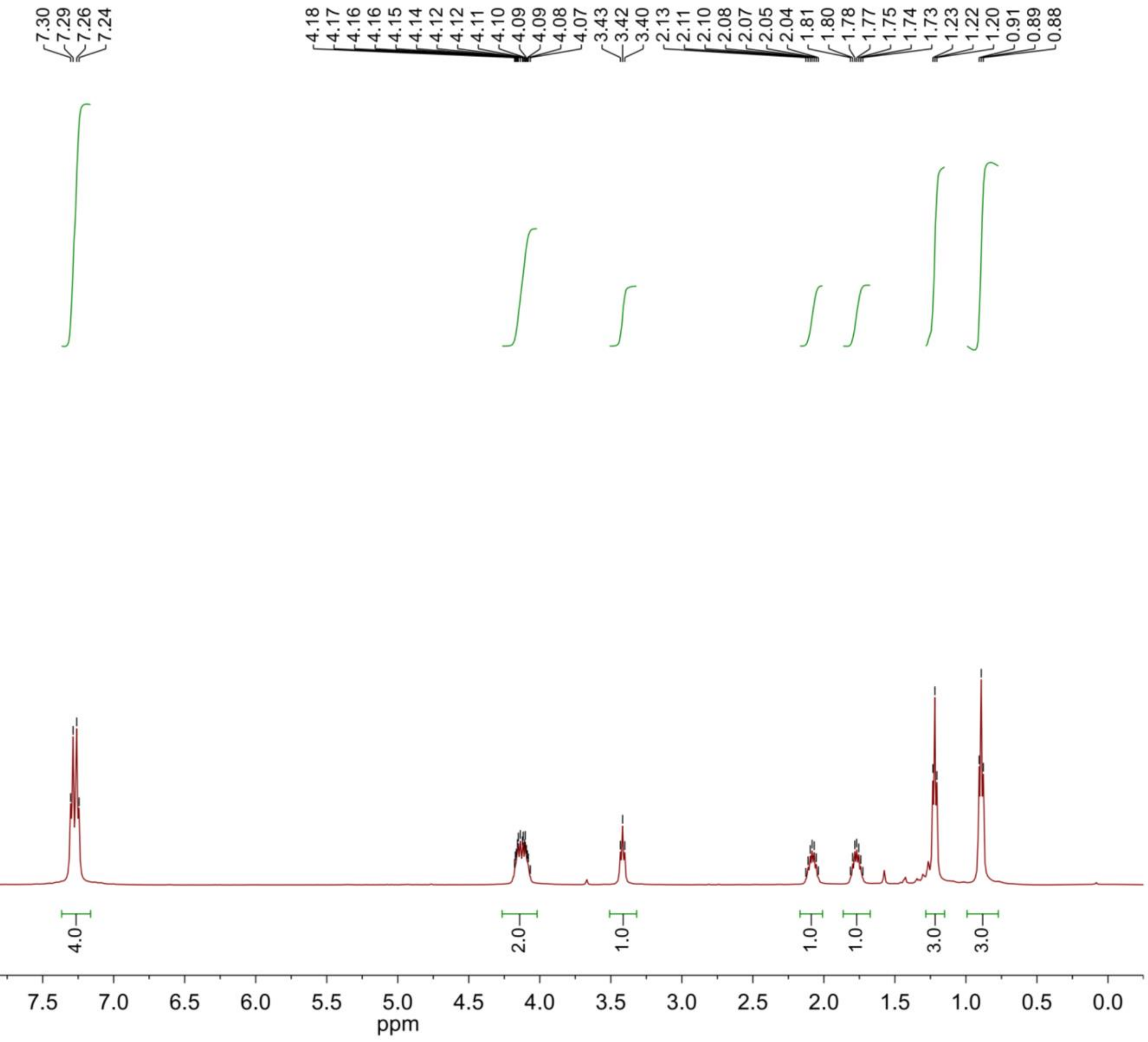

Go back to table of contents

Ethyl 2-(4-chlorophenyl)butanoate (9)

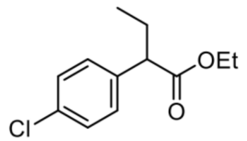

^{13}C NMR (125 MHz, $CDCl_3$)

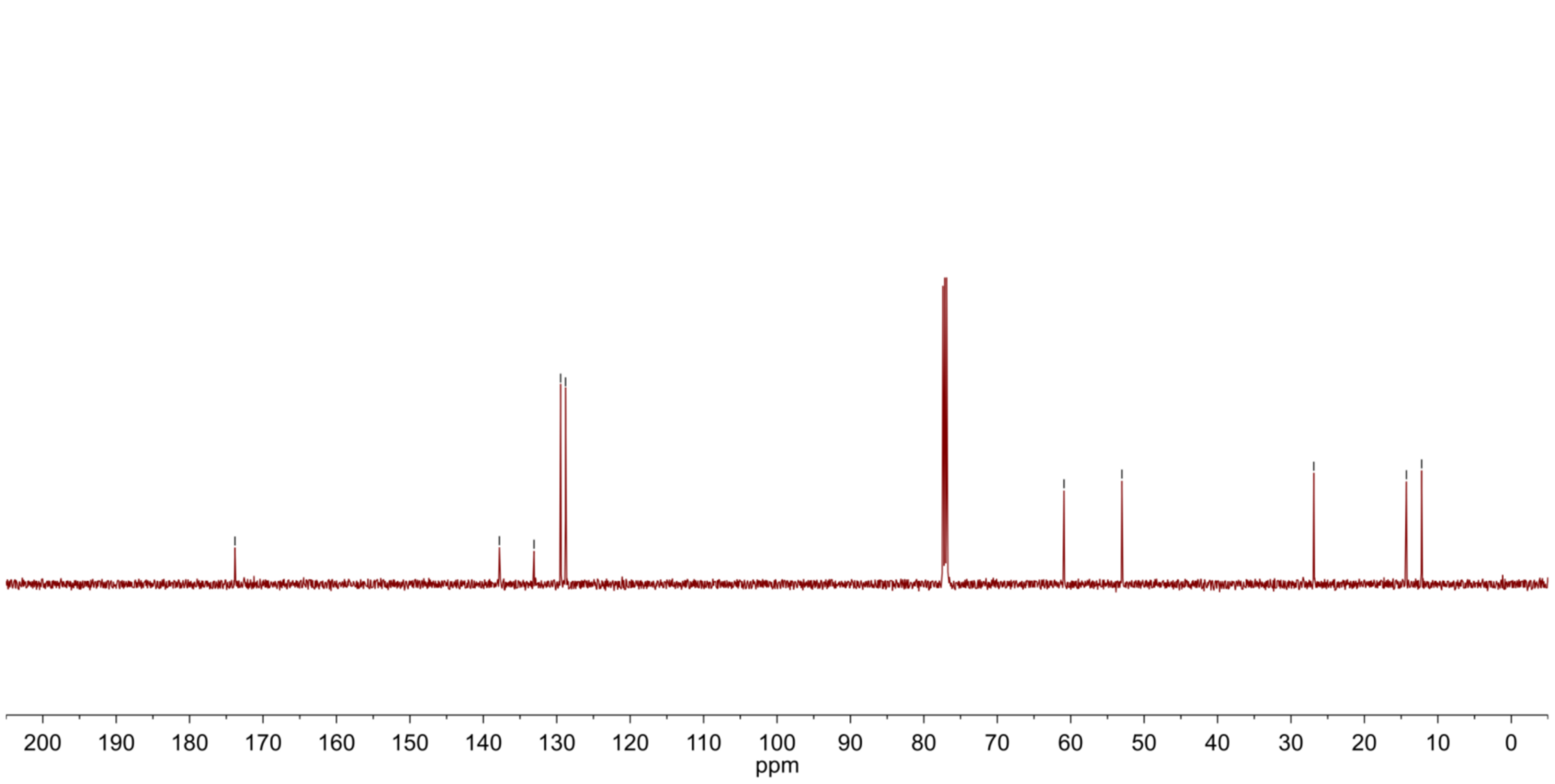

Go back to table of contents

Ethyl 2-(4-bromophenyl)butanoate (10)

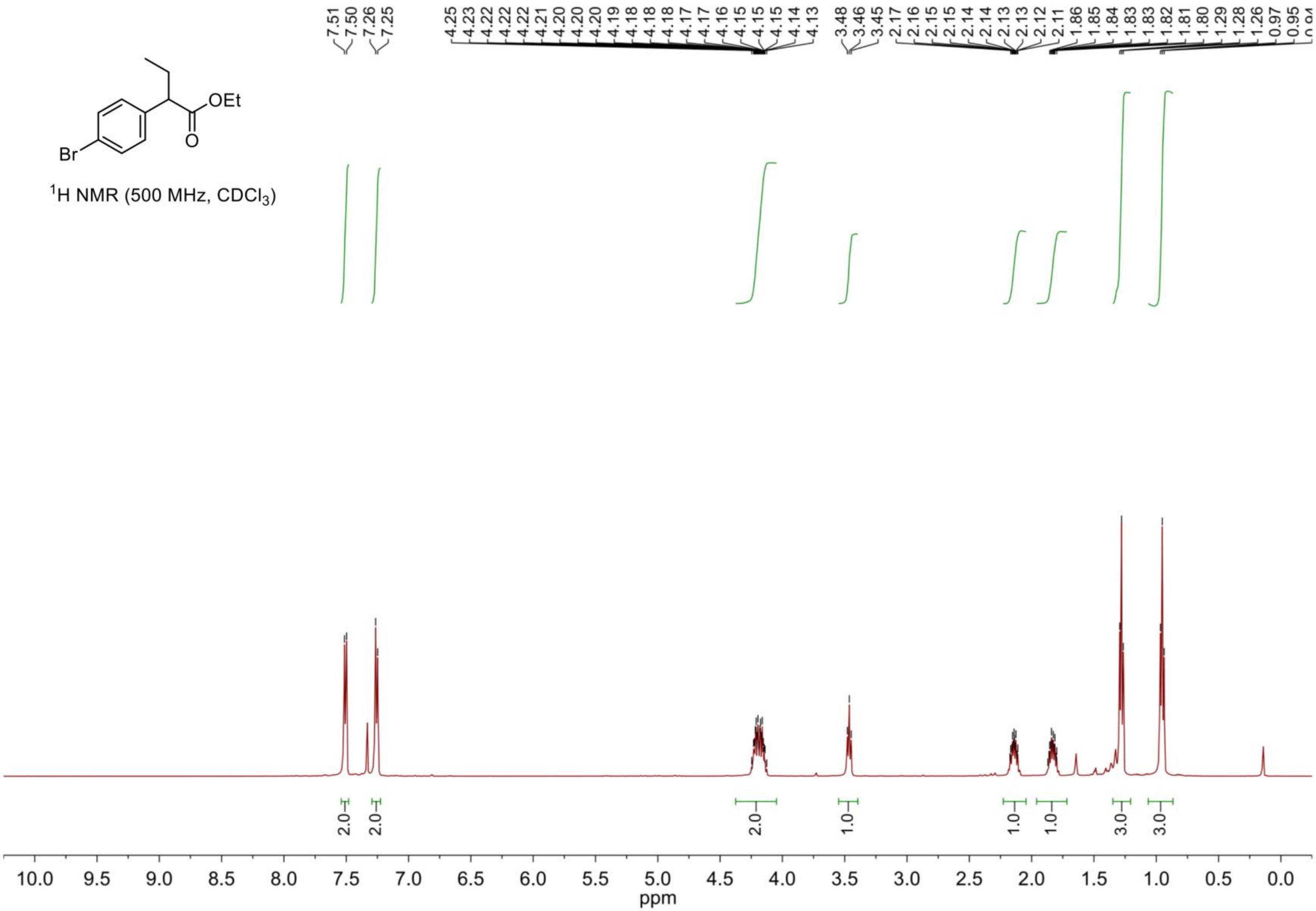

Ethyl 2-(4-bromophenyl)butanoate (10)

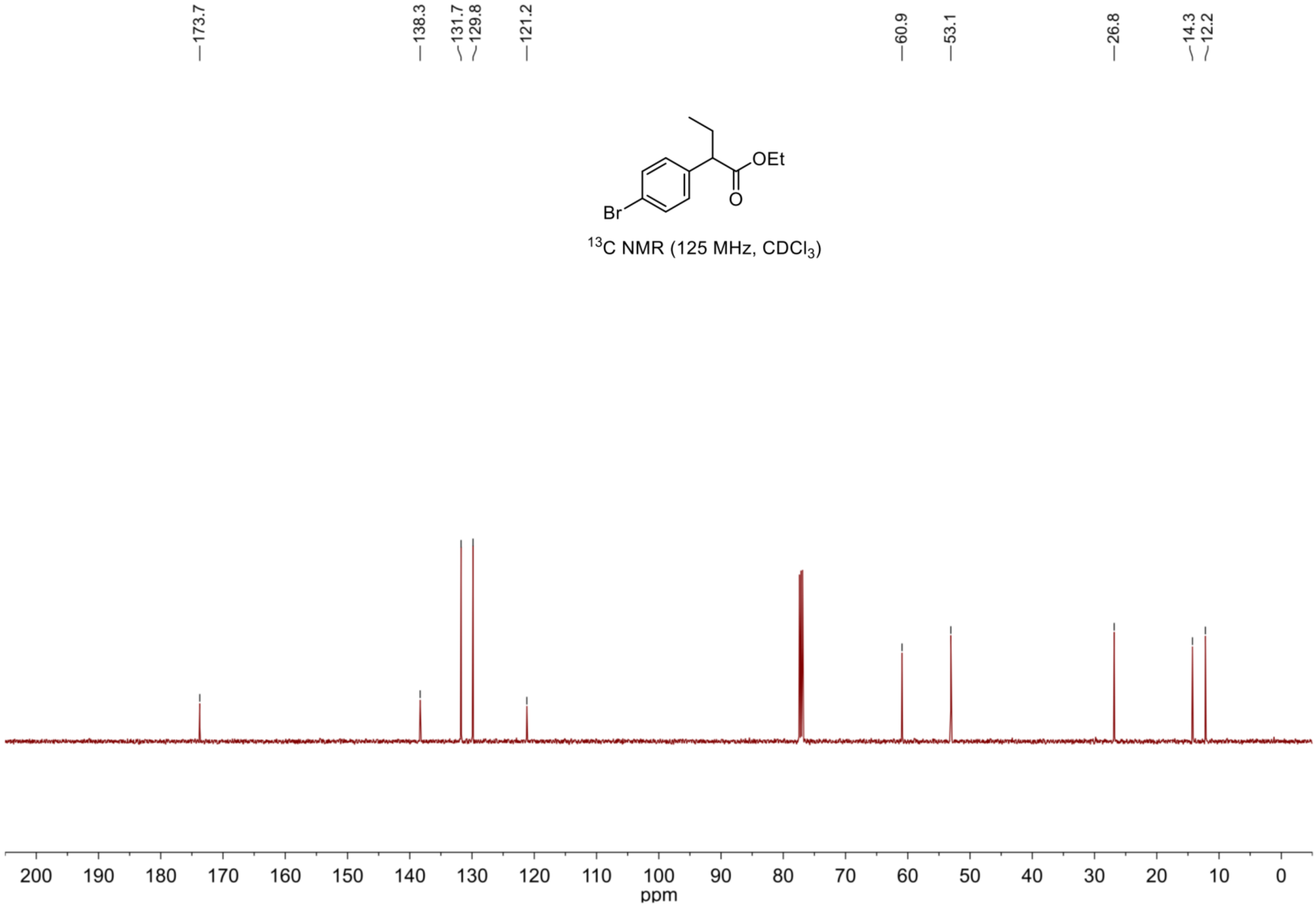

Go back to table of contents

Ethyl 2-(*p*-tolyl)butanoate (11)

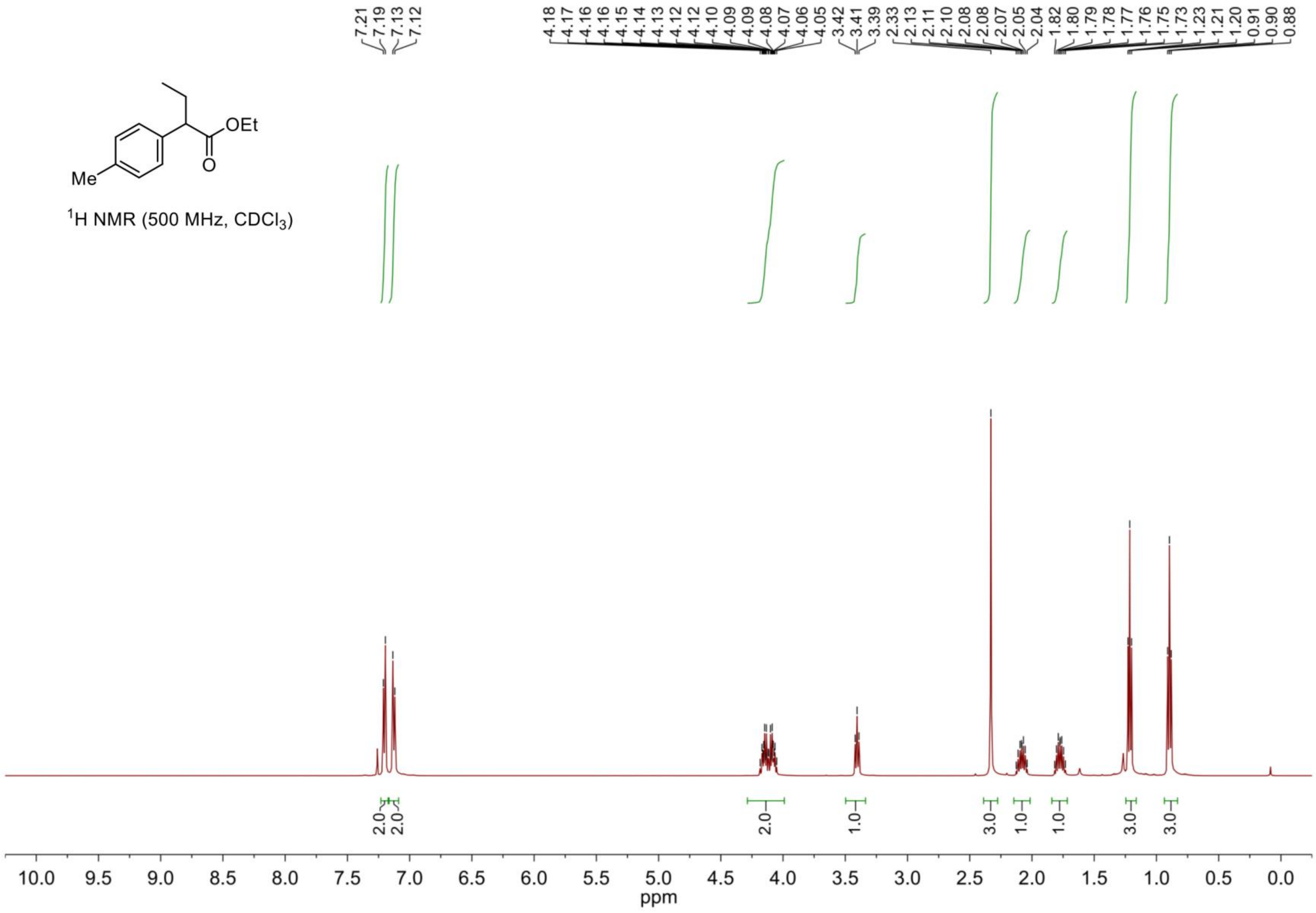

Ethyl 2-(*p*-tolyl)butanoate (11)

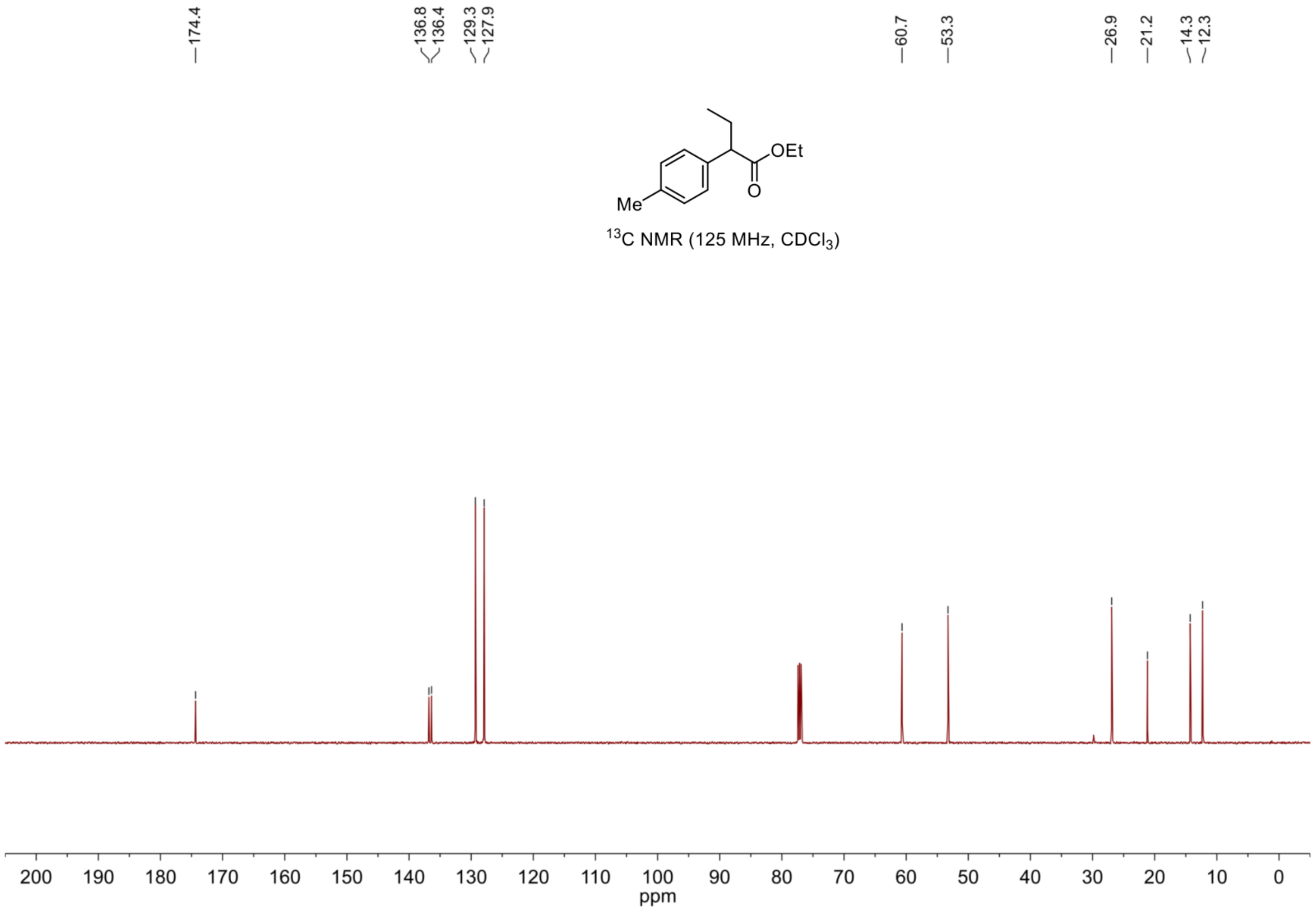

Go back to table of contents

Ethyl 2-(*m*-tolyl)butanoate (12)

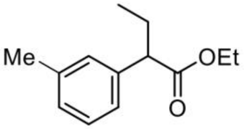

^1H NMR (500 MHz, $CDCl_3$)

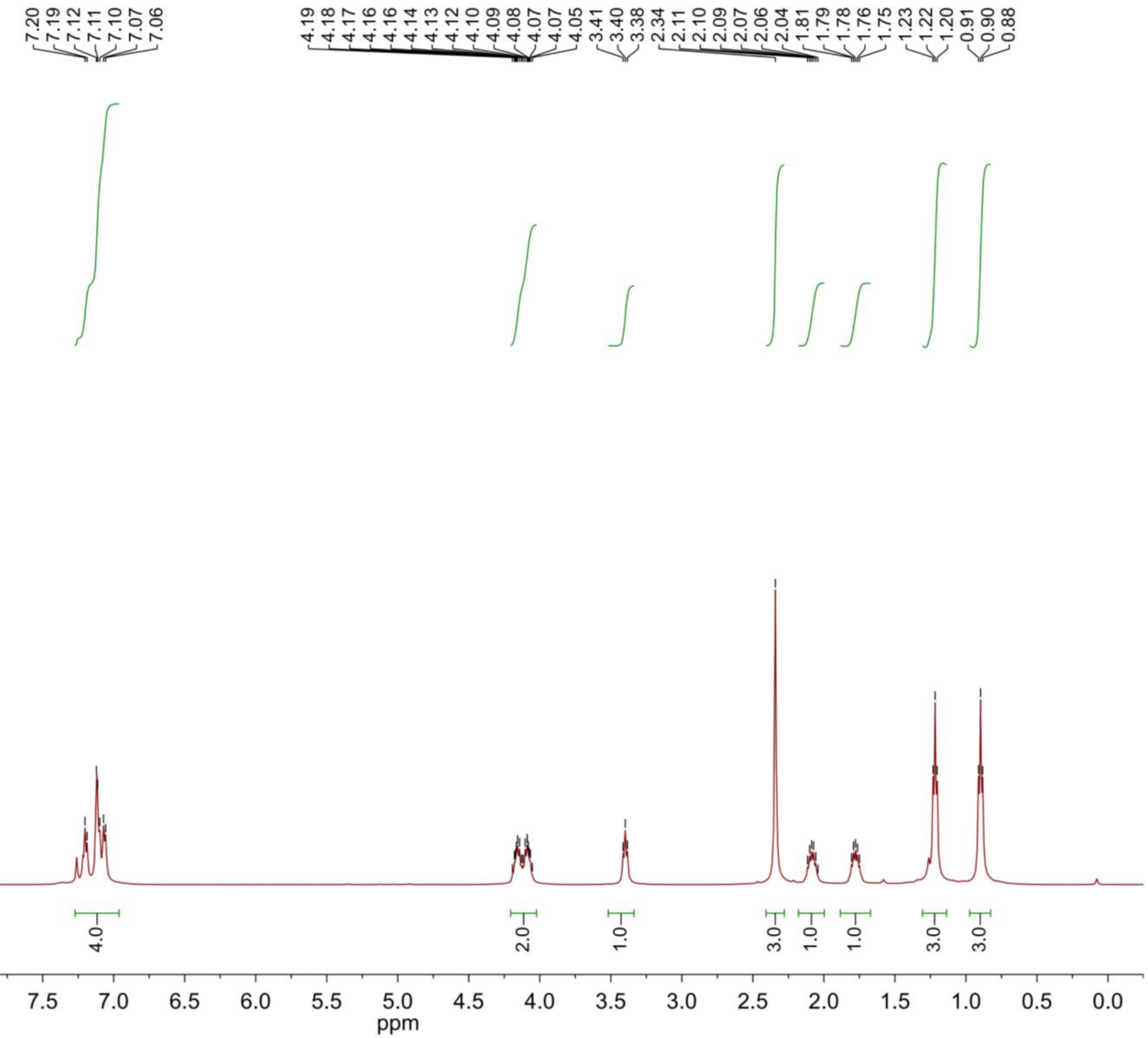

Go back to table of contents

Ethyl 2-(*m*-tolyl)butanoate (12)

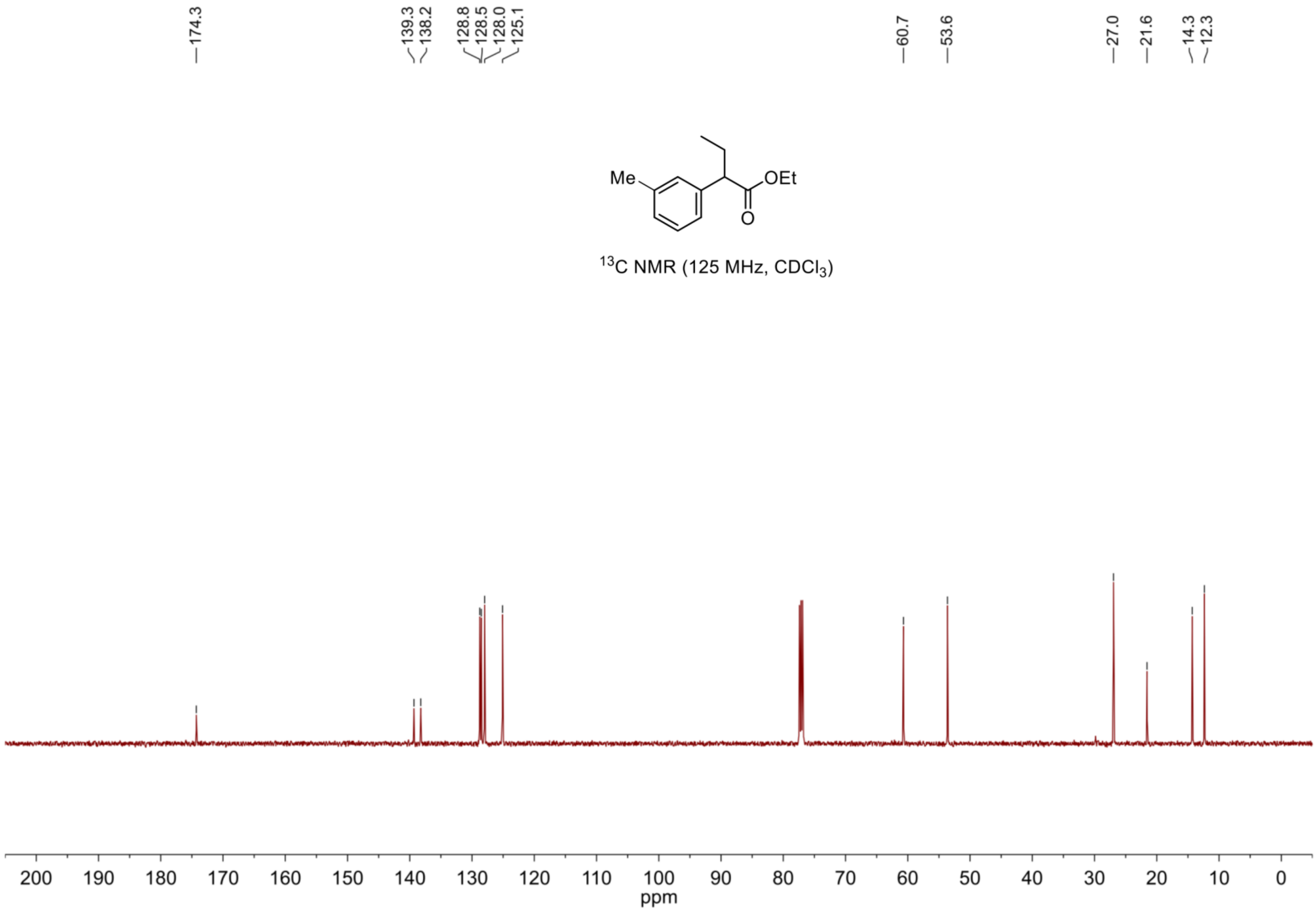

Go back to table of contents

Ethyl 2-(4-isobutylphenyl)butanoate (13)

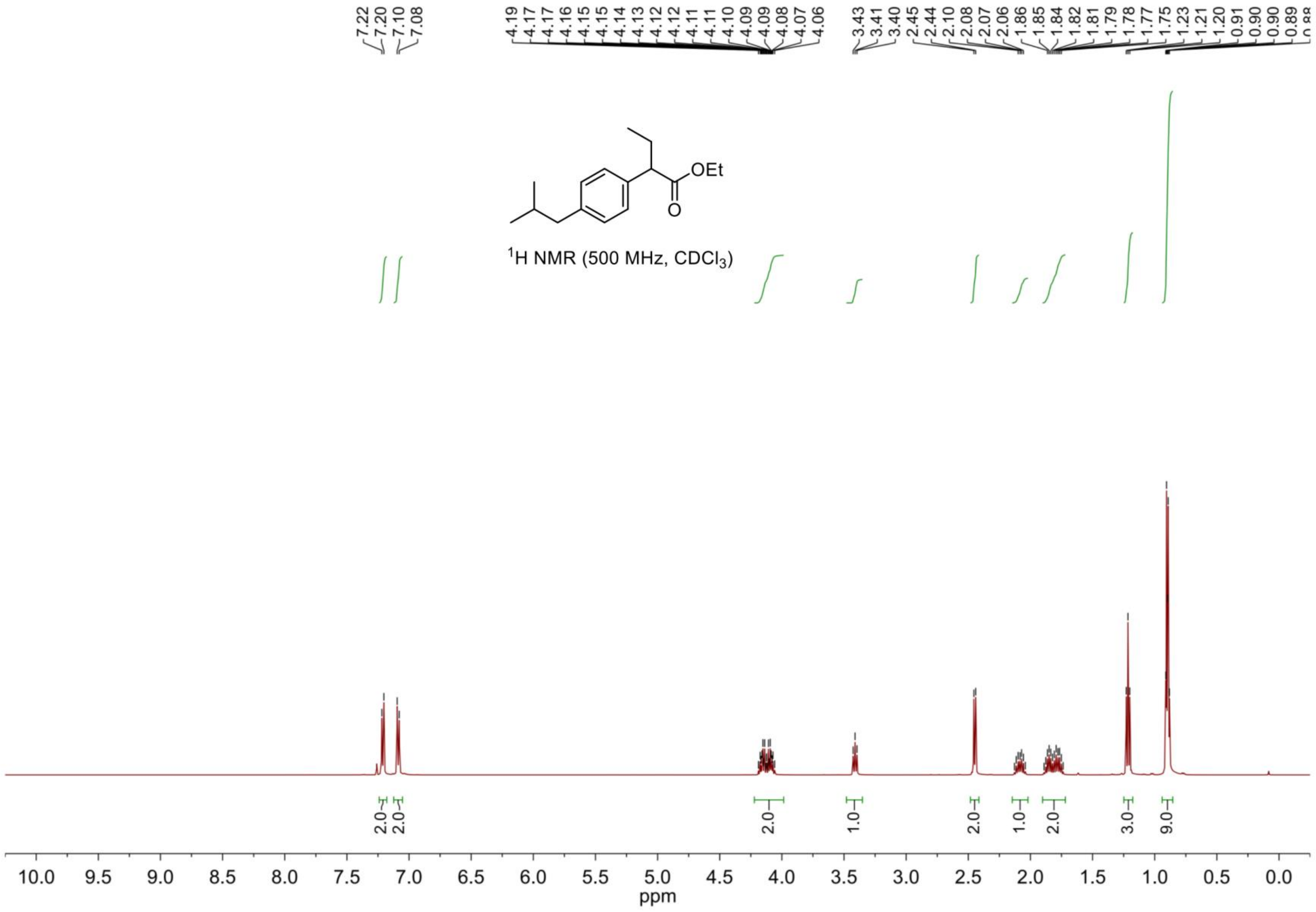

Go back to table of contents

Ethyl 2-(4-isobutylphenyl)butanoate (13)

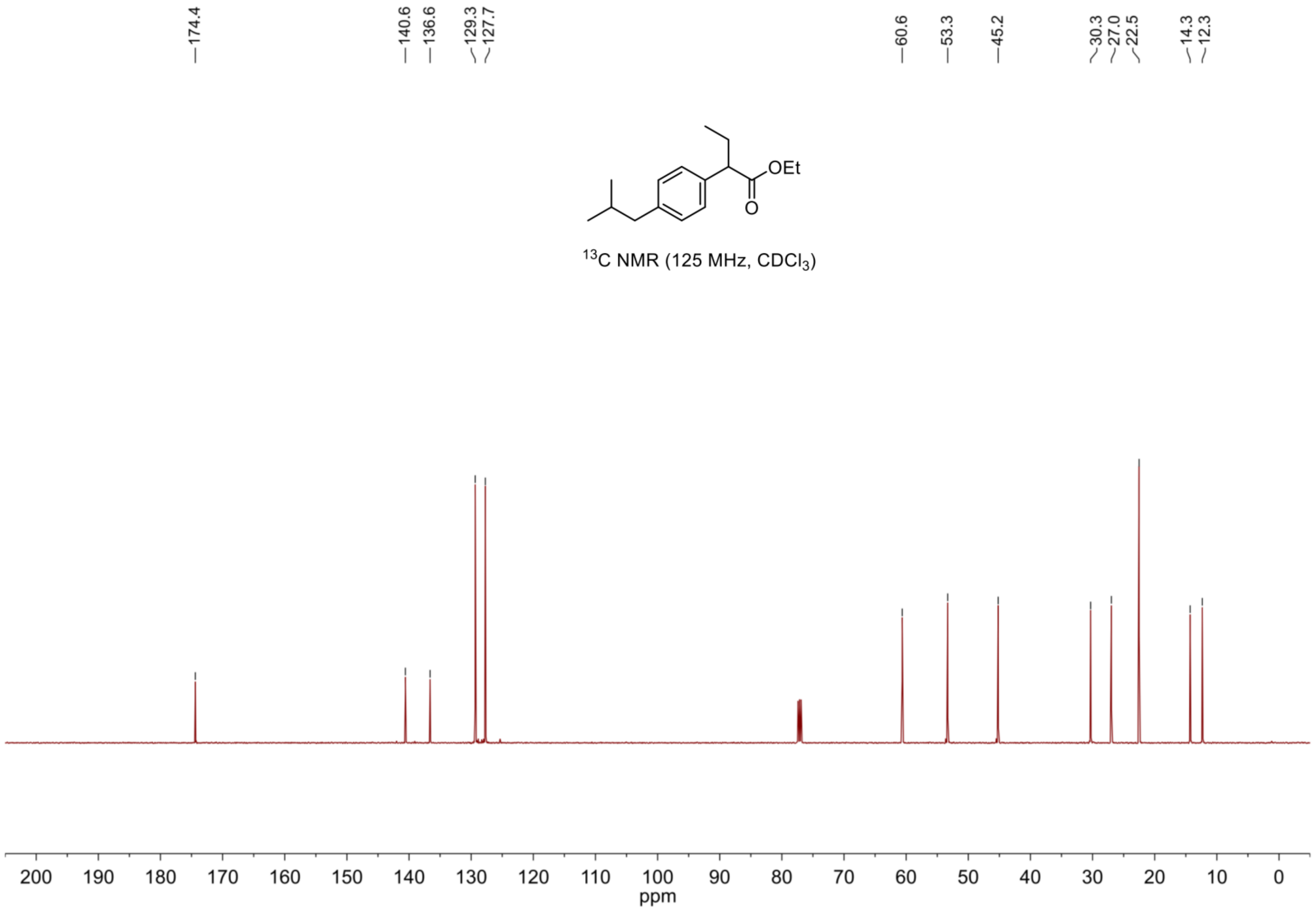

Go back to table of contents

Ethyl 2-(4-methoxyphenyl)butanoate (14)

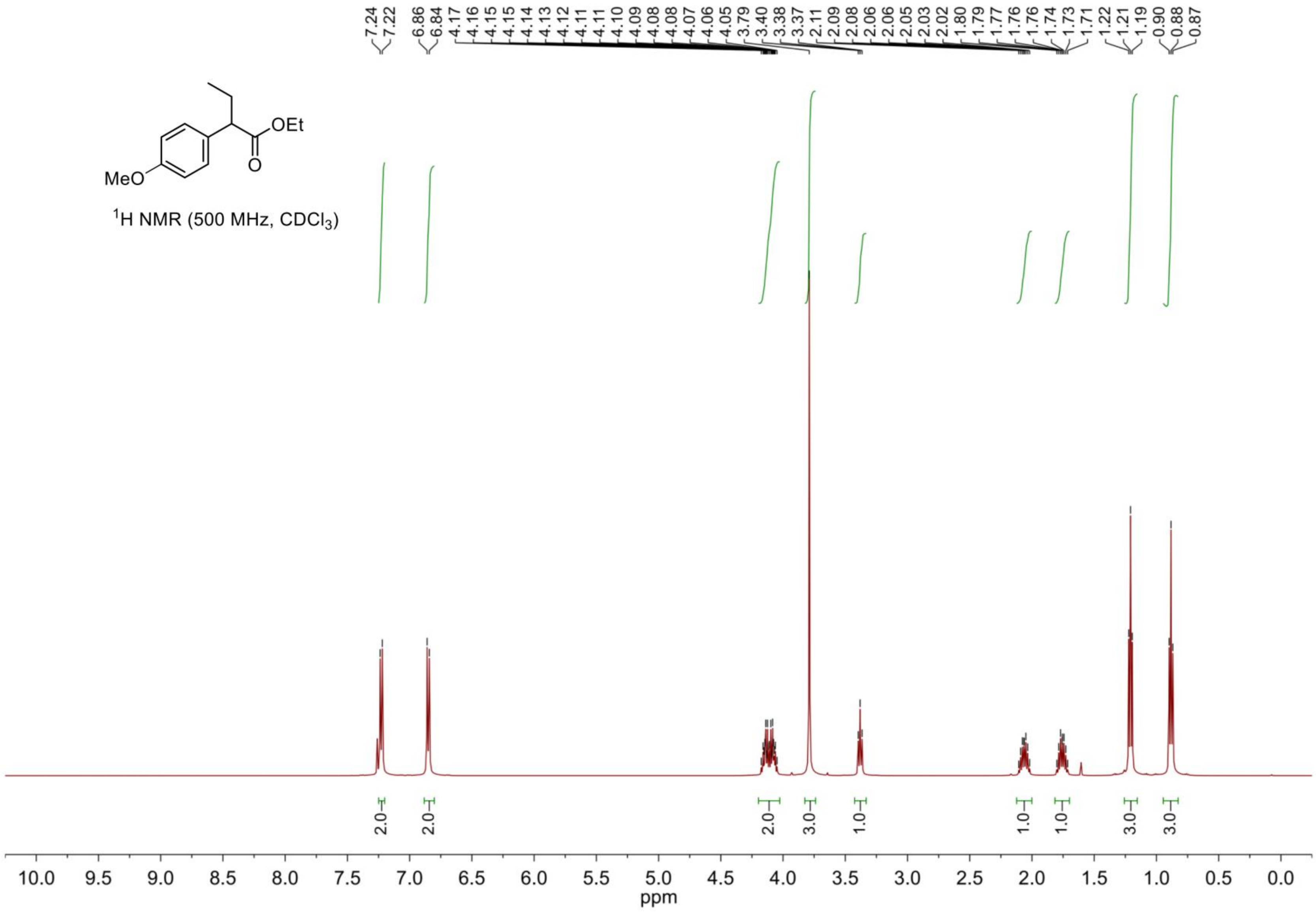

Go back to table of contents

Ethyl 2-(4-methoxyphenyl)butanoate (14)

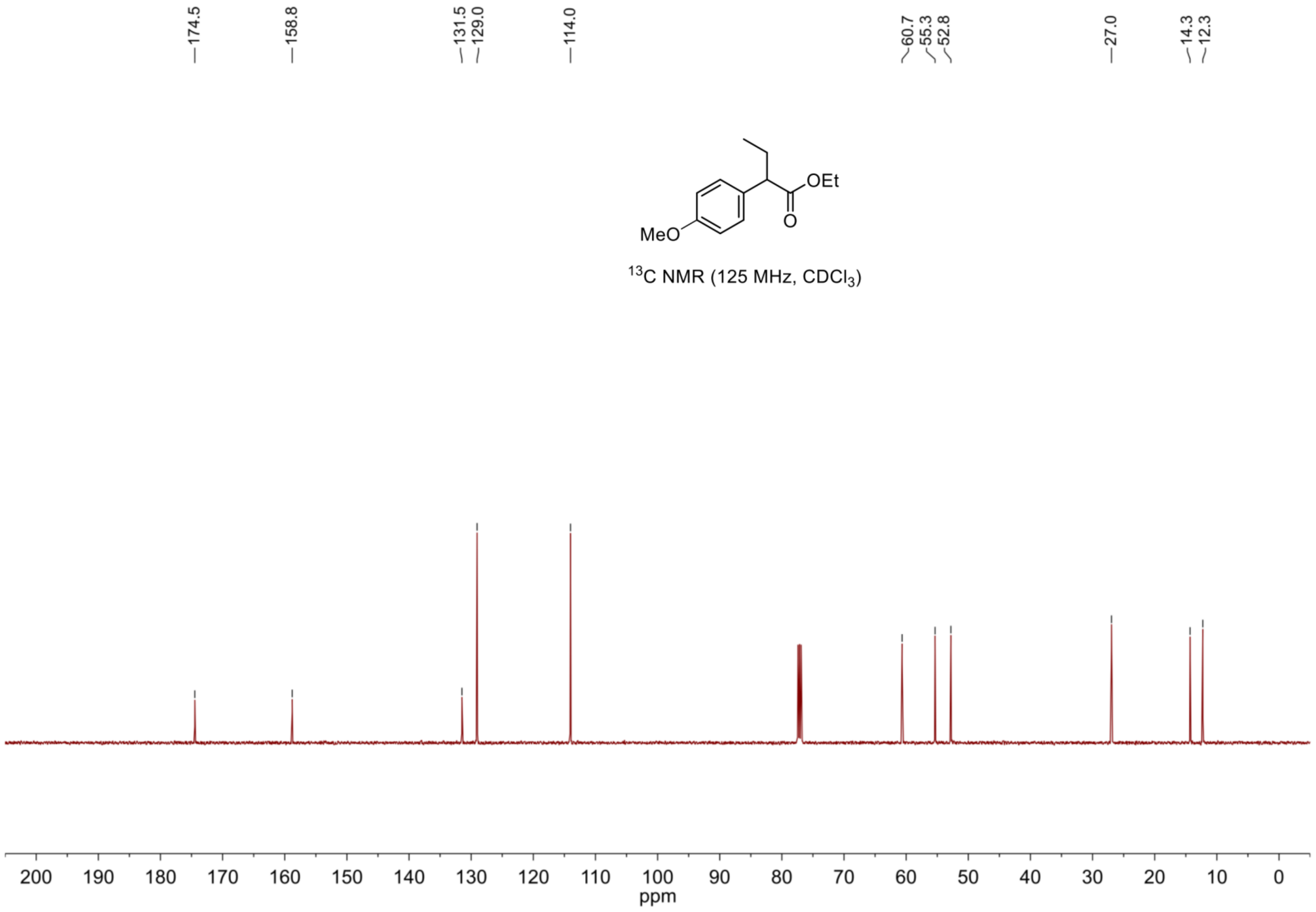

Go back to table of contents

Ethyl 2-(3,4-dimethoxyphenyl)butanoate (15)

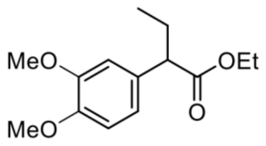

^{1}H NMR (500 MHz, $CDCl_3$)

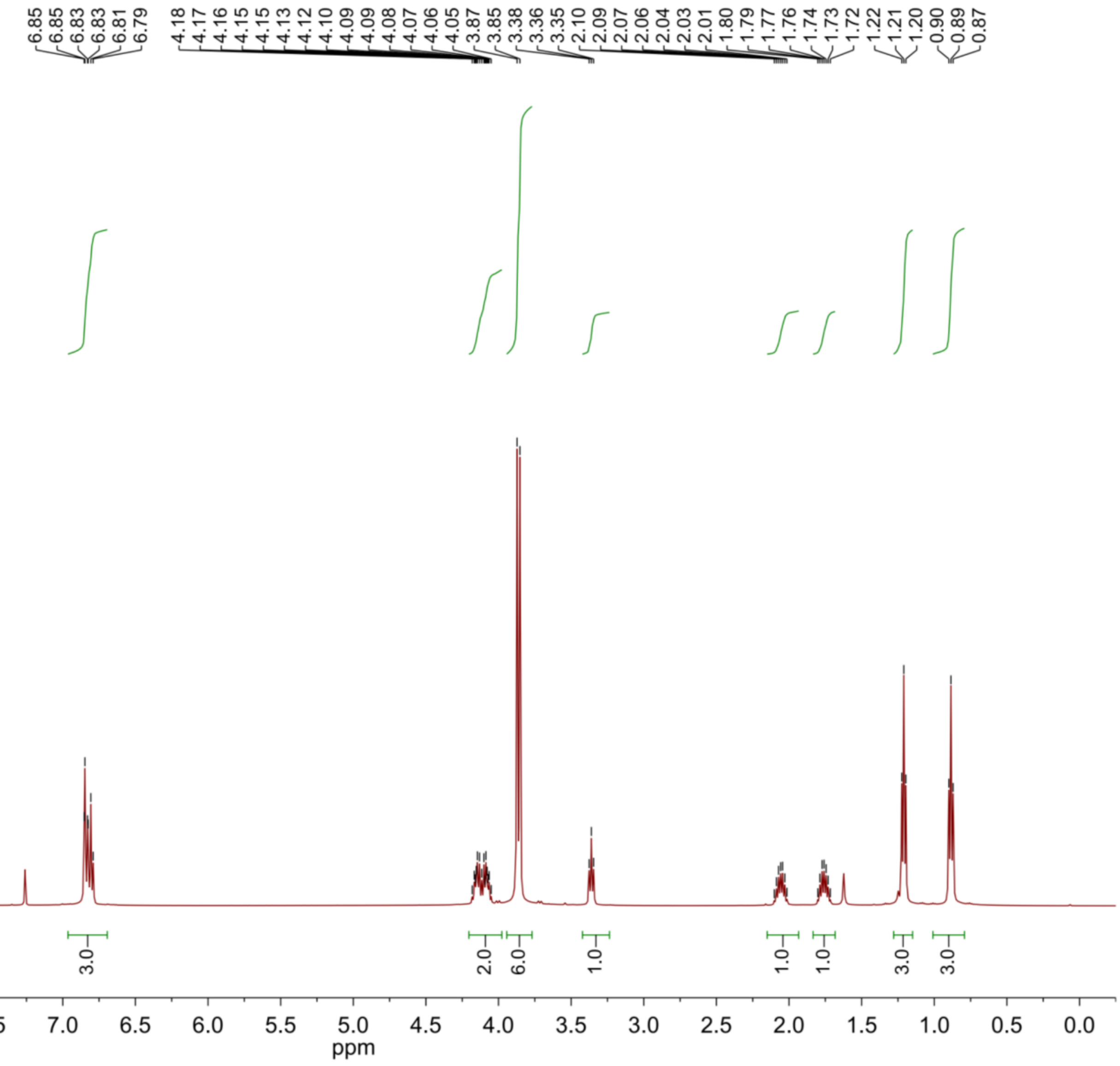

Go back to table of contents

Ethyl 2-(3,4-dimethoxyphenyl)butanoate (15)

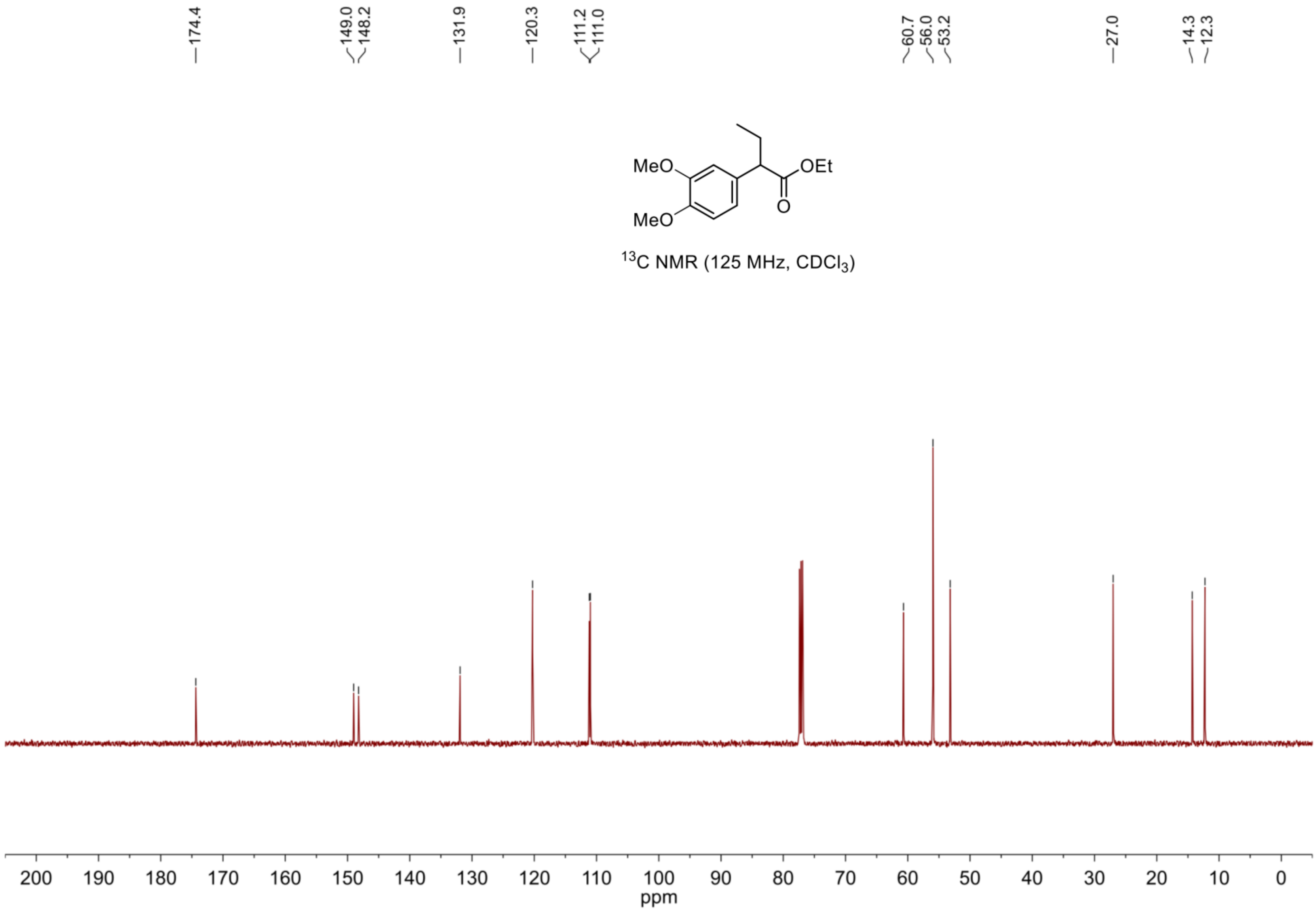

Go back to table of contents

Ethyl 2-(3-fluoro-4-methoxyphenyl)butanoate (16)

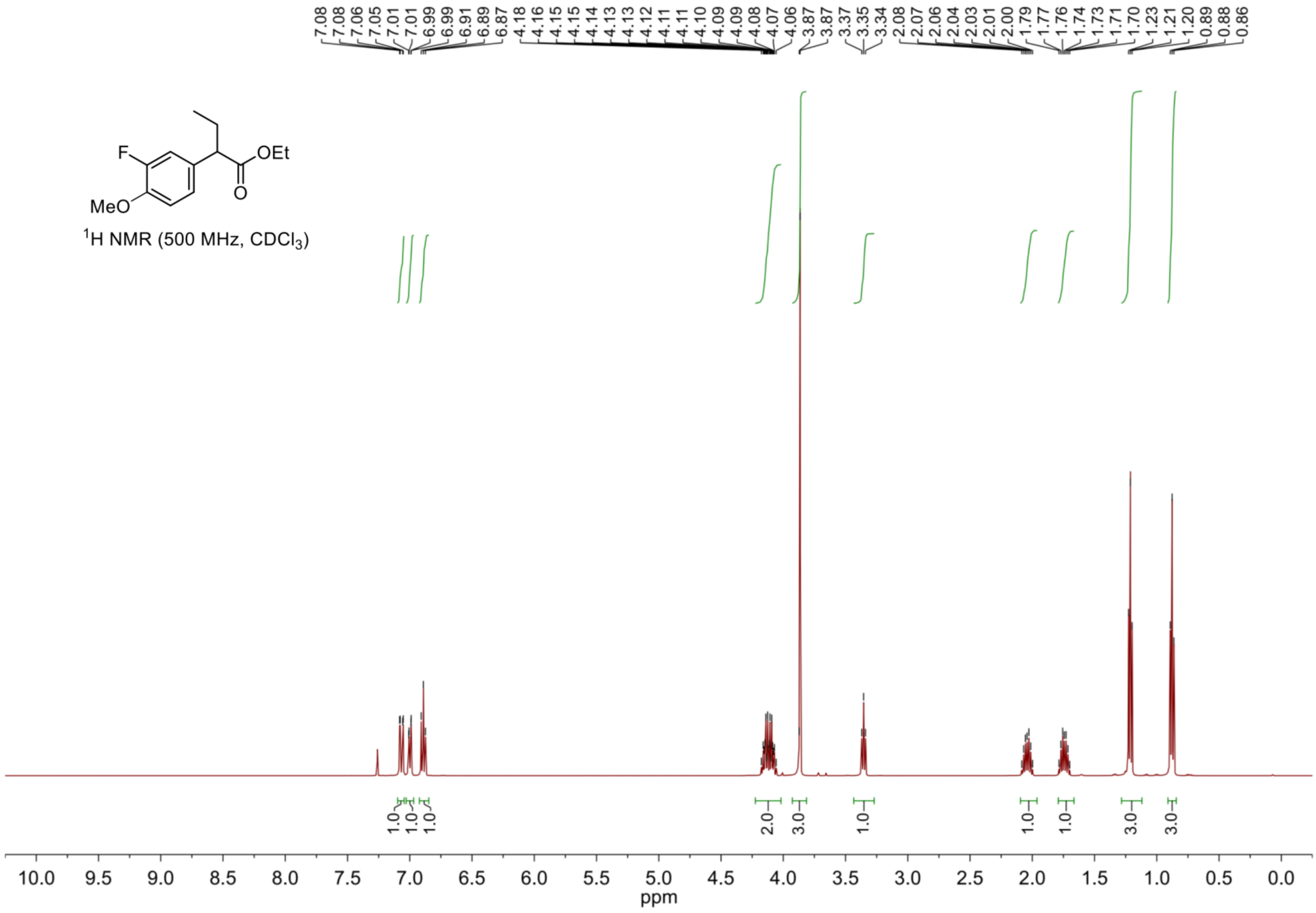

Go back to table of contents

Ethyl 2-(3-fluoro-4-methoxyphenyl)butanoate (16)

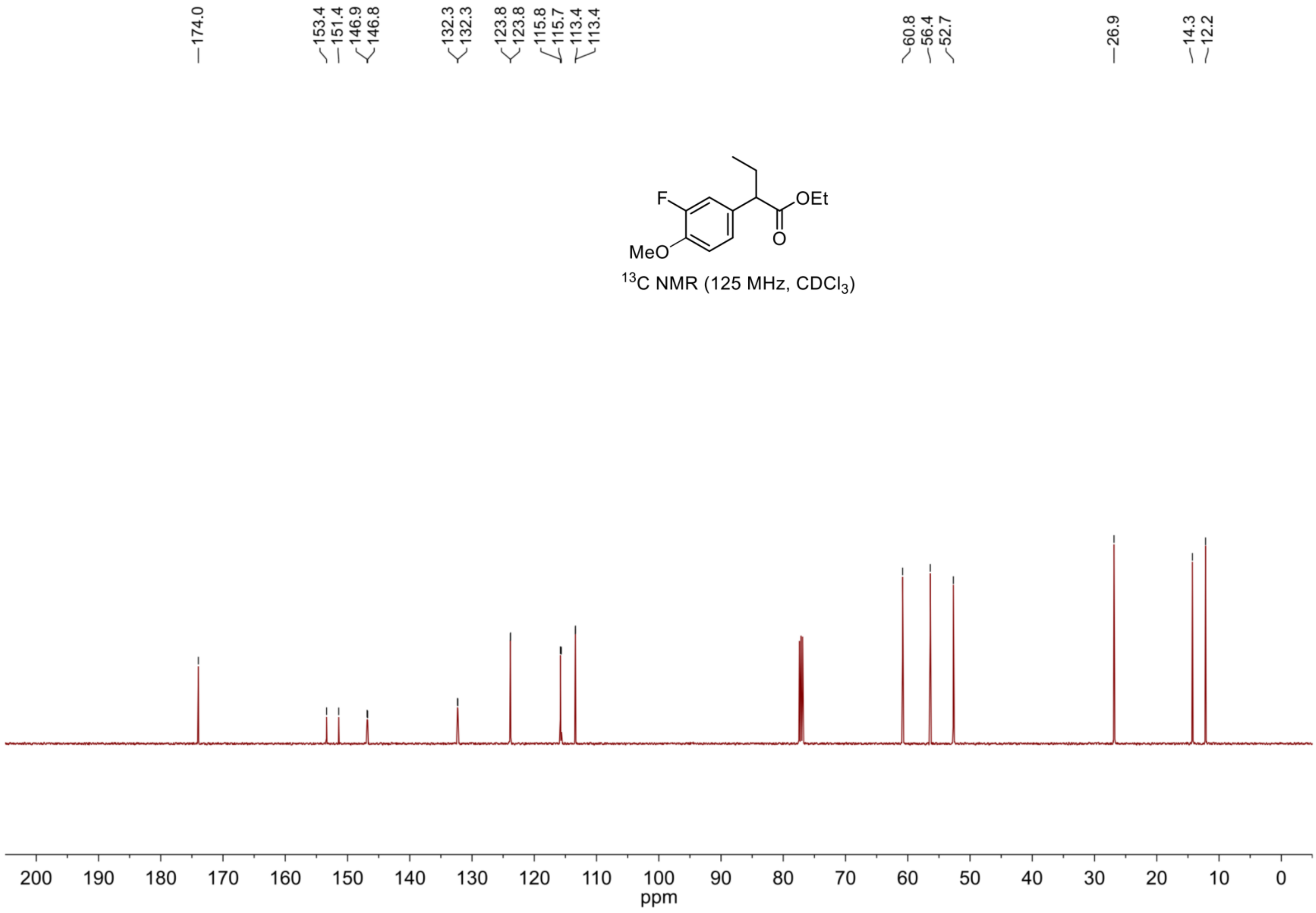

Go back to table of contents

Ethyl 2-(2-fluoro-4,5-dimethoxyphenyl)butanoate (17)

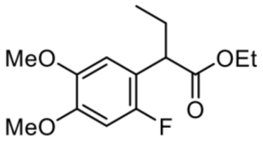

^{1}H NMR (500 MHz, $CDCl_3$)

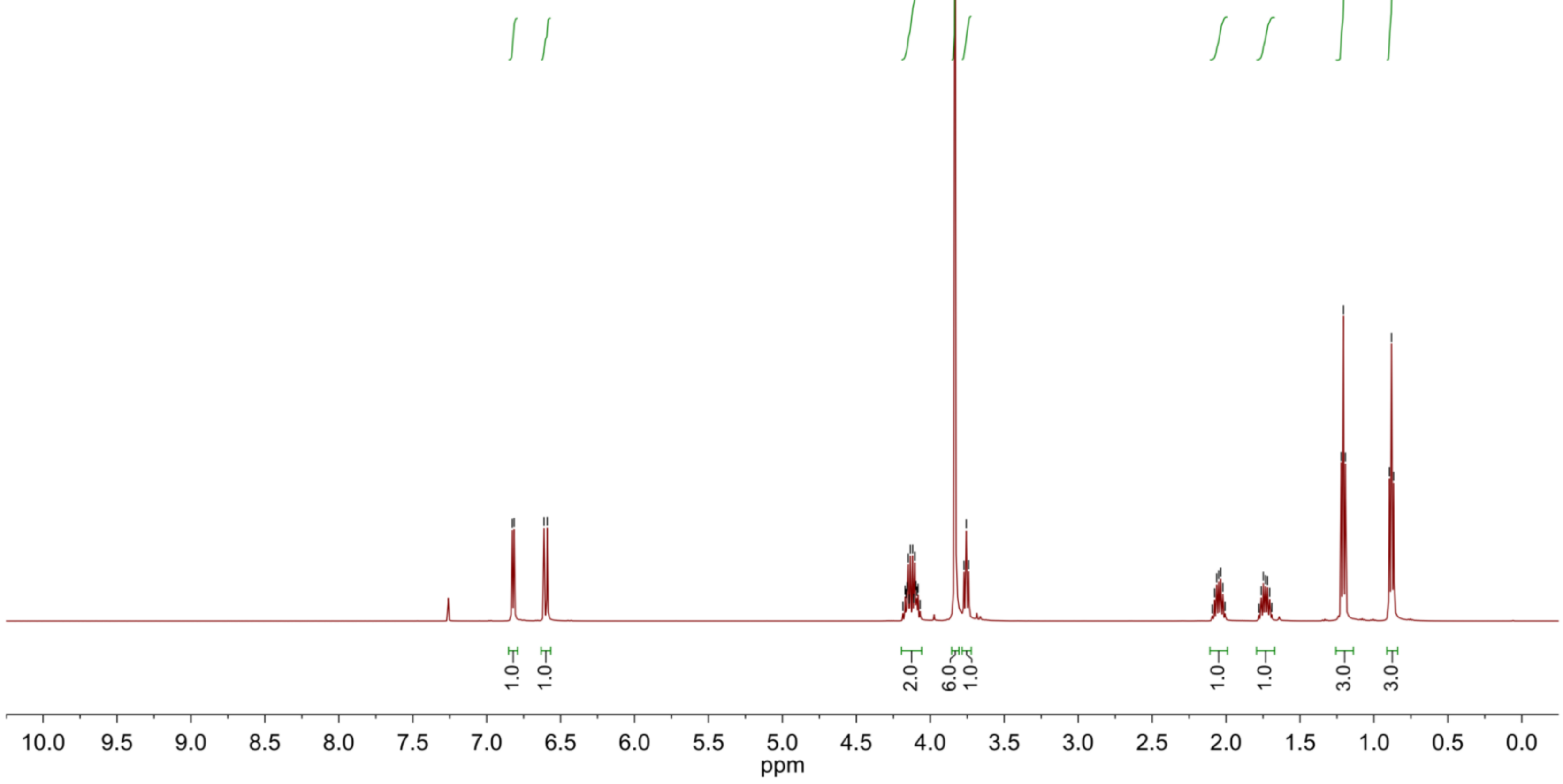

Ethyl 2-(2-fluoro-4,5-dimethoxyphenyl)butanoate (17)

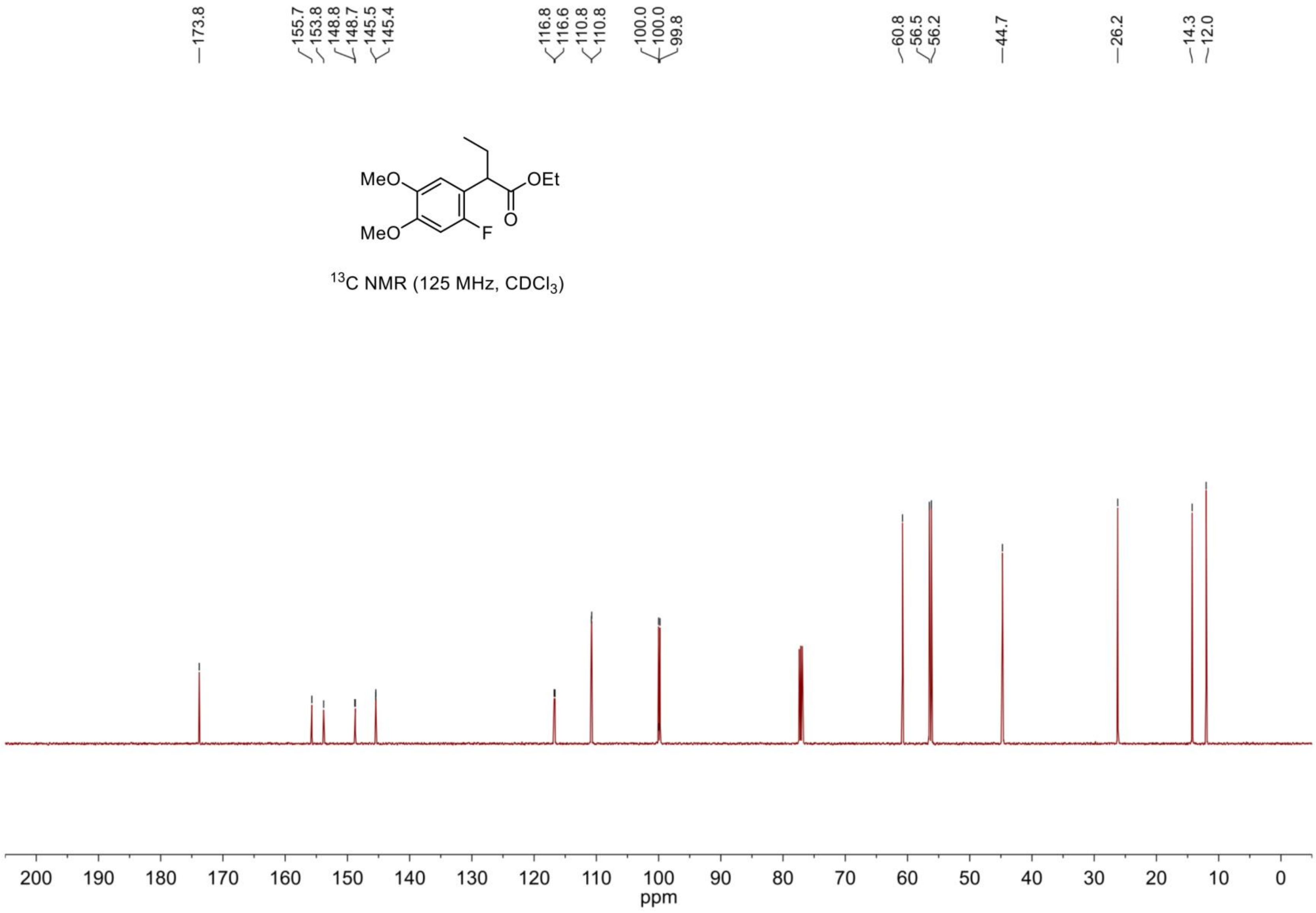

Go back to table of contents

Ethyl 2-(4-(methylthio)phenyl)butanoate (18)

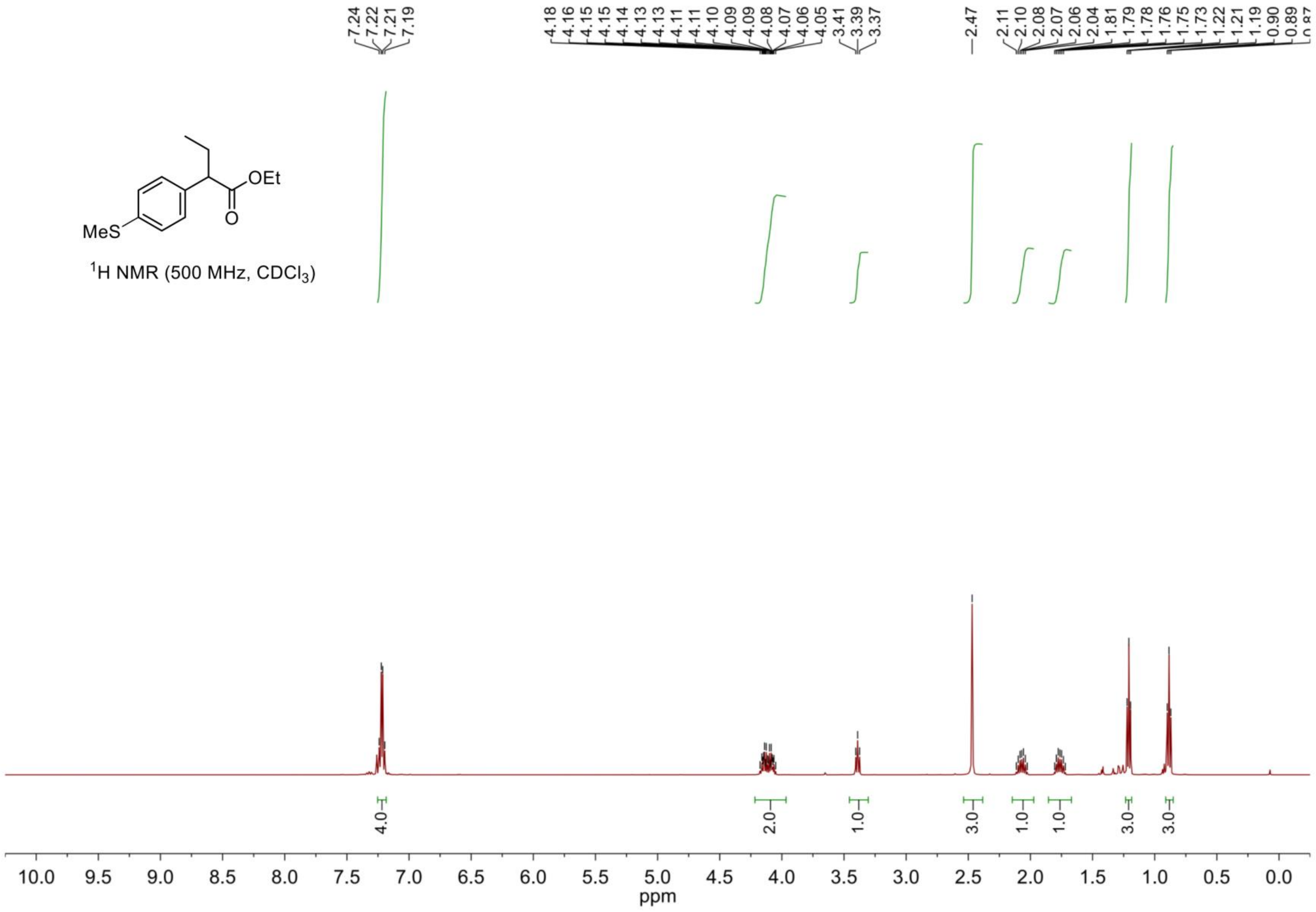

Go back to table of contents

Ethyl 2-(4-(methylthio)phenyl)butanoate (18)

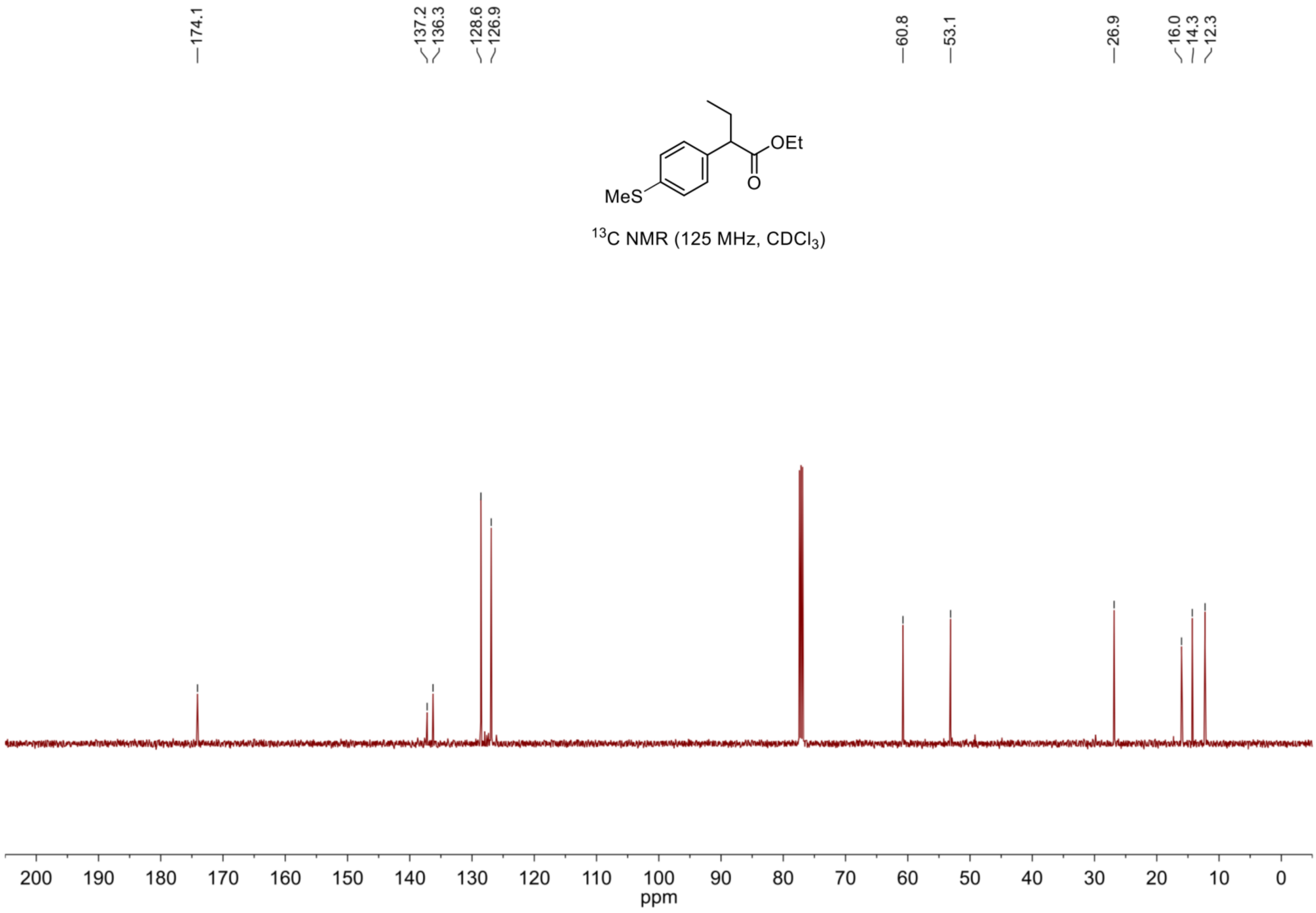

Go back to table of contents

Ethyl 2-(4-cyanophenyl)butanoate (19)

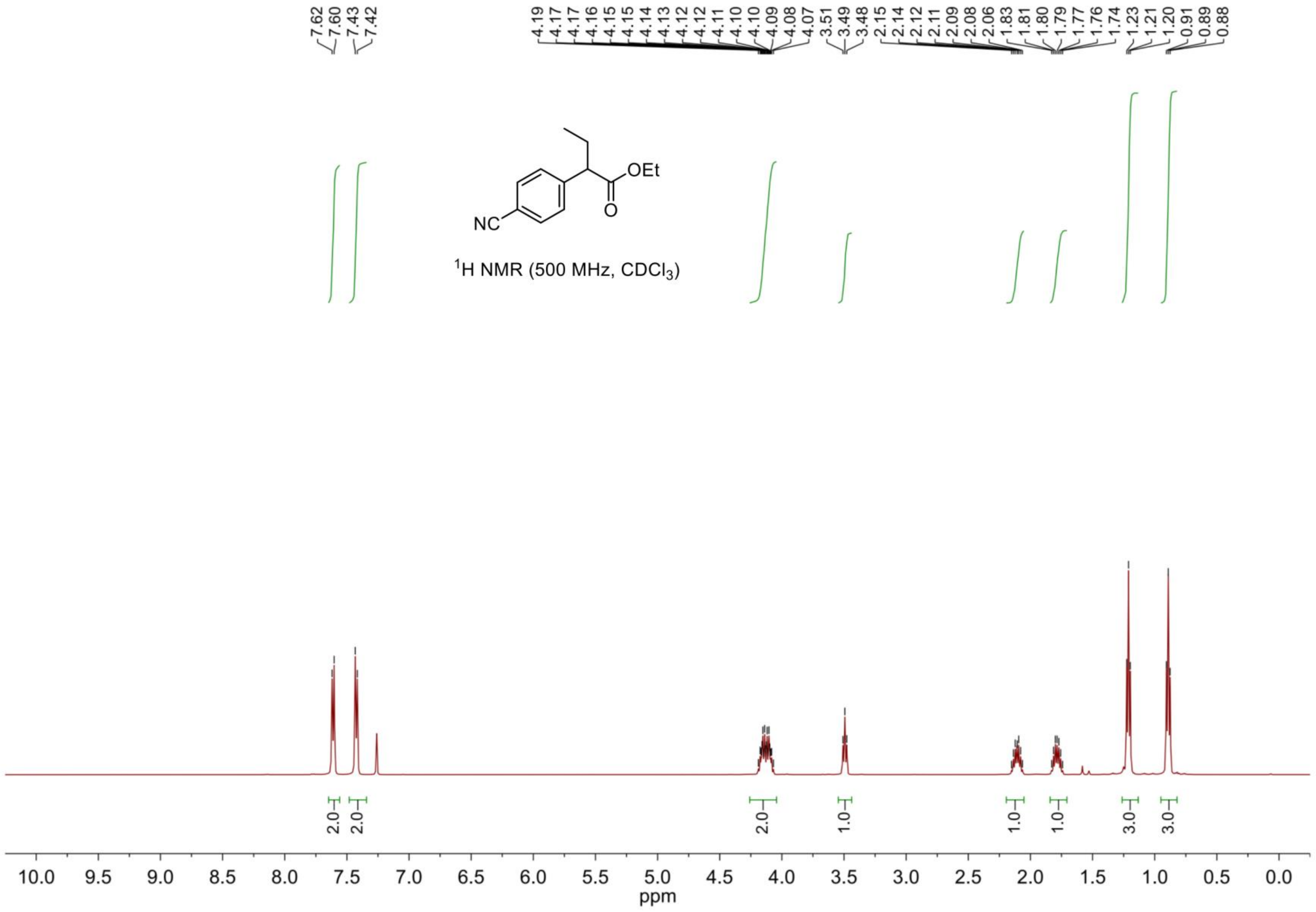

Go back to table of contents

Ethyl 2-(4-cyanophenyl)butanoate (19)

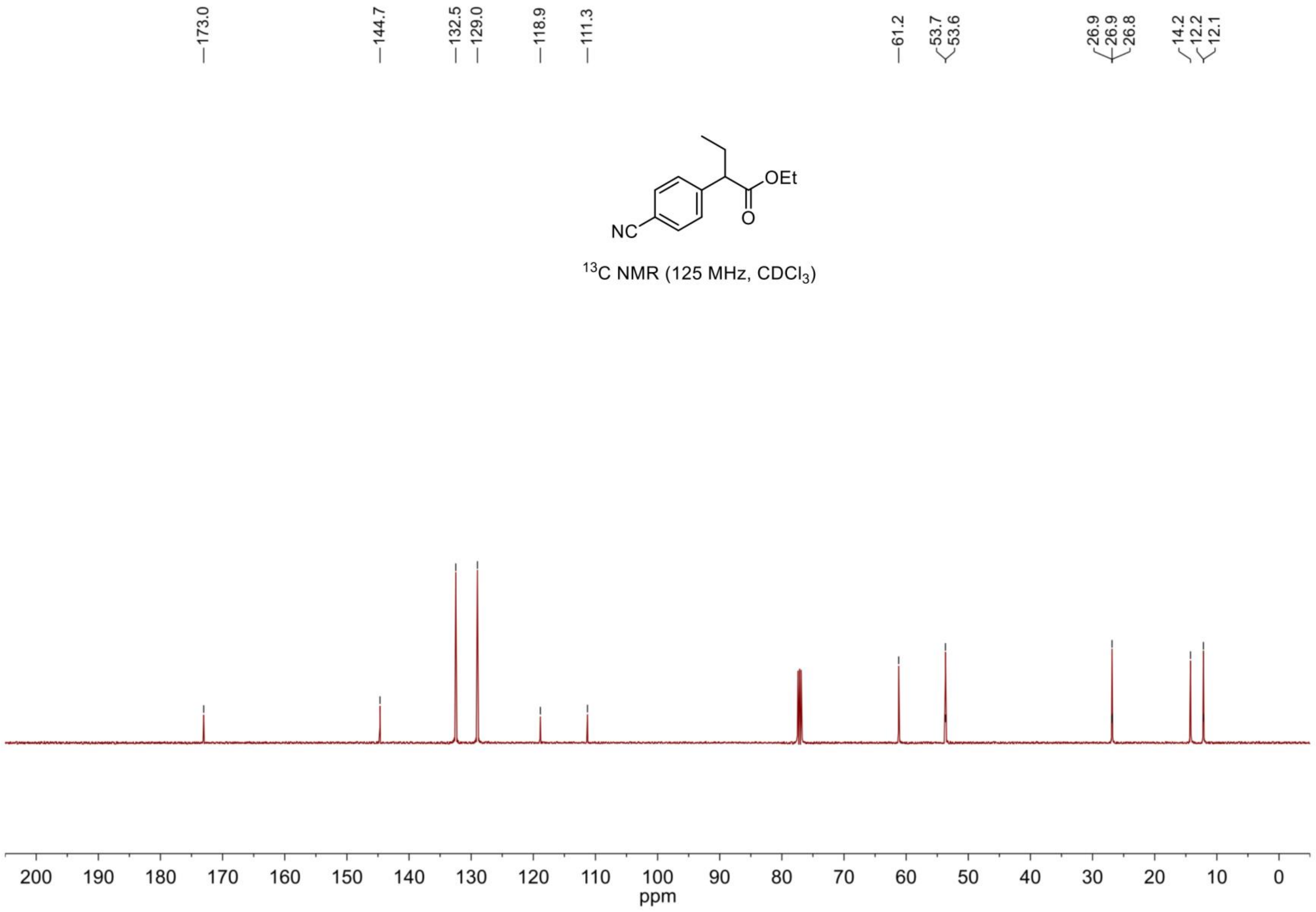

Go back to table of contents

Ethyl 2-(3-(trifluoromethyl)phenyl)butanoate (20)

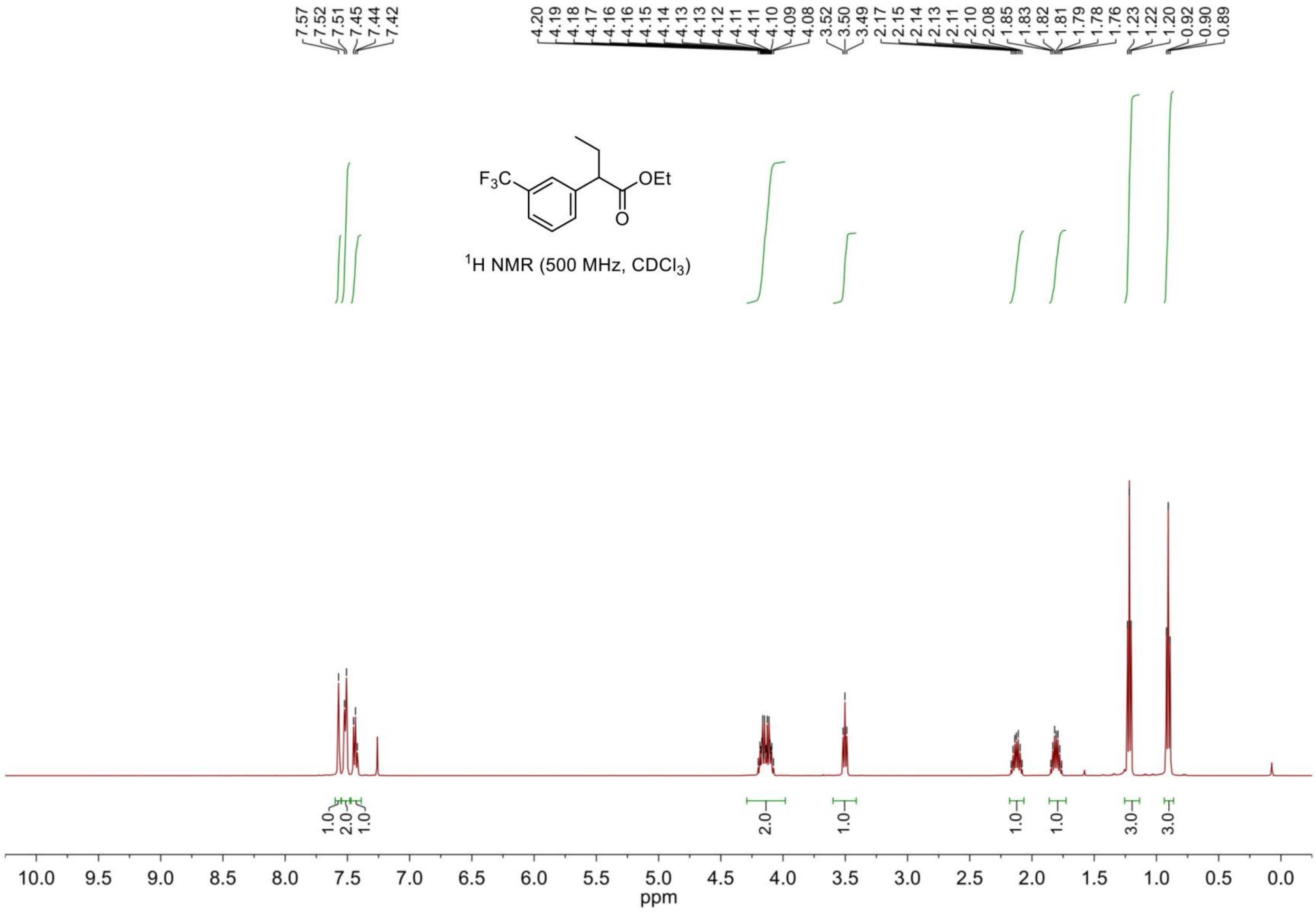

Go back to table of contents

Ethyl 2-(3-(trifluoromethyl)phenyl)butanoate (20)

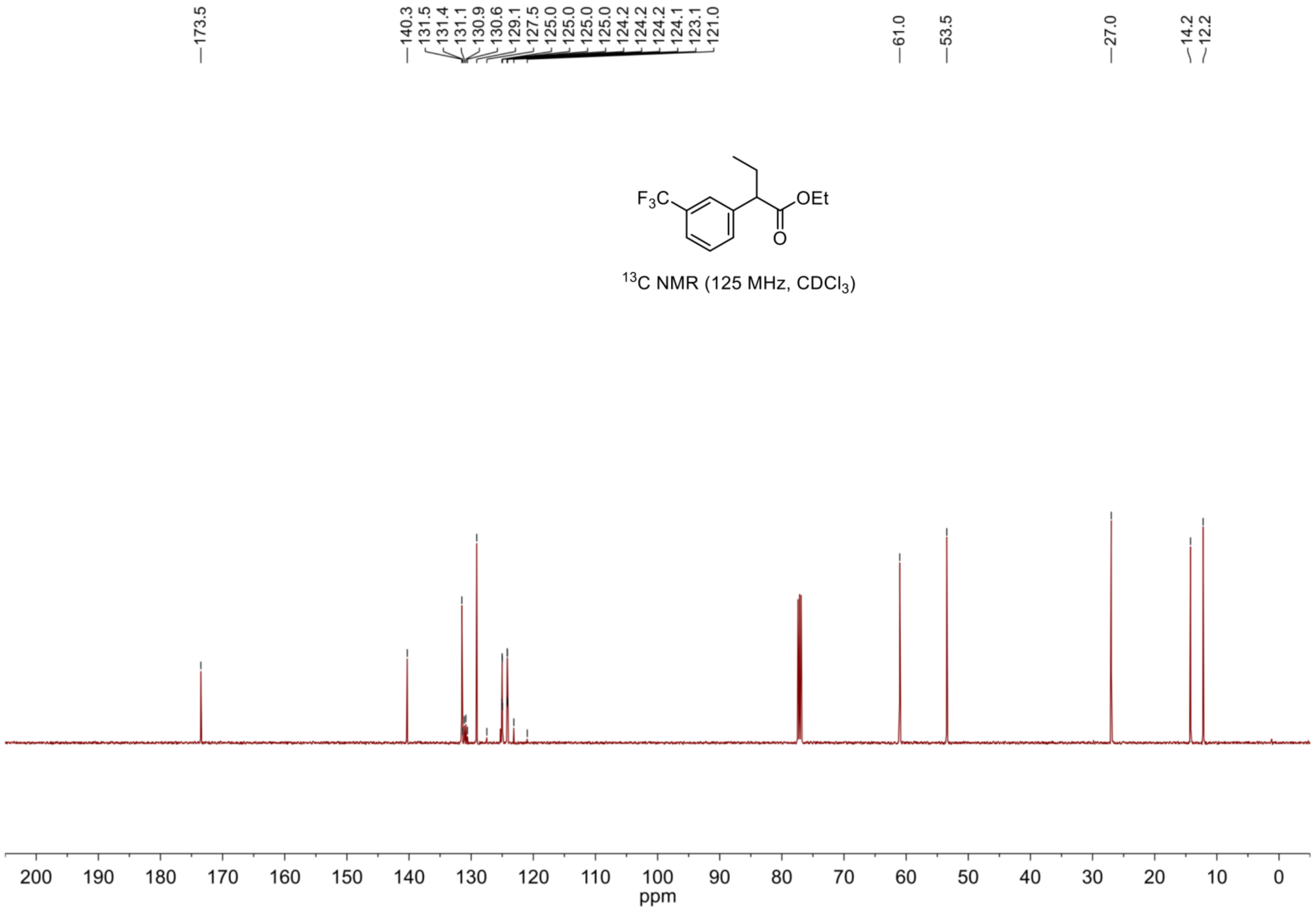

Go back to table of contents

Ethyl 2-(3-(trifluoromethoxy)phenyl)butanoate (21)

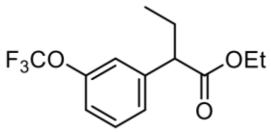

^{1}H NMR (500 MHz, $CDCl_3$)

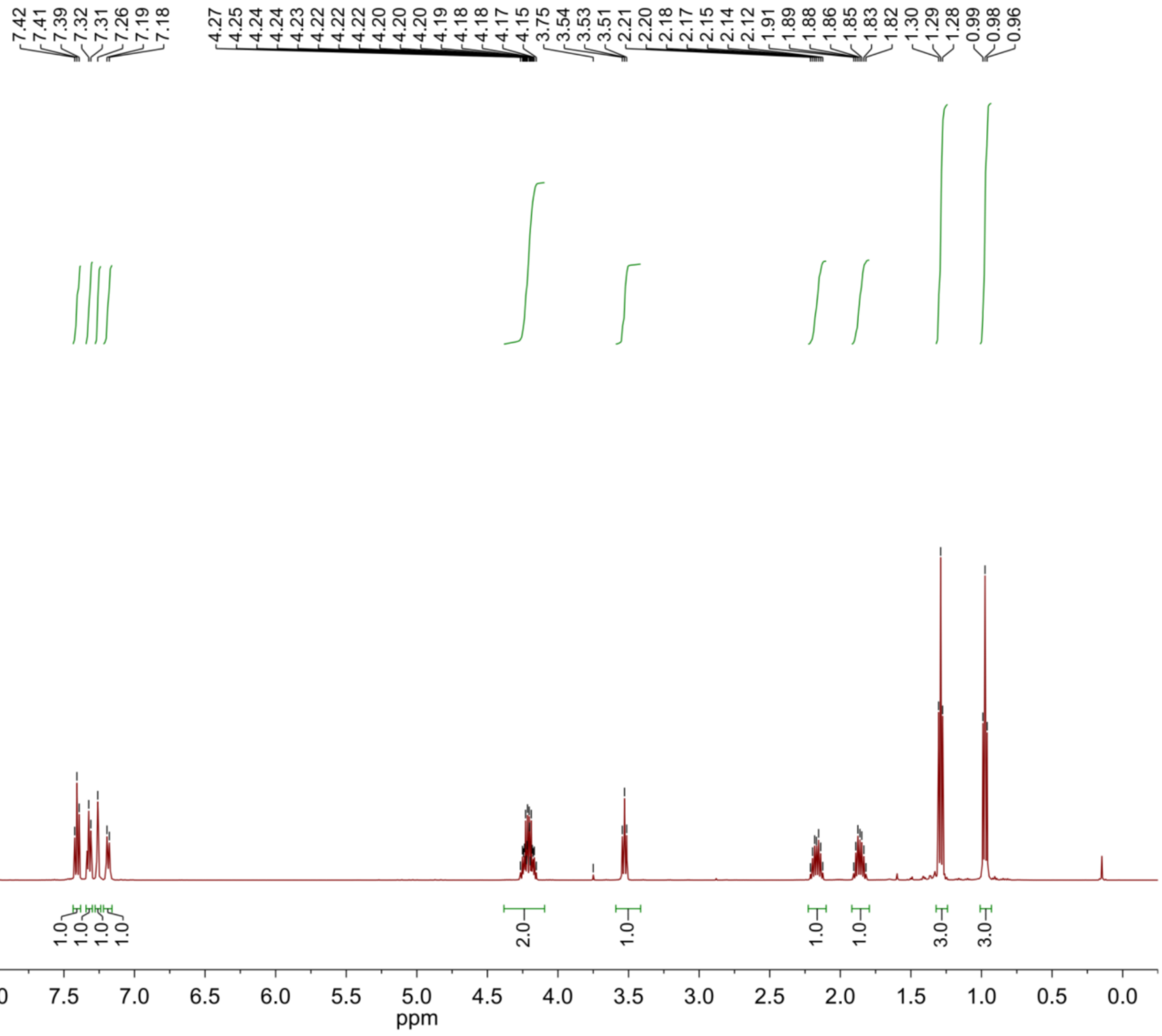

Go back to table of contents

Ethyl 2-(3-(trifluoromethoxy)phenyl)butanoate (21)

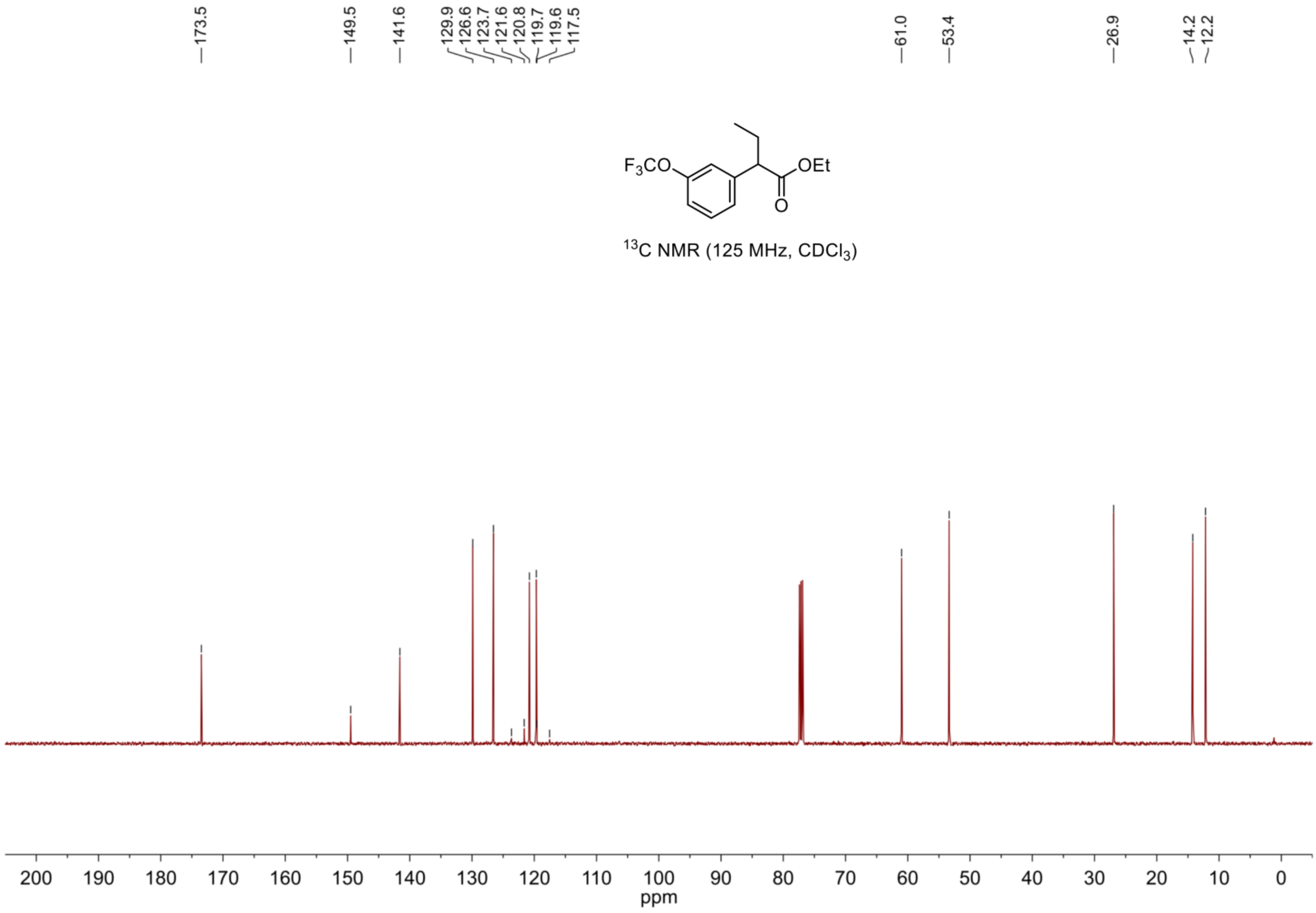

Go back to table of contents

Ethyl 2-([1,1'-biphenyl]-4-yl)butanoate (22)

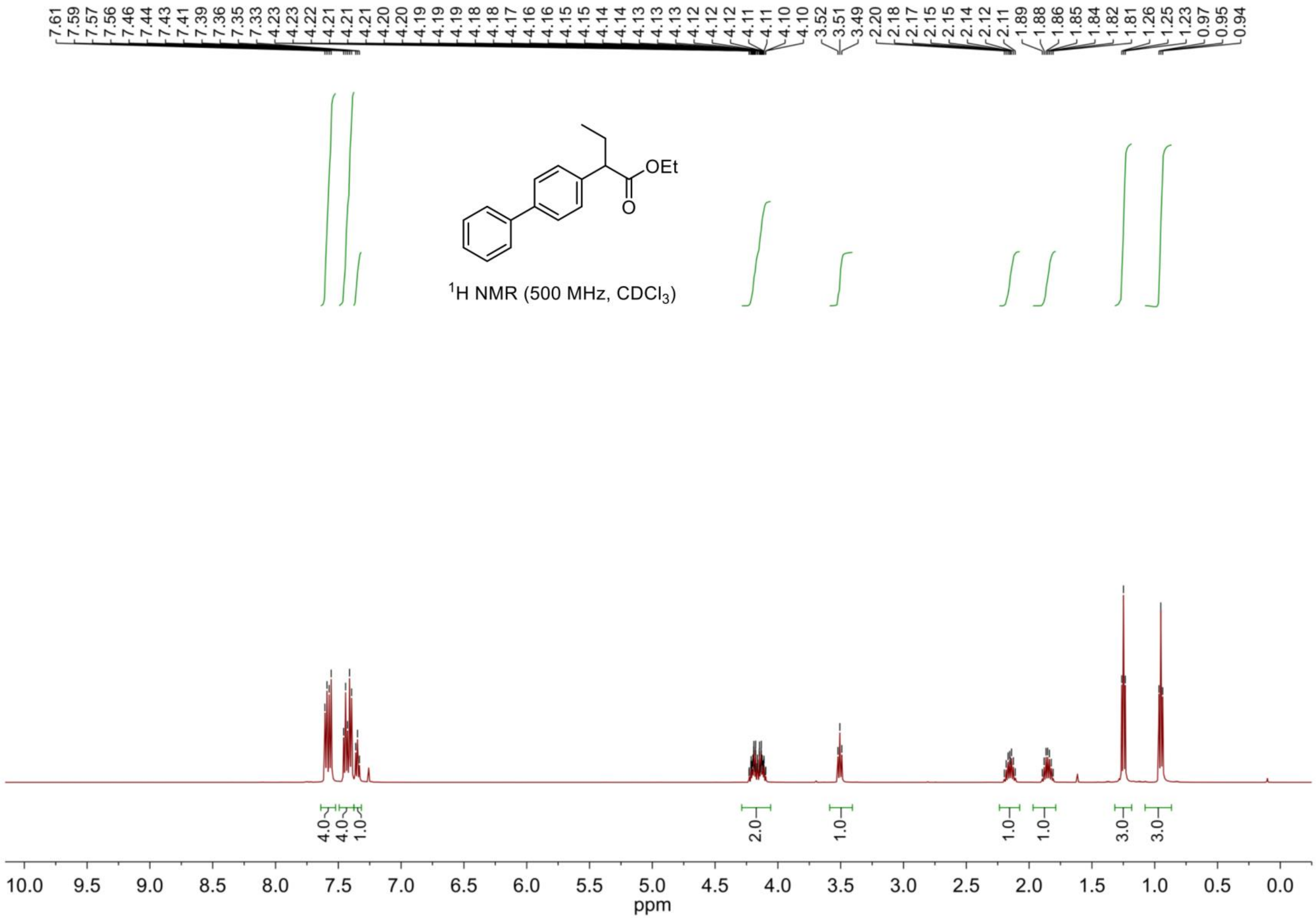

Go back to table of contents

Ethyl 2-([1,1'-biphenyl]-4-yl)butanoate (22)

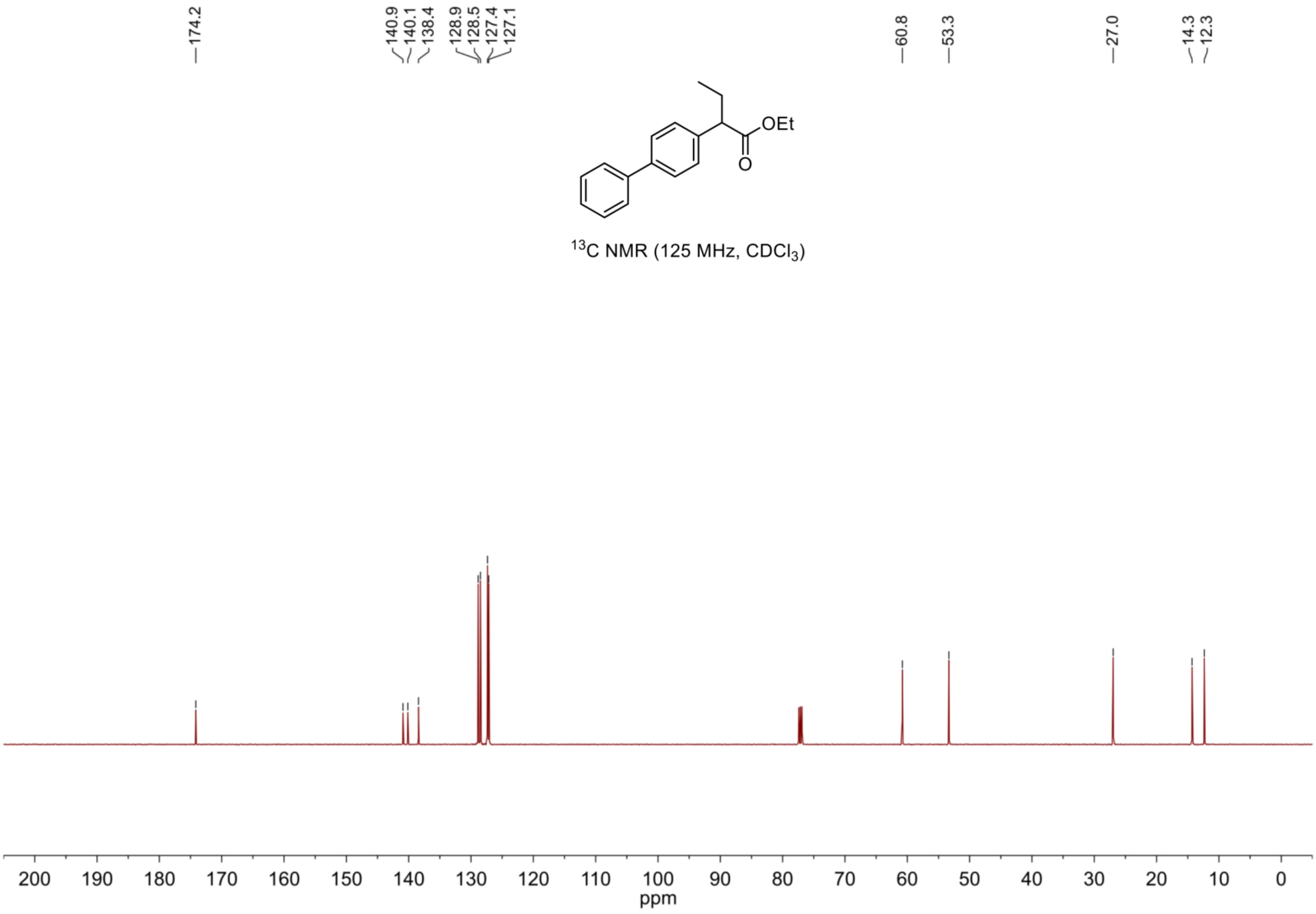

Go back to table of contents

Ethyl 2-(2-fluoro-[1,1'-biphenyl]-4-yl)butanoate (23)

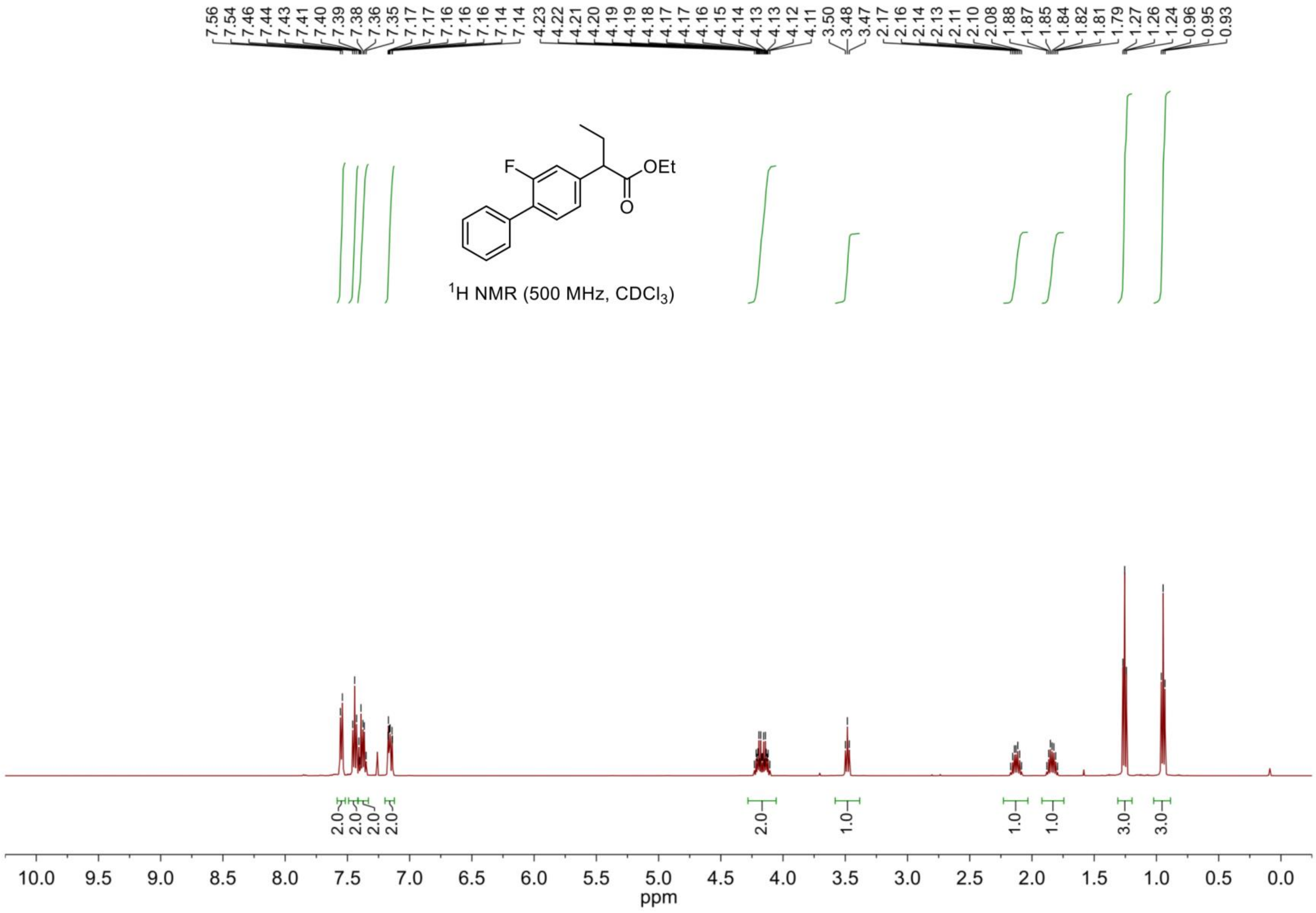

Go back to table of contents

Ethyl 2-(2-fluoro-[1,1'-biphenyl]-4-yl)butanoate (23)

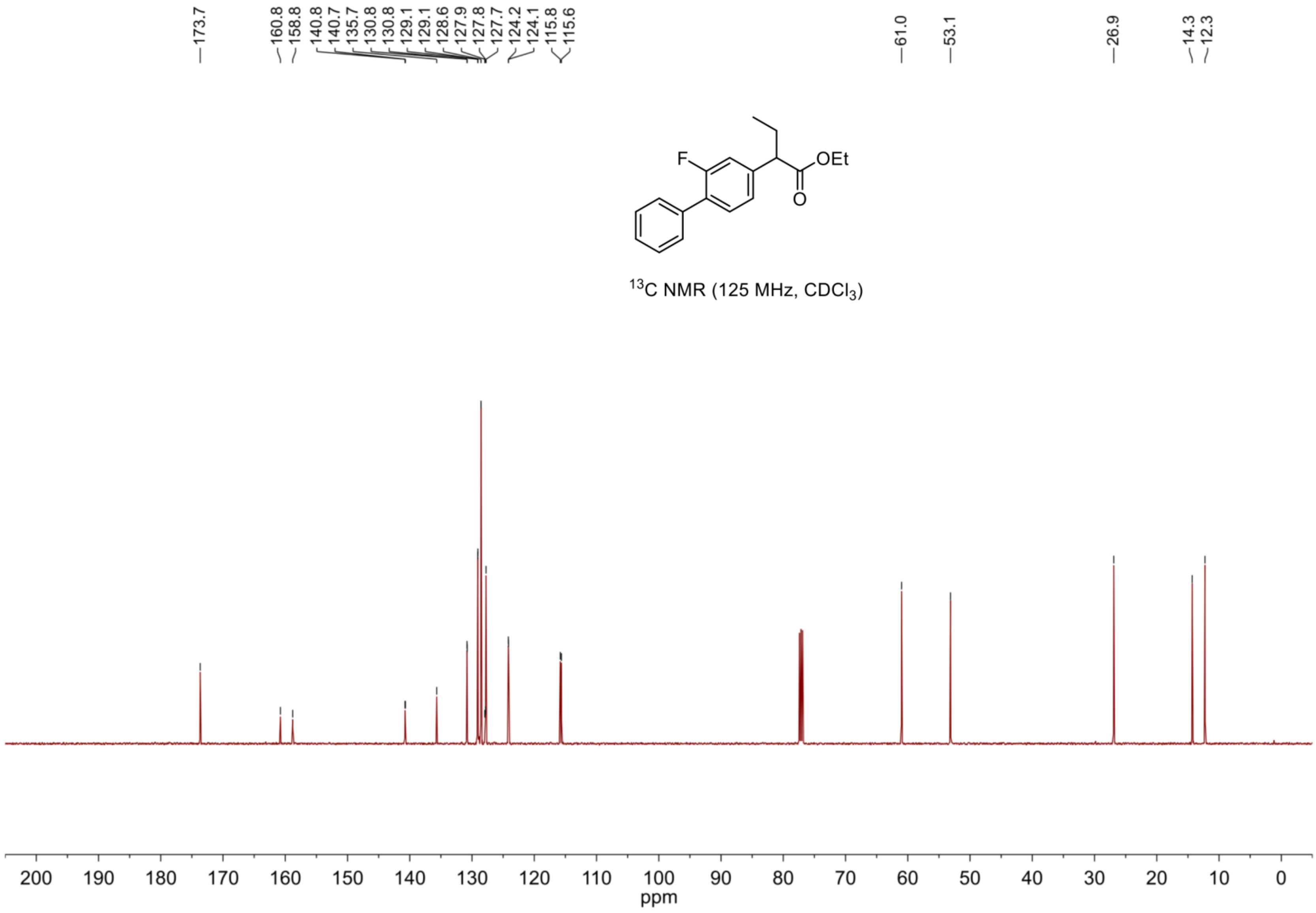

Go back to table of contents

Ethyl 2-(5,6,7,8-tetrahydronaphthalen-2-yl)butanoate (24)

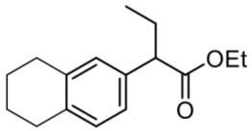

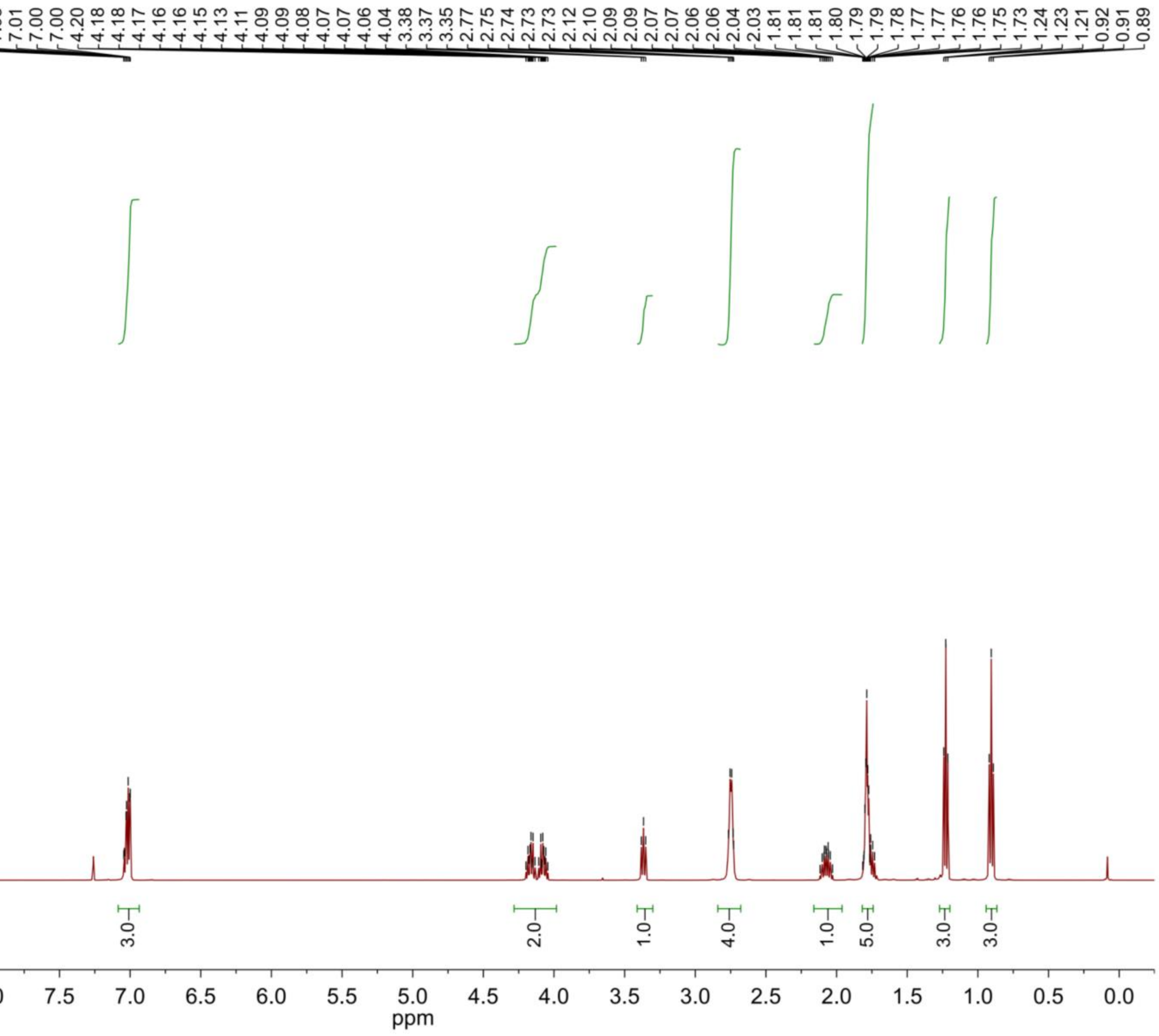

^{1}H NMR (500 MHz, $CDCl_3$)

Go back to table of contents

Ethyl 2-(5,6,7,8-tetrahydronaphthalen-2-yl)butanoate (24)

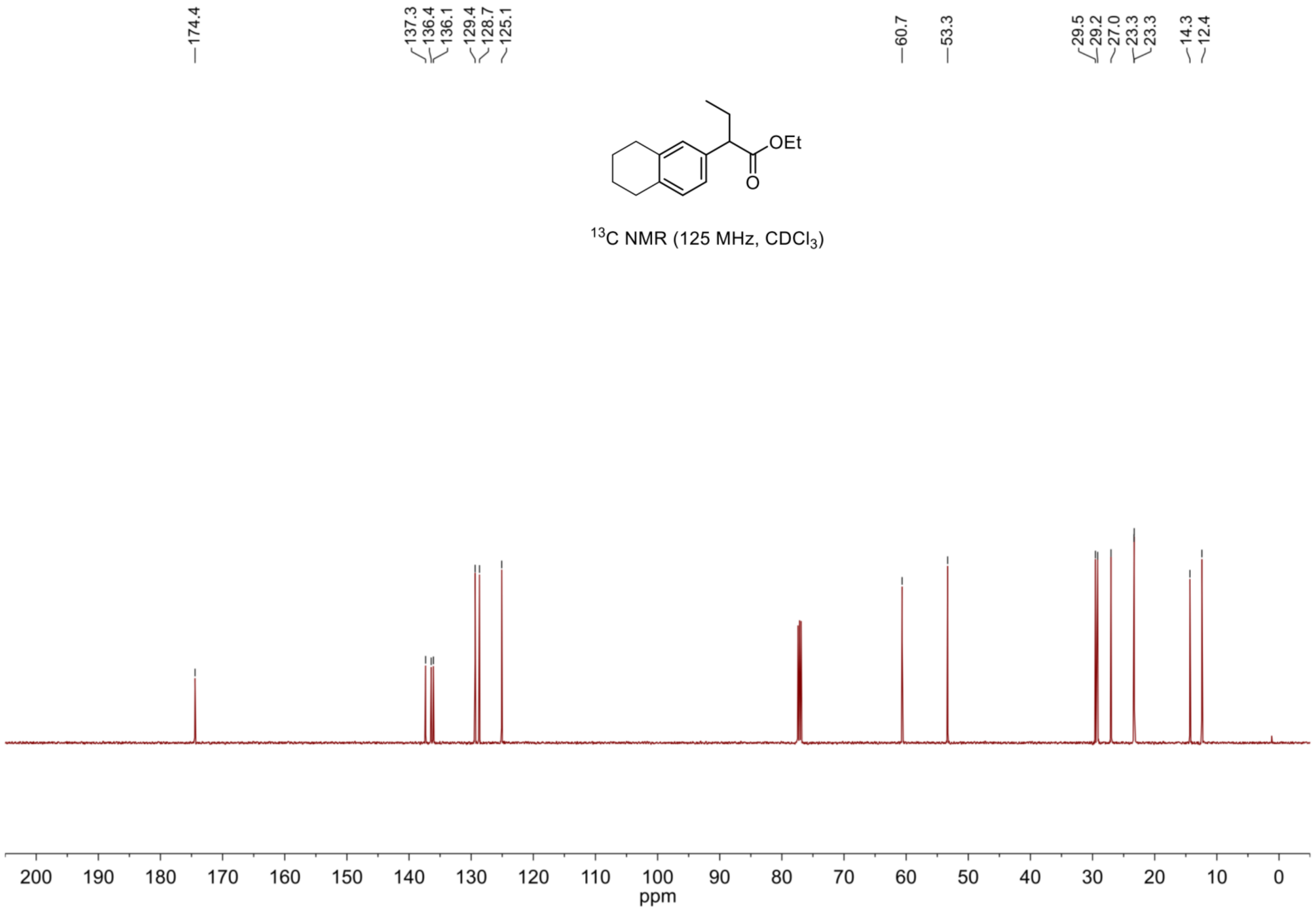

Go back to table of contents

Ethyl 2-(6-methoxynaphthalen-2-yl)butanoate (25)

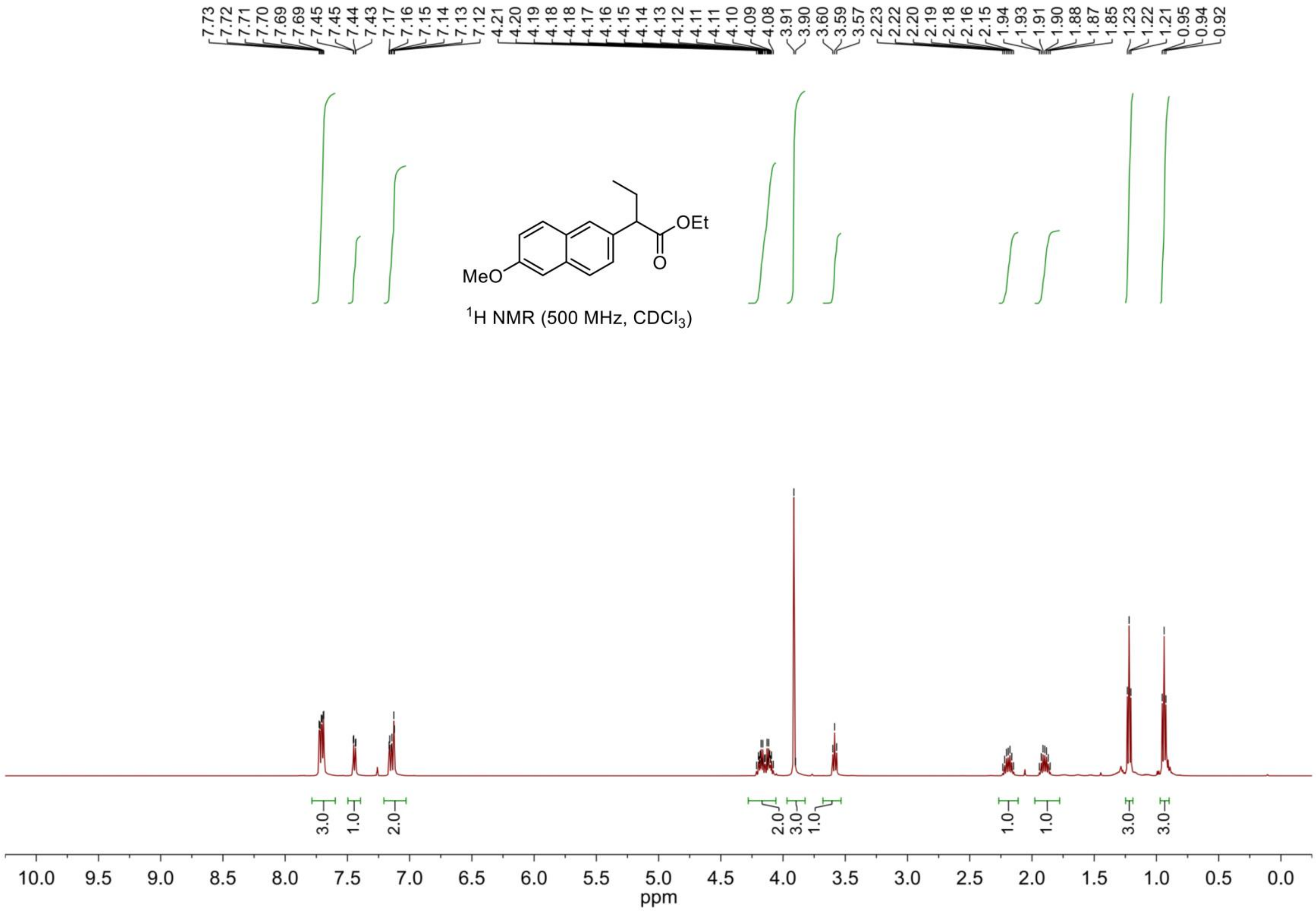

Ethyl 2-(6-methoxynaphthalen-2-yl)butanoate (25)

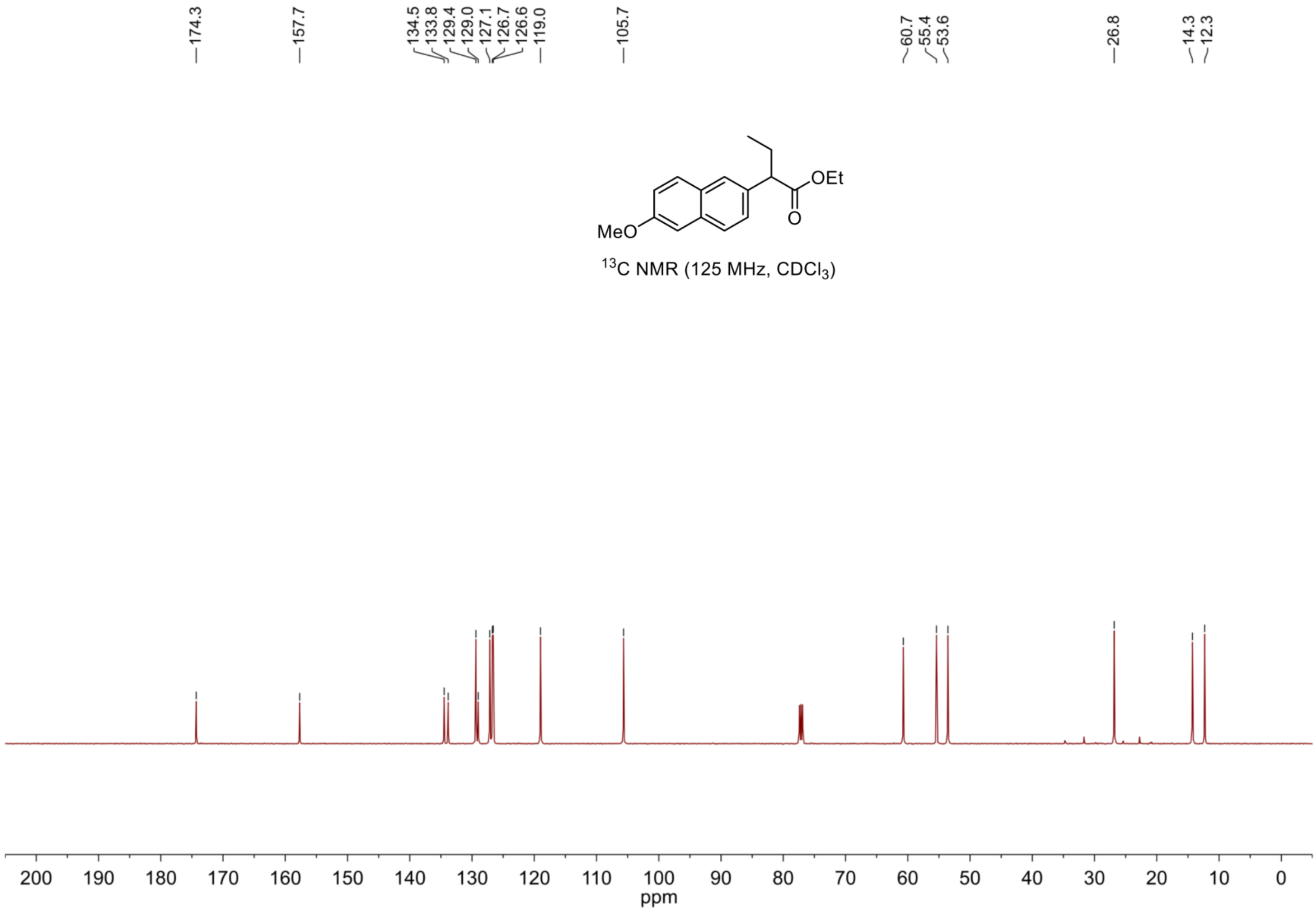

Go back to table of contents

Ethyl 2-(1,2-dihydroacenaphthylen-5-yl)butanoate (26)

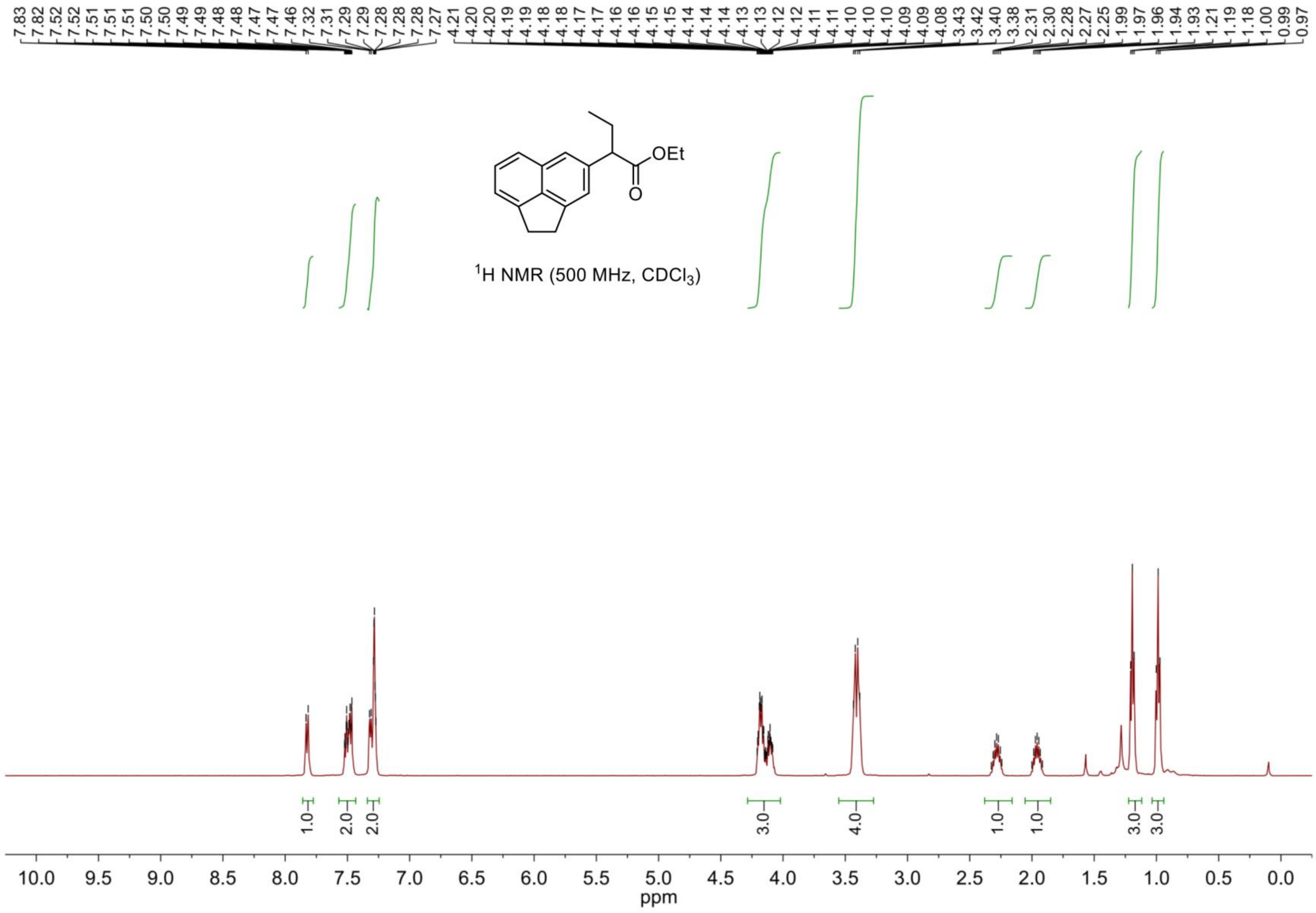

Go back to table of contents

Ethyl 2-(1,2-dihydroacenaphthylen-5-yl)butanoate (26)

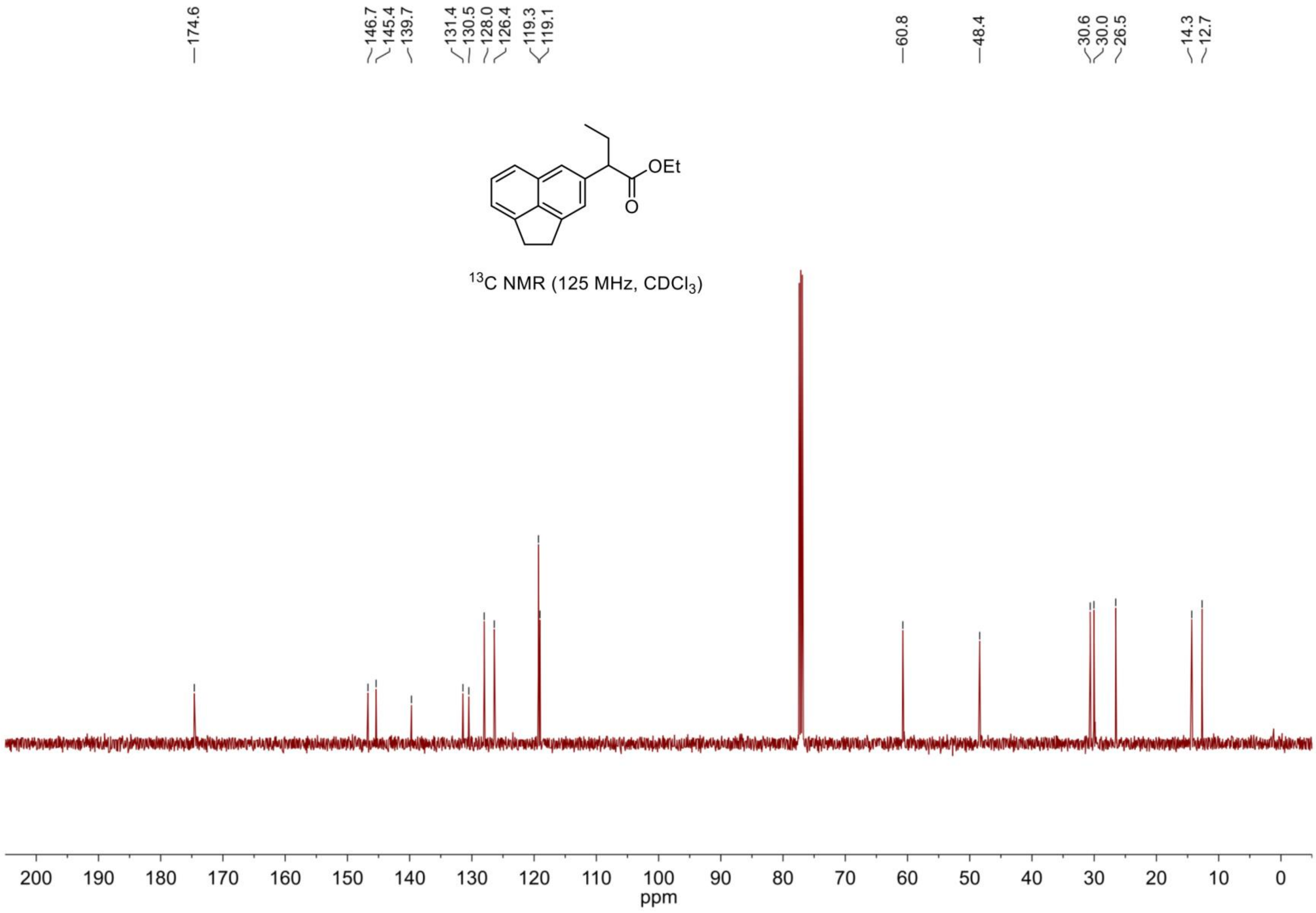

Ethyl 2-(pyren-1-yl)butanoate (27)

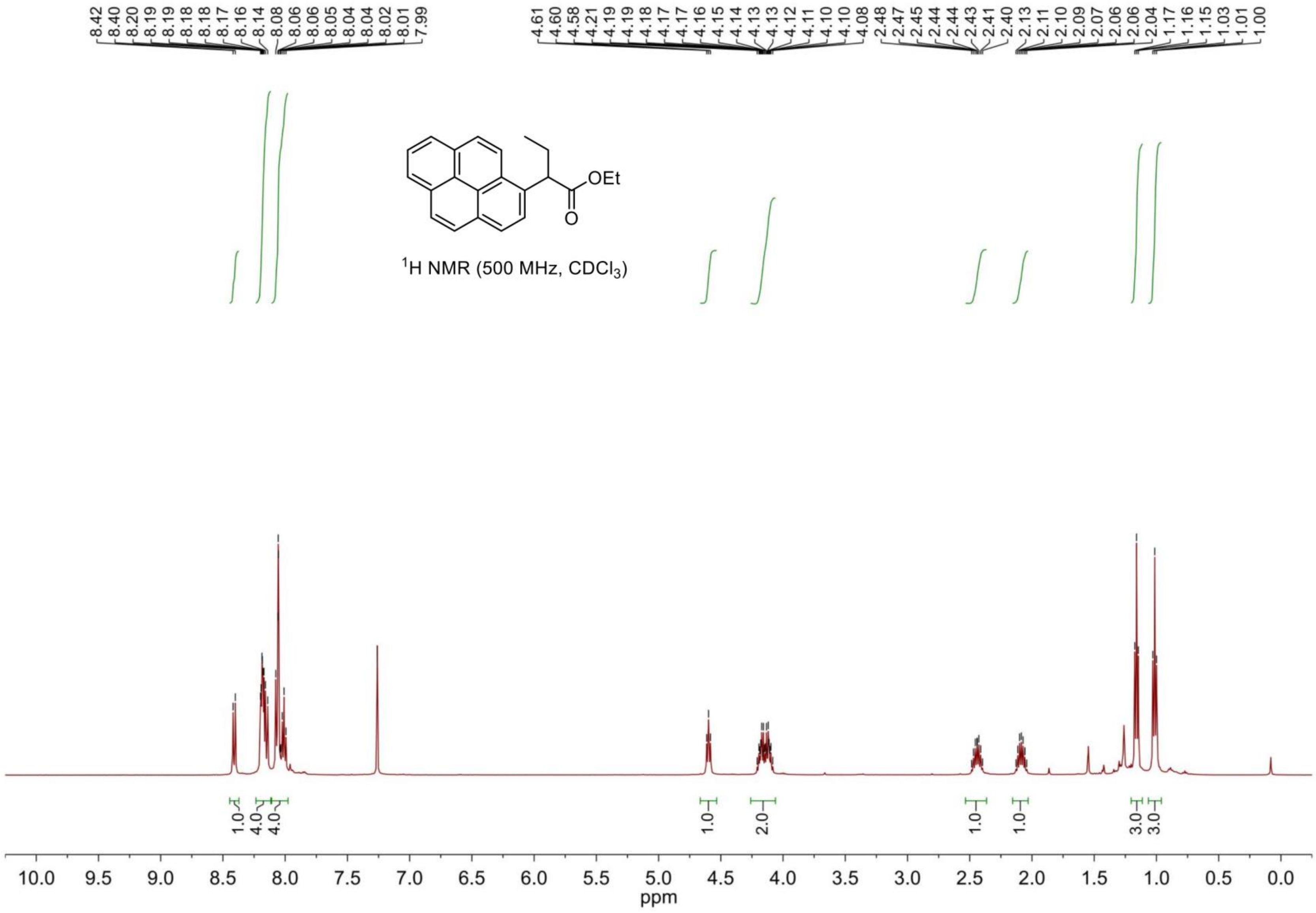

Go back to table of contents

Ethyl 2-(pyren-1-yl)butanoate (27)

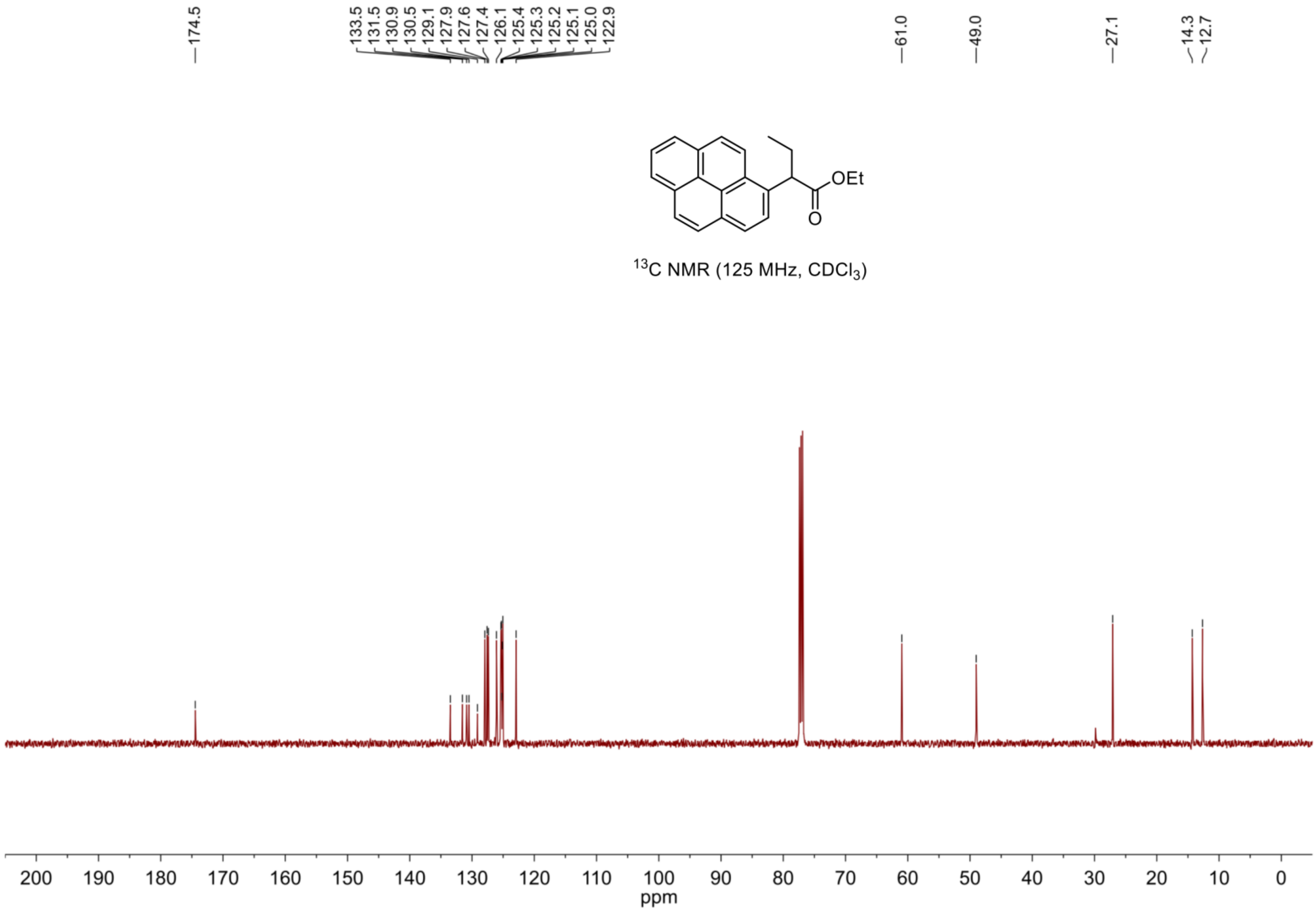

Go back to table of contents

Ethyl 2-(thiophen-2-yl)butanoate (28)

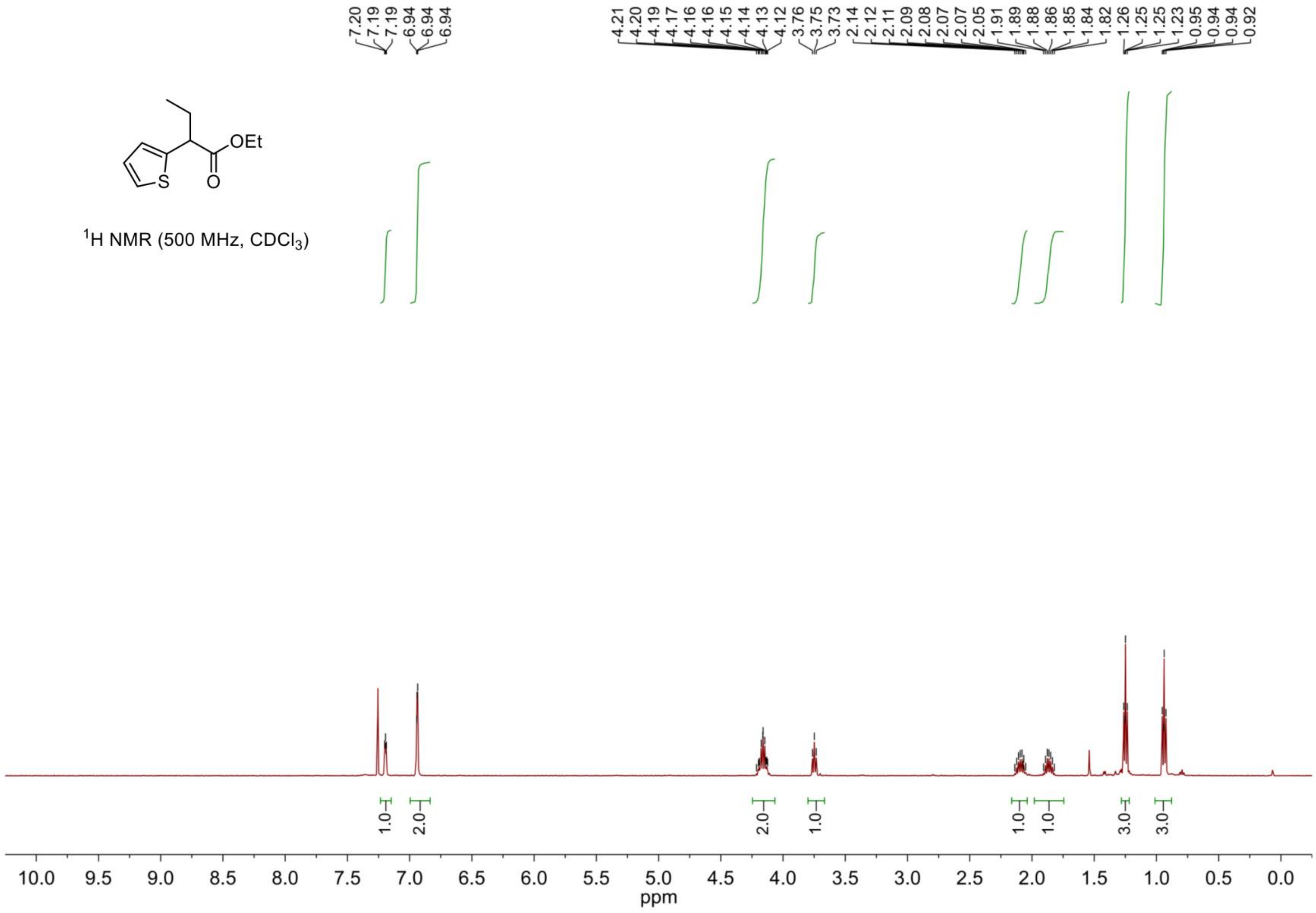

Go back to table of contents

Ethyl 2-(thiophen-2-yl)butanoate (28)

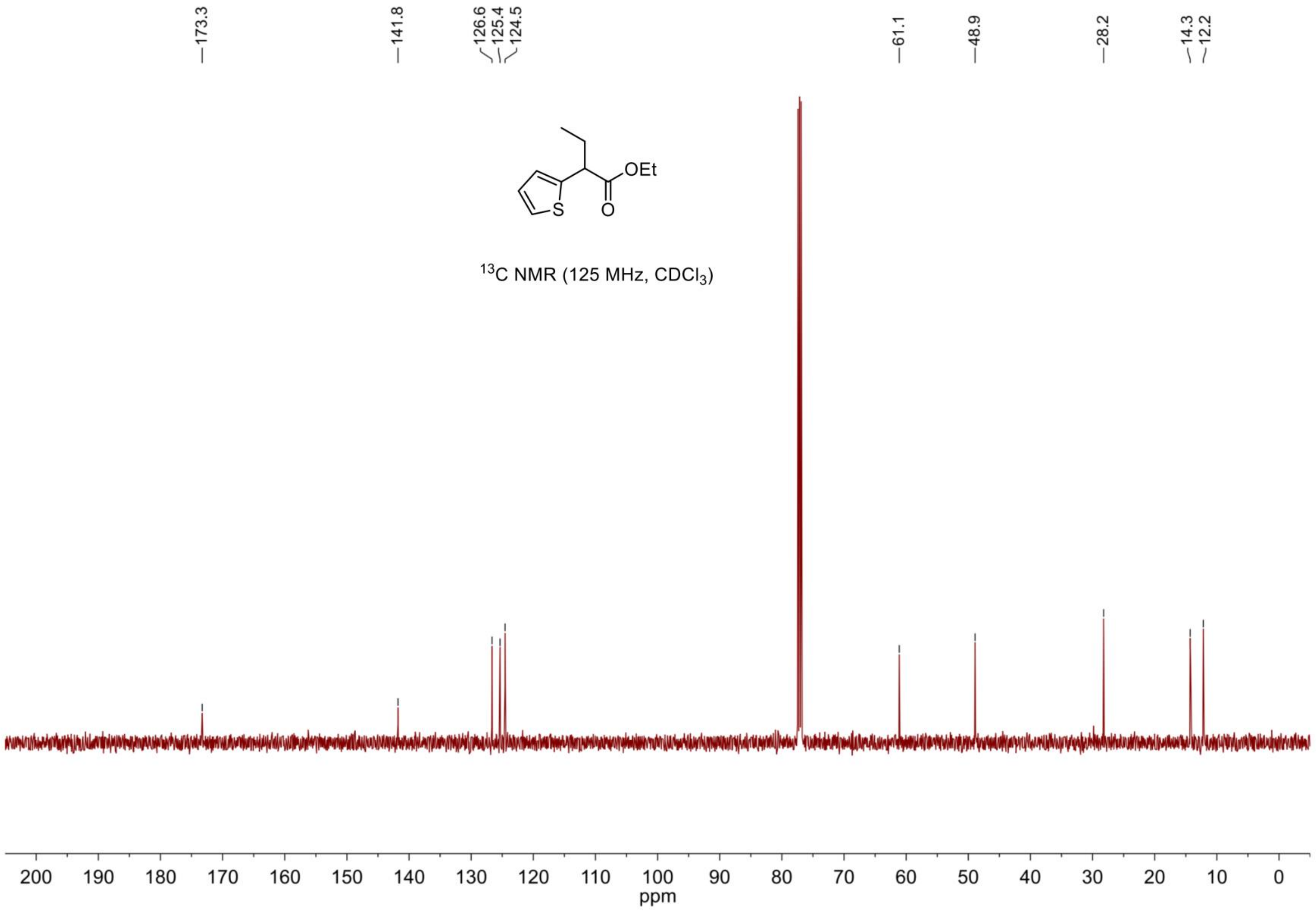

Go back to table of contents

Ethyl 2-(5-methylthiophen-2-yl)butanoate (29)

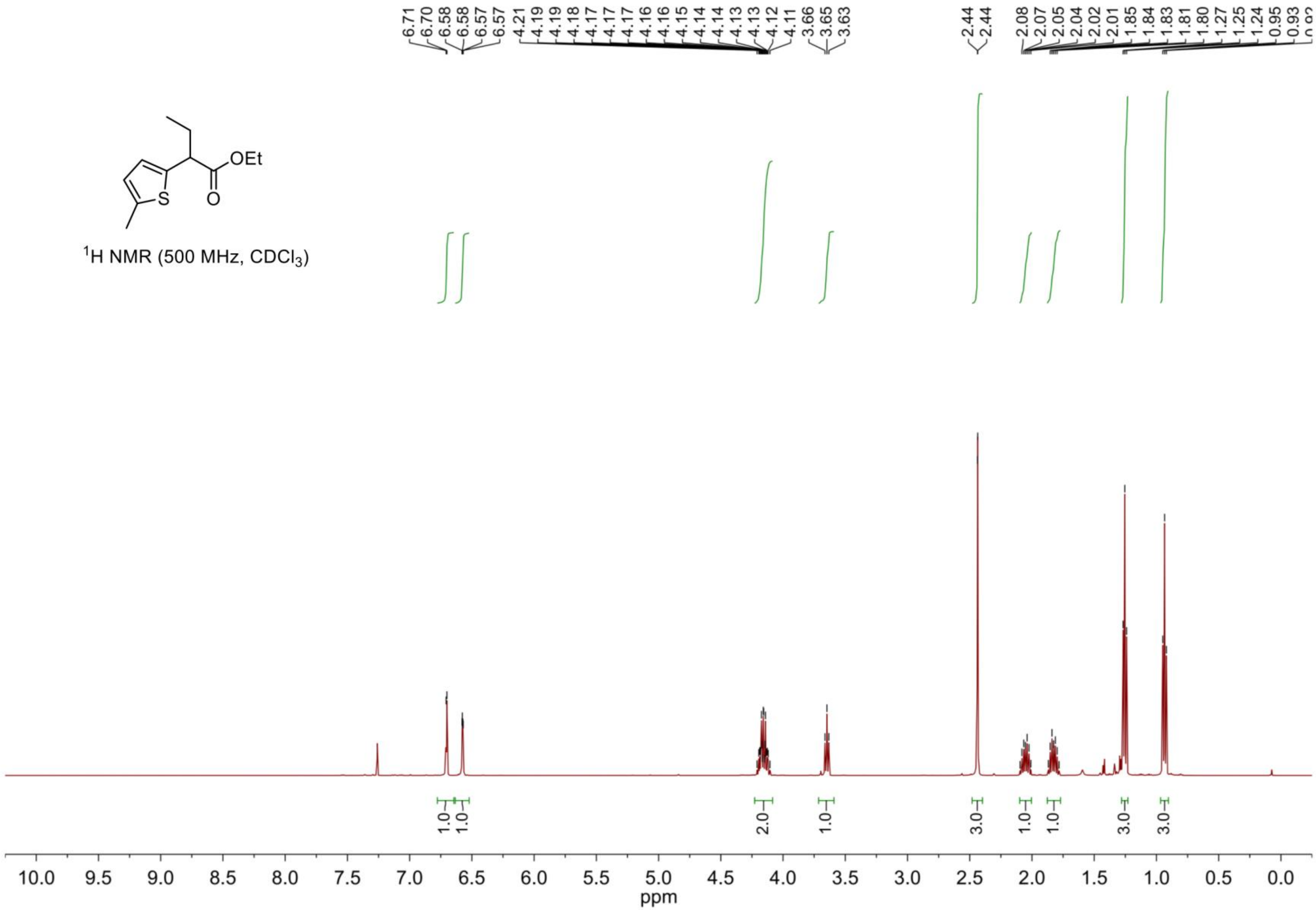

^1H NMR (500 MHz, $CDCl_3$)

Go back to table of contents

Ethyl 2-(5-methylthiophen-2-yl)butanoate (29)

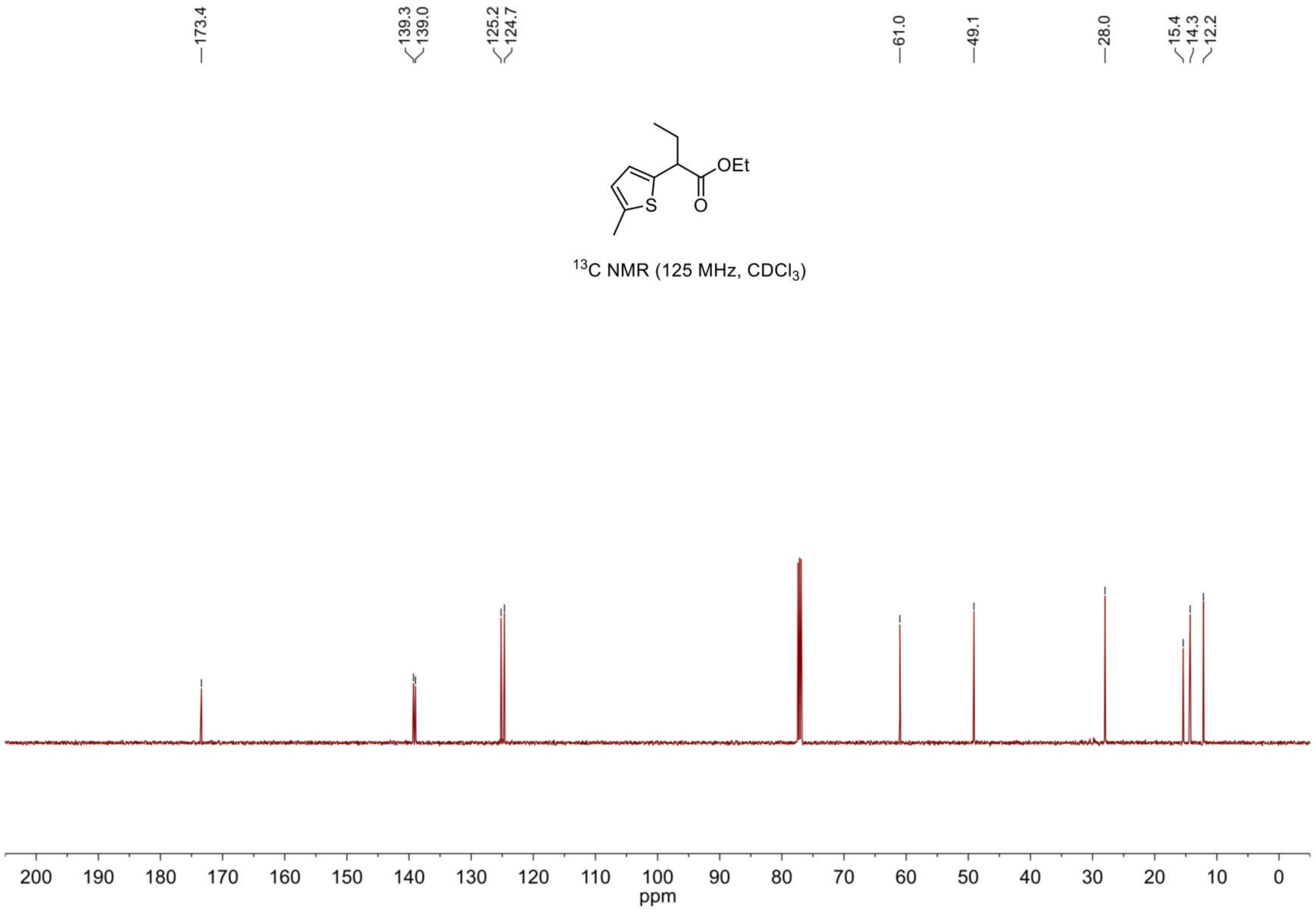

Go back to table of contents

Ethyl 2-(3-((5-(4-fluorophenyl)thiophen-2-yl)methyl)-4-methylphenyl)butanoate (30)

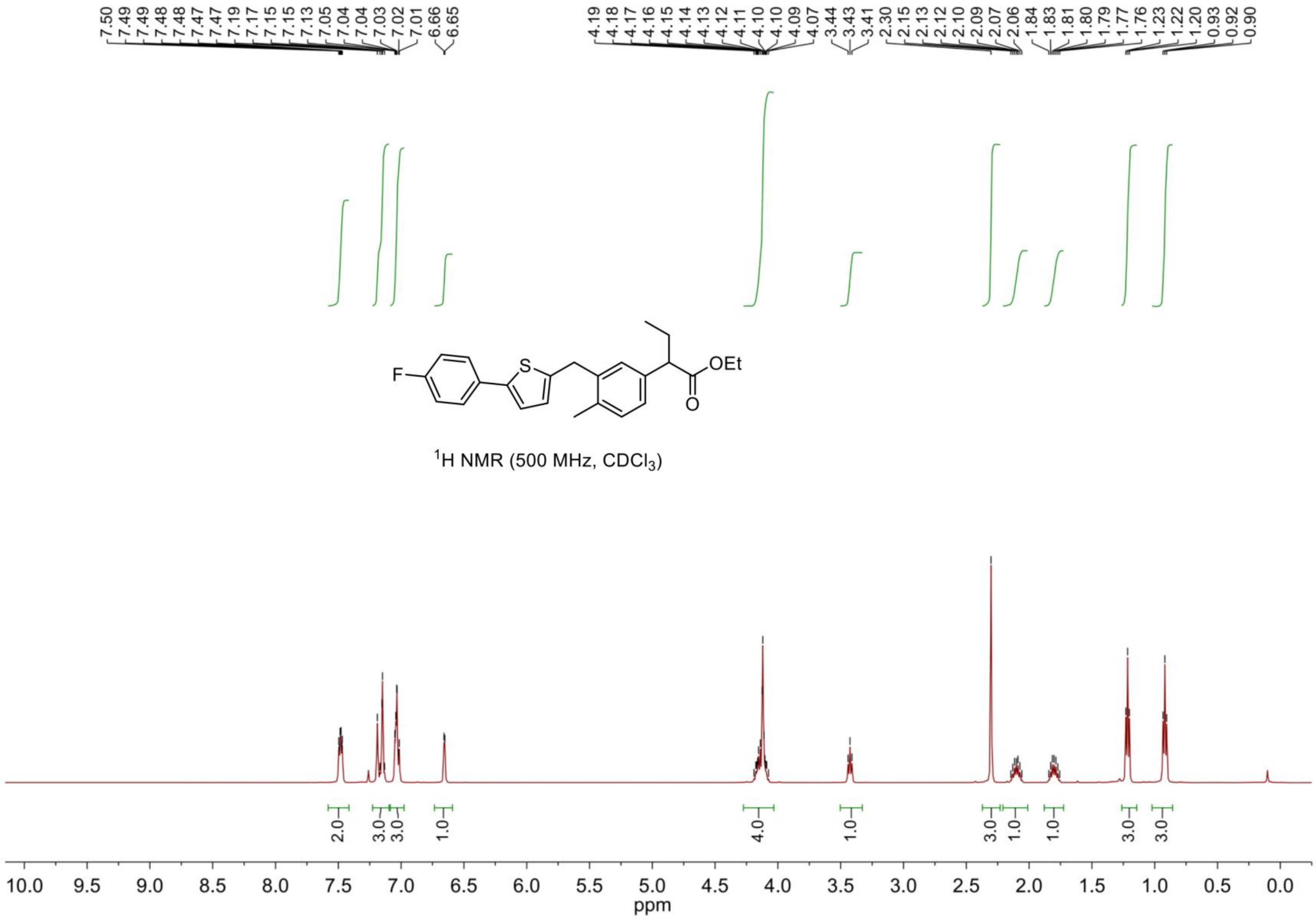

Go back to table of contents

Ethyl 2-(3-((5-(4-fluorophenyl)thiophen-2-yl)methyl)-4-methylphenyl)butanoate (30)

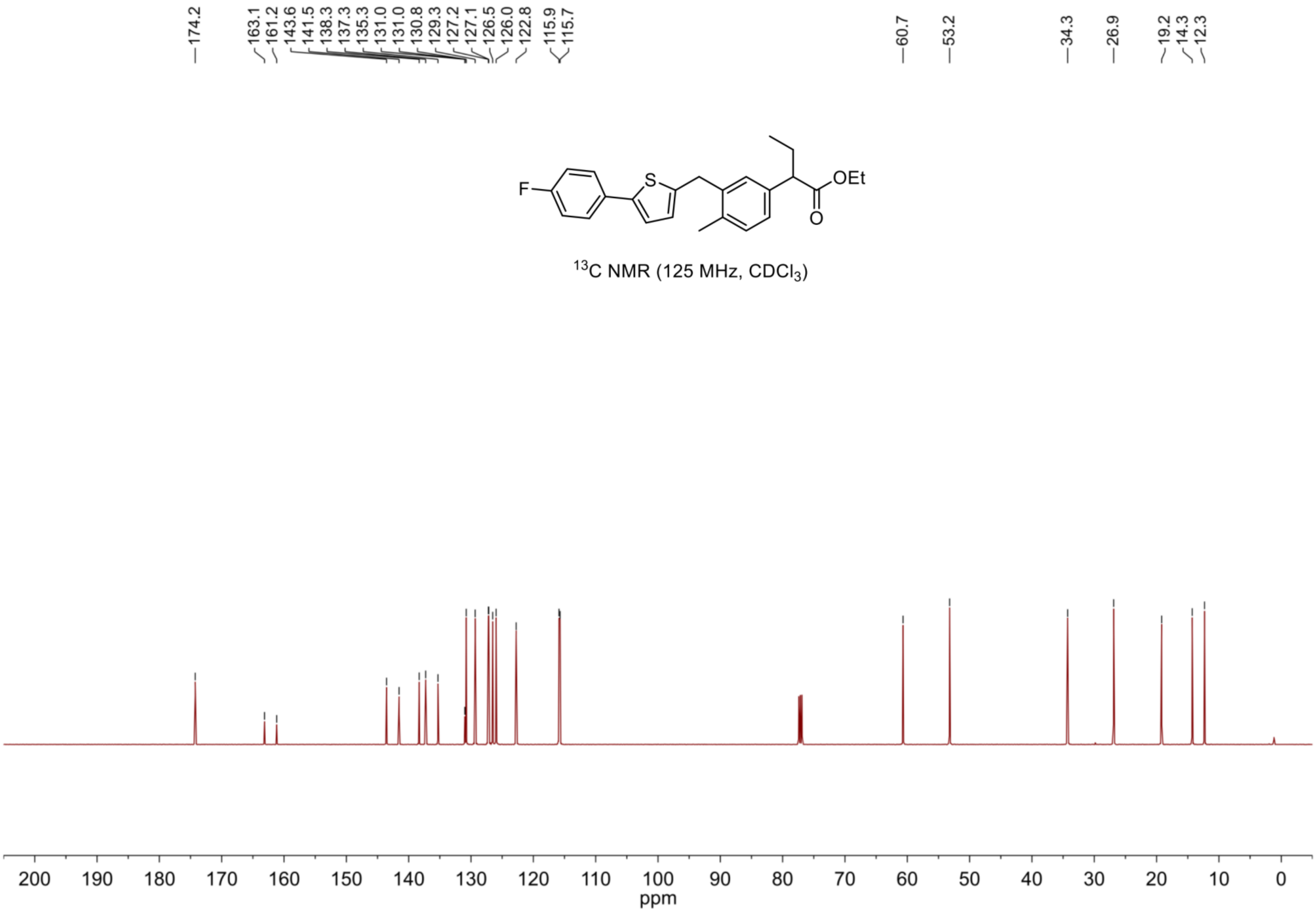

Go back to table of contents

Ethyl 2-(pyridin-3-yl)butanoate (31)

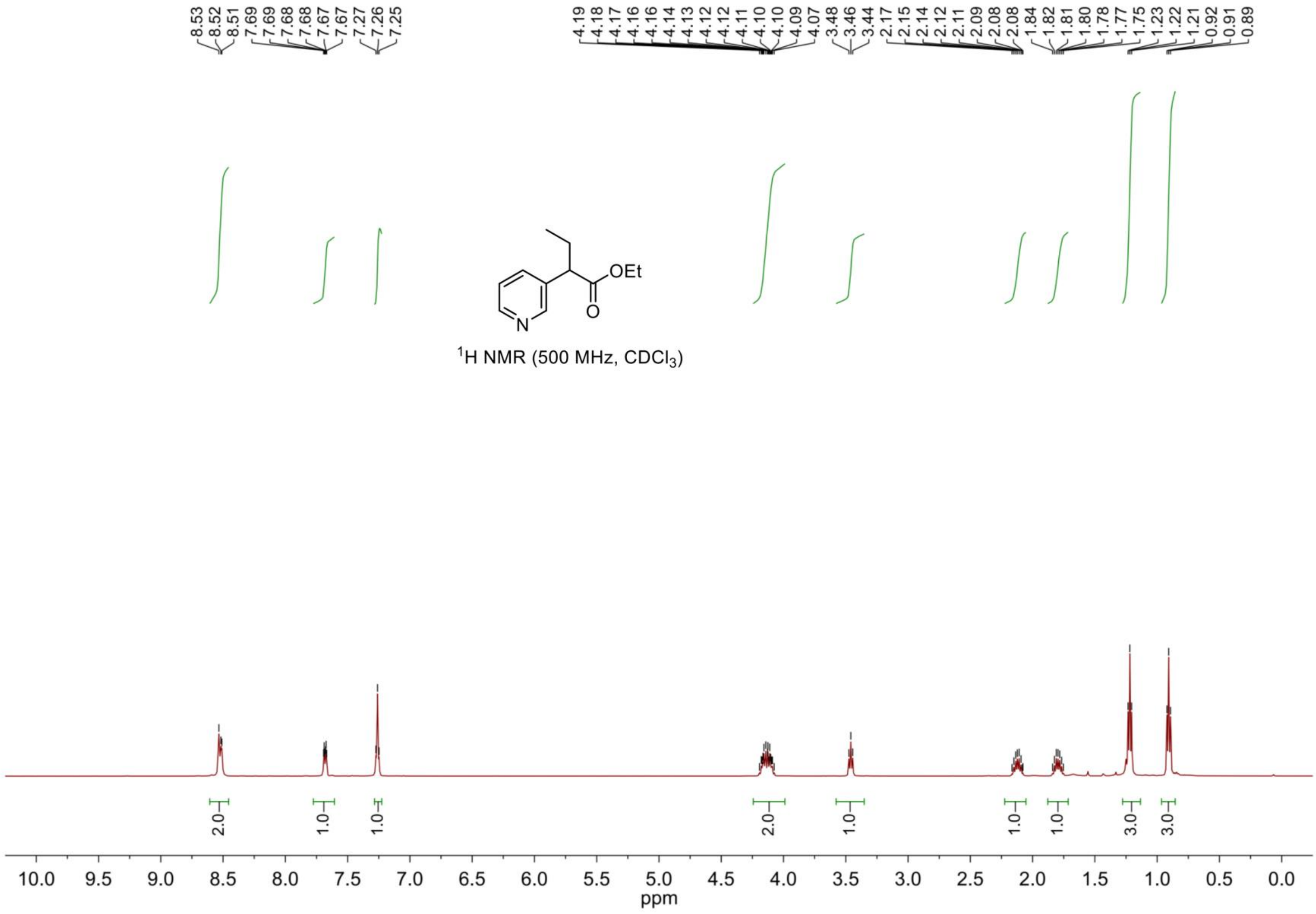

Go back to table of contents

Ethyl 2-(pyridin-3-yl)butanoate (31)

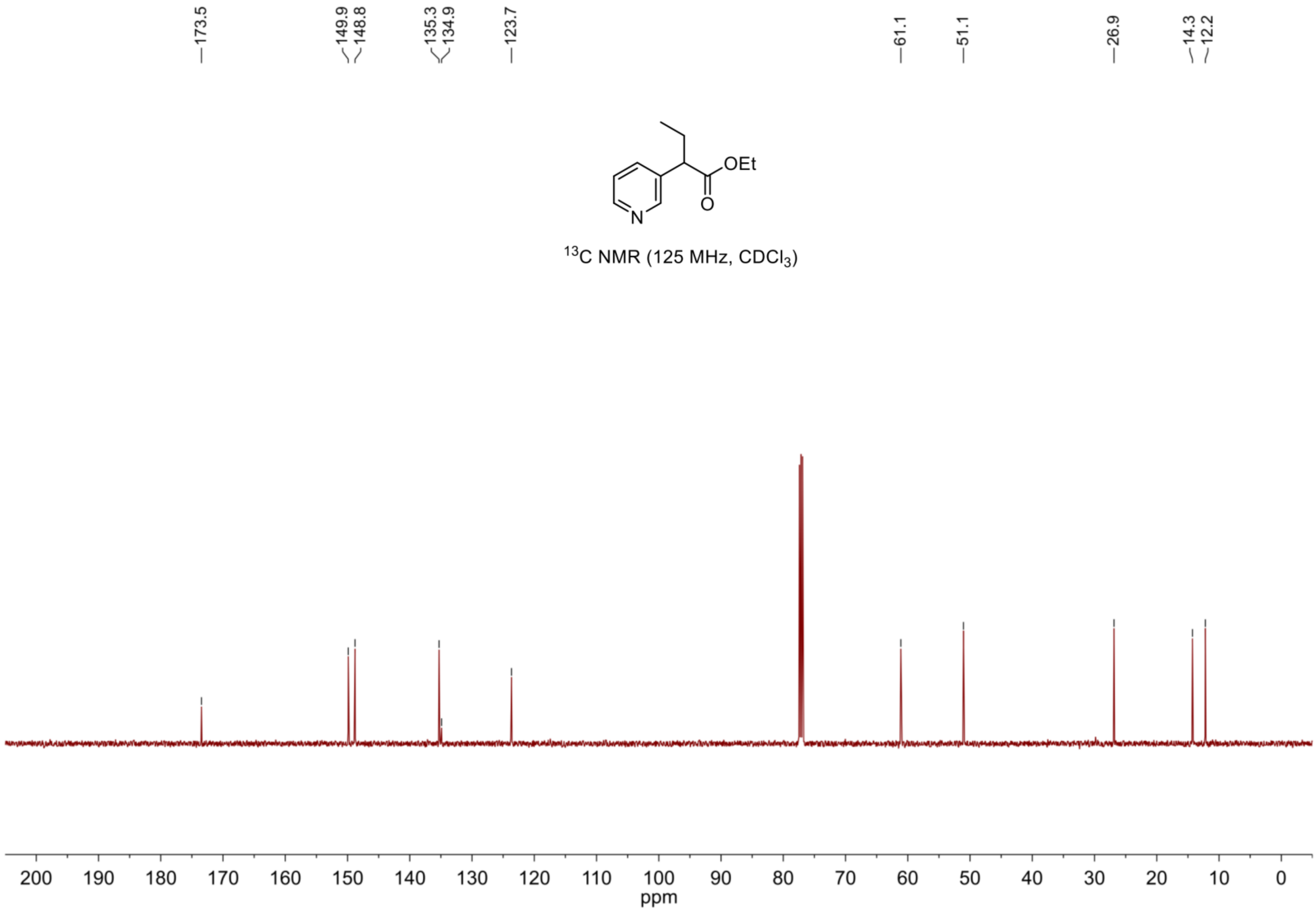

Go back to table of contents

Ethyl 2-(2,3-dihydrobenzofuran-5-yl)butanoate (32)

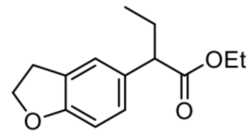

^1H NMR (500 MHz, $CDCl_3$)

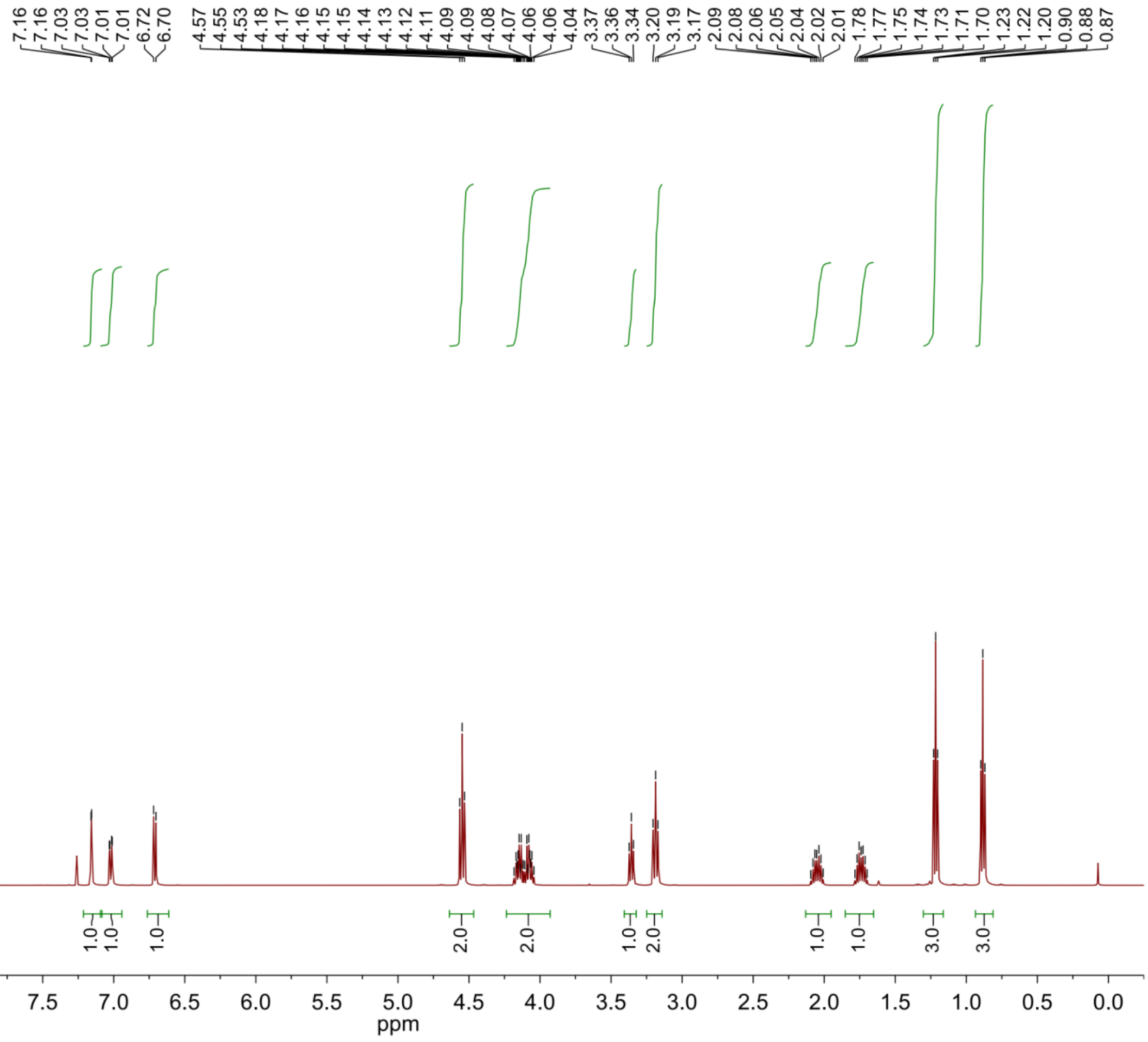

Go back to table of contents

Ethyl 2-(2,3-dihydrobenzofuran-5-yl)butanoate (32)

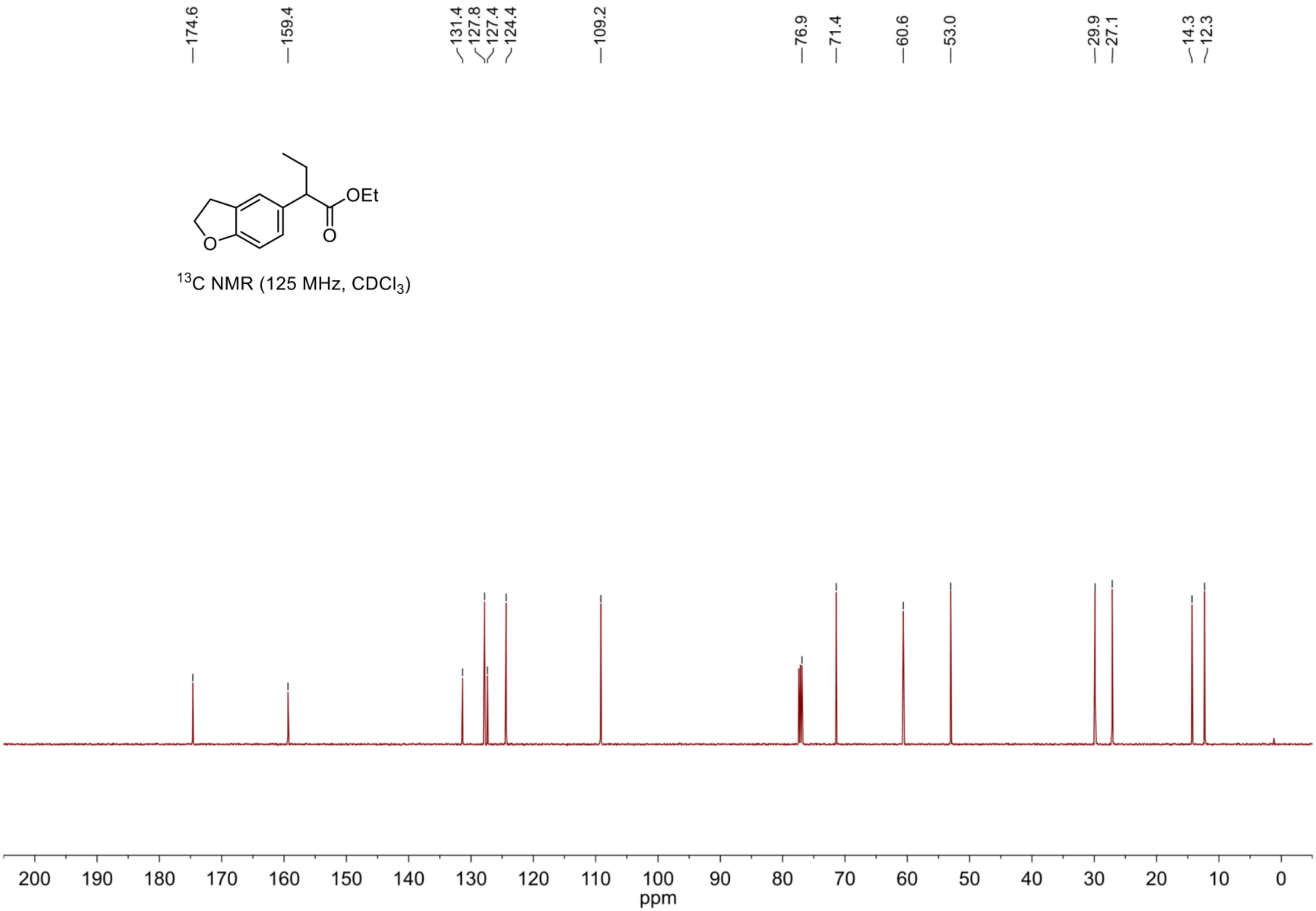

Go back to table of contents

tert-Butyl 3-(1-ethoxy-1-oxobutan-2-yl)-1H-indole-1-carboxylate (33)

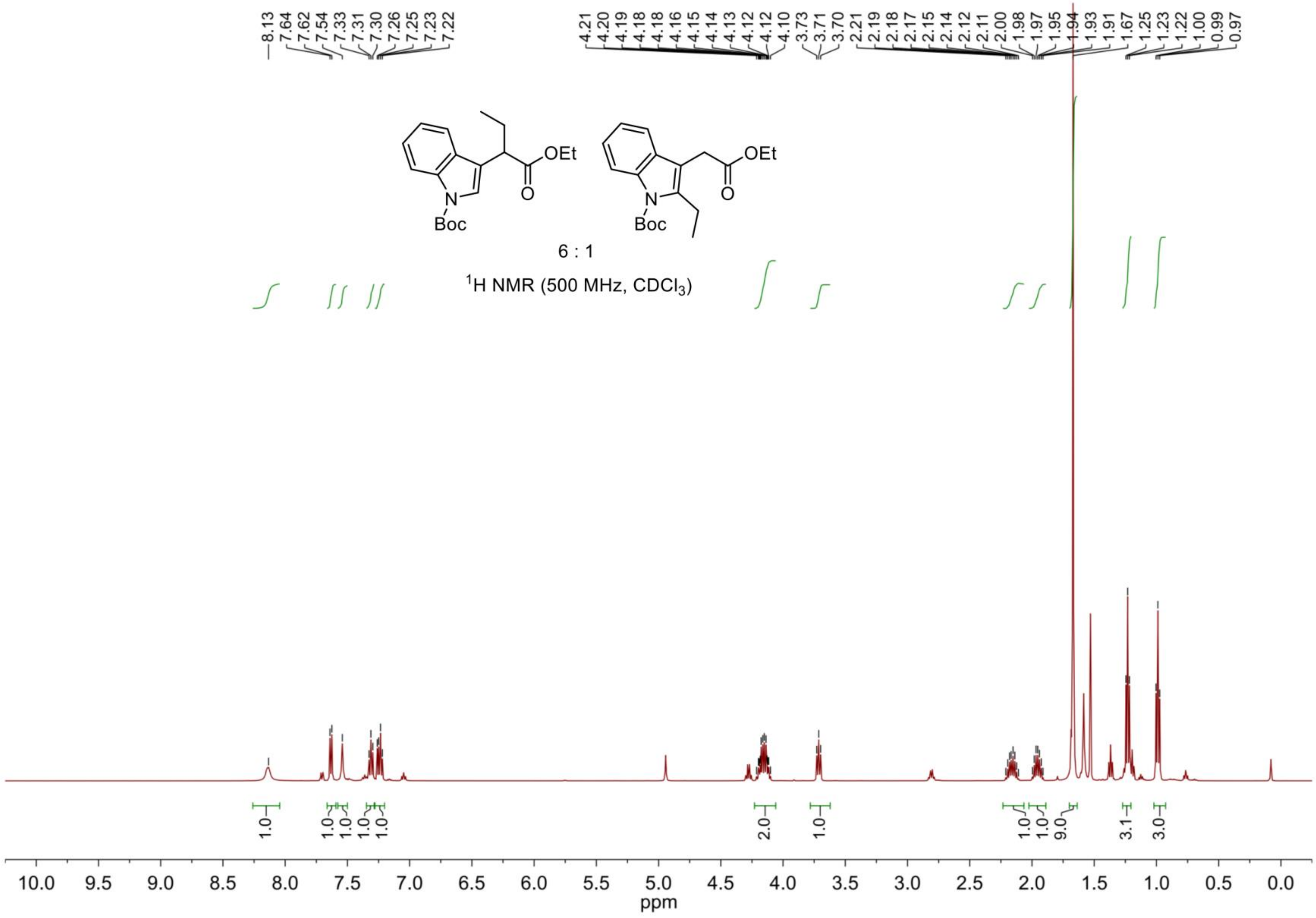

Go back to table of contents

tert-Butyl 3-(1-ethoxy-1-oxobutan-2-yl)-1*H*-indole-1-carboxylate (33)

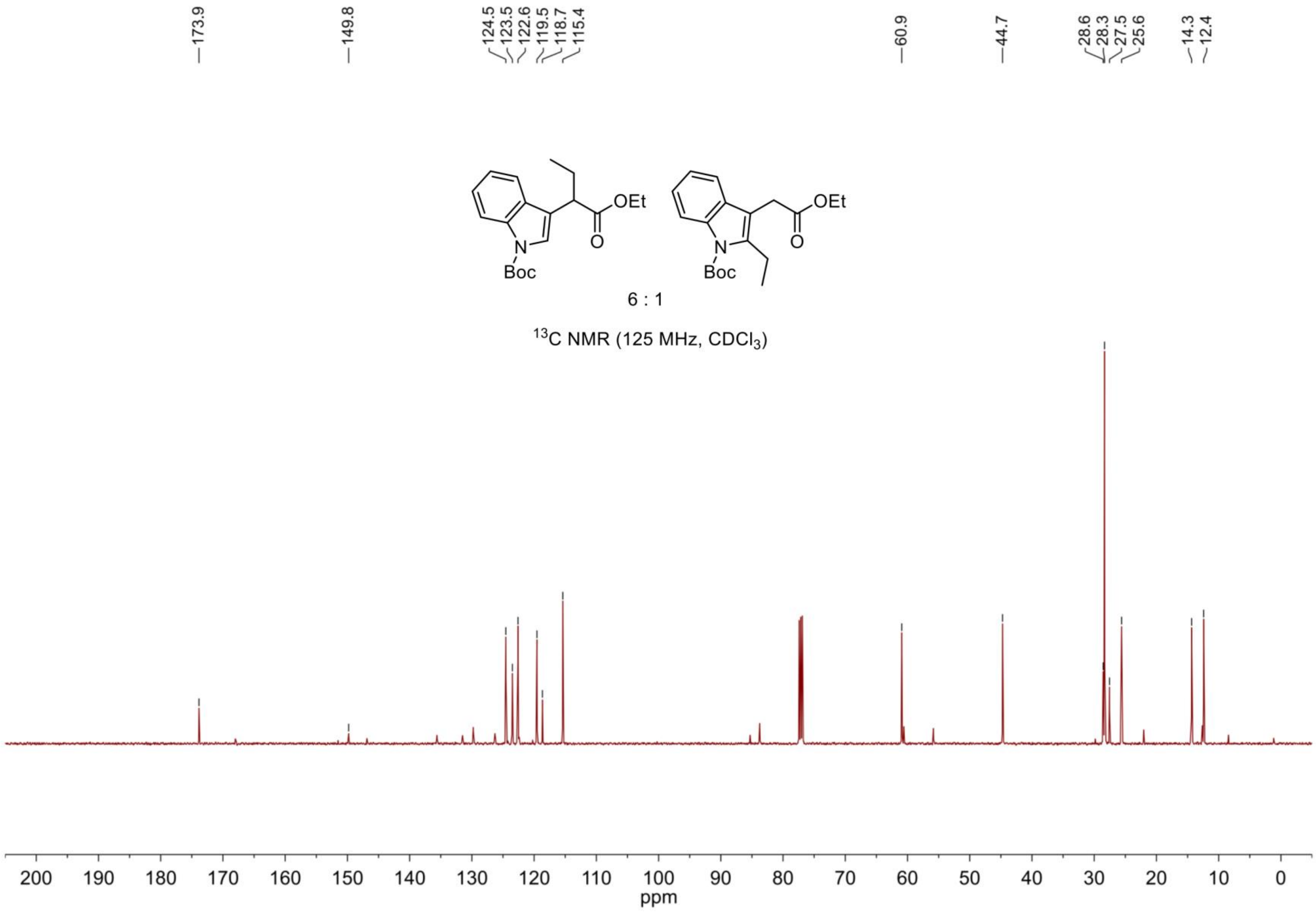

Go back to table of contents

Ethyl 2-(1-(2,5-dimethylbenzyl)-1*H*-indol-5-yl)butanoate (34)

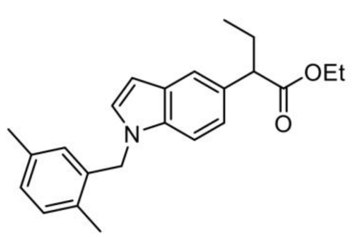

^{1}H NMR (500 MHz, $CDCl_3$)

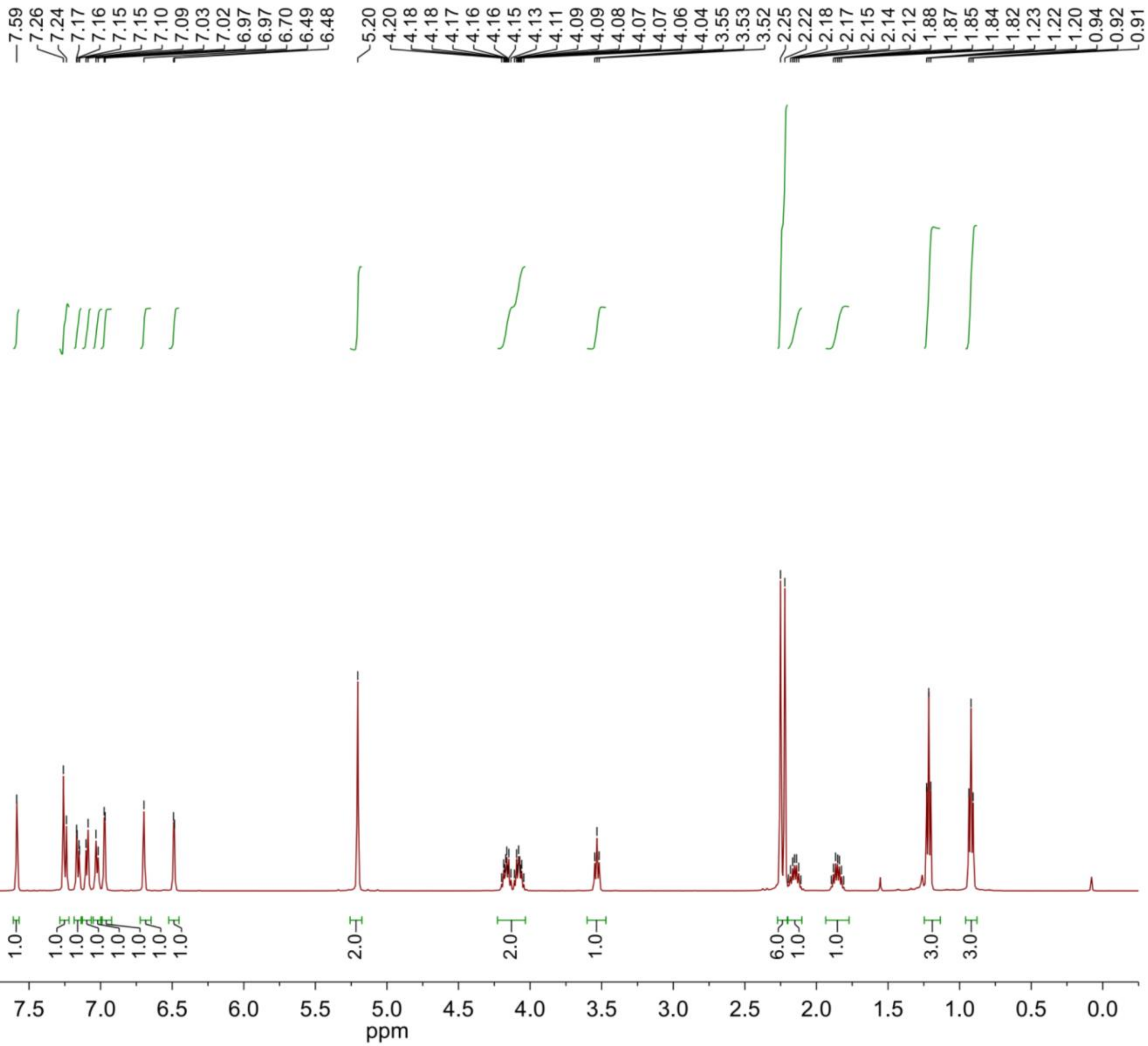

Go back to table of contents

Ethyl 2-(1-(2,5-dimethylbenzyl)-1*H*-indol-5-yl)butanoate (34)

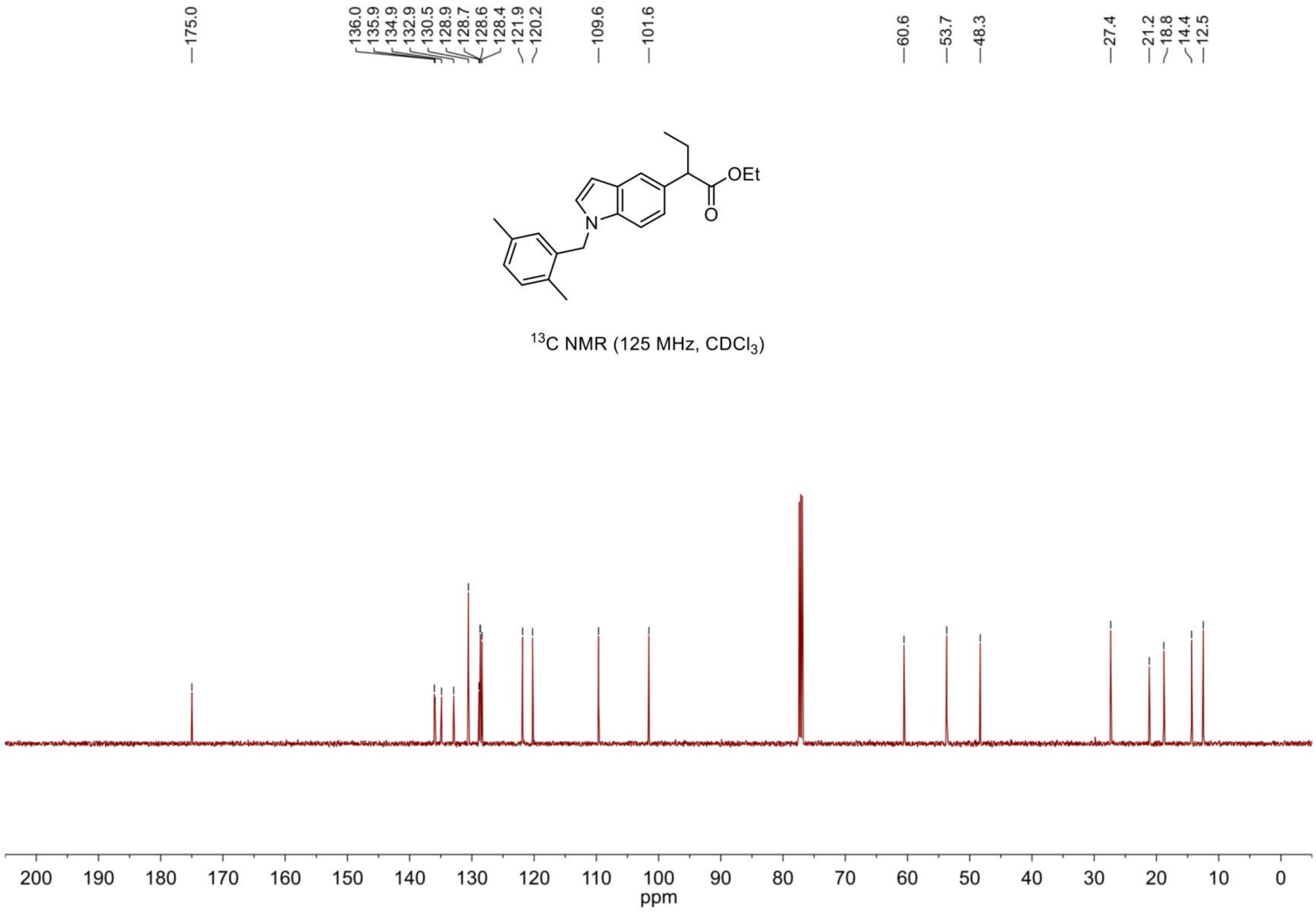

Go back to table of contents

Ethyl 2-(9-methyl-9*H*-carbazol-3-yl)butanoate (35)

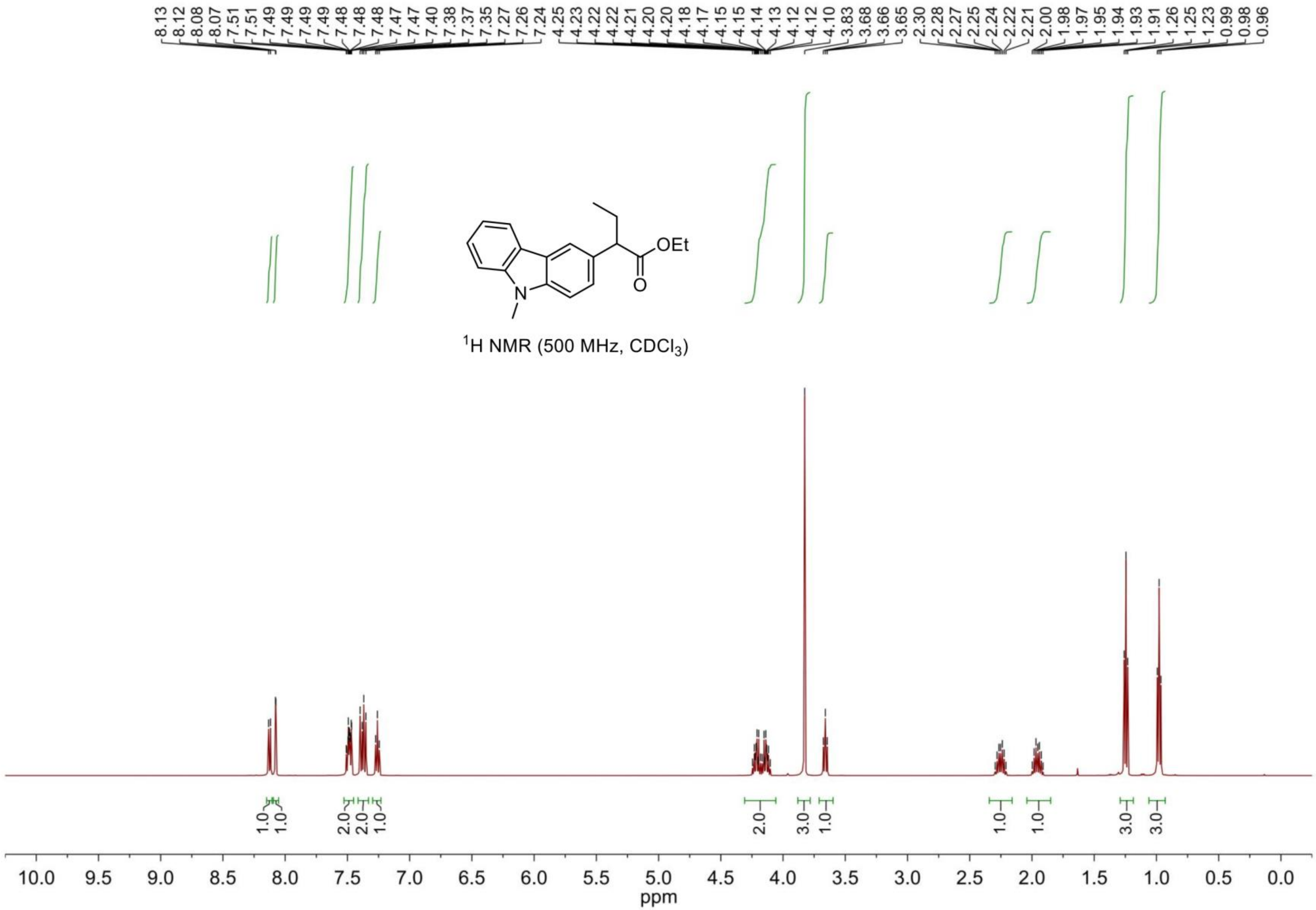

Go back to table of contents

Ethyl 2-(9-methyl-9*H*-carbazol-3-yl)butanoate (35)

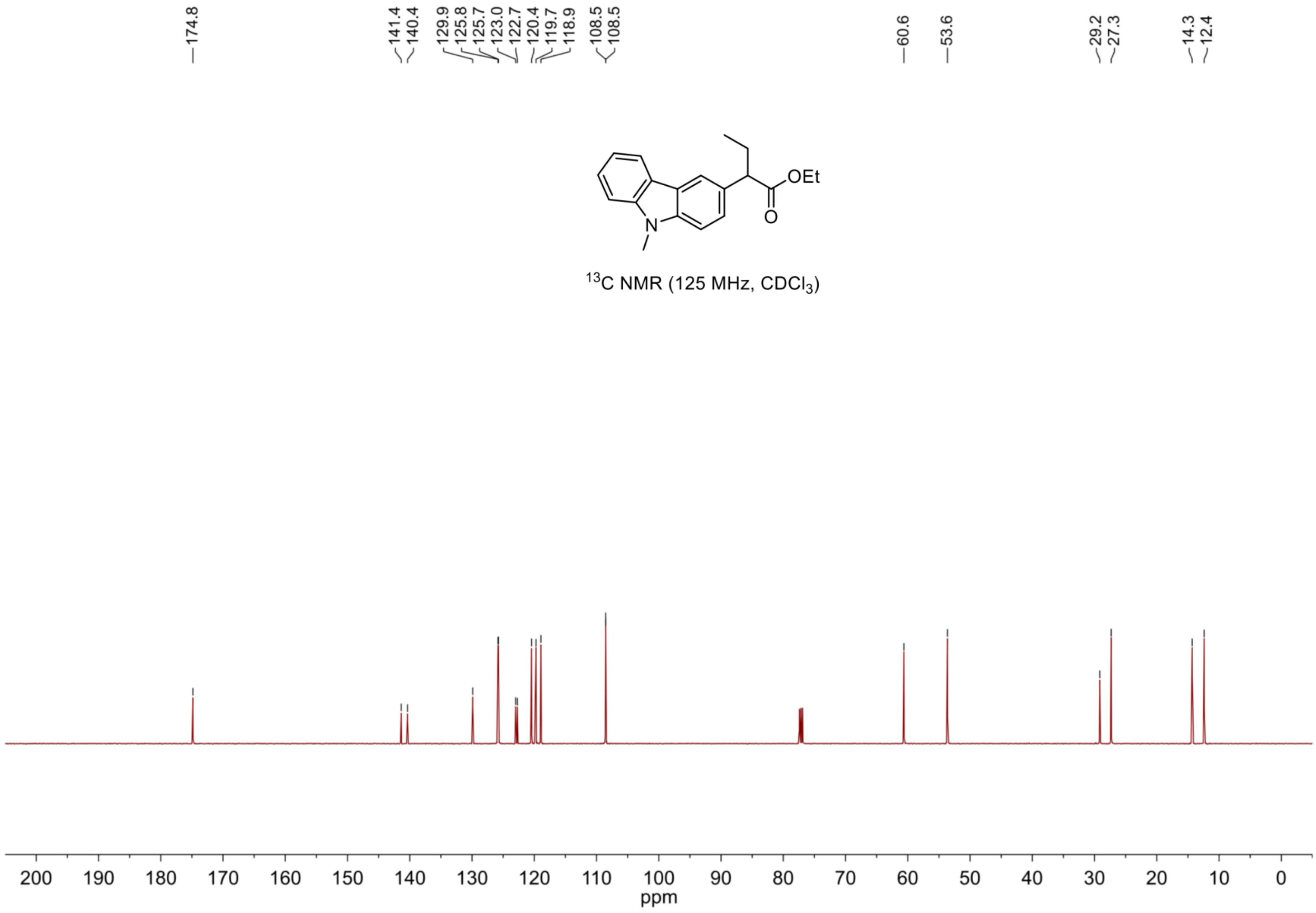

Go back to table of contents

Ethyl 2-(dibenzo[*b*,*d*]thiophen-2-yl)butanoate (36)

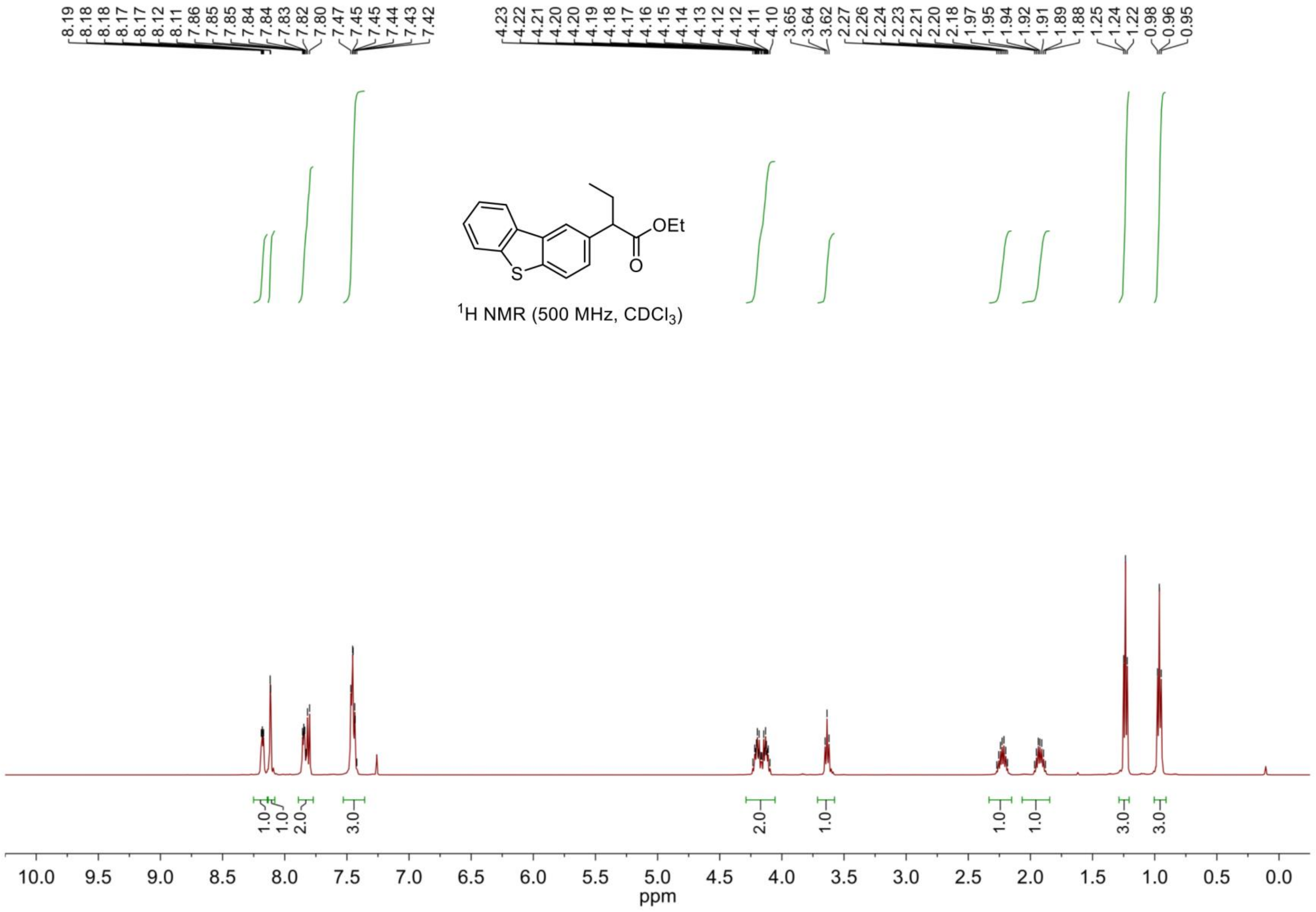

Go back to table of contents

Ethyl 2-(dibenzo[*b*,*d*]thiophen-2-yl)butanoate (36)

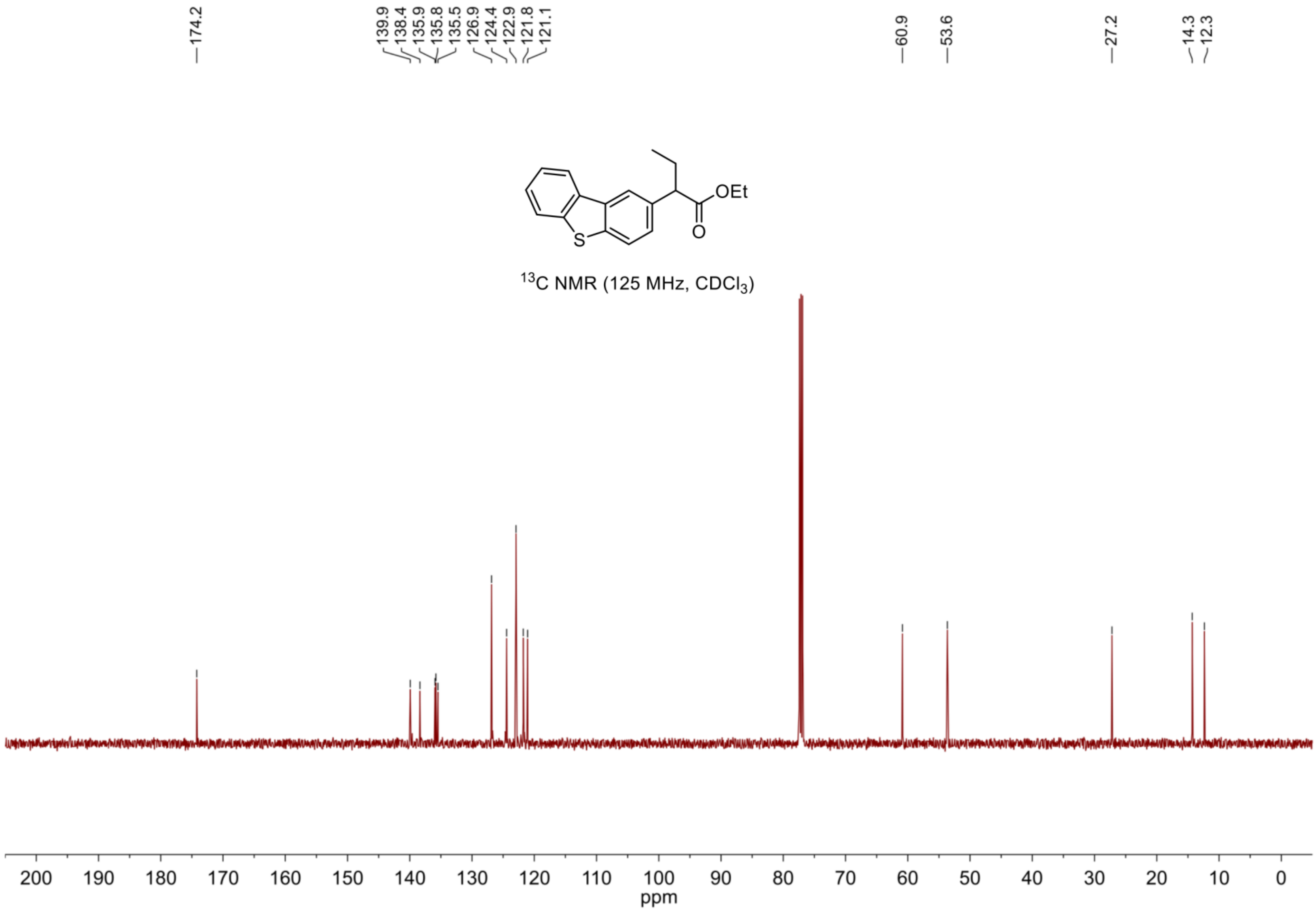

Go back to table of contents

Ethyl 2-(9,9-dimethyl-9*H*-xanthen-2-yl)butanoate (37)

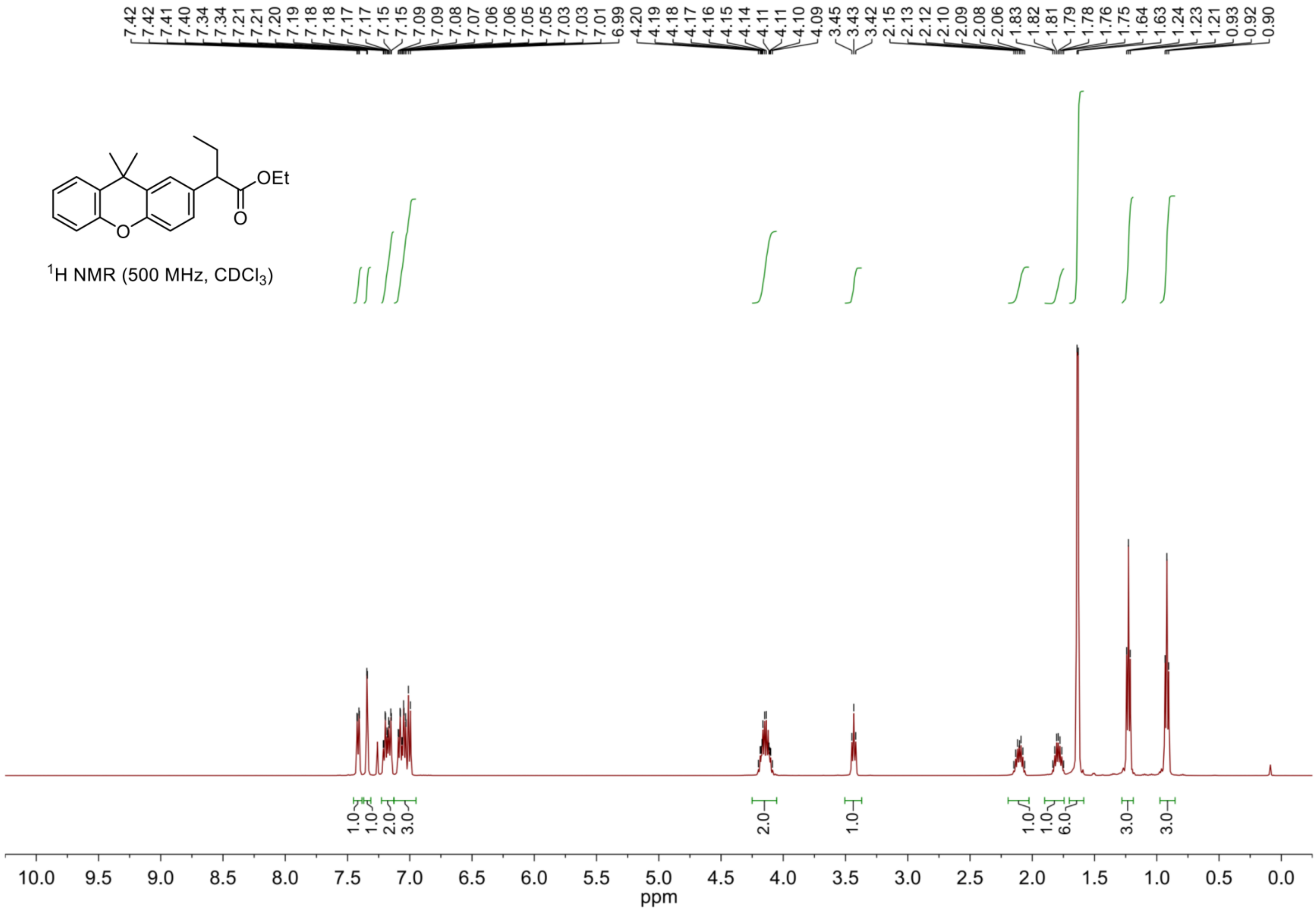

Go back to table of contents

Ethyl 2-(9,9-dimethyl-9*H*-xanthen-2-yl)butanoate (37)

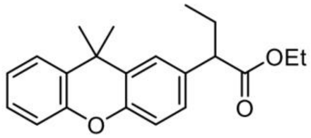

^{13}C NMR (125 MHz, $CDCl_3$)

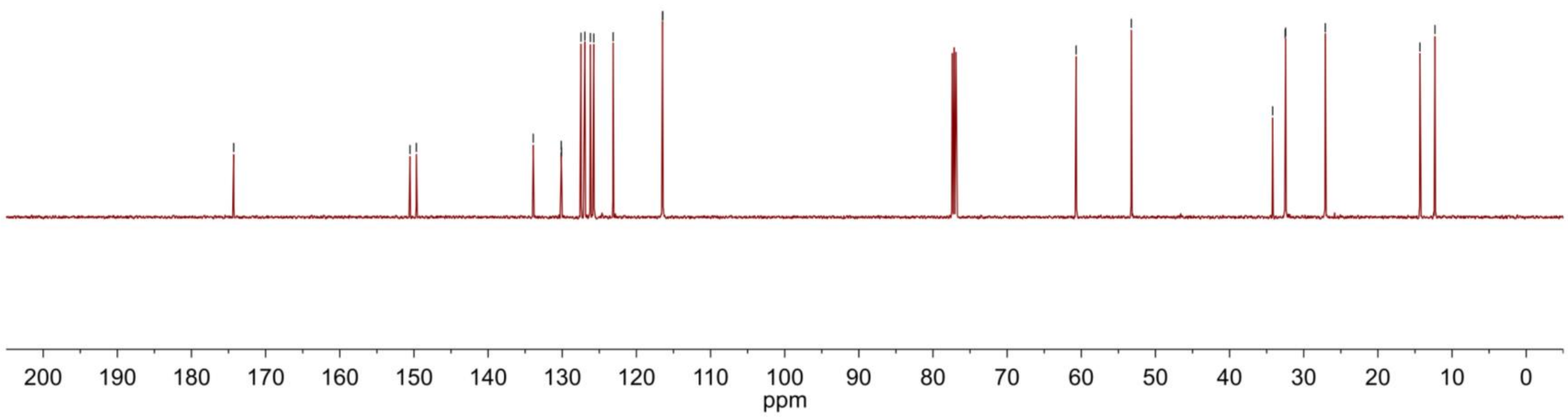

3-Ethyl-1-methylindolin-2-one (38)

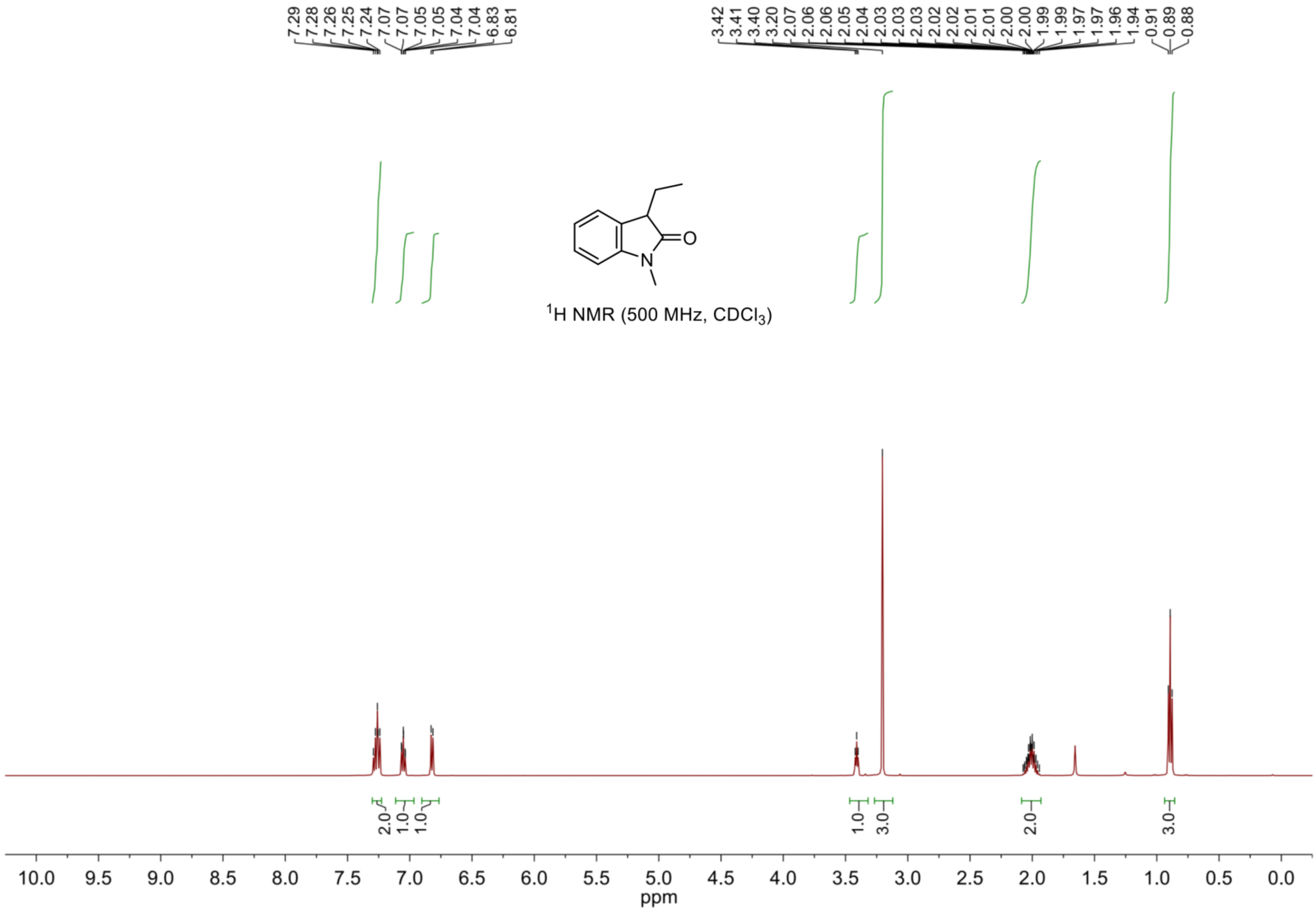

Go back to table of contents

3-Ethyl-1-methylindolin-2-one (38)

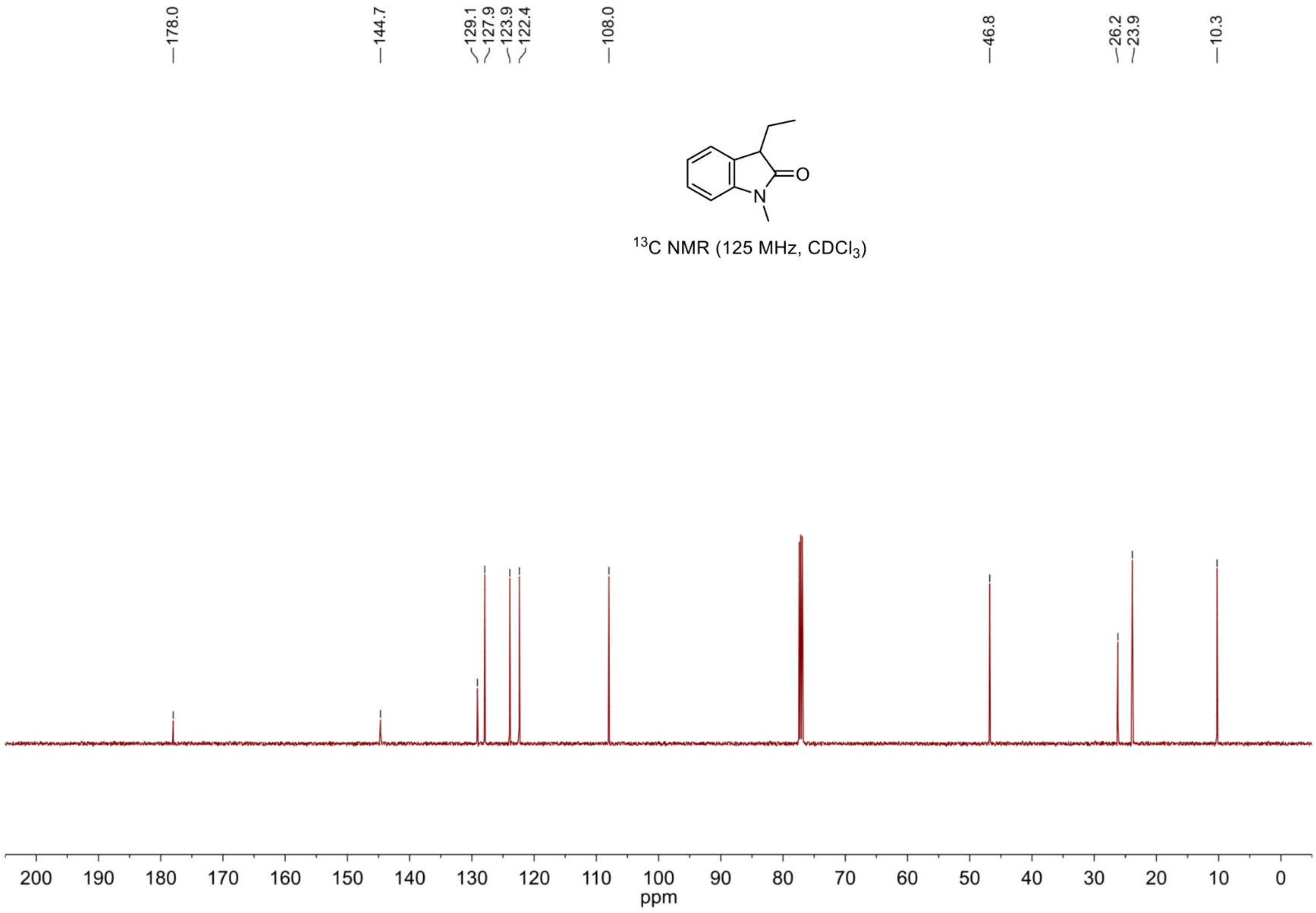

Go back to table of contents

Prop-2-yn-1-yl 2-phenylhexanoate (57)

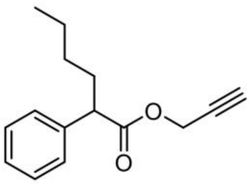

^{1}H NMR (500 MHz, $CDCl_3$)

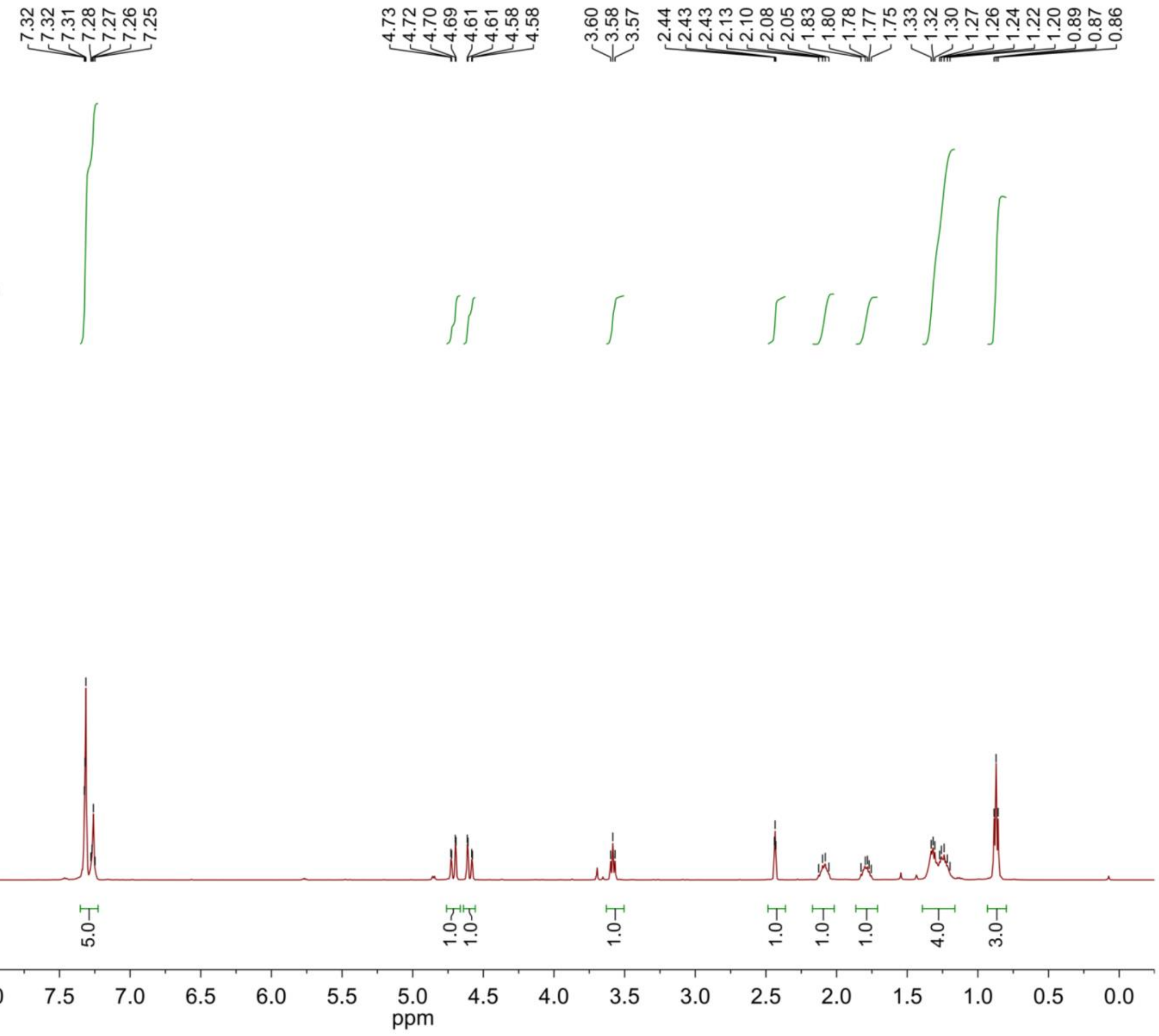

Go back to table of contents

Prop-2-yn-1-yl 2-phenylhexanoate (57)

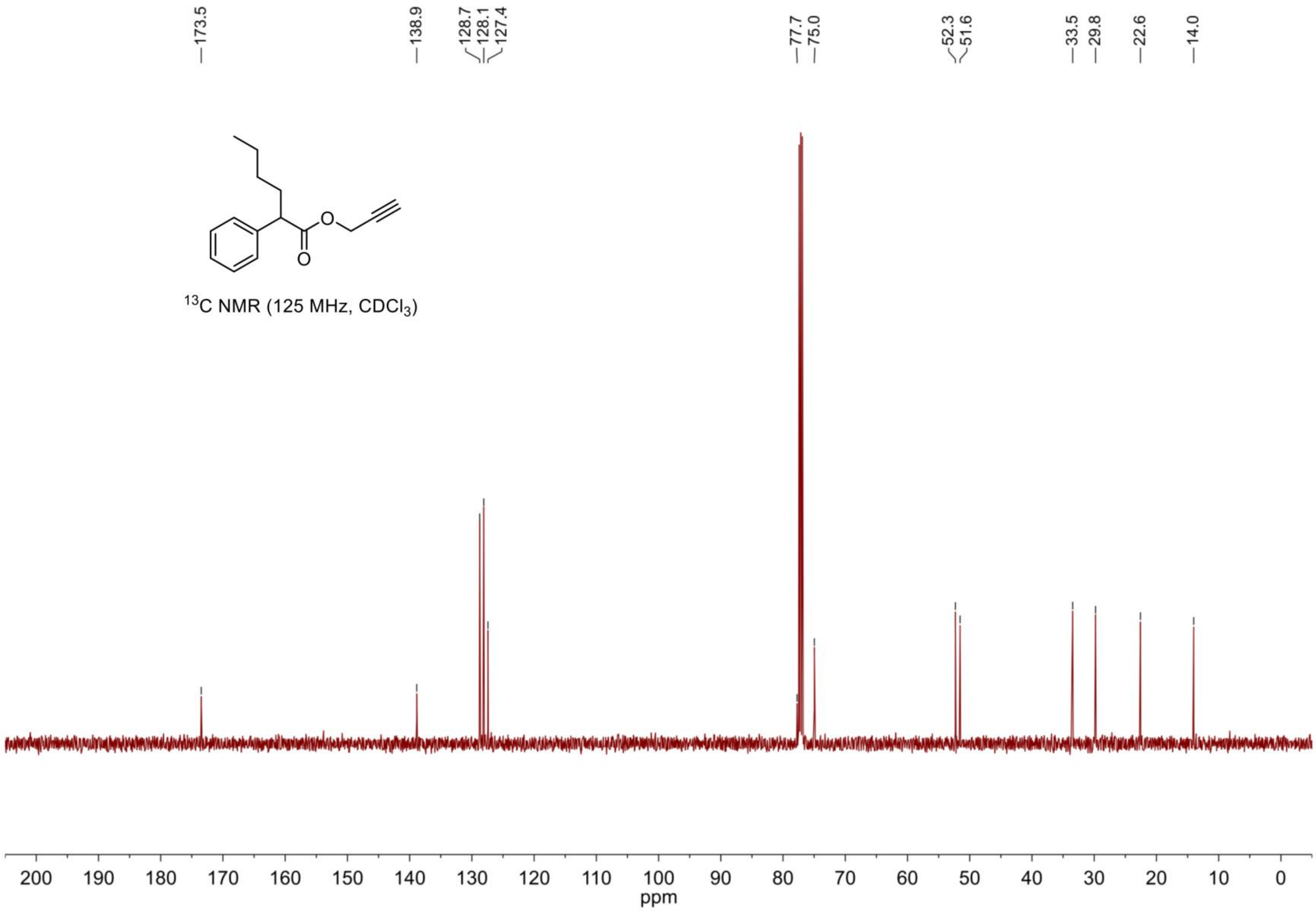

Go back to table of contents

Ethyl 2-ethyl-4-phenylbutanoate (39)

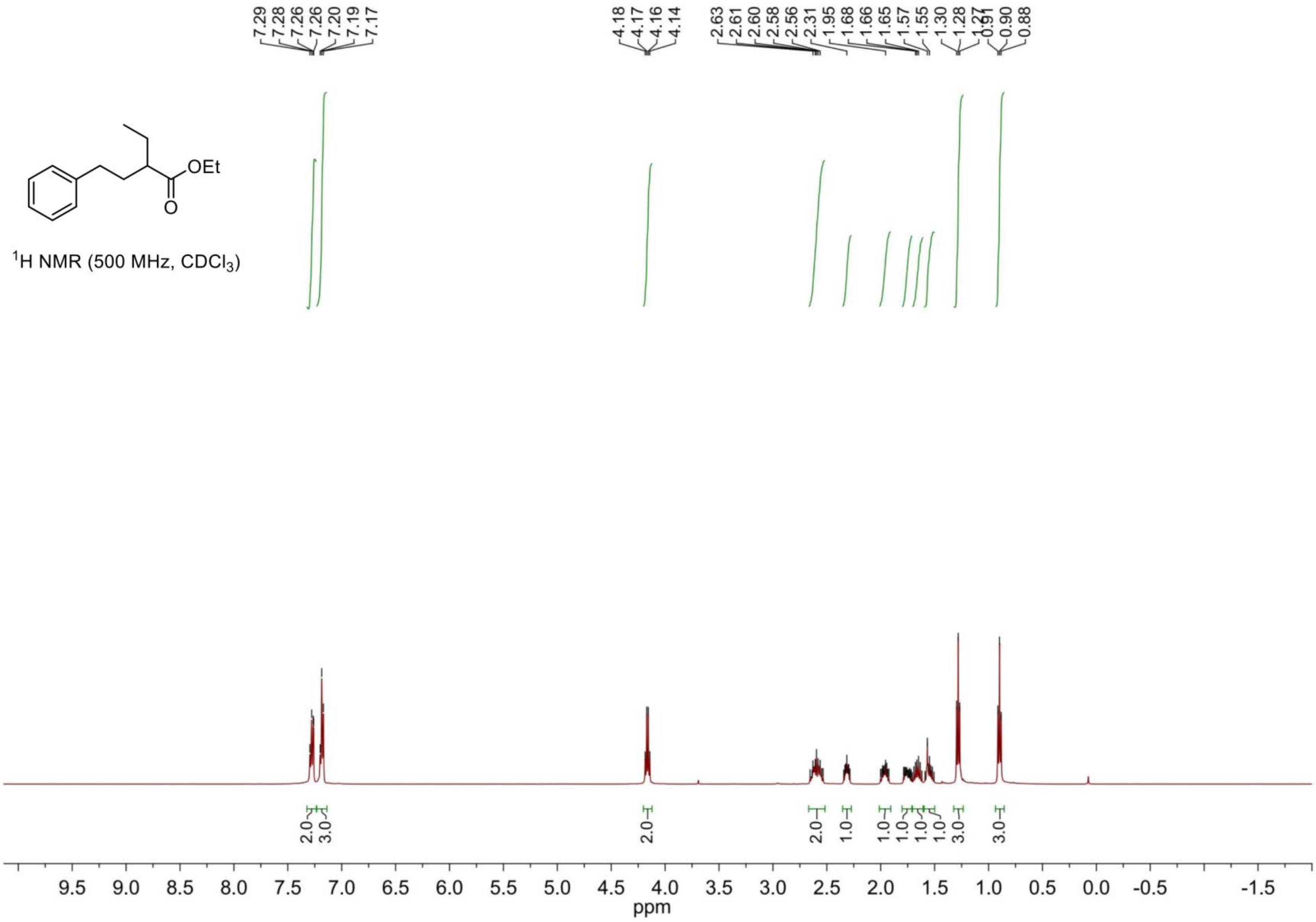

Ethyl 2-ethyl-4-phenylbutanoate (39)

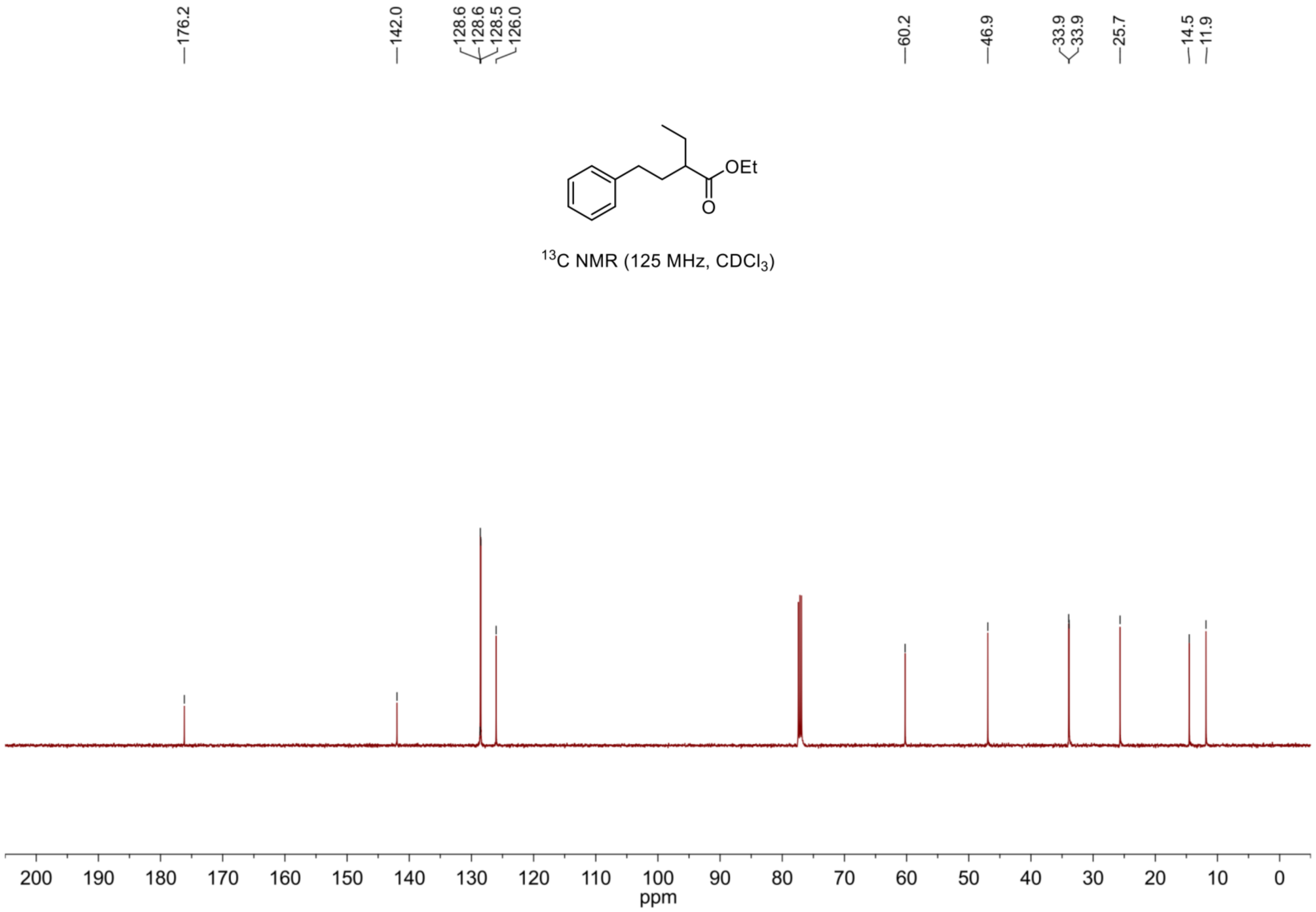

Go back to table of contents

Ethyl 2-ethyl-4-(4-fluorophenyl)butanoate (40)

^{1}H NMR (500 MHz, CDCl$_3$)

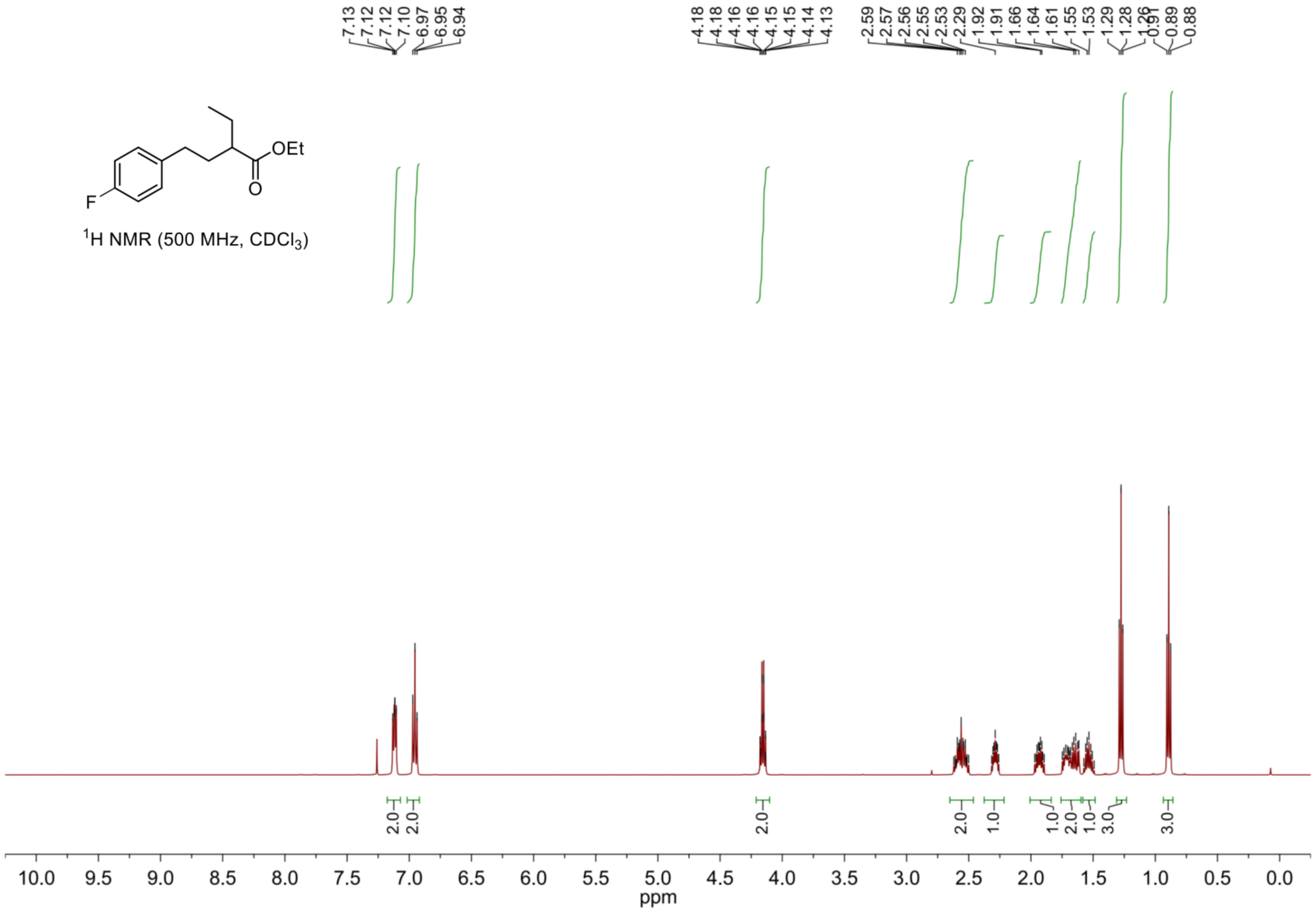

Go back to table of contents

Ethyl 2-ethyl-4-(4-fluorophenyl)butanoate (40)

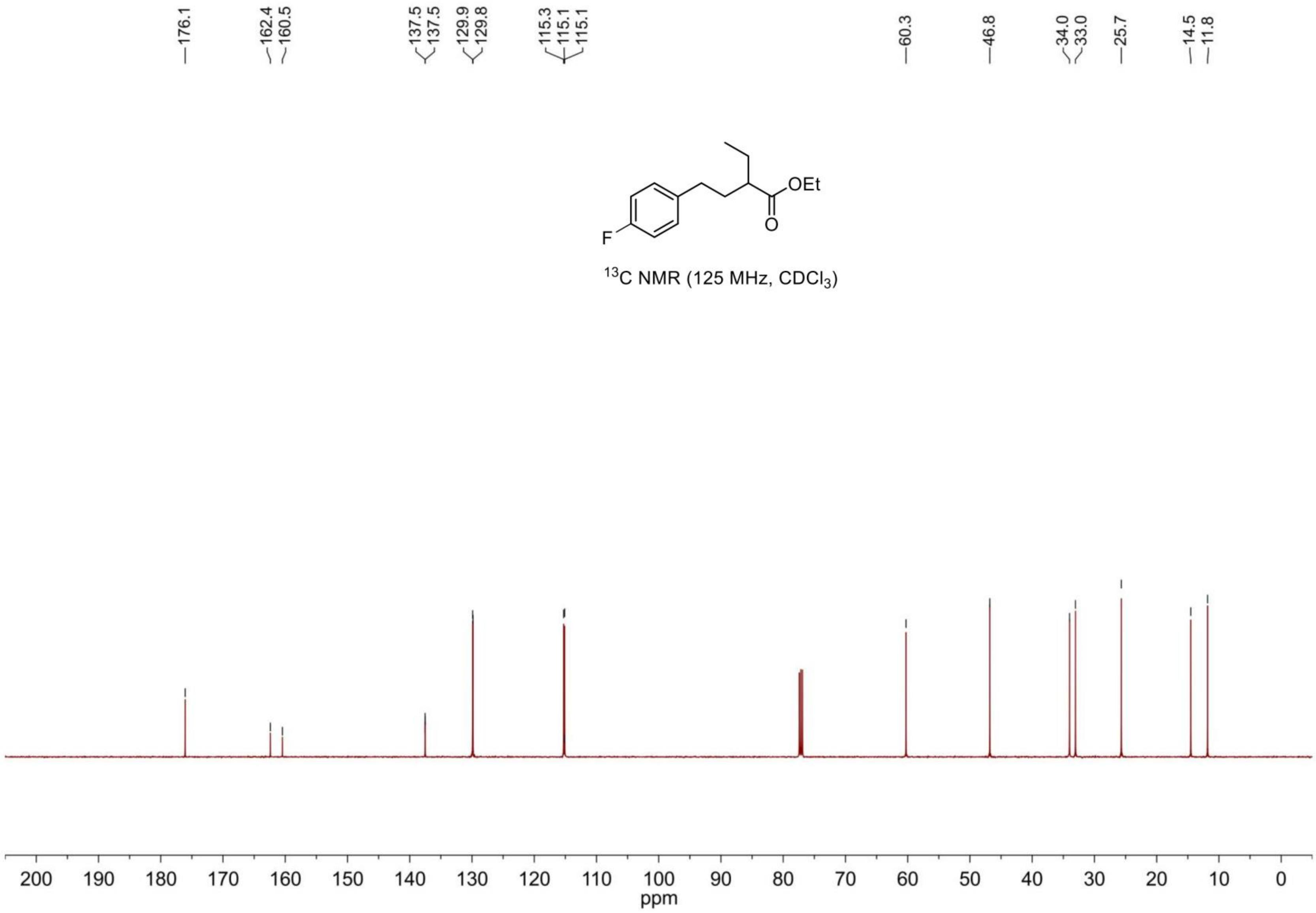

Go back to table of contents

Ethyl 4-(2-chlorophenyl)-2-ethylbutanoate (41)

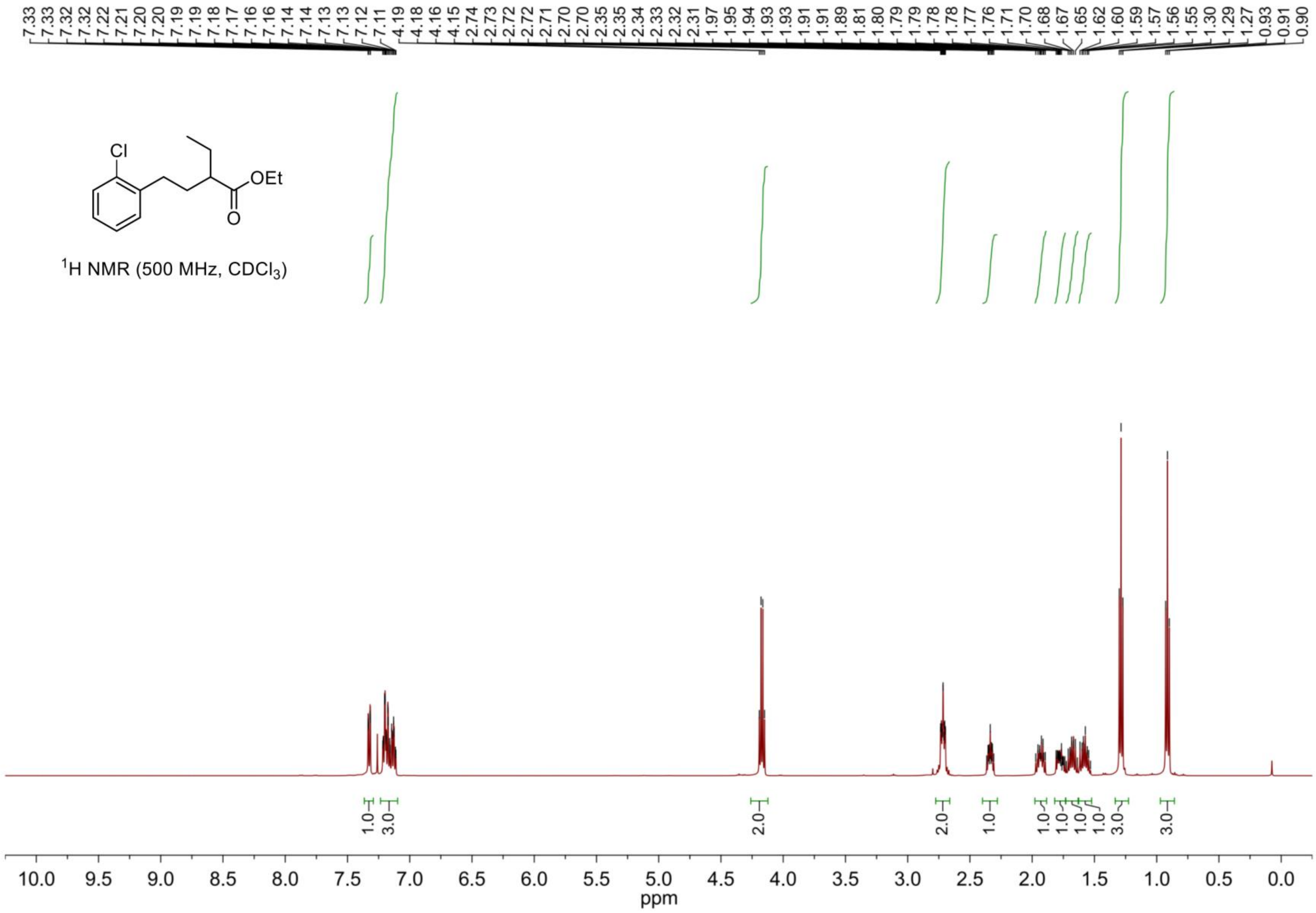

Go back to table of contents

Ethyl 4-(2-chlorophenyl)-2-ethylbutanoate (41)

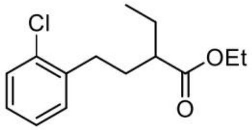

^{13}C NMR (125 MHz, $CDCl_3$)

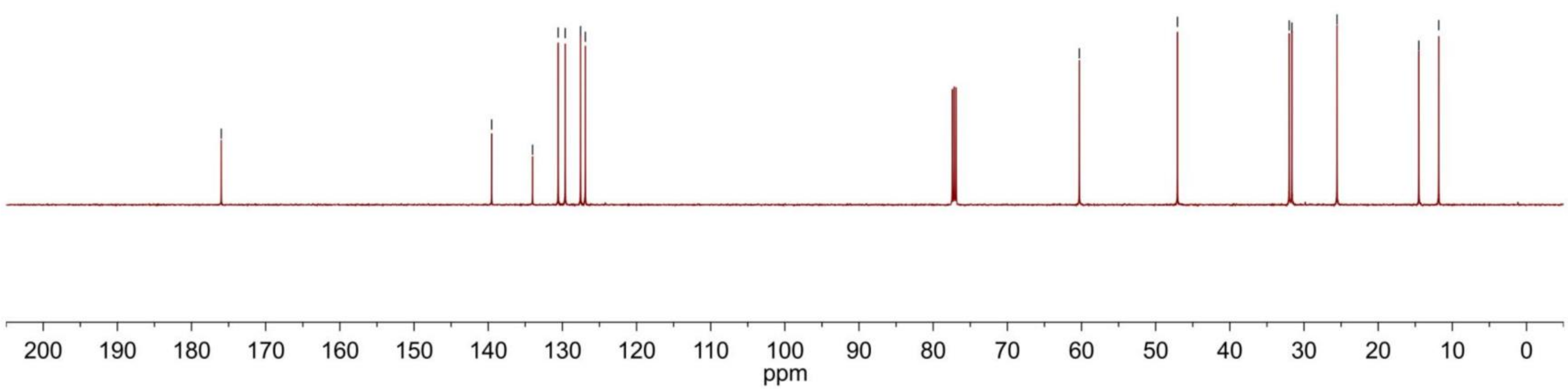

Go back to table of contents

Ethyl 4-(2,3-dihydrobenzofuran-5-yl)-2-ethylbutanoate (42)

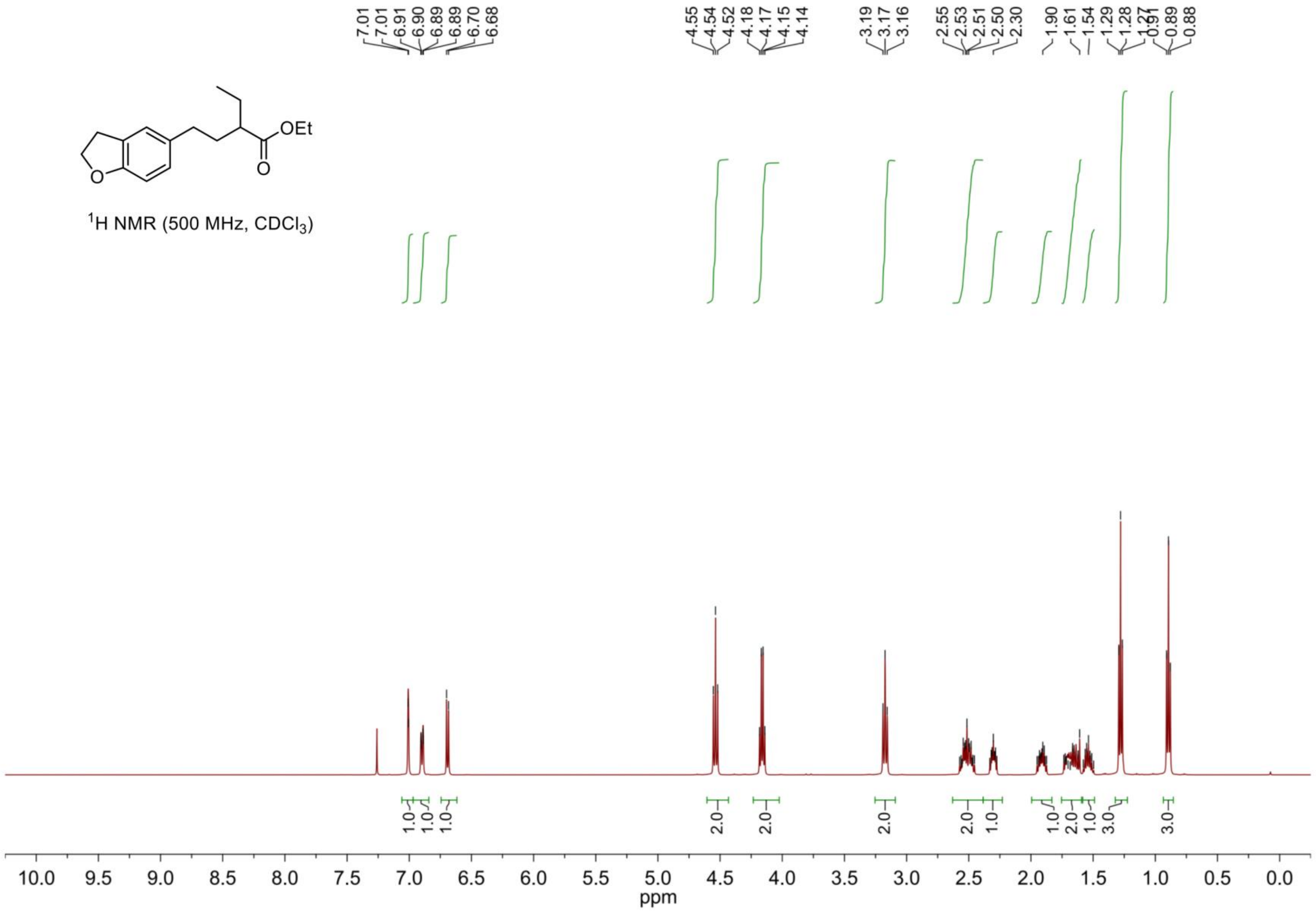

Go back to table of contents

Ethyl 4-(2,3-dihydrobenzofuran-5-yl)-2-ethylbutanoate (42)

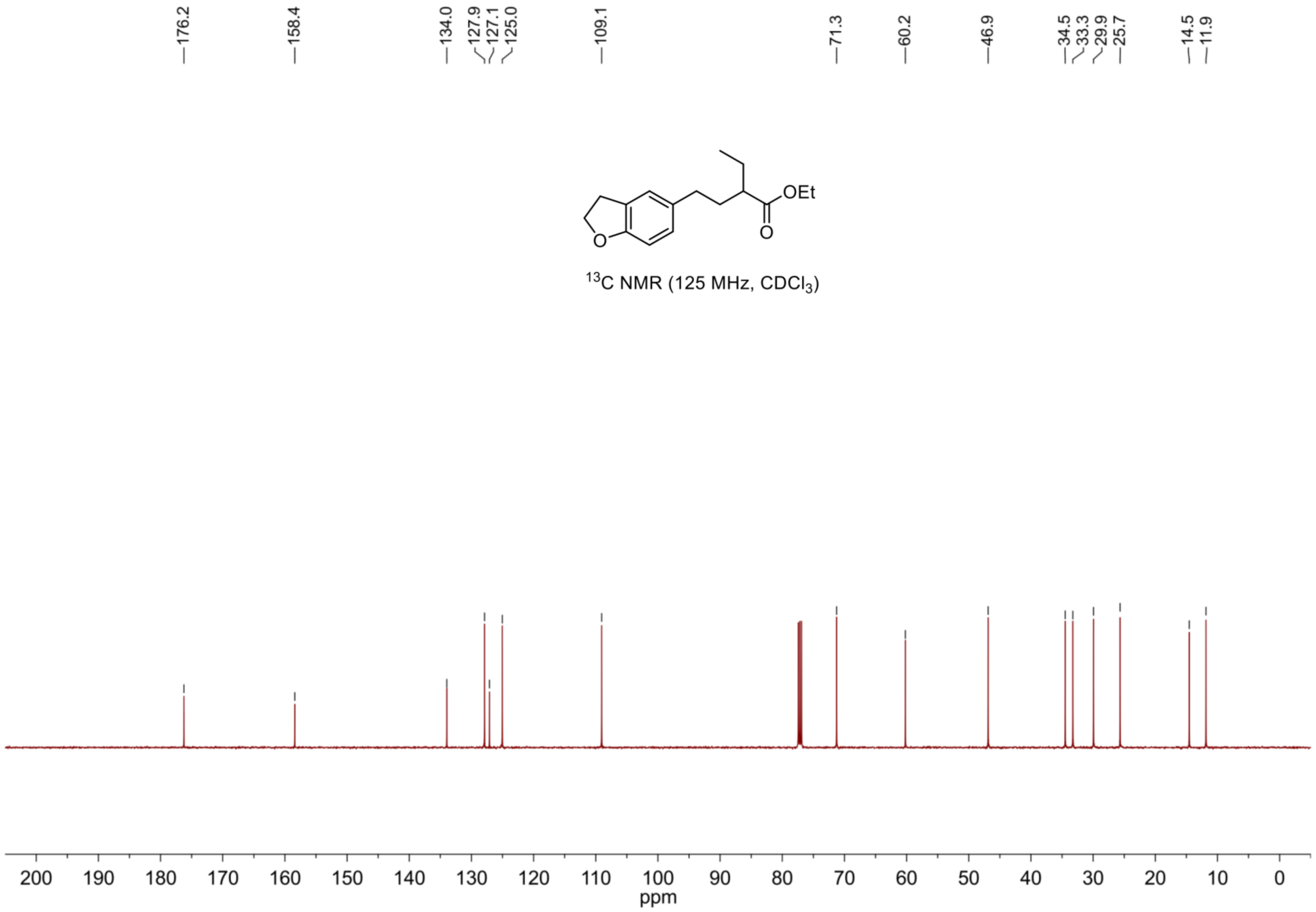

Go back to table of contents

1,2-Diphenylbutan-1-one (43)

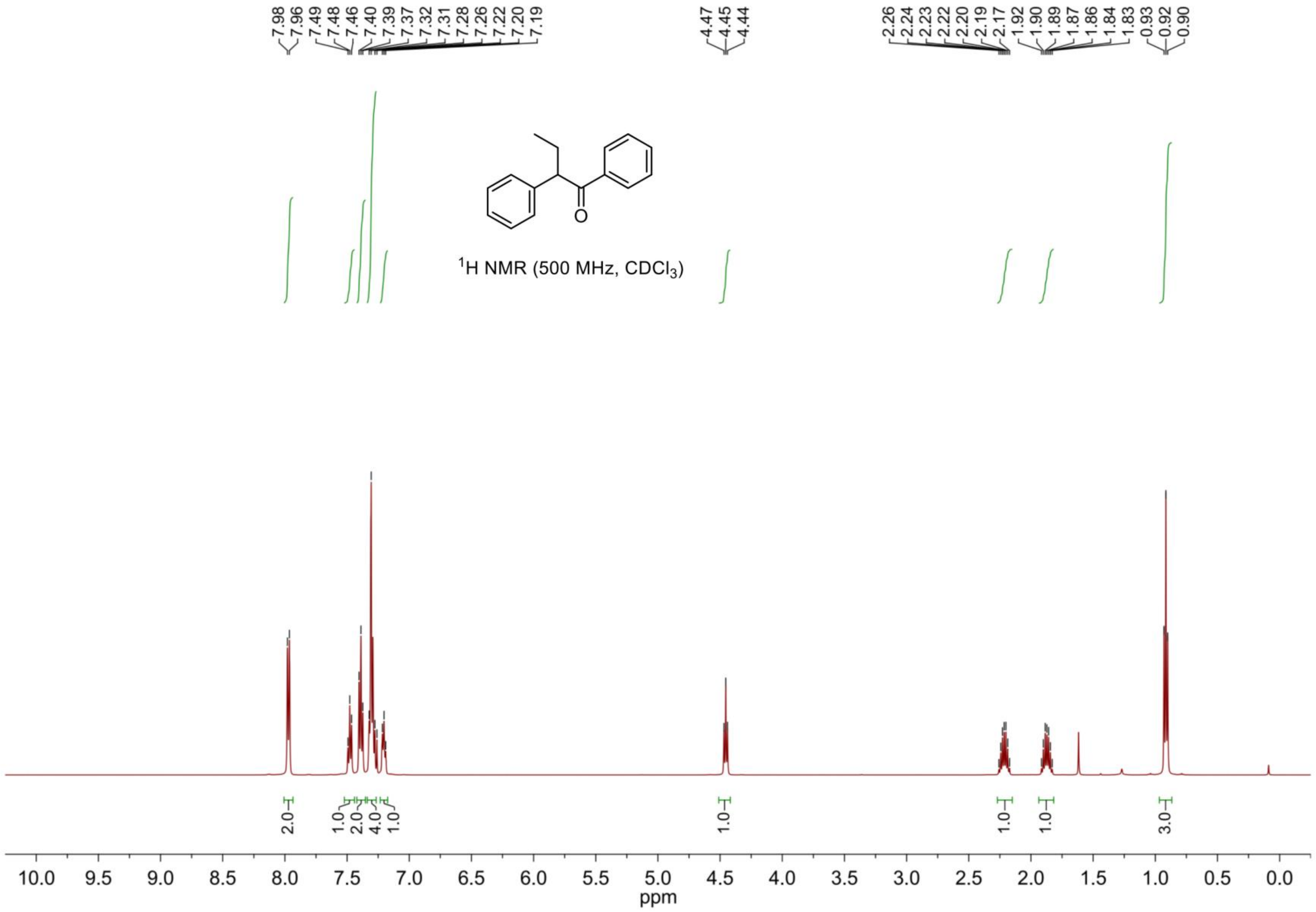

Go back to table of contents

1,2-Diphenylbutan-1-one (43)

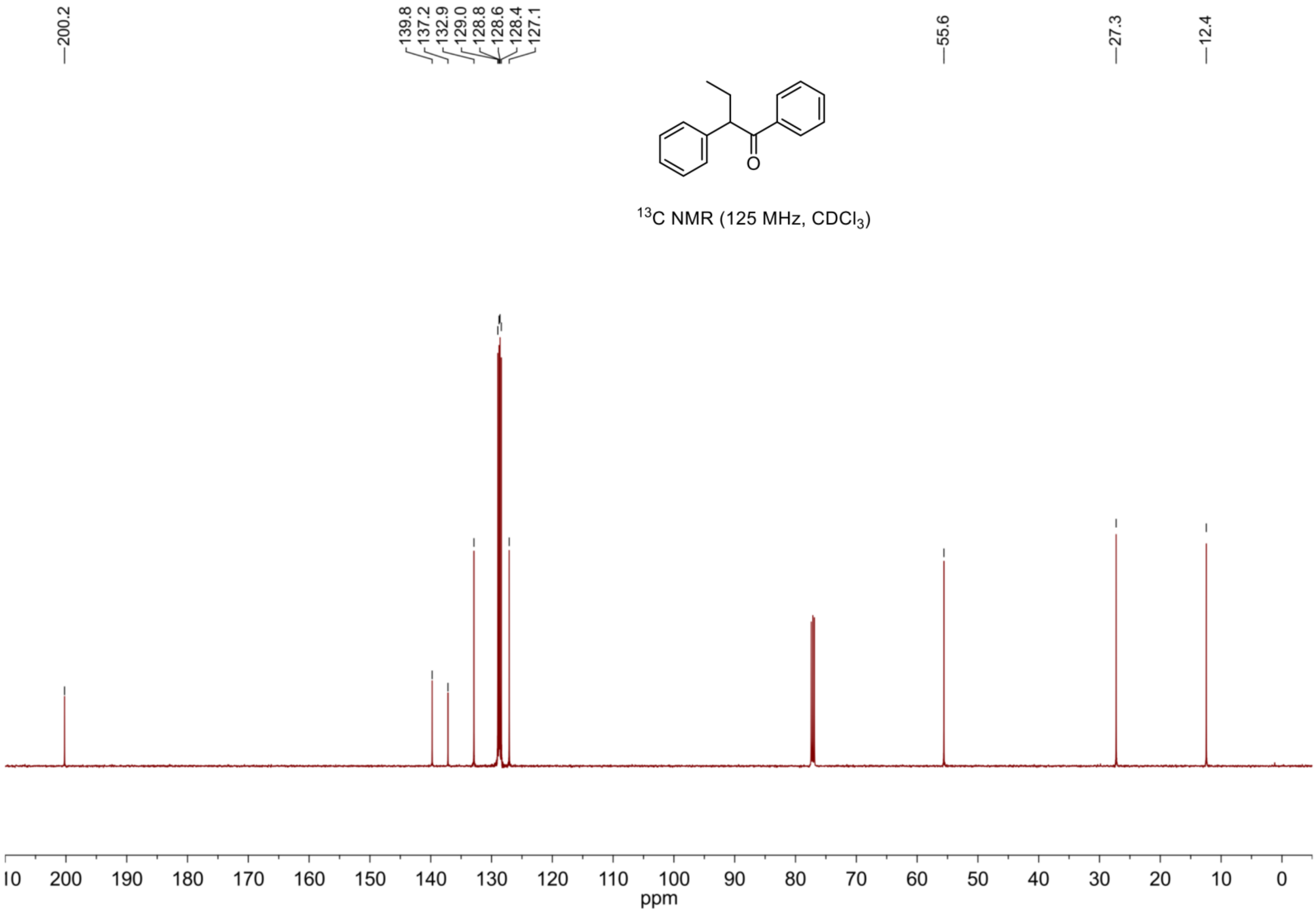

Go back to table of contents

1,2-Bis(4-methoxyphenyl)butan-1-one (44)

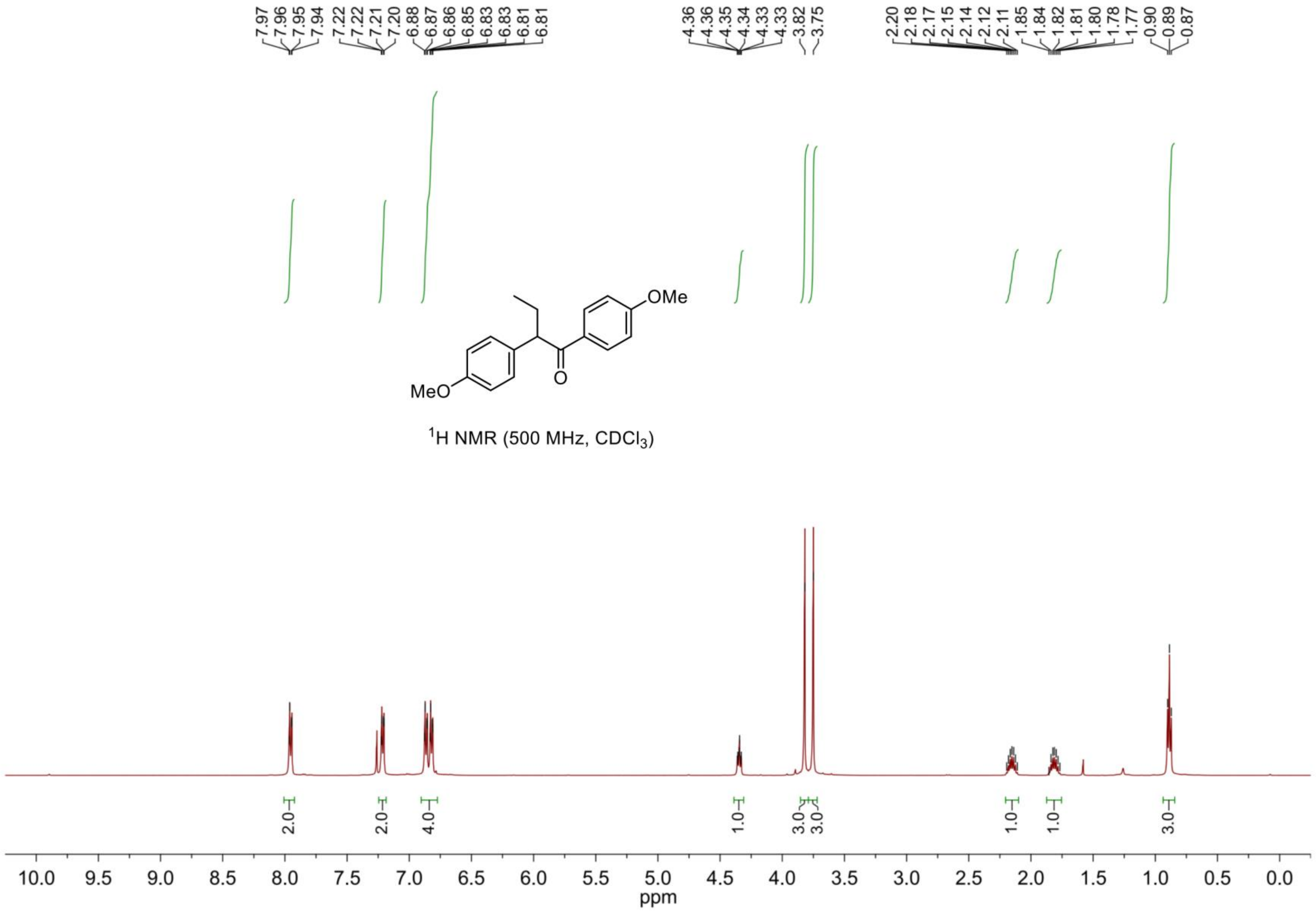

Go back to table of contents

1,2-Bis(4-methoxyphenyl)butan-1-one (44)

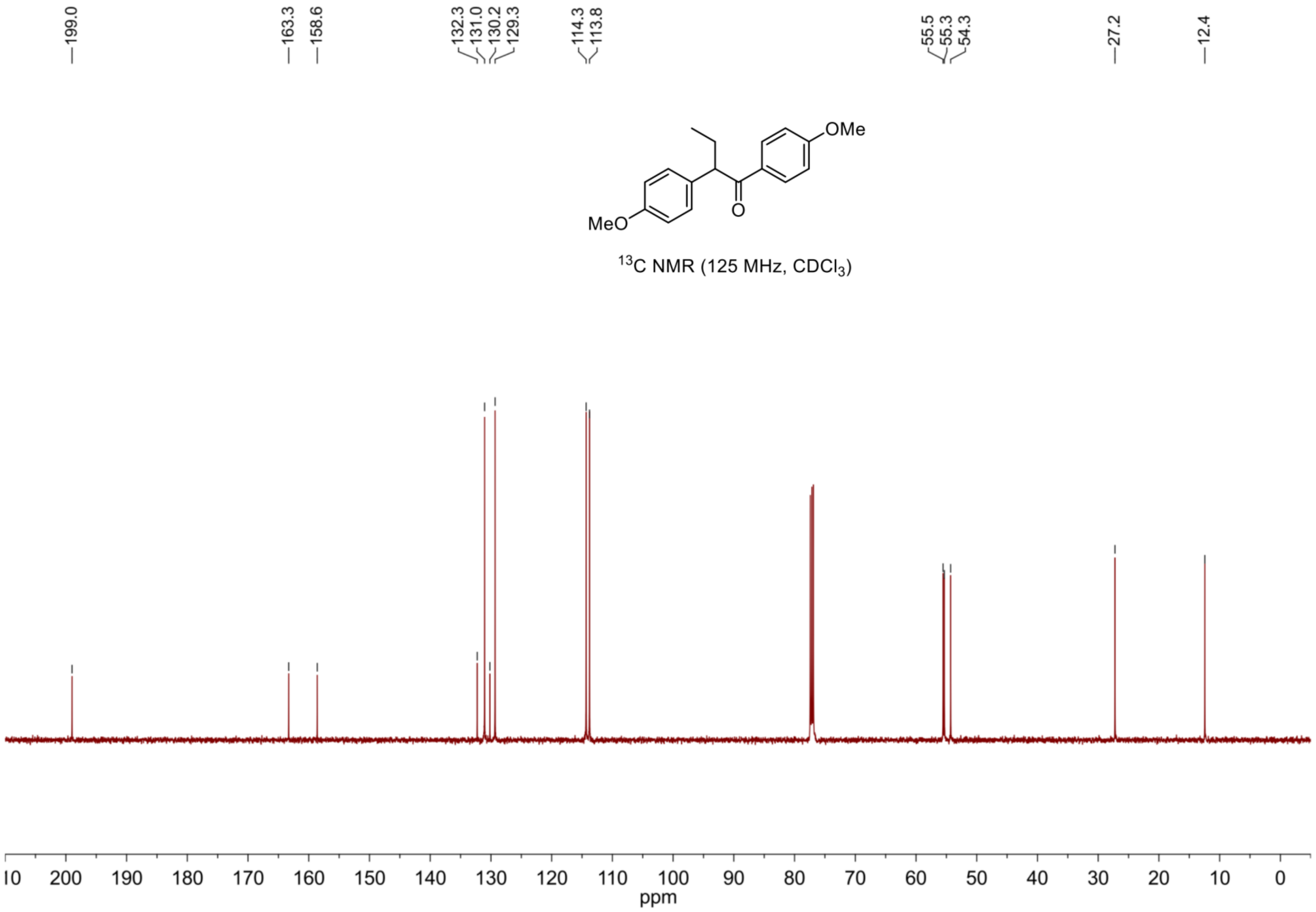

Go back to table of contents

1,2-Bis(3-methoxyphenyl)butan-1-one (45)

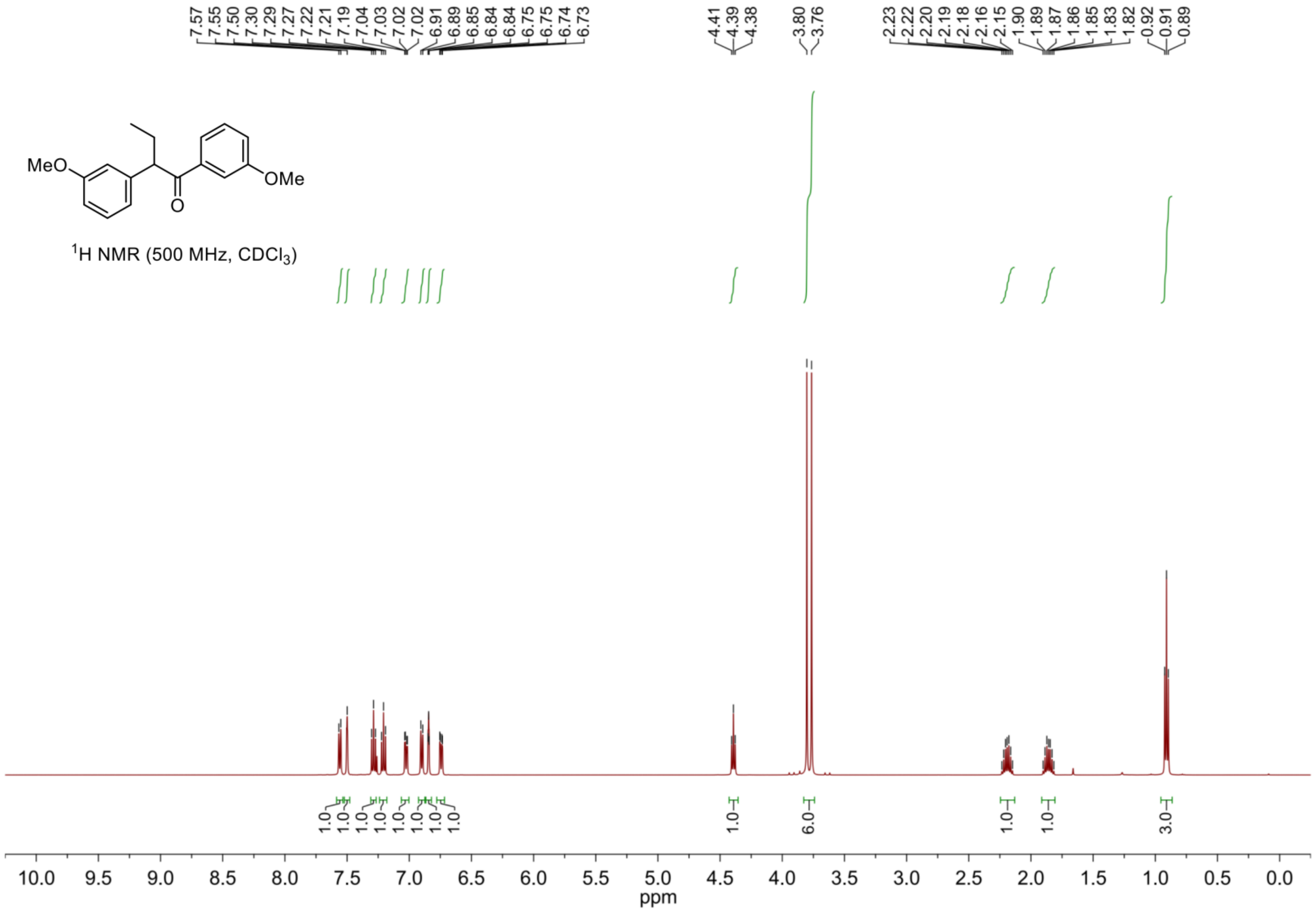

Go back to table of contents

1,2-Bis(3-methoxyphenyl)butan-1-one (45)

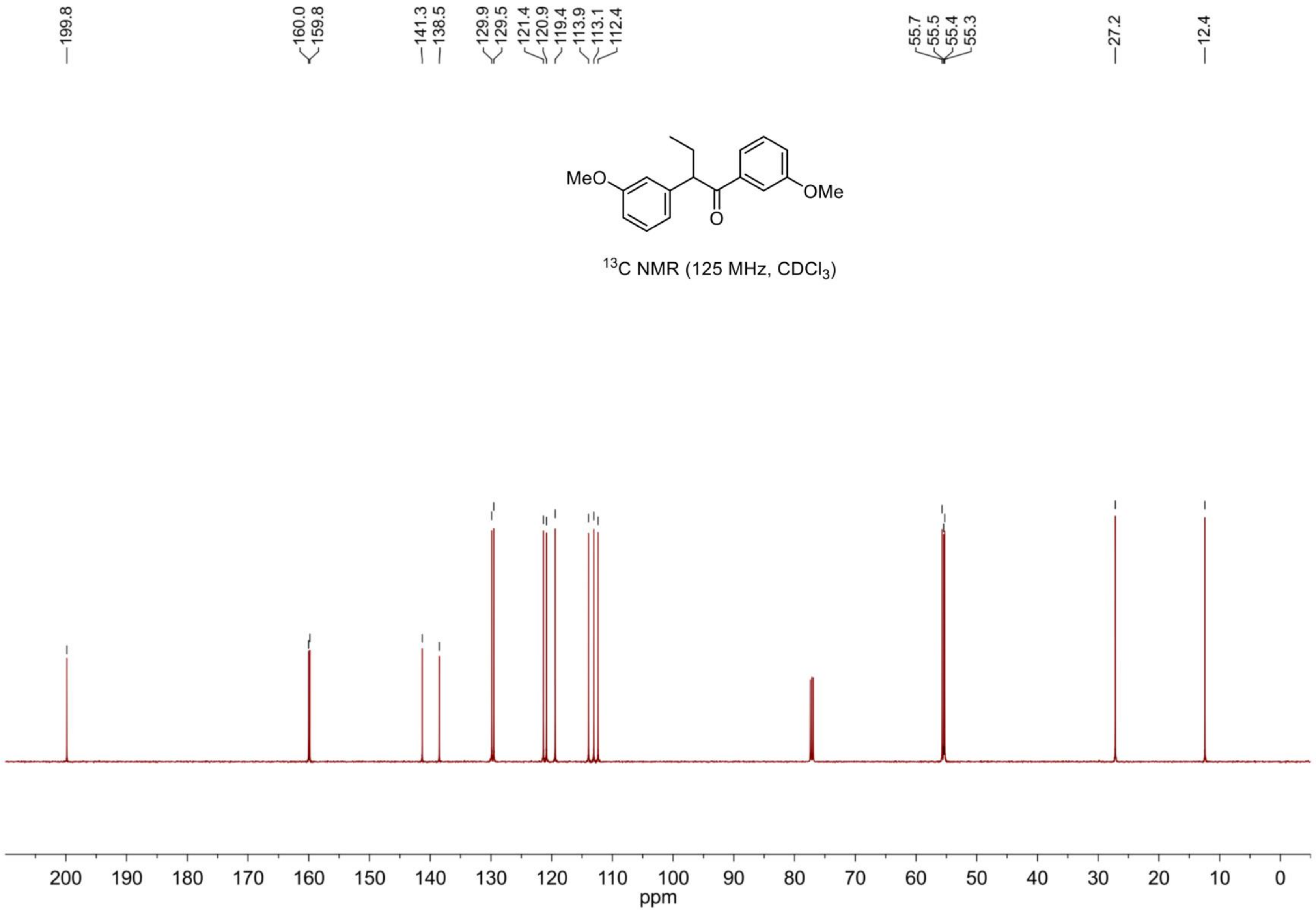

Go back to table of contents

1,2-Di-*p*-tolylbutan-1-one (46)

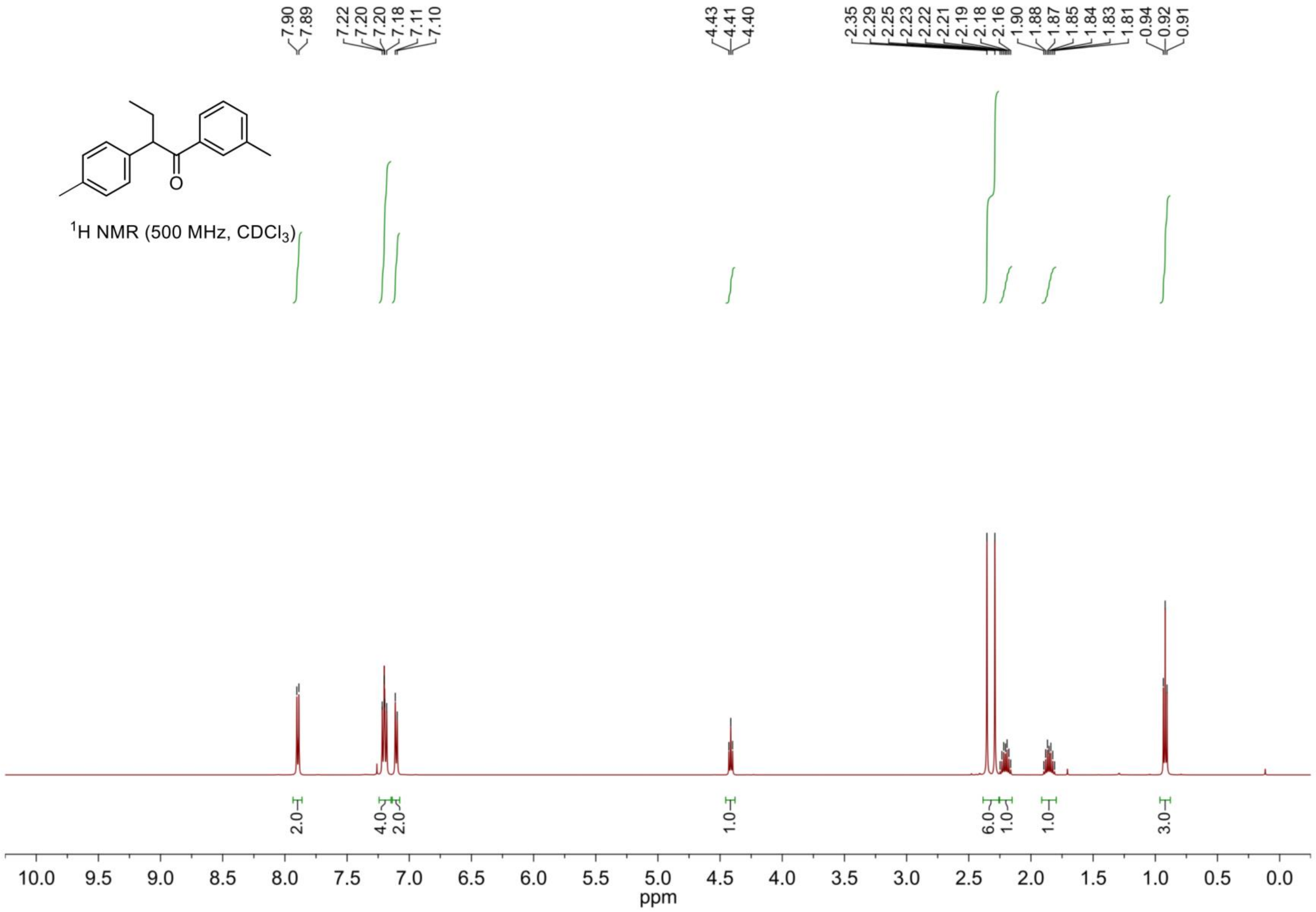

Go back to table of contents

1,2-Di-*p*-tolylbutan-1-one (46)

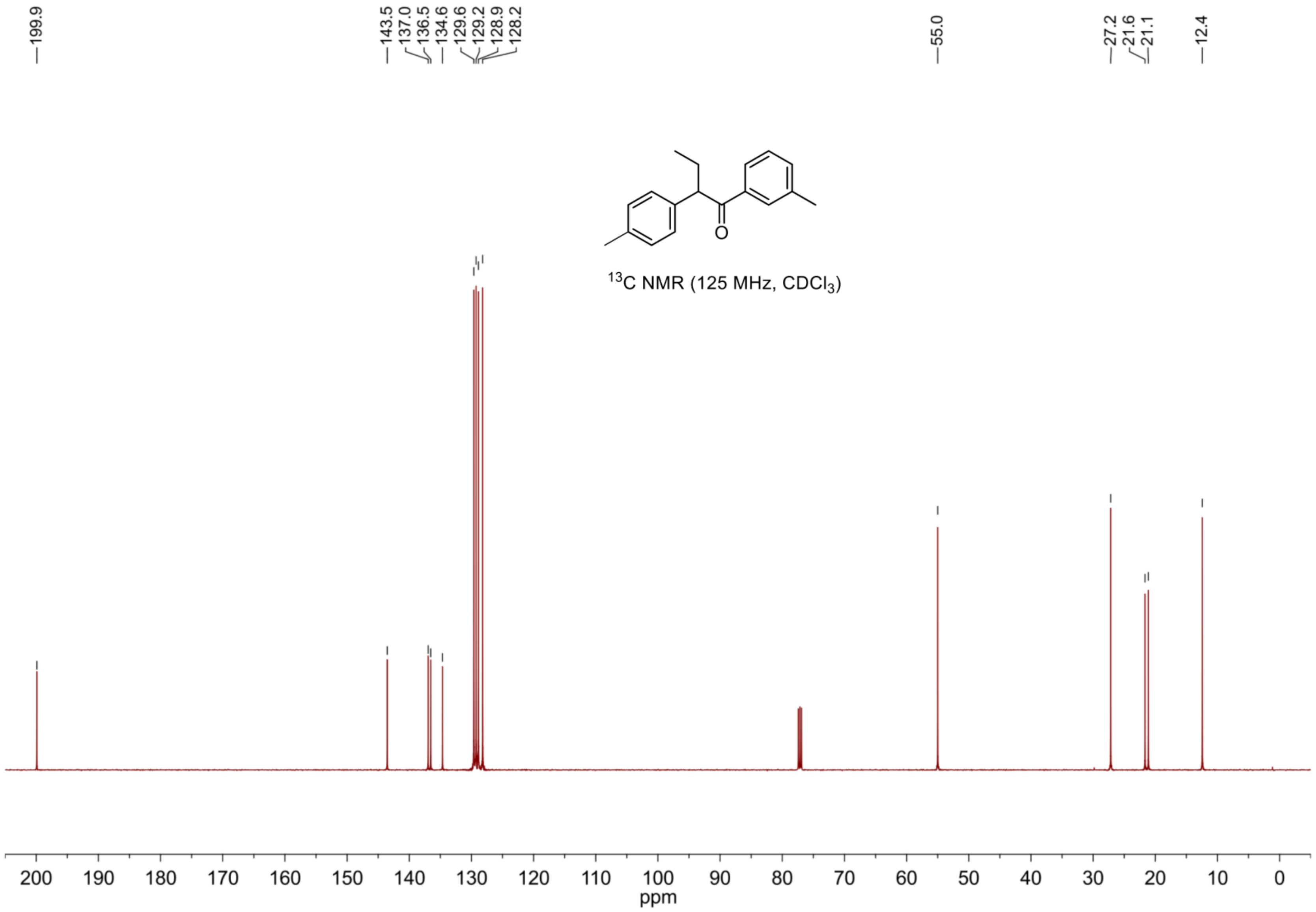

Go back to table of contents

1,2-Bis(4-fluorophenyl)butan-1-one (47)

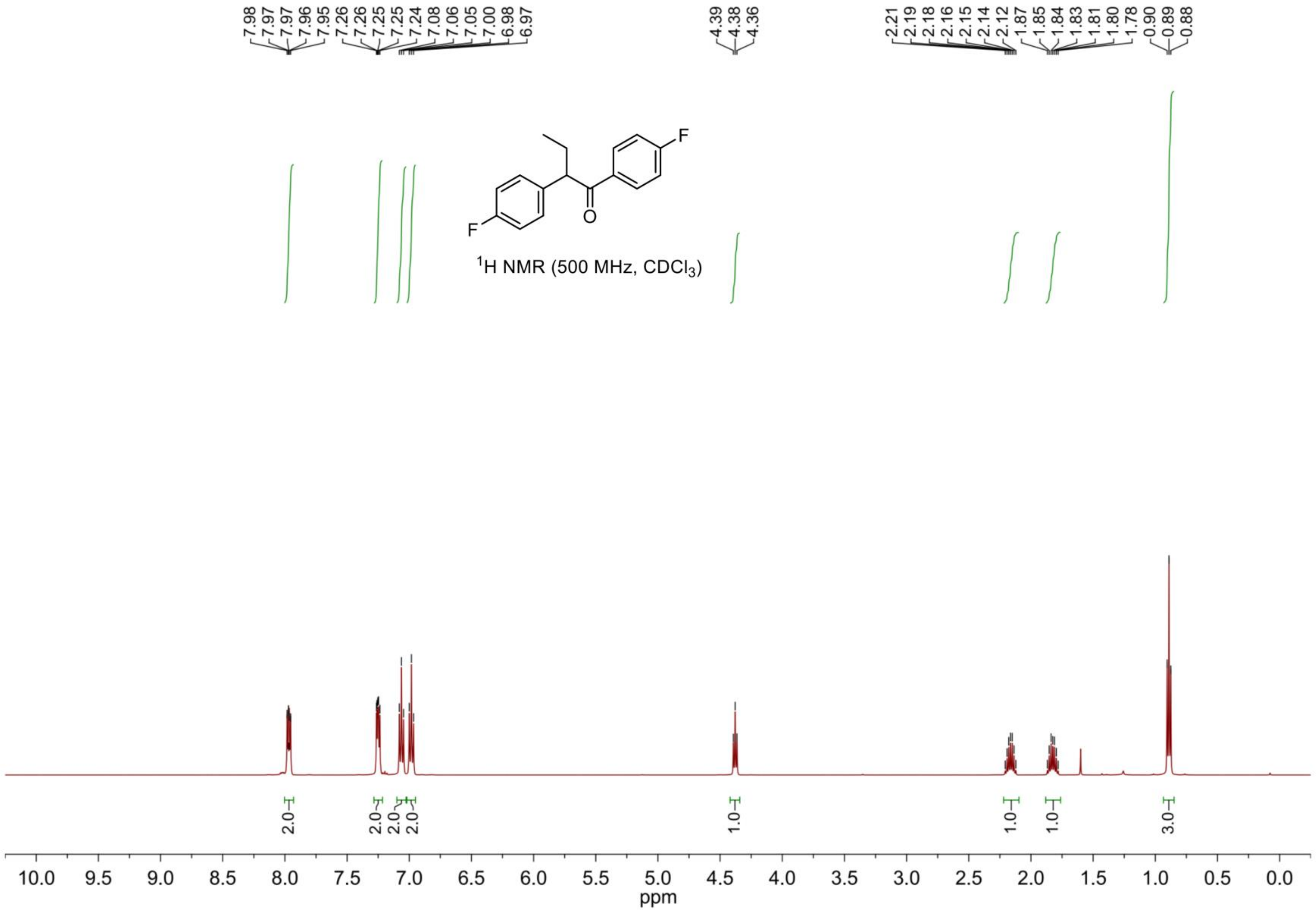

Go back to table of contents

1,2-Bis(4-fluorophenyl)butan-1-one (47)

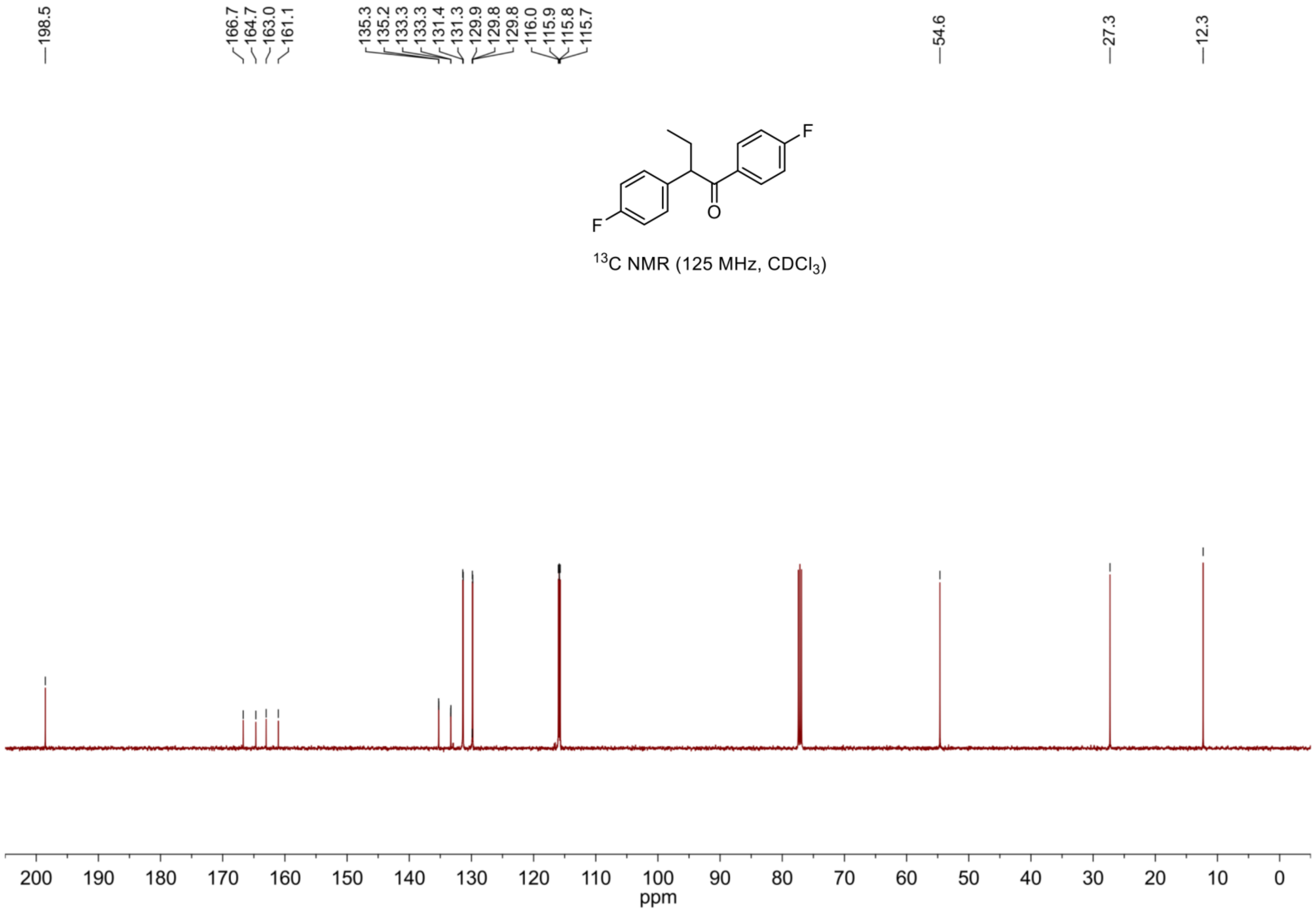

Go back to table of contents

1,2-Di(thiophen-2-yl)butan-1-one (48)

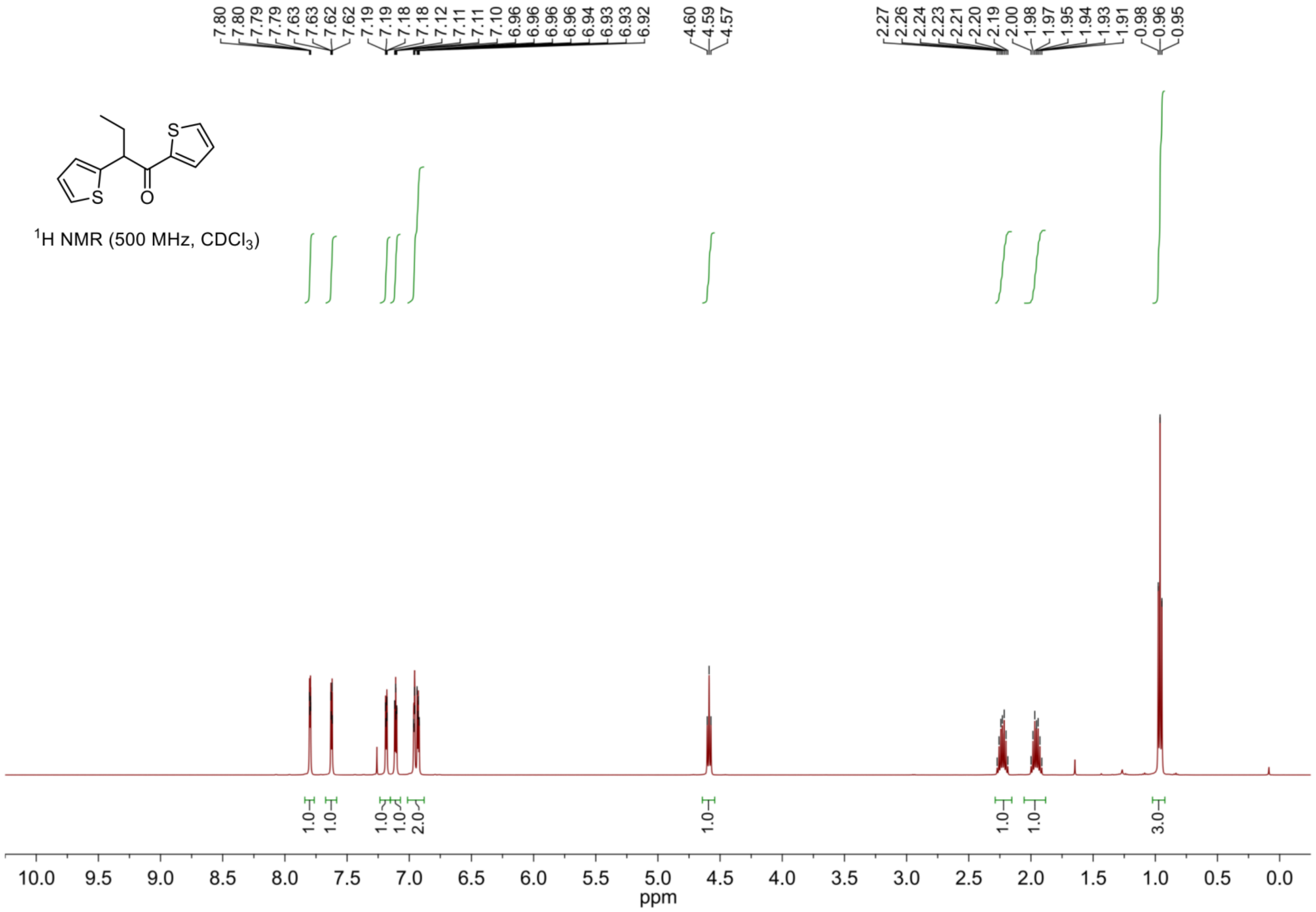

Go back to table of contents

1,2-Di(thiophen-2-yl)butan-1-one (48)

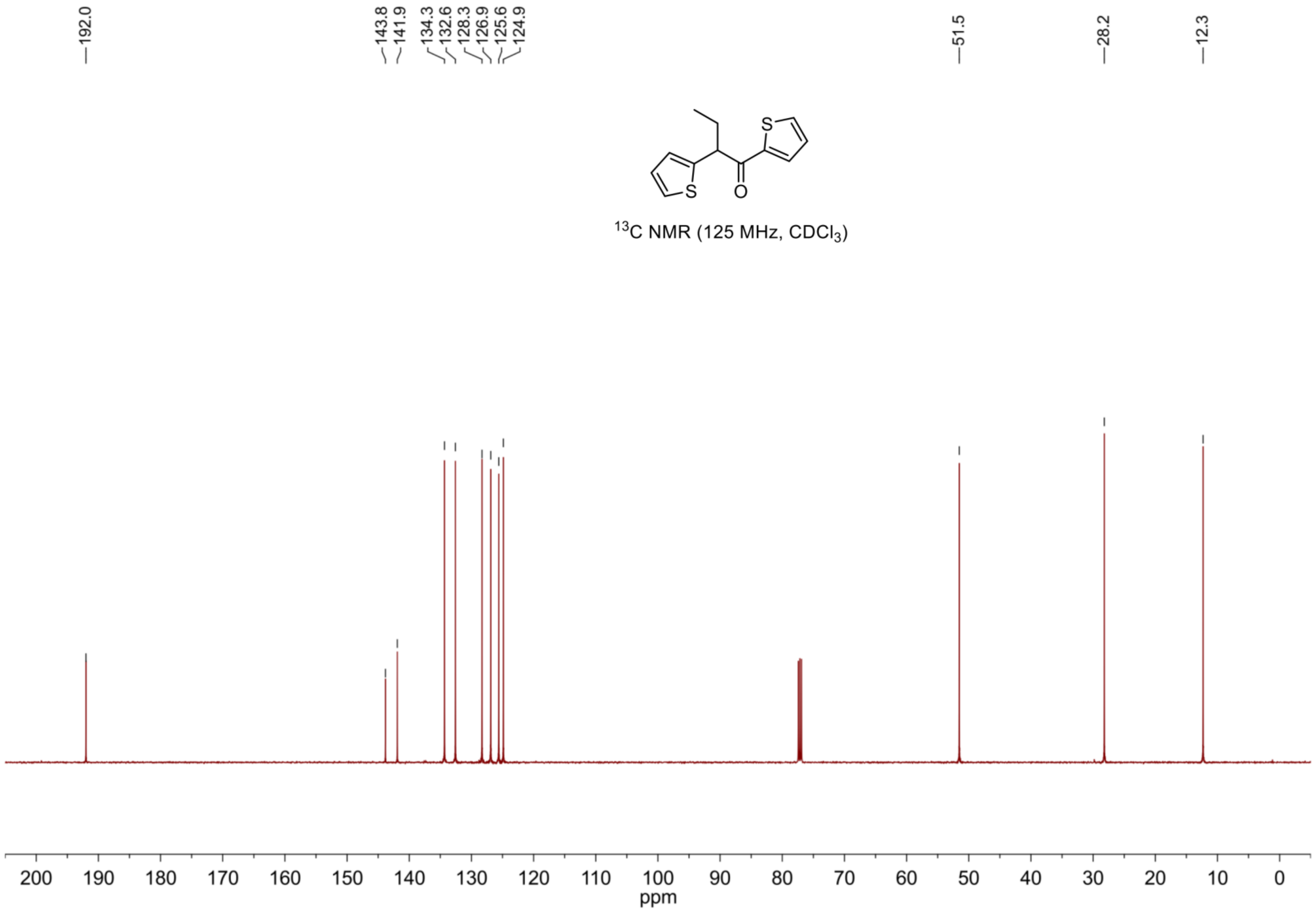

Go back to table of contents

1,2-Di(furan-2-yl)butan-1-one (49)

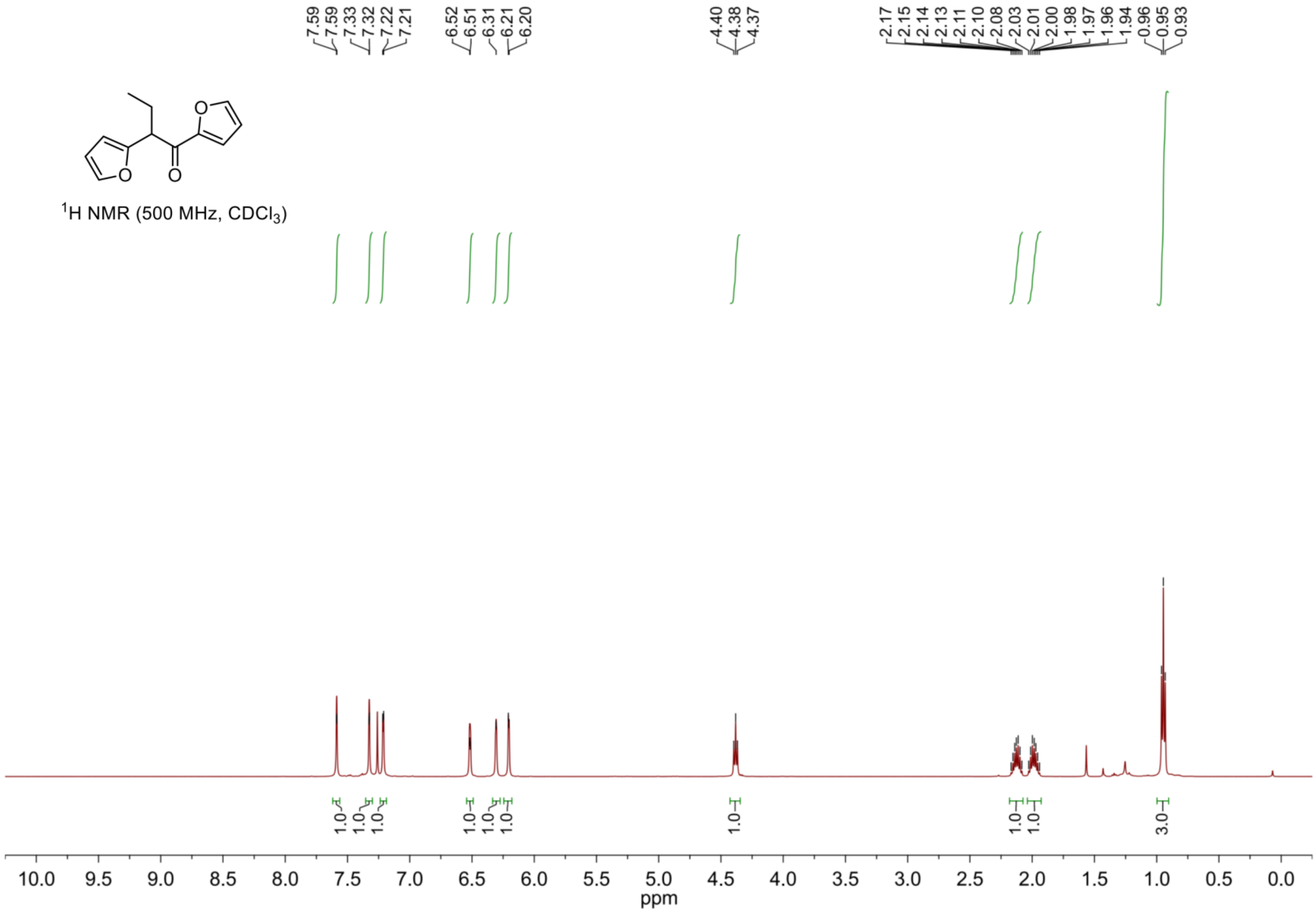

Go back to table of contents

1,2-Di(furan-2-yl)butan-1-one (49)

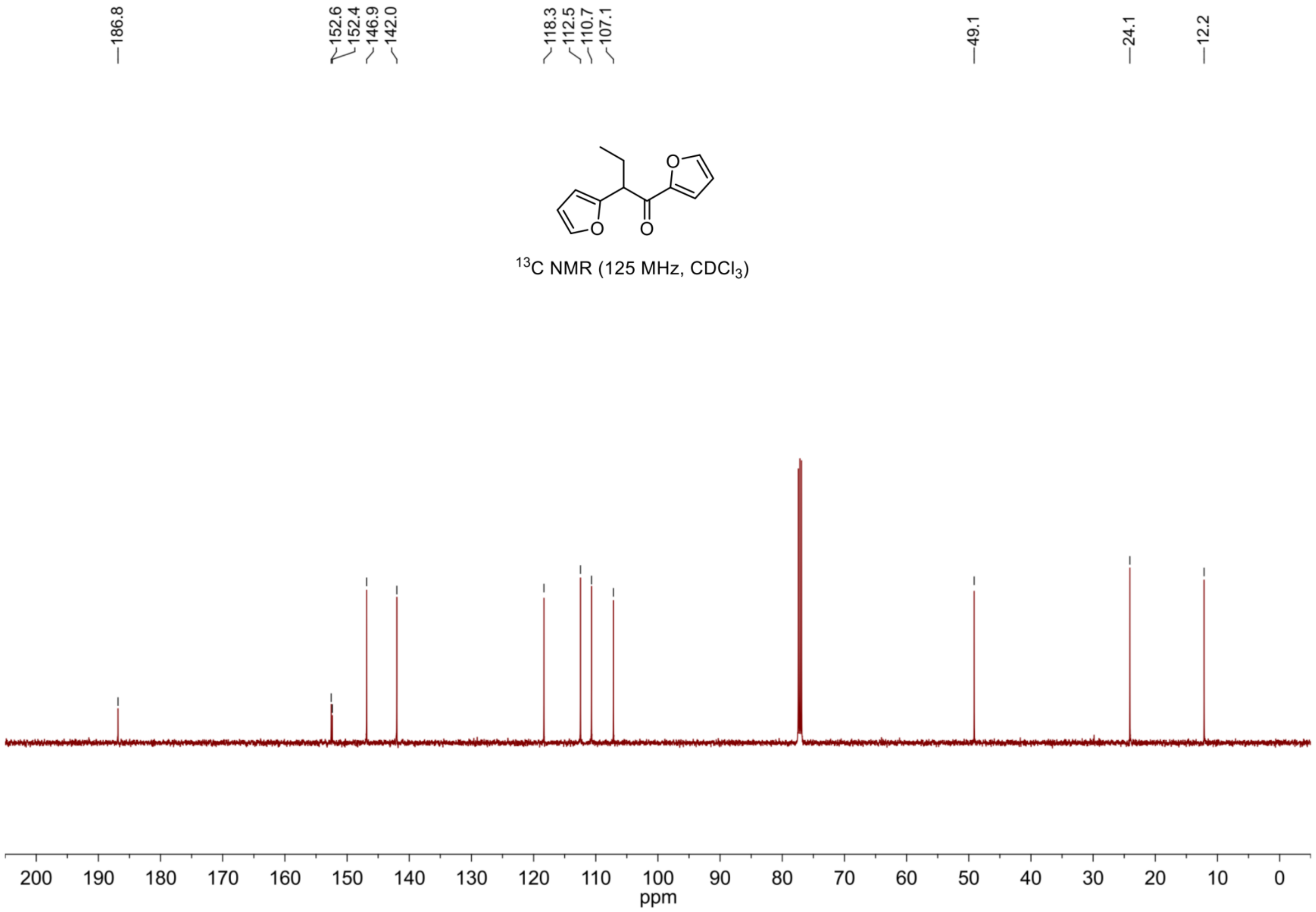

Go back to table of contents

Ethyl 2,2-diphenylacetate (50)

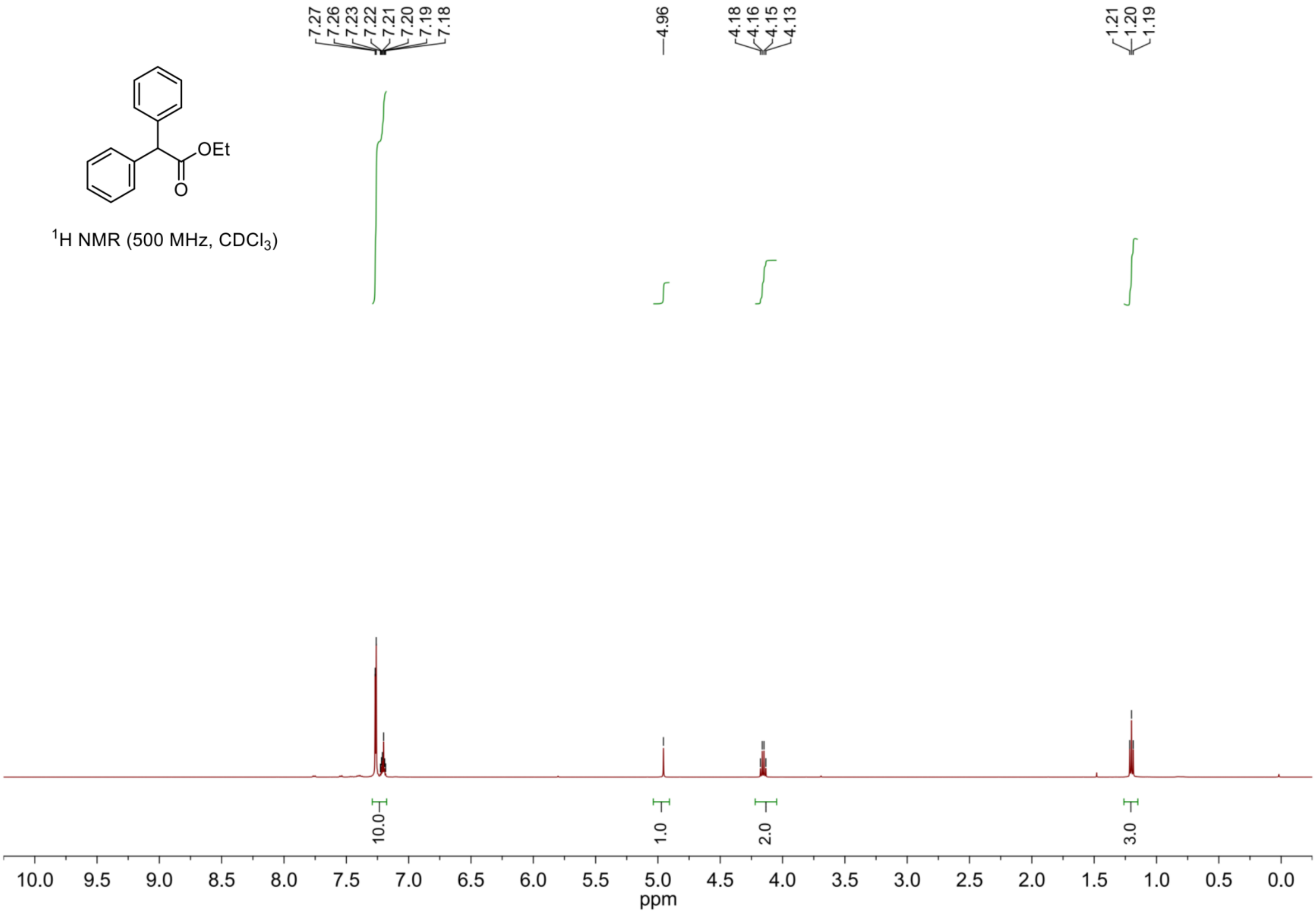

Go back to table of contents

Ethyl 2,2-diphenylacetate (50)

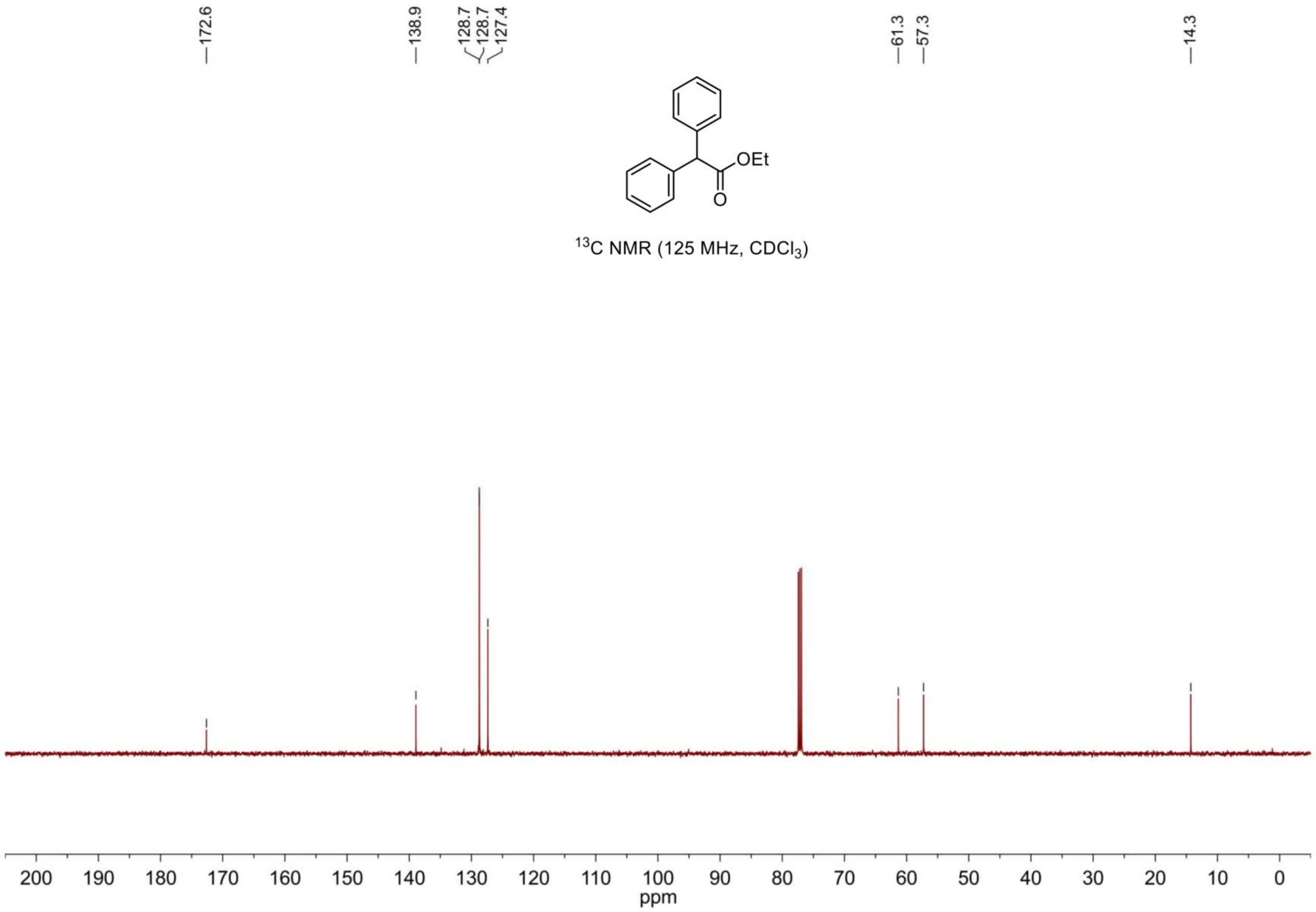

Go back to table of contents

Ethyl 2-(4-methoxyphenyl)-2-(*p*-tolyl)acetate (51)

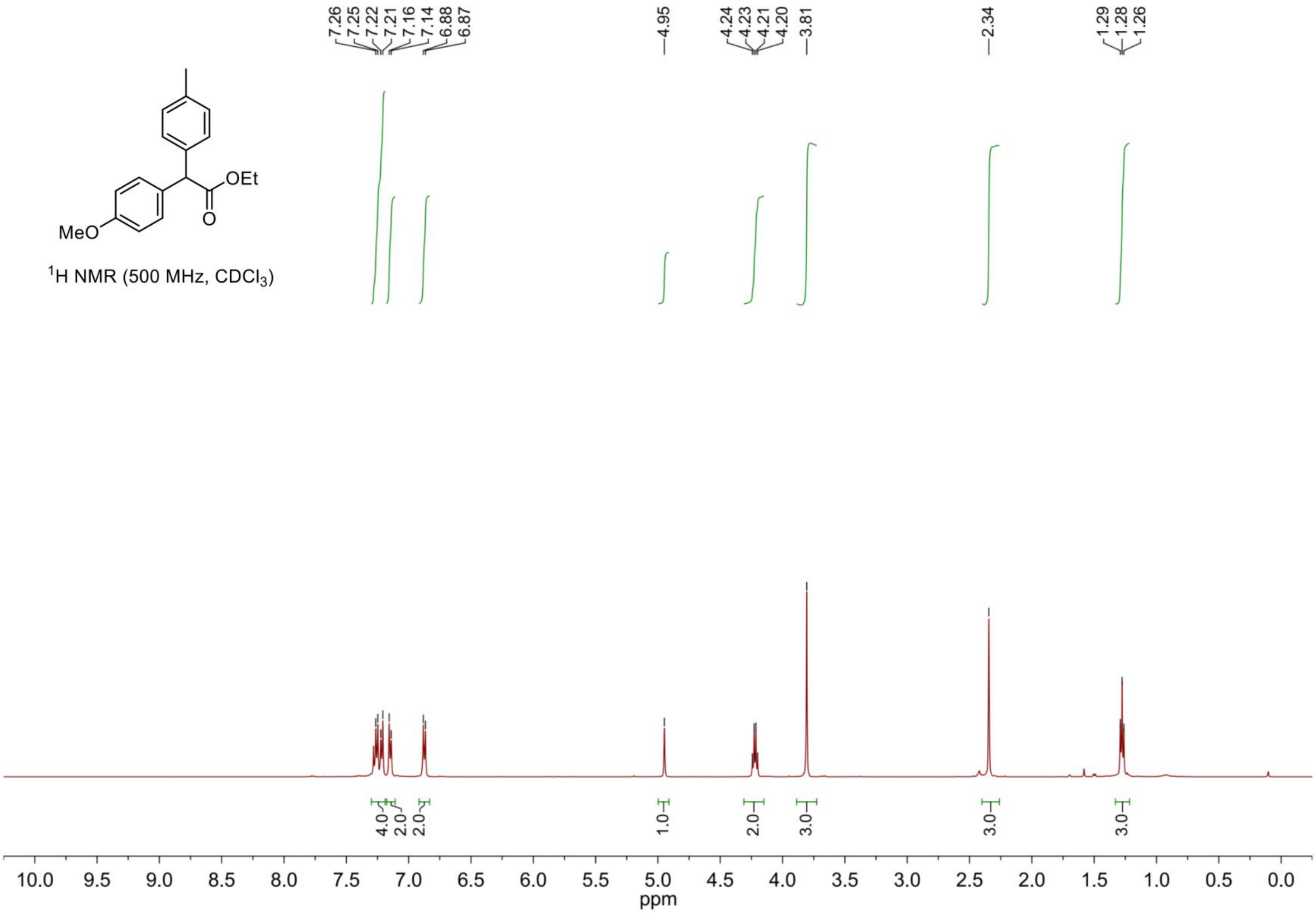

Ethyl 2-(4-methoxyphenyl)-2-(*p*-tolyl)acetate (51)

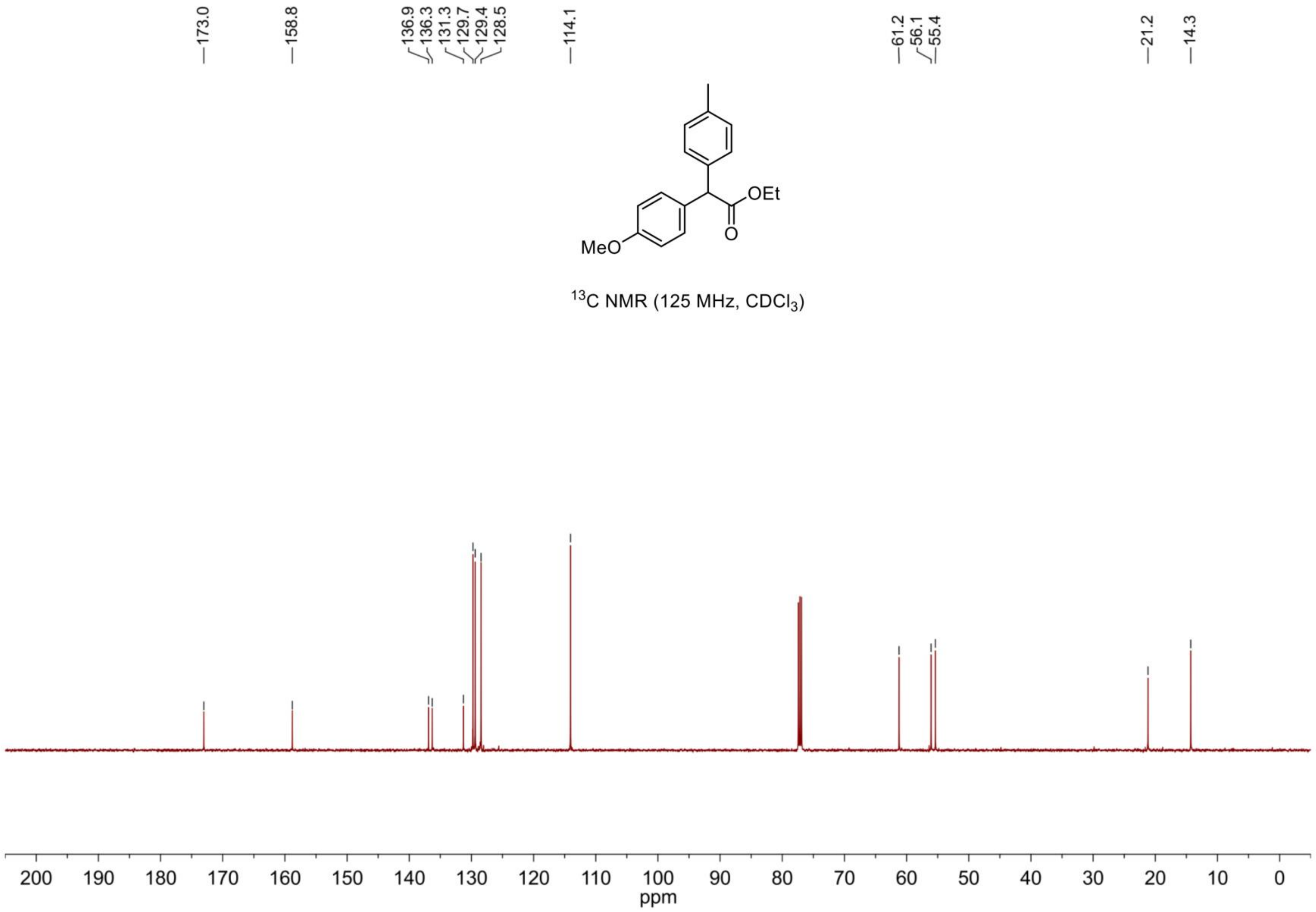

Go back to table of contents

Ethyl 2-(3,5-dimethylphenyl)-2-(3-(trifluoromethoxy)phenyl)acetate (52)

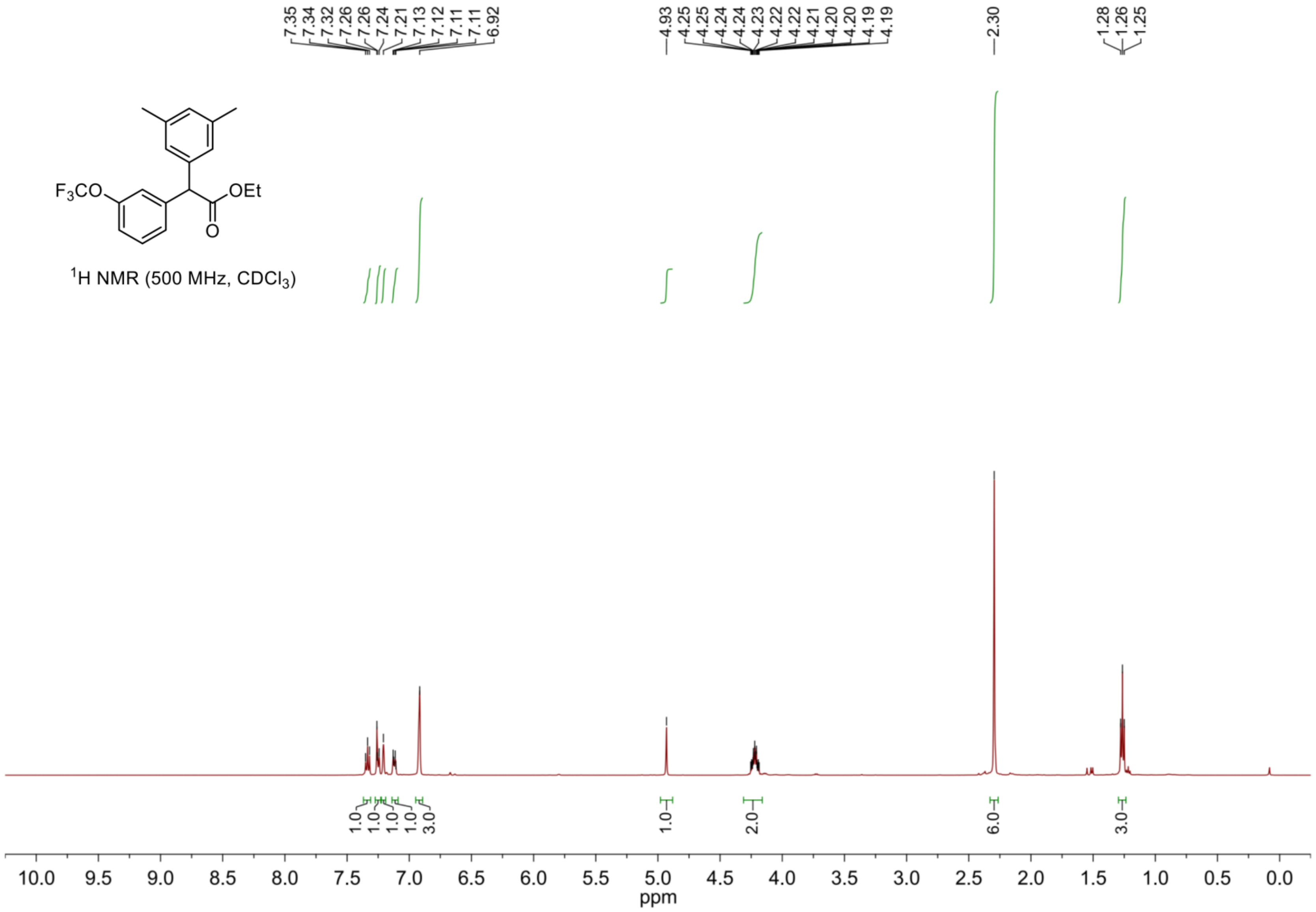

Go back to table of contents

Ethyl 2-(3,5-dimethylphenyl)-2-(3-(trifluoromethoxy)phenyl)acetate (52)

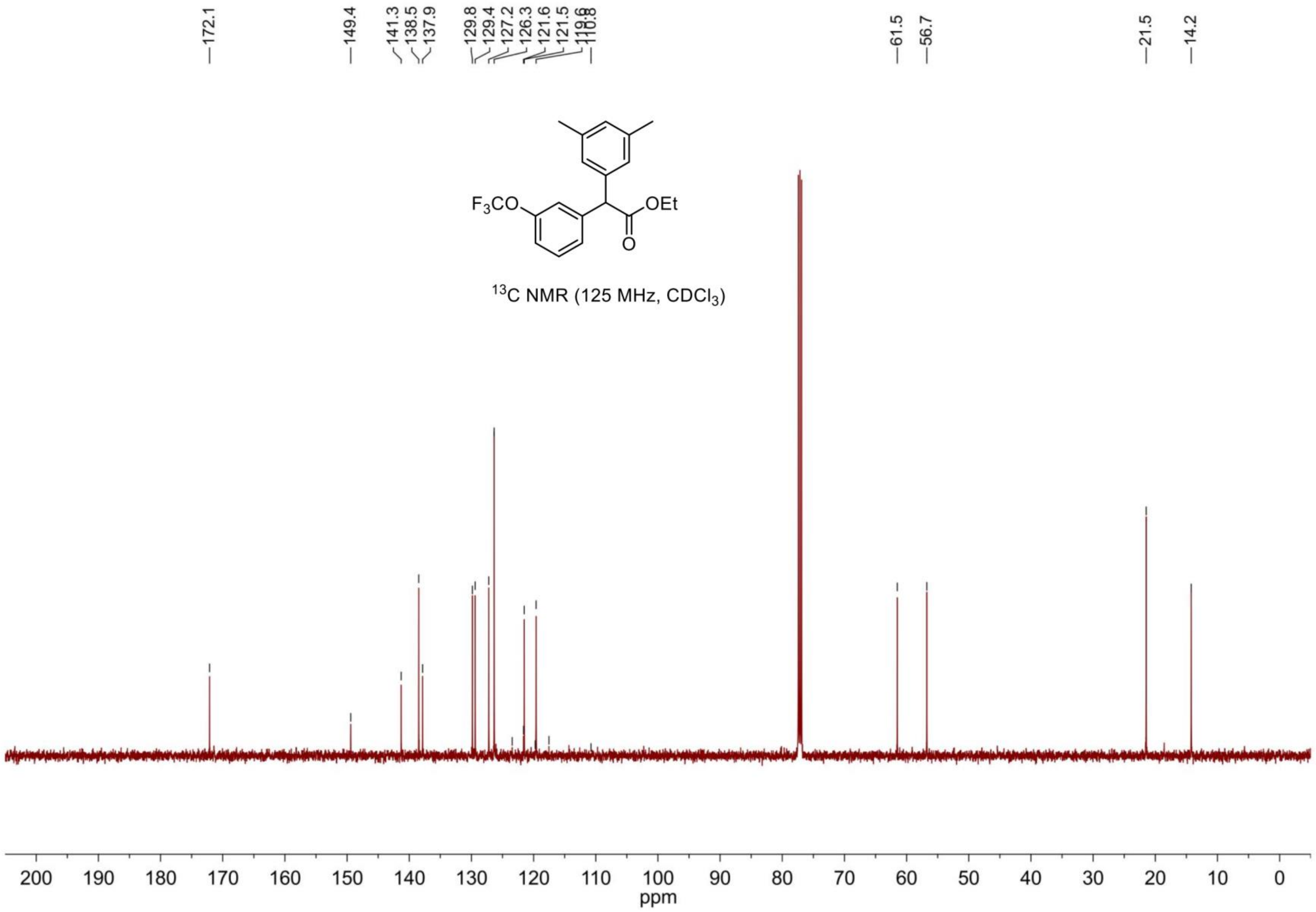

Go back to table of contents

1,2-Bis(4-methoxyphenyl)-2-phenylethan-1-one (53)

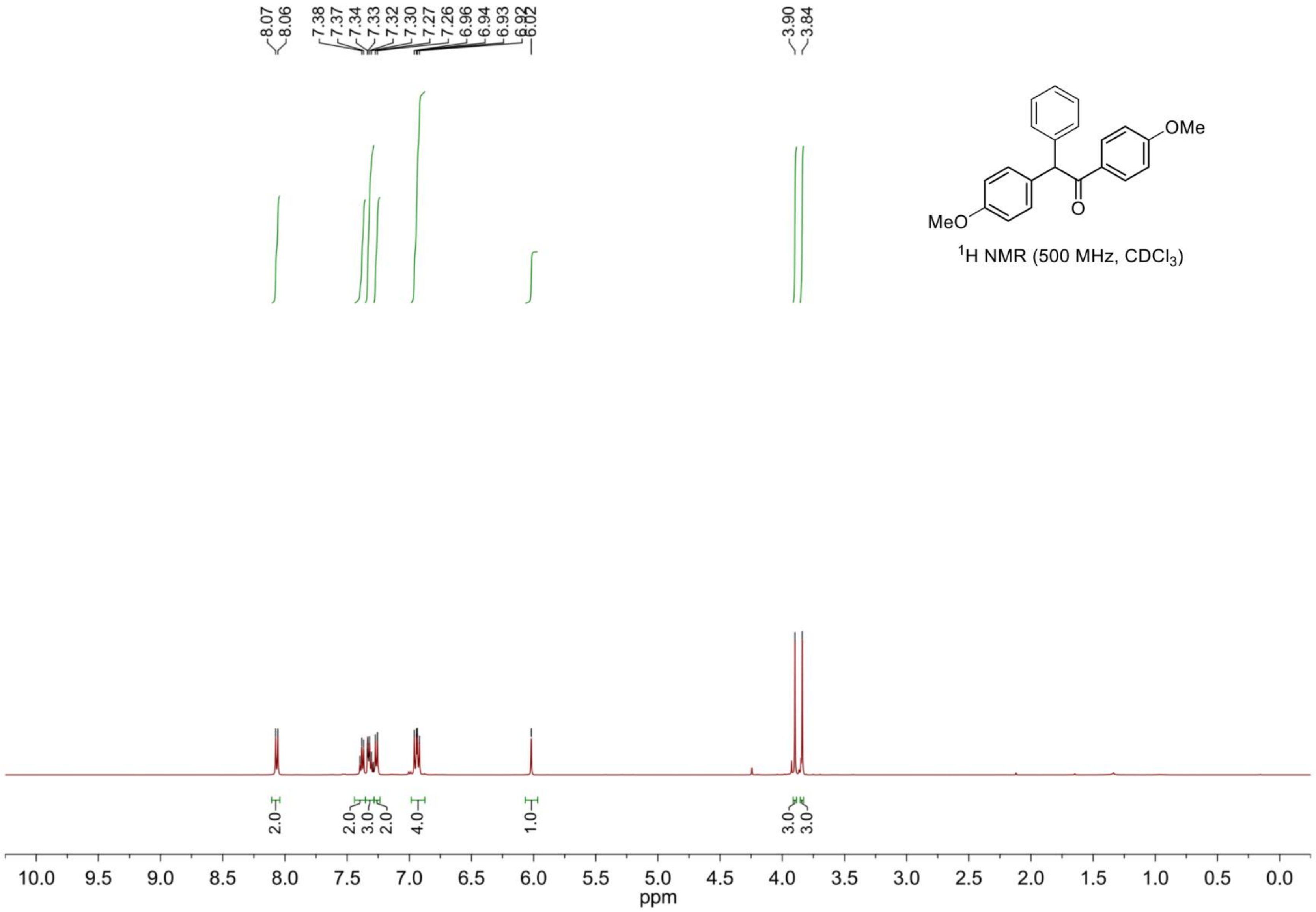

Go back to table of contents

1,2-Bis(4-methoxyphenyl)-2-phenylethan-1-one (53)

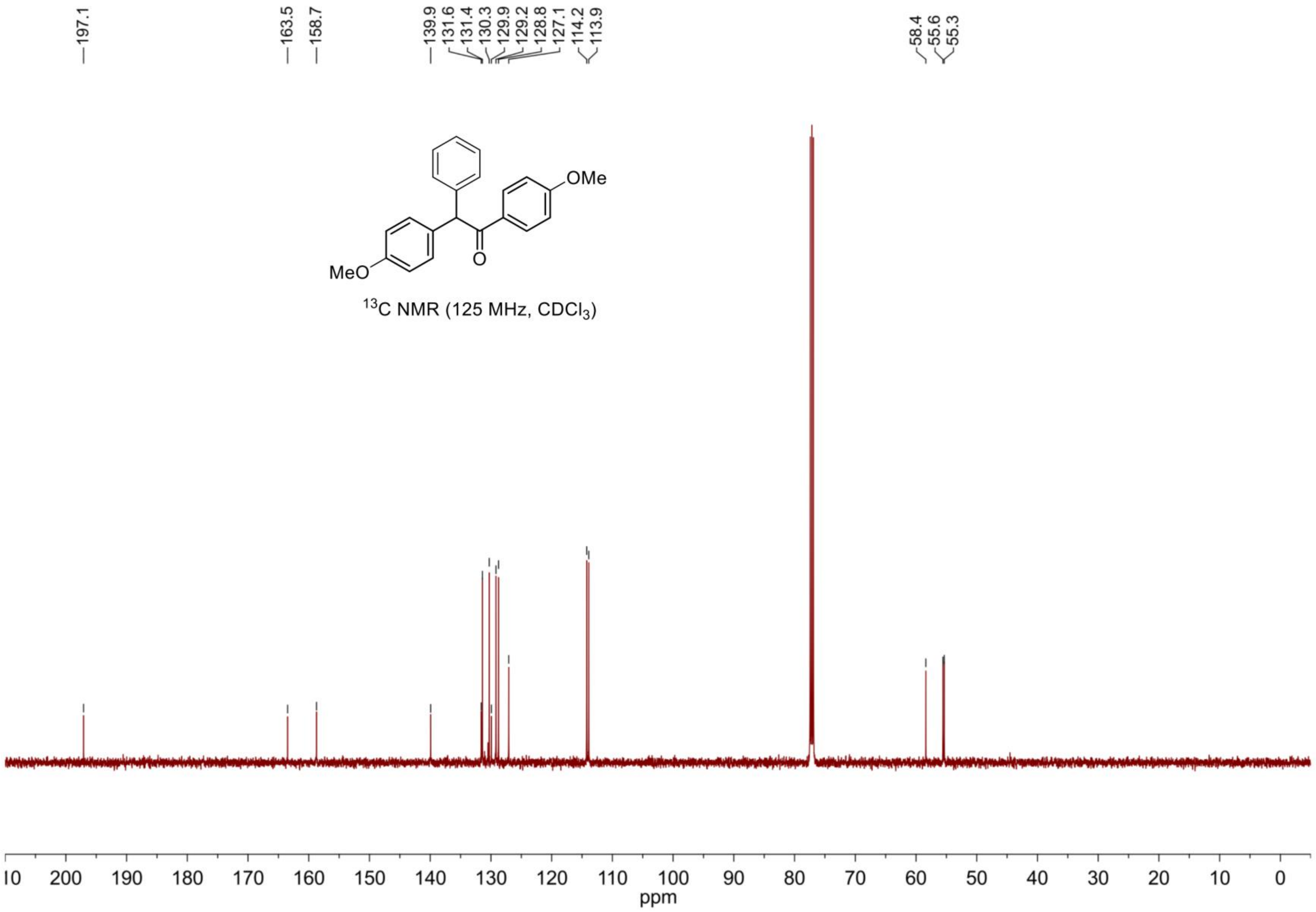

Go back to table of contents

Ethyl 7-chloro-2-phenylheptanoate (54)

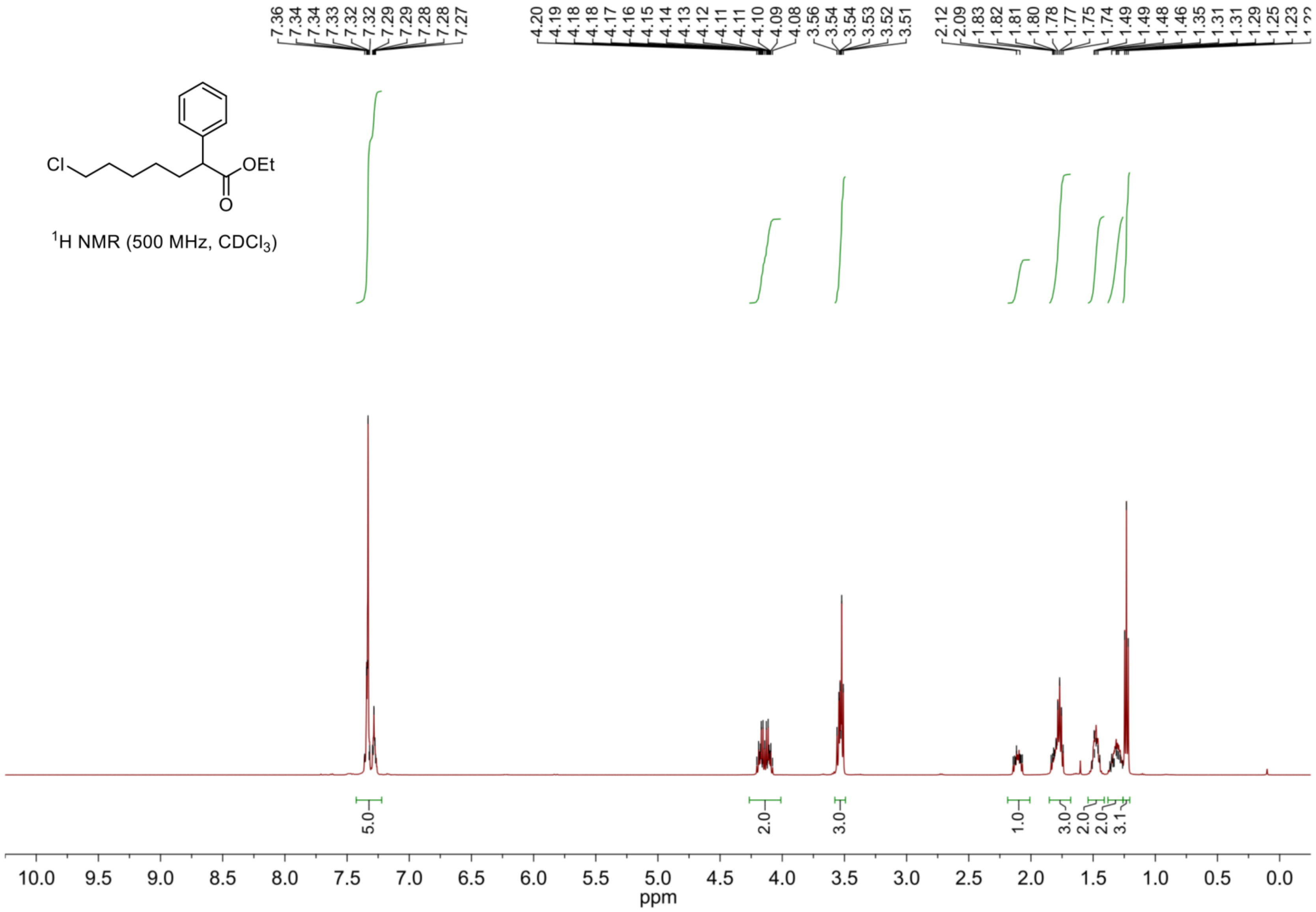

Go back to table of contents

Ethyl 7-chloro-2-phenylheptanoate (54)

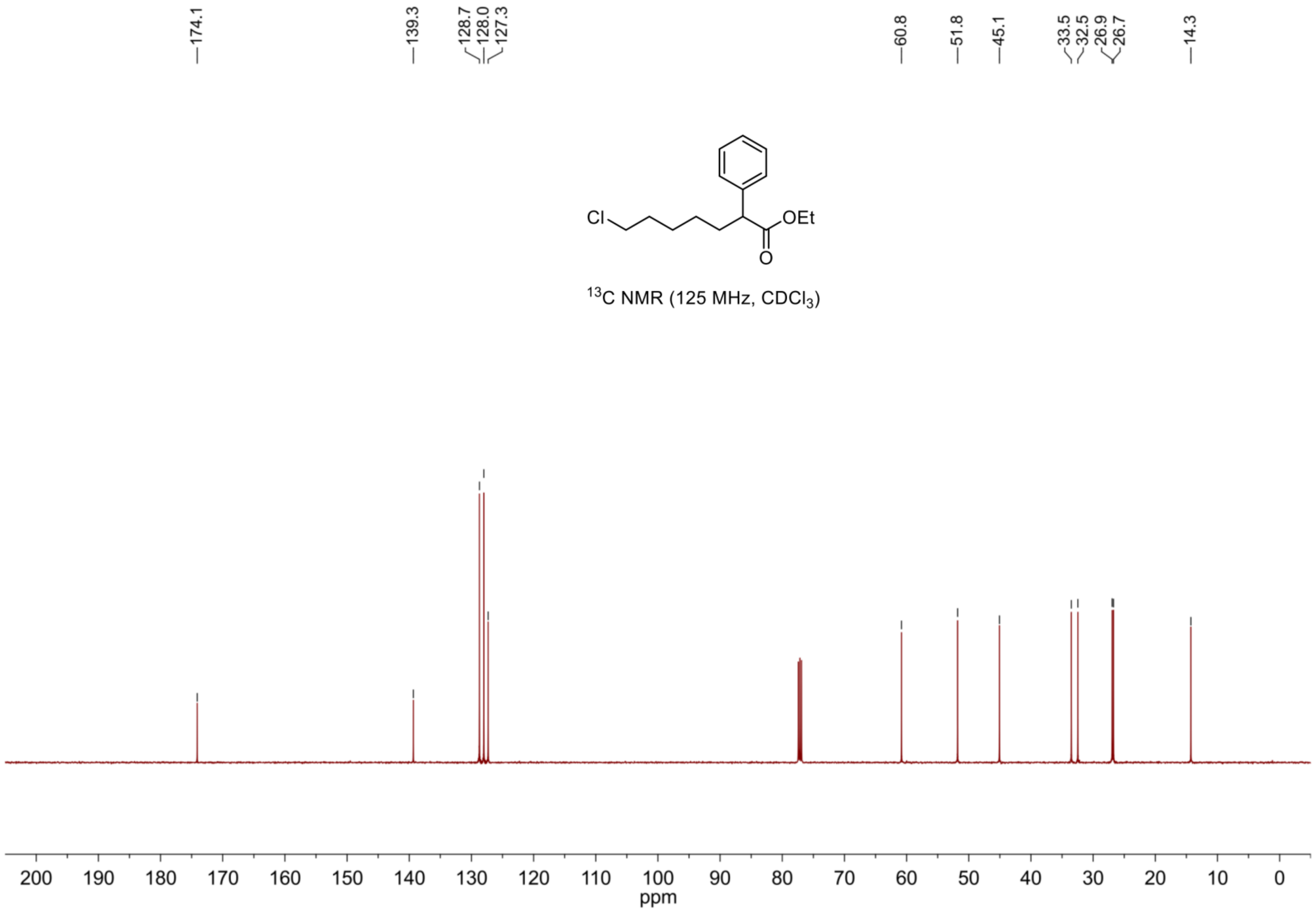

Go back to table of contents

Ehyl 2,4-diphenylbutanoate (55)

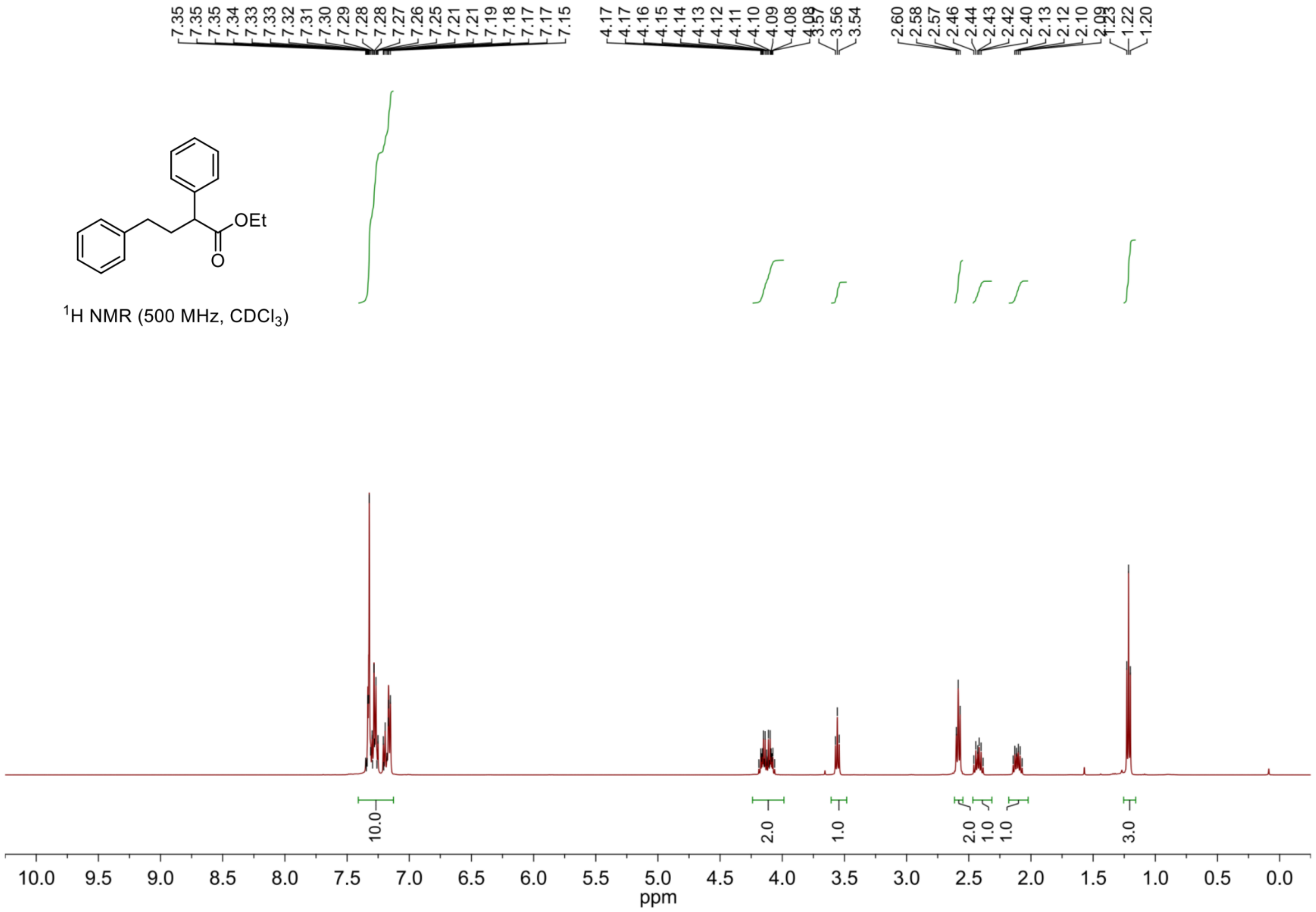

Go back to table of contents

Ehyl 2,4-diphenylbutanoate (55)

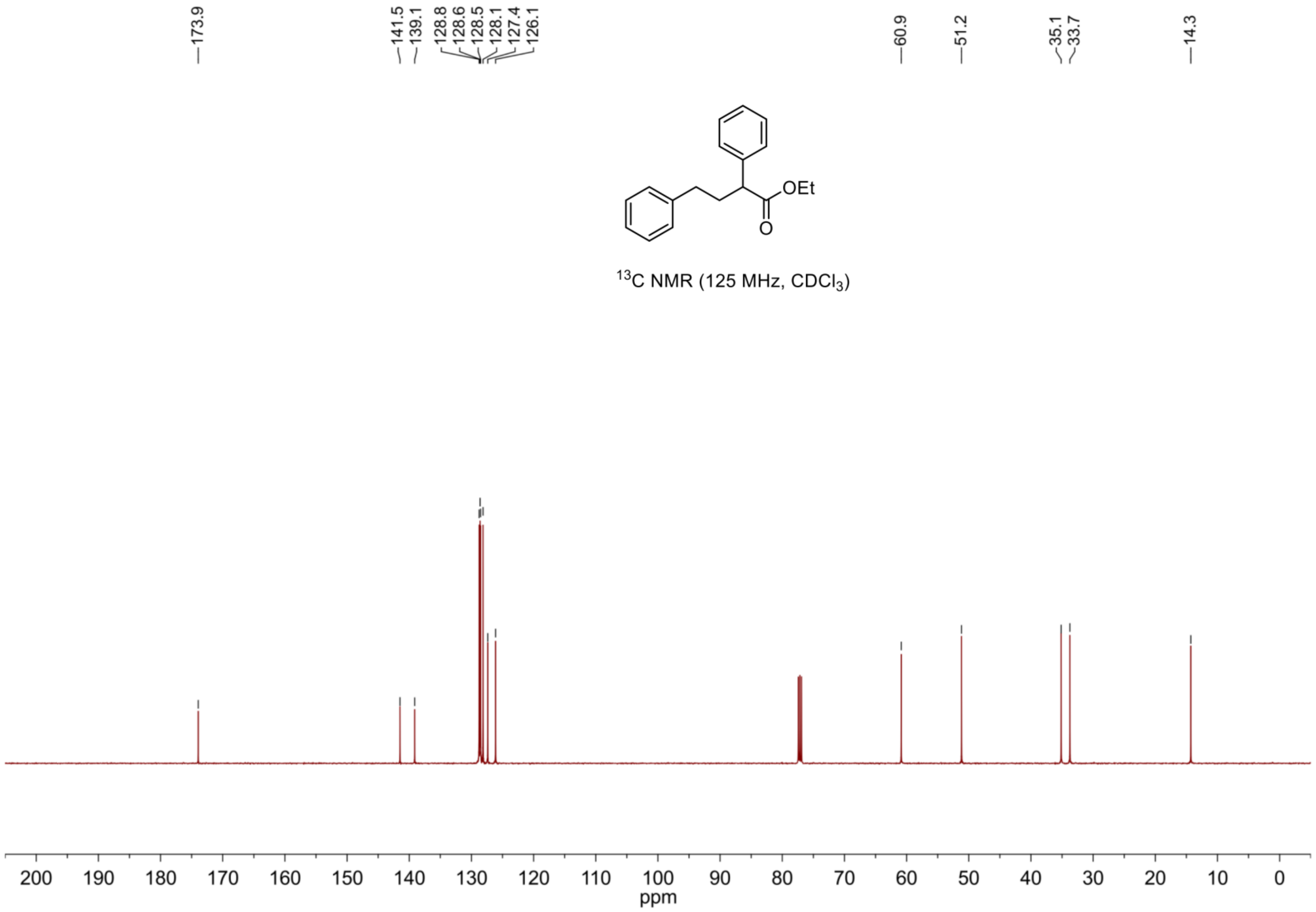

Go back to table of contents

Ethyl 2-([1,1'-biphenyl]-4-yl)-4-(4-(*tert*-butoxy)phenyl)butanoate (58)

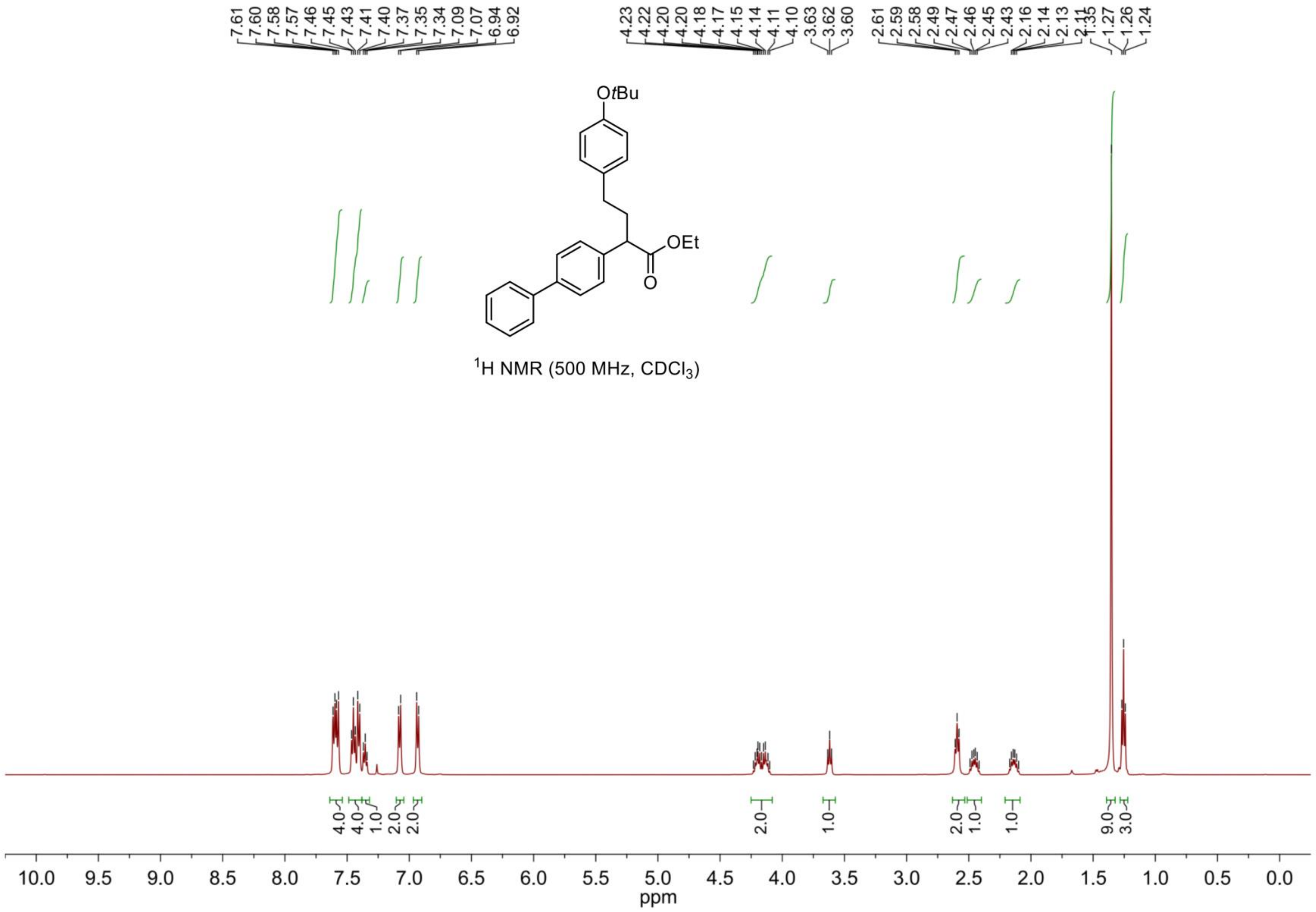

Go back to table of contents

Ethyl 2-([1,1'-biphenyl]-4-yl)-4-(4-(*tert*-butoxy)phenyl)butanoate (58)

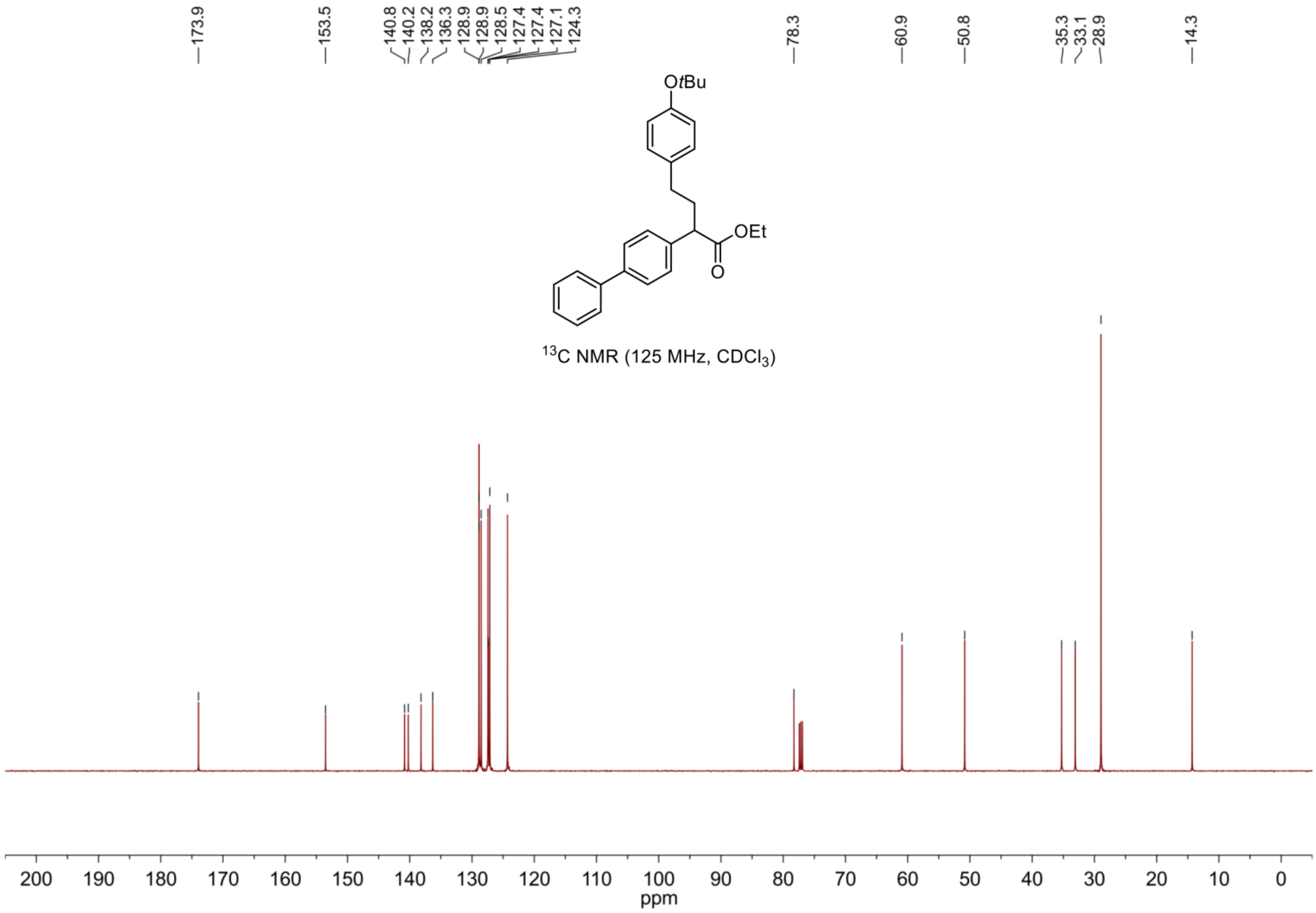

Go back to table of contents

Ethyl 2-(4-isobutylphenyl)-4-(4-methoxyphenyl)butanoate (59)

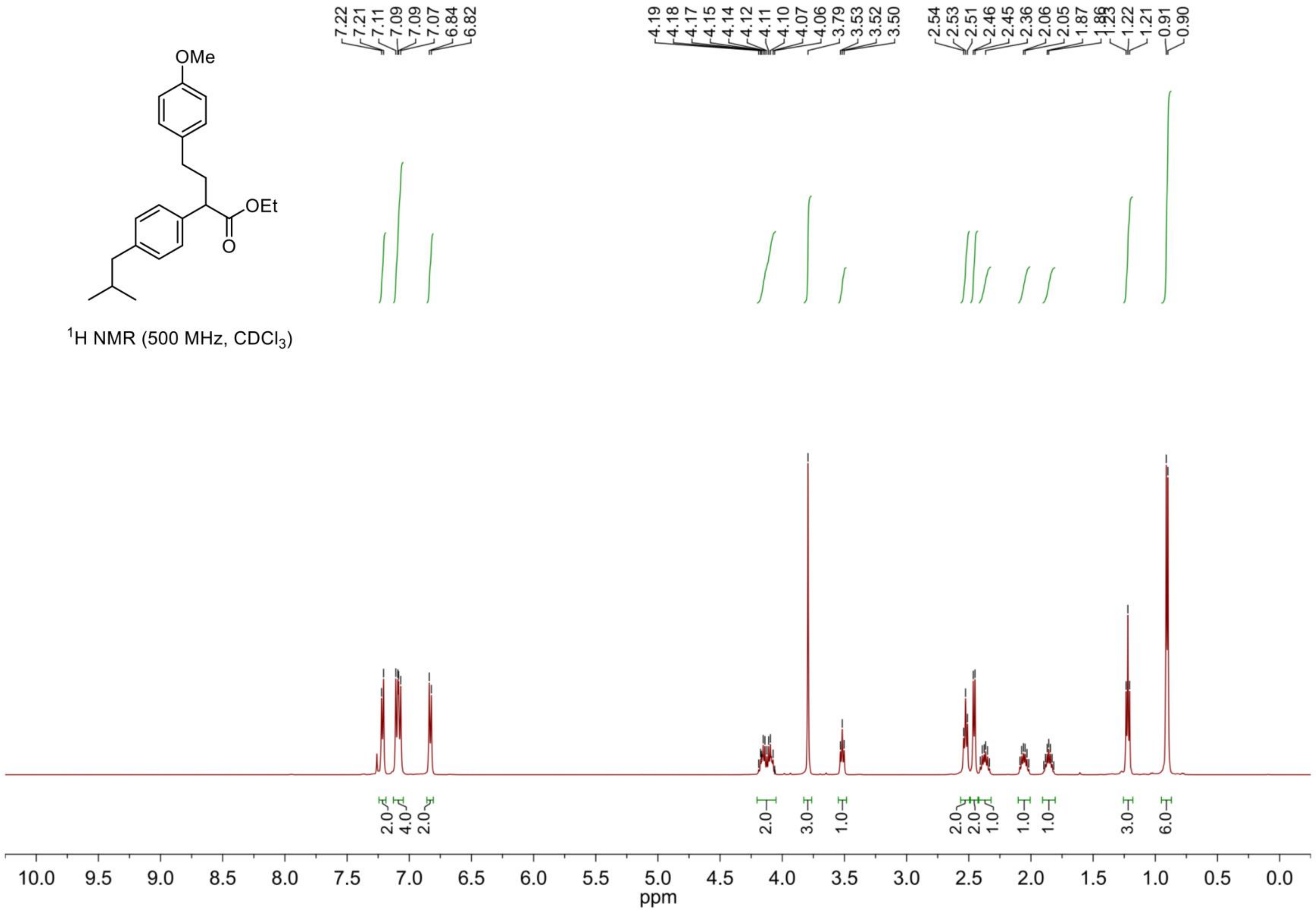

Ethyl 2-(4-isobutylphenyl)-4-(4-methoxyphenyl)butanoate (59)

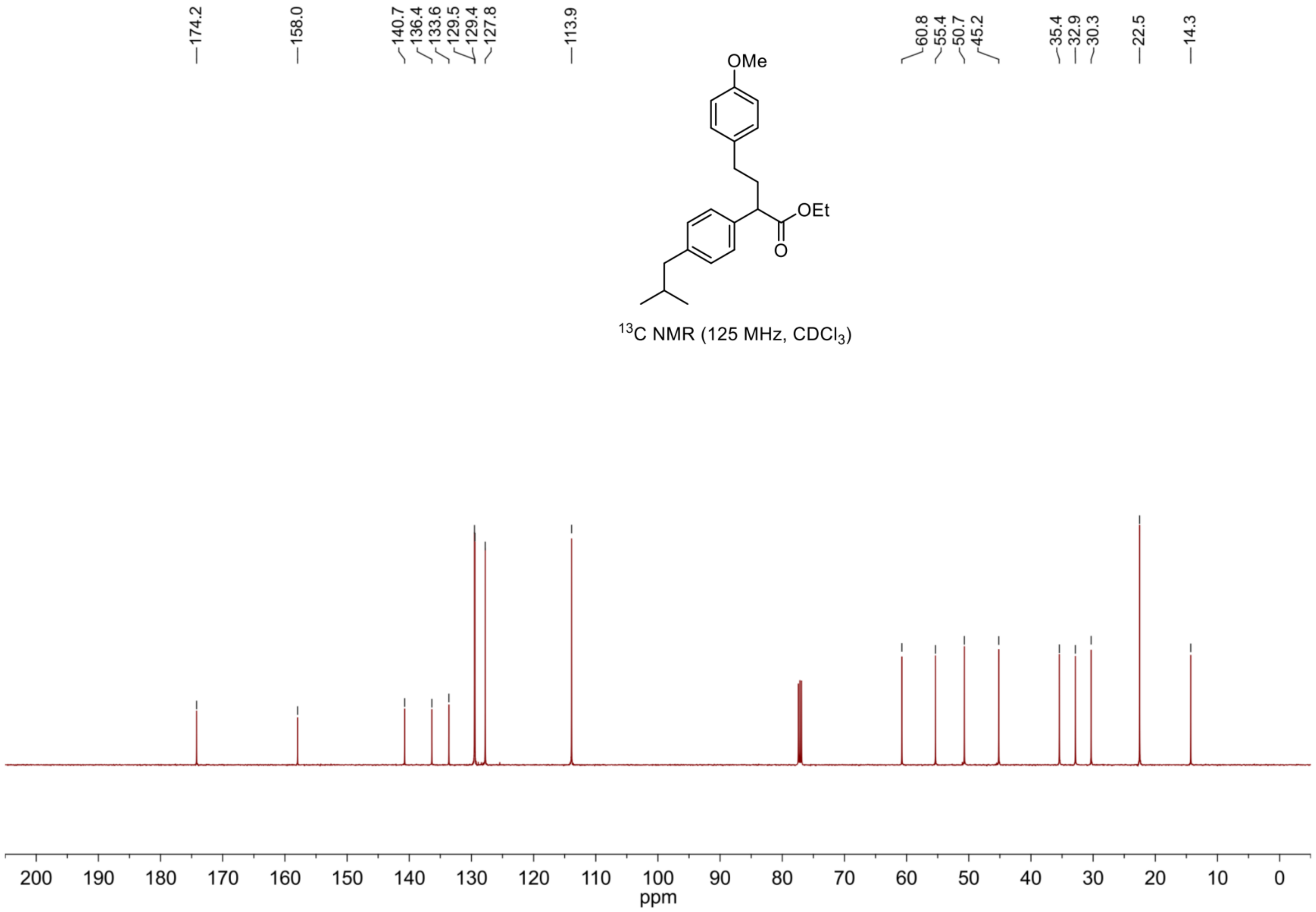

Go back to table of contents

Ethyl 4-(4-isobutylphenyl)-2-(4-methoxyphenyl)butanoate (60)

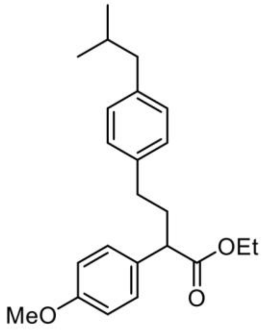

^{1}H NMR (500 MHz, CDCl$_3$)

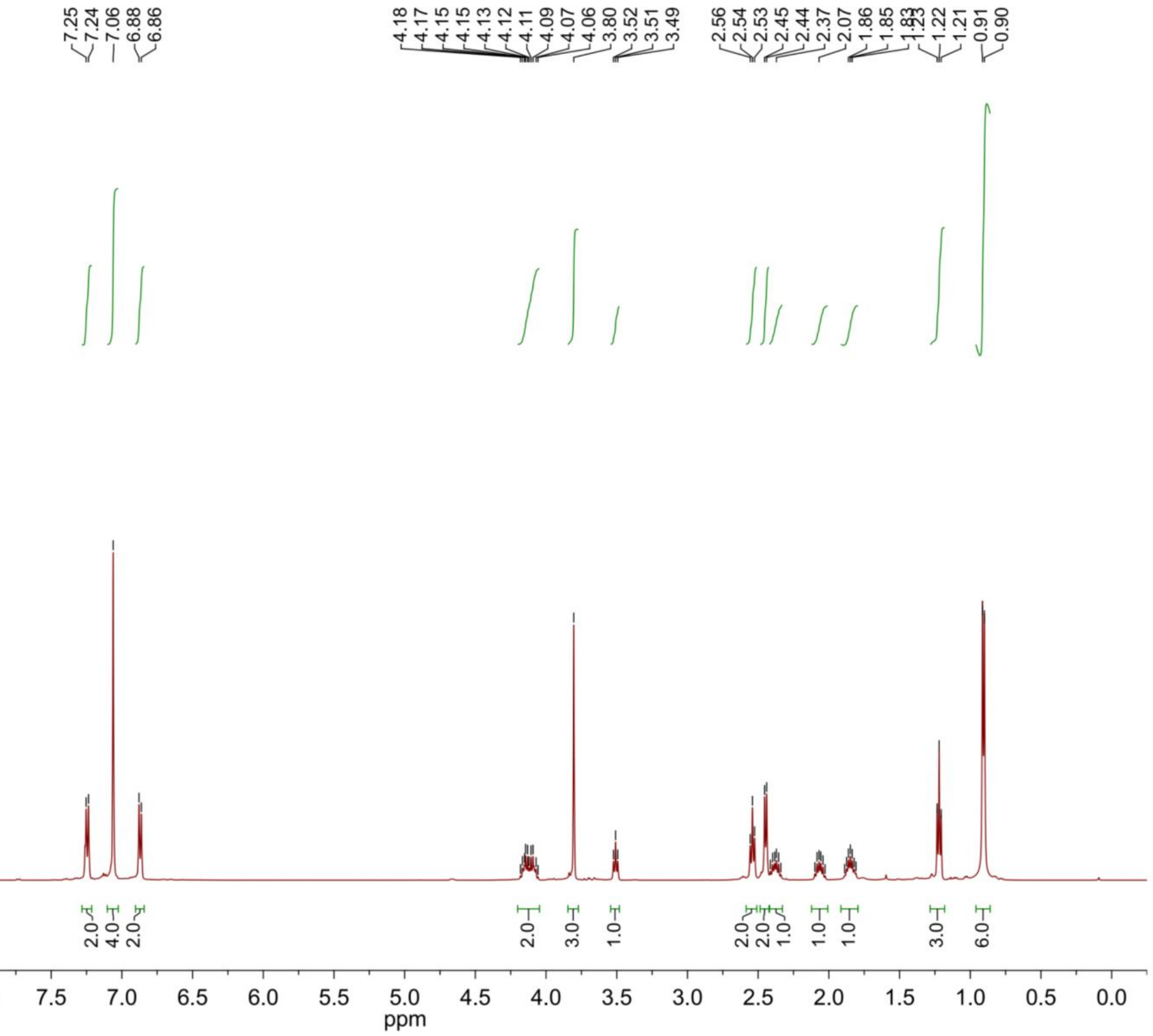

Ethyl 4-(4-isobutylphenyl)-2-(4-methoxyphenyl)butanoate (60)

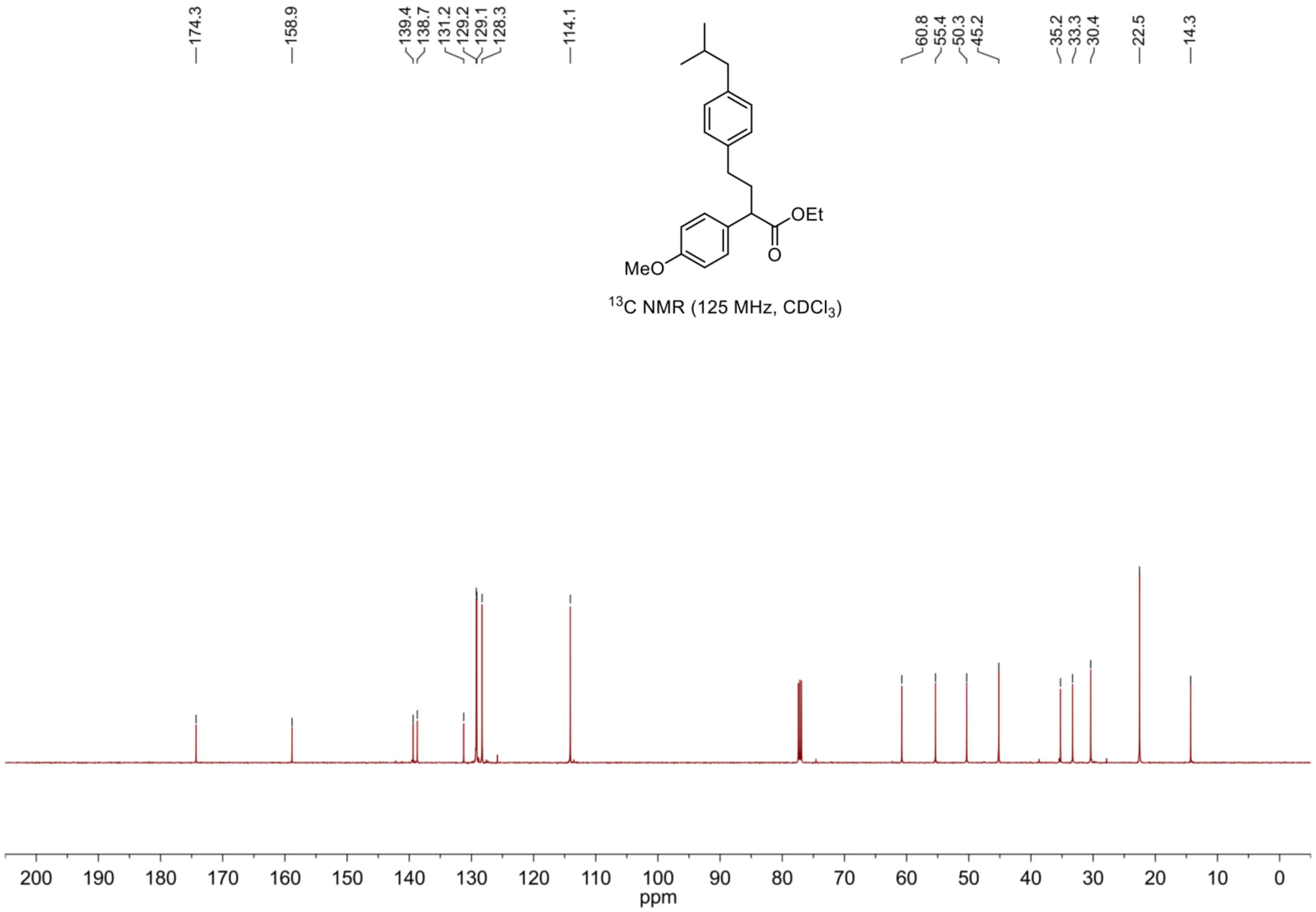

Go back to table of contents

Ethyl 4-(4-(*tert*-butyl)phenyl)-2-(9,9-dimethyl-9*H*-xanthen-2-yl)butanoate (61)

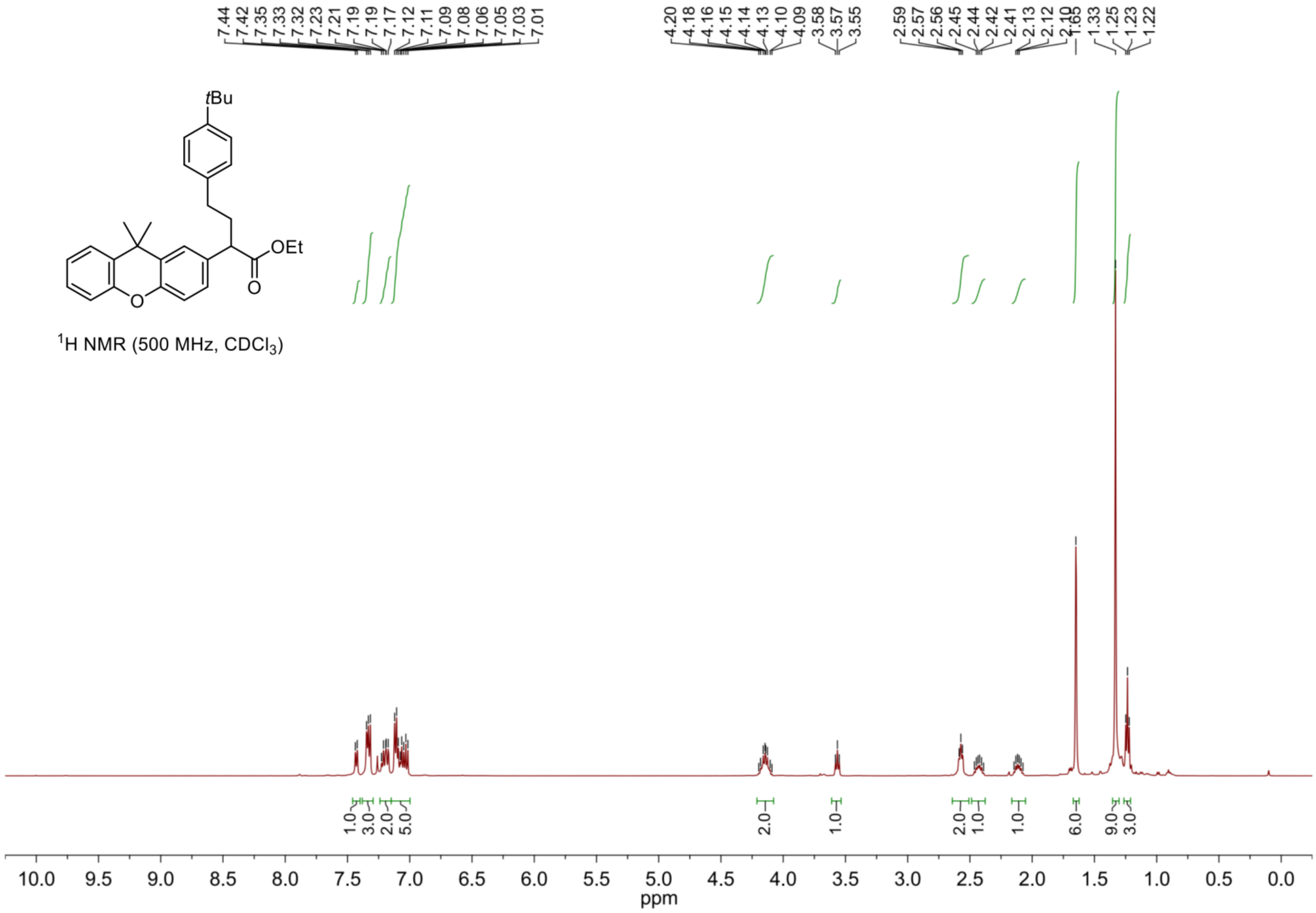

Go back to table of contents

Ethyl 4-(4-(*tert*-butyl)phenyl)-2-(9,9-dimethyl-9*H*-xanthen-2-yl)butanoate (61)

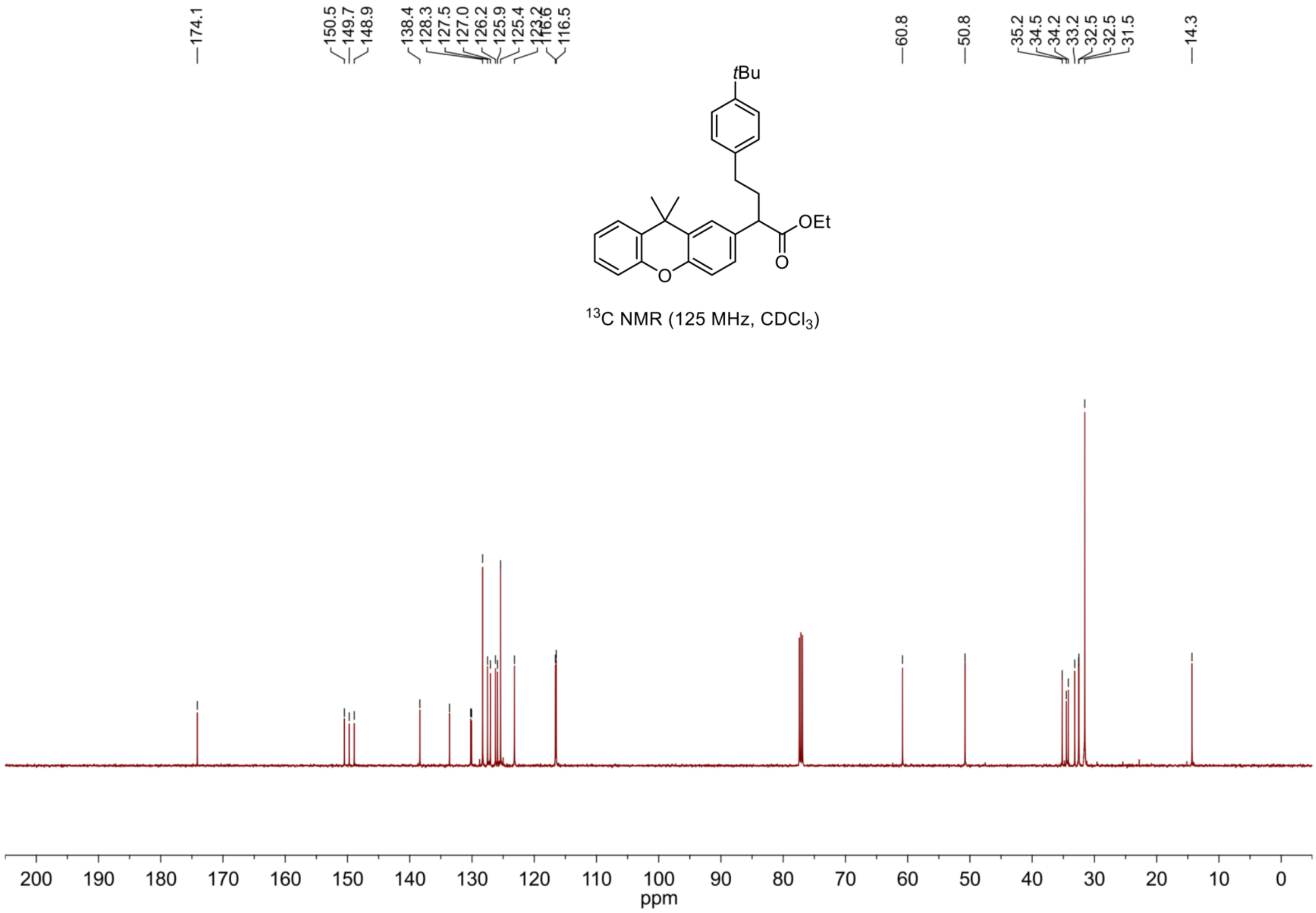

Go back to table of contents

Ethyl 2-(3-fluoro-4-methoxyphenyl)-4-(*p*-tolyl)butanoate (62)

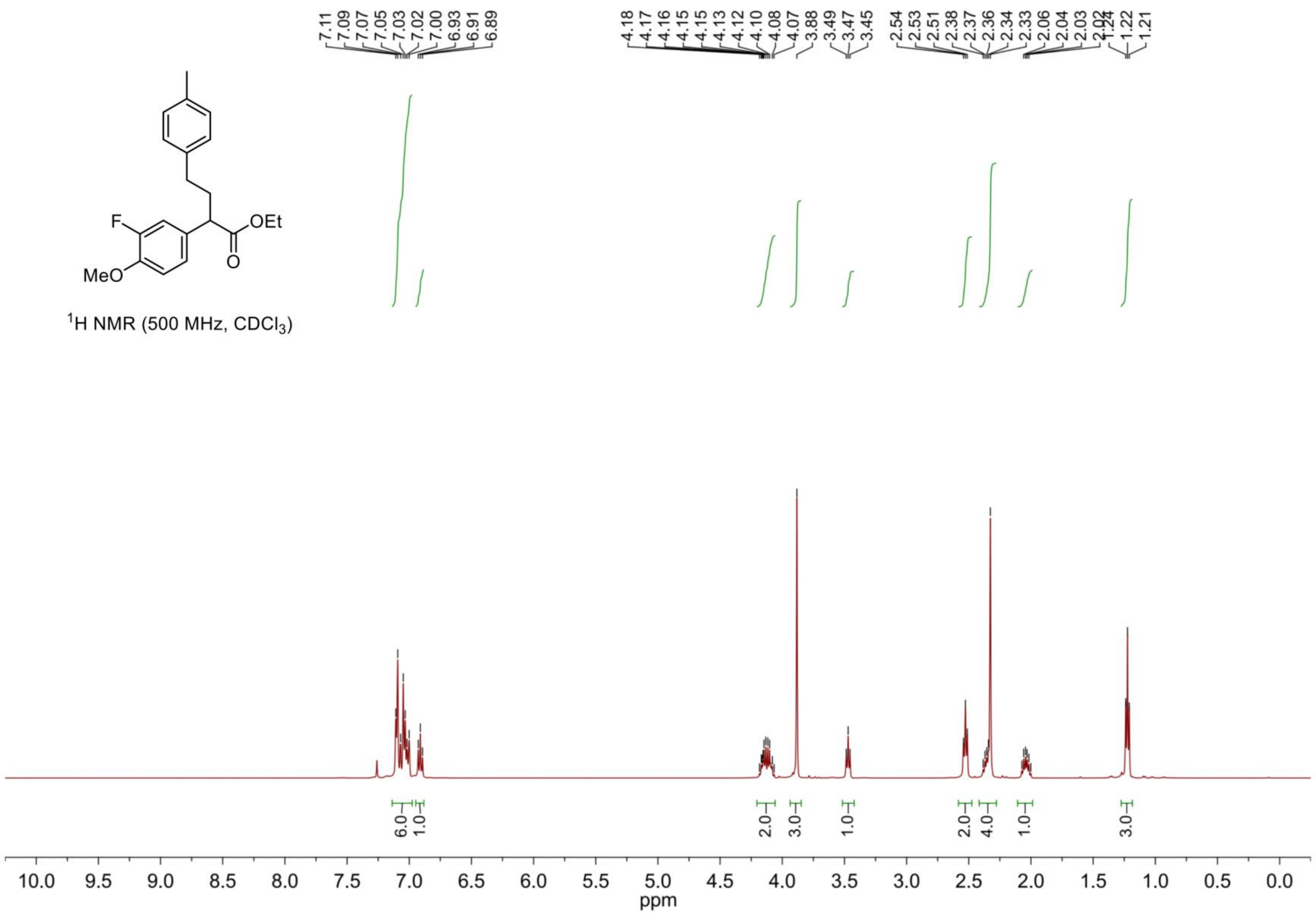

Go back to table of contents

Ethyl 2-(3-fluoro-4-methoxyphenyl)-4-(*p*-tolyl)butanoate (62)

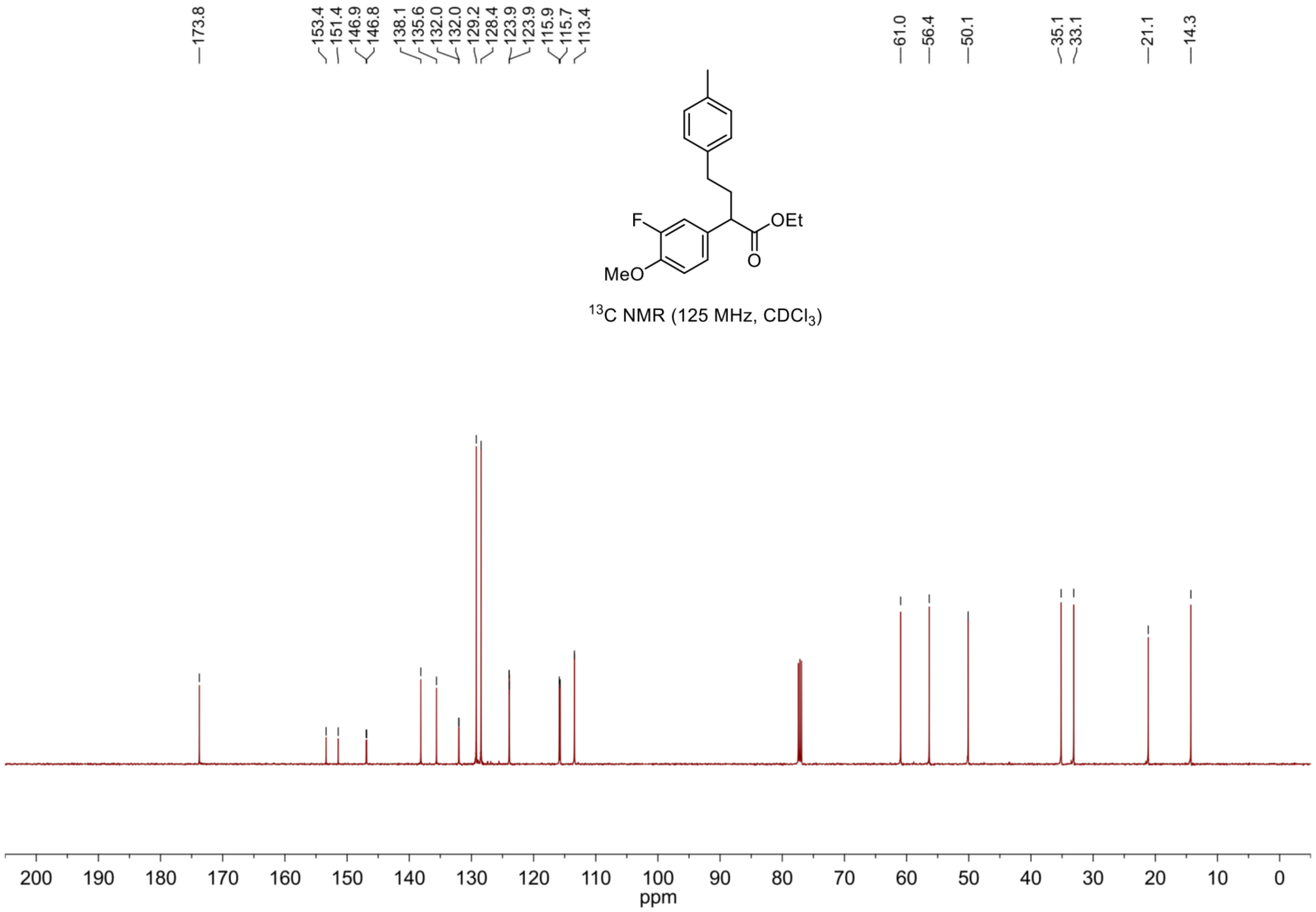

Go back to table of contents

5-(3,4-Dimethoxyphenyl)-2-phenylpentanoic acid (63)

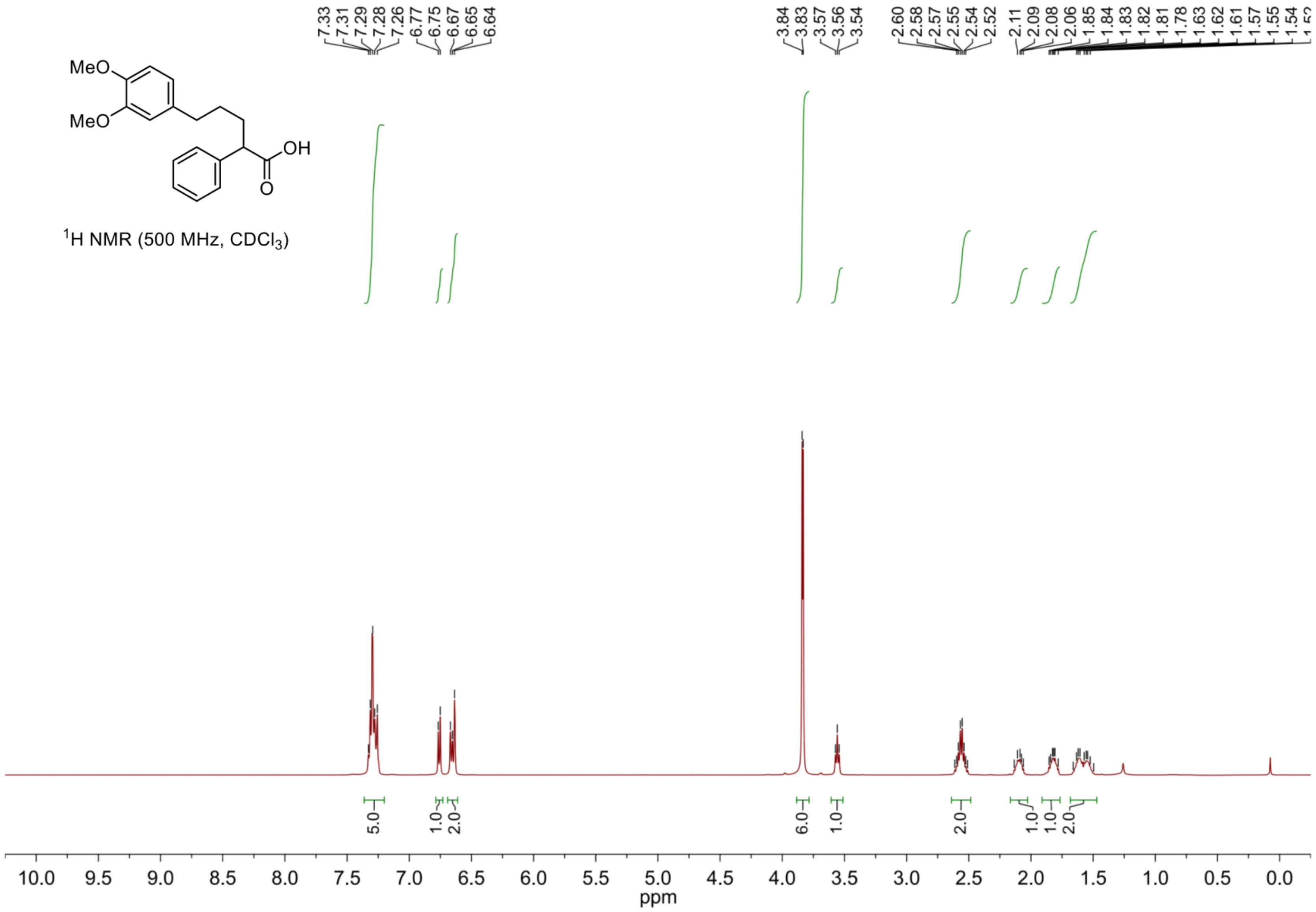

Go back to table of contents

5-(3,4-Dimethoxyphenyl)-2-phenylpentanoic acid (63)

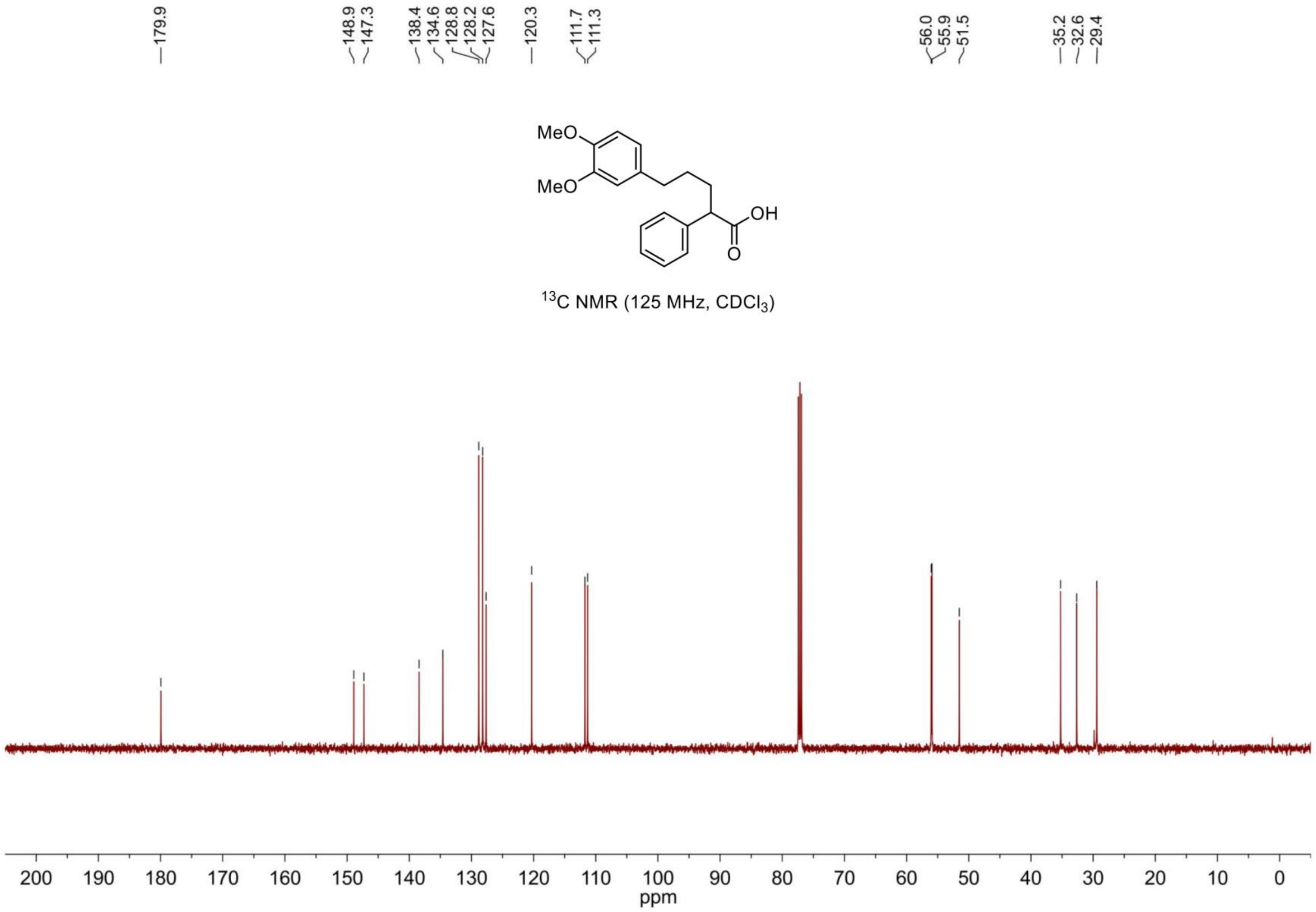

Go back to table of contents

Ethyl 2-(4-isobutylphenyl)heptanoate (64)

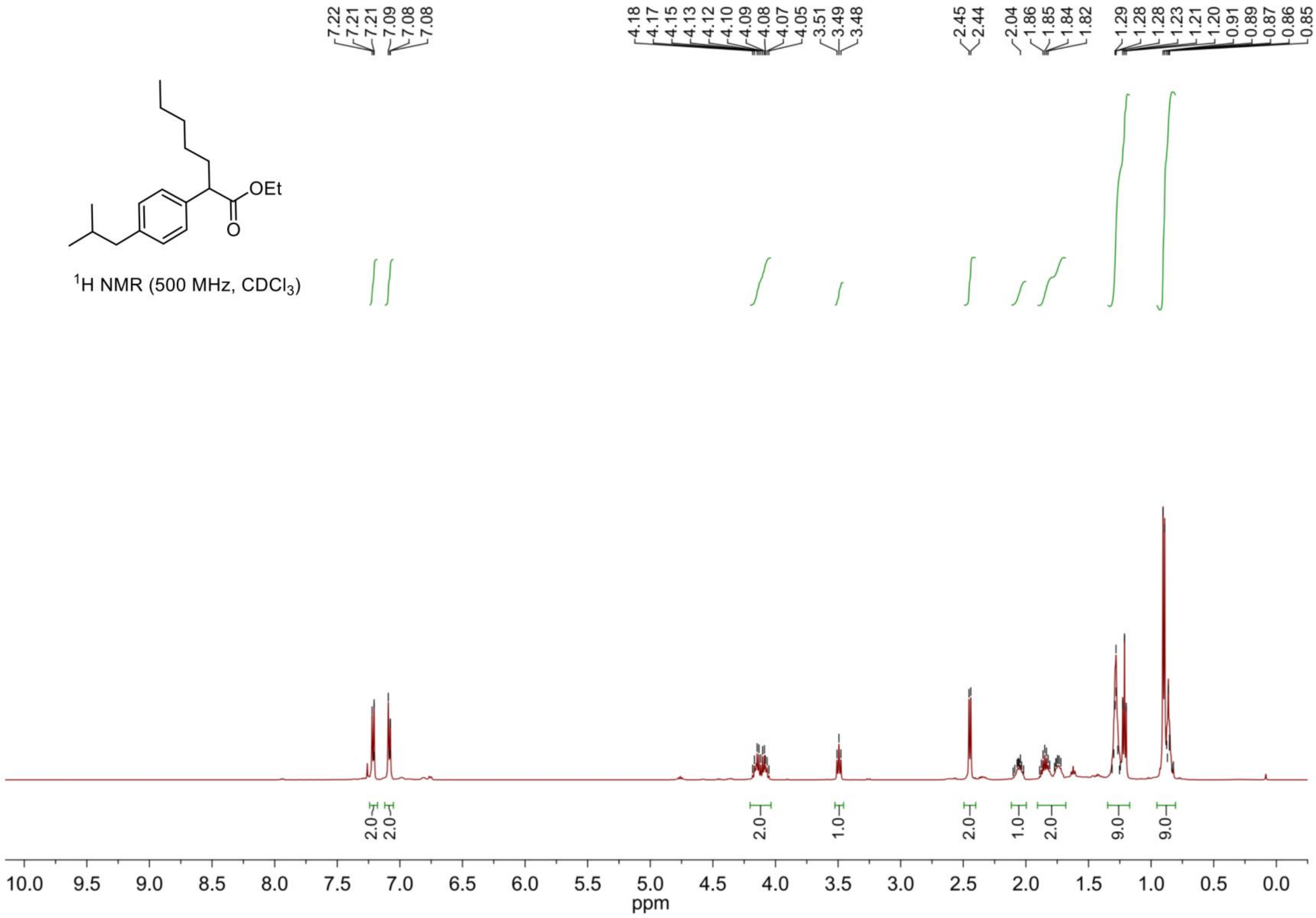

Ethyl 2-(4-isobutylphenyl)heptanoate (64)

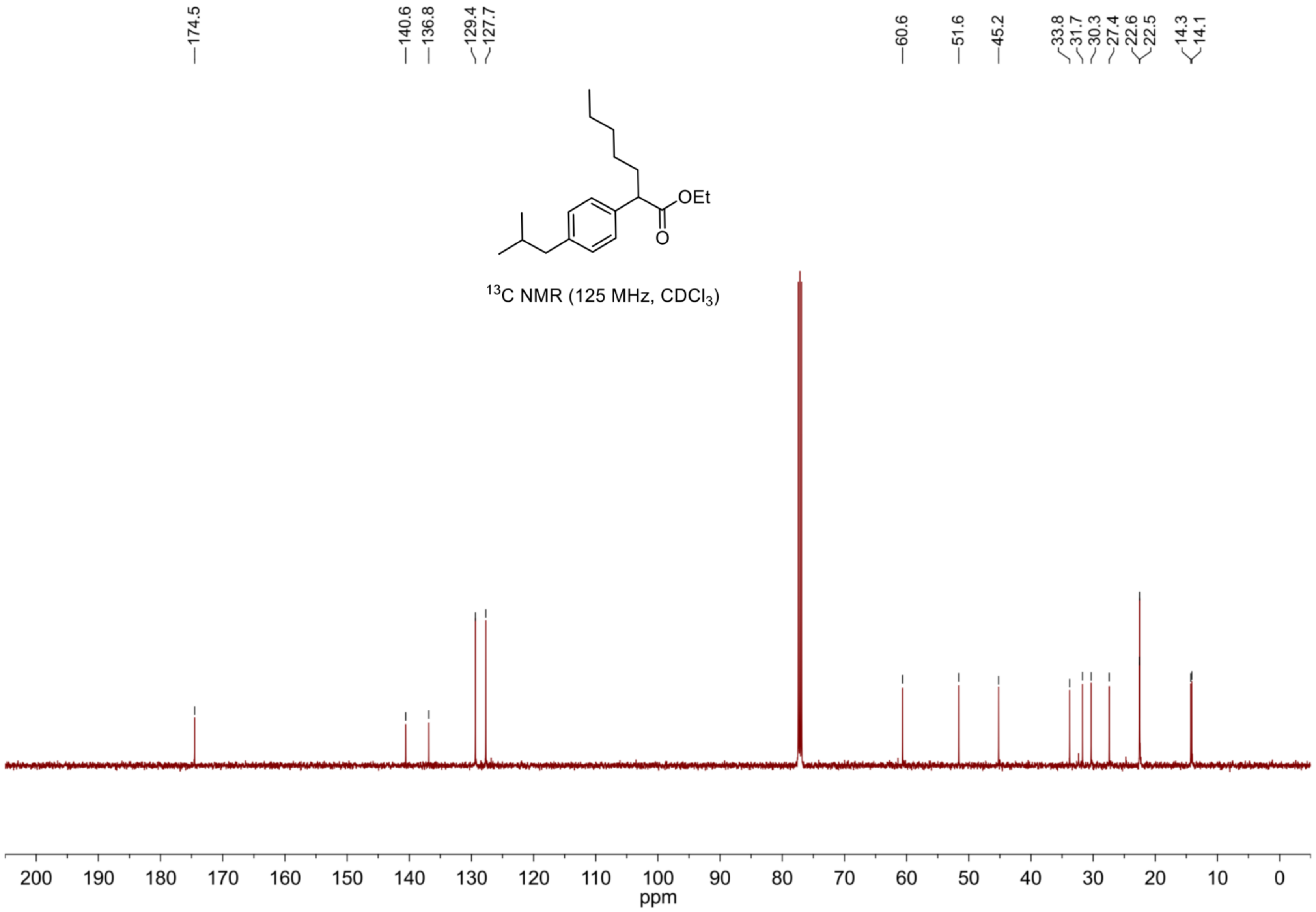

Go back to table of contents

Ethyl 2-(3-fluoro-4-methoxyphenyl)octanoate (65)

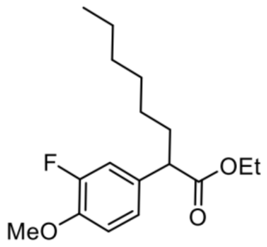

^{1}H NMR (500 MHz, $CDCl_3$)

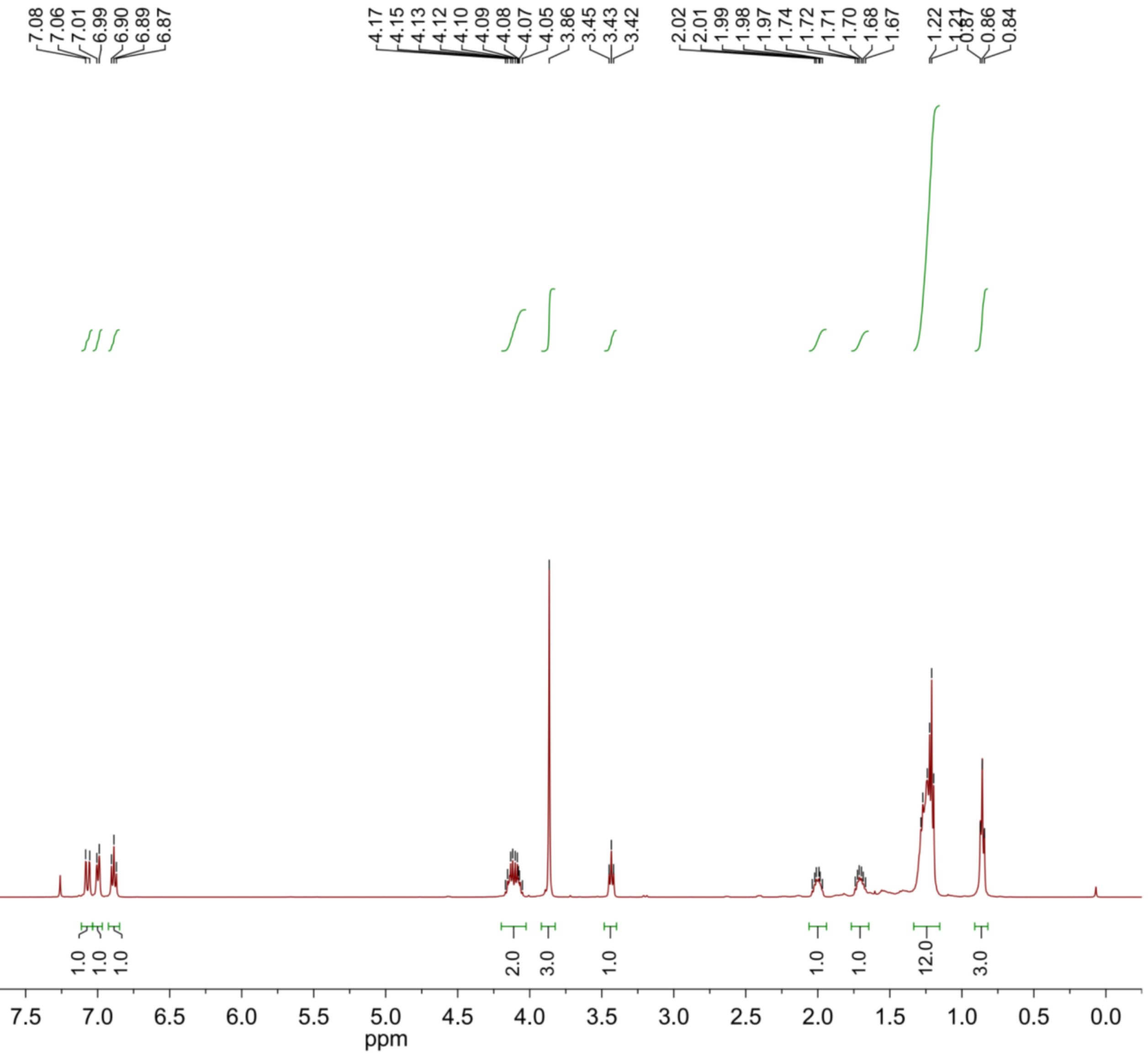

Go back to table of contents

Ethyl 2-(3-fluoro-4-methoxyphenyl)octanoate (65)

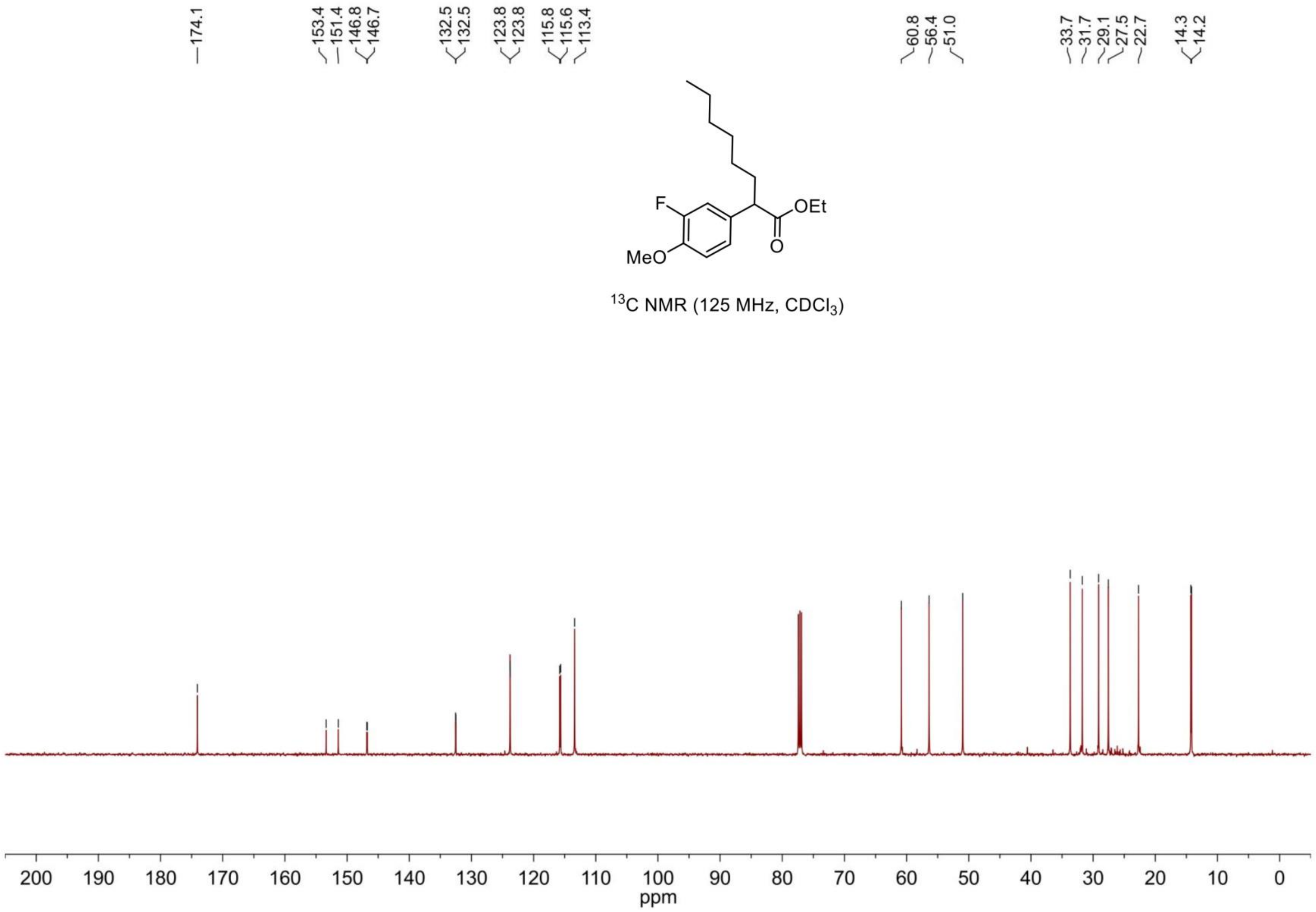

Go back to table of contents

Ethyl 2-(9,9-dimethyl-9*H*-xanthen-2-yl)-2-phenylacetate (66)

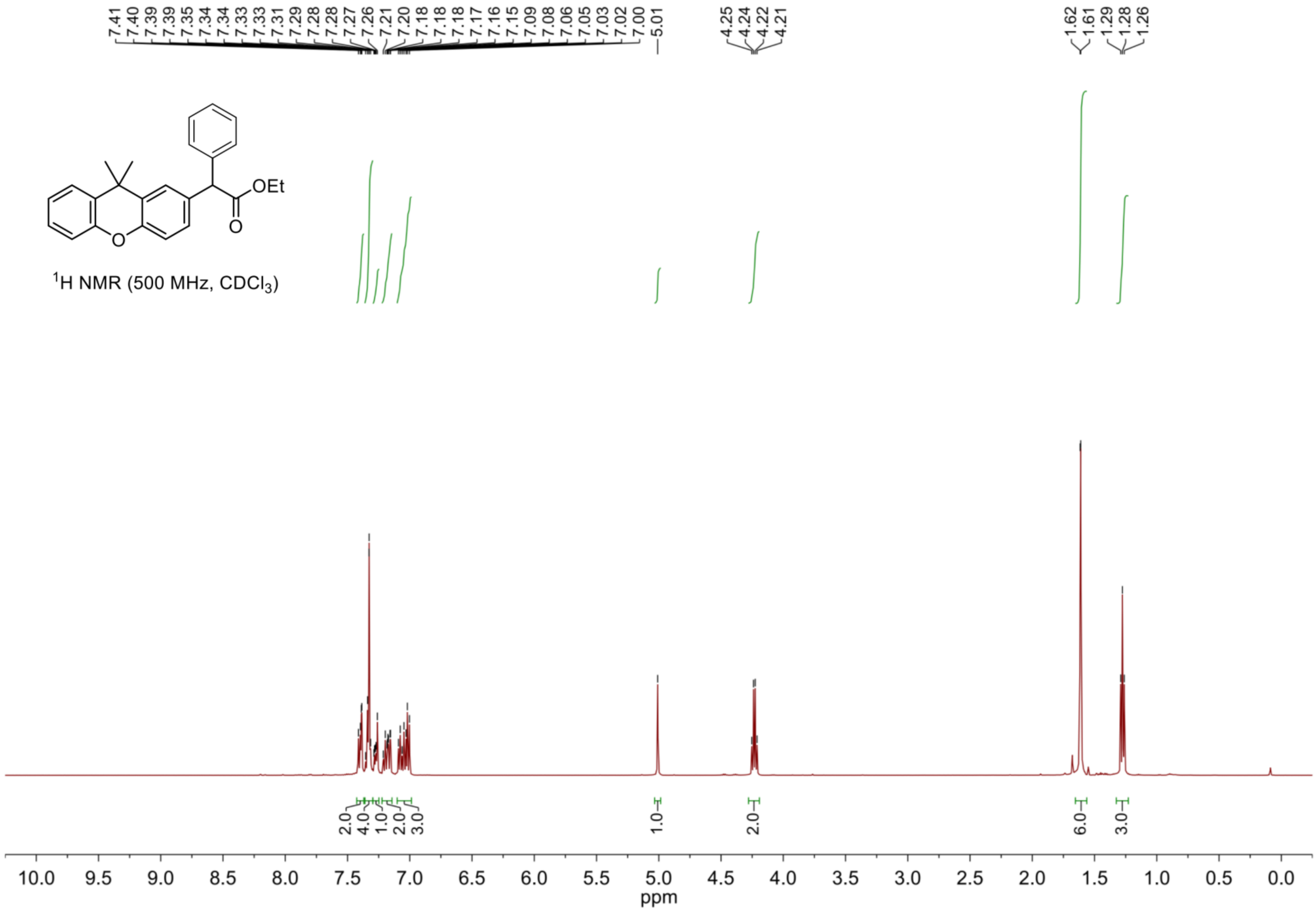

Go back to table of contents

Ethyl 2-(9,9-dimethyl-9*H*-xanthen-2-yl)-2-phenylacetate (66)

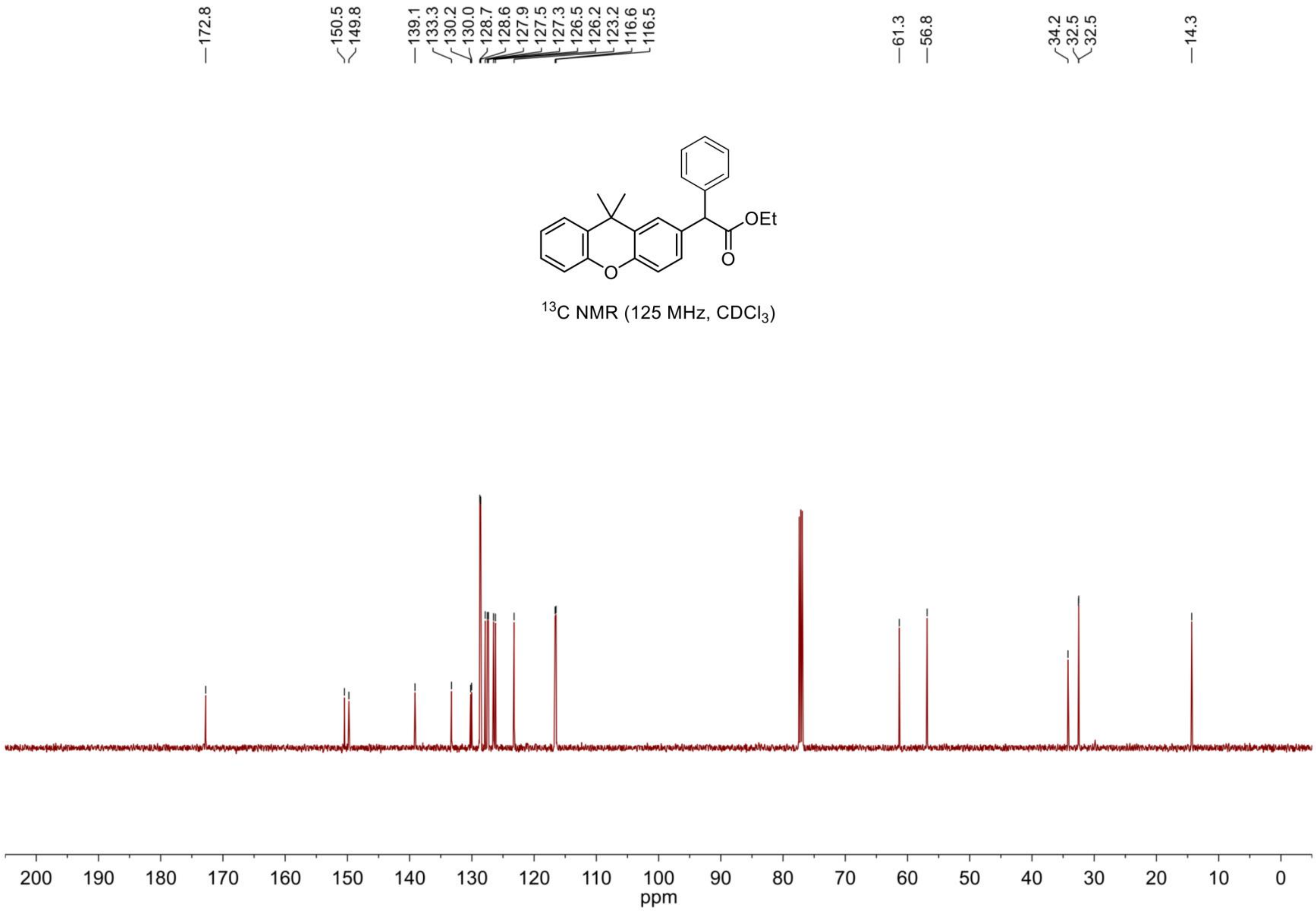

Go back to table of contents

Ethyl -2-([1,1'-biphenyl]-4-yl)-2-(3-(trifluoromethoxy)phenyl)acetate (67)

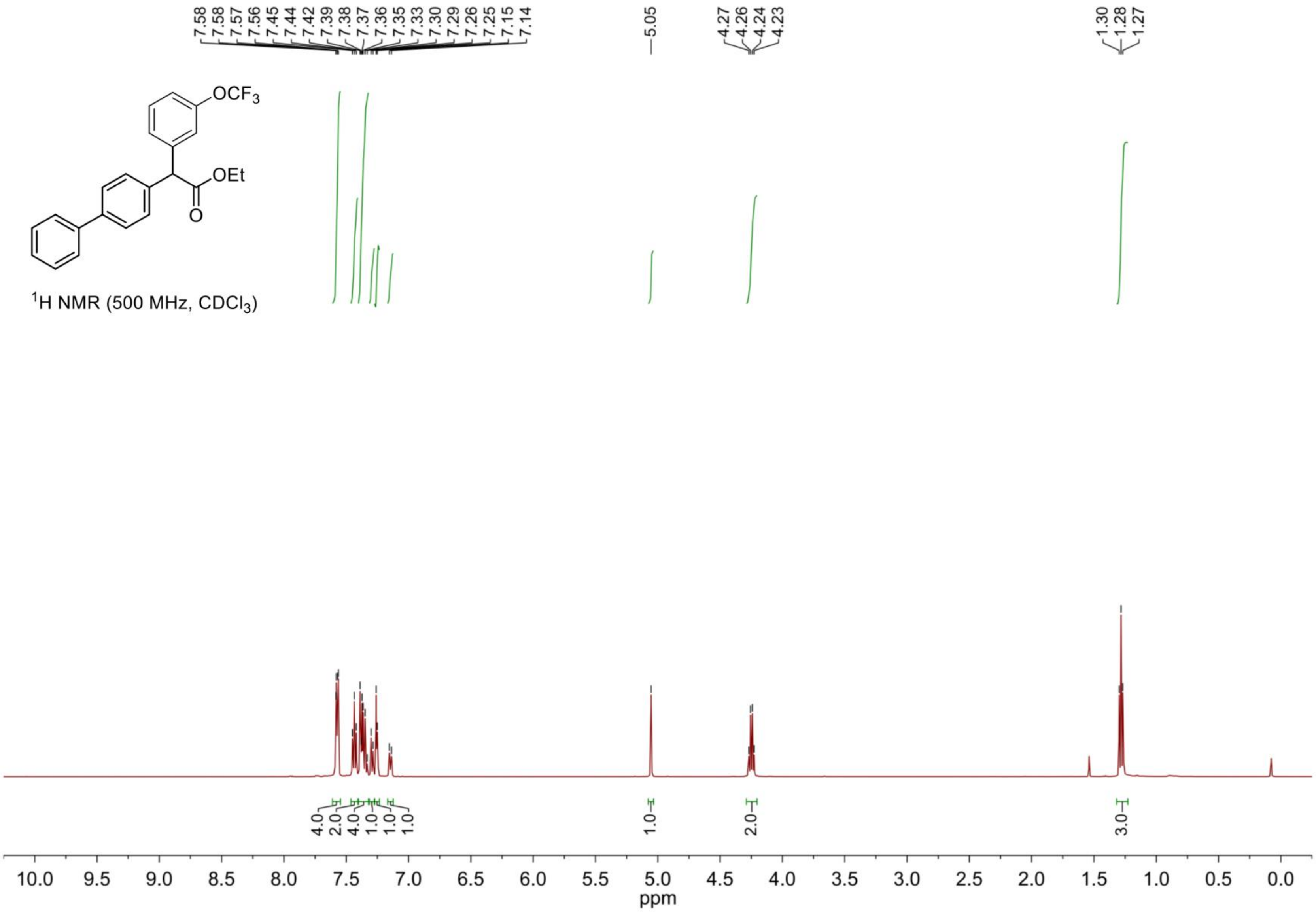

Go back to table of contents

Ethyl -2-([1,1'-biphenyl]-4-yl)-2-(3-(trifluoromethoxy)phenyl)acetate (67)

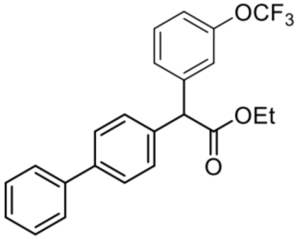

^{13}C NMR (125 MHz, $CDCl_3$)

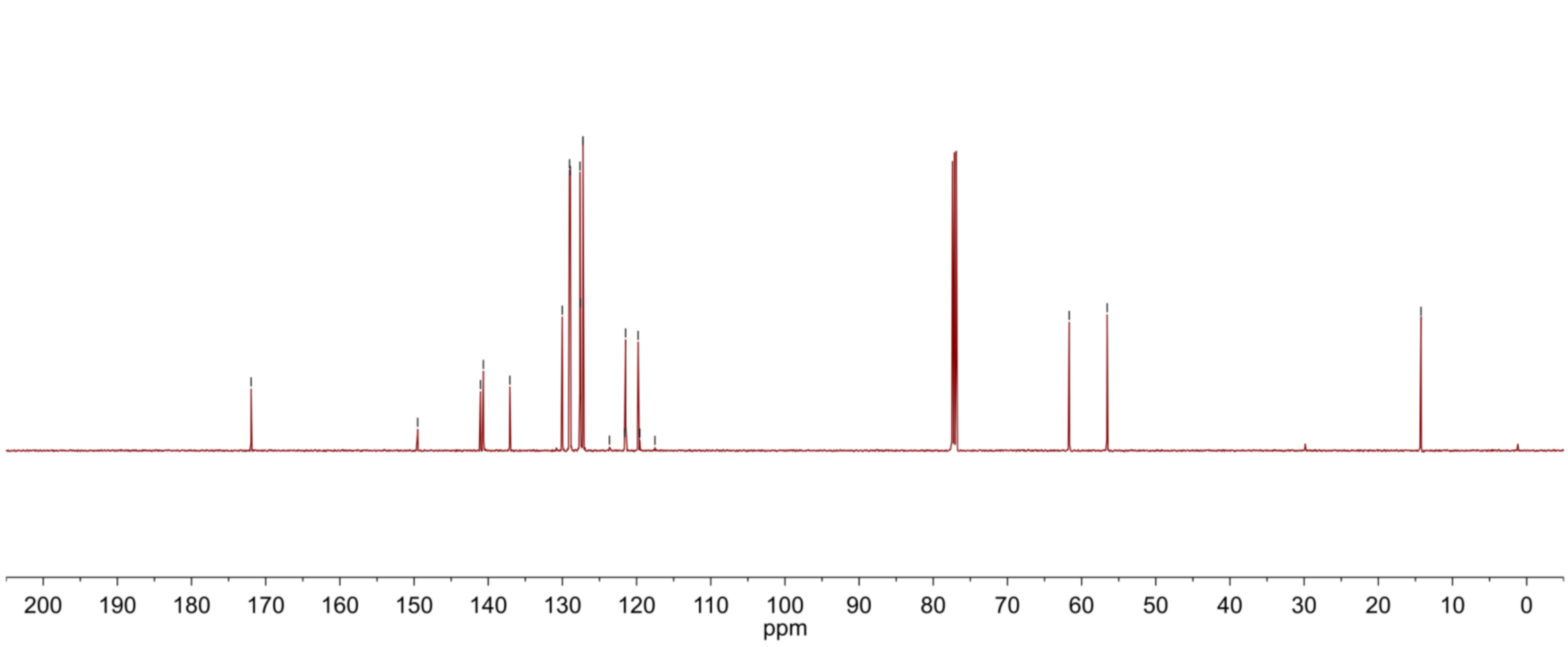

Go back to table of contents

Ethyl 2-(2-fluoro-[1,1'-biphenyl]-4-yl)-3-methylbut-3-enoate (68)

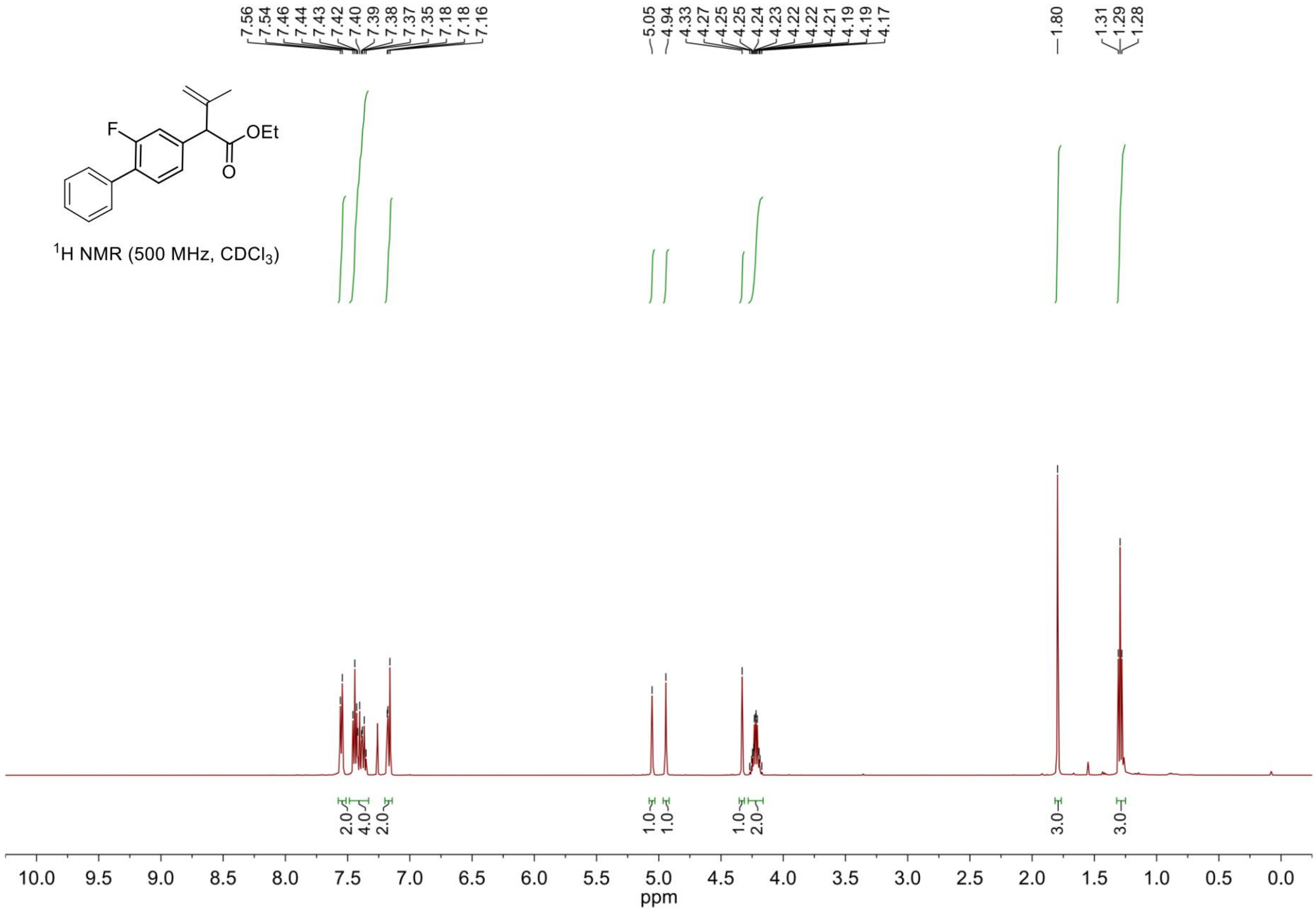

Ethyl 2-(2-fluoro-[1,1'-biphenyl]-4-yl)-3-methylbut-3-enoate (68)

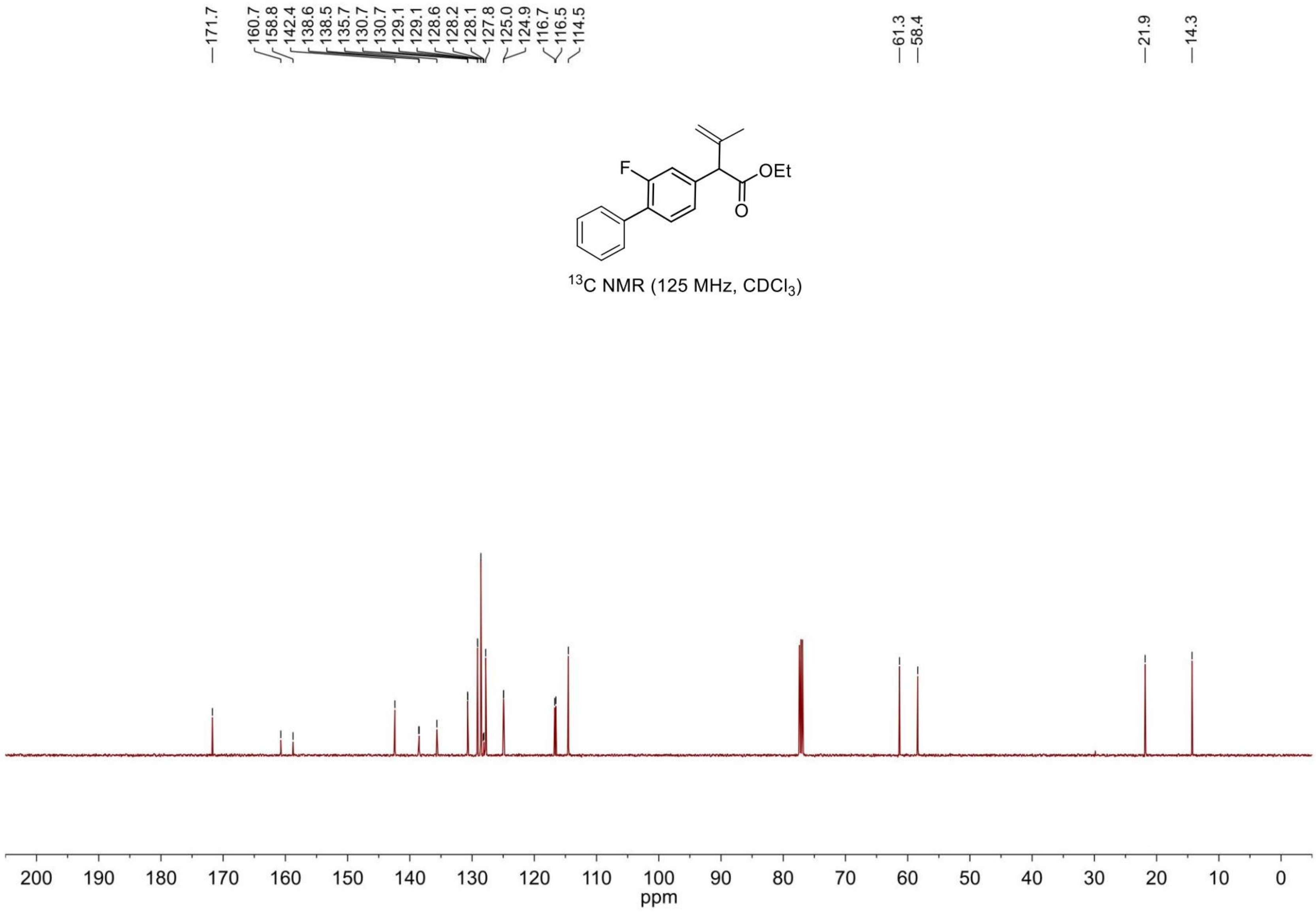

Go back to table of contents

Ethyl 2-(4-methoxyphenyl)-2-(thiophen-3-yl)acetate (69)

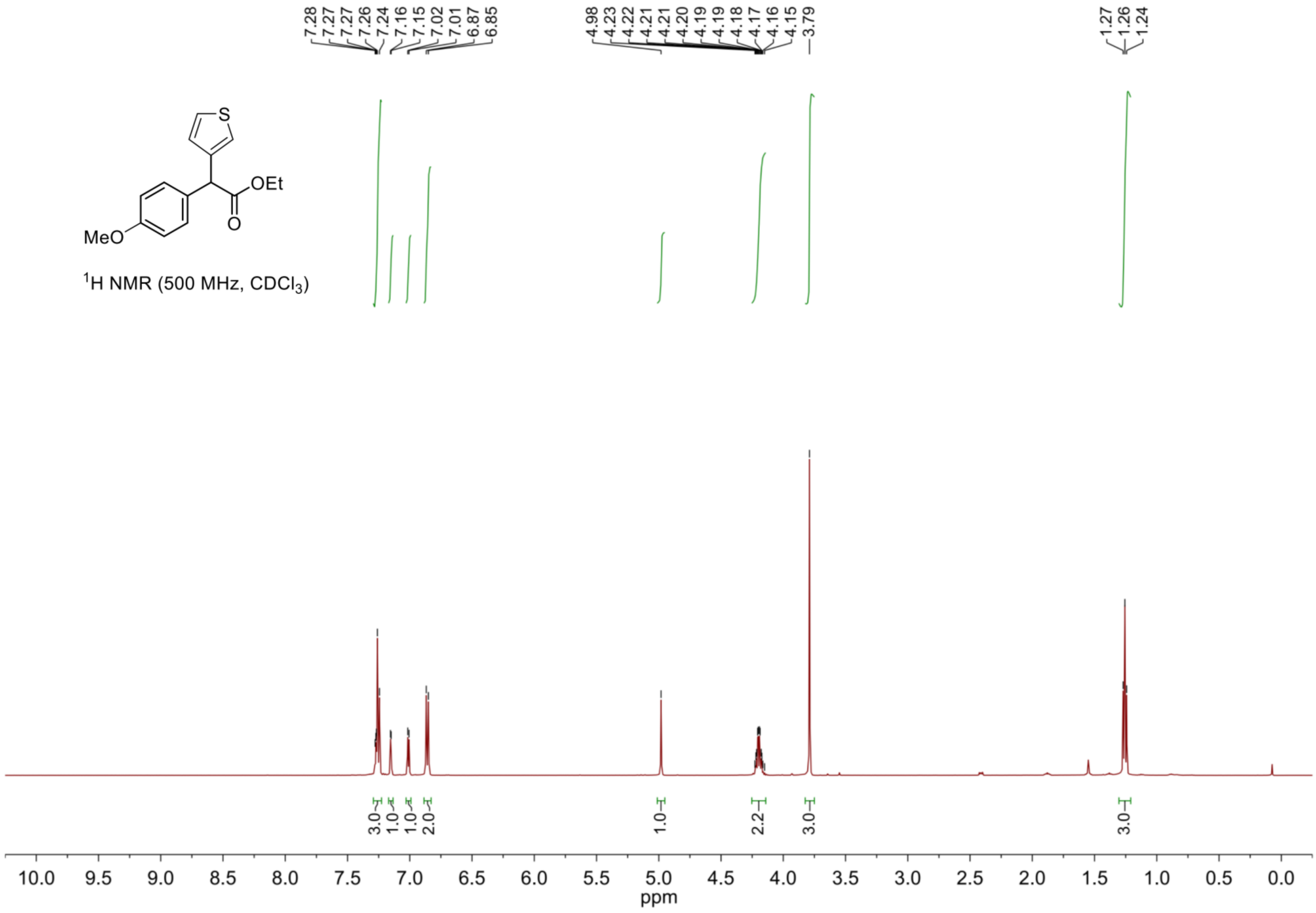

Go back to table of contents

Ethyl 2-(4-methoxyphenyl)-2-(thiophen-3-yl)acetate (69)

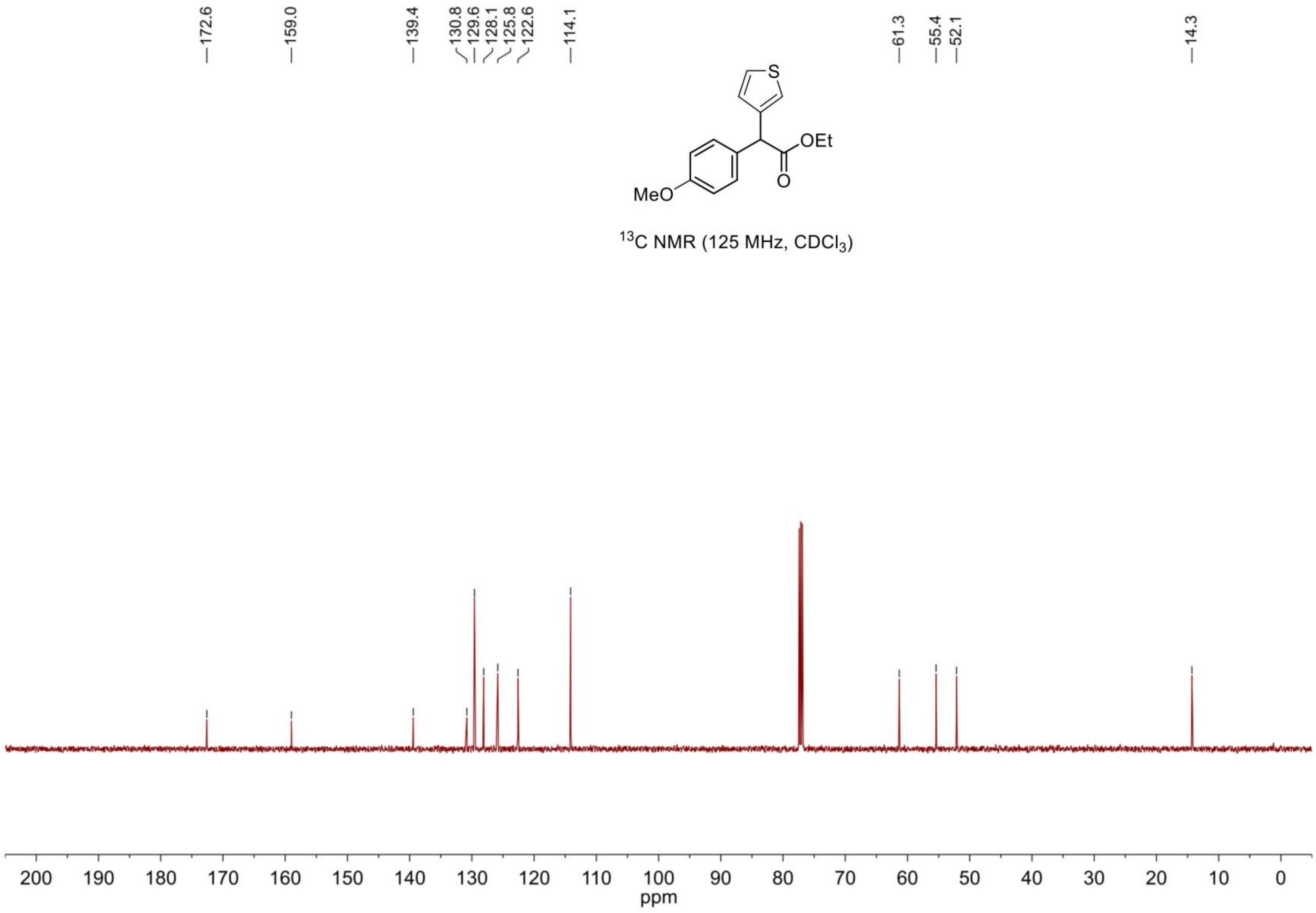

Go back to table of contents

Ethyl 2-(2,3-dihydrobenzofuran-5-yl)-2-(2-fluoro-[1,1'-biphenyl]-4-yl)acetate (70)

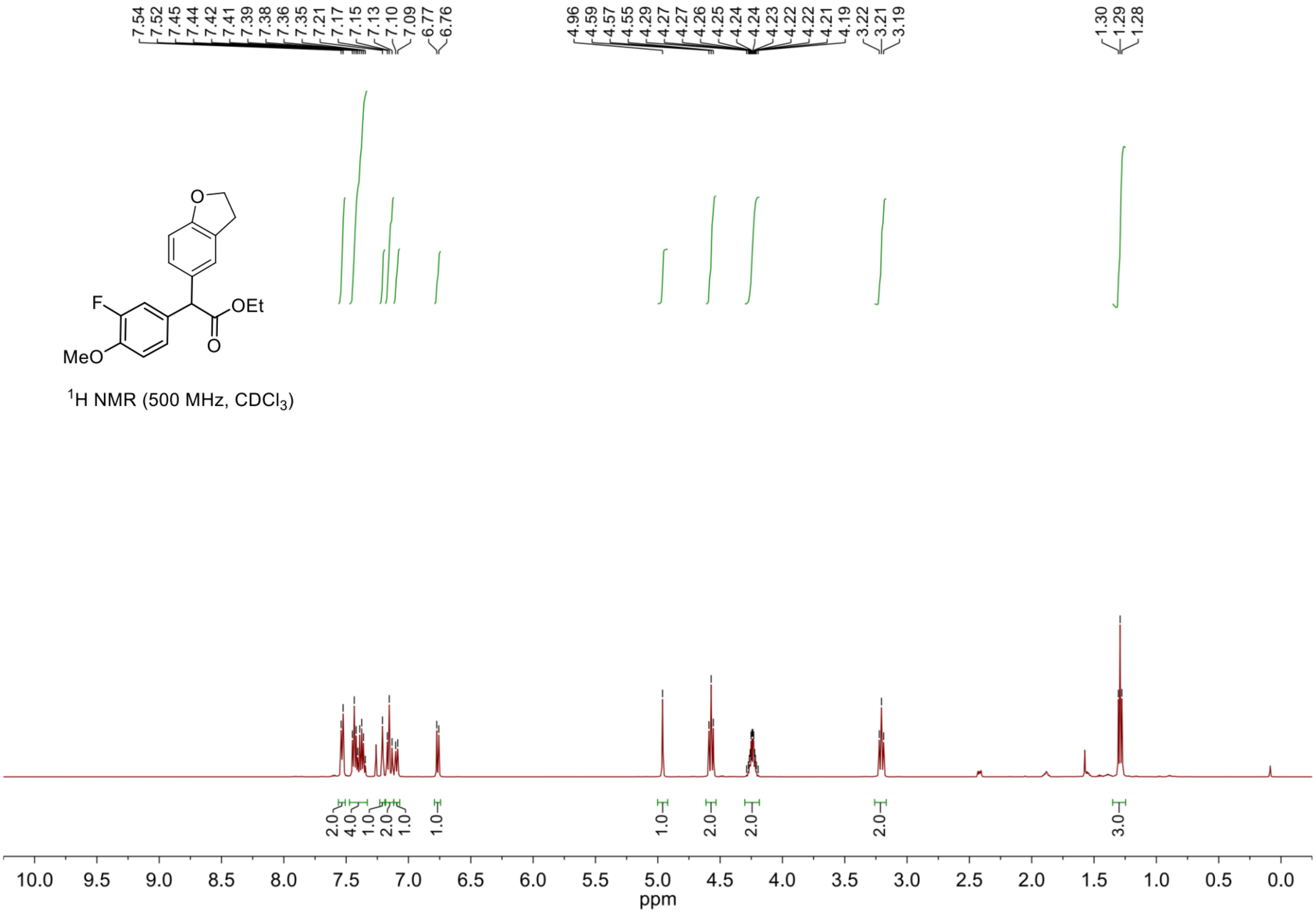

Ethyl 2-(2,3-dihydrobenzofuran-5-yl)-2-(2-fluoro-[1,1'-biphenyl]-4-yl)acetate (70)

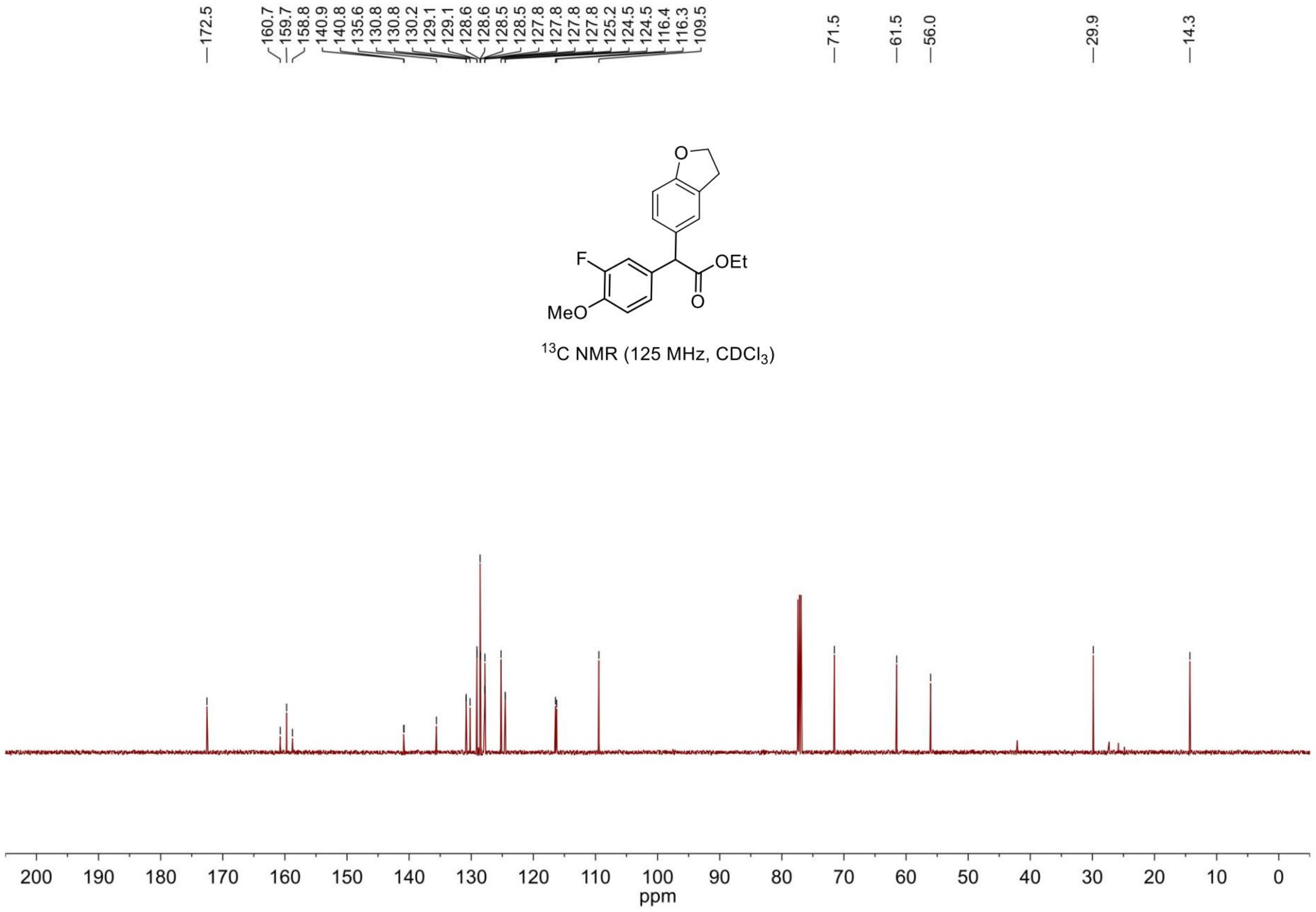

Go back to table of contents

Ethyl 2-(9,9-dimethyl-9*H*-xanthen-2-yl)-2-(1-methyl-1*H*-indol-5-yl)acetate (71)

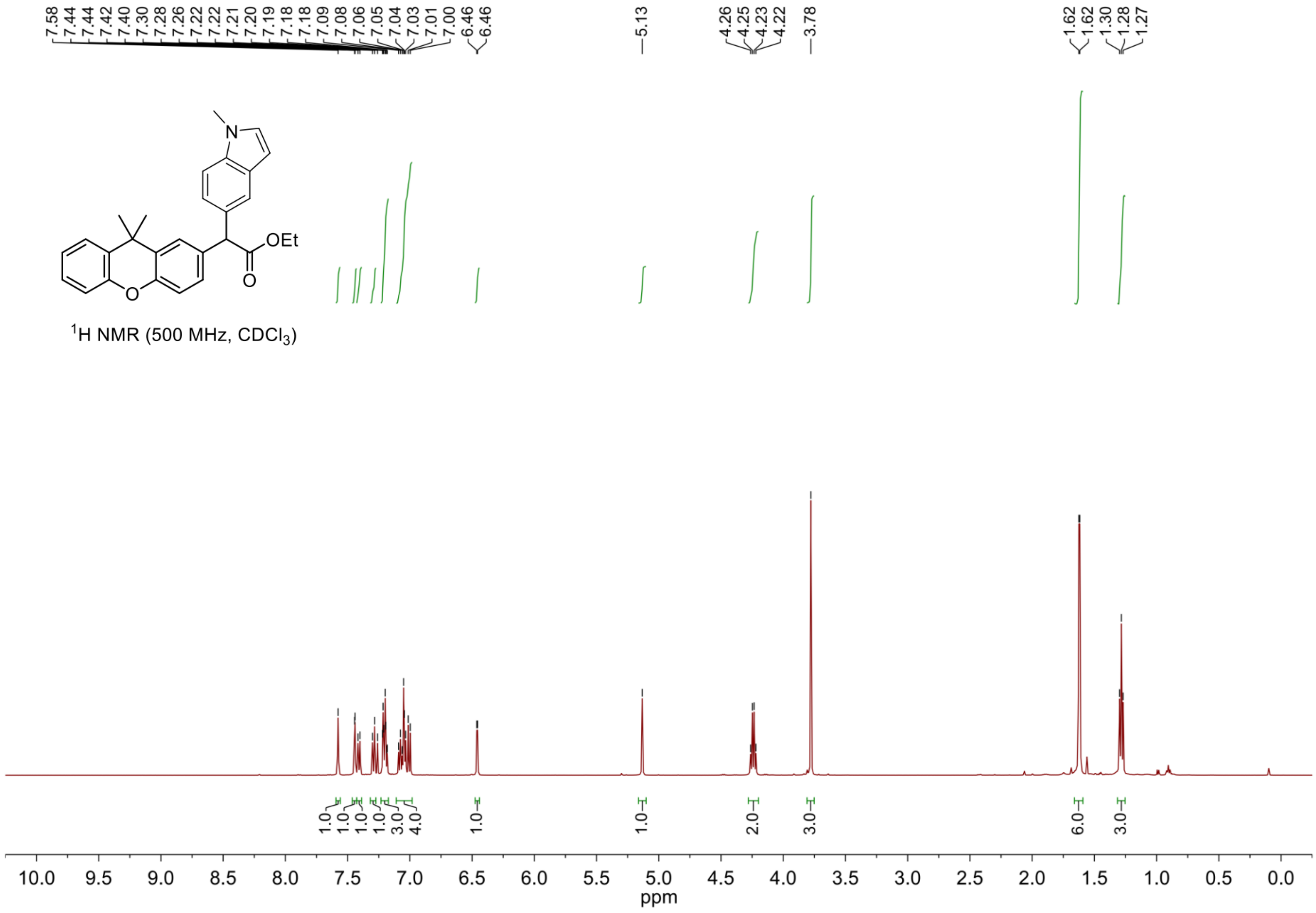

Go back to table of contents

Ethyl 2-(9,9-dimethyl-9*H*-xanthen-2-yl)-2-(1-methyl-1*H*-indol-5-yl)acetate (71)

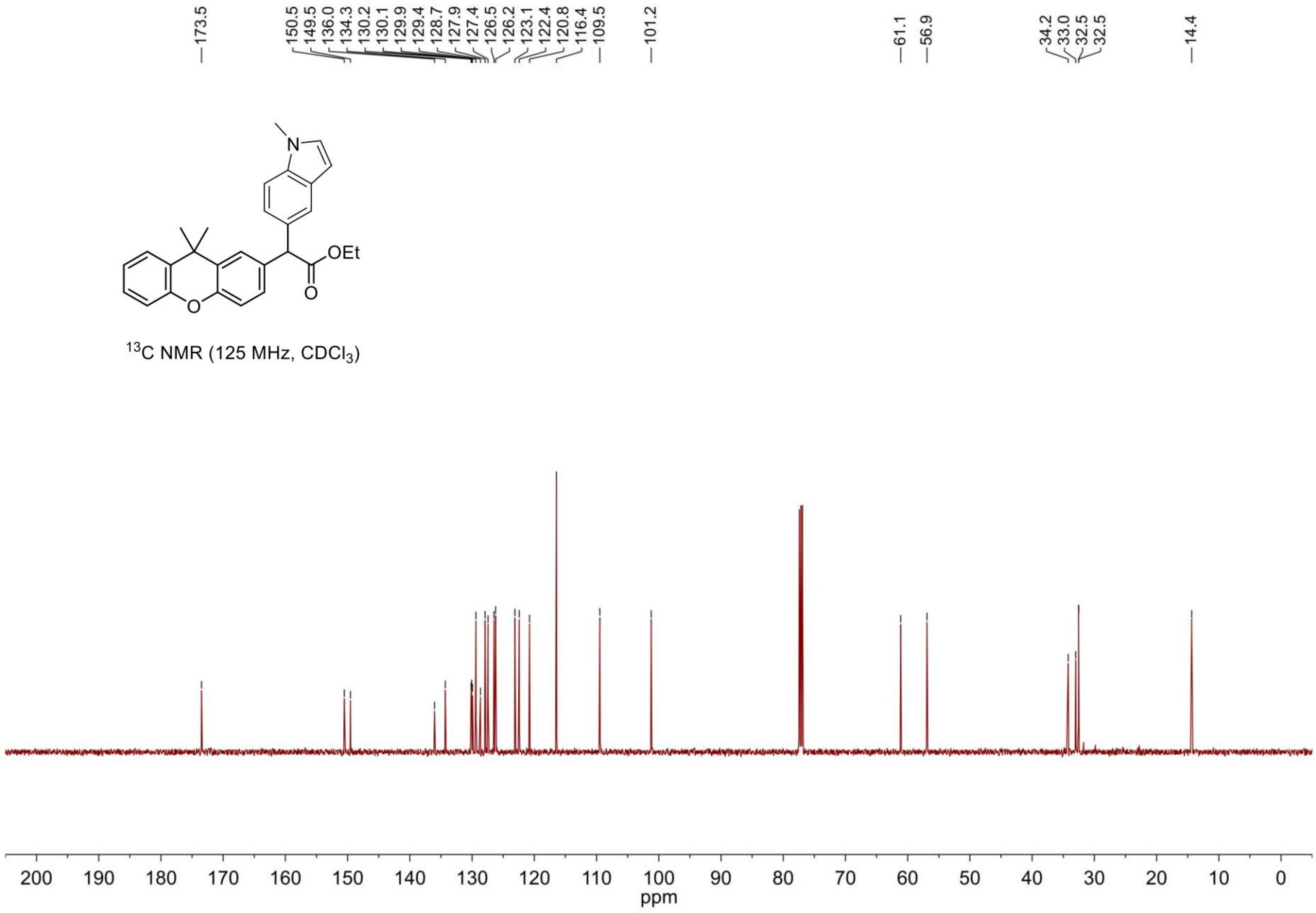

Go back to table of contents

Ibuprofen (69)

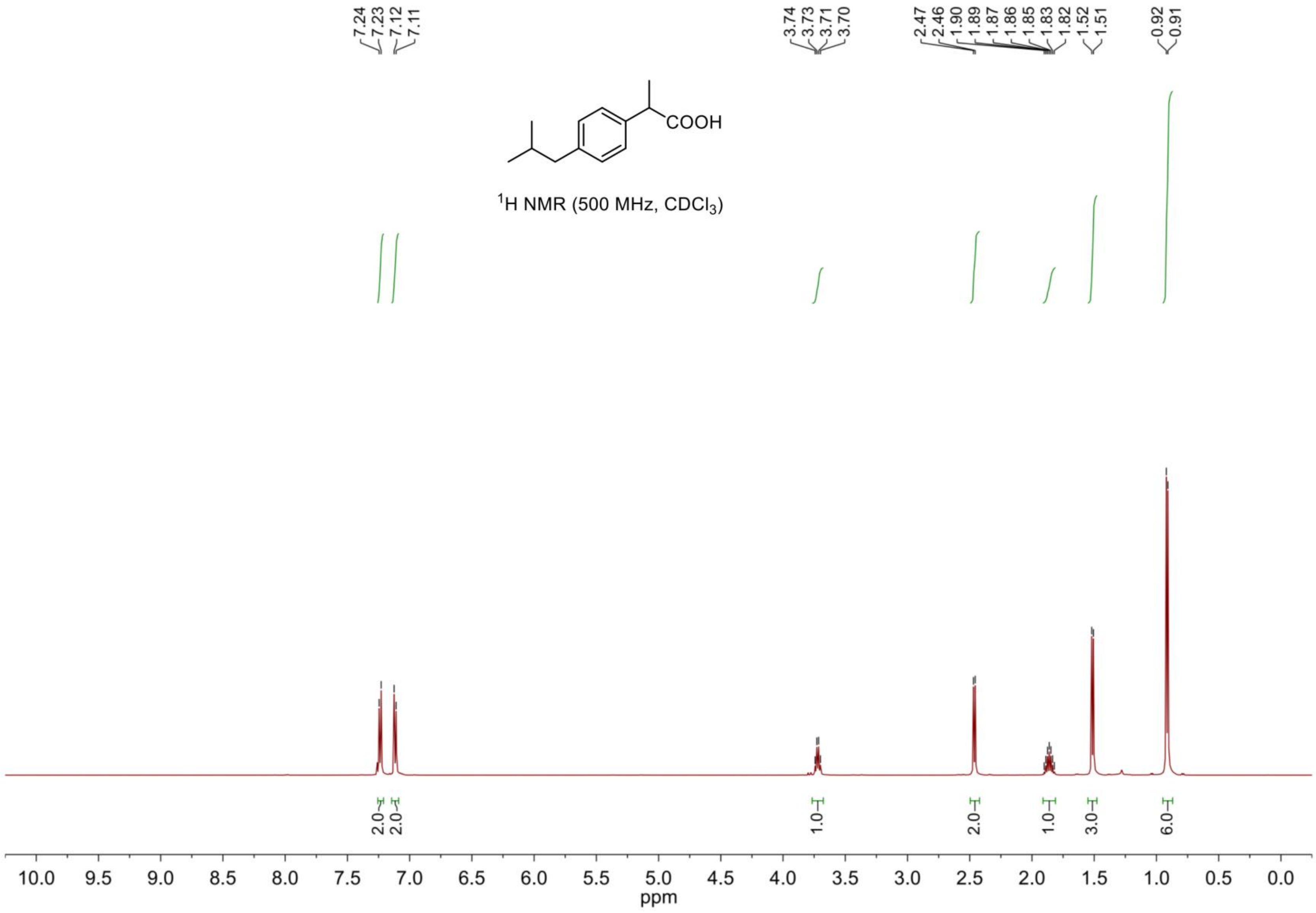

Go back to table of contents

Ibuprofen (72)

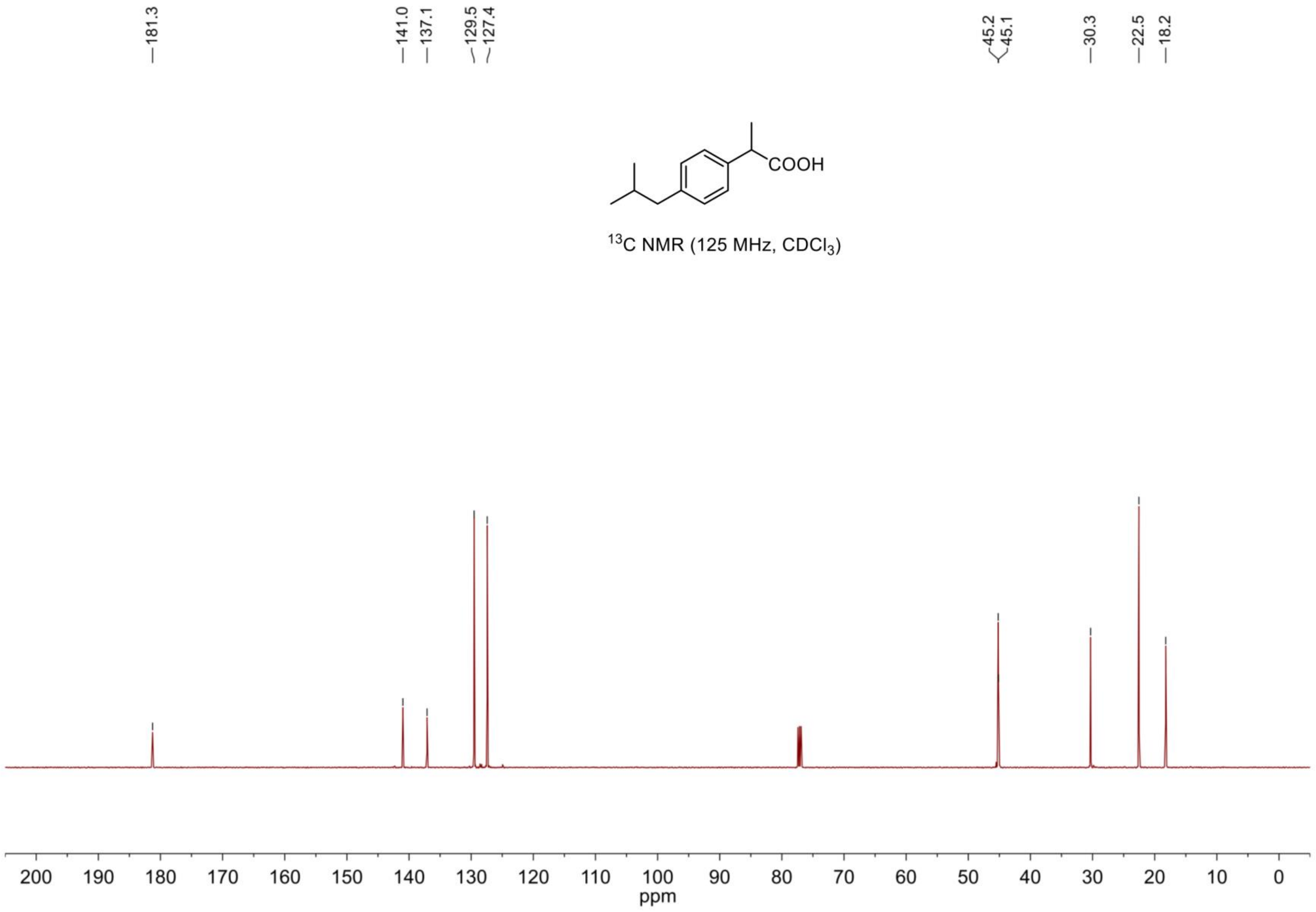

Go back to table of contents

Naproxen (72)

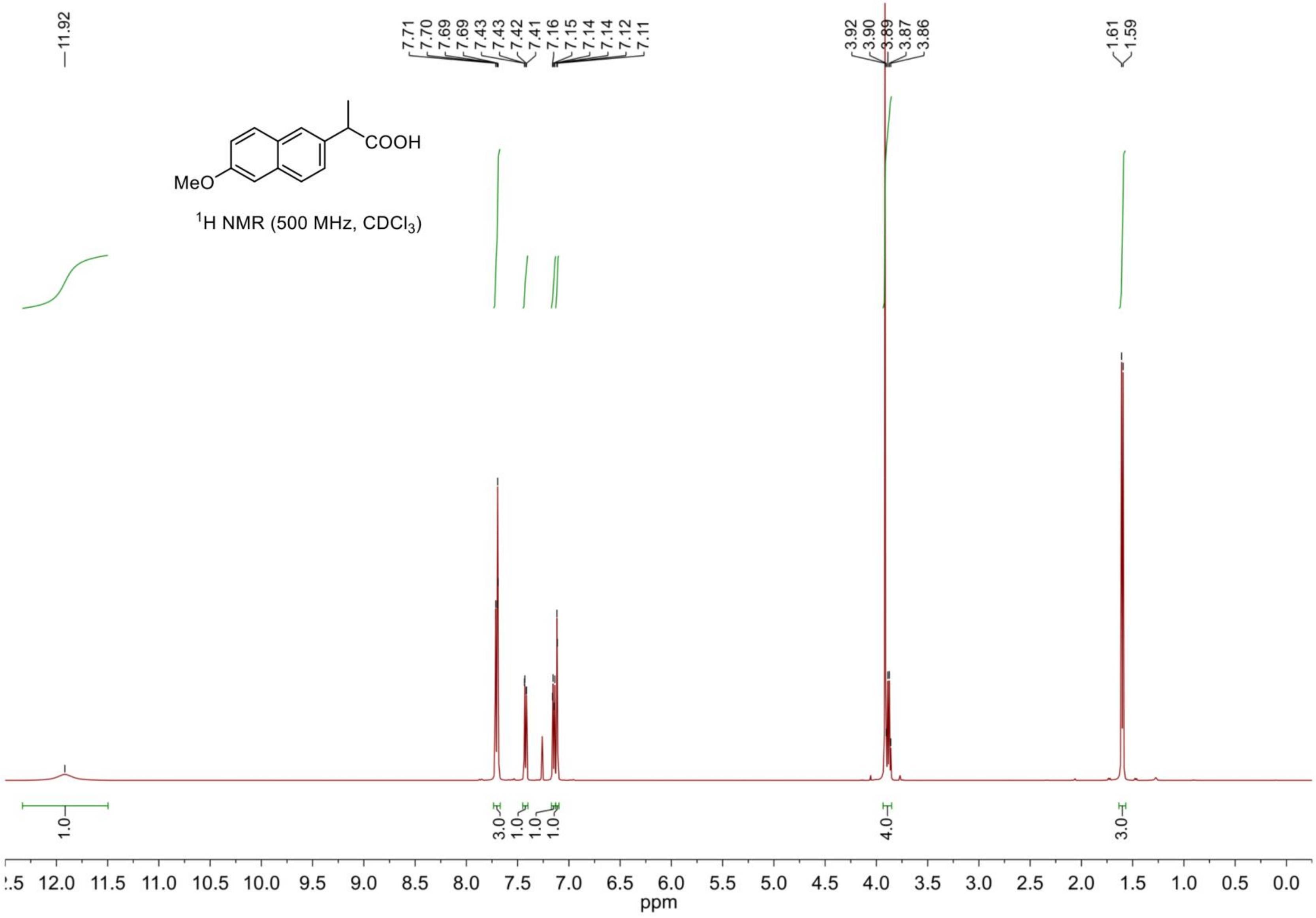

Go back to table of contents

Naproxen (73)

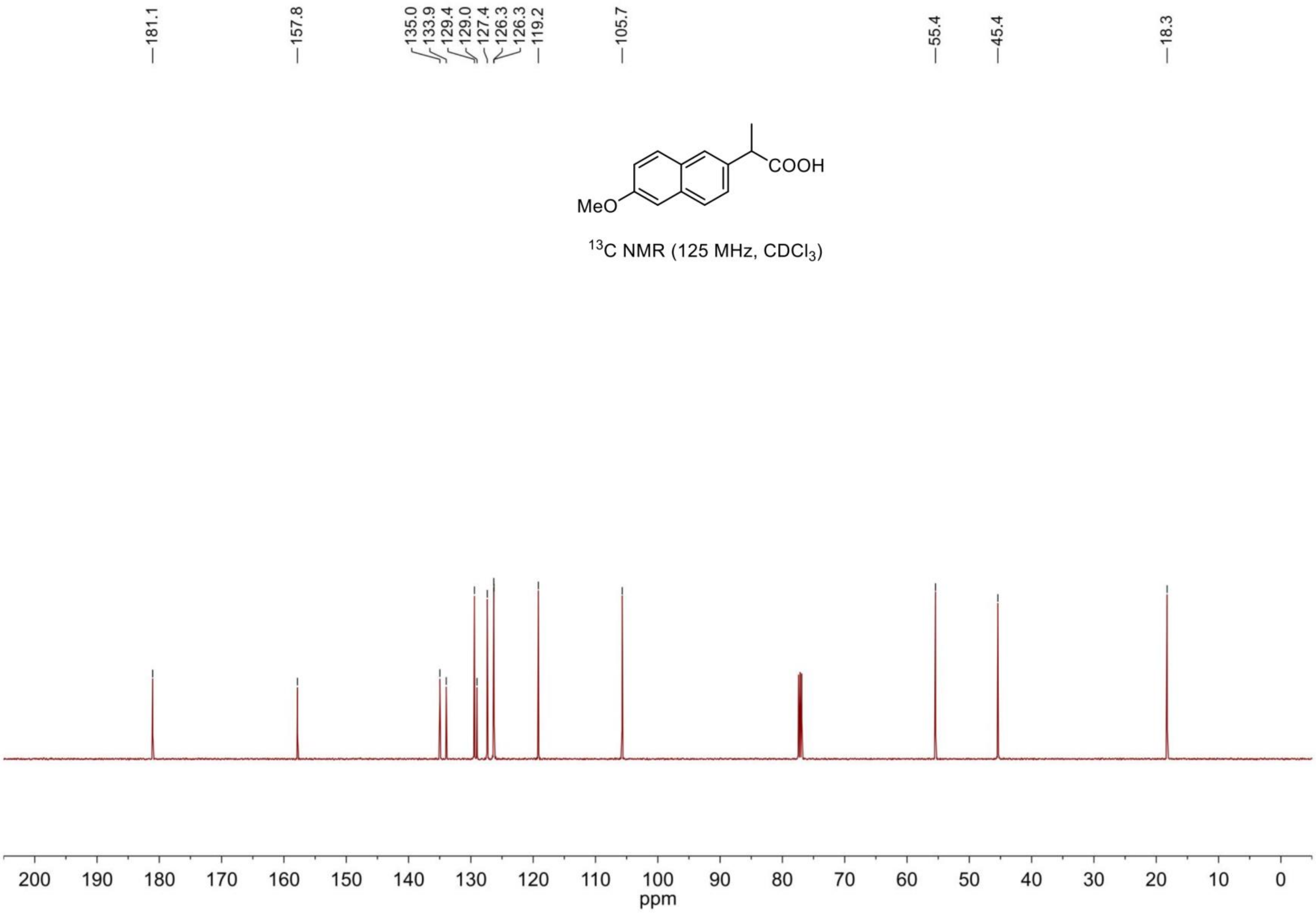
COOH
MeO
^{13}C NMR (125 MHz, $CDCl_3$)
181.1
157.8
135.0
133.9
129.4
129.0
127.4
126.3
126.3
119.2
105.7
55.4
45.4
18.3
200 190 180 170 160 150 140 130 120 110 100 90 80 70 60 50 40 30 20 10 0
ppm

Flurbiprofen (74)

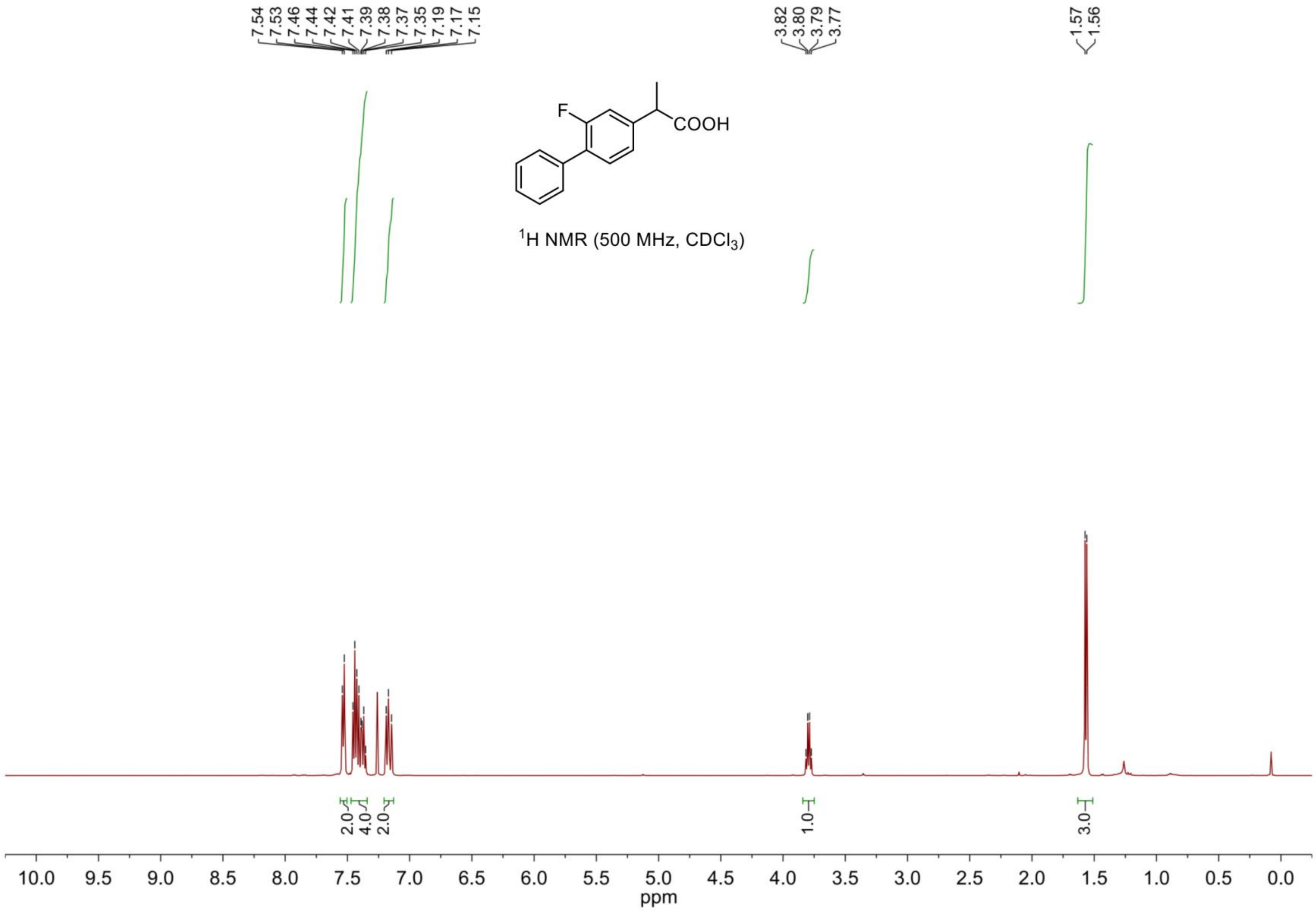

Go back to table of contents

Flurbiprofen (74)

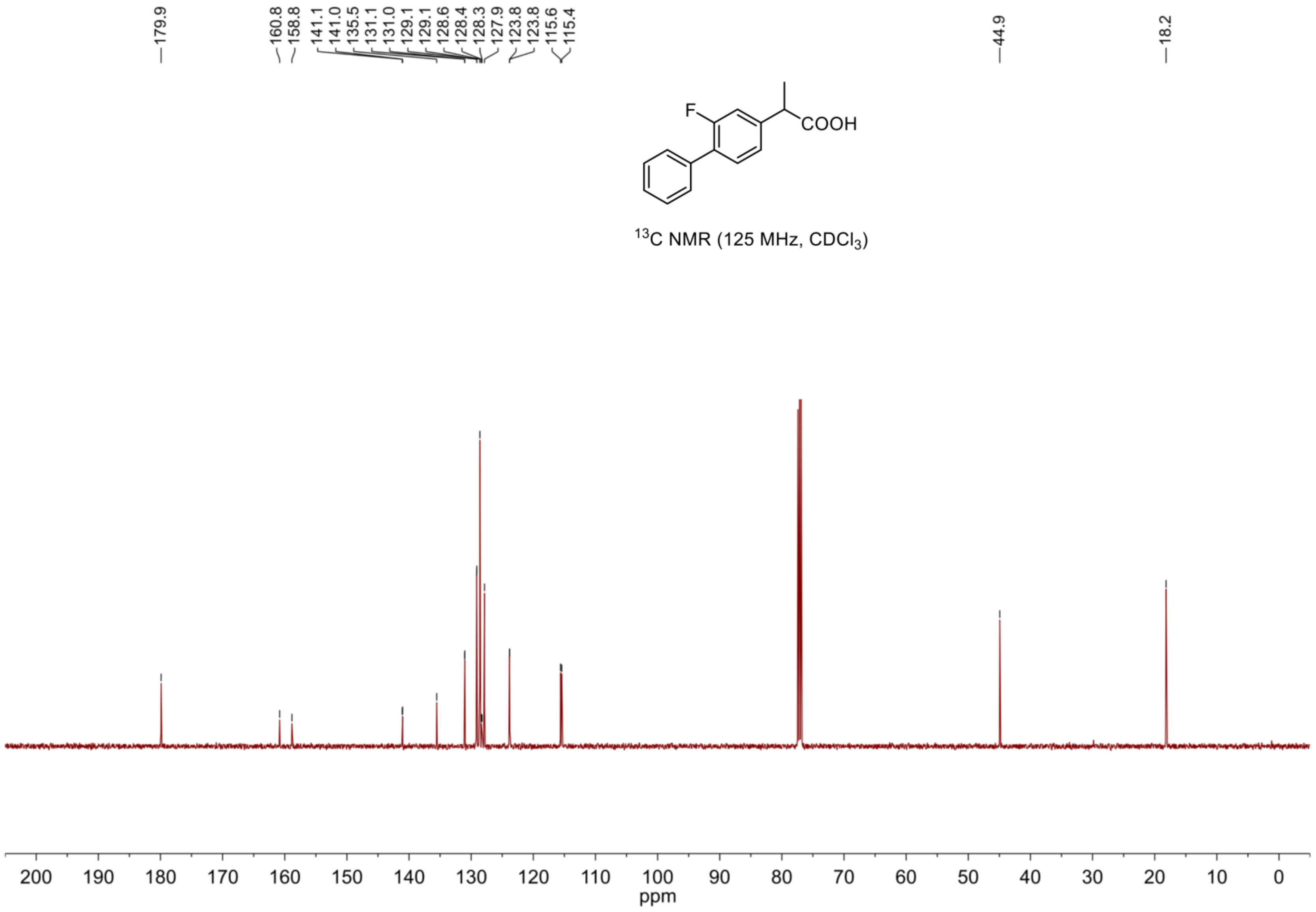

Go back to table of contents

Ethyl 2-phenylbutanoate-2-*d* (78)

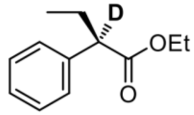

^{1}H NMR (500 MHz, $CDCl_3$)

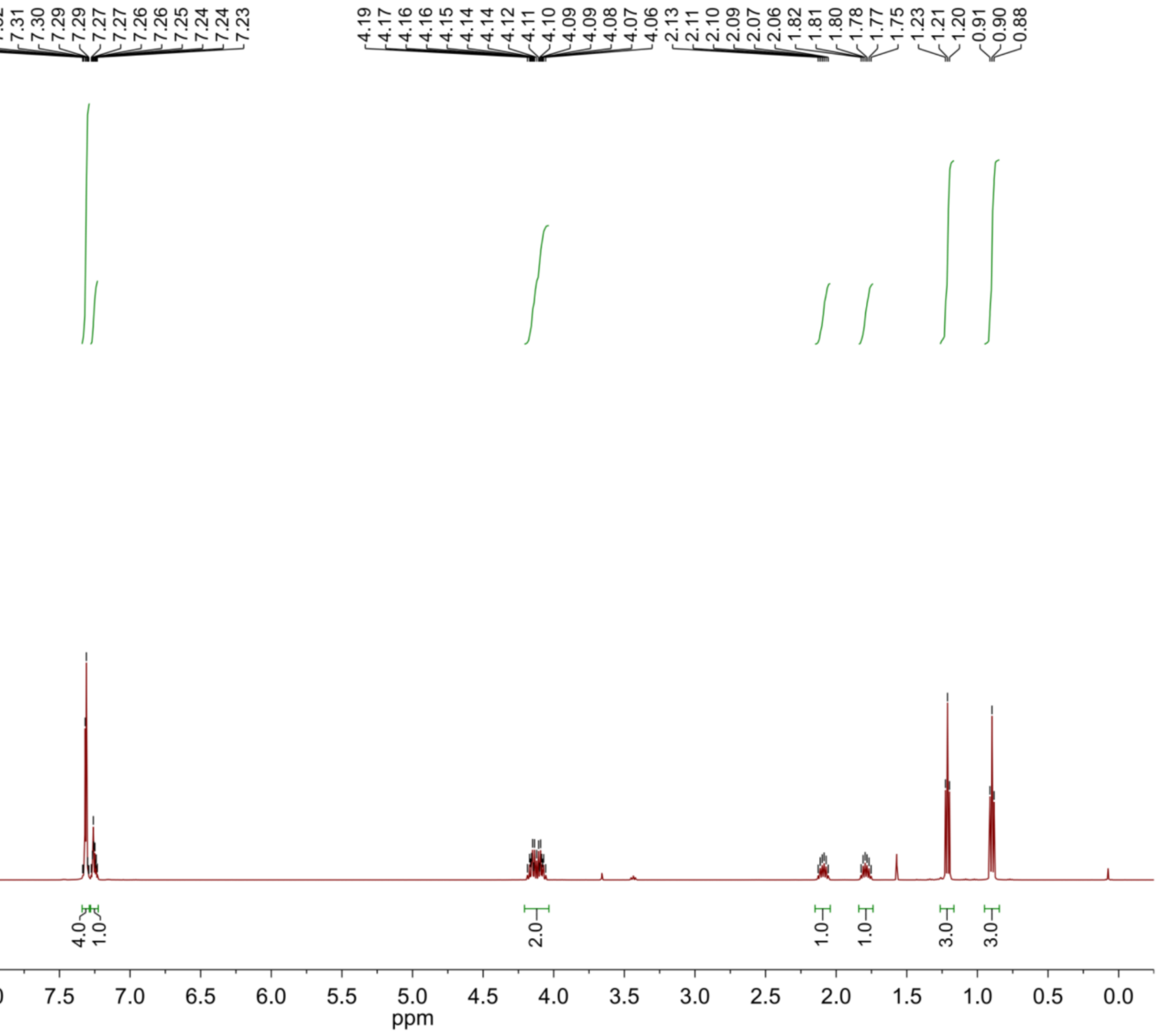

Go back to table of contents

Ethyl 2-phenylbutanoate-2-*d* (78)

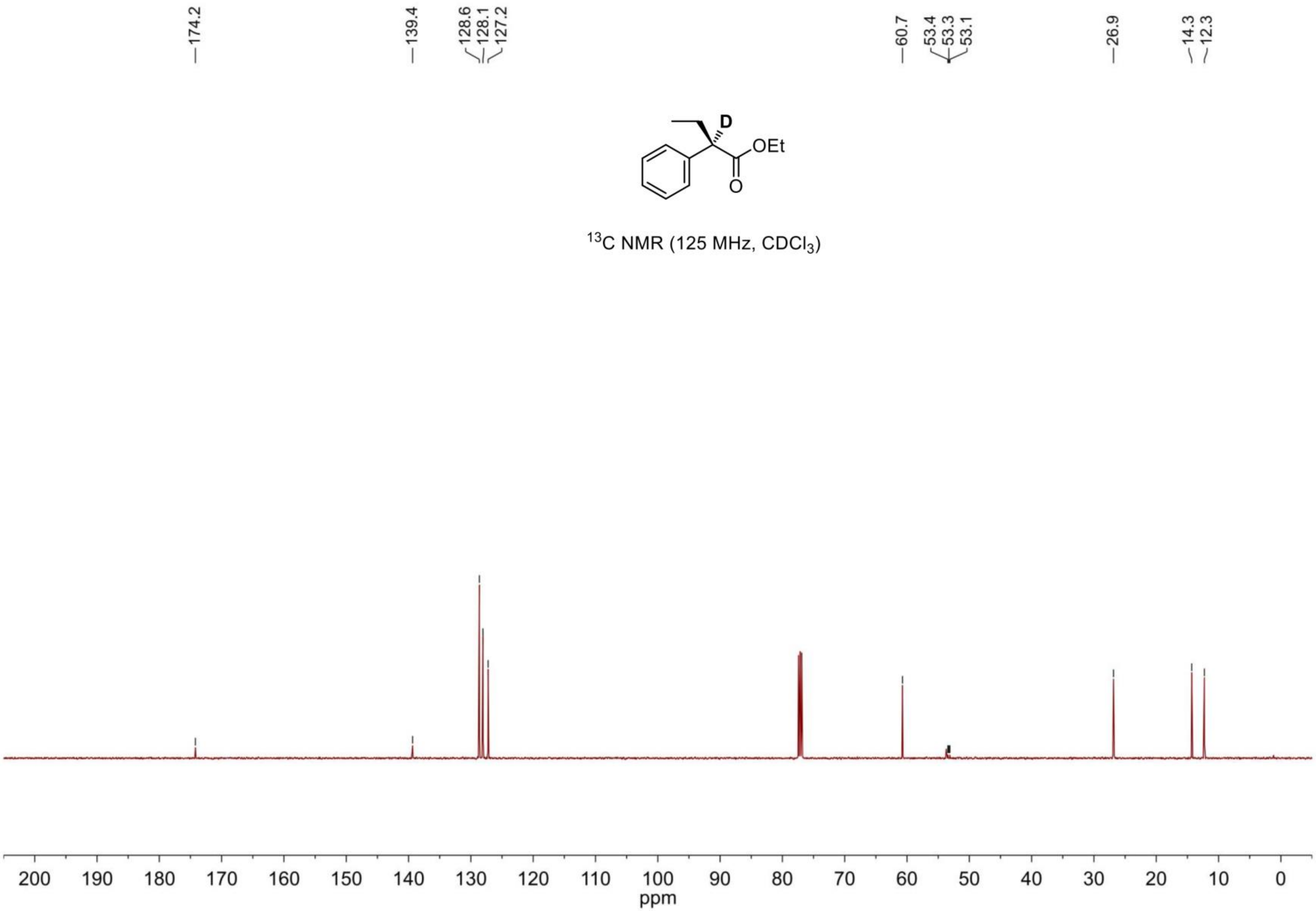

Go back to table of contents

Ethyl 2-ethyl-3-hydroxy-3-methyl-2-phenylbutanoate (79)

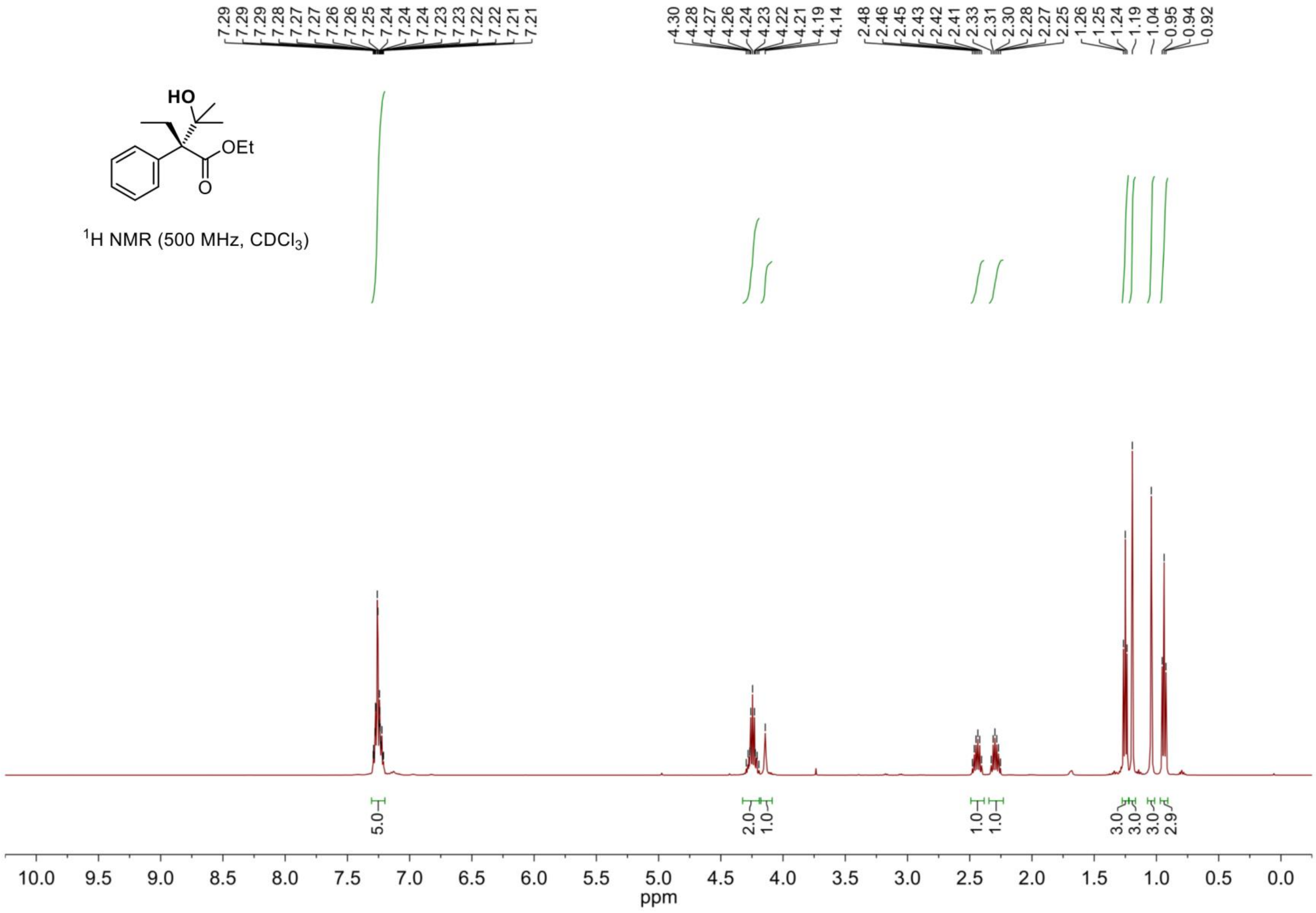

^1H NMR (500 MHz, $CDCl_3$)

Go back to table of contents

Ethyl 2-ethyl-3-hydroxy-3-methyl-2-phenylbutanoate (79)

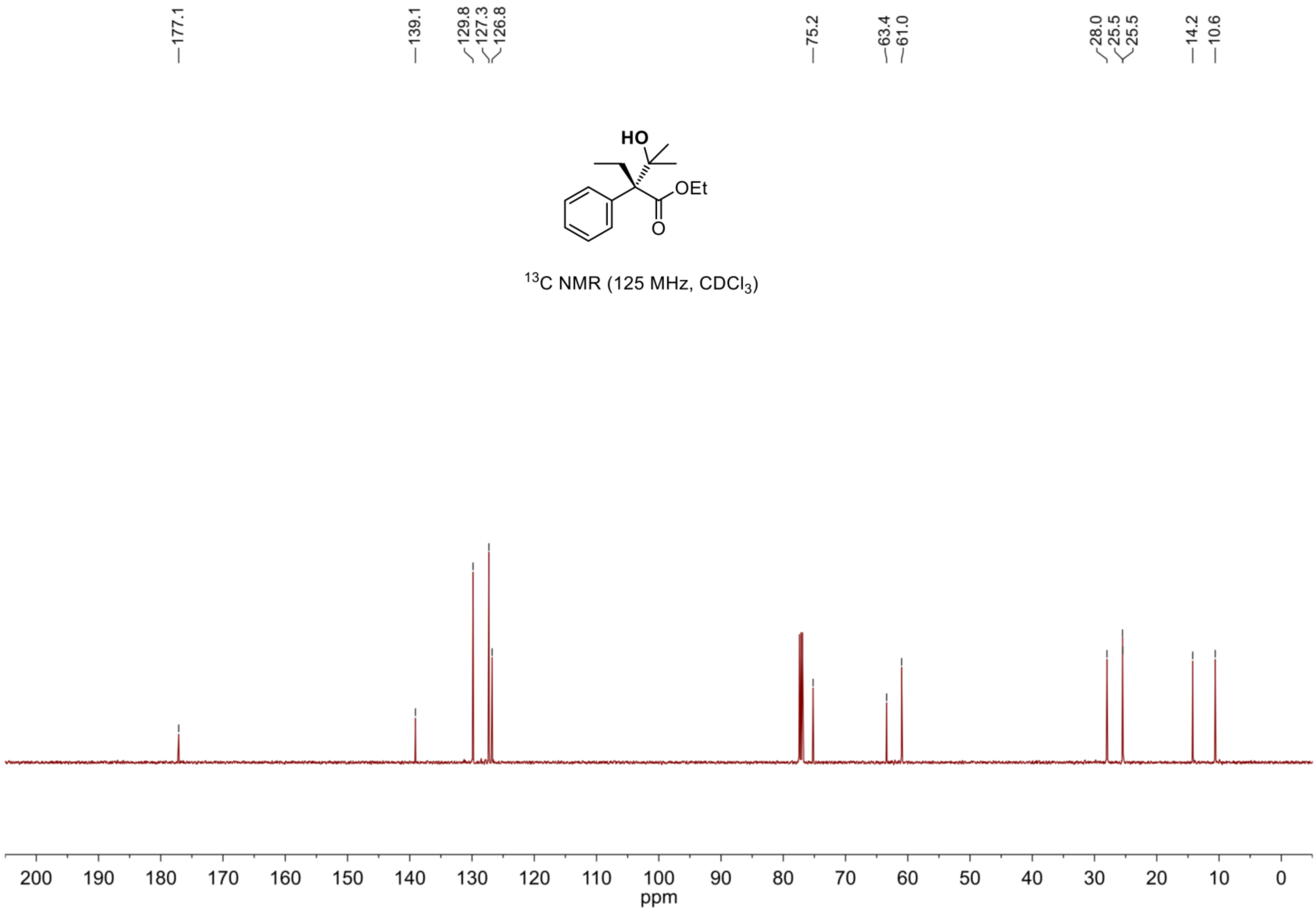

Go back to table of contents

Ethyl 2-ethyl-3-hydroxy-3-(methyl-d3)-2-phenylbutanoate-4,4,4-*d*3 (79-D)

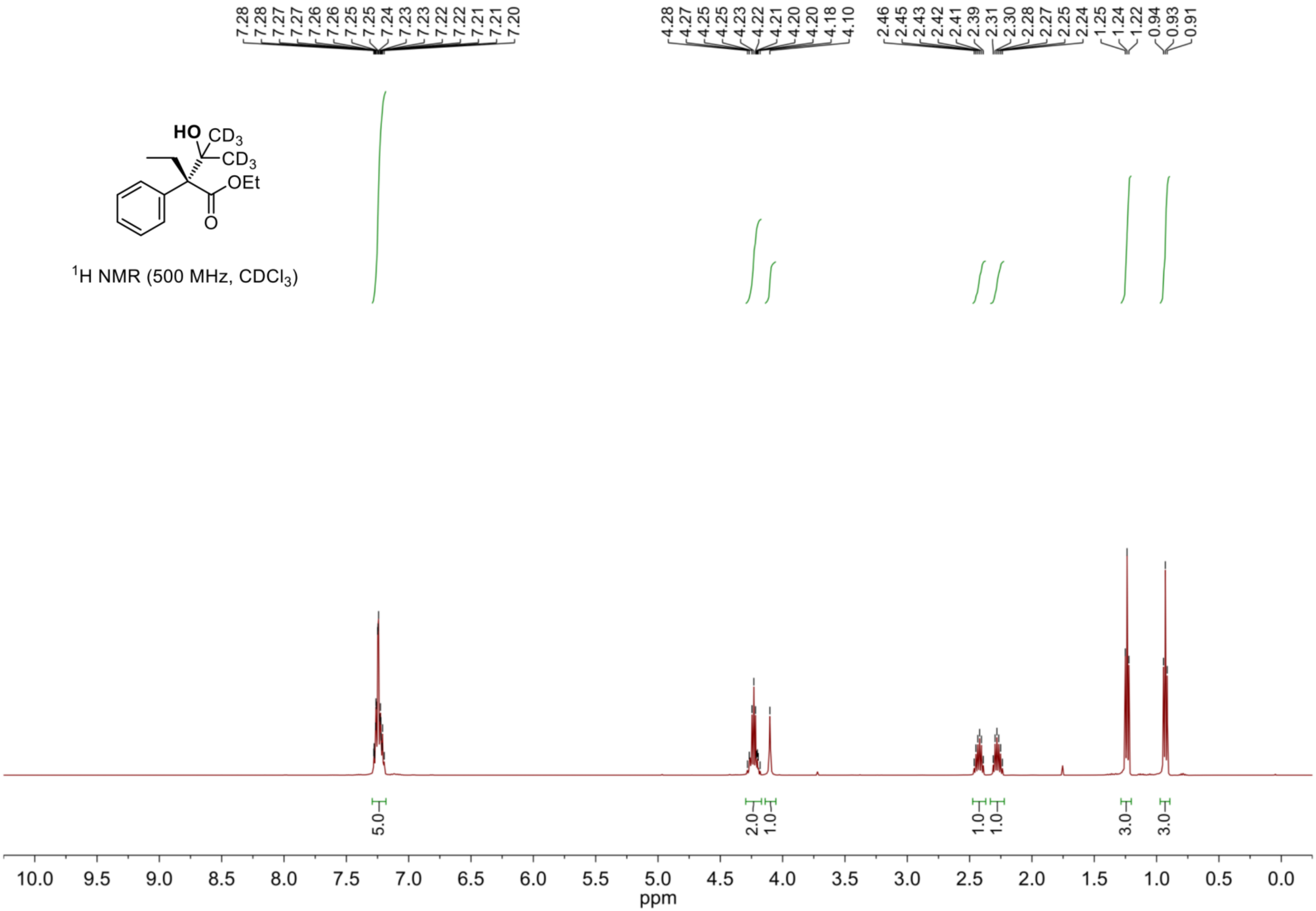

Go back to table of contents

Ethyl 2-ethyl-3-hydroxy-3-(methyl-d3)-2-phenylbutanoate-4,4,4-*d*3 (79-D)

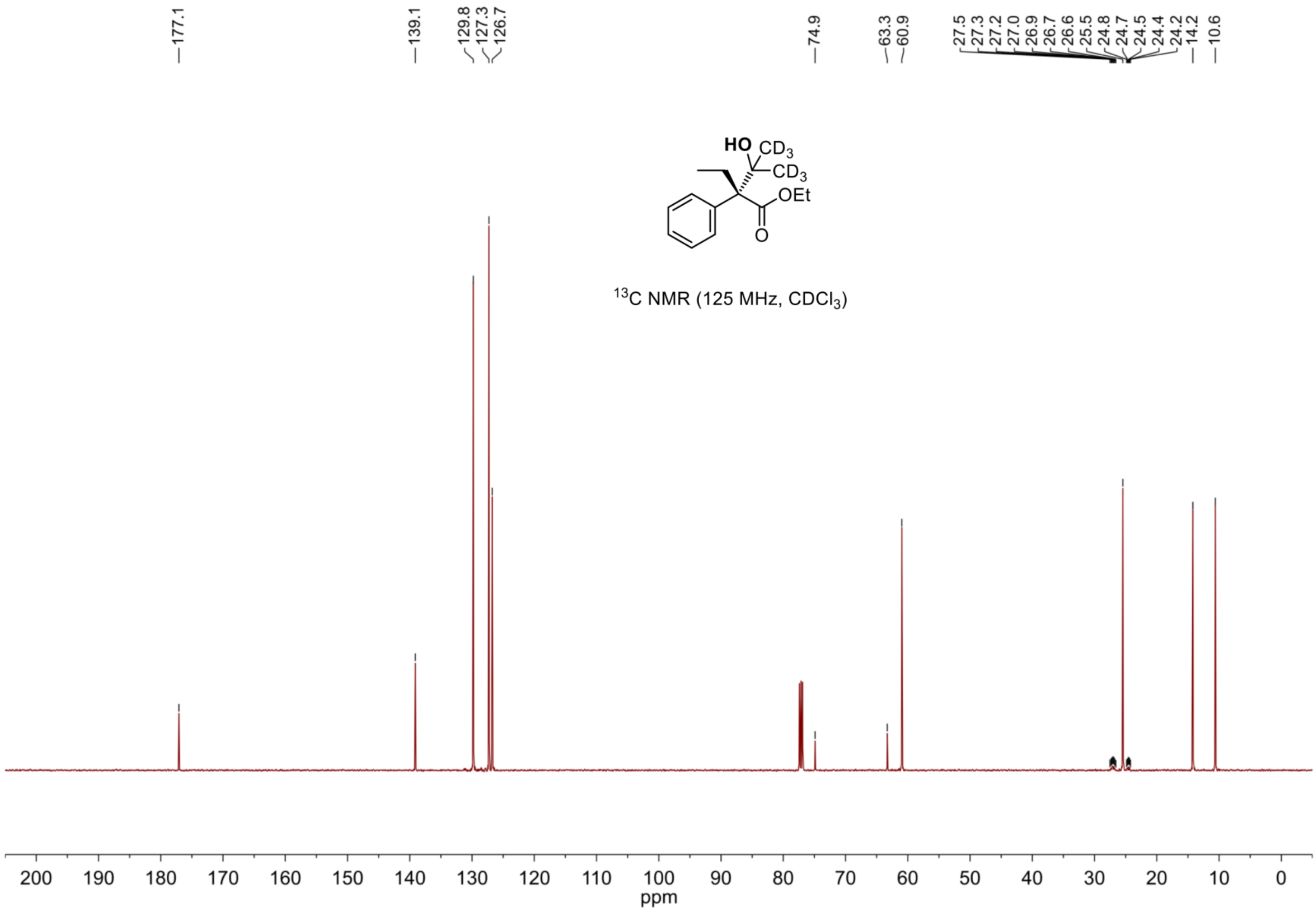

Ethyl 2-(1-hydroxycyclopentyl)-2-phenylbutanoate (80)

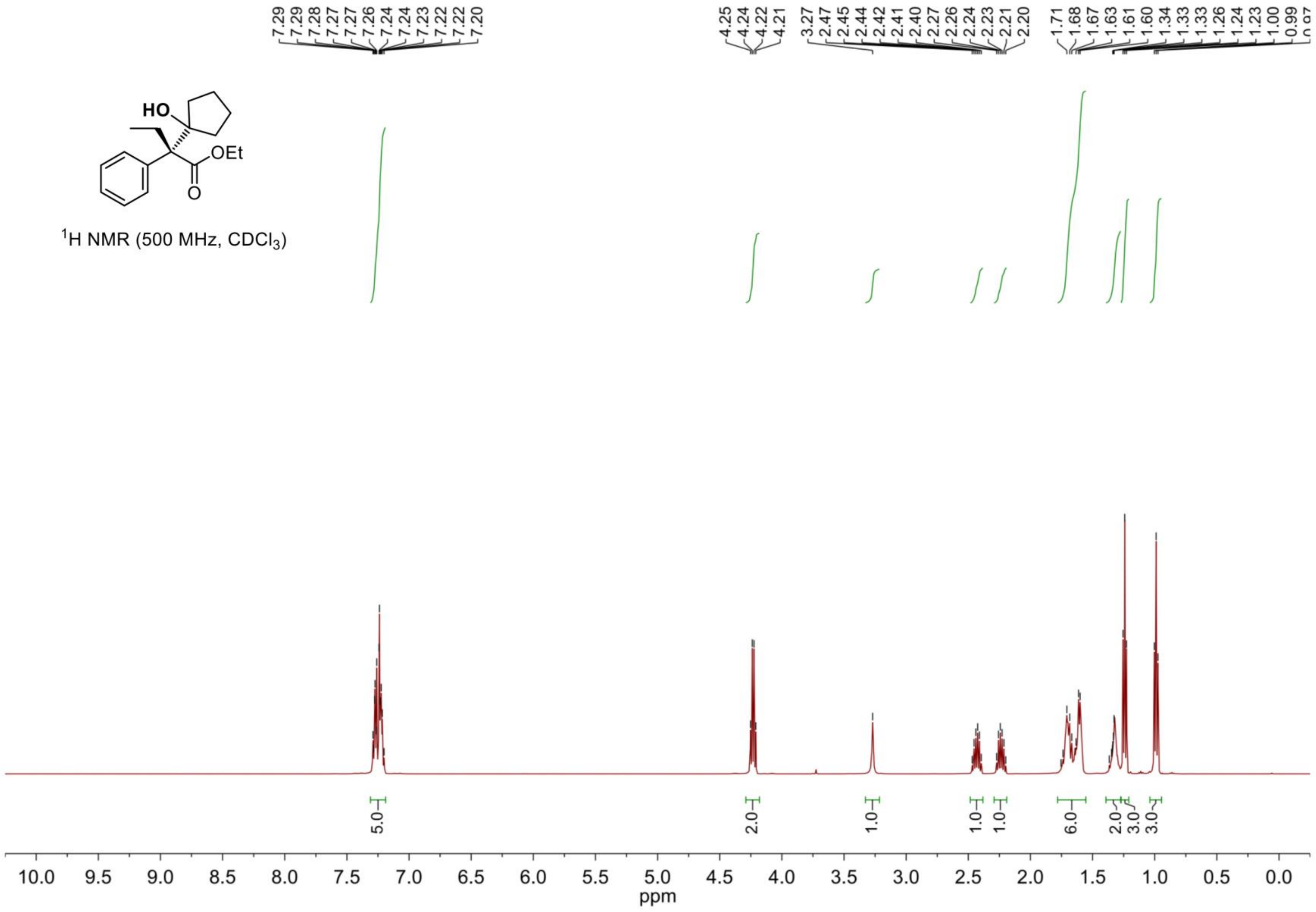

Go back to table of contents

Ethyl 2-(1-hydroxycyclopentyl)-2-phenylbutanoate (80)

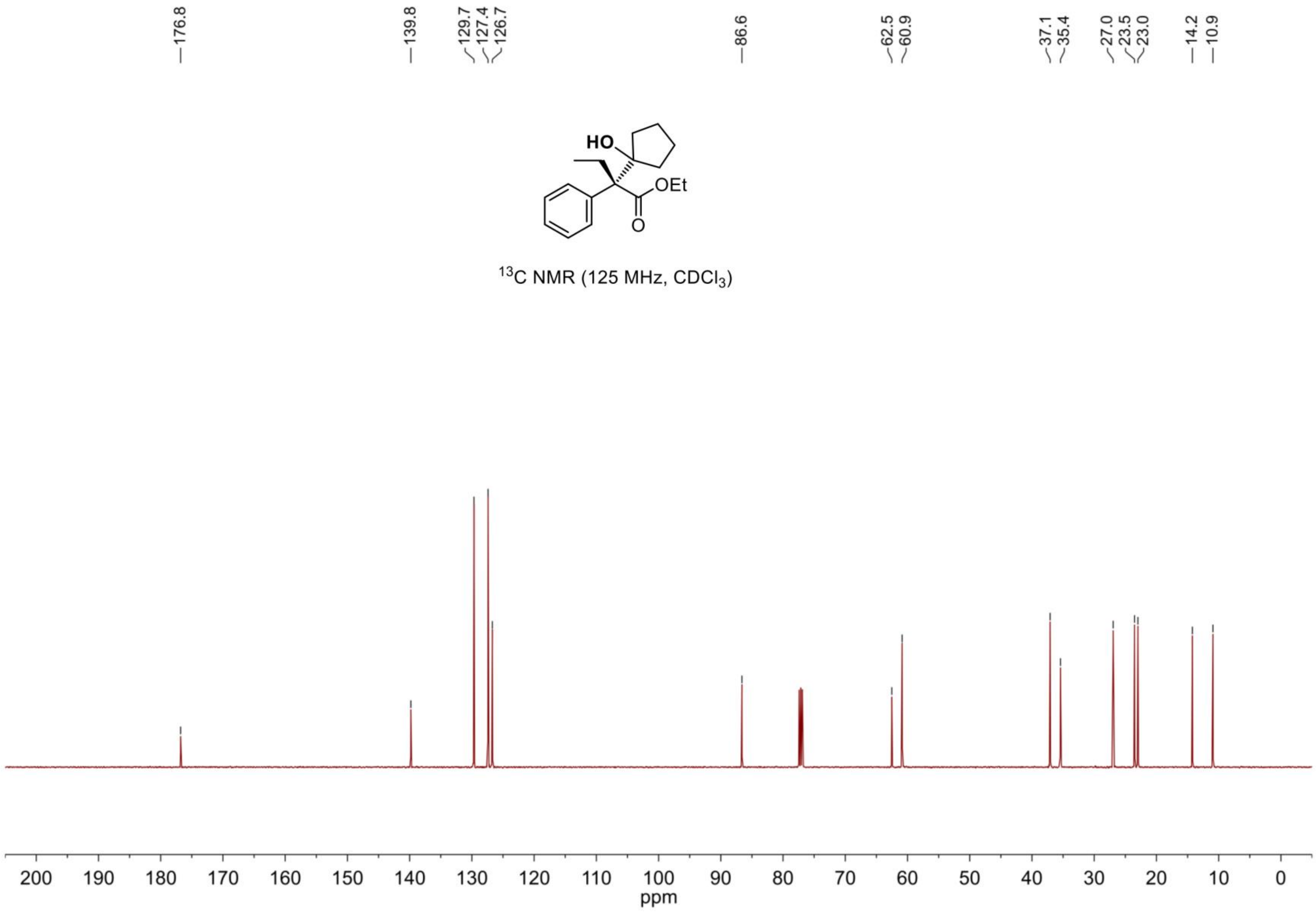

Go back to table of contents

Ethyl 2-(1-hydroxycyclohexyl)-2-phenylbutanoate (81)

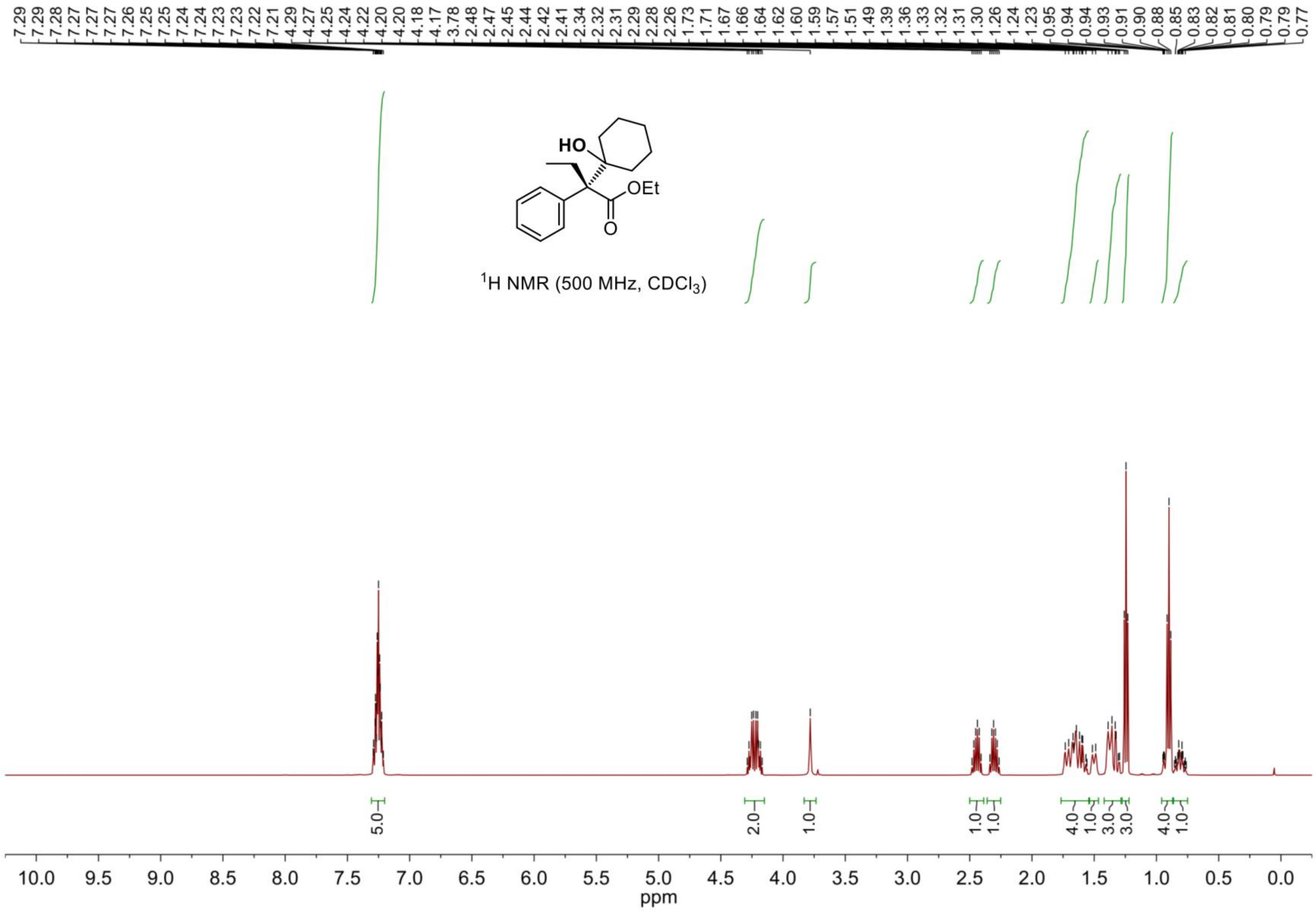

Ethyl 2-(1-hydroxycyclohexyl)-2-phenylbutanoate (81)

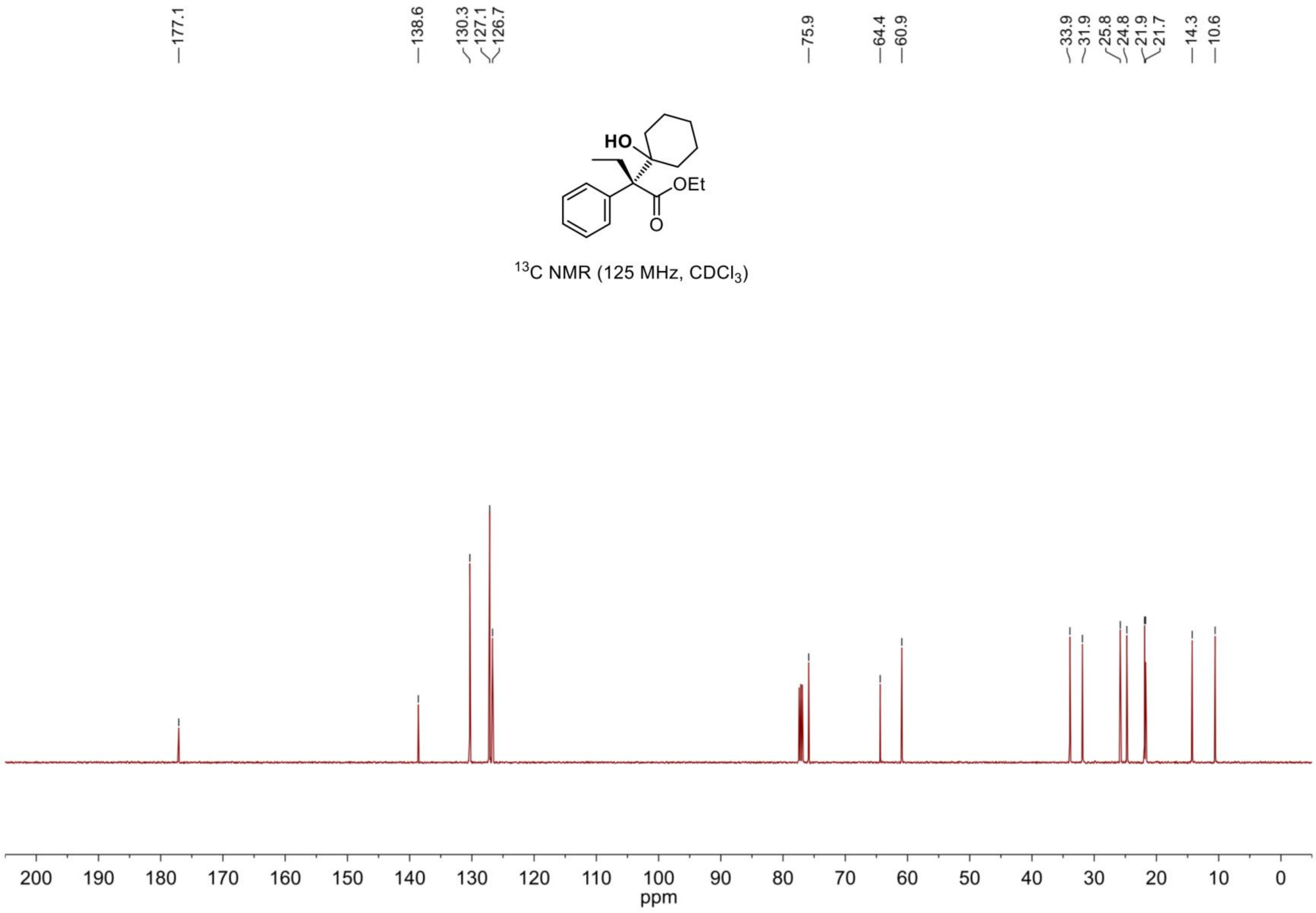

Go back to table of contents

tert-Butyl 4-(1-ethoxy-1-oxo-2-phenylbutan-2-yl)-4-hydroxypiperidine-1-carboxylate (82)

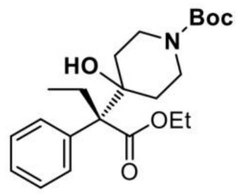

^1H NMR (500 MHz, $CDCl_3$)

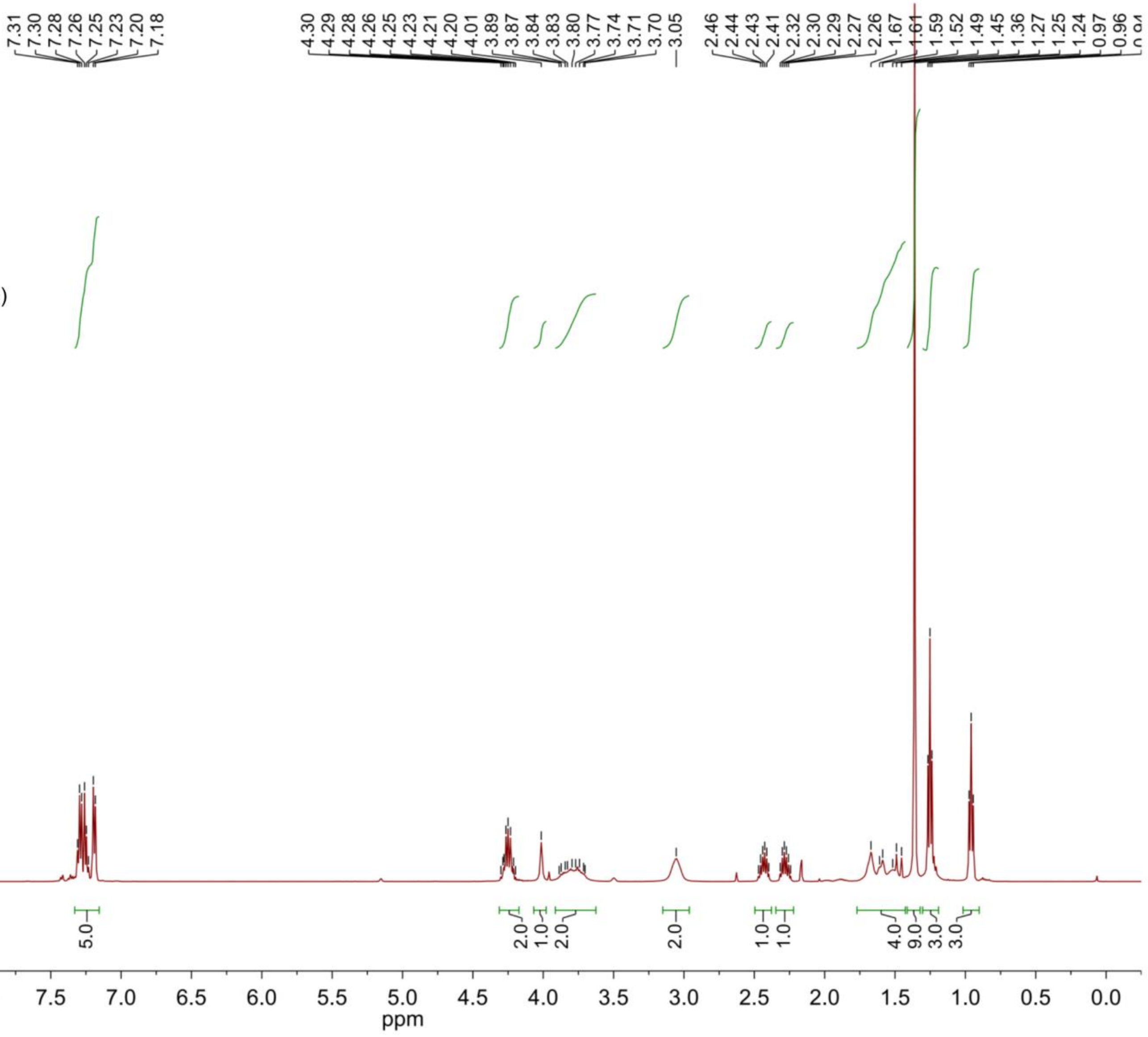

Go back to table of contents

tert-Butyl 4-(1-ethoxy-1-oxo-2-phenylbutan-2-yl)-4-hydroxypiperidine-1-carboxylate (82)

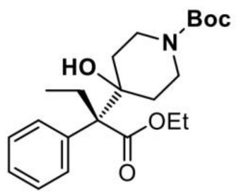

^{13}C NMR (125 MHz, $CDCl_3$)

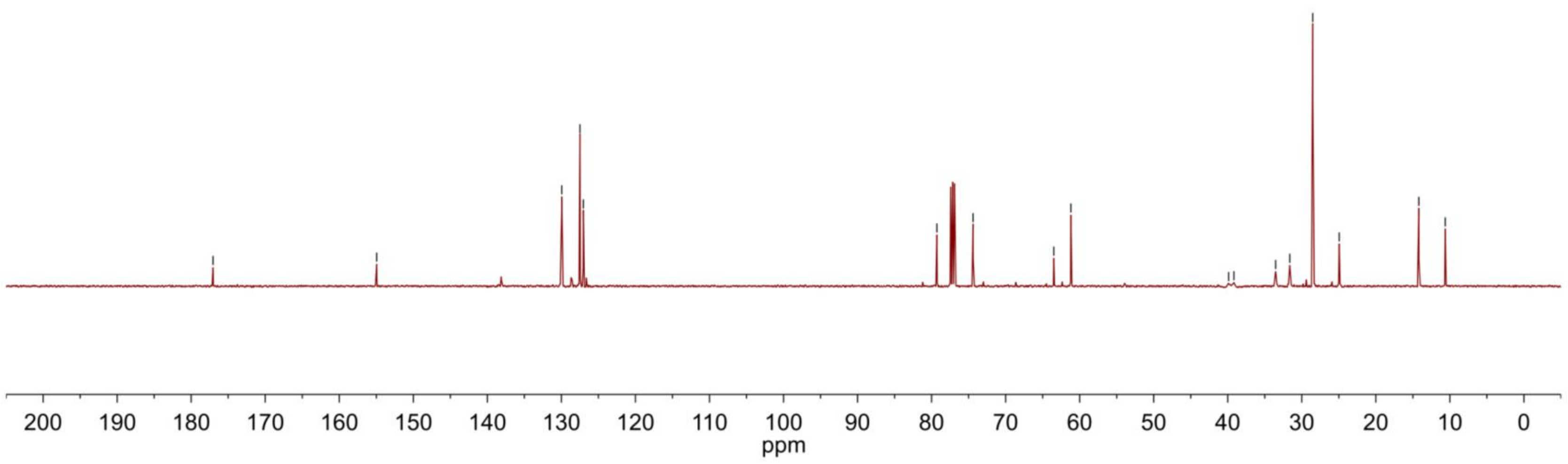

Go back to table of contents

Ethyl 2-(1-hydroxycyclobutyl)-2-phenylbutanoate (83)

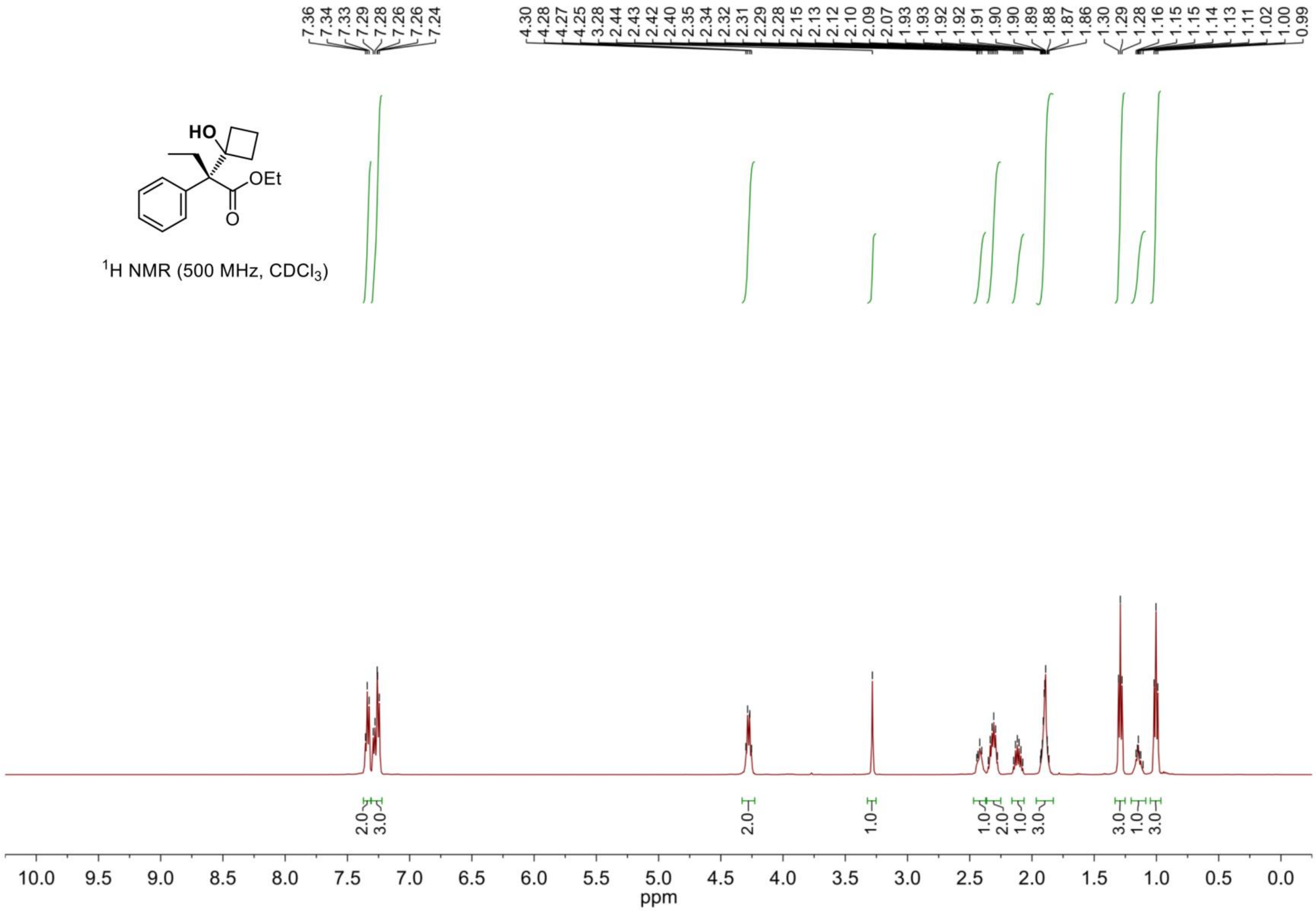

Go back to table of contents

Ethyl 2-(1-hydroxycyclobutyl)-2-phenylbutanoate (83)

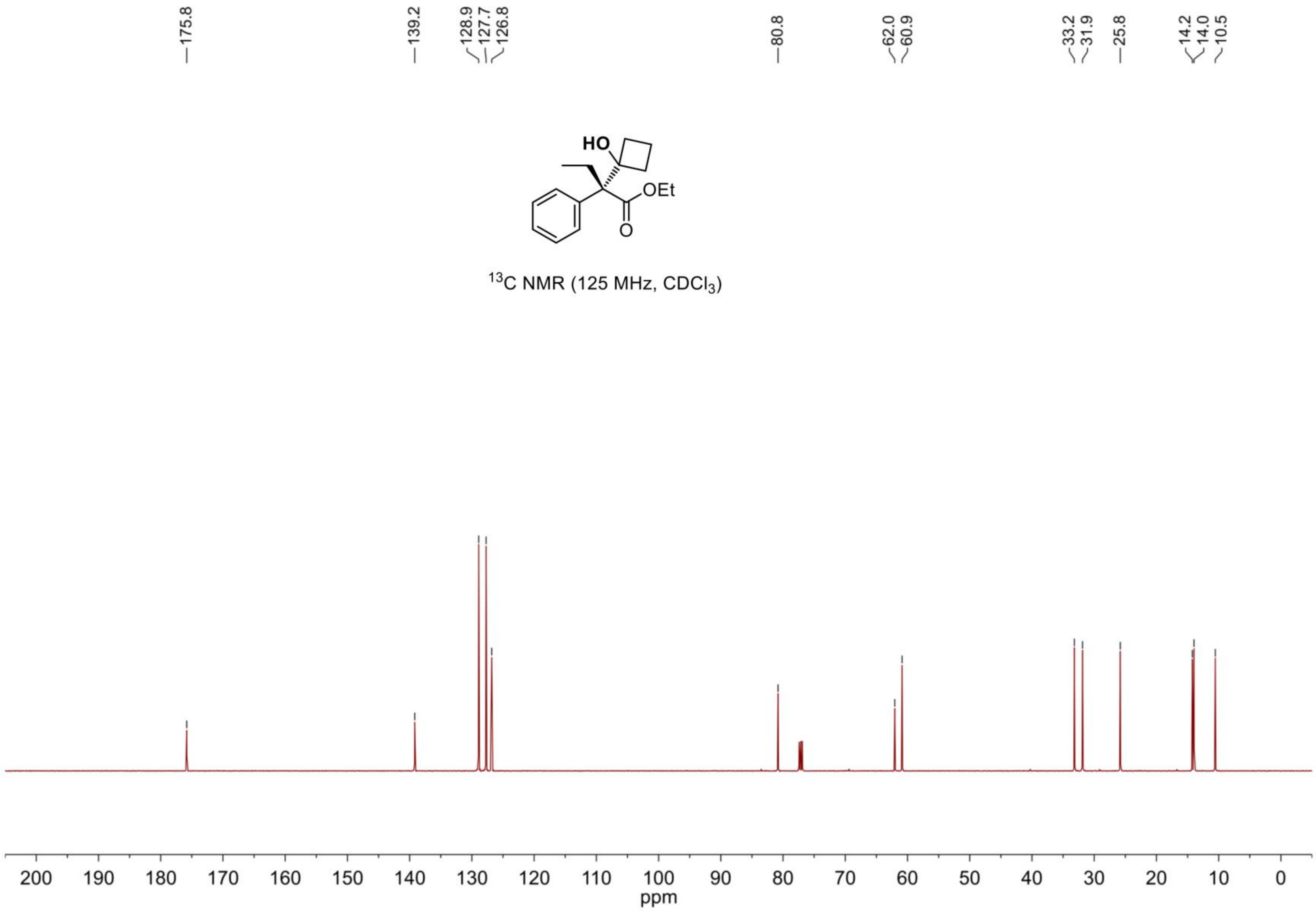

Go back to table of contents

Ethyl 2-(3-hydroxyoxetan-3-yl)-2-phenylbutanoate (84)

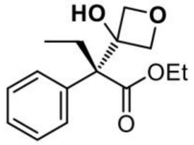

^{1}H NMR (500 MHz, $CDCl_3$)

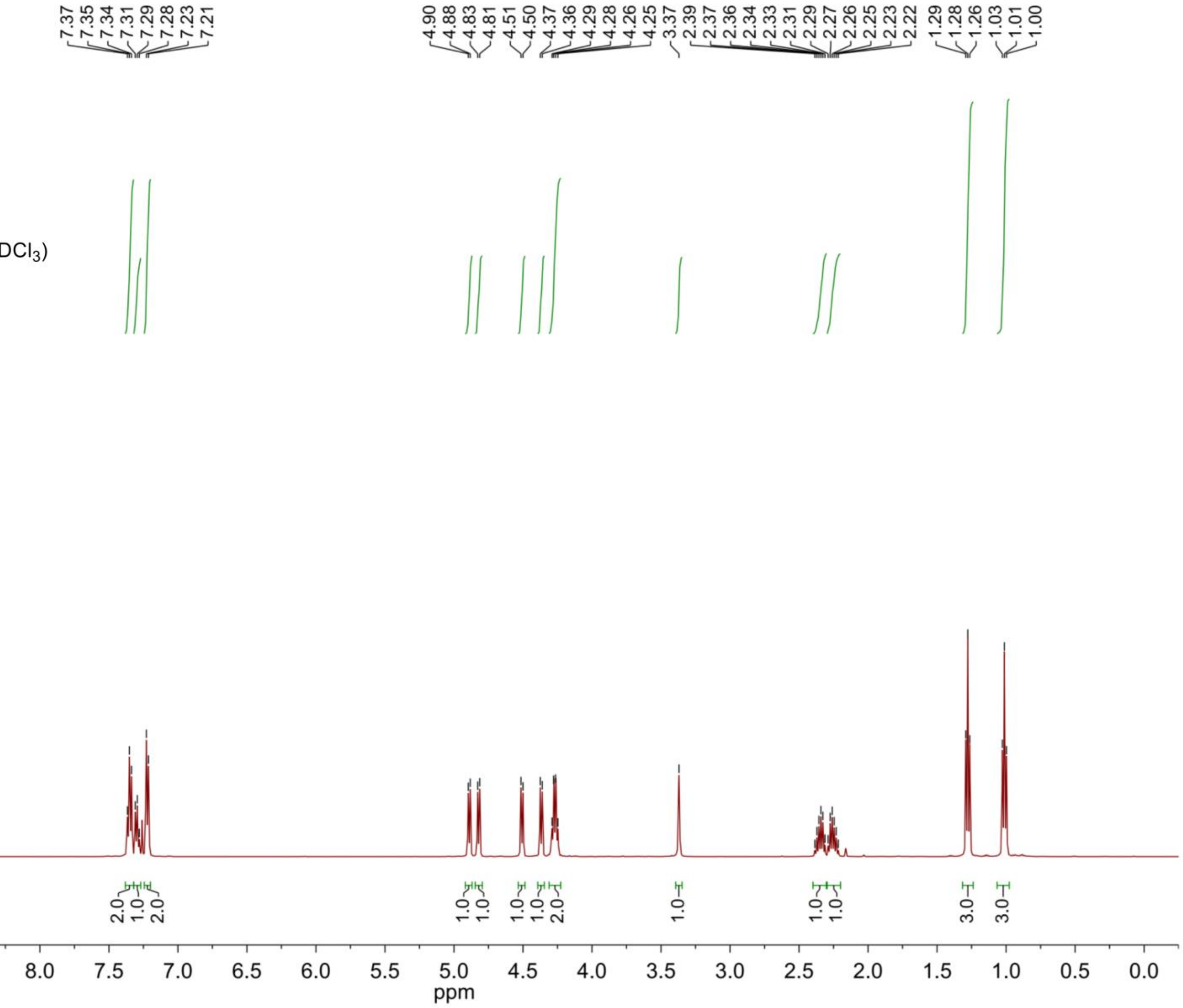

Ethyl 2-(3-hydroxyoxetan-3-yl)-2-phenylbutanoate (84)

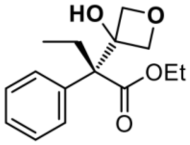

^{13}C NMR (125 MHz, $CDCl_3$)

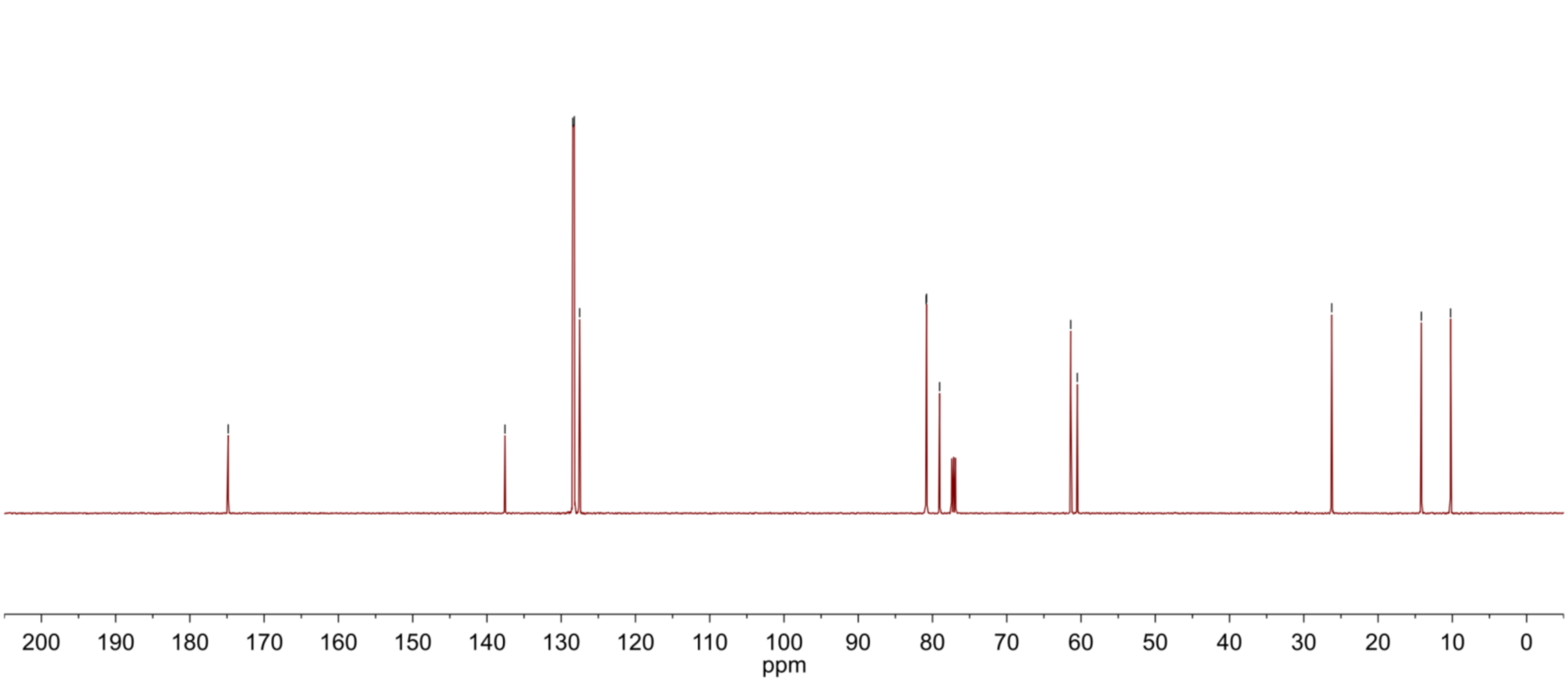

Go back to table of contents

tert-Butyl 3-(1-ethoxy-1-oxo-2-phenylbutan-2-yl)-3-hydroxyazetidine-1-carboxylate (85)

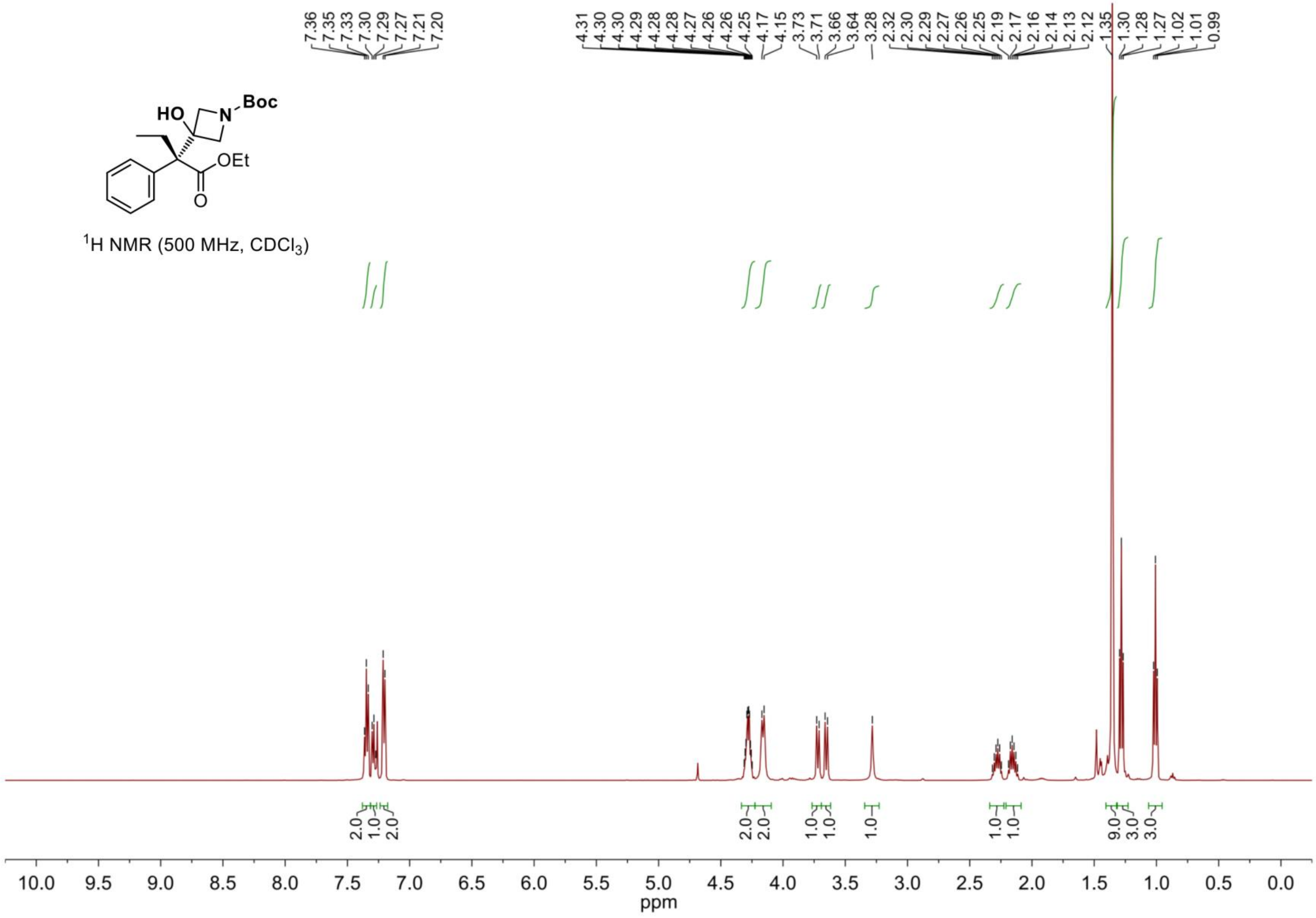

Go back to table of contents

tert-Butyl 3-(1-ethoxy-1-oxo-2-phenylbutan-2-yl)-3-hydroxyazetidine-1-carboxylate (85)

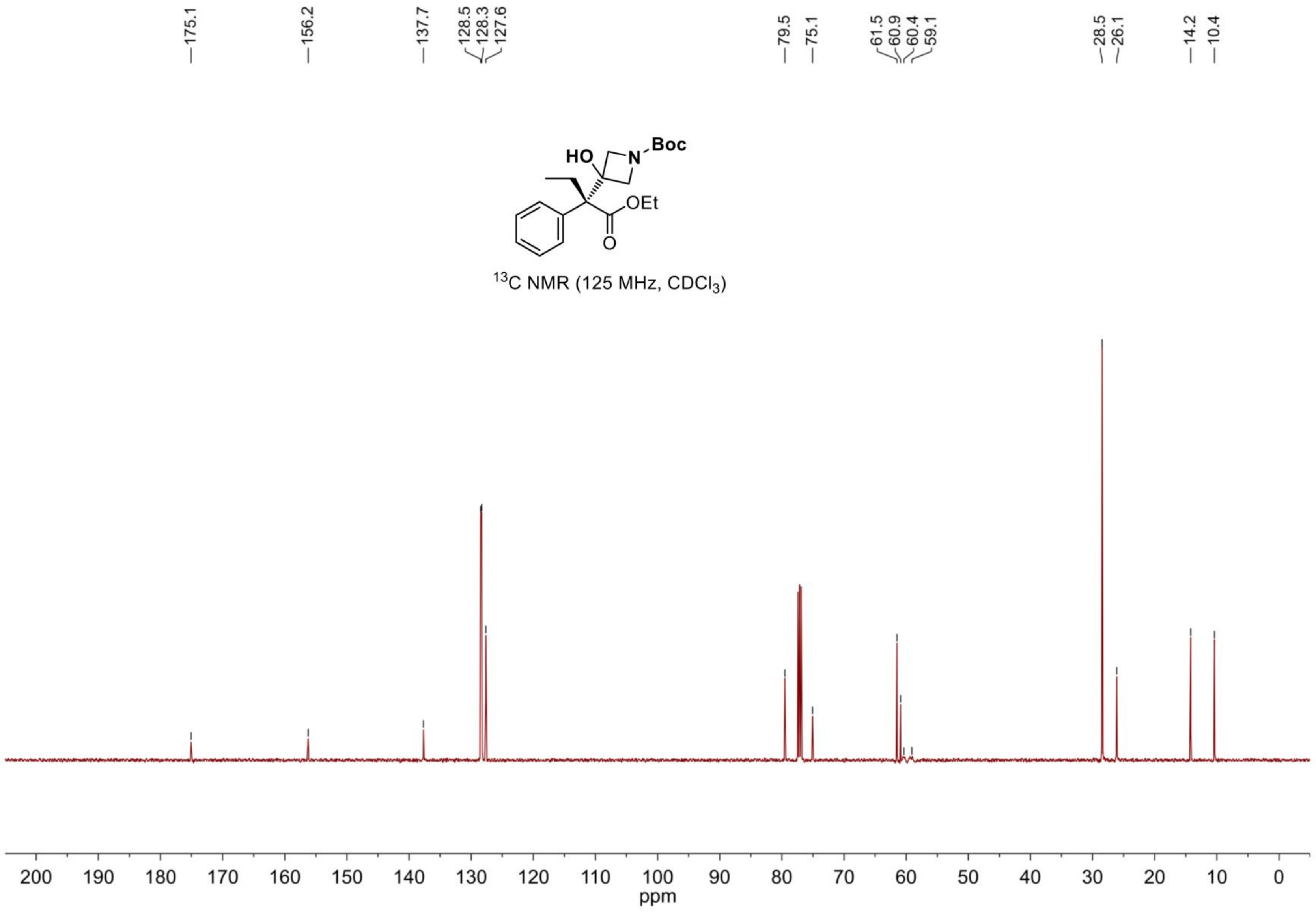

Go back to table of contents

Ethyl 2-ethyl-3-oxo-2-phenylbutanoate (86)

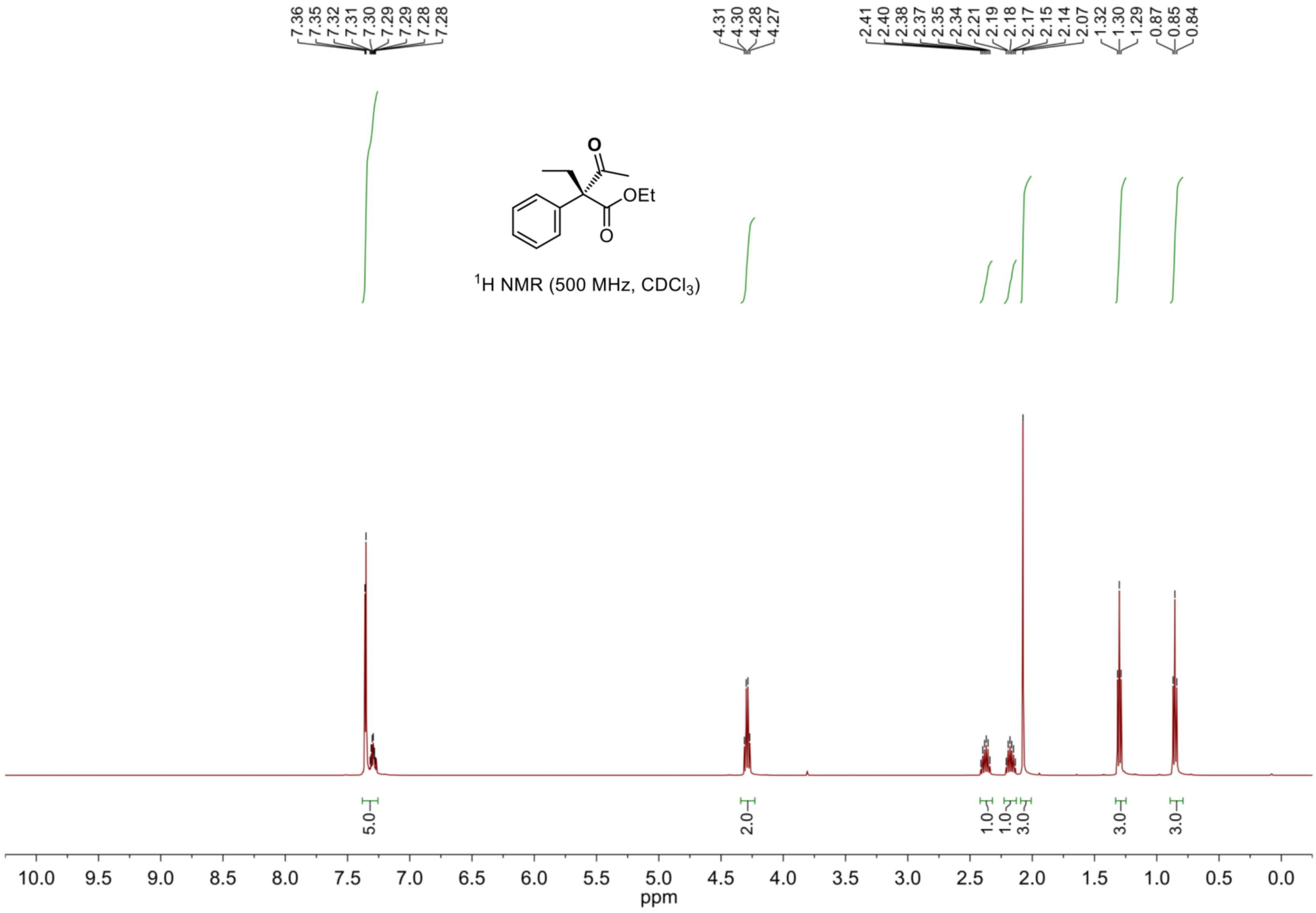

Go back to table of contents

Ethyl 2-ethyl-3-oxo-2-phenylbutanoate (86)

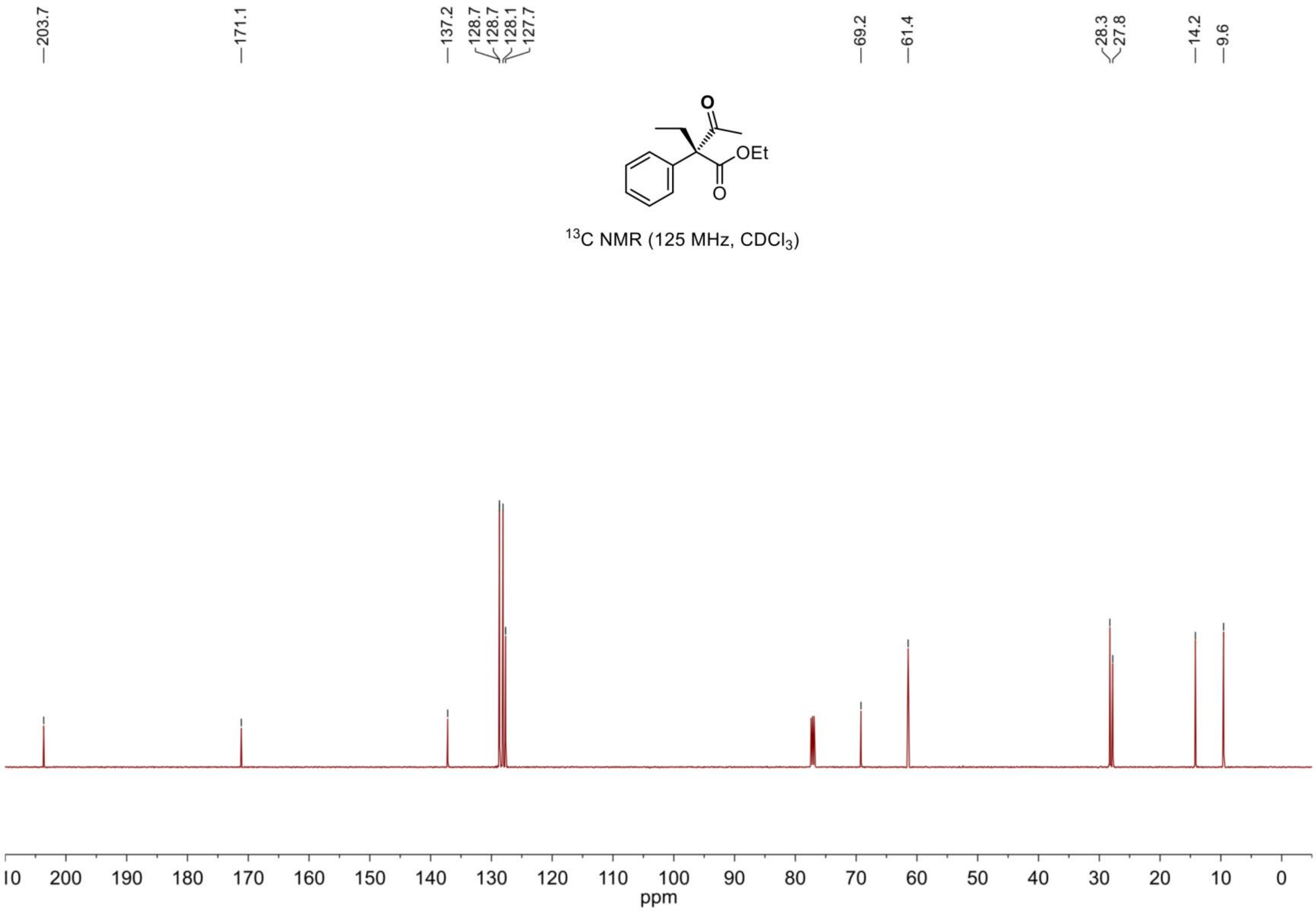

Go back to table of contents

Ethyl 2-ethyl-3-oxo-2-phenylpentanoate (87)

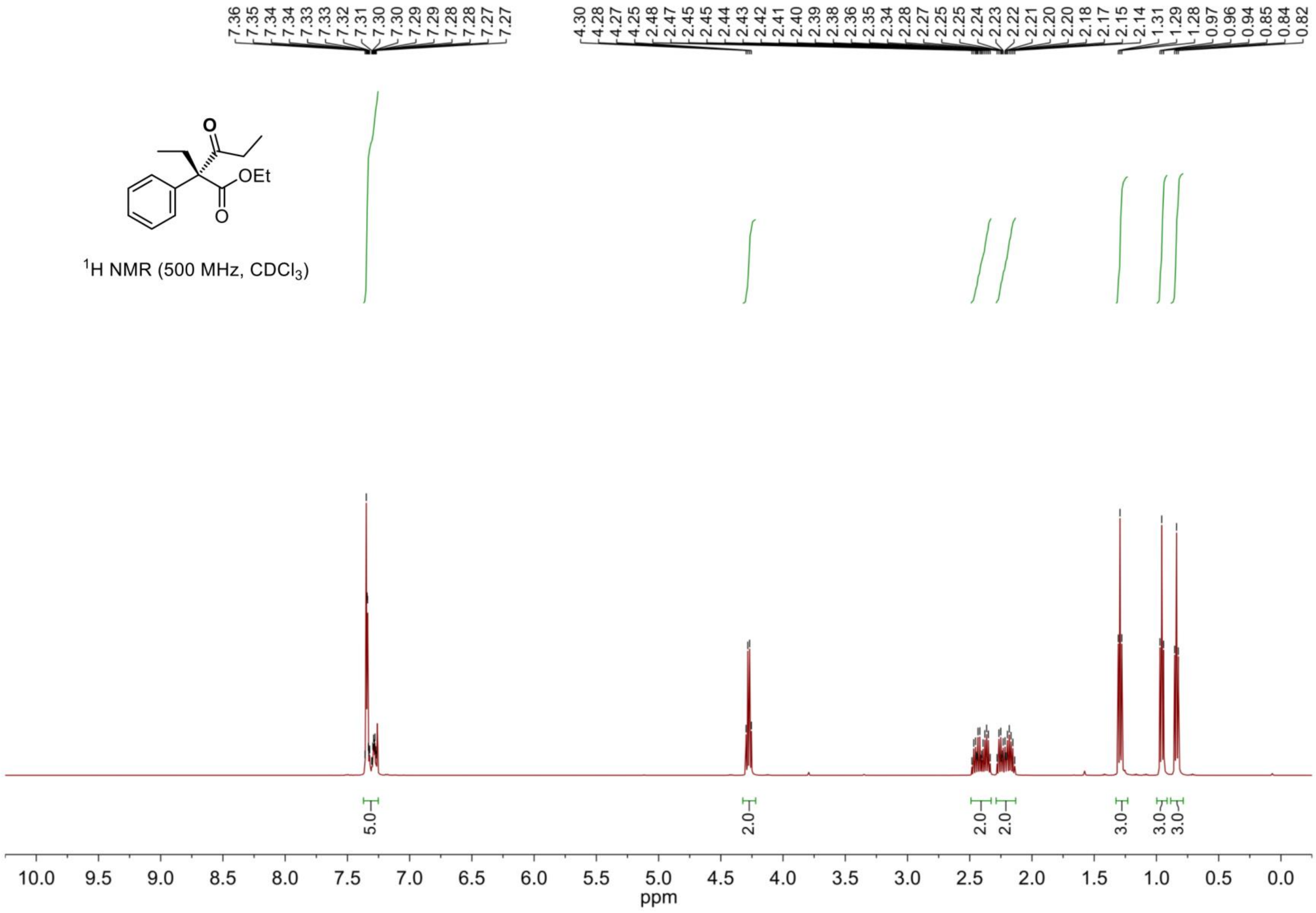

Go back to table of contents

Ethyl 2-ethyl-3-oxo-2-phenylpentanoate (87)

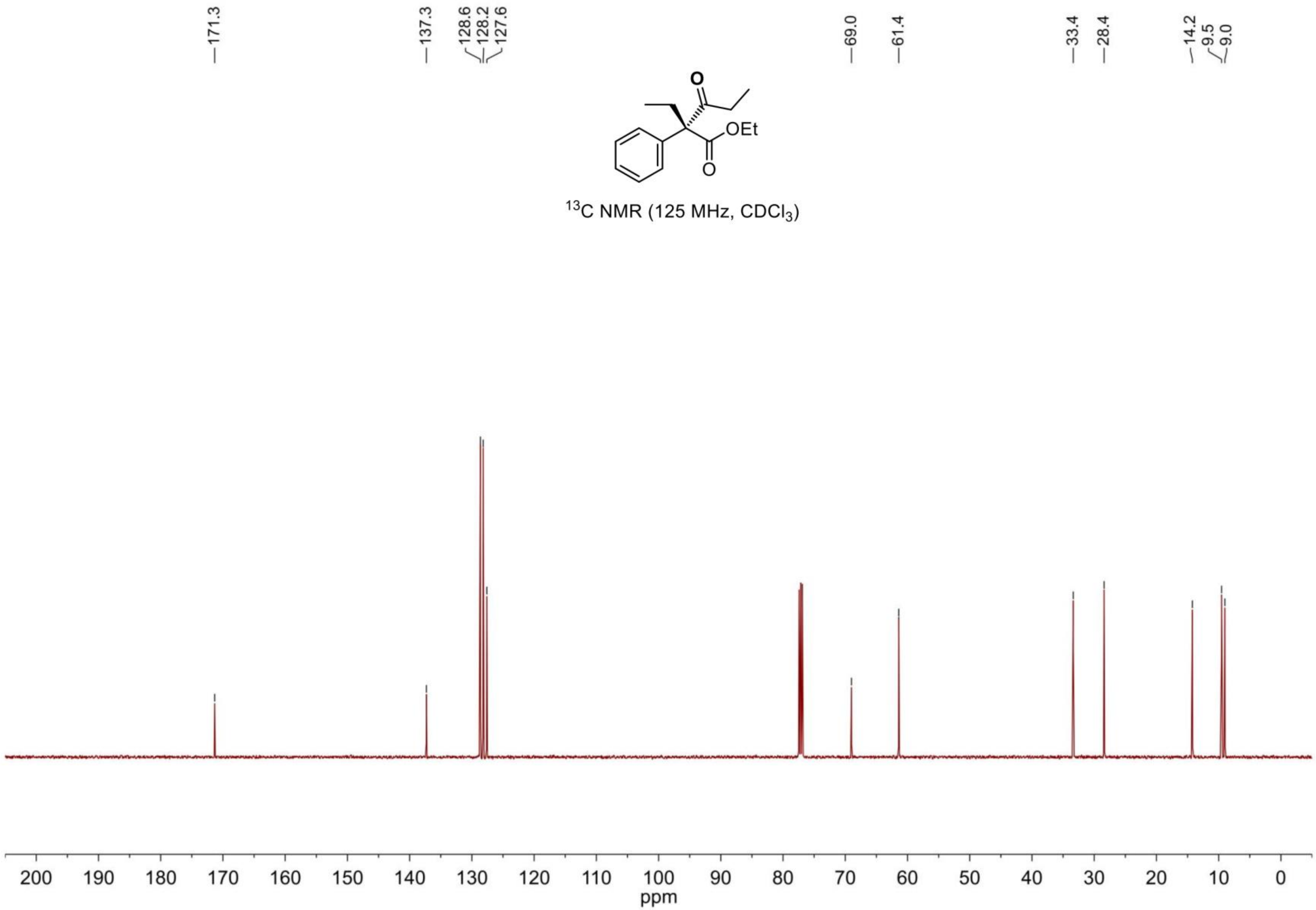

Go back to table of contents

Ethyl 2-ethyl-3-oxo-2,4-diphenylbutanoate (88)

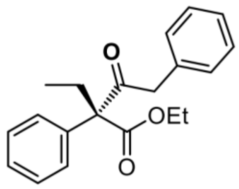

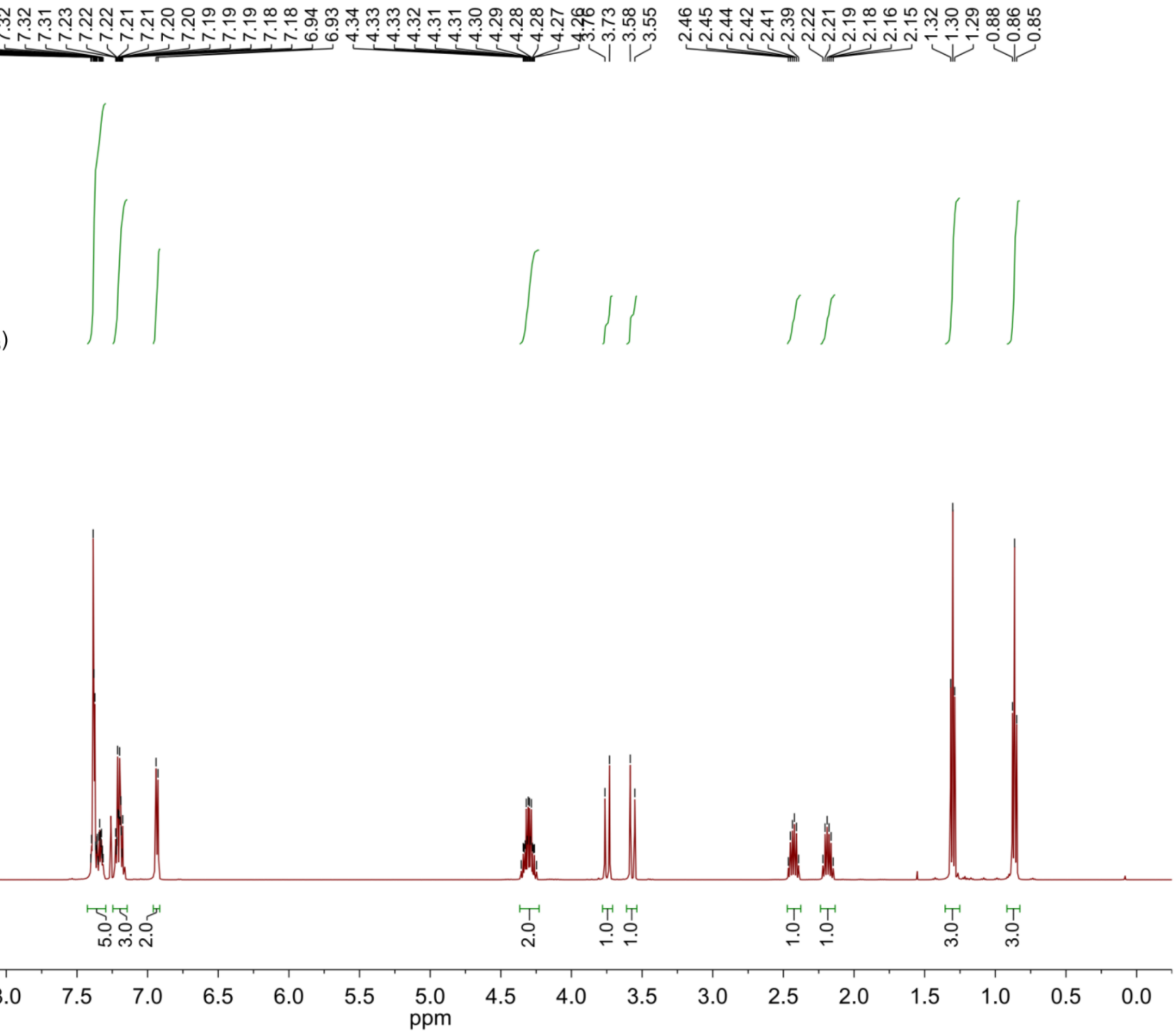

^{1}H NMR (500 MHz, $CDCl_3$)

Go back to table of contents

Ethyl 2-ethyl-3-oxo-2,4-diphenylbutanoate (88)

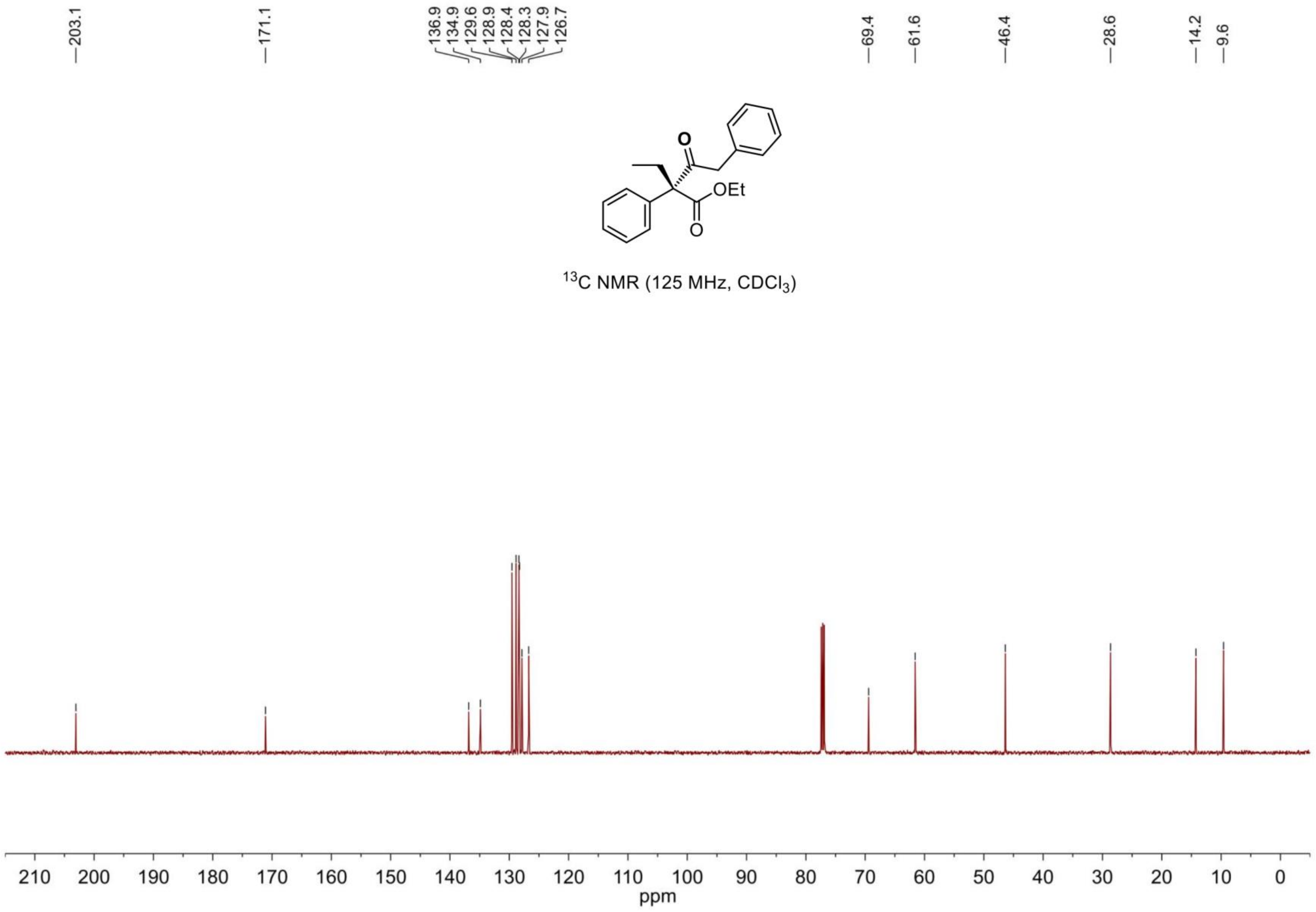

Go back to table of contents

Ethyl 4-(2-chlorophenyl)-2-ethyl-2-(3-fluoro-4-methoxyphenyl)-3-oxobutanoate (89)

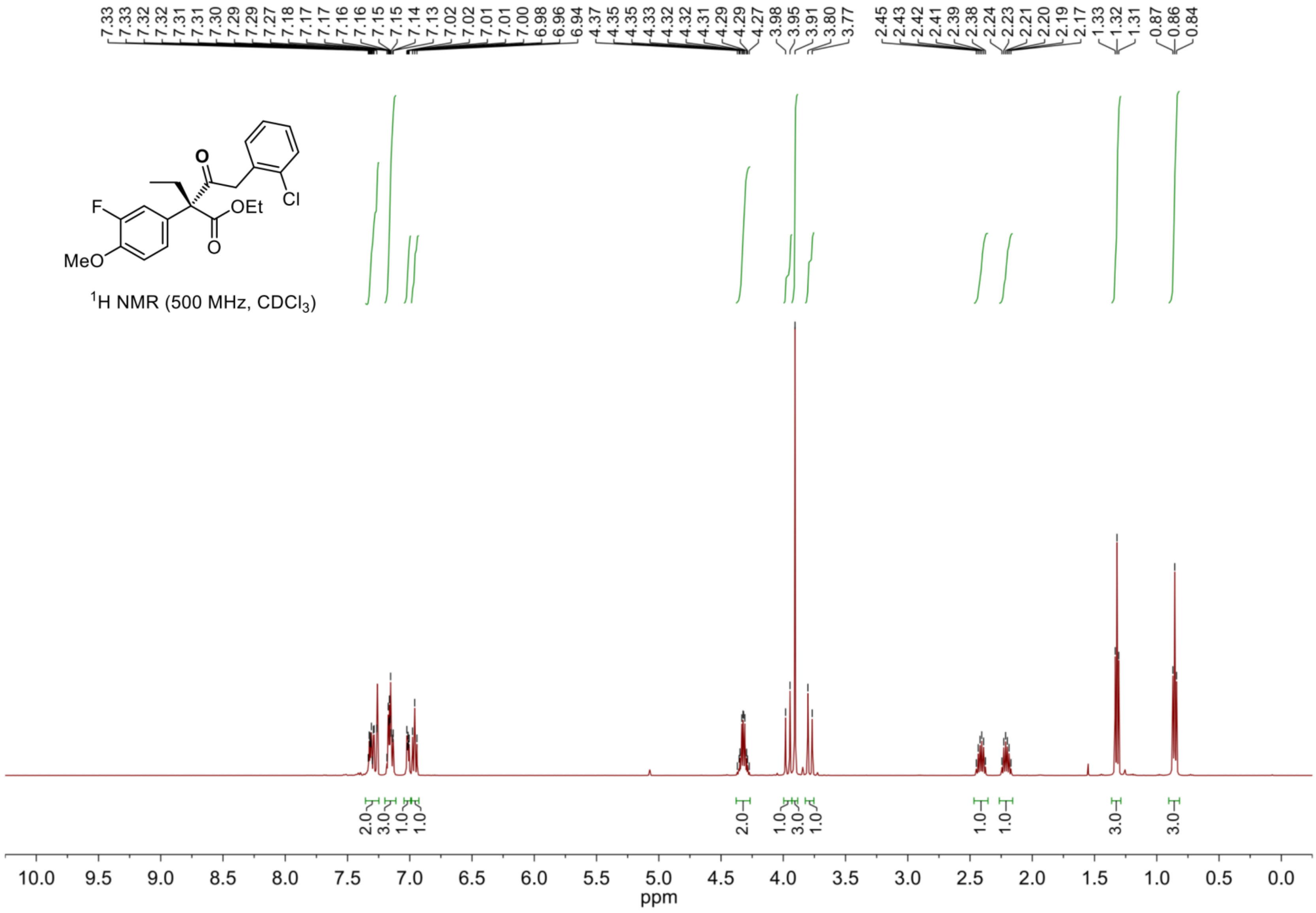

Go back to table of contents

Ethyl 4-(2-chlorophenyl)-2-ethyl-2-(3-fluoro-4-methoxyphenyl)-3-oxobutanoate (89)

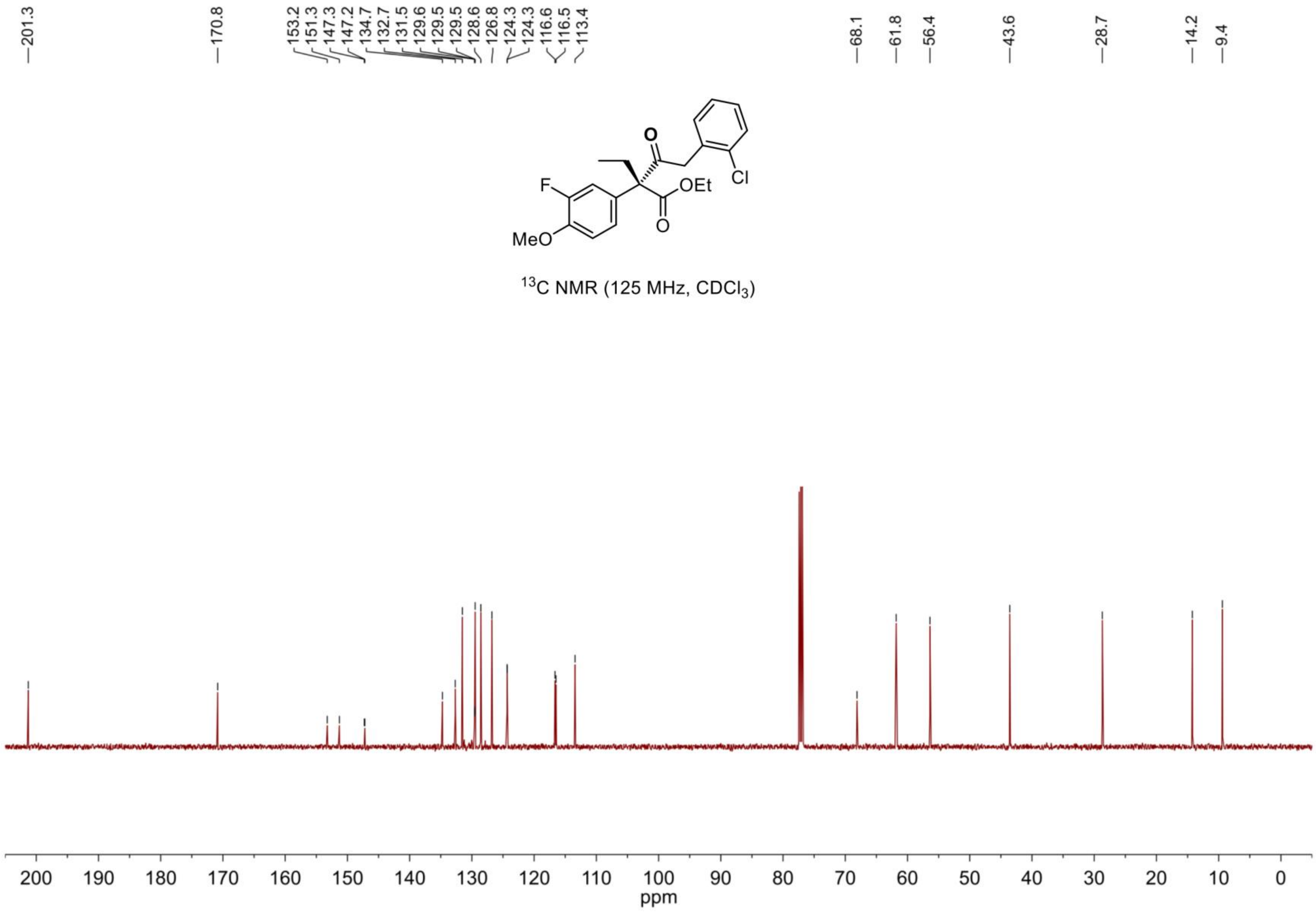

Go back to table of contents

Ethyl 2-benzoyl-2-(4-methoxyphenyl)butanoate (90)

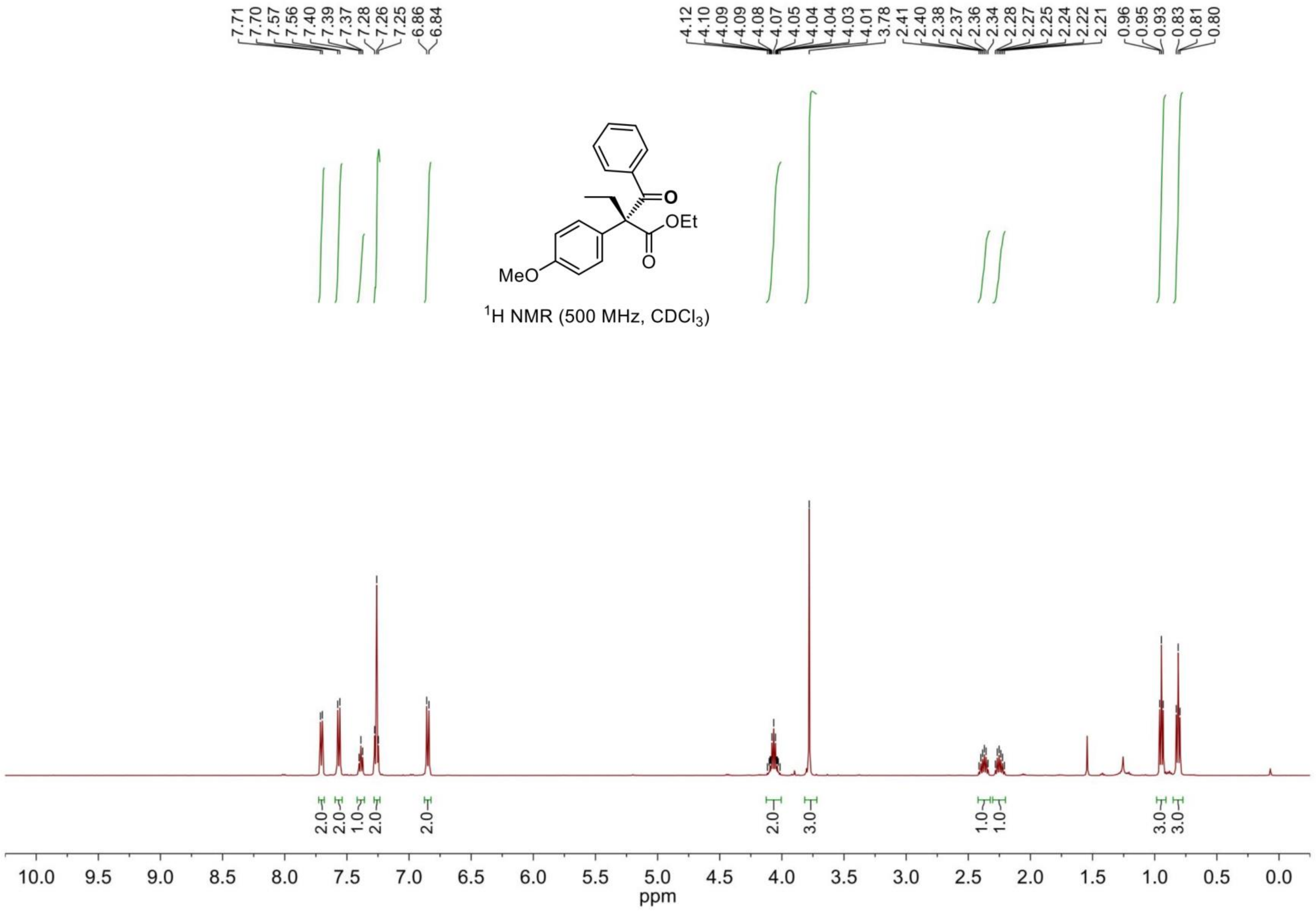

Go back to table of contents

Ethyl 2-benzoyl-2-(4-methoxyphenyl)butanoate (90)

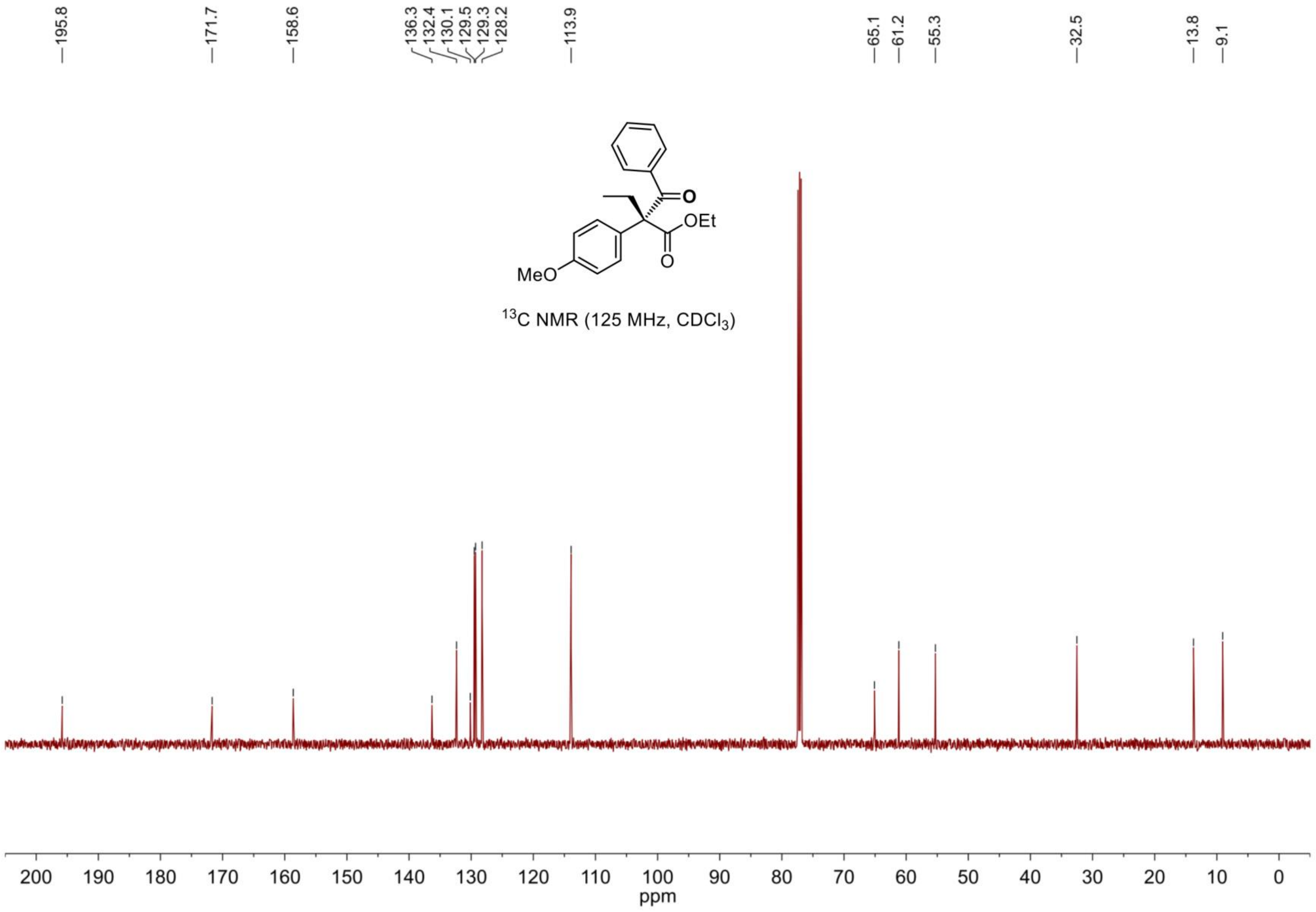

Go back to table of contents

Ethyl 2-(3,5-bis(trifluoromethyl)benzoyl)-2-(3-fluoro-4-methoxyphenyl)butanoate (91)

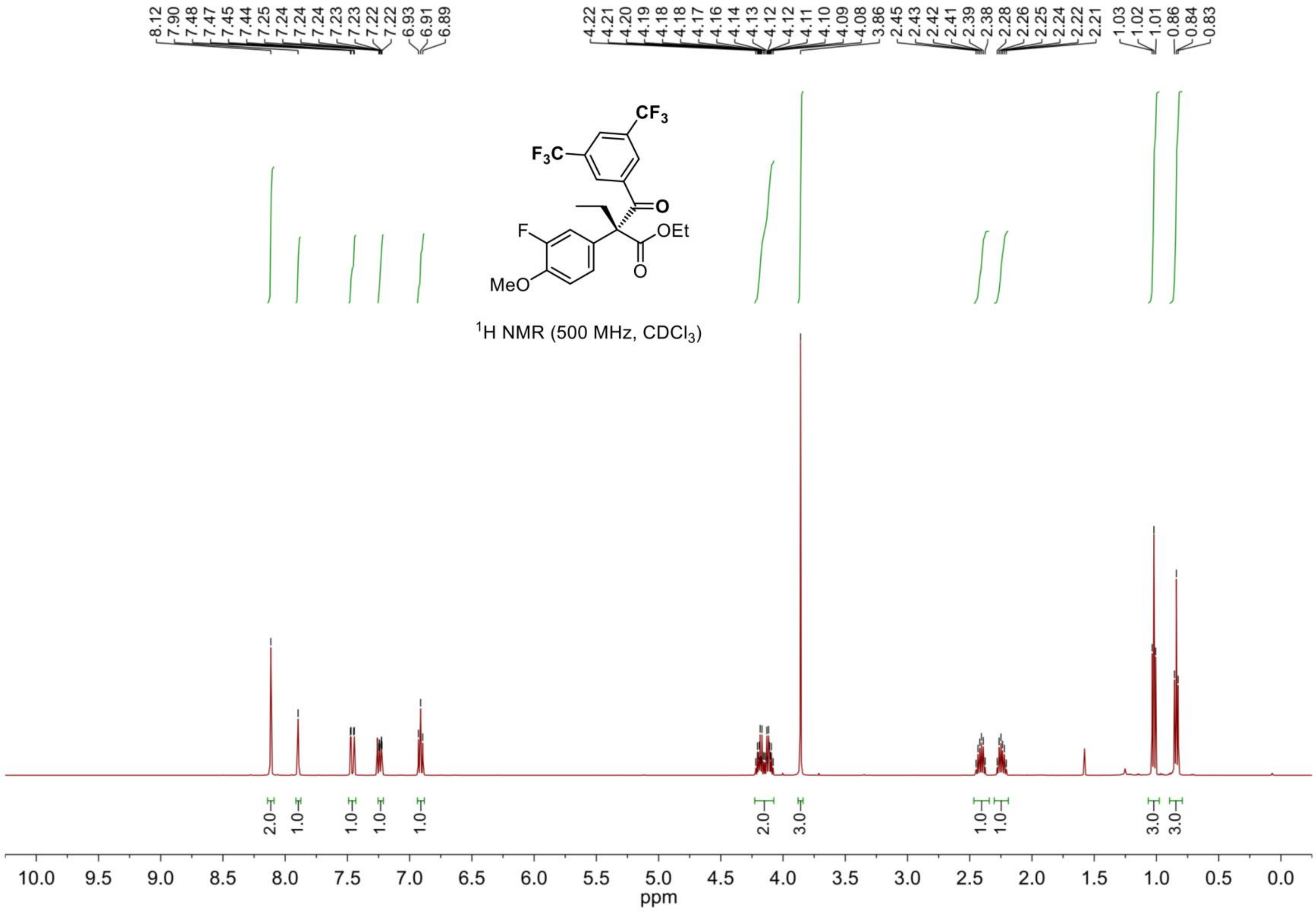

Go back to table of contents

Ethyl 2-(3,5-bis(trifluoromethyl)benzoyl)-2-(3-fluoro-4-methoxyphenyl)butanoate (91)

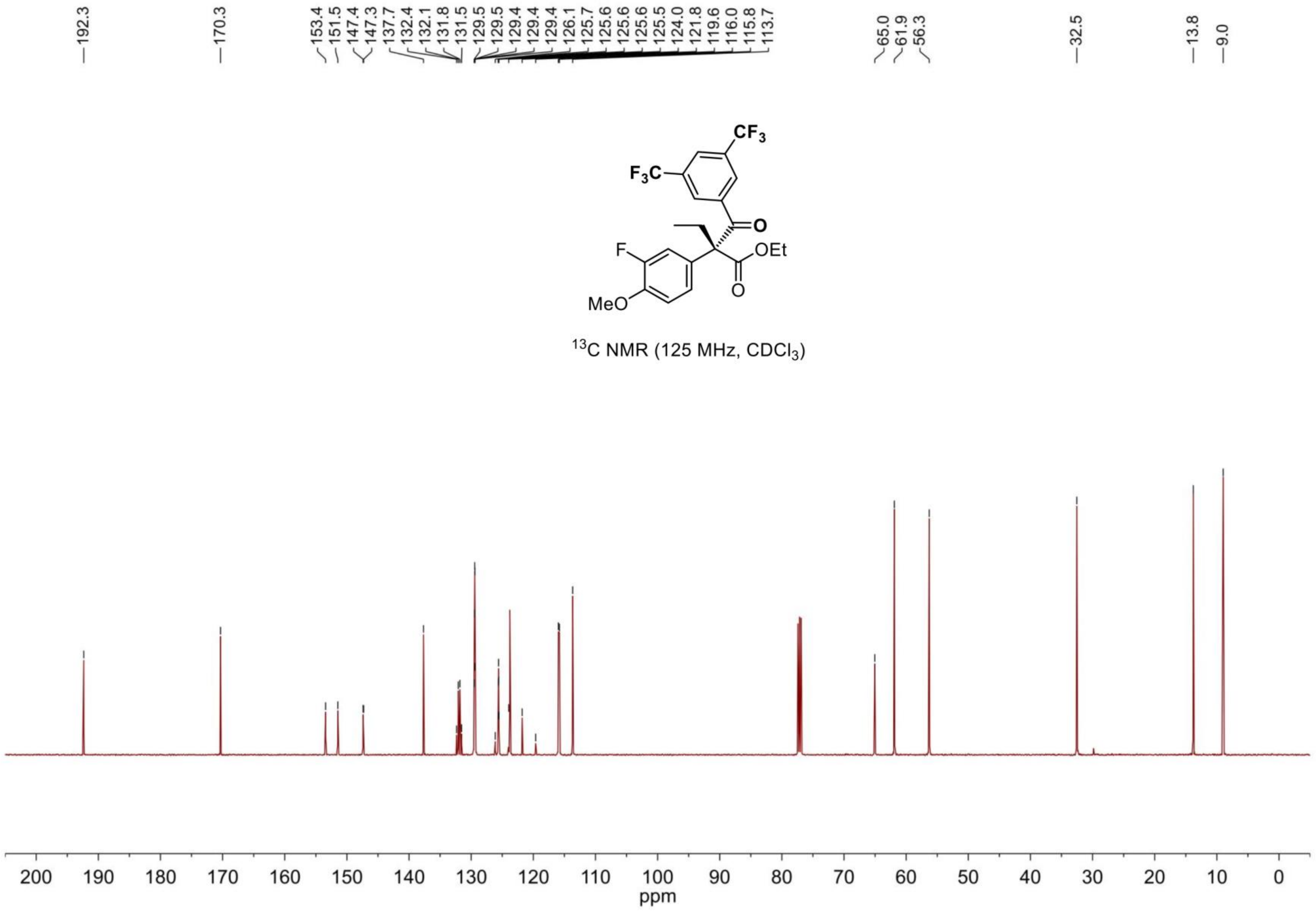

Go back to table of contents

2-Ethyl-2-phenylpent-4-enoic acid (93)

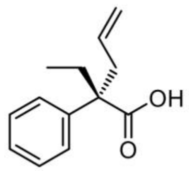

^{1}H NMR (500 MHz, $CDCl_3$)

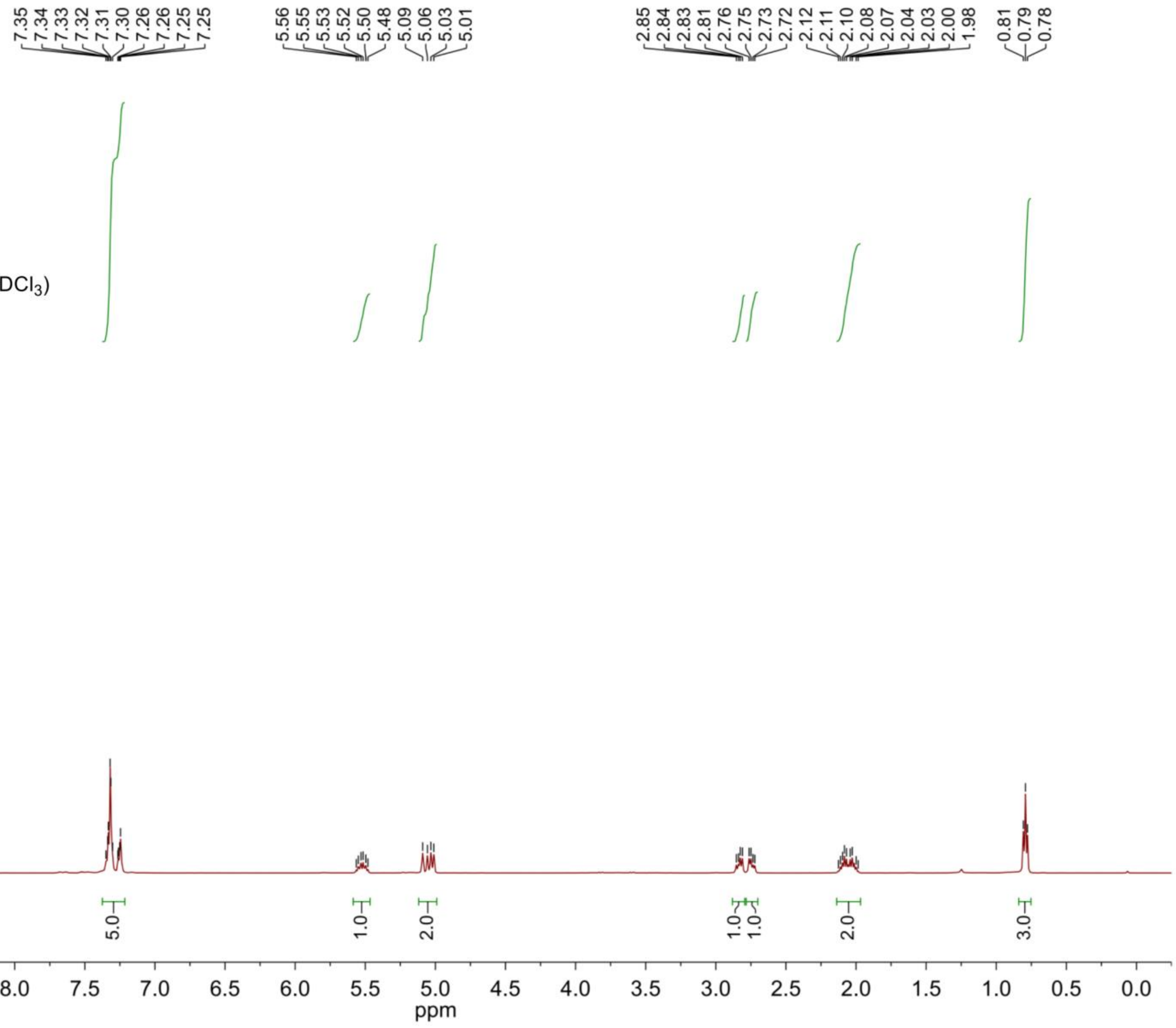

Go back to table of contents

2-Ethyl-2-phenylpent-4-enoic acid (93)

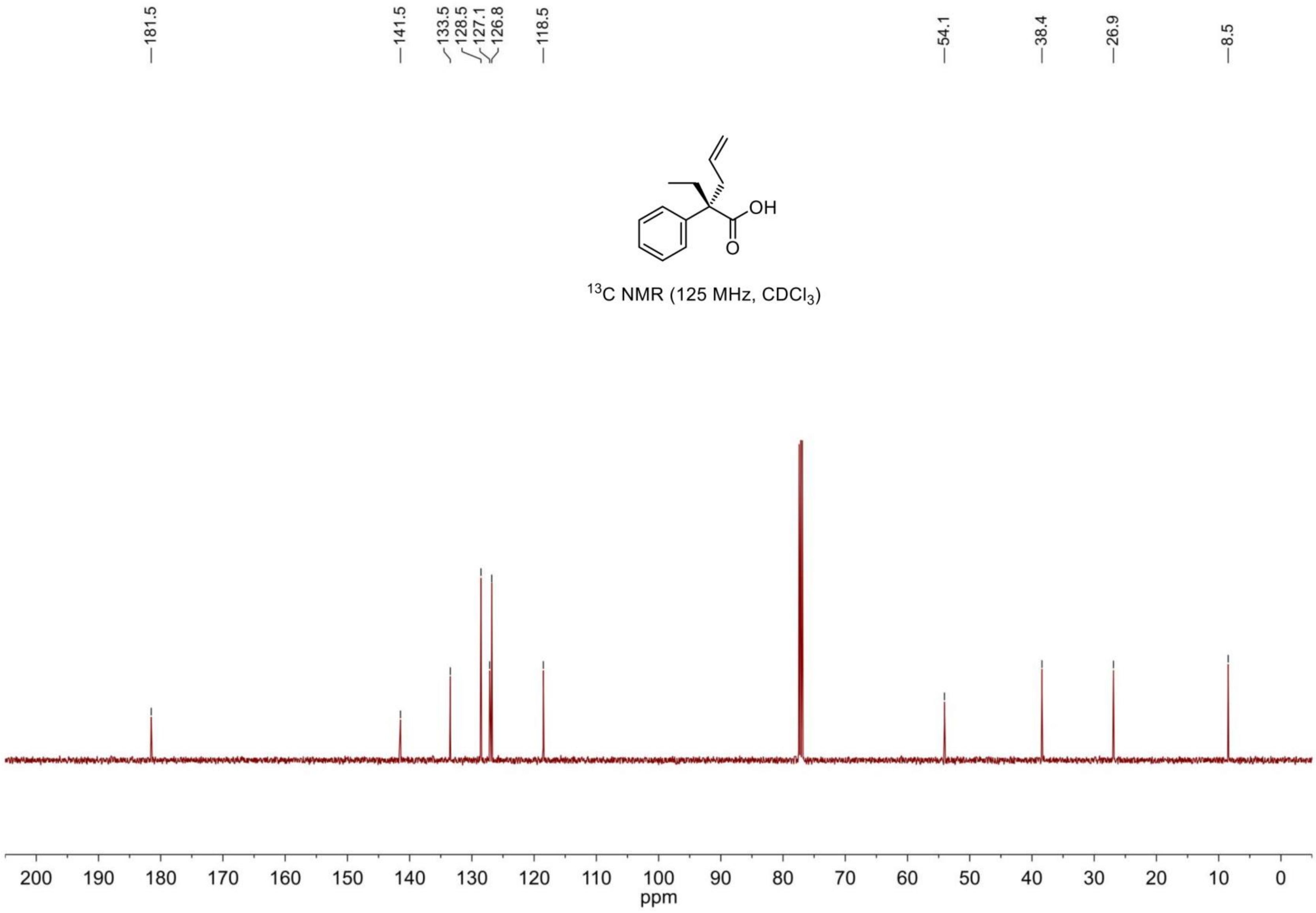

Go back to table of contents

2-Ethyl-3,3-dimethyl-2-phenylpent-4-enoic acid (94)

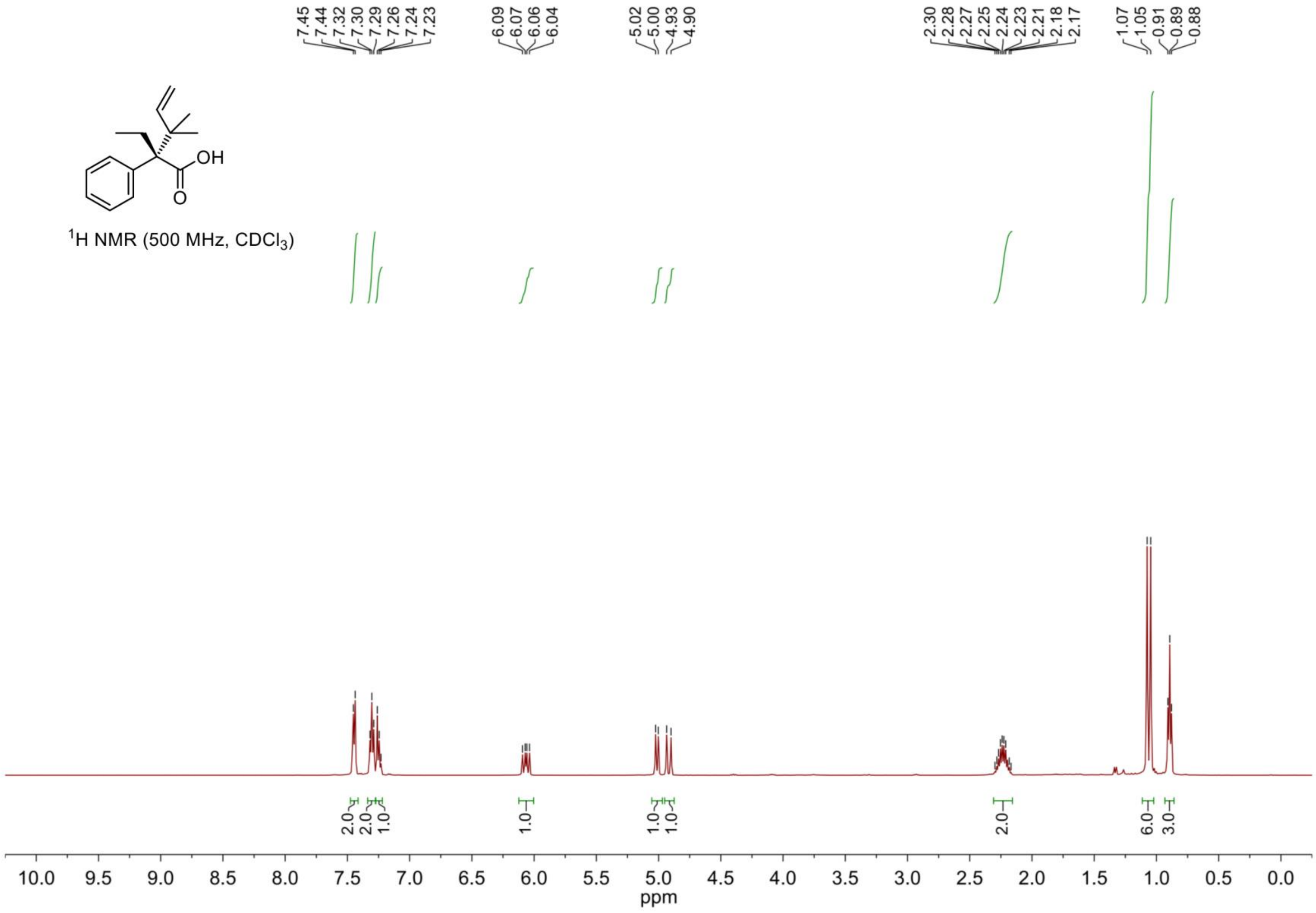

Go back to table of contents

2-Ethyl-3,3-dimethyl-2-phenylpent-4-enoic acid (94)

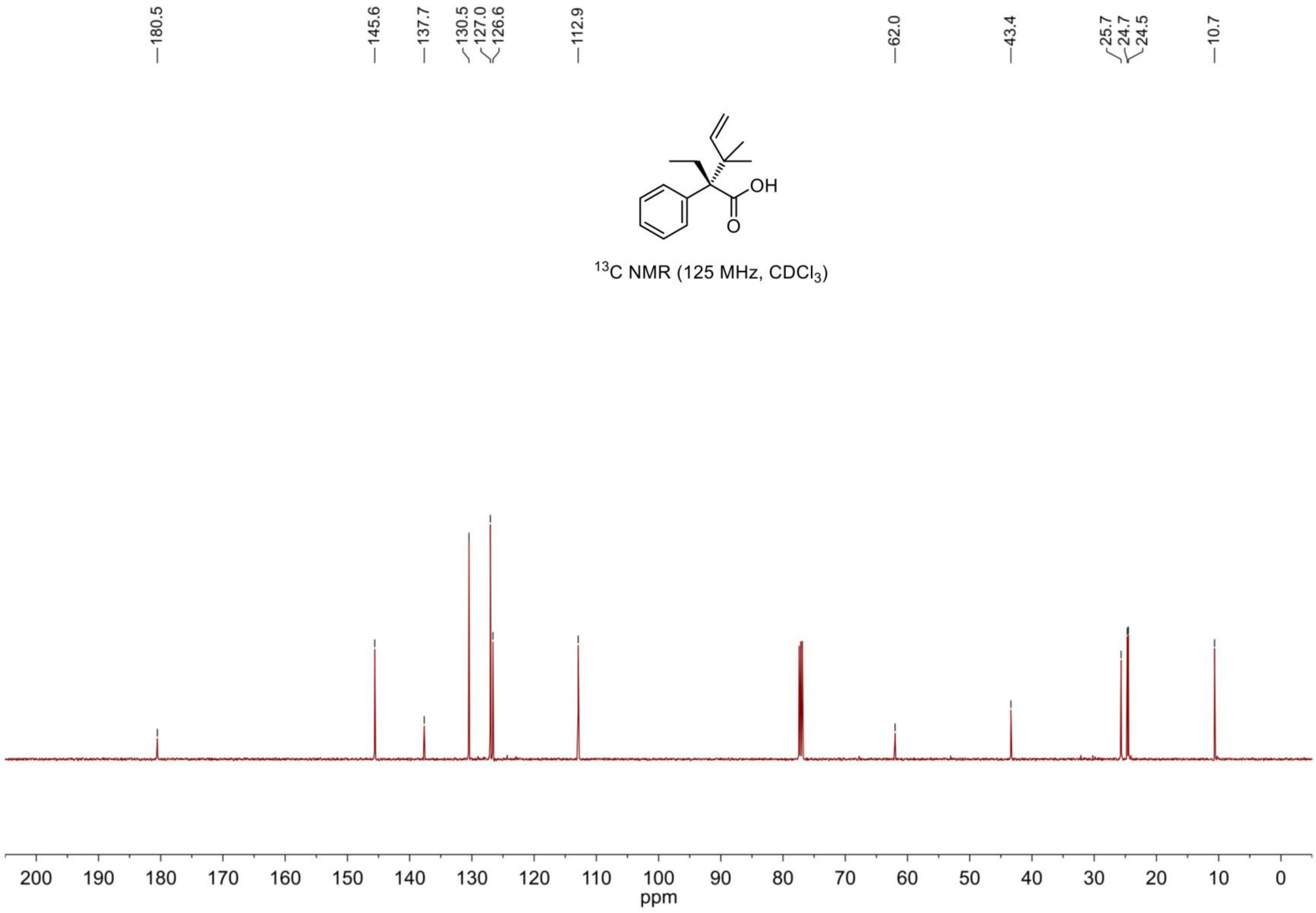

Go back to table of contents

2-Ethyl-2-phenylpenta-3,4-dienoic acid (95)

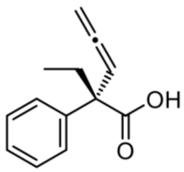

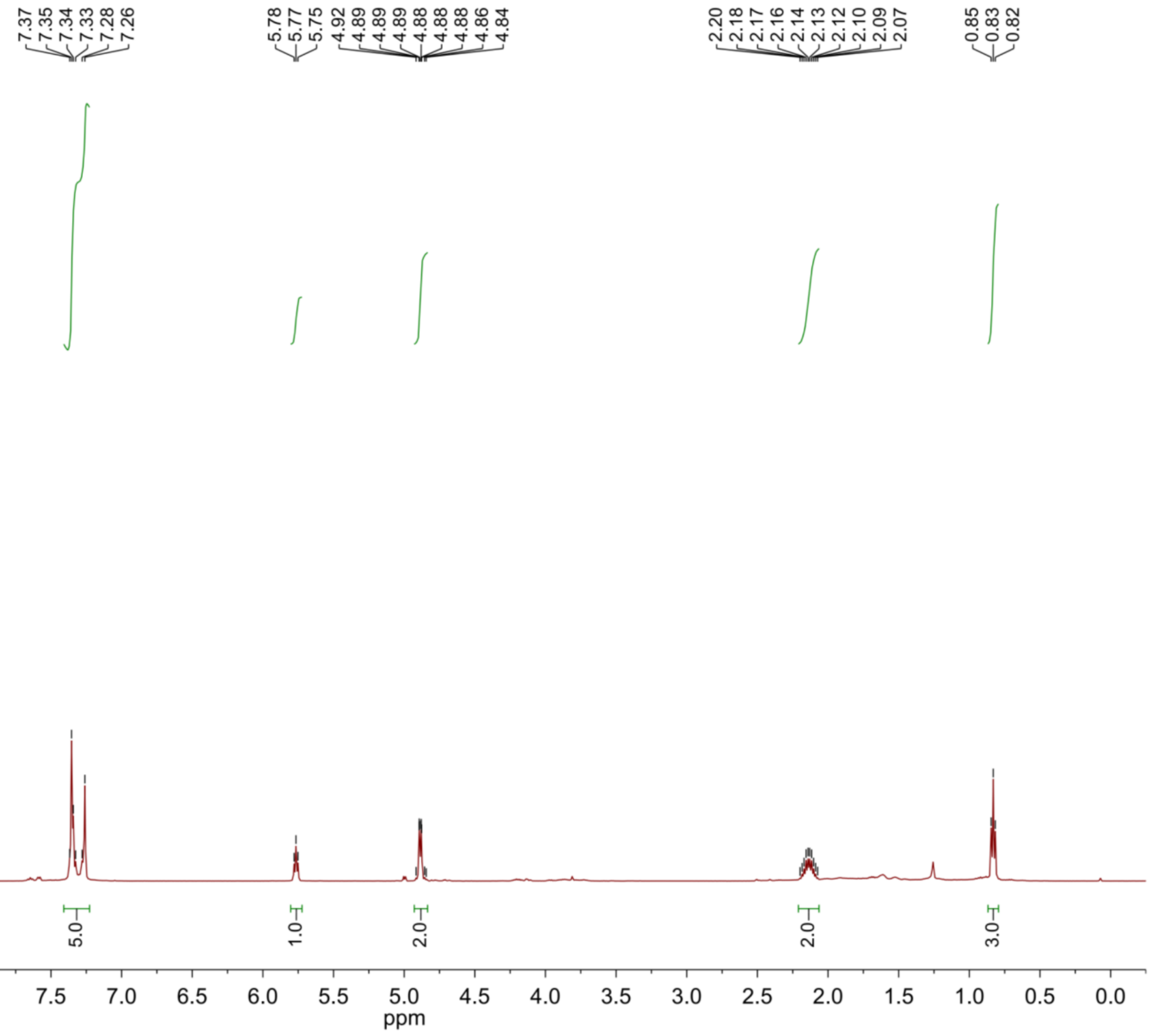

Go back to table of contents

2-Ethyl-2-phenylpenta-3,4-dienoic acid (95)

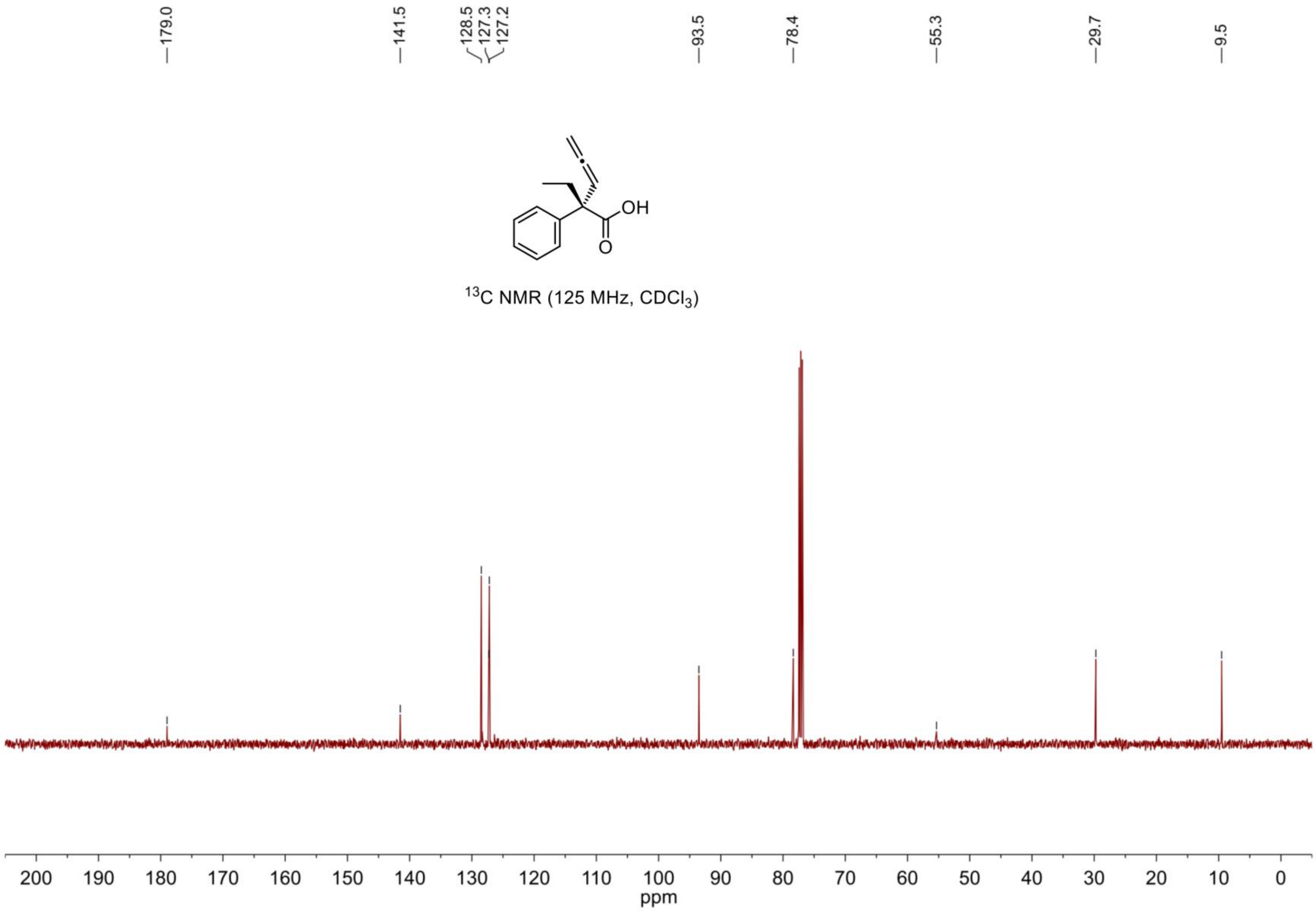

2-Ethyl-3-methylene-2-phenylpent-4-enoic acid (96)

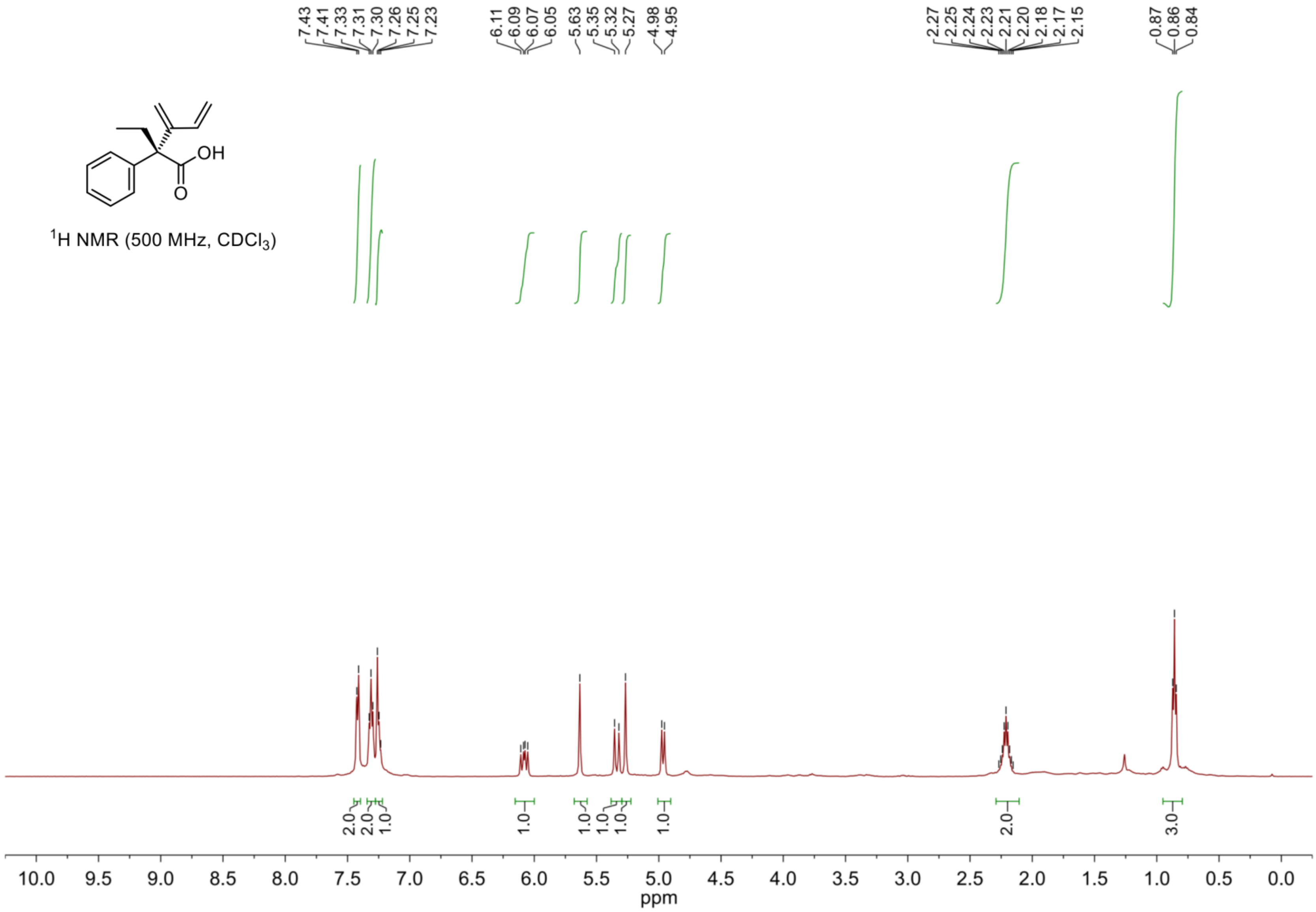

Go back to table of contents

2-Ethyl-3-methylene-2-phenylpent-4-enoic acid (96)

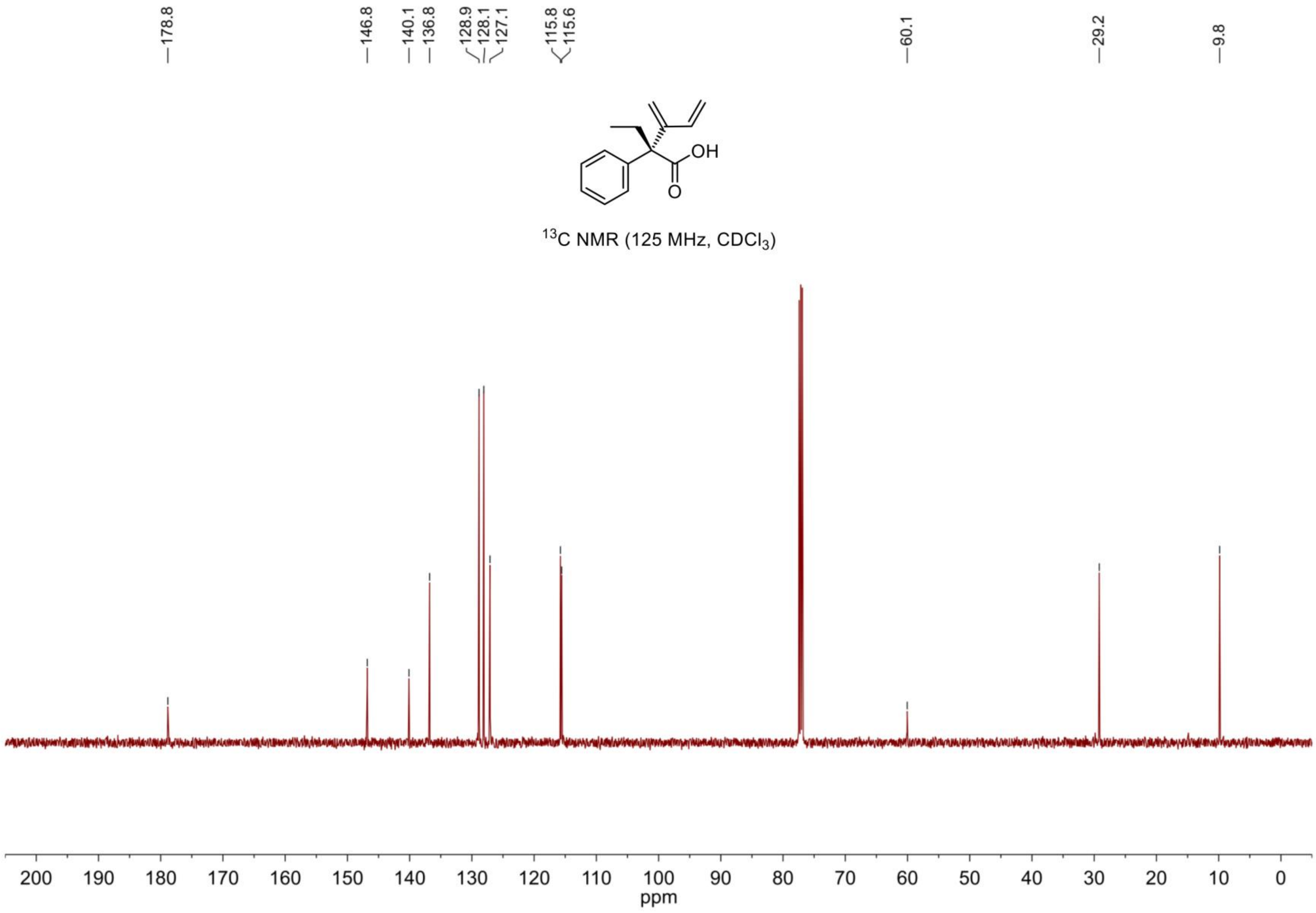

Go back to table of contents

Ethyl 2-ethyl-2-phenylpent-4-enoate (97)

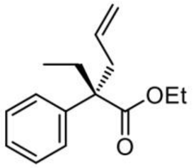

^{1}H NMR (500 MHz, $CDCl_3$)

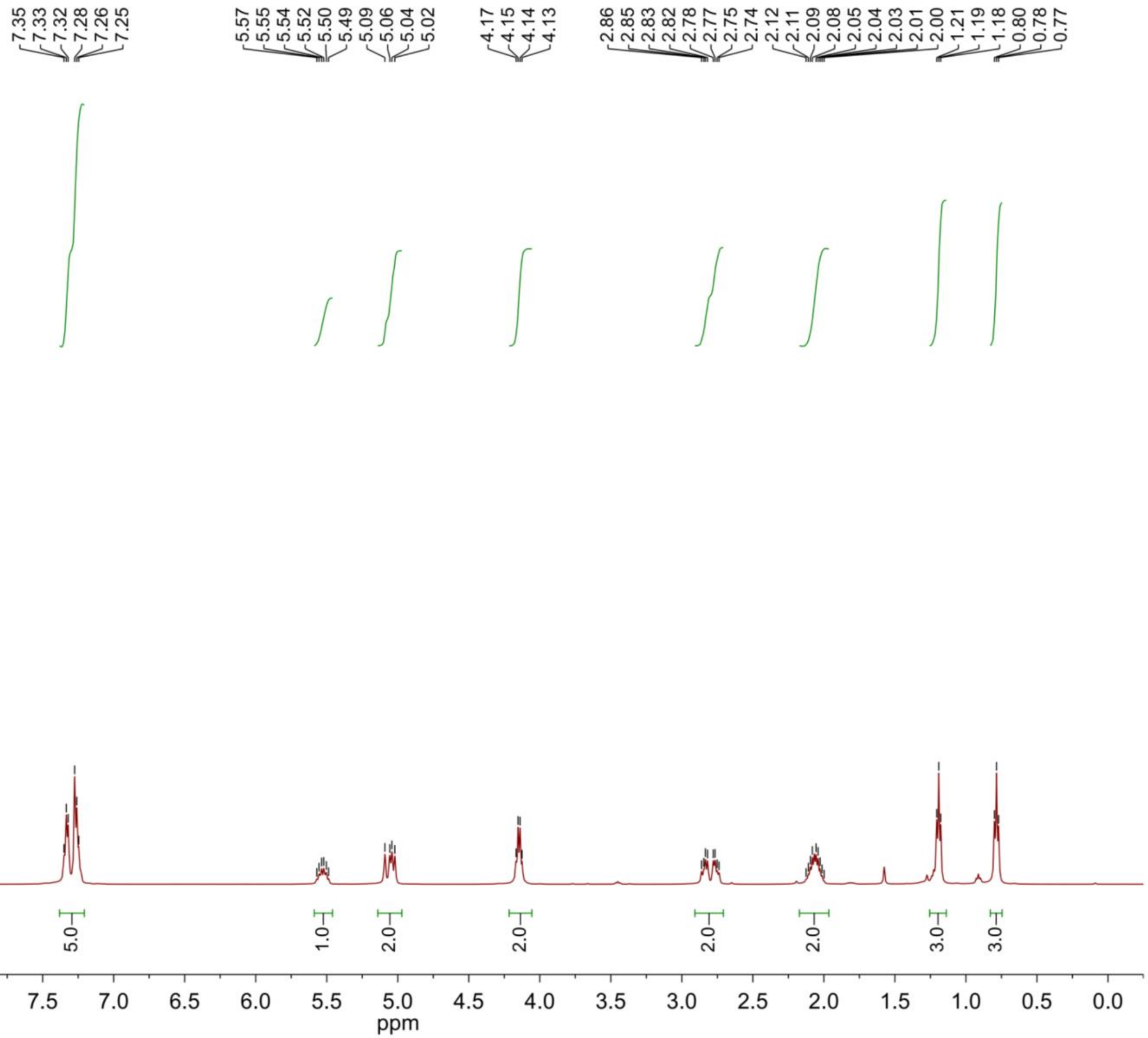

Go back to table of contents

Ethyl 2-ethyl-2-phenylpent-4-enoate (97)

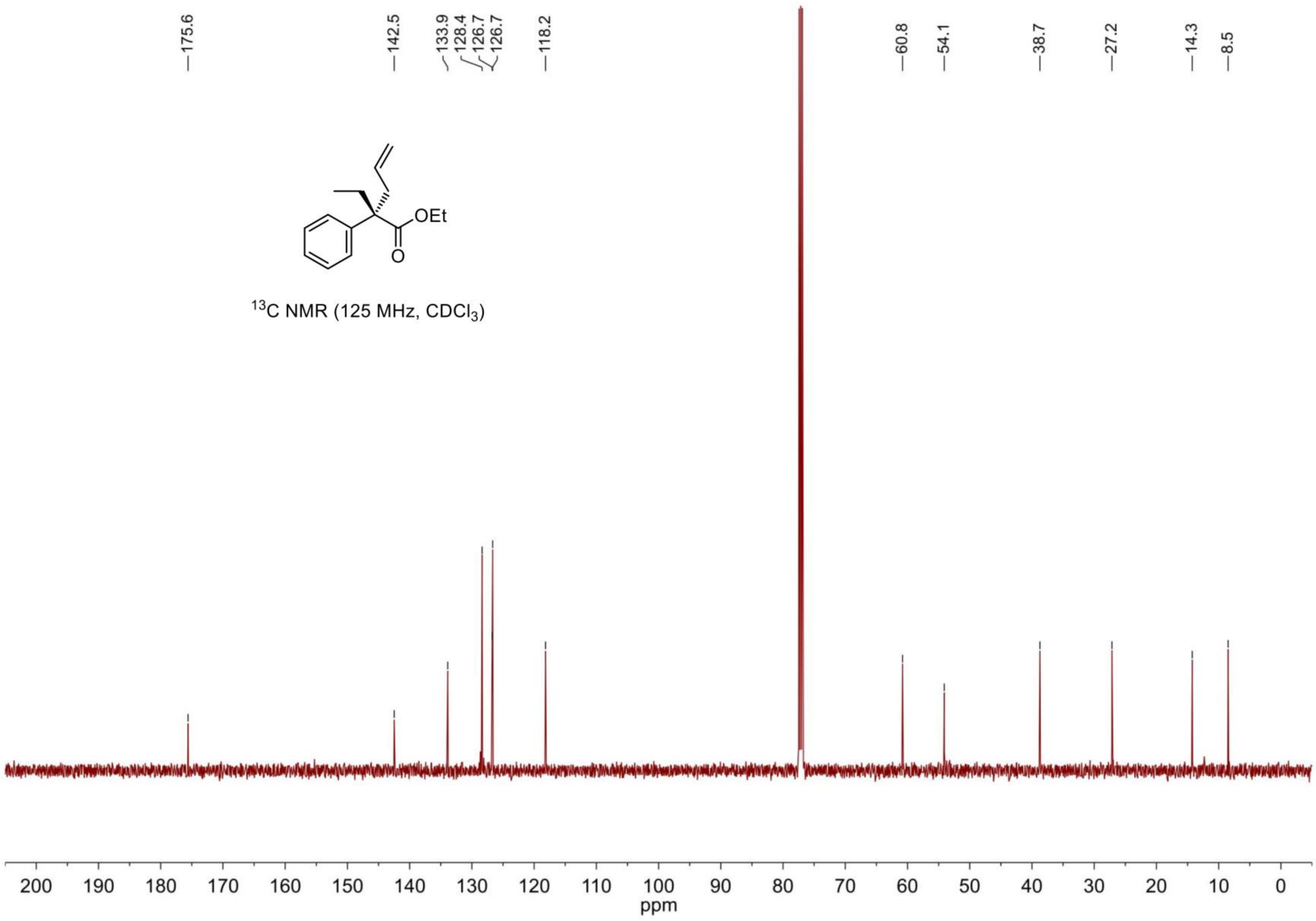

Go back to table of contents

References

[1] Ianni, A.; Waldvogel, S. R. Reliable and Versatile Synthesis of 2-Aryl-substituted Cinnamic Acid Esters. *Synthesis*. **2006**, *13*, 2103–2112.

[2] Shimada, T.; Kobayashi, Y.; Saigo, K. Synthesis of Enantiopure 6-Methoxy-2-naphthylglycolic Acid and Its Application as a Resolving Agent. *Tetrahedron: Asymmetry* **2005**, 16, 3807–3813.

[3] Thai, K.; Langdon, S. M.; Bilodeau, F.; Gravel, M. Highly Chemo-and Enantioselective Cross-Benzoin Reaction of Aliphatic Aldehydes and α-Ketoesters. *Org. Lett.* **2013**, *15*, 2214–2217.

[4] Deslandes, S.; Lamoral–Theys, D.; Frongia, C. Synthesis and Biological Evaluation of Analogs of the Marine Alkaloids Granulatimide and Isogranulatimide. *Eur. J. Med. Chem*. **2012**, *54*, 626–636.

[5] Rodriguez, N.; Manjolinho, F.; Grünberg, M. F.; Goossen, L. J. Synthesis of α, β-Unsaturated Ketones by Pd-Catalyzed Decarboxylative Allylation of α-Oxocarboxylates. *Chem. Eur. J.* **2011**, *17*, 13688–13691.

[6] Roytman, V. A.; Jin, S.; Nguyen, V. T.; Nguyen, V. D.; Haug, G. C.; Larionov, O. V.; Singleton, D. A. Bond Memory in Dynamically-Determined Stereoselectivity. *J. Am. Chem. Soc.* **2020**, *142*, 85–88.

[7] Horino, Y.; Murakami, M.; Aimono, A. Trialkylborane-Mediated Multicomponent Reaction for the Diastereoselective Synthesis of Anti-δ, δ-Disubstituted Homoallylic Alcohols. *Org. Lett.* **2018**, *21*, 476–480.

[8] Crevier, T. J.; Bennett, B. K.; Soper, J. D. C−N Bond Formation on Addition of Aryl Carbanions to the Electrophilic Nitrido Ligand in $TpOs(N)Cl_2$. *J. Am. Chem. Soc.* **2001**, *123*, 1059–1071.

[9] Fang, G. Y.; Wallner, O. A.; Blasio, N. D. Asymmetric Sulfur Ylide Reactions with Boranes: Scope and Limitations, Mechanism and Understanding. *J. Am. Chem. Soc.* **2007**, *129*, 14632–14639.

[10] Morton, C. M.; Zhu, Q.; Ripberger, H.; Troian–Gautier, L.; Toa, Z. S.; Knowles, R. R.; Alexanian, E. J. C–H Alkylation via Multisite-Proton-Coupled Electron Transfer of an Aliphatic C–H Bond. *J. Am. Chem. Soc.* **2019**, *141*, 13253–13260.

[11] Burés, J. Variable Time Normalization Analysis: General Graphical Elucidation of Reaction Orders from Concentration Profiles. *Angew. Chem. Int. Ed.* **2016**, *55*, 16084–16087.

[12] Docherty, J. H.; Nicholson, K.; Dominey, A. P.; Thomas, S. P. *ACS Catal.* **2020**, *10*, 4686–4691.

[13] Braun, M. *Modern Enolate Chemistry: From Preparation to Applications in Asymmetric Synthesis*, Wiley, 2016.

[14] He, Z. T.; Hartwig, J. F. Palladium-Catalyzed α-Arylation of Carboxylic Acids and Secondary Amides via a Traceless Protecting Strategy. *J. Am. Chem. Soc.* **2019**, *141*, 11749–11753.

[15] Gaussian 16, Revision B.01, Frisch, M. J.; Trucks, G. W.; Schlegel, H. B.; Scuseria, G. E.; Robb, M. A.; Cheeseman, J. R.; Scalmani, G.; Barone, V.; Petersson, G. A.; Nakatsuji, H.; Li, X.; Caricato, M.; Marenich, A. V.; Bloino, J.; Janesko, B. G.; Gomperts, R.; Mennucci, B.; Hratchian, H. P.; Ortiz, J. V.; Izmaylov, A. F.; Sonnenberg, J. L.; Williams-Young, D.; Ding, F.; Lipparini, F.; Egidi, F.; Goings, J.; Peng, B.; Petrone, A.; Henderson, T.; Ranasinghe, D.; Zakrzewski, V. G.; Gao, J.; Rega, N.; Zheng, G.; Liang, W.; Hada, M.; Ehara, M.; Toyota, K.; Fukuda, R.; Hasegawa, J.; Ishida, M.; Nakajima, T.; Honda, Y.; Kitao, O.; Nakai, H.; Vreven, T.; Throssell, K.; Montgomery, J. A., Jr.; Peralta, J. E.; Ogliaro, F.; Bearpark, M. J.; Heyd, J. J.; Brothers,

E. N.; Kudin, K. N.; Staroverov, V. N.; Keith, T. A.; Kobayashi, R.; Normand, J.; Raghavachari, K.; Rendell, A. P.; Burant, J. C.; Iyengar, S. S.; Tomasi, J.; Cossi, M.; Millam, J. M.; Klene, M.; Adamo, C.; Cammi, R.; Ochterski, J. W.; Martin, R. L.; Morokuma, K.; Farkas, O.; Foresman, J. B.; Fox, D. J. Gaussian, Inc., Wallingford CT, 2016.

[16] Texas Advanced Computing Center (TACC), The University of Texas at Austin

[17] Allouche, A. R. *J. Comp. Chem.*, **2011**, *32*, 174–182.

[18] Luchini, G.; Alegre-Requena, J. V.; Funes-Ardoiz, I.; Paton, R. S. GoodVibes: Automated Thermochemistry for Heterogeneous Computational Chemistry Data. *F1000Research*, **2020**, *9*, 291.

[19] Chemcraft - graphical software for visualization of quantum chemistry computations. https://www.chemcraftprog.com

[20] CYLview, 1.0b; Legault, C. Y., Université de Sherbrooke, 2009, http://www.cylview.org

[21] Yu, H. S.; He, X.; Li, S. L.; Truhlar, D. G. *Chem. Sci*. **2016**, *7*, 5032–5051.

[22] Eichkorn, K.; Weigend, F.; Truetler, O.; Ahlrichs, R. *Theor. Chem. Acc.* **1997**, *97*, 119–124.

[23] F. Weigend, *Phys. Chem. Chem. Phys*. **2006**, *8*, 1057-1065.

[24] A. V. Marenich, C. J. Cramer, D. G. Truhlar, *J. Phys. Chem. B.* **2009**, *113*, 6378-6396.

[25] S. Grimme, *Chem-Eur. J*. **2012**, *18*, 9955-9964.

[26] C.-X. Cui, D. Xu, B.-W. Ding, L.-B. Qu, Y.-P. Zhang, Y. Lan. *J. Comput. Chem.* **2019**, *40*, 657–670.

[27] (a) D. K. Deb, B. Sarkar. *Phys. Chem. Chem. Phys.,* **2019**, *21*, 14589–14597. (b) I. R. Piletic, R. Howell, L. J. Bartolotti, T. E. Kleindienst, S. M. Kaushik, E. O. Edney, *J. Phys. Chem. A* **2019**, *123*, 906–919.

[28] N. Mardirossian, M. Head-Gordon, *J. Chem. Theory Comput*. **2016**, *12*, 4303–4325.

[29] K. Eichkorn, F. Weigend, O. Truetler, R. Ahlrichs, *Theor. Chem. Acc.* **1997**, *97*, 119-124.

[30] F. Weigend, Phys. Chem. Chem. Phys. 2006, 8, 1057-1065.

[31] Y. Zhao, D. G. Truhlar, *J. Phys. Chem. A* **2005**, *109*, 5656-5667.

[32] S. Grimme, S. Ehrlich, L. Goerigk, *J. Comp. Chem*. **2011**, *32*, 1456-1465.

[33] Y. Zhao, D. G. Truhlar, *Acc. Chem. Res.* **2008**, *41*, 157-167.

[34] Y. Zhao, D. G. Truhlar, *Theor. Chem. Acc*. **2008**, *120*, 215-241.

[35] S. Grimme, J. Antony, S. Erhrlich, H. Krieg, *J. Chem. Phys*. **2010**, *132*, 154104-19.

[36] Y. Zhao, N. E. Schultz, D. G. Truhlar, *J. Chem. Theory Comput*. **2006**, *2*, 364-382.

[37] A. D. Becke, *J. Chem. Phys*. **1993**, *98*, 5648-5652.

[38] C. Lee, W. Yang, R. G Parr, *Phys. Rev. B* **1998**, *37*, 785-789.

[39] P. J. Stephens, F. J. Devlin, C. F. Chabalowski, M. J. Frisch, *J. Phys. Chem.* **1994**, *95*, 11623-11627.

[40] J. P. Perdew, M. Ernzerhof, *J. Chem. Phys*. **1996**, *105*, 9982-9985.

[41] C. Adamo, B. Vincenzo, *J. Chem. Phys*, **1999**, *110*, 6158-6170.

[42] J.-D. Chai, M. Head-Gordon, *Phys. Chem. Chem. Phys*. **2008**, *10*, 6615-6620.

[43] R. Peverati, D. G. Truhlar, *J. Phys. Chem. Lett.* **2011**, *2*, 2810-2817.
